# Designing Antibiotics with Inherent Resistance to Efflux as a Strategy to Revive Discovery against Multidrug-Resistant Pathogens

**DOI:** 10.1021/acs.jmedchem.6c00060

**Published:** 2026-05-29

**Authors:** Mark Laws, Charlotte K. Hind, Kazi Sharmin Nahar, Melanie Clifford, Caleb Marsh, Taha al Adhami, Brice Louis, Manming Xu, Saleh O. Alyemni, Shozeb Haider, Mushtaq Hassan, Nupur Gargate, Matthew E. Wand, J. Mark Sutton, Khondaker Miraz Rahman

**Affiliations:** † School of Cancer and Pharmaceutical Sciences, 4616 King’s College London, Franklin-Wilkins Building, 150 Stamford Street, London SE1 9NH, U.K.; ‡ Countermeasures Development, Evaluation and Preparedness, Public Health Microbiology, 164131 UK Health Security Agency, Manor Farm Road, Porton Down, Salisbury SP4 0JG, U.K.; § Department of Natural Sciences, University of Middlesex, The Burroughs Hendon, London NW4 4BT, U.K.; ∥ UCL School of Pharmacy, 371646 University College London, London WC1N 1AX, U.K.

## Abstract

We report a novel Efflux Resistance Breaker (ERB) strategy for designing antibiotics intrinsically resistant to efflux, using fluoroquinolones as a model class. ERB-modified fluoroquinolones showed enhanced intracellular accumulation and markedly improved antibacterial activity, with up to 512-fold reduction in MIC (MIC_90_ 0.03–2 μg/mL) across multidrug-resistant bacteria. Lead compounds **KSN-L22** (**46**) and **BL-7** (**50**) demonstrated potent activity against MRSA, *Streptococcus pneumoniae* (including MDR and PRSP), *Enterococcus faecalis* and *E. faecium* (VanA, VanB and VanD), as well as *Acinetobacter baumannii* and *Escherichia coli*. The compounds inhibited both wild-type and S84L mutant DNA gyrase (IC_50_ ∼ 3.8 μg/mL) and achieved a > 4-log bacterial load reduction in a murine thigh infection model at oral doses of 50 mg/kg. Favorable oral and intravenous PK/PD profiles, absence of toxicity at 1200 mg/kg/day, and no hERG, CYP450, or off-target liabilities were observed. ERB technology provides a promising strategy for designing antibiotics that are intrinsically less susceptible to efflux.

## Graphical abstract

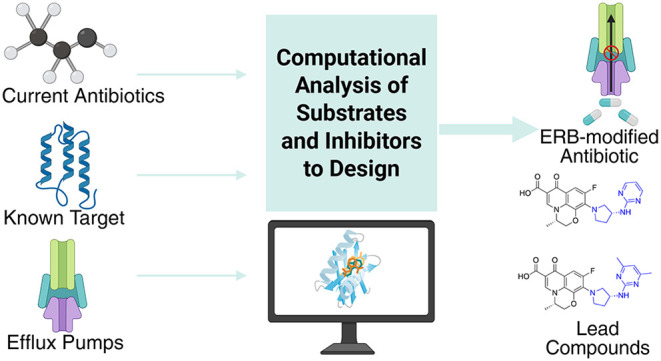

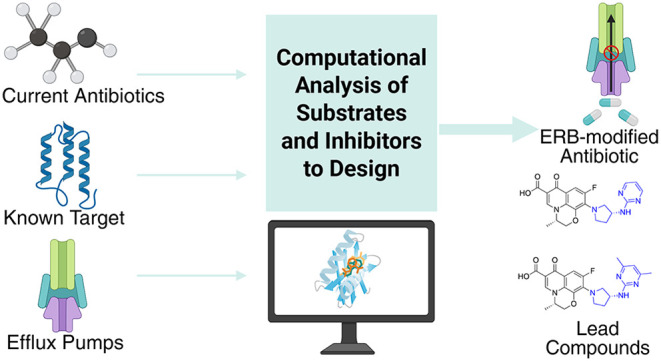

## Introduction

The introduction of antibiotics into clinical practice was one of the major achievements of the 20th century. However, these advances are now threatened by the global increase in antimicrobial resistance (AMR). The development and approval of new antibiotics are currently being outpaced by the emergence of resistance to existing drugs, a trend that must be reversed to ensure the long-term effectiveness of antibiotics.^1^ Prokaryotic efflux systems are key mechanisms of antibiotic resistance in a variety of bacterial species. These pumps have, in the case of quinolone resistance in *Staphylococcus aureus*, been shown to act as mediators of an initial, intermediate resistance phenotype from which higher level resistance mutations can then stem.^2,3^ Similar observations have been made for quinolone resistance in *Escherichia coli* ^4^ and *Streptococcus pneumoniae*,^5^ azithromycin and ethambutol resistance in *Mycobacterium* spp.,^6,7^ and metronidazole resistance in *Helicobacter pylori*.^8^ Antibiotic development programs have long sought to minimize efflux by selecting candidate compounds that are poor efflux substrates,^9^ but recent reports of efflux-mediated resistance to omadacycline,^10^ eravacycline,^10–12^ and delafloxacin,^13^ three relatively new antibiotics approved by the FDA between 2017 and 2018, indicate that this approach may be untenable long-term.

Efflux pump inhibitors (EPIs) have been pursued as adjunct therapies to safeguard approved antibiotics prone to efflux-based resistance. There has been a considerable body of research by different academic groups and pharmaceutical companies directed toward the development of EPIs,^14–16^ but no EPI has yet achieved market approval.^17^ As well as a lack of mechanistic insight and biochemical information regarding efflux pumps, we opine that this failure is rooted in a fundamental flaw in the EPI-antibiotic combination approach: that the antibiotics remain unmodified substrates and can be effluxed by different pumps despite the presence of EPIs. The intracellular concentration of EPIs also needs to be considerably higher to increase the probability of sparing the antimicrobials, but this has led to toxicity that has prevented preclinical or clinical development of EPIs. There are additional complexities around matched pharmacokinetics in combination therapy which served as an additional barrier for EPI development.^18^ In addition to combination therapy, the direct conjugation of EPIs to antibiotics has been explored with some limited success, but the efflux-inhibitory character of such conjugates typically comes at the expense of reduced antibiotic activity and/or unsuitability for further development, often due to their high molecular weight.^19–22^

Herein, we report a novel approach that both restores the activity of antibiotics that suffer efflux-mediated resistance and guides the design of new efflux-resistant antibiotics. We covalently modified antibiotic scaffolds with small fragments, termed efflux resistance breaker (ERB) fragments, which direct the compounds to inhibitor-binding pockets within efflux pumps and reduce their susceptibility to efflux. This strategy allows the ERB-antibiotics to act as substrate inhibitors of the pumps, blocking their own export from the bacterial cell and leading to high intracellular drug concentrations. As a result, bacterial growth is inhibited or the pathogen is killed, even in the presence of multiple target mutations that would normally confer resistance. Unlike traditional antibiotic-EPI conjugates, this strategy does not rely on attaching known EPI scaffolds. We validated the approach using fluoroquinolones as model antibiotics since efflux-mediated resistance is well established for this class. This strategy therefore represents a clear departure from previous unsuccessful efforts to develop EPIs.

We have shown that the ERB-modified fluoroquinolones retain target specificity for DNA gyrase in both Gram-positive and Gram-negative bacteria, with a significantly lower IC_50_ against the fluoroquinolone-resistant mutant enzyme, GyrA S84L, than the parent antibiotic **levofloxacin**. We have evidence that these compounds also act as EPIs at subinhibitory concentrations, potentiating the activity of other antibiotics that are effluxed through the same range of pumps, providing dual activity. Our microbiological data show that these ERB-fluoroquinolones overcome high levels of preexisting fluoroquinolone resistance and do not show any of the adverse safety signals^12,13^ often associated with fluoroquinolones. The *in vitro* DMPK, *in vivo* PK, toxicity, and *in vivo* efficacy data of ERB-fluoroquinolones suggest that this approach is suitable to develop new-generation antibacterials capable of overcoming efflux-mediated resistance.

## Results

### Identification of Substrate and Inhibitor Binding Pockets within Efflux Pumps

In our efforts to develop efflux-resistant antimicrobials, initially we sought to address the differential binding response to substrates and inhibitors by investigating the differences in efflux pump behavior in the presence of substrates and inhibitors.^23^ We have previously reported changes in efflux pump dynamics in response to substrates and inhibitors that affect the likelihood of a molecule being effluxed.^23^ We studied a large number of efflux pump substrates and inhibitors to determine their preferred binding site within the efflux transporter. To do this, we developed a series of molecular models of key efflux transporters from members of the ESKAPEE pathogens including NorA in *S. aureus*, AdeB in *Acinetobacter baumannii*, and MexB in *Pseudomonas aeruginosa*. Models were developed based on a combination of X-ray crystallography data, AlphaFold 3.0, and molecular dynamics simulations. Libraries of known substrates and inhibitors were subsequently docked against each model, with the goal of identifying any differences between the two groups.

A clear pattern emerged for all major efflux pumps studied; substrates and inhibitors were predicted to consistently bind within two different, adjacent internal pockets (Figure S1). As exemplified by the MFS-type pump NorA from *S. aureus* and the RND-type pump MexB from *P. aeruginosa*, hydrophobicity maps of pump internal surfaces showed the substrate binding sites to be more hydrophilic than the corresponding inhibitor binding sites. This observation suggested that efflux-prone antibiotics could be rendered less susceptible to efflux by directing them toward the more hydrophobic internal binding surfaces of the target efflux pumps, which are typically occupied by EPIs.^24–27^ We have exploited this differential binding behavior to chemically modify antibiotics with ERB fragments that make them less susceptible to efflux while maintaining their antibacterial activity.

### Chemical Modification of Fluoroquinolones to Develop First-Generation Efflux-Resistant Antibiotics

The second-generation fluoroquinolone **ciprofloxacin (1)** was selected as a representative efflux-prone antibiotic upon which to test this concept. The NorA efflux pump in *S. aureus* was chosen as a model system because it is known to contribute to fluoroquinolone resistance in this species.^28,29^ Docking studies with the NorA efflux pump predicted the C7 and C8 positions of the ciprofloxacin core to bind adjacent to the inhibitor binding site, making them suitable positions for attaching an ERB fragment. An *in silico* screen of approximately nine million fragments from the ZINC library^30^ was carried out against NorA using high-performance computing resources at King’s College London and the DockBlaster web server. This screen identified top-ranked hits that were enriched in hydrophobic, polycyclic motifs. Based on common features among the highest-scoring fragments and previous reports of efflux pump inhibition, a synthetically accessible surrogate, 1-(naphthalen-1-ylmethyl)­piperazine (**NMP**), was selected as the ERB fragment (Table S1, Figure S2). Regioisomeric ciprofloxacin-ERB conjugates were subsequently designed with ERB fragments attached at positions adjacent to the inhibitor binding site, specifically C7, the C7 piperazine ring, and C8. The C7-piperazine-substituted regioisomers **ML-77-005 (8)** (Scheme 1) and **12** (Scheme 2) and C8-substituted regioisomer **19** (Scheme 3) were synthesized and evaluated against a panel of Gram-positive bacteria, including NorA-overexpressing *S. aureus* SA-1199B and its wild-type counterpart SA-1199 ^31^ (Table 1).

**Scheme 1:**
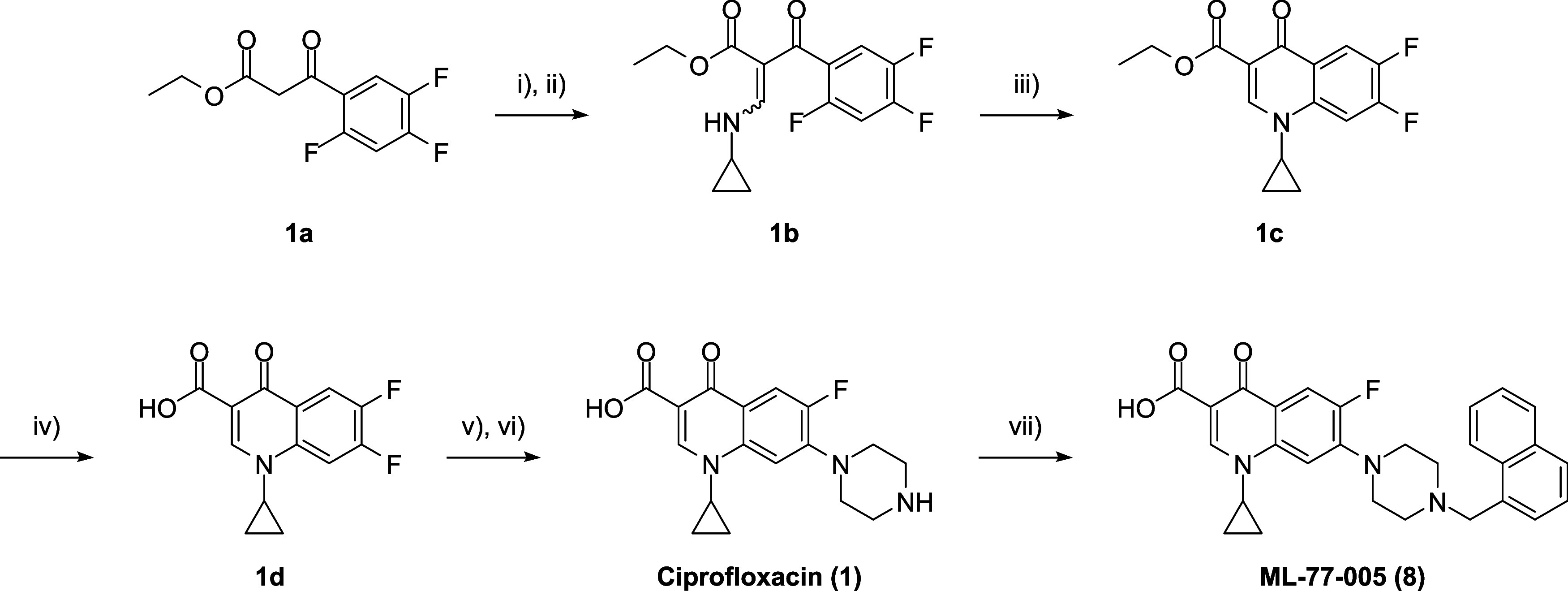
Synthesis of C7-Piperazine-Substituted Regioisomer **ML-77-005 (8)**. (i) (EtO)_3_CH, Ac_2_O, 140 °C. (ii) Cyclopropylamine, DCM, RT. (iii) DBU, LiCl, DCM, 45 °C. (iv) HCl, AcOH, reflux. (v) Boc-Piperazine, K_2_CO_3_, DMF, Reflux. vi) TFA, Dry DCM, RT. vii) 1-(Bromomethyl)­naphthalene, K_2_CO_3_, H_2_O/MeCN (1:1), RT. We Have Previously Reported Steps i–vi.^32^

**Scheme 2:**
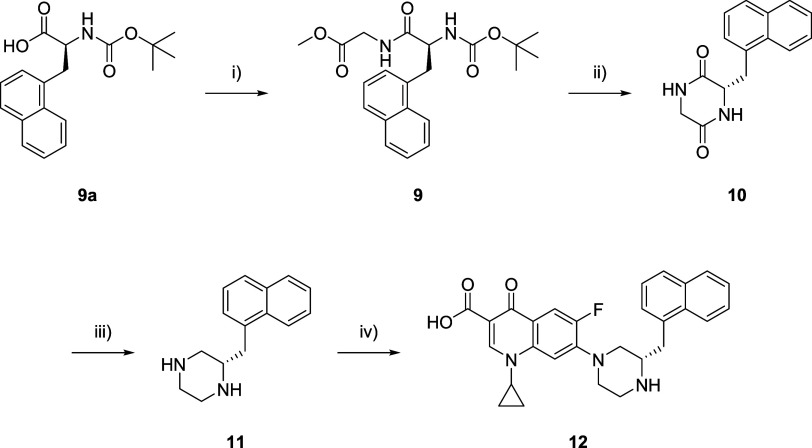
Synthesis of C7-Piperazine Regioisomer **12**. (i) HATU, DIPEA, CH_2_Cl_2_, 0 °C; Methyl Glycinate Hydrochloride, 0 °C–RT. (ii) MeOH/H_2_O (1:1), Microwave, 200 °C. (iii) LiAlH_4_, THF, 0 °C–RT. (iv) 1-Cyclopropyl-6,7-difluoro-4-oxo-1,4-dihydroquinoline-3-carboxylic acid, DMSO, Microwave, 140 °C

**Scheme 3:**
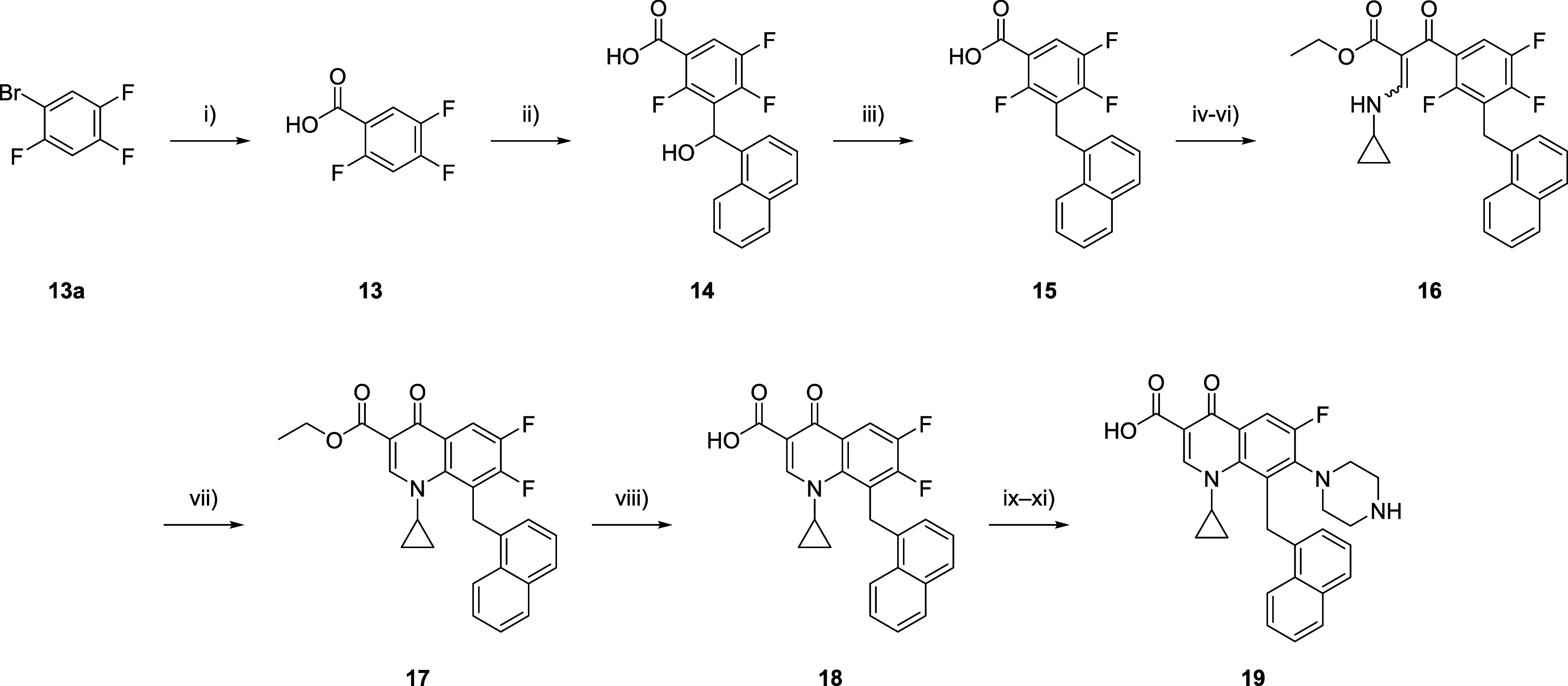
Synthetic Procedure for the Regioselective Synthesis of **19**. (i) *n*-BuLi, Et_2_O, −78 °C; CO_2_. (ii) *n*-BuLi, 1-Naphthaldehyde, THF, −78 °C. (iii) NaBH_4_, TFA, DCM, −10 °C. (iv) (COCl)_2_, Cat. DMF, DCM, 0 °C. (v) Ethyl (*E*)-3-(dimethylamino)­acrylate, Et_3_N, Toluene, 90 °C. vi) Cyclopropylamine, DCM, RT. vii) K_2_CO_3_, DMF, 90 °C. viii) 15% w/v NaOH, EtOH, RT. ix) BF_3_·Et_2_O, K_2_CO_3_, THF, Reflux. x) Piperazine, MeCN, 50 °C. xi) Et_3_N, EtOH, Reflux

**Table 1:** Antibacterial Activity of Ciprofloxacin and ERB-Fluoroquinolone Regioisomers against Gram-Positive Bacterial Strains^a^

strain		MIC, μg/mL			
		**Ciprofloxacin**	**8**	**12**	**19**
*S. aureus*	ATCC 9144	0.25	0.25	4	64
	NCTC 13616	128	2	8	32
	NCTC 13277	128	2	8	32
	USA300	128	2	8–16	32
	SA-1199	0.25	0.125	2	16
	SA-1199B	1	0.125	4	16
*E. faecalis*	NCTC 775	1	2	8	16
	NCTC 12201	0.5	0.125	8	16
	NCTC 12204	1	2	8	16

a MICs are reported in μg/mL.

The synthetic route to **8** built upon a well-reported synthesis of the commercial fluoroquinolone **ciprofloxacin** and required only one additional alkylation step. However, expeditious synthesis of the other regioisomers, with their ERB units appended at less synthetically amenable positions, proved more challenging. **12** was accessed through formation and reduction of key diketopiperazine intermediate **10** before a final S_N_Ar reaction furnished the completed regioisomer. Separately, **19** was synthesized from a polyhalogenated arene, mirroring another well-established route to ciprofloxacin, with a final regioselective S_N_Ar reaction—utilizing a borate complex to increase the electron-withdrawing character of the C4 ketone in **18** and promote nucleophilic attack at the opposite C7 position—inspired by the work of Cecchetti et al.^33^

Biological evaluation of this set of regioisomers showed that modification at the C7 piperazine position was optimal for overcoming NorA-mediated efflux. Among the regioisomers, **8**, with its ERB fragment located on the terminal C7 piperazine nitrogen, displayed the most favorable activity profile and achieved low minimum inhibitory concentration (MIC) values against NorA upregulated strains, including *S. aureus* NCTC 13616, NCTC 13277, USA300, and SA-1199B. In these strains where **1** was inactive or showed markedly reduced activity, the excellent potency of **8** was consistent with reduced efflux liability and increased intracellular accumulation. The C7 piperazine carbon regioisomer **12** displayed moderate activity, while the C8-substituted regioisomer **19** was uniformly weak, indicating suboptimal positioning of the ERB fragment. These data thus established **8** as a proof-of-concept ERB-fluoroquinolone and identified the terminal C7 piperazine nitrogen as the preferred site for ERB attachment, providing a robust foundation for subsequent SAR optimization.

Structurally, **8** can be considered as a truncated hybrid of **ciprofloxacin** and **NMP**. **NMP** has been reported to potentiate fluoroquinolones^34^ against the RND-type pumps AcrAB and AcrEF in *E. coli*,^34^ thus **8** was compared to both **ciprofloxacin** alone and a **ciprofloxacin**/**NMP** combination *in vitro* against different strains of *S. aureus*. While **NMP** afforded only a 2-fold potentiation of ciprofloxacin in fluoroquinolone-resistant strains NCTC 13616 and NCTC 13277, **8** showed 64-fold improvement over **ciprofloxacin** alone and retained comparable activity in fluoroquinolone-susceptible strain ATCC 9144 (Table S2). Further minimum inhibitory concentration (MIC) testing against a panel of an additional 12 *S. aureus* strains, the majority of which possessed point mutations in both *gyrA* and *grlA*, confirmed the superiority of **8** (MIC_90_ 2 μg/mL) versus **ciprofloxacin** (MIC_90_ 128 μg/mL) with up to 64-fold potentiation observed for the ERB-fluoroquinolone compound (Table S3). These results indicate that covalent attachment of an ERB fragment to an antibiotic, thereby directing it to hydrophobic binding pockets typically occupied by EPIs (Figure S3), is substantially more effective than coadministration of a free inhibitor or fragment with the antibiotic, which represents the traditional approach. To our knowledge, the extent of resistance reversal observed here exceeds that previously reported for antibiotic-EPI combination strategies.

To probe the structure–activity relationship of **8**, a series of analogues were synthesized with modifications to the ERB fragment structure (Table S4). These analogues were tested against an informative panel of *S. aureus* isolates including two fluoroquinolone-susceptible strains (ATCC 9144 and SA-1199), an isogenic mutant of SA-1199 having upregulated NorA expression (SA-1199B),^35^ and two MRSA strains (NCTC 13616 and NCTC 13277) which have both upregulated NorA expression and mutations in the antibiotic target DNA gyrase. The ERB fragment modifications focused on fragment hydrophobicity by introducing different heteroatoms and functional groups that affect the ability of the generated analogues to interact with hydrophobic residues within the NorA efflux pump. From this, it was concluded that ERB unit hydrophobicity was a key determinant of ERB-fluoroquinolone activity. Compared to **8**, analogues lacking a second aromatic ring (**25**) or possessing an aromatic heterocycle (**27**) showed much reduced activity against fluoroquinolone-resistant *S. aureus*, while analogues with modifications involving the orientation of the naphthalene moiety (**23**) and partial removal of its aromaticity (**29**) retained this activity. Based on these results, further analogues of **8** were synthesized (Table S4); the activities of each vs fluoroquinolone-resistant *S. aureus* further confirmed the importance of ERB unit hydrophobicity, while analogues based on the commercial fluoroquinolones **norfloxacin** (**2**), **enoxacin** (**3**), and **levofloxacin** (**7**) confirmed the wider applicability of the ERB-fluoroquinolone approach. In each case, compounds modified with the ERB approach were found to be significantly more active compared to the unmodified parent antibiotics.

### Determination of Efflux Susceptibility of ERB-Modified Ciprofloxacin Derivative **ML-77-005 (8)**

To experimentally validate the efflux susceptibility of the **ciprofloxacin** analogues modified using the ERB approach and determine the origin of the improved activity seen in **ML-77-005 (8)**, a reserpine growth assay was performed using *S. aureus* strain NCTC 13616 (Figure 1a, Figure S4).

**Figure 1:**
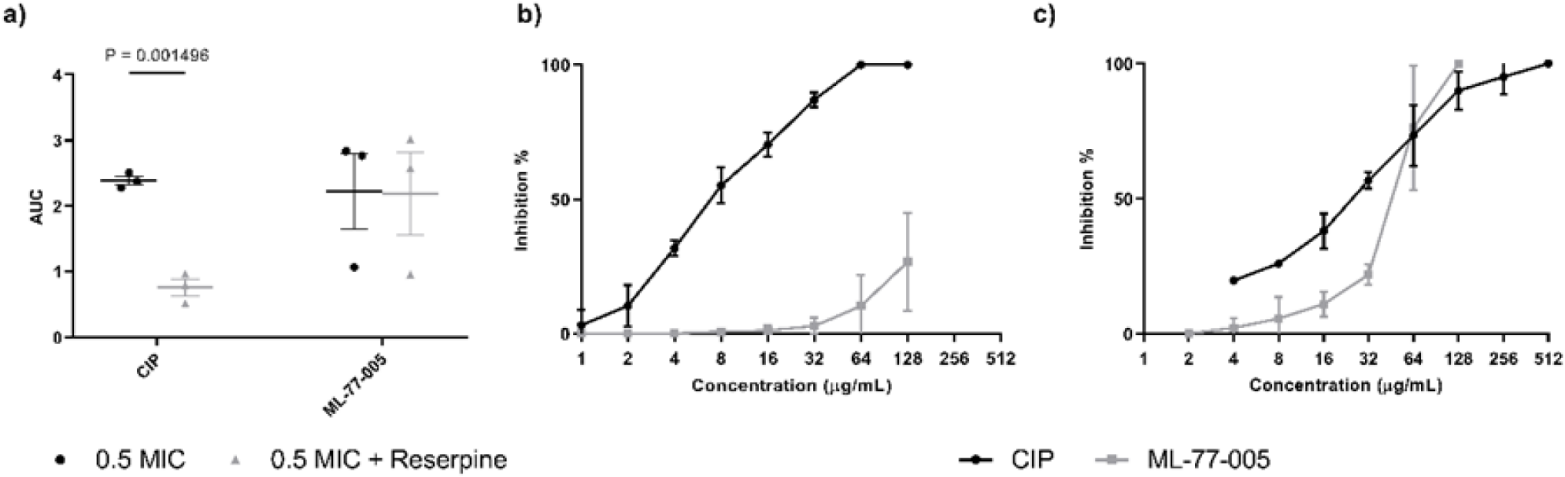
**a**), Reserpine assay data for **ciprofloxacin** (**1, CIP**) and first-generation ERB-fluoroquinolone **ML-77-005 (8)** in NorA overexpressing strain NCTC 13616. The reserpine assay uses reserpine, a natural product and promiscuous competitive EPI, to determine if an antibiotic is subject to efflux from *S. aureus* or *S. pneumoniae*.^37^ Each point represents a biological replicate, with the bar displaying the mean area under the curve (AUC) and the error bars displaying the SEM. Multiple unpaired *t* tests with Welch correction were used to compare the AUC in the presence of 0.5x MIC to the presence of 0.5x MIC with reserpine. A significant difference between the means was found only for **CIP** (*p* = 0.001496). NorA is inhibited in the presence of reserpine, therefore the significant reduction observed in AUC in the presence of CIP with reserpine suggests that **CIP** is a substrate of NorA. These data suggest that **8** is not a substrate of NorA, as no significant difference was observed in AUC when NorA was inhibited by reserpine. **b–c**) Topoisomerase supercoiling assay data for **CIP** and **8** using both **b**) WT and **c**) the S84L mutant DNA gyrase enzyme. The assay combines purified topoisomerase enzyme with relaxed plasmid DNA, with the enzyme introducing negative supercoils into the DNA unless an inhibitor prevents this; an IC_50_ can be calculated through comparison of experiments with different inhibitor concentrations. **8** has the higher IC_50_ of the two compounds against both WT and S84L enzymes (>128 and 44.76 ± 3.2 μg/mL, respectively, compared to 7.53 and 34.69 ± 7.01 μg/mL for **CIP**), thus improved target inhibition is not the cause of its superior activity in *S. aureus*.

**Ciprofloxacin** at sub-MIC concentrations was potentiated by the EPI reserpine, evidenced by a statistically significant difference (*P* = 0.002993) in the AUC of the growth curves and indicating, as previously reported,^36^ that it is an efflux substrate. However, no such potentiation was observed for **8**, inferring that it is not subject to efflux from *S. aureus*. This suggests that **8** is able to maintain high intracellular concentrations in the presence of significant NorA upregulation in fluoroquinolone-resistant *S. aureus* strains and hence is able to achieve far lower MIC values than **ciprofloxacin** (Table S3). This evidence, combined with the MIC results in the isogenic strains SA-1199 and SA-1199B with differing NorA expressions, strongly shows the efflux-resistant potential of antibiotics modified with the ERB approach compared to unmodified antibiotics.

### Type II Topoisomerase Inhibitory Activity of **ML-77-005**

One of the fundamental considerations during the ERB modification of antibiotics was to ensure they maintained on-target activity so that they could still work as an antibiotic while preventing their own efflux. Commercial antibacterial quinolones work by inhibiting type II topoisomerase enzymes.^38^ A molecular modeling experiment suggested **ML-77-005 (8)** binds to the same pocket as **ciprofloxacin** (Figure S5). A cell-free DNA gyrase inhibition assay was used to assess the activity of **8** against enzymes from both fluoroquinolone-sensitive (WT DNA gyrase) and fluoroquinolone-resistant (GyrA S84L mutant enzyme) *S. aureus* strains (Figure 1b and 1c, respectively). While **8** showed reduced inhibition of the WT enzyme compared to **ciprofloxacin**, the difference in IC_50_s of both against the S84L mutant enzyme was statistically insignificant. Notably, while the S84L mutant enzyme IC_50_ for **ciprofloxacin** (34.69 ± 7.01 μg/mL) was broadly comparable to the IC_50_ for **8** (44.76 ± 3.2 μg/mL), **8** was found to be 20 to 64-fold more phenotypically active against fluoroquinolone-resistant strains (*n* = 11 strains; MICs for **8** 2 μg/mL, MICs for **ciprofloxacin** 32–128 μg/mL) (Figure 1C, Tables S3, S4). Together, these data indicate that **8** improves upon **ciprofloxacin** in fluoroquinolone-resistant strains of *S. aureus* through resisting the action of efflux pumps such as NorA rather than by improved inhibition of type II topoisomerase enzymes. **8** also had comparable time-kill kinetics to **ciprofloxacin** in *S. aureus* strain NCTC 13616 (Figure S6).

### Resistance to **ML-77-005 (8)**

Serial passaging experiments with **ML-77-005 (8)** were carried out using both fluoroquinolone-susceptible and fluoroquinolone-resistant *S. aureus* strains to generate resistant mutants. Whole genome sequencing of the resulting mutants revealed no efflux-related mutations in either strain of *S. aureus* used; instead, a pair of previously reported target mutations (GyrA D83G,^39^ GrlA S80F^40^) as well as several off-target mutations were discovered in the mutant ATCC 9144, with no stable mutations found in the mutant NCTC 13616 (Table S5). These data indicate that ERB-fluoroquinolones, while vulnerable to the same resistance mutations as conventional fluoroquinolones, are not affected by any novel mechanisms of resistance. The difficulty in generating highly resistant mutants of *S. aureus* NCTC 13616, already resistant to commercial fluoroquinolones, may also indicate a slowed onset of resistance for an ERB-fluoroquinolone agent vs a conventional fluoroquinolone antibiotic.

### Development of Second-Generation ERB-Fluoroquinolones

Despite its strong activity against Gram-positive bacteria, **ML-77-005 (8)** showed poor aqueous solubility (<5 μM) and very high plasma protein binding (>99%). These properties are attributed to the high hydrophobicity of the naphthalene moiety. More importantly, **8** was inactive against WHO- and CDC-priority Gram-negative pathogens, including *A. baumannii* (Table S6), limiting its use for evaluating efflux susceptibility in Gram-negative bacteria.

To understand the loss of activity relative to **ciprofloxacin**, MICs were determined in Gram-negative strains in the presence and absence of the outer membrane permeabilizing agent polymyxin B nonapeptide (PMBN). Because the outer membrane of Gram-negative bacteria limits the uptake of hydrophobic compounds, PMBN was expected to improve the permeability and lower MIC values. In all Gram-negative strains tested, the addition of PMBN restored the activity of **8** to levels comparable with **ciprofloxacin** (Table S6). These results suggested that the hydrophobic ERB moiety prevented efficient permeation and intracellular accumulation in Gram-negative bacteria. The data showed that second-generation ERB-fluoroquinolones would require improved physicochemical properties, including both a more polar fluoroquinolone core and a more polar ERB fragment, to achieve acceptable activity against Gram-negative pathogens.

Our analysis of physicochemical properties of ERB-modified fluoroquinolones using SwissADME and ADMETLab 3.0 identified the third-generation fluoroquinolone **levofloxacin** core as the most balanced scaffold for ERB modification and as a suitable fluoroquinolone with superior physicochemical properties compared to ERB-modified **ciprofloxacin** (Tables S7 and S8). Although not optimal in every individual descriptor, it combined moderate molecular weight, balanced polarity and lipophilicity, the lowest conformational flexibility and rotatable bond count, acceptable predicted solubility, and no structural alerts. Its low conformational flexibility was considered particularly advantageous because it was expected to present the appended ERB fragment in a more defined orientation, thereby improving projection into the desired hydrophobic inhibitor-binding pocket of the efflux pump and reducing adoption of less favorable conformations.

The second-generation ERB fragments were designed using three main criteria: (i) retention of sufficient hydrophobic character to engage the inhibitor-binding pocket of NorA and related efflux pumps, which is enriched in hydrophobic residues and typically accommodates inhibitor-like ligands; (ii) optimization of the angle and geometry of attachment to direct the terminal heteroaromatic group more effectively into this pocket; and (iii) improvement of overall physicochemical balance, through incorporation of heteroatoms and selection of an appropriate fluoroquinolone core, to reduce the excessive hydrophobicity, poor solubility, high plasma protein binding, and limited Gram-negative permeation associated with the first-generation naphthalene-containing ERB fragment. These considerations led to prioritization of the **levofloxacin** core and pyrrolidine- and aminopyrimidine-based ERB motifs. Molecular modeling studies investigating the interaction of **levofloxacin** with the NorA efflux pump suggested that replacing the piperazine moiety with a pyrrolidine ring and introducing an exocyclic amine group would enable greater contact between the terminal aromatic/heteroaromatic group and the hydrophobic inhibitor-binding pocket of the NorA efflux pump. This provided additional flexibility to the ERB-modified **levofloxacin** and allowed the terminal pyrimidine ring to rotate to ensure optimum contact with the key residues (Phe140, Ile244, Phe303, and Phe306 for NorA), which was not possible with the more rigid linear six-membered piperazine-linked ERB fragment (Figure 2, Figure S7).

**Figure 2:**
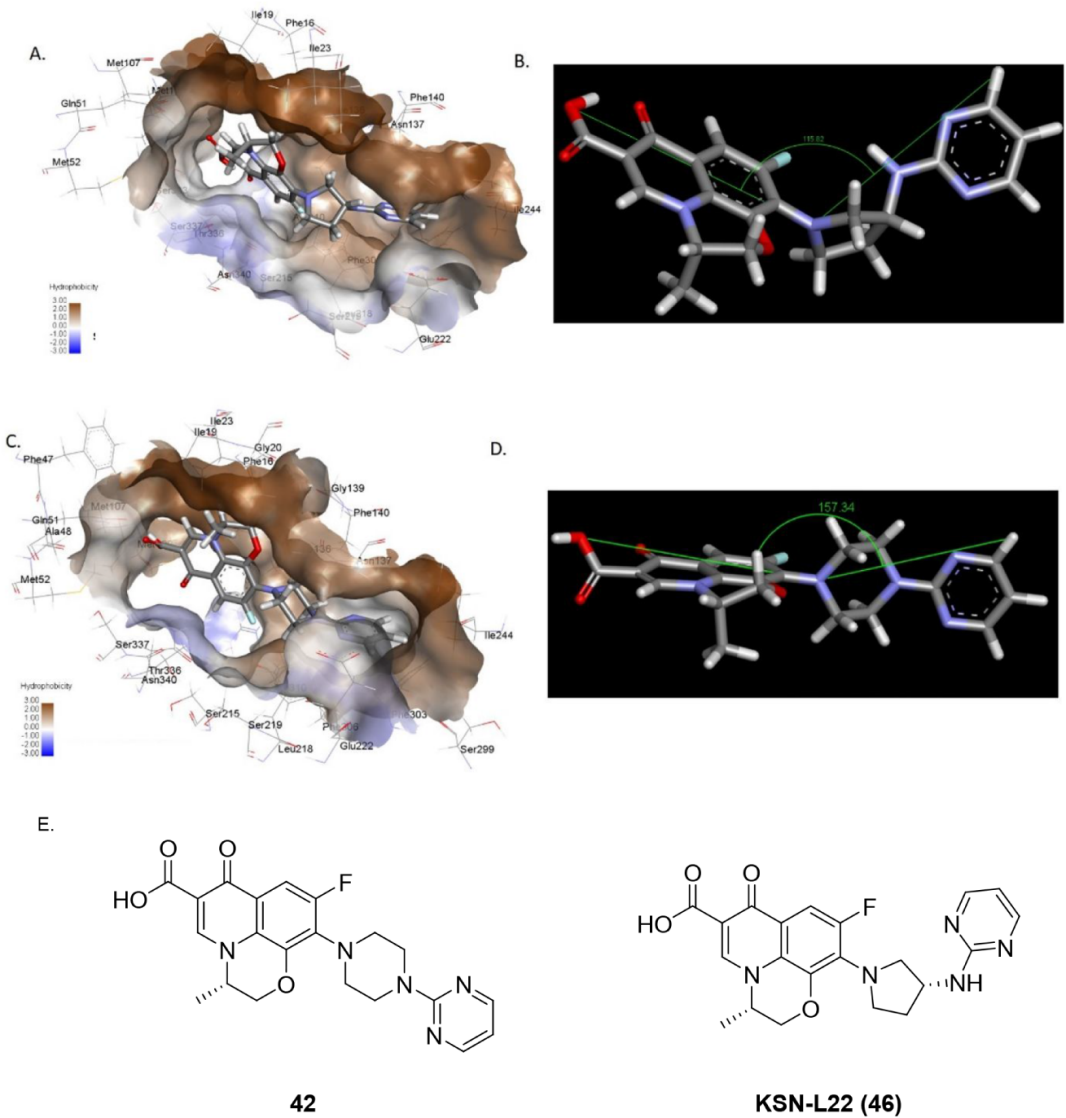
**A**) **KSN-L22 (46)** interacting with the key residues (Phe140, Ile244, Phe303, and Phe306) in NorA (PDB ID: 7LO8), **B**) the five-membered pyrrolidine ring with exocyclic amine group provides additional flexibility and curvature, **C–D**) the relatively linear structure of six-membered piperazine ring-containing **42** does not interact efficiently with the key hydrophobic residues, **E**) structures of lead second generation ERB-fluoroquinolone **42** and **46**.

To experimentally validate this observation, we synthesized the **levofloxacin core** (**6**) using a 4-step published synthetic procedure^41,42^ (Scheme 4). This core was used to generate **42** and **43** via single-step S_N_Ar functionalization (Scheme 4, Table 2). The improvement in activity observed for **42** compared to **levofloxacin (7)** against NorA-overexpressing MRSA strains was negligible. When tested in the isogenic strains SA-1199 and SA-1199B, the MIC for **42** was 4-fold higher in the NorA-overexpressing strain SA-1199B (Table 2).

**Scheme 4:**
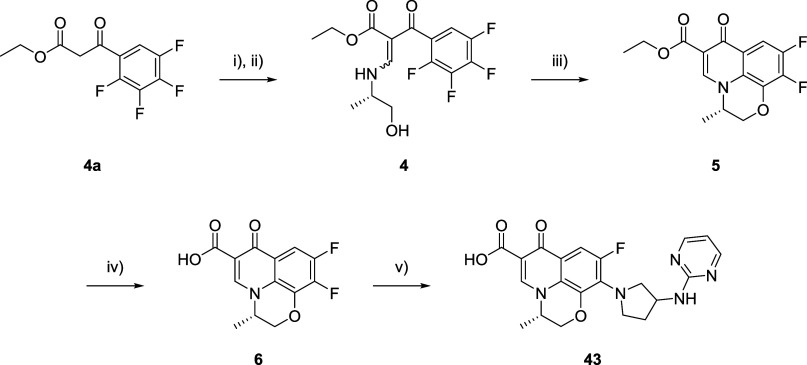
Synthesis of **Levofloxacin** Core **6** and Second Generation ERB-Fluoroquinolone **43**. (i) (EtO)_3_CH, Ac_2_O, 140 °C, 40 h. (ii) l-alaninol, DCM, RT, 96 h. (iii) K_2_CO_3_, DMAc, Microwave, 160 °C, 20 min (iv) 15% w/v NaOH, EtOH, RT, 1 h. (v) *N*-(Pyrrolidin-3-yl)­pyrimidin-2-amine, DMSO, Microwave, 140 °C, 30 min

**Table 2:**
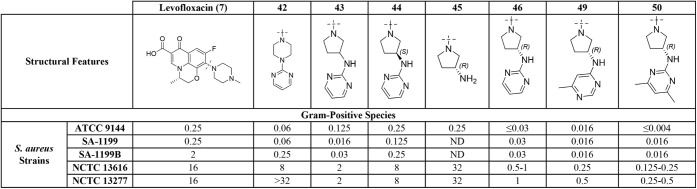
MIC Values for Second-Generation ERB-Fluoroquinolones against *S. aureus* Strains

On the other hand, the racemic compound **43** (Scheme 4) showed 8-fold potentiation compared to **levofloxacin** against fluoroquinolone-resistant NCTC 13616 and NCTC 13277 strains. It had an MIC only 2-fold higher in SA-1199B compared to SA-1199 and did not demonstrate efflux liability in the reserpine assay (Figure 3, Figure S8A). Therefore, we synthesized both diastereomers of **43** to identify which of them could form optimum contact with the key residues within the binding pocket. Compound **44**, the (S, S)-diastereomer, appeared to be less active compared to the (S, R)-diastereomer **KSN-L22 (46)** (Figure 2E, Table 2). Compound **46** was 16–32-fold more active against fluoroquinolone-resistant strains compared to **levofloxacin** with MICs of 0.5 and 1 μg/mL, while **44** showed only 2–4-fold potentiation against fluoroquinolone-resistant strains, suggesting the orientation of the terminal pyrimidine ring plays an important role in reducing efflux susceptibility and activity of the compound. To further probe the role of the terminal pyrimidine ring in reducing efflux susceptibility and antibacterial activity, we synthesized a series of analogues of **46** incorporating variations on the 2-aminopyrimidine ring by changing the position of nitrogen atoms in the aromatic ring (**48**), introducing fluorine (**47**), monomethyl substitution (**49**), dimethyl substitution (**50**) and finally removing the pyrimidine ring altogether (**45**) (Table S9 and Table 2). As expected, removal of the pyrimidine ring (**45**) removed the critical contact within the hydrophobic binding pocket of the NorA efflux pump, resulting in 32- to 64-fold loss of activity compared to **46**. Notably, the 4,6-dimethyl substituted analogue **BL-7 (50)** (Figure 2F) was found to achieve a 2–8-fold improvement in activity in strains of fluoroquinolone-resistant *S. aureus* compared to **46,** while other pyrimidine-ring containing analogues maintained activity similar to that of **46** in these strains. These data suggest that both components of the ERB fragment, the heteroaliphatic ring and the terminal heteroaromatic ring, are important for making the compounds less susceptible to efflux and overcoming efflux-mediated resistance. The observation that the dimethyl substitution present in **50** resulted in improved activity in all strains compared to **46** further outlines the importance of targeted hydrophobic contact with the efflux pump in reducing efflux susceptibility. A **moxifloxacin** core-based analogue **ML-110-014 (51)** (Table S9), containing the same ERB fragment as **46**, showed MIC values similar to **46** and was found to be 4–8-fold more active than **moxifloxacin** against fluoroquinolone-resistant strains. This further showed the ability of the ERB approach to reduce the efflux liability of fluoroquinolone antibiotics.

**Figure 3:**
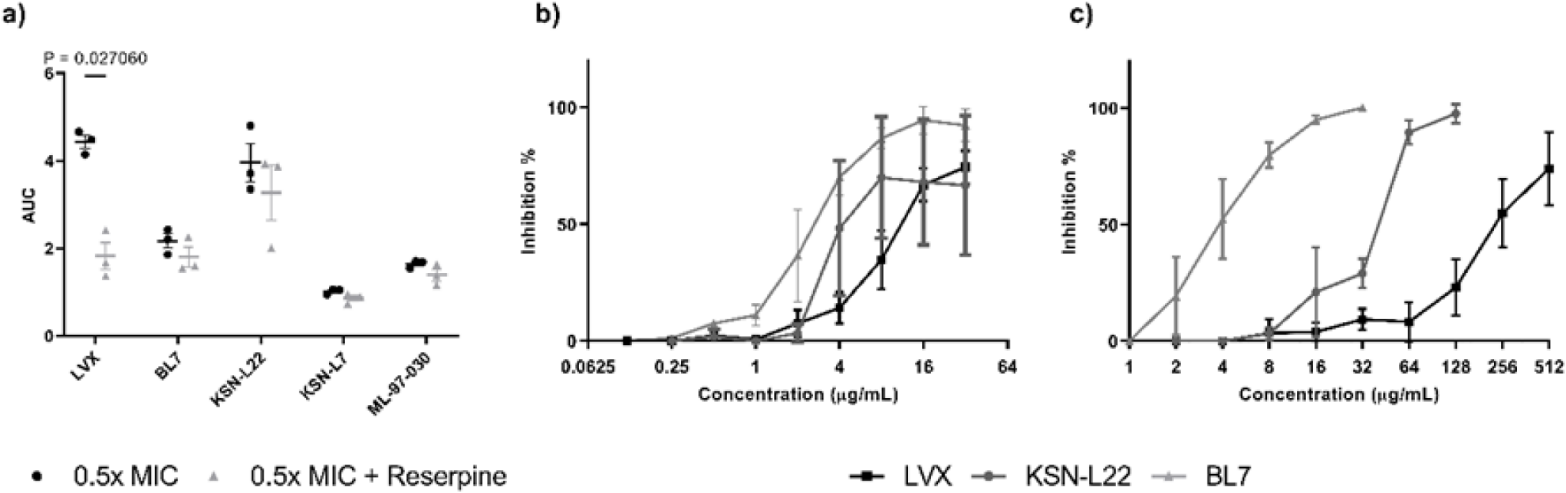
**a**), Reserpine assay data for **levofloxacin** (**7**, **LVX**), **ML-97-030 (42)**, **KSN-L7 (43)**, **KSN-L22 (46)**, **BL-7 (50)** in NorA overexpressing strain NCTC 13616. Each point represents a biological replicate, with the bar displaying the mean AUC and SEM displayed by the error bars. Multiple unpaired *t* tests with Welch correction were used to compare AUC in the presence of 0.5x MIC to the presence of 0.5x MIC with reserpine but a significant difference between the means was found only for **LVX** (*p* = 0.027060). While **LVX** is demonstrated to be a substrate of NorA, these data suggest that none of the ERB-modified antibiotics are substrates of NorA. **b–c**), Gyrase inhibition of **b**) WT GyrA and **c**) S84L mutant GyrA from *S. aureus* demonstrated comparable inhibition of the WT enzyme by **46**, **50**, and **LVX**, while both **46**, and to a greater extent **50**, demonstrated significantly better inhibitory activity against the S84L mutant enzyme than **LVX**.

### Determination of Efflux Susceptibility of Second-Generation ERB-Fluoroquinolones

The efflux susceptibilities of representative examples of second-generation ERB-fluoroquinolones **ML-97–030 (42)**, **KSN-L22 (46)**, and **BL-7 (50)** were determined using the reserpine inhibition growth assay in NCTC 13616 (Figure 3a, Figure S8) and by testing the compounds in the isogenic *S. aureus* strains SA-1199 and SA-1199B (Table 2).

None of the ERB-fluoroquinolones demonstrated a significant increase in efficacy in the presence of reserpine; **43**, a racemic mixture of two diastereomers, showed a clear reduction in efflux liability compared to **levofloxacin**, as evidenced by the smaller shift in growth curves in the presence of reserpine (Figure S8A). The (S,R)-diastereomer **46**, as well as analogues **50** and **ML-110-014 (51)**, showed minimal shift, suggesting that they are largely resistant to NorA-mediated efflux (Figure 3A, Figure S8A and S8B). Consistent with these findings, MIC determinations showed only a 2-fold difference between SA-1199 and SA-1199B for **46**, while **50** showed no difference at all, confirming their efflux resistance (Table 2). In contrast, **levofloxacin** displayed an 8-fold increase in MIC in the NorA-overexpressing strain relative to the WT and was significantly more efficacious in the presence of reserpine (*p* = 0.021648).

Together, these results demonstrate excellent alignment between the reserpine growth assay and MIC data and confirm that ERB modification markedly decreases efflux liability across multiple scaffolds, thereby restoring antibacterial activity against fluoroquinolone-resistant *S. aureus*.

### Type II Topoisomerase Inhibitory Activity of Second-Generation ERB-Fluoroquinolones

Evaluation of the inhibitory activities of **levofloxacin (7)**, **KSN-L22 (46)**, and **BL-7 (50)** against *S. aureus* DNA gyrase in a cell-free environment (Figure 3B–C) revealed a range of IC_50_ values. The data provide further evidence that ERB-modified fluoroquinolones can maintain on-target activity and possess the same mechanism of action as the parent fluoroquinolones. The activities of all compounds against the wild-type enzyme were broadly comparable (**50** IC_50_ = 2.52 ± 0.21 μg/mL, **46** IC_50_ = 3.37 ± 0.67 μg/mL, **levofloxacin** IC_50_ = 8.71 ± 0.95 μg/mL). However, against the mutant S84L enzyme, **50** (S84L IC_50_ = 3.80 ± 0.42 μg/mL) was approximately seven to 10-fold more active than **46** (S84L IC_50_ = 40.53 ± 3.86 μg/mL), which was itself approximately five to 7-fold more active against the same enzyme than **levofloxacin** (S84L IC_50_ = 196.9 ± 36.59 μg/mL). This suggests introduction of the ERB fragment provides additional contact with the mutant enzyme, leading to improved ability of these compounds to inhibit DNA gyrase in the presence of mutations. This is an additional benefit of the ERB approach, as the interactions with the bacterial target, both mutant and WT, can be considered during the design of the ERB fragment and the ERB-modified compound can show improved activity against the mutant target.

### Accumulation of ERB-Fluoroquinolones in Bacteria

An accumulation assay was performed to further validate the ERB technology. We have hypothesized that ERB-modified antibiotics, such as **KSN-L22 (46)**, are less susceptible to efflux and, therefore, accumulate more than the parent compound within bacterial cells. This could be the mechanism behind their ability to kill bacteria even in the presence of multiple target mutations (Table 2). The results showed that the level of accumulation of **levofloxacin** was lower in the NorA-overexpressing SA-1199B strain compared to the WT SA-1199 strain, and this difference was statistically significant (*p* = 0.000026) (Figure 4). The data aligned with the MIC observed for **levofloxacin** in these two strains, where an 8-fold increase in MIC was noted in the SA-1199B strain compared to SA-1199. On the other hand, no significant change in accumulation was observed for **46** in the SA-1199B strain compared with the SA-1199 strain. Again, these findings were consistent with the MIC data for **46**, as no significant changes in MIC were observed in the efflux mutant SA-1199B strain compared to those of the WT SA-1199 strain.

**Figure 4:**
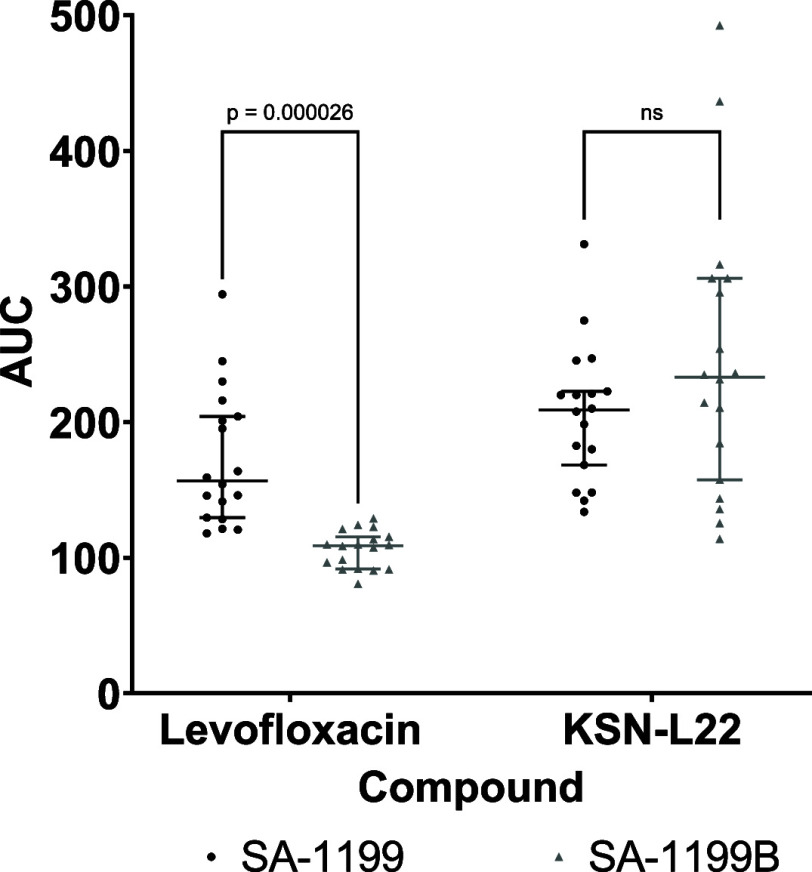
Accumulation assay comparing intracellular antibiotic retention in *S. aureus* SA-1199 WT and SA-1199B (containing upregulated NorA efflux pump) strains. Multiple unpaired *t* tests with Welch correction were applied to determine the significance of the data.

### Molecular Dynamics Simulation to Study the Interaction of **KSN-L22** **(46)** and Levofloxacin with NorA

Molecular dynamics (MD) simulations, along with Mdpocket analysis, confirmed the existence of two adjacent binding pockets within the NorA efflux pump (Figure 5a, Table S10). The volume of the inhibitor binding site is 1066 (±182.9) Å^3^, while the substrate binding site is slightly smaller with a volume of 903 (±196.2) Å^3^. To evaluate ligand binding, the distances between the center of mass (COM) of each ligand and both binding pockets were measured (Figure 5b,c).

**Figure 5:**
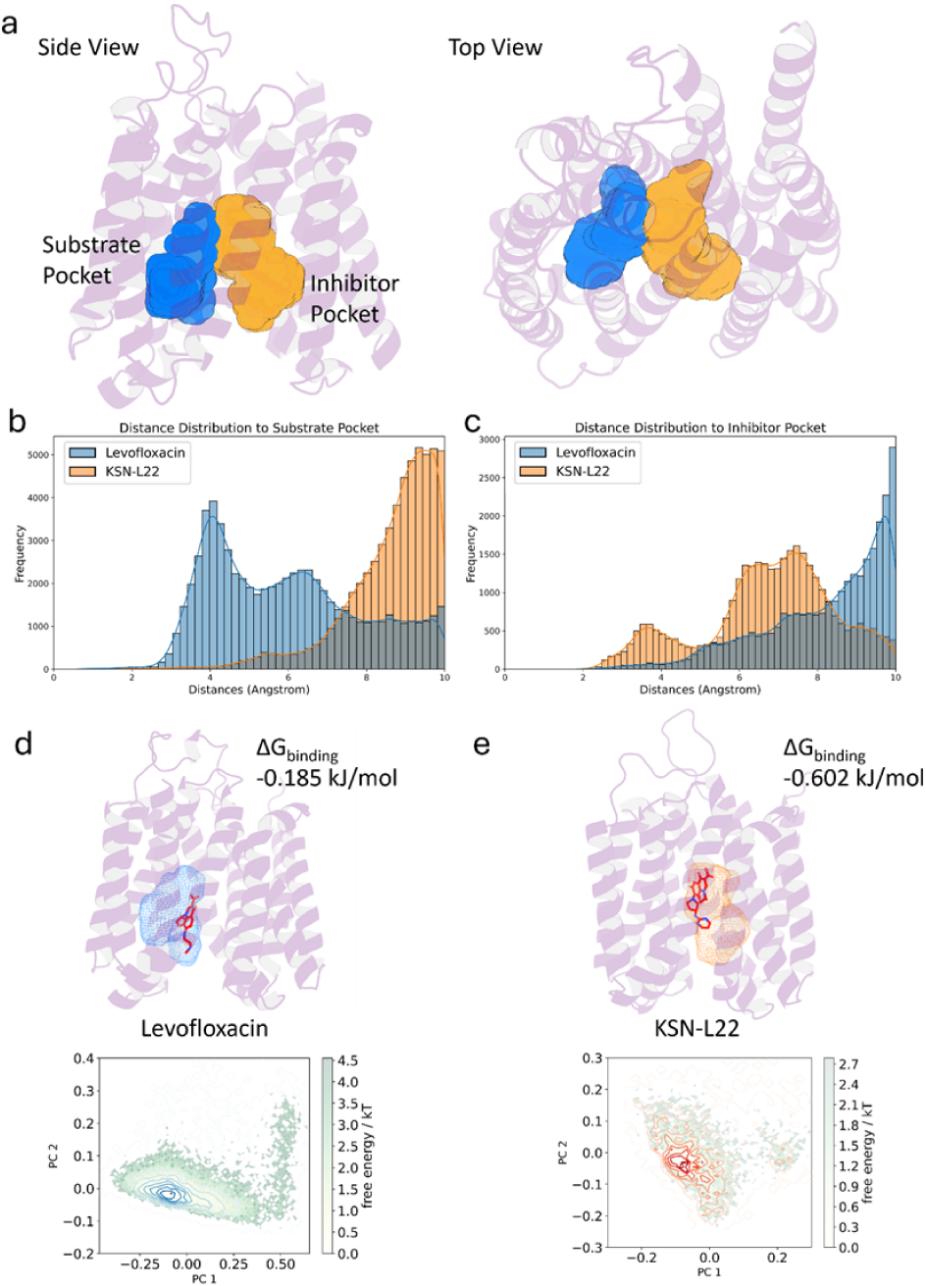
**a**) Substrate binding hydrophilic pocket (blue) and inhibitor binding hydrophobic pocket (yellow) in NorA (PDB ID: 7LO8) detected by Mdpocket. **b**) Distances between COM of the ligand to the COM of the substrate pocket. **c**) Distances between COM of the ligand to the inhibitor pocket. **d–e**) PCA projection of selected frames, and the position of each ligand inside the protein in the selected stable structure. The binding energy of each system is annotated on the top right of each figure.

The results reveal that **46** has a significantly reduced likelihood of interacting with the substrate pocket compared to that of its parent **levofloxacin**. In contrast, it exhibits a marginally stronger preference for the inhibitor binding pocket. Furthermore, the calculated binding energies suggest that **46** exhibits an approximately 3-fold stronger binding affinity to the inhibitor pocket (−0.602 kJ/mol) (Figure 5e) than **levofloxacin** to the substrate pocket (−0.185 kJ/mol) (Figure 5d), reinforcing its inhibitory potential. This provides further evidence that targeting the hydrophobic binding pocket within the efflux pump makes ERB antibiotics less susceptible to efflux.

### Activity of Second-Generation ERB-Fluoroquinolones against a Large Panel of Gram-Positive Bacteria

Lead compounds **KSN-L22** (**46**) and **BL-7** (**50**) were evaluated against a diverse panel of *Streptococcus* clinical isolates (Table S11A). **50** demonstrated consistently potent activity across *Streptococcus pyogenes* (MIC range ≤ 0.03–0.125 μg/mL, modal MIC ≤ 0.03 μg/mL; *n* = 12 isolates), *S. agalactiae* (MIC range ≤ 0.03–2 μg/mL, modal MIC 0.06 μg/mL; *n* = 10), and *S. dysgalactiae* (MIC range ≤ 0.03–0.125 μg/mL, modal MIC ≤ 0.03 μg/mL; *n* = 8). **46** also showed notable activity across the same panel, with MICs of 0.06–0.125 μg/mL in *S. pyogenes*, 0.06–0.5 μg/mL in *S. agalactiae*, and 0.0625–0.125 μg/mL in *S. dysgalactiae*, supporting its potential as a promising agent against β-hemolytic streptococci. Next, **50** was assessed against an extended panel of Gram-positive clinical isolates, including vancomycin-susceptible and -resistant *Enterococcus* spp., *Streptococcus* spp., and methicillin-susceptible and -resistant *Staphylococcus aureus* strains (Table S11B). **50** demonstrated potent activity against *E. faecalis* (MIC 0.125–0.5 μg/mL) and *E. faecium*, including VanA, VanB, and VanD phenotypes (MIC 0.125–1 μg/mL), as well as *E. gallinarum* (0.25 μg/mL). Consistent inhibition was observed across multiple *S. pneumoniae* isolates, including MDR and PRSP strains (MIC 0.06–0.125 μg/mL), and activity was maintained against *S. oralis*, *S. mutans*, *S. salivarius*, *S. sanguinis*, and *S. pyogenes* (MIC 0.03–0.25 μg/mL). **50** also exhibited broad activity against *S. aureus*, inhibiting the majority of MSSA and MRSA isolates at ≤0.03125 μg/mL, with modest MIC elevations observed in VISA, VRSA, linezolid-nonsusceptible, daptomycin-nonsusceptible and tigecycline-nonsusceptible strains. *Staphylococcus epidermidis*, *S. hemolyticus* and *S. saprophyticus* isolates were also susceptible (MIC 0.03125–0.06 μg/mL), demonstrating an excellent activity profile across key Gram-positive pathogens, including clinically relevant drug-resistant phenotypes. Overall, the ERB-modified fluoroquinolones demonstrated markedly enhanced antibacterial potency against fluoroquinolone-resistant clinical isolates, including strains with known efflux upregulation and multiple target-site mutations, whereas comparator fluoroquinolones were either inactive or required substantially higher concentrations for inhibition. These findings confirm that incorporation of the ERB motif enables sustained potency against efflux-mediated fluoroquinolone resistance mechanisms, expanding the therapeutic coverage of this class against clinically relevant resistant Gram-positive bacteria.

### Activity of Second-Generation ERB-Fluoroquinolones in Gram-Negative Bacteria

Although the ERB strategy was initially developed using **ciprofloxacin** and validated primarily against Gram-positive bacteria, the poor Gram-negative activity of **ML-77-005 (8)** highlighted the importance of outer membrane permeability and physicochemical balance in extending this approach to Gram-negative pathogens.^43^ This limitation, together with the restoration of activity in the presence of PMBN, informed the subsequent optimization of the fluoroquinolone core and ERB fragment to improve polarity and permeation. Sequence analysis revealed high levels of amino acid conservation across major efflux systems, including MFS transporters such as SmvA in *Klebsiella pneumoniae* and *P. aeruginosa* as well as RND family efflux pumps in Gram-negative bacteria. Notably, residues implicated in ERB-fluoroquinolone binding to NorA, including the conserved phenylalanine ring, were preserved across all of these systems. This conservation supports the broader applicability of the ERB strategy across both Gram-positive and Gram-negative efflux pumps and suggests the potential for developing pan-efflux-resistant antibiotics.

We evaluated second-generation ERB-fluoroquinolones **KSN-L22 (46)**, **BL-7 (50)**, and **ML-110-014 (51)** in several clinically important Gram-negative species with known upregulation of RND and MFS efflux pumps. We initially focused on *A. baumannii*, a pathogen that utilizes a number of complementary efflux systems as part of its antibiotic resistance mechanisms^44^ and for which carbapenem resistance is recognized as an urgent/critical threat by both the WHO^45^ and CDC.^46^ Across a diverse panel of 25 *A. baumannii* strains, including OXA-23- and OXA-24-producing clinical isolates, all three ERB fluoroquinolones demonstrated consistent and potent activity (Table 3).

**Table 3:** Antibacterial Activity (MIC, μg/mL) of Second-Generation ERB-Fluoroquinolones against *Acinetobacter baumannii* Strains

**MIC, μg/mL**							
Species	Strain	Resistance	**KSN-L**22 (46)	**BL-**7 (50)	ML-110-014 **(51)**	**Ciprofloxacin**	**Levofloxacin**
*Acinetobacter baumannii*	UKA1	oxa23	4	4	2	8	2
	UKA2	Low resistance	8	8	4	64	4
	UKA9		≤0.06	0.125	0.06	0.25	0.125
	UKA12	oxa23	2	4	2	>128	8
	UKA13	oxa23	2	4	2	>128	16
	UKA15	oxa23	1	4	2	16	4
	UKA19		0.25	0.5	0.25	8	1
	BAA-1709		0.125	0.125	0.06	0.25	0.125
	BAA-1710 (AYE)		4	8	4	64	4
	BAA-2093		≤0.03–0.06	-	≤0.03–0.06	≤0.125	0.06
	NCTC 10303		≤0.06	0.125	0.06	0.5	0.125
	NCTC 12156		0.125	-	0.125	1	1
	NCTC 13302	oxa24	4	4	4	32	4
	NCTC 13424	oxa23	>64	>64	>32	>128	64
	ATCC 17978		≤0.25	0.25	≤0.125	0.25	0.125
	T strain		8	8	8	64	8
	A118		≤0.06	≤0.06	0.06	0.25	≤0.06
	A318		8	16	4	32	8
	A319		8	4	4	32	4
	A600 colR		4	4	4	32	4
	A601 colS		≤0.06	0.125	≤0.03	8	2
	86	oxa23	8	8	16	128	8
	96	oxa23	4	4	4	128	8
	113	oxa23	4	4	4	64	8
	W1		4	8	4	128	8

**KSN-L22 (46)**, **BL-7 (50)**, and **ML-110-014 (51)** showed panel-wide activity comparable to **levofloxacin** (MIC_50_ = 4 μg/mL) and markedly superior to **ciprofloxacin** (MIC_50_ = 32 μg/mL), which was frequently inactive. Notably, **51** and **46** retained low-micromolar activity against multiple highly resistant OXA-23-positive strains for which **ciprofloxacin** MICs exceeded 128 μg/mL. Time-kill assays for **46** and **51** showed rapid bactericidal activity in *A. baumannii* strain AYE (Figure S9), establishing both as promising candidates for further development against this pathogen.

Lacking an established efflux assay for *A. baumannii* comparable to the reserpine assay used in *S. aureus*, a checkerboard assay comparing the MICs of efflux substrate antibiotics with and without sub-MIC concentrations of **50** was devised; **50** was inferred to be an inhibitor of both AdeB (potentiated known AdeB substrates **ciprofloxacin**, **levofloxacin**, and **tobramycin**) and AdeJ (potentiated known AdeJ substrate **ceftazidime**) but not the AdeG efflux pump (Table 4). Additionally, strains of the *A. baumannii* AB5075 Mutant Library^47^ with transposons inserted into efflux pump components and regulator genes were tested against the ERB-fluoroquinolones (Table S12). While the fluoroquinolone parent compounds were demonstrated to have MICs 4-fold or lower in mutants lacking an active AdeB protein, the efficacy of **46** was less affected by the loss of AdeB, suggesting a lower efflux susceptibility through this efflux pump.

**Table 4:** *A. baumannii* Efflux Chequerboard Assay^a^

			**MIC (μg/mL)**		
Strain	Efflux Pump	Efflux Substrates	MIC alone	+**50** (0.25x MIC)	+**50** (0.5x MIC)
*A. baumannii* ATCC 17978	AdeABC	Ciprofloxacin **(1)**	0.25	0.0017–0.188	0.007–0.055
		Levofloxacin **(7)**	0.125	0.007–0.06	0.009–0.06
		Gentamicin	4	2.13–4.25	0.03–1.75
		Tobramycin	4	1.88–3.76	0.014–0.55
	AdeFGH	Chloramphenicol	64	60	56
	AdeIJK	Ceftazidime	4–16	0.94–3.76	0.003–0.438
	N/a	Colistin	0.25	0.015–0.123	0.0017–0.015
*A. baumannii* AYE	AdeABC	Ciprofloxacin **(1)**	64	3.5–15.5	0.188–0.9
		Levofloxacin **(7)**	4	0.014–1.88	0.012–0.9
		Gentamicin	>1,024	>1,024	60.2–>1,024
		Tobramycin	256	56–248	0.375–136
	AdeFGH	Chloramphenicol	128	120	224
	AdeIJK	Ceftazidime	>1,024	>1,024	3.76–224
	N/a	Colistin	0.25–0.5	0.015	0.003–0.007

a While **BL-7 (50)** failed to potentiate chloramphenicol at sub-MIC concentrations, it successfully potentiated the action of ceftazidime and various quinolones and aminoglycosides.

Across nine *Escherichia coli* strains, the ERB-fluoroquinolones showed activity comparable to established fluoroquinolones in the majority of isolates, with MICs between 0.06 and 0.125 μg/mL observed in most strains tested (Table S13A). These results indicate that ERB modification can preserve clinically relevant activity in *E. coli* despite the introduction of hydrophobic fragments. In contrast, reduced activity was observed in *K. pneumoniae*, where the ERB-fluoroquinolones were consistently less potent than **ciprofloxacin** and **levofloxacin** across the three strains tested (Table S13A). A similar trend was evident in *P. aeruginosa*, which has well-established additional permeability barriers (Table S13B).

To determine whether ERB modification reduced on-target activity against DNA gyrase, an *in vitro* *E. coli* DNA gyrase assay was performed. **7** and **46** showed comparable inhibitory activity against wild-type *E. coli* DNA gyrase, with IC_50_ values of 0.19 ± 0.03 μg/mL and 0.29 ± 0.02 μg/mL, respectively (Figure 6).

**Figure 6:**
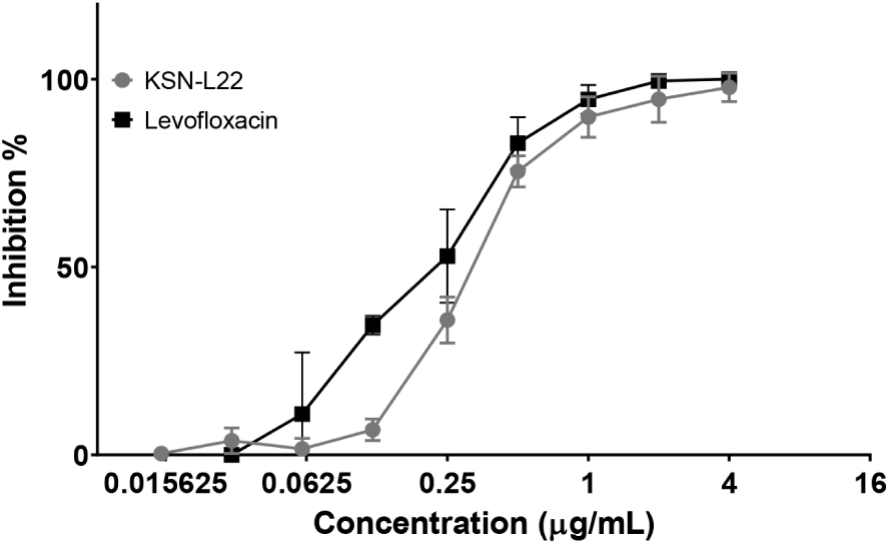
*In vitro* DNA gyrase inhibition assay using WT *E. coli* DNA gyrase demonstrates similar inhibition of the target by **levofloxacin** (**7)** and **KSN-L22** (**46)**.

In contrast, **46** showed greater potentiation than **levofloxacin** in *P. aeruginosa* when tested in the presence of the outer-membrane permeabiliser polymyxin B nonapeptide (PMBN) (MIC_50_ = 0.06 μg/mL vs 0.125 μg/mL, *n* = 7 strains; Table S13B). These results indicate that second-generation ERB-fluoroquinolones retain on-target activity but still suffer from limited penetration across the Gram-negative outer membrane, likely due to the hydrophobic ERB unit.

**46** was also evaluated against *Neisseria gonorrheae*, a CDC-designated urgent threat pathogen.^46^ In a panel of 75 clinical isolates, including extensively fluoroquinolone-resistant strains (CIP-R and multidrug-resistant backgrounds), **46** showed an MIC_90_ of 2 μg/mL, with several isolates inhibited at concentrations of ≤0.03–0.5 μg/mL (Table S13C). These results demonstrate that, when sufficient outer membrane permeation is achieved, ERB-fluoroquinolones retain activity against fluoroquinolone-resistant *N. gonorrheae*, supporting the potential of ERB modification to overcome efflux-mediated resistance in clinically relevant Gram-negative pathogens.

### Resistance to Second-Generation ERB-Fluoroquinolones

To investigate possible resistance mutations against the ERB fluoroquinolones, serial passaging experiments were conducted in several representative strains of *S. aureus* (Table S14). Whole genome sequencing of the resulting mutants again revealed no efflux-related mutations in the *S. aureus* strains used; instead, previously reported target mutations (GyrA E88K, S84L, ParC E84K, I487L) as well as several off-target mutations were identified.

The mutation frequencies of Gram-positive and Gram-negative bacteria at 2x, 4x, and 8x the agar MICs of **KSN-L22 (46)** and **BL-7 (50)** were determined to assess the likelihood of resistance emergence (Table S15). Mutation frequency studies showed that both **46** and **50** have lower mutation frequencies than **levofloxacin**, particularly in *S. aureus* NCTC 13616, where reductions were almost one log and statistically significant (*p* = 0.0006 and 0.0005, respectively). **50** consistently displayed the lowest frequencies, with no detectable mutants at ≥4× MIC in most strains. Similar trends were observed in *E. faecium*, *A. baumannii*, and *E. coli*, suggesting reduced potential for resistance emergence across Gram-positive and Gram-negative species.

### Time Kill Kinetics of Second-Generation ERB-Fluoroquinolones

Time-kill assays comparing **levofloxacin** with the ERB-fluoroquinolone compounds **KSN-L22 (46)**, **BL-7 (50)**, and **ML-110-014 (51)** demonstrated clear differences in bactericidal performance and resistance emergence (Figure S9). Against *S. aureus* NCTC 13616 and *E. faecium* NCTC 12204, the ERB-fluoroquinolones produced more rapid and sustained reductions in viable counts than **levofloxacin**, consistent with enhanced intracellular retention and bactericidal activity. In *E. coli* NCTC 12923, similarly sustained killing was observed, with no rapid regrowth detected. Rapid bactericidal activity was also observed against *A. baumannii* strain AYE (Figure S9). In contrast, in *K. pneumoniae* NCTC 13368 and *P. aeruginosa* PAO1, resistant subpopulations emerged in all three experimental repeats for both levofloxacin and the ERB fluoroquinolones. However, whole genome sequencing did not identify single-nucleotide polymorphisms, suggesting that resistance is likely driven by nongenetic or regulatory mechanisms rather than stable target-based mutations.

### *In Vitro* DMPK, Protein Binding and Toxicity Assessment for **KSN-L22 (46)** and **BL-7 (50)**

Given the structural changes introduced by ERB incorporation, we next evaluated the impact of ERB modification on solubility, protein binding, toxicity, pharmacokinetic behavior, and overall drug-like properties. These studies were undertaken to determine whether the design of the second-generation ERB fragments overcame the solubility and protein-binding liabilities associated with the first-generation ERB fragment, and whether integration of the ERB motif into the fluoroquinolone scaffold affected off-target interactions, safety liabilities, or physicochemical properties relevant to in vivo performance. Aqueous solubility studies showed a marked improvement in the physicochemical profile of the second-generation compounds relative to the first-generation lead. The second-generation lead compounds, **46** and **50**, showed substantially improved aqueous solubility (183 μM and 155.7 μM, respectively) compared with the first-generation ERB-fluoroquinolone lead compound **8**, which showed very poor solubility (<5 μM) at pH 7.4. Plasma protein binding was also markedly reduced, with values of 58% for 46 and 72% for 50, compared with >99% for **8**. Plasma protein binding studies showed that **46** exhibited 58% protein binding, whereas **50** showed a higher level of protein binding at 72%, indicating that ERB-modified analogues may vary in their extent of plasma protein binding depending on the nature of the ERB modification. To assess the effect of this increase in plasma protein binding following ERB modification, which is consistent with the increased hydrophobicity introduced by the ERB moiety, the antibacterial activity of **46** and **50** was evaluated against the same panel of *S. aureus* strains used in Table 1 in the presence of 4% albumin (Table S16). For **46**, the MICs were essentially unchanged in the presence of albumin, indicating that its slightly increased protein binding did not measurably affect antibacterial activity under these conditions. In contrast, **50**, which showed higher plasma protein binding and contains a larger, more hydrophobic ERB fragment, exhibited a 2- to 8-fold increase in MIC in the presence of 4% albumin across the strain panel. Despite this reduction in potency, **50** remained substantially more active than **levofloxacin**, with MIC values still up to 8-fold lower than those of the parent drug.

An off-target toxicity screen was performed using SafetyScreen44 for both **46** and **50** with other key biological targets including the hERG channel (Table S17 and Table S18, respectively). At 10 μM, **46** displayed a clean off-target profile, with only one significant interaction (>50% inhibition) observed for the A_2_A receptor (53.6%). Weak to moderate effects (25–50% inhibition) were seen for COX1 (30.3%), kappa opioid receptor (27.4%), and acetylcholinesterase (33.0%), with all other targets showing <25% inhibition and thus not considered significant. **50** exhibited off-target activity with 3–4 receptors, with significant inhibition of the A_2_A (82.8%) and M_2_ (62.8%) receptors. Weak to moderate inhibition (25–50%) was observed for 5-HT_2_A (41.5%), acetylcholinesterase (34.2%), PDE4D2 (31.8%), COX2 (25.9%), and M_1_ (25.5%). All remaining targets showed <25% inhibition.

**46** and **50** were evaluated for their potential to inhibit eight major human cytochrome P450 isoforms using HPLC-UV/vis and HPLC-MS/MS detection (Table S19). **46** exhibited minimal inhibition across most isoforms, with the highest effect (25.2% inhibition) observed against CYP3A (midazolam substrate) at 10 μM indicating only weak inhibition. All other isoforms showed ≤13% inhibition, suggesting low risk for clinically relevant drug–drug interactions. **50** displayed a similar low-inhibition profile, with ≤17% inhibition across CYP1A, CYP2C9, and CYP2C8, and only 10.5% inhibition of CYP3A (midazolam substrate). CYP2B6, CYP2C19, and CYP2D6 inhibition remained <10%. These findings demonstrate that both **46** and **50** have low potential to cause significant CYP450-mediated metabolic interactions, supporting their suitability for further preclinical development.

Next, the compounds were evaluated for their toxicity in the nontumor eukaryotic cell line WI38 and in ICR-2 male mice. Both **46** and **50** were found to be nontoxic at 100 μM, the highest concentration tested. The maximum tolerated dose (MTD) of **46** and **50** was evaluated in male ICR mice following oral administration at escalating doses (100, 200, and 400 mg/kg) (Table S20 and Table S21). Clinical observations, body weight, and mortality were monitored for 14 days postdose. Both compounds were well tolerated at all three doses with no mortality, no significant clinical signs of toxicity, and stable body weights. Body weight changes across all groups were within normal physiological variation (<10% loss). Based on these findings, the MTD for both **46** and **50** was determined to be ≥400 mg/kg, supporting their use in subsequent *in vivo* efficacy studies. Taken together, the SafetyScreen44 profiling, CYP450 interaction studies, and MTD data provide reassurance that ERB modification does not introduce significant off-target liabilities or drug–drug interaction risks. Both **46** and **50** exhibited clean safety profiles, minimal CYP450 inhibition, and were well tolerated up to ≥400 mg/kg in mice.

### *In Vivo* Pharmacokinetic Studies in Mice

Both oral and intravenous pharmacokinetic (PK) studies were performed for **KSN-L22 (46)** and **BL-7 (50)** to determine key PK parameters and assess their suitability for *in vivo* efficacy studies in mice (Figure S10 and Figure S11). Following intravenous administration at 5 mg/kg, both compounds achieved substantially higher systemic exposure than **levofloxacin**, as reflected by their greater AUC_0_-∞ values (15,728 ng·h/mL for **46** and 33,079 ng·h/mL for **50** vs 9,169 ng·h/mL for **levofloxacin**). **46** exhibited moderate clearance (12.5 mL/min/kg) and a steady-state volume of distribution of 1.4 L/kg, consistent with balanced plasma and tissue distribution, whereas **50** showed low clearance (2.52 mL/min/kg) and low Vss (0.35 L/kg), indicating plasma-restricted disposition. After oral dosing at 20 mg/kg, **46** demonstrated improved bioavailability (49.3%) relative to **levofloxacin** (40.7%), with a higher AUC_0_-∞ and slightly prolonged half-life (5.1 h vs 5.7 h) (Figure S10). **50** showed rapid absorption (Tmax = 0.5 h), high *C*_max_ (14,542 ng/mL), and excellent oral bioavailability (82.97%), resulting in markedly enhanced systemic exposure compared with **levofloxacin** (Figure S11).

### *In Vivo* Efficacy Studies in Mice

The main aim of the *in vivo* studies was to establish proof of concept that the ERB-modified fluoroquinolones retain antibacterial activity *in vivo* and to compare them directly with **levofloxacin** under the same dosing conditions. MRSA strains were selected because these compounds showed the most consistent improvement against *S. aureus* *in vitro*. MRSA ATCC 29213 and MRSA ATCC 33591 are well-established *S. aureus* strains for infection models and provided reliable systems for *in vivo* evaluation.

The efficacy of **KSN-L22 (46)** was assessed in a murine thigh infection model using MRSA ATCC 29213 (Figure 7a). Treatment was initiated 2 h postinfection, and animals received **46** orally at 50 mg/kg at 2, 8, and 14 h postinfection. **Levofloxacin** was used as a reference under the same regimen. At 26 h, the vehicle group showed a bacterial burden of 8.36 log_10_CFU/g. **46** treatment reduced bacterial counts to 3.19 log_10_ CFU/g, corresponding to a 5.17 log_10_ CFU/g reduction relative to vehicle (*p* < 0.0001, Kruskal–Wallis). **Levofloxacin** treatment resulted in 3.28 log_10_ CFU/g, a 5.08 log_10_ CFU/g reduction relative to vehicle (*p* < 0.0001).

**Figure 7:**
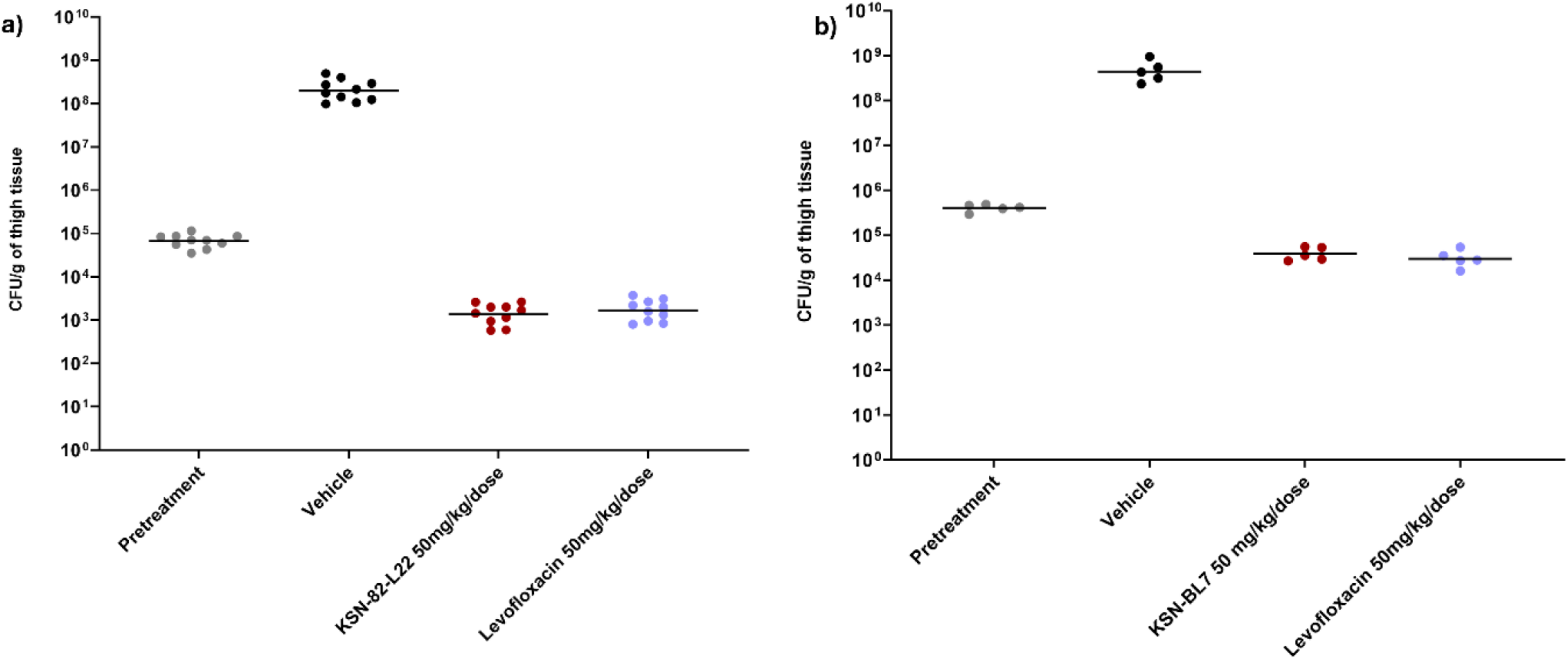
**a**) *In vivo* efficacy study of **46** in MRSA ATCC 29213 and **b**) *in vivo* efficacy study of **50** in MRSA ATCC 33591 thigh infection models, showing statistically significant reductions in bacterial burdens.

**BL-7 (50)** was evaluated in a neutropenic murine thigh infection model by using MRSA ATCC 33591 (Figure 7b). Mice were immunosuppressed with cyclophosphamide and treated orally with **50** or **levofloxacin** at 50 mg/kg TID at 2, 8, and 14 h postinfection. The vehicle group reached 8.7 log_10_ CFU/g at 26 h. **50** reduced bacterial counts to 4.6 log_10_ CFU/g, a 4.1 log_10_ CFU/g reduction relative to the vehicle (*p* = 0.0006), whereas **levofloxacin** reduced counts to 4.51 log_10_ CFU/g, corresponding to a 4.19 log_10_ CFU/g reduction relative to the vehicle (*p* = 0.0001).

## Discussion and Conclusions

Efflux pumps play a central role in bacterial resistance by actively expelling antibiotics and reducing intracellular drug concentrations. By blocking these pumps, either directly or through substrate inhibition, ERB technology increases intracellular antibiotic accumulation and enhances potency against resistant strains. This dual-action mechanism has the potential to mitigate resistance by sustaining intracellular drug levels and limiting the selective pressure for resistance mutations. A key strength of ERB technology is its ability to revitalize existing antibiotics. Fluoroquinolones, a class particularly susceptible to efflux, were used as a model system to demonstrate this approach. Proof of concept was established with **ML-77-005 (8)**, an ERB-modified **ciprofloxacin** derivative, which showed markedly improved activity relative to **ciprofloxacin** alone and to **ciprofloxacin-NMP** combinations. This enhanced potency was attributed to reduced efflux susceptibility while retaining DNA gyrase inhibition. In *S. aureus* strains with high NorA expression, ERB-fluoroquinolones, including **8**, **KSN-L22 (46**) and **BL-7 (50)** exhibited increased intracellular retention and improved antibacterial activity. Subsequent optimization yielded second-generation ERB-fluoroquinolones such as **46** and **50**, which showed enhanced interactions within inhibitor-binding pockets and robust activity against fluoroquinolone-resistant Gram-positive pathogens, including β-hemolytic *Streptococcus* spp., vancomycin-resistant *Enterococcus* phenotypes, and methicillin- and glycopeptide-resistant *Staphylococcus aureus*. Molecular dynamics simulations supported preferential binding of ERB-modified antibiotics to inhibitor pockets compared with binding of parent compounds to substrate pockets, with an approximately 3-fold increase in binding affinity. This mechanistic advantage translated into substantial reductions in MICs, typically 20- to 128-fold lower than unmodified antibiotics across clinically relevant Gram-positive pathogens.

Cellular accumulation studies provided functional validation, demonstrating significantly higher intracellular levels of **46** in efflux-overexpressing strains relative to those of **levofloxacin**. Increased accumulation was correlated with reduced efflux susceptibility and superior antibacterial activity. Importantly, biochemical assays confirmed that ERB-fluoroquinolones retained on-target type II topoisomerase inhibition, with compounds such as **46** showing enhanced activity against mutant enzymes including the S84L variant.

Preclinical profiling of **46** and **50** demonstrated a favorable balance of antibacterial potency, pharmacokinetics, and safety. Plasma protein binding for both compounds was higher than that reported for **levofloxacin** (38%), but remained lower than that of several other fluoroquinolones, including the more recent analogue delafloxacin (84%). Compound **46** showed 58% protein binding, whereas **50** showed higher protein binding at 72%, consistent with its larger and more hydrophobic ERB moiety. These findings indicate that the increased hydrophobicity associated with ERB modification can modestly increase protein binding and reduce activity in the presence of albumin in a compound-dependent manner, but does not abolish the potency advantage of the ERB-modified analogues. SafetyScreen44 and CYP450 interaction studies indicated minimal off-target liabilities and low risk of drug–drug interactions, while maximum tolerated dose studies showed both compounds were well tolerated at doses ≥400 mg/kg without mortality or significant clinical findings. These data indicate that ERB modification does not compromise the intrinsic safety profile of fluoroquinolones. Both compounds also achieved higher systemic exposure than **levofloxacin** following intravenous and oral administration, with **46** showing improved oral bioavailability and **50** exhibiting rapid absorption and high Cmax.

Consistent with their pharmacokinetic profiles, **46** and **50** demonstrated a strong *in vivo* efficacy in murine infection models. Both compounds produced dose-dependent reductions in bacterial burden in MRSA thigh infection models, with **46** achieving >4 log_10_ CFU/g reductions in an immunocompetent model and **50** showing comparable efficacy in a neutropenic model. These results demonstrate robust antibacterial activity even in immunocompromised settings and support the advancement of ERB-fluoroquinolones as promising candidates for the treatment of multidrug-resistant Gram-positive infections.

Although this study focused primarily on Gram-positive pathogens, preliminary investigations in Gram-negative bacteria highlight the broader potential of the ERB strategy and inform its next stages of optimization and development. Clinically relevant activity was observed against *A. baumannii*, *E. coli*, and *N. gonorrheae*, indicating that ERB modification can preserve antibacterial activity in Gram-negative organisms when sufficient outer membrane permeation is achieved. However, further optimization of permeability and physicochemical balance will be required to improve outer membrane penetration in *K. pneumoniae* and *P. aeruginosa* and thereby enable the development of broad-spectrum ERB antibiotics.

Current strategies to address AMR often rely on incremental modification of existing antibiotics, which frequently fail to overcome fundamental resistance mechanisms such as efflux. For example, newer generations of fluoroquinolones and tetracyclines can show improved target potency yet remain vulnerable to the same efflux pathways as earlier compounds,^48,49^ enabling resistance to emerge rapidly. Combination strategies employing efflux-pump inhibitors have also proven challenging due to toxicity, poor pharmacokinetics, and mismatched exposure profiles between EPIs and antibiotics. In contrast, ERB technology integrates efflux resistance directly into the antibiotic scaffold, avoiding the limitations of combination therapy and addressing the efflux at its source.

In summary, ERB technology represents a significant advance in antibiotic design by enabling the development of antibiotics that are intrinsically less susceptible to efflux. This study demonstrates that ERB modification enhances intracellular accumulation, reduces efflux susceptibility, and preserves antibacterial potency, as supported by complementary mechanistic, biochemical, and *in vivo* evidence. Future studies should assess whether sustained intracellular exposure conferred by ERB modification reduces the frequency of resistance emergence under prolonged selection and serial passage models. In parallel, refinement of ERB chemistry to balance hydrophobicity with Gram-negative permeation will be essential to broaden the spectrum and extend the applicability of this approach to additional antibiotic classes. Beyond revitalizing existing drugs, ERB technology provides a general framework for designing next-generation antibiotics with built-in resilience to efflux-mediated resistance at the earliest stages of discovery.

## Experimental Section

### General Materials and Methods – Chemistry

Synthetic building blocks (including **enoxacin (3)**), reagents, and solvents were purchased from Sigma-Aldrich (Merck KGaA, USA), Thermo Fisher Scientific (UK, including Acros Organics, Maybridge and Alfa Aesar), Fluorochem (UK), Insight Biotechnology (UK), Activate Scientific (UK), Enamine (Ukraine), VWR International (USA), Oxchem (USA), Apollo Scientific (UK), Combi-Blocks (USA), and Ark Pharm Inc. (USA). Silica gel, thin layer chromatography plates, and NMR tubes were purchased from Sigma-Aldrich, while SCX-2 solid phase extraction cartridges and LC-MS vials, were purchased from Biotage (Sweden) and Agilent (USA), respectively. Microwave reactions were performed in a Biotage Initiator+ Microwave Synthesizer in sealed glass vials, stirred at 600 rpm during heating, and were cooled to below 40 °C prior to removal from the reaction chamber.

Thin-layer chromatography (TLC) analysis was performed using silica gel plates (Merck silica gel 60 F254 plates) and visualized using ultraviolet (UV) light (254 nm wavelength) and/or staining with potassium permanganate solution. Manual flash column chromatography was performed using silica gel (Merck 9385, 230–400 mesh ASTM, 40–63 μM) as the stationary phase. Purification of tertiary amine-containing compounds was expedited through neutralizing the stationary phase with 3% triethylamine in the nonpolar mobile phase component prior to running the column. Automated flash column chromatography was performed using a Reveleris X2 Flash Chromatography System. Normal phase separations were carried out on Grace Reveleris Silica Flash cartridges and reverse phase separations on Biotage SNAP Ultra C18 cartridges. Preparative liquid chromatography–mass spectrometry (preparative LC-MS) was performed on an Agilent 1290 Infinity II Preparative LC/MSD System with Phenomenex Luna 5 μm C18(2) 100Å LC column (100 × 21.2 mm). Water (A) and acetonitrile (B) were used as mobile phases and formic acid (0.1%) was added to A to ensure acidic conditions throughout each purification run. Samples were wet loaded in dimethyl sulfoxide. Analytical liquid chromatography–mass spectrometry (LC-MS) was employed to monitor reaction progression and for compound identification. LC-MS analysis was performed either on a Waters Alliance 2695 HPLC coupled to a Waters Micromass ZQ instrument with a Waters 2996 PDA or an Agilent InfinityLab LC/MSD System consisting of an Agilent 1290 Infinity II Analytical-Scale LC Purification System coupled to a 6120 Quadrupole mass spectrometer. In both cases, HPLC was carried out using an Onyx Monolithic C18 column (50 × 4.6 mm) with water (A) and acetonitrile (B) as the mobile phases. Formic acid (0.1%) was added to both to ensure acidic conditions throughout the analysis. All compounds were >95% pure by LC-MS analysis. A detailed account of purification methods, flow rates, and instrument parameters for preparative and analytical LC-MS has been provided in the Supporting Information document.

Purification of compounds via recrystallization was achieved by dissolving the crude compound in a hot solvent of choice, then covering the container and leaving it to cool to room temperature until crystal formation was observed. Any insoluble contaminants or byproducts were removed by employing a hot filtration step prior to cooling. Slow crystallizations were further cooled to −20 °C from room temperature to expedite crystal formation. SCX-2 resin cartridges (1 g, 2 g, 10 g) were selected based on reaction scale; sorbent mass was selected to be ten times that of the calculated mass of the product. Cartridges were first activated by an initial wash with 2 column volumes of dichloromethane and 4 column volumes of methanol. The reaction mixtures were then poured onto the cartridge and the solvent allowed to pass through the cartridges under gravity. The cartridges were then washed with dichloromethane (three column volumes), dimethylformamide (three column volumes), and methanol (one column volume) and this cycle was repeated three times under vacuum to remove impurities. Products were eluted using 2 M ammonia solution in methanol (one column volume) and concentrated *in vacuo*.

Trituration was performed for purifying a particular compound from a mixture based on differing solubility profiles. A solvent was selected in which the desired product was poorly soluble and the unwanted byproducts were highly soluble. The crude material was suspended in the solvent, filtered and washed again, leaving the purified product in solid form and any impurities in solution. Lyophilization (freeze-drying, cryodesiccation) was carried out on a Frozen in Time Lablyo benchtop freeze drier connected to an Edwards RV5 rotary vane vacuum pump.

The specific optical rotation (OR; [α]) of chiral final compounds was determined at room temperature using a Bellingham-Stanley ADP 440+ polarimeter. The length of sample chamber used was 1 dm. High resolution mass spectra (HRMS) were obtained on a Thermo Navigator mass spectrometer coupled with liquid chromatography (LC) using electrospray ionization (ES) and time-of-flight (ToF) mass spectrometry. All NMR spectra were obtained at room temperature using a Bruker Ascend 400 MHz NMR spectrometer and interpreted using ACD/NMR Processor Academic Edition software or Mnova software (Mnova15, Mestrelab). Chemical shifts (δ H) are expressed in parts per million (ppm) relative to deuterated NMR solvents acetone, acetonitrile, chloroform, deuterium oxide, dimethyl sulfoxide, methanol, and trifluoroacetic acid.

### Building the saNorA Systems for Molecular Dynamics Simulations

The crystal structure of saNorA (protein data bank (PDB) ID: 7LO8) was obtained from the PDB. The missing loop (184–197) was modeled using AlphaFold3.^50^ The built model was then aligned to the docked structures and the ligands was extracted using pymol in order to get the docked pose. Three systems were prepared using the HTMD python package;^51^ saNorA-Apo, saNorA-Levo (with **levofloxacin** docked in the binding site) and saNorA-**46** (with **46** docked in the binding site). The protonation states were predicted based on pH 7 using PROPKA3 and PDB 2PQR as implemented in the proteinprepare function in HTMD.^52^ A pure 1,2-dipalmitoylphosphatidylcholine (DPPC) membrane 130 × 130 Å then was built using the membrane builder class as part of the HTMD package. DPPC was chosen because it resembles the bulk properties of a Gram-positive bacteria membrane.^53^ After that each of the prepared proteins were embedded into a membrane separately. The systems are then solvated with water and neutralized to a 150 mM NaCl concentration using the charmm.builder­() function. The proteins, ions and lipids were parametrized using CHARMM36m^54^ force field and TIP3P^55^ was used as the water model. The ligands were parametrized using CGENFF.^56^ First the mol2 file for each ligand was generated using Maestro in the Schrodinger suite,^57^ followed by the mol2 files being uploaded to the CGENFF Web server to generate the parameters.

A 40 ns NPT equilibration run at 310 K was performed for each system with a 5 kcal/mol flat bottom group restraints applied to the ligand with a box size of 12 × 12 × 12 to prevent the ligand from drifting away from its initial site during the equilibration step. The rest of the settings were kept the same as the default equilibration protocol in HTMD. In the production protocol we ran the simulation at the NVT ensemble, and the restraints box size was expanded to allow the ligand move inside the channel without diffusing into the solvent. Each system was run as 40 replicates for 300 ns, sampling a cumulative time of 12 μs. The simulation systems and parameters used for saNorA simulations have been specified in Table S5.

### Molecular Dynamic Analysis

After simulation, trajectories were aligned to the reference structure for further analysis. Mdpocket^58^ was used to detect pockets existence of the protein. The center of mass (COM) of the substrate binding pocket was estimated by residue I19, V44, S81, and N340. F16, I136, N137, F140, E222, F303, and F306 were used to define the COM of the ligand binding pocket. Distances between the COM of the ligands and the binding pockets were calculated using MDAnalysis.^59^ Frames within 8 Å to the pockets were considered as binding and extracted for further analysis. Selected frames are projected using Principal Component Analysis (PCA) provided by PyEMMA,^60^ with the distance from the ligand to the previously mentioned pocket residues as the only input feature. A relatively stable binding structure for each ligand is randomly selected from the energy minimum illustrated in the PCA free energy maps. The protein structure and the ligand conformation were extracted from the selected complex, and electrostatic energy analysis was conducted by APBS.^61^ The binding free energy of each ligand is calculated as

$$\Delta G_{\mathrm{binding}}=G_{\mathrm{complex}}-\left(G_{\mathrm{receptor}}+G_{\mathrm{ligand}}\right)$$

### Accumulation Assay

The accumulation assay was performed in triplicate batches of eight samples, with each batch containing either **levofloxacin (7)** or **KSN-L22 (46)**. *S. aureus* 1199 and *S. aureus* 1199B were used in these experiments to investigate the impact of upregulated NorA-mediated efflux. Minor adjustments were made to the protocol previously described by Geddes and Hergenrother.^62^ After centrifugation in oil to remove excess compound, the pellets were dissolved in 200 μL of ultrapure sterile water and subjected to five freeze–thaw cycles. Each cycle consisted of 1 min in a dry ice and ethanol bath, followed by vortexing and 3 min in a heat block at 65 °C. Miles Misra viable counts were used to determine the number of colony-forming units (CFUs) for each replicate. These counts were performed for the 0.55 OD inoculum, postcompound incubation, solvent-only control, and lysates.

The samples were filtered through a 0.45 μm PTFE filter and analyzed using an Agilent InfinityLab LC/MSD System, consisting of an Agilent 1290 Infinity II Analytical-Scale LC coupled to a 6120 Quadrupole mass spectrometer. A 20 μL sample was injected into an Onyx monolithic C18 column and run using a gradient method. The mobile phase began with 10% acetonitrile and 90% water, with the acetonitrile content increased to 90% over 7 min, maintained for 2 min, and then returned to 10%. The area under the curve was automatically calculated using Agilent ChemStation software to determine the level of accumulation within the bacterial cells. Finally, the data were analyzed using GraphPad Prism 10.4.1. Unpaired *t* test was conducted to evaluate the statistical significance of the level of efflux in the efflux mutant *S. aureus* 1199B strain compared to the wild-type *S. aureus* 1199 strain.

### Organic Synthesis

#### Synthesis of Ciprofloxacin (**1**) and Norfloxacin (**2**)

**Ciprofloxacin** (from **1a** via **1b**–**d**) and norfloxacin were synthesized using our previously reported protocol.^32^

### Synthesis of First-Generation ERB-Fluoroquinolones

#### Synthesis of **ML-77-005** (**8**) and Regioisomers

##### 1-Cyclopropyl-6-fluoro-7-(4-(naphthalen-1-ylmethyl)­piperazin-1-yl)-4-oxo-1,4-dihydroquinoline-3-carboxylic acid (**ML-77-005**, **8**)

**General procedure 1: Ciprofloxacin (1)** (100 mg, 0.30 mmol, 1 equiv) was added to a 1:1 mix of acetonitrile and distilled water (10 mL total). After stirring for 5 min, potassium carbonate (125 mg, 0.91 mmol, 3 equiv) was added and the mixture stirred for a further 5 min. Once fully dissolved, 1-(bromomethyl)­naphthalene (49 mg, 0.29 mmol, 0.95 equiv) was added slowly over the course of 15 min and the mixture subsequently stirred at room temperature until completion. The product was then extracted with dichloromethane (2 × 20 mL) using a 1 M solution of citric acid to neutralize the aqueous phase. Combined organic fractions were washed with distilled water (20 mL), dried over MgSO_4_, filtered and concentrated *in vacuo* to give the crude product. Purification was achieved using an SCX-2 catch and release cartridge to afford pure **ML-77-005 (8)** as an off-white solid, 43.3 mg (30.4% yield). For biological testing, this was converted to the HCl salt using **general procedure 2:** the freebase was dissolved in dichloromethane (2 mL), stirred for 5 min, then 4 M HCl in dioxane (530 μL, 2.12 mmol, 20 equiv) added and the flask sealed and stirred for 1 h. The mixture was concentrated *in vacuo*, washed with hexane (3 × 30 mL) and lyophilized to afford **8** salt as a pale yellow solid; ^**1**^ **H NMR (400 MHz, CDCl**_**3**_) δ 15.07 (s, 1H), 8.77 (s, 1H), 8.34 (d, *J* = 8.56 Hz, 1H), 8.03 (d, *J* = 13.09 Hz, 1H), 7.89 (d, *J* = 7.30 Hz, 1H), 7.83 (d, *J* = 7.55 Hz, 1H), 7.49–7.58 (m, 2H), 7.41–7.49 (m, 2H), 7.33 (d, *J* = 7.05 Hz, 1H), 4.02 (s, 2H), 3.49 (br. s., 1H), 3.34 (br. s., 4H), 2.76 (br. s., 4H), 1.31–1.39 (m, 2H), 1.14–1.22 (m, 2H); ^**13**^C NMR (101 MHz, DMSO-d**_6_**) δ 176.4 (d, *J* = 2.67 Hz), 165.8, 152.8 (d, *J* = 249.67 Hz), 148.2, 143.7 (d, *J* = 10.30 Hz), 139.1, 135.2, 133.4, 132.2, 131.7, 130.4, 128.8, 127.1, 126.3, 125.6, 125.4, 124.1, 119.3 (d, *J* = 7.44 Hz), 111.2 (d, *J* = 22.89 Hz), 106.9, 54.9, 50.4, 46.2 (d, *J* = 4.20 Hz), 35.9, 7.6; **LC-MS retention time** 3.10 min (method A) and 6.00 min (method B), purity ≥ 98% (both), found 472.0 [M + H]^+^ (method A) and 472.0 [M + H]^+^ (method B), calculated for C_28_H_26_FN_3_O_3_ 472.2 [M + H]^+^; **HRMS** observed 472.2023 [M + H]^+^, theoretical value 472.2031 [M + H]^+^.

##### Methyl (*S*)-(2-((tert-butoxycarbonyl)­amino)-3-(naphthalen-1-yl)­propanoyl) glycinate (**9**)

(*S*)-2-((tert-butoxycarbonyl)­amino)-3-(naphthalen-1-yl)­propanoic acid (500 mg, 1.59 mmol, 1 equiv) was dissolved in dichloromethane (10 mL), then N,N-diisopropylethylamine (1.38 mL, 7.93 mmol, 5 equiv) was added and the mixture cooled to 0 °C. Then HATU (1.21 g, 3.17 mmol, 2 equiv) was added portionwise and the mixture stirred for 15 min more. Methyl glycinate hydrochloride (398 mg, 3.17 mmol, 2 equiv) was added and the mixture allowed to warm to room temperature and stirred for 5 days. Dichloromethane (20 mL) and ethyl acetate (10 mL) were then added and the mixture washed with saturated sodium hydrogen carbonate solution (30 mL), 1 M citric acid solution (30 mL) and brine (2 × 30 mL). The organic phase was dried over MgSO_4_, filtered and concentrated *in vacuo*. The crude was recrystallized from hot acetone to yield **9**, white solid, 360 mg (58.8% yield); ^**1**^ **H NMR (400 MHz, CDCl**_**3**_) δ 8.15 (d, *J* = 8.34 Hz, 1H, H7), 7.86 (d, *J* = 7.79 Hz, 1H, H10), 7.77 (d, *J* = 8.07 Hz, 1H, H11), 7.53–7.59 (m, 1H, H8), 7.47–7.53 (m, 1H, H9), 7.31–7.42 (m, 2H, H12 + 13), 6.22 (br. s., 1H, H14), 5.19 (br. s., 1H, H13), 4.54 (q, *J* = 7.06 Hz, 1H, H5), 3.94 (d, *J* = 16.69 Hz, 1H, H2), 3.73–3.85 (m, 1H, H2), 3.69 (s, 3H, H1), 3.39–3.64 (m, 2H, H6), 1.38 (br. s., 9H, H15 + 16 + 17); ^**13**^ **C NMR (101 MHz, CDCl**_**3**_) δ 171.5 (s, D), 169.6 (s, B), 155.3 (s, Q), 133.9 (s, M), 132.8 (s, G), 131.9 (s, H), 128.8 (s, L), 127.8 (s, N), 127.7 (s, P), 126.5 (s, J), 125.8 (s, K), 125.4 (s, O), 123.5 (s, I), 80.2 (s, R), 55.4 (s, E), 52.3 (s, A), 41.1 (s, C), 35.9 (s, F), 28.2 (s, S + T + U).

##### (*S*)-3-(Naphthalen-1-ylmethyl)­piperazine-2,5-dione (**10**)

**Compound 9** (327 mg, 0.85 mmol, 1 equiv) was added to a 20 mL capacity microwave vessel fitted with a magnetic stirrer bar and dissolved in a 1:1 mix of methanol and distilled water (8 mL total). The mixture was microwaved at 200 °C for 20 min. Upon cooling, recrystallization of the product was observed; vacuum filtration and drying *in vacuo* yielded **10**, white solid, 164 mg (76.3% yield); **LC-MS** retention time 2.36 min (method A), purity ≥ 98%, found 255.0 [M + H]^+^, calculated for C_15_H_14_N_2_O_2_ 255.1 [M + H]^+^. Characterization data in agreement with published data.^63^

##### (*S*)-2-(Naphthalen-1-ylmethyl)­piperazine (**11**)

**Compound 10** (154 mg, 0.60 mmol, 1 equiv) was suspended in anhydrous tetrahydrofuran (5 mL) and the mixture cooled to 0 °C. Then lithium aluminum hydride (1 M in tetrahydrofuran; 2.41 mL, 0.60 mmol, 16 equiv) was added dropwise and the mixture left to stir at 0 °C for 20 min before being allowed to warm to room temperature and stirred for 6 days. The mixture was then cooled to −78 °C with stirring and distilled water (200 μL) added slowly, followed by 1 M hydrochloric acid solution (1.2 mL) and more distilled water (600 μL). The mixture was allowed to warm to room temperature and stirred for 30 min, after which dichloromethane (20 mL) was added and the mixture stirred for a further 30 min. After addition of more 1 M hydrochloric acid solution (20 mL), the organic layer was separated and the remaining aqueous layer washed with dichloromethane (2 × 20 mL). The aqueous layer was then basified and the product extracted using dichloromethane (4 × 20 mL). The latter organics were combined, dried over Na_2_SO_4_, filtered and concentrated *in vacuo*. Purification was achieved using an SCX-2 catch and release cartridge (see **Solid Phase Extraction**) to afford **11**, white solid, 38.0 mg (27.7% yield); ^**1**^ **H NMR (400 MHz, CDCl**_**3**_) δ 8.06 (d, *J* = 7.61 Hz, 1H), 7.86 (d, *J* = 6.42 Hz, 1H), 7.75 (d, *J* = 7.61 Hz, 1H), 7.44–7.60 (m, 2H), 7.30–7.44 (m, 2H), 3.14–3.29 (m, 1H), 2.99–3.13 (m, 2H), 2.84–2.99 (m, 3H), 2.80 (t, *J* = 11.46 Hz, 1H), 2.52–2.72 (m, 2H), 2.09 (br. s., 2H); ^**13**^ **C NMR (101 MHz, CDCl**_**3**_) δ 134.2, 133.9, 132.0, 128.7, 127.4, 127.2, 125.9, 125.6, 125.3, 123.8, 56.5, 52.5, 47.1, 46.1, 37.9; **LC-MS** retention time 1.52 min (method A), purity = 95%, found 227.1 [M + H]^+^, calculated for C_15_H_18_N_2_ 227.2 [M + H]^+^; **[α]**_**D**_^**25.0**^ = +46 deg mL g^–1^ dm^–1^ (c = 0.076 g/100 mL, CH_2_Cl_2_). Characterization data in agreement with published data.^63^

##### (*S*)-1-Cyclopropyl-6-fluoro-7-(3-(naphthalen-1-ylmethyl)­piperazin-1-yl)-4-oxo-1,4-dihydroquinoline-3-carboxylic acid (**12**)

General procedure 3, starting from **11** and using 140 °C microwave heating, was used to afford crude **12**. Upon cooling, the mixture was filtered through a Mini-UniPrep polypropylene filter (0.45 μm pore size) and purified directly using mass-directed preparative HPLC. Pure fractions were collected and freeze-dried overnight to afford **12**, light brown solid, 15.8 mg (49.9% yield); ^**1**^ **H NMR (400 MHz, CDCl**_**3**_) δ 8.70 (s, 1H, H3), 8.10 (d, *J* = 8.16 Hz, 1H), 7.96 (d, *J* = 13.20 Hz, 1H), 7.86–7.92 (m, 1H), 7.77–7.83 (m, 1H), 7.48–7.60 (m, 2H), 7.39–7.48 (m, 2H), 7.18 (d, *J* = 7.15 Hz, 1H), 3.71 (d, *J* = 10.91 Hz, 1H, H10), 3.64 (d, *J* = 11.55 Hz, 1H, H9), 3.37–3.47 (m, 1H, H13), 3.26–3.37 (m, 2H, H4 + 14), 3.16–3.25 (m, 1H, H14), 3.04–3.16 (m, 2H, H10 + 11), 2.95–3.04 (m, 1H, H11), 2.91 (t, *J* = 10.73 Hz, 1H, H9), 1.13–1.23 (m, 2H, H5 + 6), 1.01–1.10 (m, 2H, H5 + 6); ^**13**^ **C NMR (101 MHz, CDCl**_**3**_) δ 177.0 (d, *J* = 2.48 Hz, G), 167.1 (s, A), 153.5 (d, *J* = 251.20 Hz, J), 147.2 (s, C), 145.6 (d, *J* = 10.11 Hz, K), 139.0 (s, M), 134.0 (s, S), 133.6 (s, W), 132.0 (s, AB), 128.9 (s, X), 127.7 (s, V), 127.6 (s, T), 126.2 (s, Z), 125.8 (s, Y), 125.4 (s, U), 123.6 (s, AA), 119.5 (d, *J* = 7.82 Hz, H), 112.4 (d, *J* = 23.27 Hz, I), 108.0 (s, B), 104.7 (d, *J* = 3.24 Hz, L), 55.5 (d, *J* = 3.62 Hz, N), 55.1 (s, Q), 50.5 (d, *J* = 6.48 Hz, O), 45.6 (s, P), 37.5 (s, R), 35.1 (s, D), 8.1 (s, E/F), 8.0 (s, E/F); ^**19**^ **F­{** ^**1**^ **H} NMR (376 MHz, CDCl**_**3**_) δ −120.7 (s, 1F); **LC-MS** retention time 2.63 min (method A) and 5.60 min (method B), purity ≥ 98% (both), found 472.1 [M + H]^+^ (both), calculated for C_28_H_26_FN_3_O_3_ 472.2 [M + H]^+^; **HRMS** observed 472.2026 [M + H]^+^, theoretical value 472.2031 [M + H]^+^; **[α]**_**D**_^**25.0**^ = +61 deg mL g^–1^ dm^–1^ (c = 0.057 g/100 mL, CH_2_Cl_2_).

##### 2,4,5-Trifluorobenzoic acid (**13**)

1-Bromo-2,4,5-trifluorobenzene (5.29 g, 25.1 mmol, 1 equiv) was dissolved in anhydrous diethyl ether (75 mL) and the solution cooled to −78 °C over 1 h. *n*-Butyllithium (2.5 M solution in hexanes; 10.0 mL, 25.1 mmol, 1 equiv) was added dropwise over 15 min and the mixture was then stirred for a further hour at −78 °C. Carbon dioxide was then bubbled through the mixture for 30 min at −78 °C before the reaction was allowed to warm to room temperature over 30 min. The reaction was quenched through dropwise addition of 1 M HCl (3 mL) at −78 °C, then allowed to warm to room temperature and basified with 2 M NaOH. The aqueous layer was washed three times with dichloromethane, then acidified with 3 M HCl and the product extracted with three more dichloromethane washes. The combined organics were dried over Na_2_SO_4_, filtered and concentrated *in vacuo* to give **13** which was used in subsequent reactions without further purification; white solid, 3.47 g (78.6% yield); ^**1**^ **H NMR (400 MHz, CDCl**_**3**_) δ 11.63 (br. s., 1H, H6), 7.83–7.95 (m, 1H, H1), 7.07 (td, *J* = 6.24, 9.81 Hz, 1H, H4); ^**13**^ **C NMR (101 MHz, CDCl**_**3**_) δ 168.0–168.1 (m, G), 158.7 (ddd, *J* = 2.48, 10.11, 263.02 Hz, E), 154.1 (ddd, *J* = 12.21, 14.49, 261.11 Hz, C), 146.5 (ddd, *J* = 3.81, 12.59, 247.57 Hz, B), 120.6 (dt, *J* = 2.10, 20.60 Hz, A), 113.8 (ddd, *J* = 3.81, 5.15, 9.35 Hz, F), 107.4 (dd, *J* = 20.98, 28.04 Hz, D); ^**19**^ **F­{** ^**1**^ **H} NMR (376 MHz, CDCl**_**3**_) δ −107.6 (dd, *J* = 9.88, 16.01 Hz, 1F, F5), −122.7 (dd, *J* = 9.88, 21.46 Hz, 1F, F2), −140.7 (dd, *J* = 16.01, 21.46 Hz, 1F, F3); **LC-MS** retention time 2.79 min (method A), purity = 93%, found 175.1 [M – H]^−^, calculated for C_7_H_3_F_3_O_2_ 175.1 [M – H]^−^. Characterization data in agreement with published data.^64^

##### 2,4,5-Trifluoro-3-(hydroxy­(naphthalen-1-yl)­methyl)­benzoic acid (**14**)

**Compound 13** (3.32 g, 18.8 mmol, 1 equiv) was dissolved in anhydrous tetrahydrofuran (100 mL) and the solution was cooled to −78 °C over 1 h. *n*-Butyllithium (1.6 M solution in hexanes; 24.8 mL, 39.6 mmol, 2.1 equiv) was added dropwise over 1 h and the mixture was then stirred for a further 30 min at −78 °C. 1-Naphthaldehyde (2.81 mL, 20.7 mmol, 1.1 equiv) was then added dropwise over 10 min and the mixture stirred for a further 1 h at −78 °C before being allowed to warm to room temperature. The reaction was quenched through dropwise addition of 1 M NaOH. The mixture was extracted with dichloromethane (3 × 50 mL); organic layers were combined, dried over MgSO_4_, filtered and concentrated *in vacuo*. The crude product was redissolved in dimethyl sulfoxide (3 mL), filtered through a Mini-UniPrep polypropylene filter (0.45 μm pore size) and purified directly using mass-directed preparative HPLC. Pure fractions were collected and freeze-dried overnight to afford **14**, off-white solid, 1.50 g (24.0% yield); ^**1**^ **H NMR (400 MHz, CDCl**_**3**_) δ 8.15 (d, *J* = 5.96 Hz, 1H), 7.74–8.01 (m, 3H), 7.39–7.68 (m, 4H), 6.99 (s, 1H);^**13**^ **C NMR (101 MHz, CDCl**_**3**_) δ 164.2 (d, *J* = 3.24 Hz), 155.1 (ddd, *J* = 2.86, 11.06, 265.12 Hz), 154.7 (ddd, *J* = 10.30, 13.35, 261.69 Hz), 141.6 (ddd, *J* = 4.58, 14.88, 253.87 Hz), 137.8 (dt, *J* = 2.86, 15.45 Hz), 133.9, 131.2, 131.0, 129.0, 128.8, 127.3, 126.4, 125.1, 125.0, 122.8, 111.4 (ddd, *J* = 1.53, 2.29, 16.02 Hz), 108.0 (dd, *J* = 22.89, 24.60 Hz), 77.3; ^**19**^ **F­{** ^**1**^ **H} NMR (376 MHz, CDCl**_**3**_) δ −111.9 to −112.1 (m, 1F), −125.7 to −125.9 (m, 1F), −139.3 to −139.5 (m, 1F); **LC-MS** retention time 3.07 min (method A), purity ≥ 98%, found 331.0 [M – H]^−^, calculated for C_18_H_11_F_3_O_3_ 331.1 [M – H]^−^.

##### 2,4,5-Trifluoro-3-(naphthalen-1-ylmethyl)­benzoic acid (**15**)

Trifluoroacetic acid (75 mL) was added to a 500 mL beaker with magnetic stirrer bar and cooled to −10 °C in a brine/ice bath. Sodium borohydride (1.40 g, 36.2 mmol, 8 equiv) was added portionwise ensuring that the temperature of the mixture did not exceed 5 °C at any time. Then **14** (1.50 g, 4.53 mmol, 1 equiv) in dichloromethane (20 mL) was added dropwise and the mixture stirred at −10 °C for 18 h. The reaction was quenched by dropwise addition to ice and the resulting precipitate was vacuum filtered, washed with distilled water (3 × 10 mL) and redissolved in dichloromethane (20 mL). This was dried over MgSO_4_, filtered and concentrated *in vacuo* to give **15** which was used in subsequent reactions without further purification; off-white solid, 1.28 g (89.6% yield); ^**1**^ **H NMR (400 MHz, (CD**_**3**_)_**2**_ **CO)** δ 11.83 (br. s., 1H, H2), 8.28 (d, *J* = 8.53 Hz, 1H, H7), 7.95 (d, *J* = 8.16 Hz, 1H, H10), 7.78–7.89 (m, 2H, H1 + 11), 7.63 (ddd, *J* = 1.38, 6.99, 8.32 Hz, 1H, H8), 7.52–7.59 (m, 1H, H9), 7.37–7.44 (m, 1H, H12), 7.14 (d, *J* = 7.15 Hz, 1H, H13), 4.62 (s, 2H, H6); ^**13**^ **C NMR (101 MHz, (CD**_**3**_)_**2**_ **CO)** δ 163.8–163.9 (m, G), 134.9 (s, O), 134.4 (s, I), 132.6 (s, J), 129.7 (s, N), 128.4 (s, P), 127.4 (s, L), 126.8 (s, M), 126.5 (s, Q), 126.2 (s, R), 124.1 (s, K), 120.4 (dd, *J* = 16.59, 21.93 Hz, D), 118.7 (d, *J* = 20.79 Hz, A), 116.3–116.6 (m, F), 26.2 (s, H); ^**19**^ **F­{** ^**1**^ **H} NMR (376 MHz, (CD**_**3**_)_**2**_ **CO)** δ −114.2 (dd, *J* = 8.51, 16.35 Hz, 1F, F5), −131.1 (dd, *J* = 8.51, 21.46 Hz, 1F, F3), −143.1 (dd, *J* = 16.35, 21.46 Hz, 1F, F4); **LC-MS** retention time 3.61 min (method A), purity = 93%, found 315.0 [M – H]^−^, calculated for C_18_H_11_F_3_O_2_ 315.1 [M – H]^−^.

##### Ethyl 3-(cyclopropylamino)-2-(2,4,5-trifluoro-3-(naphthalen-1-ylmethyl) benzoyl)­acrylate (**16**)

**Compound 15** (1.00 g, 3.16 mmol, 1 equiv) was dissolved in anhydrous dichloromethane (25 mL) and the solution cooled to 0 °C over 30 min. Oxalyl chloride (401 μL, 4.74 mmol, 1.5 equiv) was added dropwise over 15 min followed by N,N-dimethylformamide (5 drops) and the mixture stirred for a further 5 min at 0 °C before being allowed to warm to room temperature. The mixture was stirred at room temperature for 2 weeks. The reaction was then concentrated *in vacuo*, redissolved in toluene (15 mL) and added dropwise to a flask charged with ethyl (*E*)-3-(dimethylamino)­acrylate (549 μL, 3.79 mmol, 1.2 equiv), triethylamine (1.10 mL, 7.90 mmol, 2.5 equiv) and toluene (10 mL). The original flask was then washed with toluene (2 × 5 mL) and the washes added dropwise to the new flask as well. The mixture was heated to 90 °C for 26 h, then allowed to cool to room temperature, concentrated *in vacuo* and redissolved in dichloromethane (30 mL). This was washed with saturated sodium hydrogen carbonate solution (2 × 50 mL), 0.5 M citric acid solution (2 × 50 mL) and brine (1 × 50 mL). The organic layer was dried over MgSO_4_, filtered, concentrated *in vacuo* and redissolved in dichloromethane (10 mL). Then cyclopropylamine (660 μL, 9.49 mmol, 3 equiv) was added and the mixture stirred at room temperature for 1 h. The mixture was washed with 1 M HCl solution (2 × 50 mL), 1 M NaOH solution (2 × 50 mL) and brine (1 × 50 mL), dried over MgSO_4_, filtered and concentrated *in vacuo*. The crude product was purified via automated flash column chromatography using a 50% hexane/50% dichloromethane −100% dichloromethane gradient to yield **16**, colorless oil, 369 mg (25.7% yield over three steps); ^**1**^ **H NMR (400 MHz, CDCl**_**3**_) δ 10.72 (d, *J* = 13.48 Hz, 0.75H, H14), 9.24 (d, *J* = 13.85 Hz, 0.25H, H14), 7.97–8.11 (m, 2H, H6 + 13), 7.73 (d, *J* = 7.98 Hz, 1H, H9), 7.61 (d, *J* = 8.16 Hz, 1H, H10), 7.40–7.49 (m, 1H, H7), 7.32–7.40 (m, 1H, H8), 7.24 (t, *J* = 7.66 Hz, 1H, H11), 7.14–7.20 (m, 0.25H, H1), 7.06–7.14 (m, 1H, H12), 6.98–7.06 (m, 0.75H, H1), 4.37 (s, 2H, H5), 3.78 (q, *J* = 7.15 Hz, 1.5H, H18), 3.60 (q, *J* = 7.09 Hz, 0.5H, H18), 2.69–2.83 (m, 1H, H15), 0.80 (t, *J* = 7.15 Hz, 2.25H, H19), 0.58–0.75 (m, 4H, H16 + 17), 0.55 (t, *J* = 7.11 Hz, 0.75H, H19); ^**13**^ **C NMR (101 MHz, CDCl**_**3**_) δ 188.3 (s, G), 186.2 (s, G), 168.2 (s, T), 166.5 (s, T), 160.5 (s, U), 160.1 (s, U), 154.8 – 154.9 (m, E), 153.0 (ddd, *J* = 2.48, 6.29, 245.86 Hz, E), 152.3–152.5 (m, E), 149.6 (ddd, *J* = 8.96, 14.11, 251.77 Hz, C), 149.4 (ddd, *J* = 8.58, 14.31, 251.01 Hz, C), 146.7 (ddd, *J* = 3.05, 13.54, 245.48 Hz, B), 133.7 (s, O), 133.6 (s, O), 133.4 (s, I), 133.4 (s, I), 131.5 (s, J), 128.7 (s, N), 128.6 (s, N), 127.4 (s, P), 127.2 (s, P), 126.7–127.1 (m, F), 126.2 (s, L), 126.1 (s, L), 125.9 (s, R), 125.8 (s, R), 125.6 (s, M), 125.5 (s, M), 125.3 (s, Q), 125.2 (s, Q), 123.1 (s, K), 117.3 (dd, *J* = 16.40, 23.08 Hz, D), 117.2 (dd, *J* = 16.59, 23.08 Hz, D), 115.1 (dd, *J* = 5.34, 19.65 Hz, A), 114.3 (dd, *J* = 4.96, 20.22 Hz, A), 101.7 (s, S), 59.6 (s, Y), 59.3 (s, Y), 30.3 (s, V), 29.9 (s, V), 25.6 (s, H), 13.8 (s, Z), 13.3 (s, Z), 6.3 (s, W+X); ^**19**^ **F NMR (376 MHz, CDCl**_**3**_) δ −118.8 (m, 0.25F, F4), −120.2 (m, 0.75F, F4), −134.3 (m, 0.25F, F2), −135.3 (m, 0.75F, F2), −142.3 (m, 1F, F3); **LC-MS** retention time 3.97 min (method A), purity = 93%, found 454.2 [M + H]^+^, calculated for C_26_H_22_F_3_NO_3_ 454.2 [M + H]^+^.

##### Ethyl 1-cyclopropyl-6,7-difluoro-8-(naphthalen-1-ylmethyl)-4-oxo-1,4-dihydroquinoline-3-carboxylate (**17**)

**Compound 16** (369 mg, 0.81 mmol, 1 equiv) was dissolved in N,N-dimethylformamide (10 mL), then potassium carbonate (338 mg, 2.44 mmol, 3 equiv) was added and the mixture stirred at 90 °C for 1 h. Upon cooling, the mixture was concentrated *in vacuo*, redissolved in 4:1 ethyl acetate:dichloromethane (100 mL), washed with distilled water (2 × 200 mL) and brine (200 mL), dried over MgSO_4_, filtered, concentrated *in vacuo* and washed with hexanes and diethyl ether to give **17** which was used in subsequent reactions without further purification; yellow solid, 330 mg (93.6% yield); ^**1**^ **H NMR (400 MHz, CDCl**_**3**_) δ 8.50 (s, 1H, H12), 8.37 (t, *J* = 9.54 Hz, 1H, H1), 8.13 (d, *J* = 8.34 Hz, 1H, H5), 7.93 (d, *J* = 7.70 Hz, 1H, H8), 7.76 (d, *J* = 8.34 Hz, 1H, H9), 7.53–7.61 (m, 1H, H6), 7.61–7.68 (m, 1H, H7), 7.23–7.31 (m, 1H, H10), 6.47 (d, *J* = 7.15 Hz, 1H, H11), 5.26 (s, 2H, H4), 4.39 (q, *J* = 7.09 Hz, 2H, H16), 3.31–3.42 (m, 1H, H13), 1.40 (t, *J* = 7.15 Hz, 3H, H17), 0.94–1.08 (m, 4H, H14 + 15); ^**13**^ **C NMR (101 MHz, CDCl**_**3**_) δ 172.9 (s, G), 165.1 (s, T), 153.1 (dd, *J* = 13.92, 251.77 Hz, C), 151.8 (s, U), 148.7 (dd, *J* = 16.21, 251.39 Hz, B), 138.0 (dd, *J* = 2.29, 4.20 Hz, E), 133.9 (s, O), 133.8 (s, I), 131.0 (s, J), 129.2 (s, N), 127.6 (s, P), 127.4–127.5 (m, F), 126.8 (s, L), 126.1 (s, M), 125.7 (s, Q), 123.7 (s, R), 122.1 (s, K), 118.7 (d, *J* = 15.26 Hz, D), 113.9 (dd, *J* = 2.86, 18.50 Hz, A), 110.7 (s, S), 61.1 (s, Y), 38.3 (s, V), 28.3 (d, *J* = 9.16 Hz, H), 14.4 (s, Z), 11.2 (s, W + X); ^**19**^ **F­{** ^**1**^ **H} NMR (376 MHz, CDCl**_**3**_) δ −127.9 (d, *J* = 22.82, 1F, F2), −137.1 (d, *J* = 22.82 Hz, 1F, F3); **LC-MS** retention time 3.53 min (method A), purity = 96%, found 434.1 [M + H]^+^, calculated for C_26_H_21_F_2_NO_3_ 434.2 [M + H]^+^.

##### 1-Cyclopropyl-6,7-difluoro-8-(naphthalen-1-ylmethyl)-4-oxo-1,4-dihydro quinoline-3-carboxylic acid (**18**)

**Compound 17** (330 mg, 0.76 mmol, 1 equiv) was added to ethanol (5 mL) and 15% w/v sodium hydroxide solution (5 mL) and the mixture was stirred at room temperature for 4 h. Upon completion, the mixture was neutralized using 3 M hydrochloric acid solution before extraction with dichloromethane (3 × 20 mL). Combined organics were dried over MgSO_4_, filtered, concentrated *in vacuo* and washed with hexanes to give **18**, which was used in subsequent reactions without further purification; peach solid, 232 mg (75.2% yield); ^**1**^ **H NMR (400 MHz, CDCl**_**3**_) δ 14.36 (br. s., 1H, H16), 8.76 (s, 1H, H12), 8.40 (t, *J* = 9.17 Hz, 1H, H1), 8.13 (d, *J* = 8.44 Hz, 1H, H5), 7.95 (d, *J* = 7.79 Hz, 1H, H8), 7.78 (d, *J* = 8.25 Hz, 1H, H9), 7.63–7.71 (m, 1H, H6), 7.55–7.63 (m, 1H, H7), 7.23–7.31 (m, 1H, H10), 6.43 (d, *J* = 7.15 Hz, 1H, H11), 5.33 (s, 2H, H4), 3.46–3.57 (m, 1H, H13), 1.09–1.16 (m, 2H, H14 + 15), 1.00–1.09 (m, 2H, H14 + 15); ^**13**^ **C NMR (101 MHz, CDCl**_**3**_) δ 177.4 (d, *J* = 2.29 Hz, G), 165.9 (s, T), 154.1 (dd, *J* = 13.92, 254.44 Hz, C), 151.4 (s, U), 149.2 (dd, *J* = 16.21, 254.82 Hz, B), 138.9 (dd, *J* = 2.10, 4.19 Hz, E), 133.9 (s, O), 133.4 (s, I), 130.9 (s, J), 129.3 (s, N), 127.9 (s, P), 127.0 (s, L), 126.3 (s, M), 125.6 (s, Q), 124.8–124.9 (m, F), 123.5 (s, R), 122.0 (s, K), 119.8 (d, *J* = 15.26 Hz, D), 113.3 (dd, *J* = 3.05, 18.50 Hz, A), 108.6 (s, S), 39.4 (s, V), 28.5 (dd, *J* = 1.34, 8.39 Hz, H), 11.4 (s, W + X); ^**19**^ **F­{** ^**1**^ **H} NMR (376 MHz, CDCl**_**3**_) δ −123.8 (d, *J* = 22.82, 1F, F2), −134.3 (d, *J* = 22.82 Hz, 1F, F3); **LC-MS** retention time 3.54 min (method A), purity = 96%, found 406.1 [M + H]^+^, calculated for C_24_H_17_F_2_NO_3_ 406.1 [M + H]^+^.

##### 1-Cyclopropyl-6-fluoro-8-(naphthalen-1-ylmethyl)-4-oxo-7-(piperazin-1-yl)-1,4-dihydroquinoline-3-carboxylic acid (**19**)

**Compound 18** (46.0 mg, 0.11 mmol, 1 equiv) was partially dissolved in tetrahydrofuran (2 mL), then potassium carbonate (24.0 mg, 0.17 mmol, 1.5 equiv) was added and the mixture stirred at room temperature for 5 min. Boron trifluoride diethyl etherate (17.0 μL, 0.14 mmol, 1.2 equiv) was added dropwise and the mixture stirred for a further 5 min at room temperature, then heated to 70 °C for 5 h. Upon cooling, the mixture was diluted with diethyl ether (30 mL), filtered and washed with diethyl ether (3 × 10 mL). The crude difluoroborane ester was redissolved in dichloromethane and concentrated *in vacuo*, then dissolved in acetonitrile (1 mL) with piperazine (11.0 mg, 0.13 mmol, 2.1 equiv) and heated to 50 °C for 16 h. Upon cooling, the mixture was concentrated *in vacuo*, redissolved in ethanol, triethylamine (50 μL) was added and the mixture heated to reflux for 1 h. The crude product was then concentrated *in vacuo*, resuspended in dimethyl sulfoxide, filtered and dried under vacuum to afford **19**, off-white solid, 12.1 mg (22.7% yield over three steps); ^**1**^H NMR (400 MHz, DMSO-d_6_) δ 8.73 (s, 1H, H10), 8.37 (d, *J* = 8.16 Hz, 1H, H3), 8.08 (d, *J* = 11.74 Hz, 1H, H1), 7.97 (d, *J* = 8.80 Hz, 2H, H6), 7.75 (d, *J* = 8.07 Hz, 1H, H7), 7.65 (t, *J* = 7.47 Hz, 1H, H4), 7.59 (t, *J* = 7.38 Hz, 1H, H5), 7.22 (t, *J* = 7.70 Hz, 1H, H8), 6.25 (d, *J* = 7.06 Hz, 1H, H9), 5.44 (br. s., 2H, H2), 3.74 (br. s., 1H, H11), 2.88 (s, 4H, H15 + 16), 1.15 (q, *J* = 5.72 Hz, 2H, H12 + 13), 1.00 (br. s., 2H, H12 + 13); ^**13**^C NMR (101 MHz, DMSO-d_6_ + CF_**3**_ **CO**_**2**_ **H)** δ 177.0 (d, *J* = 1.72 Hz), 165.3, 158.7 (d, *J* = 251.39 Hz), 152.7, 143.9 (d, *J* = 12.78 Hz), 139.8, 136.9, 133.3, 132.6–132.7 (m), 131.0, 128.8, 126.5, 126.5, 126.0, 125.6, 123.4 (d, *J* = 14.88 Hz), 117.0–117.2 (m), 114.1–114.2 (m), 110.7 (d, *J* = 23.46 Hz), 107.4, 47.6 (d, *J* = 5.15 Hz), 43.3, 35.9, 31.4, 10.8; ^**19**^ **F NMR** (**376 MHz**, **DMSO-d_6_** + **CF**_**3**_ **CO**_**2**_ **H)** δ −120.2 (d, *J* = 11.24 Hz, 1F); ^**19**^ **F­{** ^**1**^ **H**} **NMR** (**376 MHz**, **DMSO-d_6_** + **CF**_**3**_ **CO**_**2**_ **H)** δ −120.2 (s, 1F); **LC-MS** retention time 2.60 min (method A) and 5.55 min (method B), purity = 95% (both), found 472.1 [M + H]^+^ (method A) and 472.2 [M + H]^+^ (method B), respectively, calculated for C_28_H_26_FN_3_O_3_ 472.2 [M + H]^+^; **HRMS** observed 472.2022 [M + H]^+^, theoretical value 472.2031 [M + H]^+^.

#### Synthesis of Wider First Generation ERB-Fluoroquinolones

##### 1-Ethyl-6-fluoro-7-(4-(naphthalen-1-ylmethyl)­piperazin-1-yl)-4-oxo-1,4-dihydroquinoline-3-carboxylic acid (**20**)

General procedures 1 and 2, starting from norfloxacin (**2**), afforded pure **20** and HCl salt **20 salt**, tan solid, 1.00 g (69.5% yield); ^**1**^ **H NMR (400 MHz, CDCl**_**3**_ **)** δ 15.13 (s, 1H), 8.66 (s, 1H), 8.33 (d, *J* = 8.06 Hz, 1H), 8.07 (d, *J* = 13.09 Hz, 1H), 7.89 (d, *J* = 7.05 Hz, 1H), 7.82 (d, *J* = 7.30 Hz, 1H), 7.49–7.57 (m, 2H), 7.45 (q, *J* = 7.22 Hz, 2H), 6.81 (d, *J* = 7.05 Hz, 1H), 4.23–4.32 (m, 2H), 4.02 (s, 2H), 3.33 (br. s., 4H), 2.76 (br. s., 4H), 1.56 (t, *J* = 6.92 Hz, 3H); ^**13**^C NMR (101 MHz, DMSO-d_6_) δ 176.1 (d, *J* = 2.48 Hz), 166.1, 152.9 (d, *J* = 249.67 Hz), 148.3, 145.5 (d, *J* = 10.30 Hz), 137.1, 133.6, 133.5, 132.0, 128.2, 127.8, 127.5, 125.7, 125.7, 125.1, 124.8, 119.2 (d, *J* = 7.82 Hz), 111.0 (d, *J* = 22.89 Hz), 107.0, 105.9 (d, *J* = 3.43 Hz), 54.9, 52.4, 49.6 (d, *J* = 4.39 Hz), 49.0, 14.3; **LC-MS retention time** 3.05 min (method A) and 5.85 min (method B), purity ≥ 98% (method A) and 93% (method B), found 460.0 [M + H]^+^ (method A) and 460.1 [M + H]^+^ (method B), calculated for C_27_H_26_FN_3_O_3_ 460.2 [M + H]^+^; **HRMS** observed 460.2020 [M + H]^+^, theoretical value 460.2031 [M + H]^+^.

##### 1-Ethyl-6-fluoro-7-(4-(naphthalen-1-ylmethyl)­piperazin-1-yl)-4-oxo-1,4-dihydro-1,8-naphthyridine-3-carboxylic acid (**21**)

General procedures 1 and 2, starting from enoxacin (**3**), afforded pure **21** and HCl salt **21 salt**, light brown solid, 114 mg (79.2% yield); ^**1**^ **H NMR (400 MHz, CDCl**_**3**_ **)** δ 15.11 (br. s., 1H), 8.69 (s, 1H), 8.33 (dd, *J* = 1.64, 7.93 Hz, 1H), 8.09 (d, *J* = 13.35 Hz, 1H), 7.86–7.91 (m, 1H), 7.80–7.86 (m, 1H), 7.49–7.58 (m, 2H), 7.41–7.46 (m, 2H), 4.38 (q, *J* = 7.05 Hz, 2H), 3.99 (s, 2H), 3.84–3.90 (m, 4H), 2.65–2.71 (m, 4H), 1.48 (t, *J* = 7.18 Hz, 3H); ^**13**^C NMR (101 MHz, DMSO-d_6_) δ 177.0, 166.2, 164.9, 148.6, 133.9, 132.6, 132.0–132.1 (m), 130.9–131.0 (m), 129.3, 127.5–127.6 (m), 126.8–126.9 (m), 125.9, 114.2, 108.8, 63.3, 51.0 (d, *J* = 3.81 Hz), 47.7, 44.2, 15.3; **LC-MS retention time** 3.05 min (method A) and 5.85 min (method B), purity ≥ 98% (both), found 461.1 [M + H]^+^ (both), calculated for C_26_H_25_FN_4_O_3_ 461.2 [M + H]^+^; **HRMS** observed 461.1974 [M + H]^+^, theoretical value 461.1983 [M + H]^+^.

##### 1-Cyclopropyl-6-fluoro-7-(4-(naphthalen-2-ylmethyl)­piperazin-1-yl)-4-oxo-1,4-dihydroquinoline-3-carboxylic acid (**23**)

General procedures 1 and 2 afforded **23** and HCl salt **23 salt**, pale yellow solid, 219 mg (81.2% yield); ^**1**^ **H NMR (400 MHz, CDCl**_**3**_) δ 15.05 (br. s., 1H), 8.72 (s, 1H), 7.96 (d, *J* = 13.09 Hz, 1H), 7.80–7.88 (m, 3H), 7.78 (s, 1H), 7.54 (dd, *J* = 1.45, 8.37 Hz, 1H), 7.44–7.52 (m, 2H), 7.34 (d, *J* = 7.18 Hz, 1H), 3.77 (s, 2H), 3.46–3.57 (m, 1H), 3.31–3.44 (m, 4H), 2.67–2.80 (m, 4H), 1.31–1.41 (m, 2H), 1.13–1.21 (m, 2H); ^**13**^C NMR (101 MHz, DMSO-d_6_) δ 176.4 (d, *J* = 1.91 Hz), 165.8, 152.8 (d, *J* = 247.95 Hz), 148.3, 139.1, 136.6, 135.6, 133.1, 132.5, 131.3, 128.4, 128.3, 128.0, 127.7, 127.2, 126.8, 119.4–119.5 (m), 111.2 (d, *J* = 22.89 Hz), 106.9–107.1 (m), 106.9, 58.8, 50.3, 46.2 (d, *J* = 4.20 Hz), 35.9, 7.6; **LC-MS retention time** 2.95 min (method A) and 5.93 min (method B), purity ≥ 98% (method A) and 87% (method B), found 472.0 [M + H]^+^ (method A) and 472.1 [M + H]^+^ (method B), calculated for C_28_H_26_FN_3_O_3_ 472.2 [M + H]^+^; **HRMS** observed 472.2020 [M + H]^+^, theoretical value 472.2031 [M + H]^+^.

##### 1-Ethyl-6-fluoro-7-(4-(naphthalen-2-ylmethyl)­piperazin-1-yl)-4-oxo-1,4-dihydroquinoline-3-carboxylic acid (**24**)

General procedures 1 and 2, starting from norfloxacin (**2**), afforded pure **24** and HCl salt **24 salt**, pale yellow solid, 126 mg (46.1% yield); ^**1**^ **H NMR (400 MHz, CDCl**_**3**_ **)** δ 15.16 (br. s., 1H), 8.64 (s, 1H), 8.00 (d, *J* = 13.09 Hz, 1H), 7.80–7.86 (m, 3H), 7.77 (s, 1H), 7.53 (dd, *J* = 1.64, 8.44 Hz, 1H), 7.45–7.51 (m, 2H), 6.81 (d, *J* = 6.80 Hz, 1H), 4.29 (q, *J* = 7.05 Hz, 2H), 3.77 (s, 2H), 3.32–3.39 (m, 4H), 2.69–2.77 (m, 4H), 1.55 (t, *J* = 7.18 Hz, 3H); ^**13**^C NMR (101 MHz, DMSO-d_6_) δ 176.6 (d, *J* = 2.67 Hz), 166.5, 153.2 (d, *J* = 249.10 Hz), 149.2, 144.5 (d, *J* = 15.26 Hz), 137.6, 135.4, 133.6, 133.0, 131.8, 128.9, 128.7, 128.5, 128.2, 127.6, 127.2, 120.5 (d, *J* = 6.49 Hz), 111.9 (d, *J* = 23.08 Hz), 107.7, 107.0 (d, *J* = 1.91 Hz), 59.3, 50.7–50.8 (m), 49.6, 46.8–46.9 (m), 14.9; **LC-MS retention time** 2.95 min (method A) and 5.95 min (method B), purity ≥ 98% (method A) and 89% (method B), found 460.1 [M + H]^+^ (both), calculated for C_27_H_26_FN_3_O_3_ 460.2 [M + H]^+^; **HRMS** observed 460.2019 [M + H]^+^, theoretical value 460.2031 [M + H]^+^. Characterization data in agreement with published data.^65^

##### 7-(4-Benzylpiperazin-1-yl)-1-cyclopropyl-6-fluoro-4-oxo-1,4-dihydro quinoline-3-carboxylic acid (**25**)

General procedures 1 and 2 afforded pure **25** and HCl salt **25 salt**, pale yellow solid, 112 mg (93.3% yield); ^**1**^ **H NMR (400 MHz, CDCl**_**3**_ **)** δ 15.05 (br. s., 1H), 8.76 (s, 1H), 8.00 (d, *J* = 13.09 Hz, 1H), 7.32–7.42 (m, 5H), 7.27–7.32 (m, 1H), 3.62 (s, 2H), 3.53 (br. s., 1H), 3.29–3.44 (m, 4H), 2.62–2.75 (m, 4H), 1.31–1.44 (m, 2H), 1.20 (br. s., 2H); ^**13**^C NMR (101 MHz, DMSO-d_6_) δ 176.4 (d, *J* = 2.29 Hz), 165.8, 152.8 (d, *J* = 249.10 Hz), 148.2, 143.7 (d, *J* = 11.64 Hz), 139.1, 131.5, 129.5–129.6 (m), 128.8, 119.4, 111.2 (d, *J* = 22.89 Hz), 106.9 (d, *J* = 2.86 Hz), 106.9, 58.6, 50.1, 46.1–46.2 (m), 36.0, 7.6; **LC-MS retention time** 2.83 min (method A) and 5.38 min (method B), purity ≥ 98% (both), found 422.0 [M + H]^+^ (method A) and 422.1 [M + H]^+^ (method B), calculated for C_24_H_24_FN_3_O_3_ 422.2 [M + H]^+^; **HRMS** observed 422.1864 [M + H]^+^, theoretical value 422.1874 [M + H]^+^. Characterization data in agreement with published data.^66^

##### 7-(4-Benzylpiperazin-1-yl)-1-ethyl-6-fluoro-4-oxo-1,4-dihydroquinoline-3-carboxylic acid (**26**)

General procedures 1 and 2, starting from norfloxacin (**2**), afforded pure **26** and HCl salt **26 salt**, off-white solid, 77.0 mg (63.2% yield); ^**1**^ **H NMR (400 MHz, CDCl**_**3**_) δ 15.14 (br. s., 1H), 8.66 (s, 1H), 8.02 (d, *J* = 13.09 Hz, 1H), 7.32–7.38 (m, 4H), 7.28–7.32 (m, 1H), 6.82 (d, *J* = 6.80 Hz, 1H), 4.31 (q, *J* = 7.05 Hz, 2H), 3.61 (s, 2H), 3.31–3.37 (m, 4H), 2.66–2.72 (m, 4H), 1.57 (t, *J* = 6.92 Hz, 3H); ^**13**^C NMR (101 MHz, DMSO-d**_6_**) δ 176.2, 166.0, 152.7 (d, *J* = 248.15 Hz), 148.8, 143.9 (d, *J* = 12.40 Hz), 137.1, 131.5, 129.6, 128.8, 119.6, 111.5 (d, *J* = 23.08 Hz), 107.2, 106.4–106.5 (m), 58.6, 50.1, 49.1, 46.2–46.3 (m), 14.5; **LC-MS retention time** 2.95 min (method A) and 5.37 min (method B), purity ≥ 98% (both), found 410.0 [M + H]^+^ (both), calculated for C_23_H_24_FN_3_O_3_ 410.2 [M + H]^+^; **HRMS** observed 410.1863 [M + H]^+^, theoretical value 410.1874 [M + H]^+^. Characterization data in agreement with published data.^67^

##### 1-Cyclopropyl-6-fluoro-4-oxo-7-(4-(quinolin-8-ylmethyl)­piperazin-1-yl)-1,4-dihydroquinoline-3-carboxylic acid (**27**)

General procedures 1 and 2 afforded pure **27** and HCl salt **27 salt**, tan solid, 61.3 mg (45.2% yield); ^**1**^H NMR (400 MHz, DMSO-d**_6_**) δ 15.10 (br. s., 1H), 9.04 (s, 1H), 8.64 (s, 1H), 8.50 (d, *J* = 7.52 Hz, 1H), 8.26 (br. s., 1H), 8.13 (d, *J* = 6.79 Hz), 1H), 7.89 (d, *J* = 13.02 Hz, 1H), 7.73 (t, *J* = 7.06 Hz, 1H), 7.62–7.70 (m, 1H), 7.56 (d, *J* = 6.24 Hz, 1H), 5.01 (br. s., 2H), 3.82 (br. s., 1H), 3.53–3.78 (m, 4H), 1.29 (br. s., 2H), 1.16 (br. s., 2H); ^**13**^C NMR (101 MHz, DMSO-d**_6_**) δ 176.3 (d, *J* = 2.10 Hz), 165.8, 152.8 (d, *J* = 249.86 Hz), 150.7, 148.1, 146.3, 143.8–143.9 (m), 139.0, 136.9, 133.2–133.3 (m), 130.0–130.1 (m), 128.0, 126.3, 122.0, 119.2 (d, *J* = 9.35 Hz), 111.1 (d, *J* = 23.08 Hz), 106.8, 106.7 (d, *J* = 1.91 Hz), 54.0–54.1 (m), 50.9–51.0 (m), 46.3–46.6 (m), 35.9, 7.6; **LC-MS retention time** 2.88 min (method A) and 5.62 min (method B), purity ≥ 98% (both), found 473.0 [M + H]^+^ (method A) and 473.1 [M + H]^+^ (method B), calculated for C_27_H_25_FN_4_O_3_ 473.2 [M + H]^+^; **HRMS** observed 473.1974 [M + H]^+^, theoretical value 473.1983 [M + H]^+^.

##### 1-Ethyl-6-fluoro-4-oxo-7-(4-(quinolin-8-ylmethyl)­piperazin-1-yl)-1,4-dihydroquinoline-3-carboxylic acid (**28**)

General procedures 1 and 2, starting from norfloxacin (**2**), afforded pure **28** and HCl salt **28 salt**, off-white solid, 67.5 mg (49.3% yield); ^**1**^ **H NMR (400 MHz, CDCl**_**3**_ **)** δ 15.15 (br. s., 1H), 8.96 (dd, *J* = 1.76, 4.28 Hz, 1H), 8.67 (s, 1H), 8.19 (dd, *J* = 1.76, 8.31 Hz, 1H), 8.06 (d, *J* = 13.35 Hz, 1H), 7.90 (d, *J* = 7.05 Hz, 1H), 7.78 (dd, *J* = 1.26, 8.06 Hz, 1H), 7.57 (dd, *J* = 7.05, 8.06 Hz, 1H), 7.44 (dd, *J* = 4.15, 8.18 Hz, 1H), 6.84 (d, *J* = 6.80 Hz, 1H), 4.40 (s, 2H), 4.31 (q, *J* = 7.55 Hz, 2H), 3.38–3.44 (m, 4H), 2.84–2.90 (m, 4H), 1.58 (t, *J* = 7.18 Hz, 3H); ^**13**^C NMR (101 MHz, DMSO-d_6_) δ 176.2, 166.0, 152.9, 152.6 (d, *J* = 250.05 Hz), 150.8, 148.8, 146.2, 143.8, 137.1, 137.0, 133.7, 130.4, 128.1, 126.4, 122.2, 119.9–120.0 (m), 111.5 (d, *J* = 23.27 Hz), 107.2, 106.4–106.5 (m), 54.2, 50.8, 49.1, 46.1–46.2 (m), 14.4; **LC-MS retention time** 2.85 min (method A) and 5.48 min (method B), purity ≥ 98% (both), found 461.0 [M + H]^+^ (both), calculated for C_26_H_25_FN_4_O_3_ 461.2 [M + H]^+^; **HRM**S observed 461.1973 [M + H]^+^, theoretical value 461.1983 [M + H]^+^.

##### 1-Cyclopropyl-6-fluoro-4-oxo-7-(4-((5,6,7,8-tetrahydronaphthalen-1-yl)­methyl)­piperazin-1-yl)-1,4-dihydroquinoline-3-carboxylic acid (**29**)

General procedures 1 and 2 afforded pure **29** and HCl salt **29 salt**, light brown solid, 96.5 mg (70.8% yield); ^**1**^ **H NMR (400 MHz, CDCl**_**3**_ **)** δ 15.06 (br. s., 1H), 8.75 (s, 1H), 7.98 (d, *J* = 13.09 Hz, 1H), 7.34 (d, *J* = 6.80 Hz, 1H), 7.12 (dd, *J* = 1.51, 7.30 Hz, 1H), 7.08 (t, *J* = 7.30 Hz, 1H), 7.03 (dd, *J* = 1.51, 7.30 Hz, 1H), 3.47–3.59 (m, 3H), 3.31–3.37 (m, 4H), 2.79–2.88 (m, 4H), 2.66–2.71 (m, 4H), 1.76–1.88 (m, 4H), 1.34–1.41 (m, 2H), 1.20 (m, 2H); ^**13**^ **C NMR (101 MHz, CDCl**_**3**_) δ 177.0 (d, *J* = 2.48 Hz), 166.9, 153.6 (d, *J* = 251.58 Hz), 147.3, 145.8–145.9 (m), 139.0, 137.6, 136.6, 128.6–128.8 (m), 127.4–127.6 (m), 124.9, 119.5 (d, *J* = 7.25 Hz), 112.2 (d, *J* = 23.46 Hz), 107.9, 104.7–104.8 (m), 60.3–60.4 (m), 52.7, 49.7–49.8 (m), 35.3, 30.1, 25.9, 23.2, 22.8, 8.2; **LC-MS retention time** 3.17 min (method A) and 6.07 min (method B), purity ≥ 98% (both), found 476.1 [M + H]^+^ (both), calculated for C_28_H_30_FN_3_O_3_ 476.2 [M + H]^+^; **HRMS** observed 476.2334 [M + H]^+^, theoretical value 476.2344 [M + H]^+^.

##### 1-Ethyl-6-fluoro-4-oxo-7-(4-((5,6,7,8-tetrahydronaphthalen-1-yl)­methyl) piperazin-1-yl)-1,4-dihydroquinoline-3-carboxylic acid (**30**)

General procedures 1 and 2, starting from norfloxacin (**2**), afforded pure **30** and HCl salt **30 salt**, light brown solid, 59.7 mg (43.2% yield); ^**1**^ **H NMR (400 MHz, CDCl**_**3**_) δ 15.15 (br. s., 1H), 8.67 (s, 1H), 8.04 (d, *J* = 13.09 Hz, 1H), 7.12 (dd, *J* = 1.76, 7.30 Hz, 1H), 7.08 (t, *J* = 7.30 Hz, 1H), 7.03 (dd, *J* = 1.76, 7.30 Hz, 1H), 6.83 (d, *J* = 6.29 Hz, 1H), 4.25–4.36 (m, 2H), 3.52 (s, 2H), 3.28–3.35 (m, 4H), 2.78–2.87 (m, 4H), 2.65–2.71 (m, 4H), 1.75–1.87 (m, 4H), 1.58 (t, *J* = 6.55 Hz, 3H); ^**13**^ **C NMR (101 MHz, CDCl**_**3**_ **)** δ 176.8 (d, *J* = 2.67 Hz), 167.1, 153.5 (d, *J* = 251.77 Hz), 147.0, 146.1 (d, *J* = 11.06 Hz), 137.6, 137.1, 136.6, 128.6, 127.4, 124.8, 120.2 (d, *J* = 7.82 Hz), 112.5 (d, *J* = 23.27 Hz), 108.1, 103.7–103.8 (m), 60.3, 52.6, 49.8–49.9 (m), 49.7, 30.1, 25.8, 23.2, 22.8, 14.4; **LC-MS retention time** 3.18 min (method A) and 6.00 min (method B), purity ≥ 98% (both), found 464.1 [M + H]^+^ (method A) and 464.0 [M + H]^+^ (method B), calculated for C_27_H_30_FN_3_O_3_ 464.2 [M + H]^+^; **HRMS** observed 464.2333 [M + H]^+^, theoretical value 464.2344 [M + H]^+^.

##### 1-Cyclopropyl-7-(4-(4-(dimethylamino)­benzyl)­piperazin-1-yl)-6-fluoro-4-oxo-1,4-dihydroquinoline-3-carboxylic acid (**31**)

General procedures 1 and 2 afforded pure **31** and HCl salt **31 salt**, off-white solid, 67.3 mg (50.5% yield); ^**1**^ **H NMR (400 MHz, CDCl**_**3**_ **)** δ 15.07 (br. s., 1H), 8.77 (s, 1H), 8.02 (d, *J* = 13.09 Hz, 1H), 7.35 (d, *J* = 7.30 Hz, 1H), 7.19–7.23 (m, *J* = 8.56 Hz, 2H), 6.70–6.75 (m, *J* = 8.56 Hz, 2H), 3.52 (s, 3H), 3.31–3.37 (m, 4H), 2.96 (s, 6H), 2.64–2.70 (m, 4H), 1.34–1.40 (m, 2H), 1.16–1.22 (m, 2H); ^**13**^ **C NMR (101 MHz, CDCl**_**3**_ **)** δ 177.1 (d, *J* = 2.93 Hz), 167.1, 153.7 (d, *J* = 251.62 Hz), 150.0, 147.4, 146.1 (d, *J* = 10.27 Hz), 139.1, 130.2, 125.0, 119.7 (d, *J* = 8.07 Hz), 112.4 (d, *J* = 23.48 Hz), 112.4, 108.1, 104.7 (d, *J* = 2.93 Hz), 62.4, 52.5, 49.9 (d, *J* = 5.14 Hz), 40.7, 35.2, 8.2; **LC-MS retention time** 5.48 min (method B), purity ≥ 98%, found 465.0 [M + H]^+^, calculated for C_26_H_29_FN_4_O_3_ 465.2 [M + H]^+^; **HRMS** observed 465.2285 [M + H]^+^, theoretical value 465.2296 [M + H]^+^.

##### 7-(4-(4-(Dimethylamino)­benzyl)­piperazin-1-yl)-1-ethyl-6-fluoro-4-oxo-1,4-dihydroquinoline-3-carboxylic acid (**32**)

General procedures 1 and 2, starting from norfloxacin (**2**), afforded pure **32** and HCl salt **32 salt**, yellow solid, 75.0 mg (55.7% yield); ^**1**^ **H NMR (400 MHz, CDCl**_**3**_ **)** δ 15.14 (br. s., 1H), 8.67 (s, 1H), 8.05 (d, *J* = 13.09 Hz, 1H), 7.21 (d, *J* = 8.56 Hz, 2H), 6.82 (d, *J* = 7.05 Hz, 1H), 6.72 (d, *J* = 8.69 Hz, 2H), 4.31 (q, *J* = 6.88 Hz, 2H), 3.52 (s, 2H), 3.29–3.37 (m, 4H), 2.96 (s, 6H), 2.63–2.70 (m, 4H), 1.57 (t, *J* = 6.80 Hz, 3H); ^**13**^ **C NMR (101 MHz, CDCl**_**3**_ **)** δ 177.0 (d, *J* = 2.93 Hz), 167.3, 153.5 (d, *J* = 251.62 Hz), 150.0, 147.0, 146.3 (d, *J* = 11.00 Hz), 137.1, 130.2, 125.0, 120.4 (d, *J* = 8.07 Hz), 112.7 (d, *J* = 23.48 Hz), 112.4, 108.3, 103.6 (d, *J* = 2.93 Hz), 62.4, 52.4, 49.9 (d, *J* = 5.13 Hz), 49.7, 40.7, 14.4; **LC-MS retention time** 5.43 min (method B), purity ≥ 98%, found 453.1 [M + H]^+^, calculated for C_25_H_29_FN_4_O_3_ 453.2 [M + H]^+^; **HRMS** observed 453.2286 [M + H]^+^, theoretical value 453.2296 [M + H]^+^.

##### 7-(4-(Benzo­[*d*]­[1,3]­dioxol-4-ylmethyl)­piperazin-1-yl)-1-cyclopropyl-6-fluoro-4-oxo-1,4-dihydroquinoline-3-carboxylic acid (**33**)

The reaction afforded pure **33** and HCl salt **33 salt**, off-white solid, 47.8 mg (67.6% yield); ^**1**^ **H NMR (400 MHz, CDCl**_**3**_) δ 15.04 (br. s., 1H), 8.76 (s, 1H), 8.01 (d, *J* = 13.09 Hz, 1H), 7.35 (d, *J* = 7.05 Hz, 1H), 6.86 (dd, *J* = 2.01, 7.55 Hz, 1H), 6.83 (t, *J* = 7.30 Hz, 1H), 6.78 (dd, *J* = 2.01, 7.30 Hz, 1H), 5.98 (s, 2H), 3.63 (s, 2H), 3.53 (br. s., 1H), 3.33–3.40 (m, 4H), 2.69–2.76 (m, 4H), 1.38 (m, 2H), 1.19 (br. s., 2H); ^**13**^ **C NMR (100 MHz, CDCl**_**3**_) δ 177.1 (d, *J* = 2.93 Hz), 167.1, 153.7 (d, *J* = 251.62 Hz), 147.4, 147.2, 146.3, 146.0 (d, *J* = 10.27 Hz), 139.0, 123.4, 121.4, 119.7 (d, *J* = 8.07 Hz), 118.6, 112.4 (d, *J* = 24.21 Hz), 108.1, 107.7, 104.7–104.8 (m), 100.7, 56.2, 52.5, 49.8 (d, *J* = 5.14 Hz), 35.2, 8.2; **LC-MS retention time** 2.87 min (method A) and 5.48 min (method B), purity ≥ 98% (both), found 466.0 [M + H]^+^ (method A) and 466.1 [M + H]^+^ (method B), calculated for C_25_H_24_FN_3_O_5_ 466.2 [M + H]^+^; **HRMS** observed 466.1762 [M + H]^+^, theoretical value 466.1773 [M + H]^+^.

##### 7-(4-(Benzo­[*d*]­[1,3]­dioxol-4-ylmethyl)­piperazin-1-yl)-1-ethyl-6-fluoro-4-oxo-1,4-dihydroquinoline-3-carboxylic acid (**34**)

General procedures 1 and 2, starting from norfloxacin (**2**), afforded pure **34** and HCl salt **34 salt**, white solid, 179 mg (84.3% yield); ^**1**^ **H NMR (400 MHz, CDCl**_**3**_ **)** δ 15.13 (br. s., 1H), 8.66 (s, 1H), 8.03 (d, *J* = 13.35 Hz, 1H), 6.85 (dd, *J* = 2.01, 7.55 Hz, 1H), 6.79–6.84 (m, 2H), 6.78 (dd, *J* = 2.01, 7.30 Hz, 1H), 5.98 (s, 2H), 4.27–4.35 (m, 2H), 3.63 (s, 2H), 3.32–3.38 (m, 4H), 2.69–2.75 (m, 4H), 1.57 (t, *J* = 6.80 Hz, 3H); ^**13**^C NMR (101 MHz, DMSO-d_6_) δ 176.2 (d, *J* = 2.48 Hz), 166.0, 152.7 (d, *J* = 249.29 Hz), 148.8, 147.3, 146.5, 142.5, 137.1, 125.0 (d, *J* = 6.10 Hz), 121.9, 119.9–120.0 (m), 111.5 (d, *J* = 22.32 Hz), 109.9, 107.2, 106.5 (d, *J* = 2.86 Hz), 101.4, 50.1–50.2 (m), 49.1, 46.3 (d, *J* = 4.20 Hz), 14.4; **LC-MS retention time** 2.87 min (method A) and 5.48 min (method B), purity ≥ 98% (both), found 454.1 [M + H]^+^ (method A) and 454.0 [M + H]^+^ (method B), calculated for C_24_H_24_FN_3_O_5_ 454.2 [M + H]^+^; **HRMS** observed 454.1763 [M + H]^+^, theoretical value 454.1773 [M + H]^+^.

##### 1-Cyclopropyl-6-fluoro-7-(4-((4-fluoronaphthalen-1-yl)­methyl)­piperazin-1-yl)-4-oxo-1,4-dihydroquinoline-3-carboxylic acid (**35**)

General procedures 1 and 2 afforded pure **35** and HCl salt **35 salt**, pale yellow solid, 74.7 mg (53.2% yield); ^**1**^ **H NMR (400 MHz, CDCl**_**3**_ **)** δ 15.01 (br. s., 1H), 8.65 (s, 1H), 8.32 (d, *J* = 7.24 Hz, 1H), 8.13 (d, *J* = 8.99 Hz, 1H), 7.87 (d, *J* = 13.11 Hz, 1H), 7.53–7.68 (m, 2H), 7.39 (br. s., 1H), 7.30 (d, *J* = 6.24 Hz, 1H), 7.08 (dd, *J* = 8.12, 9.86 Hz, 1H), 3.97 (br. s., 2H), 3.50 (br. s., 1H), 3.35 (br. s., 4H), 2.75 (br. s., 4H), 1.34 (d, *J* = 4.77 Hz, 2H), 1.16 (br. s., 2H); ^**13**^ **C NMR (101 MHz, CDCl**_**3**_ **)** δ 176.8 (d, *J* = 2.48 Hz), 166.8, 158.6 (d, *J* = 250.44 Hz), 153.4 (d, *J* = 251.58 Hz), 147.2, 145.7–145.9 (m), 138.9, 133.6 (d, *J* = 4.39 Hz), 129.2–129.4 (m), 127.3–127.4 (m), 126.8, 126.0, 124.7, 124.0 (d, *J* = 16.02 Hz), 120.9 (d, *J* = 5.53 Hz), 119.4 (d, *J* = 7.44 Hz), 112.0 (d, *J* = 23.46 Hz), 108.4 (d, *J* = 20.04 Hz), 107.8, 104.7 (d, *J* = 3.05 Hz), 60.6–60.7 (m), 52.6, 49.6–49.8 (m), 35.2, 8.1; **LC-MS retention time** 3.18 min (method A) and 6.08 min (method B), purity ≥ 98% (both), found 490.0 [M + H]^+^ (method A) and 490.1 [M + H]^+^ (method B), calculated for C_28_H_25_F_2_N_3_O_3_ 490.2 [M + H]^+^; **HRMS** observed 490.1929 [M + H]^+^, theoretical value 490.1937 [M + H]^+^.

##### 1-Ethyl-6-fluoro-7-(4-((4-fluoronaphthalen-1-yl)­methyl)­piperazin-1-yl)-4-oxo-1,4-dihydroquinoline-3-carboxylic acid (**36**)

General procedures 1 and 2, starting from norfloxacin (**2**), afforded pure **36** and HCl salt **36 salt**, white solid, 64.9 mg (45.7% yield); ^**1**^ **H NMR (400 MHz, CDCl**_**3**_ **)** δ 15.13 (s, 1H), 8.65 (s, 1H), 8.31–8.35 (m, 1H), 8.12–8.17 (m, 1H), 8.04 (d, *J* = 13.09 Hz, 1H), 7.55–7.62 (m, 2H), 7.38 (dd, *J* = 5.41, 7.68 Hz, 1H), 7.09 (dd, *J* = 7.81, 10.32 Hz, 1H), 6.80 (d, *J* = 7.05 Hz, 1H), 4.28 (q, *J* = 7.05 Hz, 2H), 3.97 (s, 2H), 3.29–3.34 (m, 4H), 2.71–2.76 (m, 4H), 1.55 (t, *J* = 7.05 Hz, 3H); ^**13**^C NMR (101 MHz, DMSO-d_6_) δ 176.2 (d, *J* = 2.48 Hz), 166.0, 148.8, 143.8–143.9 (m), 137.1, 133.7–133.8 (m), 132.2–132.3 (m), 128.2, 127.1, 124.6, 123.1 (d, *J* = 15.83 Hz), 122.1–122.2 (m), 120.5–120.6 (m), 119.9, 111.5 (d, *J* = 22.70 Hz), 109.4 (d, *J* = 19.07 Hz), 107.2, 106.4, 54.6, 50.4–50.5 (m), 49.1, 46.3–46.4 (m), 14.4; **LC-MS retention time** 3.13 min (method A) and 6.07 min (method B), purity ≥ 98% (both), found 478.0 [M + H]^+^ (method A) and 478.1 [M + H]^+^ (method B), calculated for C_27_H_25_F_2_N_3_O_3_ 478.2 [M + H]^+^; **HRMS** observed 478.1925 [M + H]^+^, theoretical value 478.1937 [M + H]^+^.

##### 7-(4-(Benzo­[*b*]­thiophen-7-ylmethyl)­piperazin-1-yl)-1-cyclopropyl-6-fluoro-4-oxo-1,4-dihydroquinoline-3-carboxylic acid (**37**)

The reaction afforded pure **37** and HCl salt **37 salt**, off-white solid, 34.7 mg (32.9% yield); ^**1**^ **H NMR (400 MHz, CDCl**_**3**_) δ 15.09 (br. s., 1H), 8.78 (s, 1H), 8.03 (d, *J* = 13.09 Hz, 1H), 7.79 (d, *J* = 7.81 Hz, 1H), 7.47 (d, *J* = 5.29 Hz, 1H), 7.34–7.41 (m, 3H), 7.27–7.31 (m, 1H), 3.89 (s, 2H), 3.48–3.56 (m, 1H), 3.36–3.42 (m, 4H), 2.73–2.79 (m, 4H), 1.37 (q, *J* = 6.71 Hz, 2H), 1.17–1.23 (m, 2H); ^**13**^C NMR (100 MHz, DMSO-d**_6_**) δ 176.4 (d, *J* = 2.29 Hz), 165.8, 152.6 (d, *J* = 247.00 Hz), 148.2–148.3 (m), 140.4–140.5 (m), 139.1, 127.8–127.9 (m), 124.7–124.9 (m), 119.3, 111.2 (d, *J* = 22.89 Hz), 106.8, 57.8–58.0 (m), 50.7, 46.2–46.3 (m), 35.9, 7.6; **LC-MS retention time** 3.15 min (method A) and 6.12 min (method B), purity ≥ 98% (both), found 477.9 [M + H]^+^ (method A) and 478.0 [M + H]^+^ (method B), calculated for C_26_H_24_FN_3_O_3_S 478.2 [M + H]^+^; **HRMS** observed 478.1584 [M + H]^+^, theoretical value 478.1595 [M + H]^+^.

##### 7-(4-(Benzo­[*b*]­thiophen-7-ylmethyl)­piperazin-1-yl)-1-ethyl-6-fluoro-4-oxo-1,4-dihydroquinoline-3-carboxylic acid (**38**)

General procedures 1 and 2, starting from **norfloxacin (2)** was used to afford pure **38** and HCl salt **38 salt**, yellow solid, 84.5 mg (35.1% yield); ^**1**^ **H NMR (400 MHz, CDCl**_**3**_ **)** δ 15.09 (br. s., 1H), 8.66 (s, 1H), 8.02 (d, *J* = 13.02 Hz, 1H), 7.81 (d, *J* = 8.07 Hz, 1H), 7.47 (d, *J* = 5.41 Hz, 1H), 7.35–7.42 (m, 3H), 6.85 (d, *J* = 6.42 Hz, 1H), 4.31 (q, *J* = 6.94 Hz, 2H), 3.98 (br. s., 2H), 3.44 (br. s., 4H), 2.85 (br. s., 4H), 1.57 (t, *J* = 7.11 Hz, 3H); ^**13**^ **C NMR (100 MHz, CDCl**_**3**_ **)** δ 177.0 (d, *J* = 2.29 Hz), 167.2, 153.5 (d, *J* = 250.00 Hz), 147.1, 140.4, 137.1, 127.2, 124.5, 124.0, 123.4, 120.7, 112.7 (d, *J* = 22.89 Hz), 108.4, 104.0, 61.6–61.7 (m), 52.4, 49.8, 49.7–49.8 (m), 14.5; **LC-MS retention time** 3.15 min (method A) and 6.03 min (method B), purity ≥ 98% (both), found 466.0 [M + H]^+^ (both), calculated for C_25_H_24_FN_3_O_3_S 466.2 [M + H]^+^; **HRMS** observed 466.1584 [M + H]^+^, theoretical value 466.1595 [M + H]^+^.

##### 7-(4-([1,1’-Biphenyl]-4-ylmethyl)­piperazin-1-yl)-1-cyclopropyl-6-fluoro-4-oxo-1,4-dihydroquinoline-3-carboxylic acid (**39**)

General procedures 1 and 2 afforded pure **39** and HCl salt **39 salt**, pale yellow solid, 107 mg (75.1% yield); ^**1**^ **H NMR (400 MHz, CDCl**_**3**_ **)** δ 15.00 (br. s., 1H), 8.75 (s, 1H), 8.00 (d, *J* = 13.09 Hz, 1H), 7.56–7.63 (m, 4H), 7.41–7.48 (m, 4H), 7.33–7.39 (m, 2H), 3.66 (s, 2H), 3.53 (tt, *J* = 3.75, 7.08 Hz, 1H), 3.35–3.41 (m, 4H), 2.70–2.76 (m, 4H), 1.35–1.41 (m, 2H), 1.17–1.22 (m, 2H); ^**13**^ **C NMR (100 MHz, CDCl**_**3**_ **)** δ 177.1 (d, *J* = 2.20 Hz), 167.1, 153.7 (d, *J* = 251.62 Hz), 147.4, 146.0 (d, *J* = 10.27 Hz), 140.8, 140.3, 139.1, 136.7, 129.6, 128.8, 127.3, 127.1, 127.0, 119.7 (d, *J* = 8.07 Hz), 112.3 (d, *J* = 23.48 Hz), 108.1, 104.7 (d, *J* = 3.67 Hz), 62.6, 52.7, 49.8 (d, *J* = 5.13 Hz), 35.2, 8.2; **LC-MS retention time** 3.22 min (method A) and 6.32 min (method B), purity ≥ 98% (both), found 498.1 [M + H]^+^ (both), calculated for C_30_H_28_FN_3_O_3_ 498.2 [M + H]^+^; **HRMS** observed 498.2186 [M + H]^+^, theoretical value 498.2187 [M + H]^+^.

##### 7-(4-([1,1’-Biphenyl]-4-ylmethyl)­piperazin-1-yl)-1-ethyl-6-fluoro-4-oxo-1,4-dihydroquinoline-3-carboxylic acid (**40**)

General procedures 1 and 2 starting from **norfloxacin (2)** was used to afford pure **40** and HCl salt **40 salt**, pale yellow solid, 124 mg (85.7% yield); ^**1**^ **H NMR (400 MHz, CDCl**_**3**_ **)** δ 15.12 (br. s., 1H), 8.66 (s, 1H), 8.04 (d, *J* = 13.09 Hz, 1H), 7.56–7.63 (m, 4H), 7.41–7.48 (m, 4H), 7.33–7.39 (m, 1H), 6.83 (d, *J* = 6.29 Hz, 1H), 4.26–4.37 (m, 2H), 3.66 (s, 2H), 3.37 (br. s., 4H), 2.73 (br. s., 4H), 1.58 (t, *J* = 6.17 Hz, 3H); ^**13**^ **C NMR (101 MHz, CDCl**_**3**_ **)** δ 177.0 (d, *J* = 2.93 Hz), 167.2, 153.5 (d, *J* = 252.36 Hz), 147.0, 146.2 (d, *J* = 11.00 Hz), 140.8, 140.3, 137.1 (d, *J* = 1.47 Hz), 136.7, 129.6, 128.8, 127.3, 127.1, 127.0, 120.4 (d, *J* = 8.07 Hz), 112.7 (d, *J* = 22.74 Hz), 108.3, 103.7 (d, *J* = 3.67 Hz), 62.5, 52.6, 49.9 (d, *J* = 5.13 Hz), 49.7, 14.4; **LC-MS retention time** 6.18 min (method B), purity ≥ 98%, found 486.2 [M + H]^+^, calculated for C_29_H_28_FN_3_O_3_ 486.2 [M + H]^+^; **HRMS** observed 486.2184 [M + H]^+^, theoretical value 486.2187 [M + H]^+^.

#### Synthesis of Levofloxacin (**7**)

##### Ethyl (*R*)-3-((1-hydroxypropan-2-yl)­amino)-2-(2,3,4,5-tetrafluorobenzoyl) acrylate (**4**)

Ethyl 2,3,4,5-tetrafluorobenzoylacetate (5.00 g, 18.9 mmol, 1 equiv) was dissolved in triethyl orthoformate (6.30 mL, 37.9 mmol, 2 equiv) and heated at 140 °C for 30 min. Acetic anhydride (5.37 mL, 56.8 mmol, 3 equiv) was then added and the mixture refluxed at 140 °C for another 40 h and monitored by TLC (10% ethyl acetate/90% hexanes). Upon completion, the reaction was cooled to room temperature, dichloromethane (15 mL) was added and the mixture stirred at room temperature for 5 min. Then l-alaninol (3.01 mL, 37.9 mmol, 2 equiv) was added and the reaction was stirred for 48 h at room temperature. Then a further 2 equiv of l-alaninol was added, and the reaction stirred for a further 48 h. The crude was concentrated *in vacuo* and purified by flash column chromatography (1:1 ethyl acetate/hexanes rising to 3:1 ethyl acetate/hexanes) to give **4**, yellow oil, 5.77 g (87.3% yield); *R*_*f*_ = 0.81 in ethyl acetate; ^**1**^ **H NMR (400 MHz, CDCl**_**3**_) δ 10.93 (br. s., 0.75H, H3), 9.58 (br. s., 0.25H, H3), 8.21 (d, *J* = 14.21 Hz, 1H, H2), 7.10 (br. s., 0.25H, H1), 6.98 (br. s., 0.75H, H1), 3.93–4.19 (m, 2H, H8), 3.73–3.85 (m, 1H, H6), 3.56–3.72 (m, 2H, H4 + 6), 2.44 (br. s., 1H, H7), 1.31–1.42 (m, 3H, H5), 1.10 (t, *J* = 7.06 Hz, 2.25H, H9), 0.98 (t, *J* = 6.24 Hz, 0.75H, H9); ^**13**^ **C NMR (100 MHz, CDCl**_**3**_ **)** δ 186.9 (s, G), 185.1 (s, G), 168.3 (s, I), 166.6 (s, I), 159.9 (s, J), 159.4 (s, J), 127.0–127.3 (m, F), 110.4–110.8 (m, A), 109.8 (d, *J* = 20.03 Hz, A), 101.0 (s, H), 66.1 (s, M), 60.4 (s, N), 60.0 (s, N), 59.7 (s, N), 57.8 (s, K), 57.4 (s, K), 17.0 (s, L), 14.2 (s, O), 14.0 (s, O), 13.6 (s, O); **LC-MS** retention time 3.35 min (method A), purity ≥ 98%, found 350.1 [M + H]^+^, calculated for C_15_H_15_F_4_NO_4_ 350.1 [M + H]^+^. Mass spectrometry data in agreement with published data.

##### Ethyl (*S*)-9,10-difluoro-3-methyl-7-oxo-2,3-dihydro-7H-[1,4]­oxazino­[2,3,4-ij]­quinoline-6-carboxylate (**5**)

**Compound 4** (5.93 g, 17.0 mmol, 1 equiv) was dissolved in dimethylacetamide (40 mL) and stirred for 5 min to dissolve. The solution was then divided evenly into two 20 mL capacity microwave vessels fitted with magnetic stirrer bars and potassium carbonate (7.04 g, 51.0 mmol, 3 equiv) was divided evenly and added to the two vessels. Each microwave vessel was then, in turn, microwaved at 160 °C for 20 min. Upon cooling, the contents of each vessel was added to dichloromethane (200 mL) and washed with distilled water (300 mL). The organic layers were combined and washed two further times with distilled water (2 × 100 mL). The organics were dried over Na_2_SO_4_, decanted and concentrated *in vacuo* to afford crude **5**. This material was used in subsequent reactions without further purification; **5**, off-white solid, 4.65 g (88.5% yield); ^**1**^ **H NMR (400 MHz, CDCl**_**3**_ **)** δ 8.36 (s, 1H, H4), 7.76 (dd, *J* = 8.07, 10.18 Hz, 1H, H1), 4.40–4.52 (m, 3H, H5 + 7), 4.36 (q, *J* = 7.00 Hz, 2H, H8), 1.60 (d, *J* = 6.51 Hz, 3H, H6), 1.39 (t, *J* = 7.02 Hz, 3H, H9); ^**13**^C NMR (100 MHz, DMSO-d_6_) δ 171.1, 164.2, 148.1 (dd, *J* = 11.16, 245.77 Hz), 146.6, 140.9 (dd, *J* = 16.98, 249.86 Hz), 135.2–135.4 (m), 124.4 (d, *J* = 3.24 Hz), 123.7 (d, *J* = 6.10 Hz), 109.8, 103.7 (d, *J* = 19.07 Hz), 68.8, 59.9, 53.8, 17.6, 14.3; ^**19**^ **F­{** ^**1**^ **H} NMR (376 MHz, CDCl**_**3**_) δ −136.4 (d, *J* = 21.46 Hz, 1F), −151.3 (d, *J* = 21.45 Hz, 1F); **LC-MS retention time** 2.85 min (method A), purity = 75%, found 310.1 [M + H]^+^, calculated for C_15_H_13_F_2_NO_4_ 310.1 [M + H]^+^; **[α]**_**D**_^**25.3**^ = −21 deg mL g^–1^ dm^–1^ (c = 0.117 g/100 mL, CH_2_Cl_2_). Characterization data in agreement with published data.^68^

##### (*S*)-9,10-Difluoro-3-methyl-7-oxo-2,3-dihydro-7H-[1,4]­oxazino­[2,3,4-ij]­quinoline-6-carboxylic acid (**6**)

To **Compound 5** (2.02 g, 6.53 mmol, 1 equiv) was added ethanol (20 mL) and a 15% w/v aqueous solution of sodium hydroxide (20 mL) and the suspension stirred at room temperature for 1 h. The mixture was then acidified to pH 3 using a 1 M hydrochloric acid and a few drops of 37% hydrochloric acid, vacuum filtered and washed with distilled water (3 × 100 mL). Precipitate was collected and dried *in vacuo* to afford the crude **6**. This material was used in subsequent reactions without further purification; **6**, off-white solid, 1.60 g (87.2% yield); ^**1**^H NMR (400 MHz, DMSO-d_6_) δ 14.80 (s, 1H, H8), 9.09 (s, 1H, H4), 7.80 (dd, *J* = 7.79, 10.36 Hz, 1H, H1), 5.02 (q, *J* = 6.48 Hz, 1H, H5), 4.64–4.73 (m, 1H, H7), 4.44–4.55 (m, 1H, H7), 1.47 (d, *J* = 6.79 Hz, 3H, H6); ^**13**^C NMR (100 MHz, DMSO-d_6_) δ 176.4 (d, *J* = 2.48 Hz, G), 165.6 (s, I), 148.9 (dd, *J* = 11.44, 248.53 Hz, C), 147.1 (s, J), 141.6 (dd, *J* = 17.36, 252.53 Hz, B), 135.9 (dd, *J* = 3.43, 11.64 Hz, D), 125.3 (d, *J* = 3.82 Hz, E), 121.4 (dd, *J* = 2.29, 7.63 Hz, F), 107.7 (s, H), 103.5 (d, *J* = 19.46 Hz, A), 68.9 (s, M), 55.0 (s, K), 17.8 (s, L); ^**19**^ **F­{** ^**1**^ **H}** **NMR (376 MHz, DMSO-d_6_)** δ −135.9 (d, *J* = 22.47 Hz, 1F, F2), −151.0 (d, *J* = 22.48 Hz, 1F, F3); **LC-MS retention time** 3.11 min (method A), purity = 81%, found 282.1 [M + H]^+^, calculated for C_13_H_9_F_2_NO_4_ 282.1 [M + H]^+^; **[α]**_**D**_^**25.9**^ = −11 deg mL g^–1^ dm^–1^ (c = 0.088 g/100 mL, CH_2_Cl_2_). Characterization data in agreement with published data.^69^

##### (*S*)-9-Fluoro-3-methyl-10-(4-methylpiperazin-1-yl)-7-oxo-2,3-dihydro-7H-[1,4]­oxazino­[2,3,4-ij]­quinoline-6-carboxylic acid (**Levofloxacin**, **7**)

**Compound 6** (100 mg, 0.36 mmol, 1 equiv) and 1-methylpiperazine (118 μL, 1.07 mmol, 3 equiv) were added to a 5 mL capacity microwave vessel fitted with a magnetic stirrer bar and dissolved in dimethyl sulfoxide (2 mL). The mixture was microwaved at 180 °C for 20 min. Upon cooling, the mixture was filtered through a Mini-UniPrep polypropylene filter (0.45 μm pore size) and purified directly using mass-directed preparative HPLC. Pure fractions were collected and freeze-dried overnight to afford pure **7**, light brown solid, 90.5 mg (70.4% yield); ^**1**^H NMR (400 MHz, DMSO-d_6_) δ 15.14 (br. s., 1H, H6), 8.96 (s, 1H, H2), 7.56 (d, J = 11.65 Hz, 1H, H1), 4.93 (br. s., 1H, H3), 4.51–4.69 (m, 1H, H5), 4.25–4.50 (m, 1H, H5), 3.39 (br. s., 4H, H7 + 8), 2.73 (br. s., 4H, H9 + 10), 2.43 (br. s., 3H, H11), 1.45 (br. s., 3H, H4); ^**13**^C NMR (101 MHz, DMSO-d_6_) δ 177.2 (d, *J* = 2.86 Hz, G), 167.8 (s, I), 156.3 (d, *J* = 247.19 Hz, B), 146.7 (s, J), 141.1 (d, *J* = 6.87 Hz, D), 132.0 (d, *J* = 14.49 Hz, C), 125.5 (s, E), 120.8 (d, *J* = 8.77 Hz, F), 107.4 (s, H), 104.2 (d, *J* = 23.46 Hz, A), 69.0 (s, M), 56.1 (s, K), 54.9 (s, P + Q), 49.0 (d, *J* = 2.48 Hz, N + O), 44.8 (s, R), 18.6 (s, L); ^**19**^ **F­{** ^**1**^H} NMR (376 MHz, DMSO-d_6_) δ −120.3 (s, 1F); **LC-MS retention time** 1.81 min (method A) and 3.26 min (method B), purity ≥ 98% (both), found 362.1 [M + H]^+^ (both), calculated for C_15_H_13_F_2_NO_4_ 362.1 [M + H]^+^; **HRMS** observed 362.1509 [M + H]^+^, theoretical value 362.1511 [M + H]^+^; **[α]**_**D**_^**25.2**^ = −43 deg mL g^–1^ dm^–1^ (c = 0.092 g/100 mL, MeOH). Characterization data in agreement with published data.^37^

#### Synthesis of Second-Generation ERB-Fluoroquinolones

##### (*S*)-9-Fluoro-3-methyl-10-(4-(naphthalen-1-ylmethyl)­piperazin-1-yl)-7-oxo-2,3-dihydro-7H-[1,4]­oxazino­[2,3,4-ij]­quinoline-6-carboxylic acid (22)

General procedure 3 was used to afford pure **22**, off-white powder, 51.8 mg (29.9% yield); ^1^H NMR (400 MHz, DMSO-d_6_) δ 14.98 (br. s., 1H, H6), 8.94 (s, 1H, H2), 8.32 (d, *J* = 7.89 Hz, 1H, H18), 7.92 (d, *J* = 7.70 Hz, 1H, H15), 7.85 (d, *J* = 6.97 Hz, 1H, H14), 7.37–7.66 (m, 5H, H1 + 12 + 13 + 16 + 17), 4.81–4.99 (m, 1H, H3), 4.56 (d, *J* = 11.37 Hz, 1H, H5), 4.35 (d, *J* = 10.82 Hz, 1H, H5), 3.94 (s, 2H, H11), 3.28 (br. s., 4H, H7 + 8), 2.58 (br. s., 4H, H9 + 10), 1.43 (d, *J* = 6.24 Hz, 3H, H4); ^**13**^C NMR (101 MHz, DMSO-d_6_) δ 176.3 (d, *J* = 3.24 Hz, G), 166.0 (s, I), 155.5 (d, *J* = 246.81 Hz, B), 146.1 (s, J), 140.3 (d, *J* = 7.06 Hz, D), 133.7 (s, S), 133.5 (s, W), 132.1 (s, AB), 132.0 (d, *J* = 14.11 Hz, C), 128.2 (s, X), 127.8 (s, V), 127.5 (s, T), 125.8 (s, Z), 125.7 (s, Y), 125.2 (s, U), 124.8 (s, AA), 124.8 (s, E), 119.7 (d, *J* = 9.54 Hz, F), 106.6 (s, H), 103.2 (d, *J* = 24.03 Hz, A), 68.0 (s, M), 60.5 (s, R), 54.8 (s, K), 53.5 (s, P + Q), 50.3 (d, *J* = 3.62 Hz, N + O), 17.9 (s, L); **LC-MS** retention time 3.00 min (method A) and 5.92 min (method B), purity ≥ 98% (both), found 488.1 [M + H]^+^ (both), calculated for C_28_H_26_FN_3_O_4_ 488.2 [M + H]^+^; **HRMS** observed 488.1977 [M + H]^+^, theoretical value 488.1980 [M + H]^+^.

##### (*S*)-9-Fluoro-3-methyl-7-oxo-10-(4-(pyrimidin-4-yl)­piperazin-1-yl)-2,3-dihydro-7H-[1,4]­oxazino­[2,3,4-ij]­quinoline-6-carboxylic acid (41)

**Compound 6** (1.37 g, 4.87 mmol, 1 equiv) was dissolved in N,N-dimethylformamide (20 mL) with 4-(piperazin-1-yl)­pyrimidine (1.00 g, 6.09 mmol, 1.25 equiv) and stirred at 140 °C for 90 h. Upon cooling, the mixture was added to 50 mL dichloromethane and washed with 100 mL brine (back extracted with 50 mL more dichloromethane) and 100 mL distilled water. Organic layers were combined, dried over MgSO_4_, filtered and concentrated *in vacuo* to yield the crude product. Purification was achieved via flash column chromatography (100% dichloromethane to 100% acetonitrile to 1% water/acetonitrile; product subsequently eluted with 2 M ammonia in methanol). Pure fractions were concentrated *in vacuo*, redissolved in dichloromethane, filtered and concentrated again to afford **41**, orange solid, 234 mg (18.1% yield); *R*_*f*_ = 0.31 in 5% methanol/dichloromethane; ^**1**^ **H NMR (400 MHz, (CD**_**3**_ **)**_**2**_ **CO)** δ 14.90 (br. s., 1H, H6), 8.78 (s, 1H, H2), 8.52 (s, 1H, H13), 8.21 (d, *J* = 6.11 Hz, 1H, H11), 7.63 (d, *J* = 12.23 Hz, 1H, H1), 6.82 (dd, *J* = 0.92, 6.17 Hz, 1H, H12), 4.95–5.05 (m, 1H, H3), 4.73 (dd, *J* = 1.71, 11.62 Hz, 1H, H5), 4.57 (dd, *J* = 1.71, 11.62 Hz, 1H, H5), 3.78–3.95 (m, 4H, H9 + 10), 3.41–3.55 (m, 4H, H7 + 8), 1.66 (d, *J* = 6.72 Hz, 3H, H4); ^**13**^ **C NMR (101 MHz, (CD**_**3**_ **)**_**2**_ **CO)** δ 178.0 (d, *J* = 2.20 Hz), 166.8, 162.6, 159.2, 157.0 (d, *J* = 246.49 Hz), 156.7, 146.9, 141.9 (d, *J* = 6.60 Hz), 133.0 (d, *J* = 13.94 Hz), 126.2, 122.1 (d, *J* = 8.80 Hz), 108.6, 104.6 (d, *J* = 23.47 Hz), 104.3, 69.4, 56.6, 51.1 (d, *J* = 3.67 Hz), 45.4, 18.4; **LC-MS** retention time 2.70 min (method A) and 5.27 min (method B), purity ≥ 98% (both), found 426.0 [M + H]^+^ (both), calculated for C_21_H_20_FN_5_O_4_ 426.2 [M + H]^+^; **HRMS** observed 426.1568 [M + H]^+^, theoretical value 426.1572 [M + H]^+^.

##### (*S*)-9-Fluoro-3-methyl-7-oxo-10-(4-(pyrimidin-2-yl)­piperazin-1-yl)-2,3-dihydro-7H-[1,4]­oxazino­[2,3,4-ij]­quinoline-6-carboxylic acid (**42**)

General procedure 3: Compound **6** (100 mg, 0.36 mmol, 1 equiv) was dissolved in dimethyl sulfoxide (2 mL) with 2-(piperazin-1- yl)­pyrimidine (146 mg, 0.89 mmol, 2.5 equiv) in a 5 mL capacity microwave vessel fitted with a magnetic stirrer bar and microwaved at 200 °C for 20 min. Upon cooling, the mixture was filtered through a Mini-UniPrep polypropylene filter (0.45 μm pore size). Recrystallization occurred upon leaving overnight; crystals were vacuum filtered and further concentrated *in vacuo* to afford **42**, orange solid, 37.2 mg (24.6% yield); ^**1**^H NMR (400 MHz, DMSO-d**_6_**) δ 15.11 (br. s., 1H, H6), 8.97 (s, 1H, H2), 8.39 (d, *J* = 4.68 Hz, 2H, H11 + 12), 7.58 (d, *J* = 12.20 Hz, 1H, H1), 6.65 (t, *J* = 4.72 Hz, 1H, H13), 4.93 (q, *J* = 6.63 Hz, 1H, H3), 4.56–4.63 (m, 1H, H5), 4.35–4.44 (m, 1H, H5), 3.81–3.95 (m, 4H, H9 + 10), 3.33–3.41 (m, 4H, H7 + 8), 1.46 (d, *J* = 6.69 Hz, 3H, H4); ^**13**^C NMR (101 MHz, DMSO-d**_6_**) δ 176.4 (d, *J* = 3.24 Hz, G), 166.0 (s, I), 161.3 (s, R), 158.0 (s, S+T), 155.5 (d, *J* = 246.81 Hz, B), 146.2 (s, J), 140.5 (d, *J* = 7.06 Hz, D), 131.8 (d, *J* = 14.11 Hz, C), 124.8 (s, E), 120.0 (d, *J* = 9.54 Hz, F), 110.3 (s, U), 106.7 (s, H), 103.3 (d, *J* = 24.03 Hz, A), 68.1 (s, M), 54.8 (s, K), 50.0 (d, *J* = 3.62 Hz, O + N), 44.2 (s, P + Q), 17.9 (s, L); **LC-MS** retention time 3.34 min (method A) and 7.30 min (method B), purity ≥ 98% (both), found 426.1 [M + H]^+^ (both), calculated for C_21_H_20_FN_5_O_4_ 426.2 [M + H]^+^; **HRMS** observed 426.1567 [M + H]^+^, theoretical value 426.1572 [M + H]^+^.

##### (3*S*)-9-Fluoro-3-methyl-7-oxo-10-(3-(pyrimidin-2-ylamino)­pyrrolidin-1-yl)-2,3-dihydro-7H-[1,4]­oxazino­[2,3,4-ij]­quinoline-6-carboxylic acid (**43**)

General procedure 3 starting with **6**, using 160 °C microwave heating, was used to afford crude **43**. The DMSO was evaporated and the compound was crystallized using hot ethanol to afford **43** in in 64% yield; ^**1**^H NMR (400 MHz, DMSO-d**_6_**) δ 15.27 (s, 1H), 8.88 (s, 1H), 8.30 (d, *J* = 4.7 Hz, 2H), 7.53 (d, *J* = 14.1 Hz, 1H), 7.44 (t, *J* = 6.1 Hz, 1H), 6.60 (t, *J* = 4.8 Hz, 1H), 4.87 (d, *J* = 7.1 Hz, 1H), 4.52 (d, *J* = 11.4 Hz, 1H), 4.42 (m, *J* = 5.9 Hz, 1H), 4.28 (d, *J* = 9.1 Hz, 1H), 4.05 – 3.85 (m, 2H), 3.82–3.61 (m, 2H), 2.20–1.94 (m, 2H), 1.45 (d, *J* = 6.7 Hz, 3H); ^13^C NMR (100 MHz, DMSO-d**_6_**) δ 176.0 (d, *J* = 3.24 Hz), 166.2, 162.0, 157.9, 152.7 (d, *J* = 245.86 Hz), 146.0, 135.6 (m), 130.9 (m), 125.0, 115.9 (d, *J* = 8.96 Hz), 110.3, 106.1, 103.7 (d, *J* = 23.84 Hz), 67.7, 56.7 (m), 54.7, 50.5 (m), 49.8 (d, *J* = 8.39 Hz), 30.7, 17.9; **LC-MS** retention time 2.95 min; **HRMS** observed 426.1572 [M + H]^+^, theoretical value 426.1570 [M + H]^+^.

##### (*S*)-9-Fluoro-3-methyl-7-oxo-10-((*S*)-3-(pyrimidin-2-ylamino)­pyrrolidin-1-yl)-2,3-dihydro-7H-[1,4]­oxazino­[2,3,4-ij]­quinoline-6-carboxylic acid (**44**)

General procedure 3 starting with **6**, using 160 °C microwave heating, was used to afford crude **44**. The DMSO was evaporated and the compound was crystallized using hot ethanol to afford **44** in 67% yield; ^1^H NMR (400 MHz, DMSO-d_6_) δ 8.89 (s, 1H), 8.33 (d, J = 4.65 Hz, 2H), 7.54 (d, J = 14.43 Hz, 1H), 6.65–6.63 (m, 1H), 4.87 (d, J = 6.36 Hz, 1H), 4.52 (d, J = 10.76 Hz, 1H), 4.43 (br. s., 1H), 4.28 (d, J = 10.03 Hz, 1H), 4.04–3.90 (m, 2H), 3.77–3.63 (m, 3H), 2.20–1.96 (m, 2H), 1.45 (d, J = 6.60 Hz, 3H); ^13^C NMR (101 MHz, DMSO-d_6_) δ 176.0 (d, *J* = 3.43 Hz), 166.3, 161.6, 157.9, 152.7 (d, *J* = 245.48 Hz), 146.0, 135.7 (d, *J* = 8.77 Hz), 131.0 (d, *J* = 13.35 Hz), 125.0, 115.9 (d, *J* = 9.35 Hz), 110.3, 106.1, 103.7 (d, *J* = 24.61 Hz), 67.8, 56.8 (d, *J* = 4.96 Hz), 54.8, 50.6 (m), 49.8 (d, *J* = 8.20 Hz), 30.7, 17.9; **LC-MS** retention time 2.94 min, found 426.1 [M + H]^+^, calculated for C_21_H_20_FN_5_O_4_ 426.2 [M + H]^+^; **HRMS** observed 426.1566 [M + H]^+^, theoretical value 426.1572 [M + H]^+^.

##### (*S*)-10-((*R*)-3-Aminopyrrolidin-1-yl)-9-fluoro-3-methyl-7-oxo-2,3-dihydro-7H-[1,4]­oxazino­[2,3,4-ij]­quinoline-6-carboxylic acid (**45**)

(*S*)-10-((*R*)-3-((tert-butoxycarbonyl)­amino)­pyrrolidin-1-yl)-9-fluoro-3-methyl-7-oxo-2,3-dihydro-7H-[1,4]­oxazino­[2,3,4-ij]­quinoline-6-carboxylic acid (**45a**, 65 mg, 0.145 mmol, 1 equiv) was dissolved in methanol and 2 M HCl in dioxane (2 mL) was added. The reaction was left at room temperature for 3 h. The solvent was evaporated and **45** was isolated as a free base in 85% yield; ^**1**^H NMR (400 MHz, DMSO-d_6_) δ 15.41 (s, 1H), 8.88 (s, 1H), 7.52 (d, *J* = 14.21 Hz, 1H), 7.16 (d, *J* = 5.96 Hz, 1H), 4.86 (q, *J* = 6.72 Hz, 1H), 4.52 (dd, *J* = 1.56, 11.46 Hz, 1H), 4.27 (dd, *J* = 2.15, 11.32 Hz, 1H), 3.98–4.10 (m, 1H), 3.83–3.92 (m, 1H), 3.80 (tdd, *J* = 3.51, 6.75, 10.08 Hz, 1H), 3.64–3.74 (m, 1H), 3.49 (ddd, *J* = 2.43, 5.04, 10.41 Hz, 1H), 1.96–2.10 (m, 1H), 1.82 (qd, *J* = 6.37, 12.36 Hz, 1H), 1.44 (d, *J* = 6.79 Hz, 3H), 1.39 (s, 9H); ^13^C NMR (101 MHz, DMSO-d_6_) δ 176.0 (d, *J* = 3.24 Hz), 166.2, 155.3, 152.7 (d, *J* = 245.86 Hz), 146.0, 135.6 (d, *J* = 8.78 Hz), 130.9 (d, *J* = 13.35 Hz), 125.0, 116.0 (m), 106.1, 103.7 (d, *J* = 24.41 Hz), 77.8, 67.8, 56.7 (m), 54.8, 49.9 (d, *J* = 1.53 Hz), 49.7 (d, *J* = 7.44 Hz), 30.8, 28.2, 17.9. **LC-MS** retention time 1.92 min, purity ≥ 98%, found 348.1 [M – H]^+^, calculated for C_17_H_18_FN_3_O_4_ [M – H]^+^; **HRMS** observed 348.1354 [M + H]^+^, theoretical value 348.1354 [M + H]^+^.

##### (*S*)-9-Fluoro-3-methyl-7-oxo-10-((*R*)-3-(pyrimidin-2-ylamino)­pyrrolidin-1-yl)-2,3-dihydro-7H-[1,4]­oxazino­[2,3,4-ij]­quinoline-6-carboxylic acid (**KSN-L22**, **46**)

General procedure 3 starting with **6**, using 160 °C microwave heating, was used to afford crude **46**. The DMSO was evaporated and the compound was crystallized using hot ethanol to afford **46** in 71% yield; ^**1**^H NMR (400 MHz, DMSO-d_6_) δ 15.41 (br. s., 1H), 8.89 (s, 1H), 8.30 (d, *J* = 4.58 Hz, 2H), 7.54 (d, *J* = 14.12 Hz, 1H), 7.47 (d, *J* = 6.24 Hz, 1H), 6.61 (t, *J* = 4.77 Hz, 1H), 4.87 (d, *J* = 6.42 Hz, 1H), 4.52 (d, *J* = 11.55 Hz, 1H), 4.44–4.40 (m, 1H), 4.28 (d, *J* = 10.82 Hz, 1H), 4.0–3.86 (m, 2H), 3.84–3.61 (m, 2H), 2.19–1.96 (m, 2H), 1.45 (d, *J* = 6.60 Hz, 3H); ^13^C NMR (100 MHz, DMSO-d_6_) δ 176.0 (d, *J* = 3.43 Hz), 166.2, 162.0, 157.9, 152.7 (d, *J* = 245.67 Hz), 146.0, 135.6 (d, *J* = 8.77 Hz), 131.0 (d, *J* = 13.16 Hz), 125.0, 115.9 (d, *J* = 9.35 Hz), 110.3, 106.1, 103.7 (d, *J* = 24.41 Hz), 67.7, 56.7 (d, *J* = 5.72 Hz), 54.8, 50.5 (d, *J* = 0.95 Hz), 49.8 (d, *J* = 7.44 Hz), 30.7, 17.9; **LC-MS** retention time 2.88 min, purity ≥ 98%, found 426.2 [M + H]^+^, calculated for C_21_H_20_FN_5_O_4_ 426.2 [M + H]^+^; **HRMS** observed 426.1568 [M + H]^+^, theoretical value 426.1572 [M + H]^+^.

##### (*S*)-9-Fluoro-10-((*R*)-3-((5-fluoropyrimidin-2-yl)­amino)­pyrrolidin-1-yl)-3-methyl-7-oxo-2,3-dihydro-7H-[1,4]­oxazino­[2,3,4-ij]­quinoline-6-carboxylic acid (**47**)

General procedure 3 starting with **6**, using 160 °C microwave heating, was used to afford crude **47**. The DMSO was evaporated and the compound was crystallized using hot ethanol to afford **47** in 66.2% yield; ^**1**^H NMR (400 MHz, DMSO-d_6_) δ 15.42 (s, 1H), 8.89 (s, 1H), 8.40 (d, *J* = 0.92 Hz, 2H), 7.57 (d, *J* = 6.24 Hz, 1H), 7.54 (d, *J* = 14.12 Hz, 1H), 4.90–4.84 (m, 1H), 4.52 (dd, *J* = 1.47, 11.37 Hz, 1H), 4.40–4.32 (m, 1H), 4.30–4.26 (m, 1H), 4.00–3.86 (m, 2H), 3.82–3.62 (m, 2H), 2.20–1.93 (m, 2H), 1.45 (d, *J* = 6.79 Hz, 3H); ^13^C NMR (100 MHz, DMSO-d_6_) δ 176.0 (d, *J* = 3.24 Hz), 166.2, 159.2, 152.7 (d, *J* = 245.67 Hz), 150.5 (d, *J* = 7.82 Hz), 146.0, 145.5 (m), 135.7 (d, *J* = 8.39 Hz), 130.9 (d, *J* = 12.40 Hz), 125.0, 115.9 (d, *J* = 9.54 Hz), 106.1, 103.7 (d, *J* = 24.60 Hz), 67.7, 56.6 (d, *J* = 6.10 Hz), 54.7, 51.1, 49.7 (d, *J* = 6.48 Hz), 30.7, 17.9; **LC-MS** retention time 3.33 min, purity ≥ 98%,, found 444.1 [M + H]^+^, calculated for C_21_H_19_F_2_N_5_O_4_ 444.1 [M + H]^+^; **HRMS** observed 445.1475 [M + H]^+^, theoretical value 445.1478 [M + H]^+^.

##### (*S*)-9-Fluoro-3-methyl-7-oxo-10-((*R*)-3-(pyrazin-2-ylamino)­pyrrolidin-1-yl)-2,3-dihydro-7H-[1,4]­oxazino­[2,3,4-ij]­quinoline-6-carboxylic acid (**48**)

General procedure 3 starting with **6**, using 160 °C microwave heating, was used to afford crude **48**. The DMSO was evaporated and the compound was crystallized using hot ethanol to afford **48** in 72% yield; ^**1**^ **H NMR (400 MHz, DMSO-d**_6_ **)** δ 8.89 (s, 1H), 7.96 (s, 1H), 7.69 (d, *J* = 2.38 Hz, 1H), 7.54 (d, *J* = 14.12 Hz, 1H), 7.38 (d, *J* = 6.24 Hz, 1H), 4.89–4.84 (m, 1H), 4.56–4.48 (m, 1H), 4.45–4.35 (m, 1H), 4.32–4.24 (m, 1H), 4.07–3.99 (m, 1H), 3.95–3.75 (m, 3H), 3.65–3.57 (m, 1H), 2.27–2.16 (m, 1H), 1.98–1.87 (m, 1H), 1.45 (d, *J* = 6.79 Hz, 3H); ^**13**^C NMR (100 MHz, DMSO-d_6_) δ 176.1 (d, *J* = 3.43 Hz), 166.3, 154.7, 152.8 (d, *J* = 245.48 Hz), 146.1, 141.5, 135.7 (d, *J* = 8.77 Hz), 133.6, 131.3, 131.0 (d, *J* = 13.35 Hz), 125.1, 116.1 (d, *J* = 9.16 Hz), 106.2, 103.8 (d, *J* = 24.41 Hz), 67.8, 56.8 (d, *J* = 5.53 Hz), 54.8, 50.0 (d, *J* = 1.34 Hz), 49.8 (d, *J* = 7.63 Hz), 31.0, 18.0; **LC-MS** retention time 3.00 min; purity ≥ 98%, **HRMS** observed 426.1575 [M + H]^+^, theoretical value 426.1572 [M + H]^+^.

##### (*S*)-9-Fluoro-3-methyl-10-((*R*)-3-((6-methylpyrimidin-4-yl)­amino)­pyrrolidin-1-yl)-7-oxo-2,3-dihydro-7H-[1,4]­oxazino­[2,3,4-ij]­quinoline-6-carboxylic acid (**49**)

General procedure 3 starting with **6**, using 160 °C microwave heating, was used to afford crude **49**. The DMSO was evaporated and the compound was crystallized using hot ethanol to afford **49** in 69% yield; ^1^H NMR (400 MHz, DMSO-d_6_) δ 8.90 (s, 1H), 8.33 (s, 1H), 7.57–7.50 (m, 2H), 6.36 (br. s., 1H), 4.88 (q, *J* = 6.91 Hz, 1H), 4.54–4.47 (m, 2H), 4.32–4.25 (m, 1H), 4.04–3.97 (m, 1H), 3.91–3.76 (m, 2H), 3.59 (dd, *J* = 2.57, 5.50 Hz, 1H), 2.24–2.15 (m, 4H), 1.91 (dd, *J* = 6.05, 11.92 Hz, 1H), 1.45 (d, *J* = 6.60 Hz, 3H); ^13^C NMR (100 MHz, DMSO-d_6_) δ 176.0 (d, *J* = 3.05 Hz), 166.2, 162.0, 157.7, 152.8 (d, *J* = 245.67 Hz), 146.0, 135.7 (d, *J* = 8.58 Hz), 130.8 (d, *J* = 13.16 Hz), 125.0, 116.1 (d, *J* = 9.16 Hz), 106.1, 103.7 (d, *J* = 24.60 Hz), 67.8, 56.7 (d, *J* = 4.77 Hz), 54.8, 49.8 (m), 30.9, 23.3, 18.0; **LC-MS** retention time 2.26 min, purity ≥ 98%, found 440.1 [M + H]^+^, calculated for C_22_H_22_FN_5_O_4_ 440.2 [M + H]^+^; **HRMS** observed 440.1759 [M + H]^+^, theoretical value 440.1729 [M + H]^+^.

##### (*S*)-10-((*R*)-3-((4,6-Dimethylpyrimidin-2-yl)­amino)­pyrrolidin-1-yl)-9-fluoro-3-methyl-7-oxo-2,3-dihydro-7H-[1,4]­oxazino­[2,3,4-ij]­quinoline-6-carboxylic acid (**BL-7**, **50**)

General procedure 3 starting with **6**, using 160 °C microwave heating, was used to afford crude **50**. The DMSO was evaporated and the compound was crystallized using hot ethanol to afford **50** in 74% yield; ^1^H NMR (400 MHz, DMSO-d_6_) δ 15.43 (s, 1H), 8.89 (s, 1H), 7.54 (d, *J* = 14.12 Hz, 1H), 7.25 (d, *J* = 6.60 Hz, 1H), 6.39 (s, 1H), 4.87 (d, *J* = 6.97 Hz, 1H), 4.52 (d, *J* = 10.27 Hz, 1H), 4.49–4.41 (m, 1H), 4.28 (d, *J* = 9.72 Hz, 1H), 3.99–3.92 (m, 1H), 3.87–3.80 (m, 2H), 3.64–3.56 (m, 1H), 2.23–2.18 (m, 6H), 2.18–2.09 (m, 1H), 2.02–1.90 (m, 1H), 1.45 (d, *J* = 6.60 Hz, 3H); ^13^C NMR (100 MHz, DMSO-d_6_) δ 176.0 (d, *J* = 3.24 Hz), 166.7, 166.3, 161.9, 152.7 (d, *J* = 245.67 Hz), 146.0, 135.7 (d, *J* = 8.77 Hz), 131.0 (d, *J* = 13.16 Hz), 125.0, 115.9 (d, *J* = 9.16 Hz), 108.9, 106.1, 103.7 (d, *J* = 24.41 Hz), 67.8, 56.8 (d, *J* = 5.72 Hz), 54.8, 50.3 (d, *J* = 1.14 Hz), 49.8 (d, *J* = 7.06 Hz), 30.8, 23.5, 17.9; **LC-MS** retention time 2.69 min, purity ≥ 98%, found 454.2 [M + H]^+^, calculated for C_23_H_24_FN_5_O_4_ 454.2 [M + H]^+^ **HRMS** observed 454.1887 [M + H]^+^, theoretical value 454.1885 [M + H]^+^.

##### (*R*)-1-Cyclopropyl-6-fluoro-8-methoxy-4-oxo-7-(3-(pyrimidin-2-ylamino) pyrrolidin-1-yl)-1,4-dihydroquinoline-3-carboxylic acid (**ML-110-014**, **51**)

General procedure 3, starting from 1-cyclopropyl-6,7-difluoro-8-methoxy-4-oxo-1,4-dihydroquinoline-3-carboxylic acid (50.0 mg, 0.17 mmol, 1 equiv) and using microwave heating at 140 °C for 30 min, was used to afford crude **51**. Upon cooling, the mixture was filtered through a Mini-UniPrep polypropylene filter (0.45 μm pore size) and purified directly using mass-directed preparative HPLC. Collated pure fractions were lyophilized to afford **51** as a light brown solid in 35.5% yield (26.4 mg); ^**1**^H NMR (400 MHz, DMSO-d_6_) δ 15.18 (s, 1H, H6), 8.65 (s, 1H, H2), 8.30 (d, *J* = 4.77 Hz, 2H, H13 + 14), 7.66 (d, *J* = 13.94 Hz, 1H, H1), 7.52 (d, *J* = 6.14 Hz, 1H, H12), 6.61 (t, *J* = 4.77 Hz, 1H, H15), 4.47 (sxt, *J* = 5.81 Hz, 1H, H11), 4.09–4.18 (m, 1H, H3), 3.85–3.93 (m, 1H, H9), 3.78–3.85 (m, 1H, H8), 3.64–3.74 (m, 1H, H8), 3.53–3.60 (m, 4H, H7 + 9), 2.21 (dq, *J* = 6.25, 12.70 Hz, 1H, H10), 1.99–2.10 (m, 1H, H10), 1.06–1.13 (m, 2H, H4 + 5), 0.94–1.03 (m, 2H, H4 + 5); ^**13**^C NMR (101 MHz, DMSO-d_6_) δ 176.0 (d, *J* = 3.43 Hz, G), 165.9 (s, I), 162.0 (s, S), 158.0 (s, T+U), 150.2 (s, J), 141.0 (d, *J* = 7.44 Hz, D), 136.9 (d, *J* = 10.68 Hz, C), 134.5 (s, E), 117.1 (d, *J* = 8.96 Hz, F), 110.4 (s, V), 106.4 (d, *J* = 23.65 Hz, A), 106.3 (s, H), 61.3 (s, N), 56.4 (d, *J* = 5.53 Hz, P), 50.7 (s, R), 49.5 (d, *J* = 7.06 Hz, O), 40.7 (s, K), 30.8 (s, Q), 8.9 (d, *J* = 3.43 Hz, L + M); **LC-MS** retention time 2.728 min (method A), purity = 95%, found 440.1 [M + H]^+^, calculated for C_22_H_22_FN_5_O_4_ 440.2 [M + H]^+^; **HRMS** observed 440.1727 [M + H]^+^, theoretical value 440.1729 [M + H]^+^; **[α]**_**D**_^**23.9**^ = −88 deg mL g^–1^ dm^–1^ (c = 0.128 g/100 mL, MeOH).

### General Materials and Methods – Biology

All bacteria were maintained on tryptic soy agar (TSA), except *Streptococcus* spp., which were maintained on Columbia agar plates containing 5% horse blood. All bacterial assays were carried out in tryptic soy broth (TSB), except for *Streptococcus* spp. which were grown in cation-adjusted Mueller Hinton broth containing 2.5% lysed horse blood. Bacteria were grown to stationary phase in liquid culture overnight, with shaking at 200 rpm, before being back diluted to the relevant starting concentration for each assay below. MICs were measured by the microbroth dilution method. Briefly, a 2-fold dilution series of compound was prepared (100 μL/well) in a 96 well plate and incubated with bacteria back-diluted from an overnight culture to a starting concentration of 1 × 105 CFU/mL for 20 h at 37 °C. Absorbance at OD600 was read on a CLARIOstar spectrophotometer (BMG Labtech) and the MIC was defined as the minimum concentration of compound where no visible growth was detected. The compounds were prepared as a DMSO stock and equivalent concentrations of DMSO had no effect on bacterial growth. MICs were also performed in the presence of the outer membrane permeator, polymyxin b nonapeptide (PMBN) following the same MIC method but with the following variation. The 2-fold dilution series of compound was prepared as above, then 50 μL of PMBN at a final concentration of 30 μg/mL was added to relevant wells, while 50 μL/well of blank media was added to the remaining wells. Bacteria were then added at 50 μL/well for a starting concentration as above. Thirty μg/mL of PMBN had no effect on bacterial growth. The MICs were read and defined as above. All animal procedures were performed by the contract research organization Pharmacology Discovery Services Taiwan in accordance with the *Guide for the Care and Use of Laboratory Animals*. The procedures were approved by the Institutional Ethics Committee of Pharmacology Discovery Services Taiwan, Ltd. (approval no. AB127662), in compliance with animal welfare legislation in Taiwan.

### Gyrase Assay

Gyrase inhibition was determined through biochemical assay kits from Inspiralis Ltd. (Norwich, UK), according to manufacturer’s instructions, and as described previously.^70^ Briefly, 1U of enzyme was incubated with 0.5 μg of relaxed pBR233 DNA in the presence of compound in 1% DMSO for 30 min at 37 °C. A control reaction contained no compound but still contained 1% DMSO. The reactions were stopped through Stop Buffer addition and the DNA separated through addition of chloroform/iso-amyl alcohol (24;1). Samples were run on a 1% agarose gel at 75 V for 2 h, which was then stained with 1 μg/mL ethidium bromide and visualized using a NuGenius gel imager (Syngene). The intensity of the supercoiled DNA band was quantified using Syngene software, and the inhibition of the supercoiling of the DNA by the compounds was calculated.

### Checkerboard Assays for Synergy

Checkerboard assays were performed by preparing two separate 2-fold dilution series of compounds in two 96 well plates, one horizontally, one vertically. The two dilutions were then combined into a single plate and bacteria added for a final starting concentration of 1 × 105 CFU/mL. Plates were incubated at 37 C for 20 h and the absorbance (OD600) was read on a CLARIOstar spectrophotometer (BMG Labtech). The fractional inhibitory concentration was calculated as follows: ΣFIC = [(MIC of A in combination)/(MIC of A alone)] + [(MIC of B in combination)/(MIC of B alone)].

### Time Kills

Time-kill assays were performed as defined previously.^70^ Briefly, in glass universals, compound dilutions at 4 × MIC concentration were prepared in 3 mL of broth, alongside an untreated control. Bacteria were added for a starting concentration of 1 × 105 CFU/mL. Universals were incubated at 37 C, with shaking at 200 rpm for 24 h. Samples were taken at 0, 1-, 2-, 4-, 6-, and 24-h time points, and the CFU/mL was calculated using the Miles-Misra technique.

### Passaging for Resistance

In order to examine resistance mechanisms, bacteria were passaged with increasing concentrations of compounds as follows. One × 105 CFU/mL bacteria were incubated in 3 mL of broth with 0.25 x MIC con centration of compound at 37 C, with shaking at 200 rpm. After 24–48 h, 30 μL of this sample was transferred to 3 mL broth containing 0.5 × MIC concentration of compound. This step was re peated at increasing concentrations until either no growth was detected after 48 h incubation, or until 4 x MIC concentration was reached. Bacteria from the liquid passaging were then passaged 10 times on solid agar in the absence of the compound to determine whether stable mutations were likely to be present. MICs were performed on the first and 10th passage.

### Whole Genome Sequencing

Genomic DNA was isolated from stationary phase bacterial liquid cultures using a Wizard Genomic DNA purification kit (Promega, UK) as per manufacturer’s instructions. The isolated gDNA was sequenced by UKHSA-GSDU on an Illumina (HiSeq 2500), as previously described. Potential individual mutations were identified using Galaxy.^71^

### Mutation Frequencies

Mutation frequencies were performed on solid media at supra-inhibitory concentrations. First, the agar MIC was performed to account for any issues in solubility of compounds in solid media. Agar plates were prepared with 2-fold dilutions of compound. One μL spots of bacteria at a concentration of 1 × 105 CFU/mL was dropped onto plates, which were incubated for 24 h at 37 °C, before agar MICs were read by eye.

For the mutation frequency assay, bacteria from an overnight culture were grown to mid exponential phase in liquid broth, then 100 μL was spread onto an agar plate containing compound at concentrations above the agar MIC. A Miles-Misra was also conducted on an antibiotic-free agar plate to determine the CFU/mL of the starting population. Plates were incubated at 37 °C for 24 h, when the agar MIC was checked and then for an additional 24 h, at which point the colonies were counted. The mutation frequency was calculated by dividing the number of colonies on supra inhibitory con-centration plates by the starting population.

### Galleria mellonella Efficacy Study

The nonanimal infection model, *Galleria mellonella* was used as an efficacy model and a toxicity test. Larvae were procured from livefoods.co.uk and stored for up to 2 weeks at 8 °C to maintain the larval state. For toxicity testing, 10 μL compound was injected into 10 healthy larvae at 50 mg/kg through the front proleg. Ten additional larvae were also injected with PBS as a control. Larvae were incubated at 37 °C in the dark and survival was recorded for 5 days. For efficacy testing, 10 larvae per sample were injected with 10 μL bacteria at LD50 doses (NCTC 13616 at 1 × 107 CFU/10 μL and NCTC 12923 at 1 × 106 CFU/10 μL) through the front left proleg. Larvae were left to heal for 30 min at room temperature and then injected through the front right proleg with compound. Larvae were incubated and assessed as stated above.

### Statistics

Statistical analyses were performed with GraphPad Prism V9.5.0. The area under the curve (AUC) of NCTC 13616 growth curves exposed to 0.5x MIC with and without reserpine was determined, and unpaired *t* tests with Welch correction were used to compare the means for each compound of interest. Unpaired *t* tests were used when comparing two samples in the gyrase assays. For the survival curves of the *G. mellonella* experiments, statistical significance was assessed using the Mantel-Cox (log-rank) test.

### Maximum Tolerated Dose (MTD) Study

The maximum tolerated doses of **KSN-L22 (46)** and **KSN-BL-7 (50)** were determined in male ICR mice using an oral dose-escalation protocol conducted by Eurofins Pharmacology Discovery Services (Taipei, Taiwan). Mice (20–30 g) received three oral doses of **50** at 6-h intervals, beginning at 100 mg/kg (10 mL/kg in 5% DMSO/5% Solutol suspension). Dose escalation proceeded to 200 mg/kg and 400 mg/kg as no mortality or clinical signs of distress were observed over 72 h. Animals were monitored for acute toxic signs (mortality, convulsions, tremors, muscle relaxation, sedation) and autonomic effects (diarrhea, salivation, lacrimation, vasodilation, piloerection) at 0–60 min postdose and at 2, 24, 48, and 72 h after the final dose. Body weights were recorded predose and at 72 h, and gross necropsy was performed on all animals without tissue collection

### Pharmacokinetics Study

Pharmacokinetic studies were performed in male ICR (CD-1) mice obtained from Charles River, UK. Animals were housed in sterilized individually ventilated cages with HEPA-filtered air, free access to sterile food and water, 12-h light–dark cycles, and controlled temperature and humidity. Single-dose pharmacokinetic profiles were determined following oral (20 mg/kg) or intravenous (5 mg/kg) administration of each test compound, with **levofloxacin** used as a comparator at identical doses and routes. Blood samples were collected from the caudal vein into anticoagulant-treated capillaries at predetermined time points up to 8 h, with terminal cardiac puncture at 8 h. Plasma samples were stored at −80 °C until analysis. Urine was collected in metabolic cages at timed intervals up to 24 h and stored at −80 °C. A compound-specific LC-MS/MS method was developed for each test article using matrix-matched calibrators following protein precipitation of samples. Concentration–time data were analyzed by noncompartmental methods to determine Cmax, Tmax, half-life, CL, Vss, AUC0–t, AUCinf, oral bioavailability, urinary excretion, and renal clearance, with **levofloxacin** included in all analyses for comparison.

### *In Vivo* Efficacy Study of **KSN-L22** and **BL-7**

Efficacy was evaluated using neutropenic murine thigh infection models performed in two independent studies. Neutropenia was induced by intraperitoneal cyclophosphamide administered 4 days (150 mg/kg) and 1 day (100 mg/kg) before infection. In one study, male ICR mice were infected under isoflurane anesthesia in both lateral thighs with 5 × 10^3^ CFU of *Staphylococcus aureus* ATCC 29213 and given subcutaneous buprenorphine for analgesia. **KSN-L22 (46)** was prepared freshly before dosing and administered orally at 20 or 50 mg/kg at 2, 8, and 14 h postinfection, with **levofloxacin** used as a positive control at identical doses and schedule. Animals were euthanized at 18 h postinfection when vehicle-treated mice reached clinical end points, and thigh tissues were excised, homogenized, and plated on CLED agar for quantitative culture. Bacterial burdens were compared using Kruskal–Wallis tests with Conover–Inman posthoc analysis.

In a separate study, female ICR mice were infected intramuscularly with 1 × 10^5^ CFU (actual 1.68 × 10^5^ CFU/mouse) of methicillin-resistant *S. aureus* ATCC 33591. **BL-7 (50)** and **levofloxacin (7)** were administered orally at 10 or 50 mg/kg three times (2, 8, and 14 h postinfection), and vancomycin was included as an intravenous reference control (30 mg/kg at 2 and 8 h postinfection). Animals were euthanized at 2 h (baseline) or 26 h postinfection, thigh tissues were homogenized, serially diluted, and plated on nutrient agar to determine CFU/g. Statistical significance was assessed using one-way ANOVA with Dunnett’s test, and bactericidal activity was defined as a ≥ 1-log_10_ reduction relative to initial counts.

### Aqueous Solubility Study of **ML-77-005 (8)**, **KSN-L22 (46)**, and **BL-7 (50)**

Kinetic aqueous solubility was determined using a standard LC-UV/MS-based assay. Test compounds were prepared from 10 mM DMSO stock solutions and diluted to a nominal concentration of 200 μM in phosphate-buffered saline (PBS, pH 7.4) containing 2% DMSO. Parallel dilutions in DMSO were used as reference standards. Samples were equilibrated at room temperature for 2 h with shaking, then filtered, and the filtrates were analyzed by LC-UV with mass spectrometric confirmation of the analyte peak. Solubility was determined by comparing the UV peak area of the PBS filtrate with those of the DMSO reference standards. The effective range of the assay was 10–200 μM.

### Plasma Protein Binding Study of **ML-77-005 (8)**, **KSN-L22 (46)**, and **BL-7 (50)**

Plasma protein binding was measured using a Rapid Equilibrium Dialysis (RED) assay. Test compounds and control compounds were added to plasma at 10 μM and dialyzed against phosphate-buffered saline (PBS, pH 7.4) for 4 h at 37 °C. After incubation, samples from the plasma and buffer compartments were collected, treated to ensure comparable sample matrices, and analyzed by LC-MS/MS after protein precipitation with acetonitrile containing an internal standard. The percentage of drug bound to plasma proteins was calculated by comparing the analyte response in the plasma compartment with that in the buffer compartment.

### Cytochrome P450 Interaction Study

Cytochrome P450 inhibition was assessed in vitro using human recombinant enzymes in microsomal preparations. **KSN-L22** **(46)** and **BL-7** **(50)** were incubated with individual CYP isoforms and specific probe substrates under standard assay conditions according to Eurofins validated procedures. Enzyme activity was quantified by monitoring formation of substrate-specific metabolites, and percent inhibition was calculated relative to vehicle control. Results were interpreted using Eurofins assay criteria, with ≥ 50% inhibition considered a significant interaction requiring follow-up concentration–response analysis and IC_50_ determination, and ≤ 25% inhibition regarded as not meaningful.

### TOff-target Screening Using SafetyScreen Pharmacology Panel

Off-target pharmacology was evaluated using the Eurofins SafetyScreen44 in vitro panel. **KSN-L22 (46)** and **BL-7 (50)** were tested at a fixed concentration of 10 μM across a broad range of human molecular targets, including G-protein-coupled receptors, ion channels (including hERG potassium channel), transporters, kinases, and metabolic enzymes. Binding assays were performed using radioligand displacement, and enzyme or uptake assays measured inhibition of control activity using validated detection methods (scintillation counting, fluorimetry or photometry). Responses were expressed as percent inhibition of control binding or activity. Based on Eurofins interpretation guidelines, effects ≥50% were considered significant, effects between 25% and 50% weak to moderate, and effects <25% were not considered biologically relevant.

## Supplementary Material































## Notes

PDB file corresponding to the molecular model shown in Figure 2a (PDB)
PDB file corresponding to the molecular model shown in Figure 2c (PDB)
PDB file corresponding to the molecular model shown in Figure 5a (PDB)
PDB file corresponding to the molecular model shown in Figure 5d (PDB)
PDB file corresponding to the molecular model shown in Figure 5e (PDB)
PDB file corresponding to the molecular model shown in Figure S1c (PDB)
PDB file corresponding to the molecular model shown in Figure S3a (PDB)
PDB file corresponding to the molecular model shown in Figure S3b (PDB)
PDB file corresponding to the molecular model shown in Figure S3c (PDB)
PDB file corresponding to the molecular model shown in Figure S5a (PDB)
PDB file corresponding to the molecular model shown in Figure S5b (PDB)
PDB file corresponding to the molecular model shown in Figure S7a (PDB)
PDB file corresponding to the molecular model shown in Figure S7b (PDB)
Molecular formula strings (CSV)
Gradient conditions for analytical and preparative LC-MS, characterization data (HRMS, NMR, and LC-MS spectra), additional experimental results, supporting figures and tables associated with this study (PDF)

## Abbreviations

AdeABC: Acinetobacter drug efflux pump ABC family
AdeFGH: Acinetobacter drug efflux pump FGH family
AdeIJK: Acinetobacter drug efflux pump IJK family
AMR: antimicrobial resistance
AUC: area under the curve
CDC: Centers for Disease Control and Prevention
CFU: colony forming unit
CIP: ciprofloxacin
CIP-R: ciprofloxacin resistant
Cmax: maximum plasma concentration
COM: center of mass
CYP: cytochrome P450
DIPEA: N,N-diisopropylethylamine
DMAP: 4-(dimethylamino)­pyridine
DMF: N,N-dimethylformamide
DMPK: drug metabolism and pharmacokinetics
DNA: DNA
EDCI: 1-ethyl-3-(3-(dimethylamino)­propyl)­carbodiimide
EPI: efflux pump inhibitor
ERB: efflux resistance breaker
FDA: Food and Drug Administration
GyrA: DNA gyrase subunit A
hERG: human ether-à-go-go–related gene potassium channel
IC_50_: half-maximal inhibitory concentration
LC-MS: liquid chromatography–mass spectrometry
LVX: levofloxacin
MDR: multidrug resistant
MD: molecular dynamics
MFS: major facilitator superfamily
MIC: minimum inhibitory concentration
MIC_50_: minimum inhibitory concentration inhibiting 50% of isolates
MIC_90_: minimum inhibitory concentration inhibiting 90% of isolates
MRSA: methicillin-resistant *Staphylococcus aureus*
MTD: maximum tolerated dose
NMP: 1-(naphthalen-1-ylmethyl)­piperazine
ParC: DNA topoisomerase IV subunit C
PK: pharmacokinetics
PMBN: polymyxin B nonapeptide
PRSP: penicillin-resistant *Streptococcus pneumoniae*
RND: resistance–nodulation–division
SAR: structure–activity relationship
SEM: standard error of the mean
TID: three times daily
TLC: thin-layer chromatography
Tmax: time to maximum plasma concentration
VISA: vancomycin-intermediate *Staphylococcus aureus*
VRE: vancomycin-resistant enterococcus
WHO: World Health Organization
WT: wild type

## References

[1] Nicolaou K. C., Rigol S. (2018). A brief history of
antibiotics and select advances in their synthesis. J. Antibiot..

[2] Costa S. S., Viveiros M., Rosato A. E., Melo-Cristino J., Couto I. (2015). Impact of efflux in the development of multidrug resistance phenotypes
in Staphylococcus aureus. BMC Microbiol..

[3] Papkou A., Hedge J., Kapel N., Young B., MacLean R. C. (2020). Efflux
pump activity potentiates the evolution of antibiotic resistance across
S. aureus isolates. Nat. Commun..

[4] Shuster Y., Steiner-Mordoch S., Alon Cudkowicz N., Schuldiner S. (2016). A Transporter
Interactome Is Essential for the Acquisition of Antimicrobial Resistance
to Antibiotics. PLoS One.

[5] Jumbe N. L., Louie A., Miller M. H., Liu W., Deziel M. R., Tam V. H., Bachhawat R., Drusano G. L. (2006). Quinolone efflux
pumps play a central role in emergence of fluoroquinolone resistance
in Streptococcus pneumoniae. Antimicrob. Agents
Chemother..

[6] Schmalstieg A. M., Srivastava S., Belkaya S., Deshpande D., Meek C., Leff R., van Oers N. S., Gumbo T. (2012). The antibiotic
resistance arrow of time: efflux pump induction is a general first
step in the evolution of mycobacterial drug resistance. Antimicrob. Agents Chemother..

[7] Srivastava S., Musuka S., Sherman C., Meek C., Leff R., Gumbo T. (2010). Efflux-pump-derived
multiple drug resistance to ethambutol monotherapy
in Mycobacterium tuberculosis and the pharmacokinetics and pharmacodynamics
of ethambutol. J. Infect. Dis..

[8] Tsugawa H., Suzuki H., Muraoka H., Ikeda F., Hirata K., Matsuzaki J., Saito Y., Hibi T. (2011). Enhanced bacterial
efflux system is the first step to the development of metronidazole
resistance in Helicobacter pylori. Biochem.
Biophys. Res. Commun..

[9] Piddock L. J., Jin Y. F. (1999). Antimicrobial activity
and accumulation of moxifloxacin
in quinolone-susceptible bacteria. J. Antimicrob.
Chemother..

[10] Lee Y., Chen H., Yang Y., Chou Y., Chang T., Hsu W., Lin I., Sun J. (2020). AdeABC Efflux Pump Controlled by
AdeRS Two Component System Conferring Resistance to Tigecycline, Omadacycline
and Eravacycline in Clinical Carbapenem Resistant Acinetobacter nosocomialis. Front Microbiol..

[11] Shi Y., Hua X., Xu Q., Yang Y., Zhang L., He J., Mu X., Hu L., Leptihn S., Yu Y. (2020). Mechanism of eravacycline
resistance in Acinetobacter baumannii mediated by a deletion mutation
in the sensor kinase adeS, leading to elevated expression of the efflux
pump AdeABC. Infect., Genet. Evol..

[12] Wen Z., Shang Y., Xu G., Pu Z., Lin Z., Bai B., Chen Z., Zheng J., Deng Q., Yu Z. (2020). Mechanism
of Eravacycline Resistance in Clinical Enterococcus faecalis Isolates
From China. Front Microbiol..

[13] Cho J. C., Crotty M. P., White B. P., Worley M. V. (2018). What Is Old Is New
Again: Delafloxacin, a Modern Fluoroquinolone. Pharmacotherapy.

[14] Lomovskaya O., Bostian K. A. (2006). Practical applications and feasibility of efflux pump
inhibitors in the clinic—A vision for applied use. Biochem. Pharmacol..

[15] Mahmood H. Y., Jamshidi S., Sutton J. M., Rahman K. M. (2016). Current Advances
in Developing Inhibitors of Bacterial Multidrug Efflux Pumps. Curr. Med. Chem..

[16] Opperman T. J., Nguyen S. T. (2015). Recent advances
toward a molecular mechanism of efflux
pump inhibition. Front Microbiol..

[17] Laws M., Shaaban A., Rahman K. M. (2019). Antibiotic resistance
breakers: current
approaches and future directions. FEMS Microbiol.
Rev..

[18] Li X. Z., Plésiat P., Nikaido H. (2015). The challenge of efflux-mediated
antibiotic resistance in Gram-negative bacteria. Clin. Microbiol. Rev..

[19] German N., Wei P., Kaatz G. W., Kerns R. J. (2008). Synthesis and evaluation of fluoroquinolone
derivatives as substrate-based inhibitors of bacterial efflux pumps. Eur. J. Med. Chem..

[20] Xiao Z. P., Wang X. D., Wang P. F., Zhou Y., Zhang J. W., Zhang L., Zhou J., Zhou S. S., Ouyang H., Lin X. Y. (2014). Design,
synthesis, and evaluation of novel fluoroquinolone-flavonoid
hybrids as potent antibiotics against drug-resistant microorganisms. Eur. J. Med. Chem..

[21] Butler M. M., Lamarr W. A., Foster K. A., Barnes M. H., Skow D. J., Lyden P. T., Kustigian L. M., Zhi C., Brown N. C., Wright G. E. (2007). Antibacterial activity
and mechanism of action
of a novel anilinouracil-fluoroquinolone hybrid compound. Antimicrob. Agents Chemother..

[22] Samosorn S., Tanwirat B., Muhamad N., Casadei G., Tomkiewicz D., Lewis K., Suksamrarn A., Prammananan T., Gornall K. C., Beck J. L. (2009). Antibacterial
activity
of berberine-NorA pump inhibitor hybrids with a methylene ether linking
group. Bioorg. Med. Chem..

[23] Jamshidi S., Sutton J. M., Rahman K. M. (2018). Mapping
the Dynamic Functions and
Structural Features of AcrB Efflux Pump Transporter Using Accelerated
Molecular Dynamics Simulations. Sci. Rep..

[24] Allgood S. C., Su C.-C., Crooks
Amy L., Meyer Christian T., Zhou B., Betterton
Meredith D., Barbachyn Michael R., Yu Edward W., Detweiler Corrella S. (2023). Bacterial efflux pump modulators
prevent bacterial growth in macrophages and under broth conditions
that mimic the host environment. mBio.

[25] Da
Cruz A. V., Jiménez-Castellanos J.-C., Börnsen C., Van Maele L., Compagne N., Pradel E., Müller R. T., Meurillon V., Soulard D., Piveteau C. (2023). Pyridylpiperazine
efflux pump inhibitor boosts in vivo antibiotic efficacy against *K. pneumoniae*. EMBO Mol. Med..

[26] Wang S., Wang K., Song K., Lai Z. W., Li P., Li D., Sun Y., Mei Y., Xu C., Liao M. (2024). Structures
of the Mycobacterium tuberculosis efflux pump EfpA reveal the mechanisms
of transport and inhibition. Nat. Commun..

[27] Nakashima R., Sakurai K., Yamasaki S., Hayashi K., Nagata C., Hoshino K., Onodera Y., Nishino K., Yamaguchi A. (2013). Structural
basis for the inhibition of bacterial multidrug exporters. Nature.

[28] Ng E., Trucksis M., Hooper D. C. (1994). Quinolone
resistance mediated by
norA: physiologic characterization and relationship to flqB, a quinolone
resistance locus on the Staphylococcus aureus chromosome. Antimicrob. Agents Chemother..

[29] Muñoz-Bellido J. L., Manzanares M. A., Andrés J. M., Zufiaurre M. G., Ortiz G., Hernández M. S., García-Rodríguez J. (1999). Efflux pump-mediated
quinolone resistance in Staphylococcus aureus strains wild type for
gyrA, gyrB, grlA, and norA. Antimicrob. Agents
Chemother..

[30] Irwin J. J., Shoichet B. K. (2005). ZINC– a free database of commercially available
compounds for virtual screening. J. Chem. Inf.
Mod..

[31] Markham P. N., Westhaus E., Klyachko K., Johnson M. E., Neyfakh A. A. (1999). Multiple
novel inhibitors of the NorA multidrug transporter of Staphylococcus
aureus. Antimicrob. Agents Chemother..

[32] Laws M., Hind C., Favaron A., Jamshidi S., Evans B., Clifford M., Sutton J. M., Rahman K. M. (2020). N1-Benzofused Modification
of Fluoroquinolones Reduces Activity Against Gram-Negative Bacteria. ACS Omega.

[33] Cecchetti V., Fravolini A., Lorenzini M. C., Tabarrini O., Terni P., Xin T. (1996). Studies on
6-Aminoquinolones: Synthesis
and Antibacterial Evaluation of 6-Amino-8-methylquinolones. J. Med. Chem..

[34] Bohnert J. A., Kern W. V. (2005). Selected arylpiperazines
are capable of reversing multidrug
resistance in Escherichia coli overexpressing RND efflux pumps. Antimicrob. Agents Chemother..

[35] Laws M., Hind C., Rahman K. M., Sutton J. M., Wand M. E. (2021). Whole Genome
Sequencing of Staphylococcus aureus SA-1199B Reveals Previously Unreported
Mutations. Int. J. Antimicrob. Agents.

[36] Beyer R., Pestova E., Millichap J. J., Stosor V., Noskin G. A., Peterson L. R. (2000). A convenient assay
for estimating the possible involvement
of efflux of fluoroquinolones by *Streptococcus pneumoniae* and *Staphylococcus aureus*: evidence for diminished
moxifloxacin, sparfloxacin, and trovafloxacin efflux. Antimicrob. Agents Chemother..

[37] Schmitz F. J., Fluit A. C., Luckefahr M., Engler B., Hofmann B., Verhoef J., Heinz H. P., Hadding U., Jones M. E. (1998). The effect
of reserpine, an inhibitor of multidrug efflux pumps, on the in-vitro
activities of ciprofloxacin, sparfloxacin and moxifloxacin against
clinical isolates of Staphylococcus aureus. J. Antimicrob. Chemother..

[38] Hooper D. C., Jacoby G. A. (2016). Topoisomerase Inhibitors:
Fluoroquinolone Mechanisms
of Action and Resistance. Cold Spring Harbor
Perspect. Med..

[39] Lahiri S.
D., Kutschke A., McCormack K., Alm R. A. (2015). Insights into the
mechanism of inhibition of novel bacterial topoisomerase inhibitors
from characterization of resistant mutants of Staphylococcus aureus. Antimicrob. Agents Chemother..

[40] Lai C. C., Chen C. C., Lu Y. C., Chuang Y. C., Tang H. J. (2018). The clinical
significance of silent mutations with respect to ciprofloxacin resistance
in MRSA. Infect. Drug. Resist..

[41] Mitscher L. A., Sharma P. N., Chu D. T., Shen L. L., Pernet A. G. (1987). Chiral
DNA gyrase inhibitors. 2. Asymmetric synthesis and biological activity
of the enantiomers of 9-fluoro-3-methyl-10-(4-methyl-1-piperazinyl)-7-oxo-2,
3-dihydro-7H-pyrido [1, 2, 3-de]-1, 4-benzoxazine-6-carboxylic acid
(ofloxacin). J. Med. Chem..

[42] Baumann M., Baxendale I. R. (2013). An overview
of the synthetic routes to the best selling
drugs containing 6-membered heterocycles. Beilstein
J. Org. Chem..

[43] Saxena D., Maitra R., Bormon R., Czekanska M., Meiers J., Titz A., Verma S., Chopra S. (2023). Tackling the
outer membrane: facilitating compound entry into Gram-negative bacterial
pathogens. Npj Antimicrob Resist..

[44] Xu C., Bilya S. R., Xu W. (2019). adeABC efflux gene in Acinetobacter
baumannii. New Microbes New Infect..

[45] Davies, O. L. ; Bennett, S. WHO publishes list of bacteria for which new antibiotics are urgently needed; World Health Organisation, 2017. https://www.who.int/news-room/detail/27-02-2017-who-publishes-list-of-bacteria-for-which-new-antibiotics-are-urgently-needed (accessed 13 November 2024).

[46] Bell, B. ; Bell, M. ; Bowen, A. ; Antibiotic resistance threats in the United States, 2013; Centers for Disease Control and Prevention, 2013. https://www.cdc.gov/drugresistance/pdf/ar-threats-2013-508.pdf (accessed 06 November 2024).

[47] Gallagher L. A., Ramage E., Weiss E.J., Radey M., Hayden H.S., Held K.G., Huse H.K., Zurawski D.V., Brittnacher M.J., Manoil C. (2015). Resources for Genetic and Genomic Analysis of Emerging
Pathogen Acinetobacter baumannii. J. Bacteriol..

[48] Blair J. M. A., Webber M. A., Baylay A. J., Ogbolu D. O., Piddock L. J. V. (2015). Molecular
mechanisms of antibiotic resistance. Nat. Rev.
Microbiol..

[49] Sharma D., Misba L., Khan A. U. (2019). Antibiotics versus biofilm: an emerging
battleground in microbial communities. Antimicrob.
Resist. Infect. Cont..

[50] Waterhouse A., Bertoni M., Bienert S., Studer G., Tauriello G., Gumienny R., Heer F. T., de Beer T. A. P., Rempfer C., Bordoli L. (2018). SWISS-MODEL:
homology modelling of protein structures
and complexes. Nucleic Acids Res..

[51] Doerr S., Harvey M. J., Noe F., De Fabritiis G. H. (2016). High-Throughput
Molecular Dynamics for Molecular Discovery. J. Chem. Theory Comput..

[52] Martinez-Rosell G., Giorgino T., De Fabritiis G. (2017). PlayMolecule
ProteinPrepare: A Web
Application for Protein Preparation for Molecular Dynamics Simulations. J. Chem. Inf. Model..

[53] Pogozheva I. D., Armstrong G. A., Kong L., Hartnagel T. J., Carpino C. A., Gee S. E., Picarello D. M., Rubin A. S., Lee J., Park S. (2022). Comparative
Molecular Dynamics Simulation Studies of Realistic Eukaryotic, Prokaryotic,
and Archaeal Membranes. J. Chem. Inf. Model..

[54] Huang J., Rauscher S., Nawrocki G., Ran T., Feig M., de Groot B. L., Grubmuller H., MacKerell A. (2017). CHARMM36m:
an improved force field for folded and intrinsically disordered proteins. Nat. Methods.

[55] Jorgensen W. L., Jenson C. (1998). Temperature
dependence of TIP3P, SPC, and TIP4P water
from NPT Monte Carlo simulations: Seeking temperatures of maximum
density. J. Comput. Chem..

[56] Vanommeslaeghe K., MacKerell D. A. (2012). Automation
of the CHARMM General Force Field (CGenFF)
I: bond perception and atom typing. J. Chem.
Inf. Model..

[57] Harvey M.
J., Giupponi G., Fabritiis G. D. (2009). ACEMD: Accelerating Biomolecular
Dynamics in the Microsecond Time Scale. J. Chem.
Theory Comput..

[58] Schmidtke P., Bidon-Chanal A., Luque F. J., Barril X. (2011). MDpocket: open-source
cavity detection and characterization on molecular dynamics trajectories. Bioinformatics.

[59] Gowers, R. ; Linke, M. ; Barnoud, J. ; Reddy, T. ; Melo, M. ; Seyler, S. L. ; Dotson, D. ; Domanski, J. ; Buchoux, S. ; Kenney, I. MDAnalysis: a Python package for the rapid analysis of molecular dynamics simulations. PeerJ 2016.

[60] Scherer M.
K., Trendelkamp-Schroer B., Paul F., Perez-Hernandez G., Hoffmann M., Plattner N., Wehmeyer C., Prinz J. H., Noe F. (2015). PyEMMA 2: A Software
Package for Estimation, Validation, and Analysis
of Markov Models. J. Chem. Theory Comput..

[61] Jurrus E., Engel D., Star K., Monson K., Brandi J., Felberg L. E., Brookes D. H., Wilson L., Chen J., Liles K. (2018). Improvements to the APBS biomolecular solvation software
suite. Protein Sci..

[62] Geddes E. J., Li Z., Hergenrother P. J. (2021). An LC-MS/MS
assay and complementary
web-based tool to quantify and predict compound accumulation in E.
coli. Nat. Prot..

[63] Aicher, T. D. ; Chen, Z. ; Chen, Y. ; Faul, M. M. ; Krushinski, J. H. J. ; Le Huerou, Y. ; Pineiro-Nunez, M. M. ; Rocco, V. P. ; Ruley, K. M. ; Schaus, J. M. Piperazine Substituted Aryl Benzodiazepines and Their Use as Dopamine Receptor Antagonists for the Treatment of Psychotic Disorders WO 2,003,082,877 A1, 2003.

[64] Sanchez J.
P., Domagala J. M., Hagen S. E., Heifetz C. L., Hutt M. P., Nichols J. B., Trehan A. K. (1988). Quinolone antibacterial agents. Synthesis
and structure-activity relationships of 8-substituted quinoline-3-carboxylic
acids and 1,8-naphthyridine-3-carboxylic acids. J. Med. Chem..

[65] Dixit S. K., Yadav N., Kumar S., Good L., Awasthi S. K. (2014). Synthesis
and antibacterial activity of novel fluoroquinolone analogs. Med. Chem. Res..

[66] Thakur M. S., Nayal O. S., Upadhyay R., Kumar N., Maurya S. K. (2018). 2-Aminoquinazolin-4­(3H)-one
as an Organocatalyst for the Synthesis of Tertiary Amines. Org. Lett..

[67] Mirazur Rahman, K. ; Jamshidi, S. ; Benjamin Laws, M. ; Nahar, K. ; Mark Sutton, J. ; Hind, C. Antibiotic resistance breakers WO 2018220365 A1, 2017.

[68] Rueping M., Stoeckel M., Sugiono E., Theissmann T. (2010). Asymmetric
metal-free synthesis of fluoroquinolones by organocatalytic hydrogenation. Tetrahedron.

[69] Hong, W. ; Lee, K.-J. Baylis-Hillman Route to Several Quinolone Antibiotic Intermediates. Synthesis 2006, 37.

[70] Picconi P., Hind C. K., Nahar K. S., Jamshidi S., Di Maggio L., Saeed N., Evans B., Solomons J., Wand M. E., Sutton J. M., Rahman K. M. (2020). New Broad-Spectrum Antibiotics Containing
a Pyrrolobenzodiazepine Ring with Activity against Multidrug-Resistant
Gram-Negative Bacteria. J. Med. Chem..

[71] Jalili V., Afgan E., Gu Q., Clements D., Blankenberg D., Goecks J., Taylor J., Nekrutenko A. (2020). The Galaxy
Platform for Accessible, Reproducible and Collaborative Biomedical
Analyses: 2020 Update. Nucleic Acids Res..

