# Supplementary material for: Designing Antibiotics with Inherent Resistance to Efflux as a Strategy to Revive Discovery against Multidrug-Resistant Pathogens
Source: J Med Chem. 2026 May 29;69(11):13071–98. doi: 10.1021/acs.jmedchem.6c00060 (PMC13266999; doi:10.1021/acs.jmedchem.6c00060)
Supplement: Supplementary file 15 [file jm6c00060_si_015.pdf]

Designing antibiotics with inherent resistance to efflux as a strategy to revive discovery against multidrug resistant pathogens

Mark Laws[± 1], Charlotte K. Hind[± 2], Kazi Sharmin Nahar[± 1,3], Melanie Clifford[2], Caleb Marsh[2], Taha al Adhami[1], Brice Louis[1], Manming Xu[4], Saleh O Alyemni[4], Shozeb Haider[4], Mushtaq Hassan[1], Nupur Gargate[1], Matthew E. Wand[2], J. Mark Sutton*[1, 2] & Khondaker Miraz Rahman*[1]

[±] These authors contributed equally to this work.

Affiliations

[1]School of Cancer and Pharmaceutical Sciences, King's College London, Franklin-Wilkins Building, 150 Stamford Street, London SE1 9NH, United Kingdom

[2]Countermeasures Development, Evaluation and Preparedness, Public Health Microbiology, UK Health Security Agency, Manor Farm Road, Porton Down, Salisbury SP4 0JG, United Kingdom

[3]Department of Natural Sciences, University of Middlesex, The Burroughs, Hendon, London NW4 4BT, United Kingdom

[4]UCL School of Pharmacy, University College London, London WC1N 1AX, United Kingdom

Correspondence

Correspondence should be addressed to Khondaker Miraz Rahman (k.miraz.rahman@kcl.ac.uk) and J. Mark Sutton (mark.sutton@ukhsa.gov.uk)

Supporting Information

Table of Contents

Section 1: Experimental Methods – Chemistry S3

Section 2: HRMS and NMR Spectra S5

2.1 HRMS Spectra S5

2.1.1 HRMS Spectra for Compound 7 .... S5

2.1.2 HRMS Spectra for First Generation ERB-fluoroquinolones and Regioisomers S6

2.1.3 HRMS Spectra for Wider First-Generation ERB-fluoroquinolones S9

2.1.4 HRMS Spectra for Second-Generation ERB-fluoroquinolones S23

2.2 NMR Spectra S32

2.2.1 ^{1}H, ^{13}C and ^{19}F NMR Spectra for Levofloxacin and Precursors S33

2.2.2 ^{1}H, ^{13}C and ^{19}F NMR Spectra for ML-77-005 Regioisomers and Precursors S47

2.2.3 ^{1}H and ^{13}C spectrum for Wider First-Generation ERB-Fluoroquinolones S79

2.2.4 ^{1}H and ^{13}C-NMR Spectra for Second Generation ERB-fluoroquinolones S119

2.3 LC Traces of Lead Compounds S141

Section 3: Supporting Figures S143

Section 4: Supporting Tables S155

Section 1: Experimental Methods – Chemistry

Preparative Liquid Chromatography-Mass Spectrometry (preparative LC-MS)

Preparative liquid chromatography-mass spectrometry (preparative LC-MS) was performed on an Agilent 1290 Infinity II Preparative LC/MSD System comprised of the following subunits: 1290 MS flow modulator, 1290 prep fraction collector, 1290 prep column compartment, 1290 prep bin pump, 1290 valve drive, 1260 prep autosampler, 1260 diode array detector WR, 1260 quat pump, InfinityLab LC/MSD, MS40 rough pump and Genius NM32LA-A 230V nitrogen generator. The column used was a Phenomenex Luna® 5µm C18(2) 100Å LC column (100 x 21.2 mm). Mobile phases were water (A) and acetonitrile (B); formic acid (0.1%) was added to A to ensure acidic conditions throughout each purification run. Samples were wet loaded in dimethyl sulfoxide. The following methods were employed for compound purification:

Method 1 was run over a total time of 10 minutes at a flow rate of 20 mL/min. Mobile phase A consisted of water with 0.1% formic acid, and mobile phase B consisted of acetonitrile with 0.1% formic acid. The gradient started at 95% A and 5% B at 1 minute, changed to 70% A and 30% B at 2 minutes, then to 50% A and 50% B at 5.5 minutes. The gradient was then increased to 10% A and 90% B at 7 minutes and held at 10% A and 90% B until 8.5 minutes. It was then returned to 80% A and 20% B at 9 minutes and maintained at 80% A and 20% B until 10 minutes.

Method 2 was run over a total time of 10 minutes at a flow rate of 20 mL/min. Mobile phase A consisted of water with 0.1% formic acid, and mobile phase B consisted of acetonitrile with 0.1% formic acid. The gradient started at 95% A and 5% B at 1 minute, changed to 80% A and 20% B at 2.5 minutes, and then increased to 10% A and 90% B at 6.5 minutes. This composition was held at 10% A and 90% B until 8.5 minutes. The gradient was then returned to 80% A and 20% B at 9 minutes and maintained at 80% A and 20% B until 10 minutes.

Method 3 was run over a total time of 10 minutes at a flow rate of 20 mL/min. Mobile phase A consisted of water with 0.1% formic acid, and mobile phase B consisted of acetonitrile with 0.1% formic acid. The gradient started at 60% A and 40% B at 1 minute, changed to 20% A and 80% B at 7.4 minutes, and then increased to 10% A and 90% B at 7.5 minutes. This composition was held at 10% A and 90% B until 9.5 minutes. The gradient was then returned to 70% A and 30% B at 10 minutes.

Method 4 was run over a total time of 6 minutes at a flow rate of 20 mL/min. Mobile phase A consisted of water with 0.1% formic acid, and mobile phase B consisted of acetonitrile with 0.1% formic acid. The gradient started at 50% A and 50% B at 1 minute, changed to 35% A and 65% B at 5 minutes, and then increased to 10% A and 90% B at 5.25 minutes. This composition was held at 10% A and 90% B until 5.5 minutes. The gradient was then returned to 50% A and 50% B at 6 minutes.

Liquid Chromatography-Mass Spectrometry (LC-MS)

LC-MS analysis was performed either on a Waters Alliance 2695 HPLC coupled to a Waters Micromass ZQ instrument with a Waters 2996 PDA or an Agilent InfinityLab LC/MSD System consisting of an Agilent 1290 Infinity II Analytical-Scale LC Purification System coupled to a 6120 Quadrupole mass spectrometer. In both cases, high-performance liquid chromatography was carried out using an Onyx™ Monolithic C18 column (50 x 4.6 mm) with water (A) and acetonitrile (B) as the mobile phases. Formic acid (0.1%) was added to both to ensure acidic conditions throughout the analysis. 100-200 µL of

eluent was split *via* a zero dead volume T piece and passed into the mass spectrometer. The wavelength range of the UV detector was 220-500 nm. Gradient conditions used were as follows:

Method A was run over a total time of 5 minutes at a flow rate of 1.0 mL/min. Mobile phase A consisted of water with 0.1% formic acid, and mobile phase B consisted of acetonitrile with 0.1% formic acid. The gradient was at 10% A and 90% B at 3 minutes, changed to 5% A and 95% B at 3.5 minutes, and was held at 5% A and 95% B until 4.5 minutes. The gradient was then returned to 95% A and 5% B at 5 minutes.

Method B was run over a total time of 10 minutes at a flow rate of 0.5 mL/min. Mobile phase A consisted of water with 0.1% formic acid, and mobile phase B consisted of acetonitrile with 0.1% formic acid. The gradient was at 50% A and 50% B at 3 minutes, changed to 20% A and 80% B at 5 minutes, and then increased to 5% A and 95% B at 6.5 minutes. This composition was held at 5% A and 95% B until 8 minutes. The gradient was then returned to 95% A and 5% B at 8.2 minutes and maintained at 95% A and 5% B until 10 minutes.

For the Waters system: Mass spectrometry data (both ESI+ and ESI- modes) were collected using the following Waters Micromass ZQ parameters: capillary (kV), 3.38; cone (V), 35; extractor (V), 3.0; source temperature (°C), 100; de-solvation temperature (°C), 200; cone flow rate (L/h), 50; de-solvation flow rate (L/h), 250.

For the Agilent system: Analytical liquid chromatography was carried out using the following parameters: injection volume 10 µL; draw speed 100 µL/min; ejection speed 400 µL/min; wait time after drawing 1.2 s. Mass spectrometry data (both ESI+ and ESI- modes) were collected using the following parameters: capillary voltage 4 kV (ESI+), 3.5 kV (ESI-); drying gas flow 13.0 L/min; nebuliser pressure 50 psig (method A), 30 psig (method B), 60 psig (maximum); drying gas temperature 350°C; mass range 150-1,200 Da; fragmentor 70; gain 1.00; stepsize 0.10; speed 2,600 u/sec.

Section 2: HRMS and NMR Spectra

2.1 HRMS Spectra

2.1.1 HRMS Spectra for Compound 7

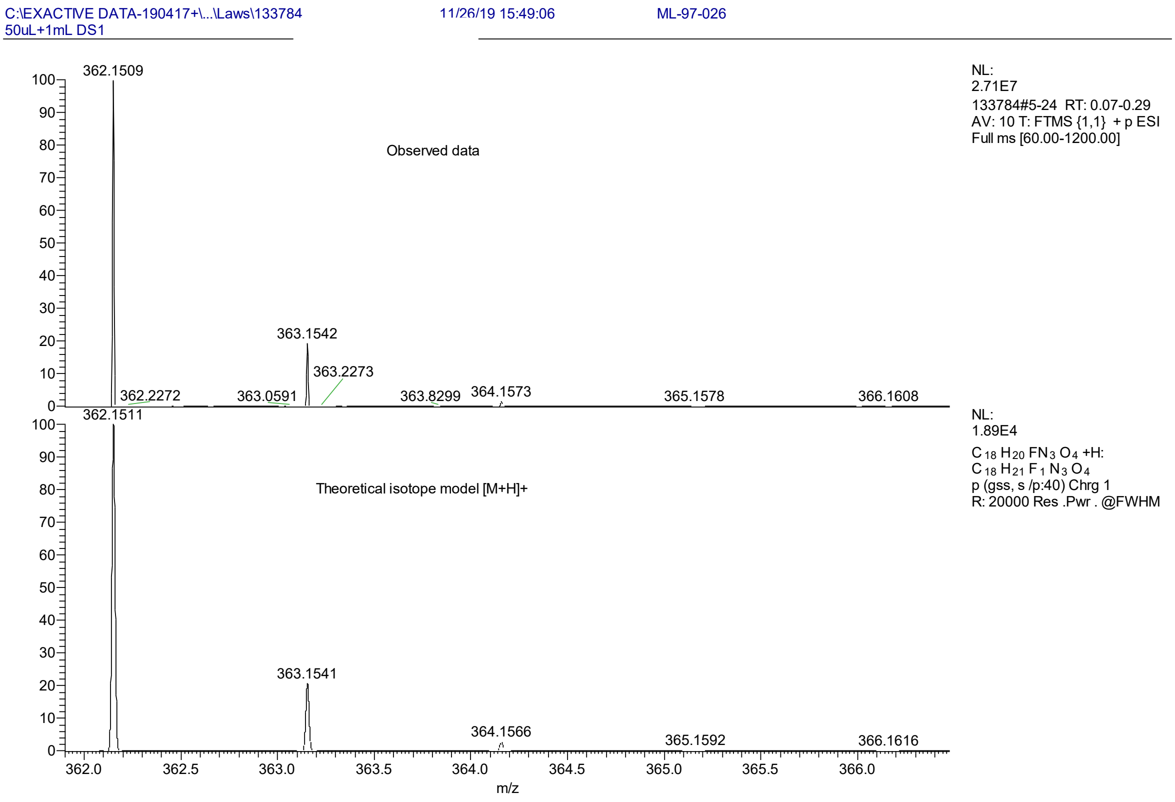

2.1.2 HRMS Spectra for First Generation ERB-fluoroquinolones and Regioisomers

Note – HRMS for first generation ERB-fluoroquinolones were run using the HCl salts of each compound.

8 Salt (ML-77-005 HCl salt)

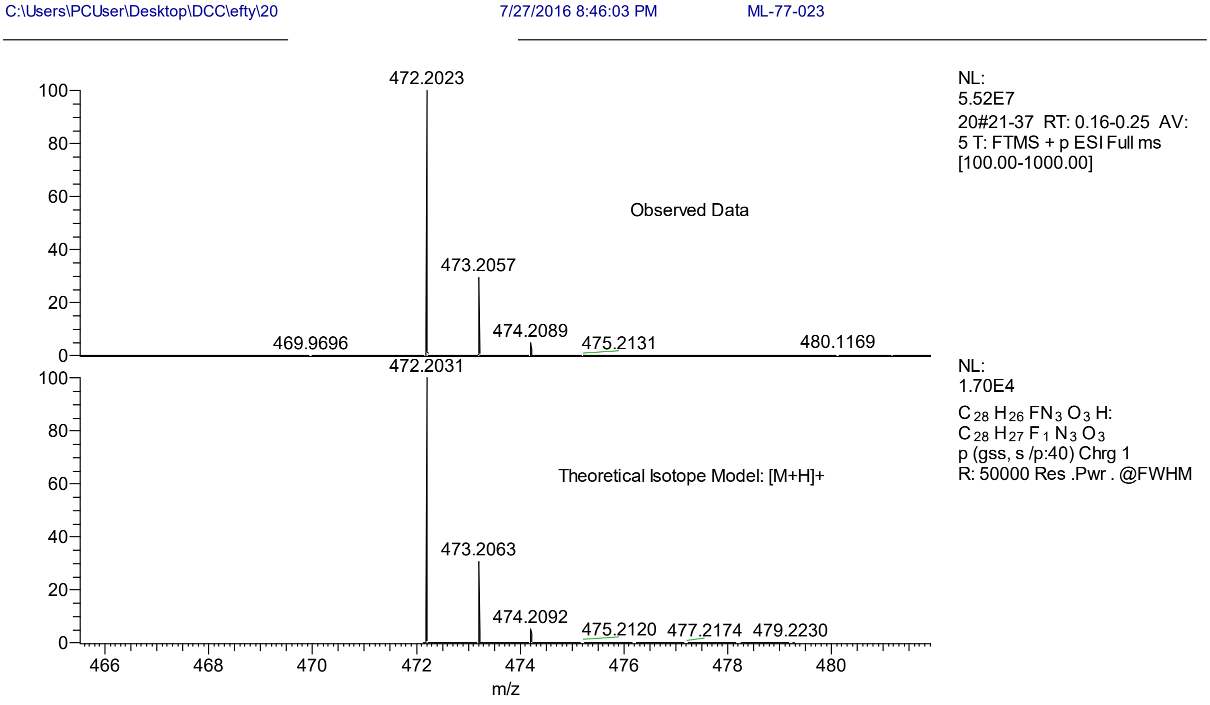

Compound 12

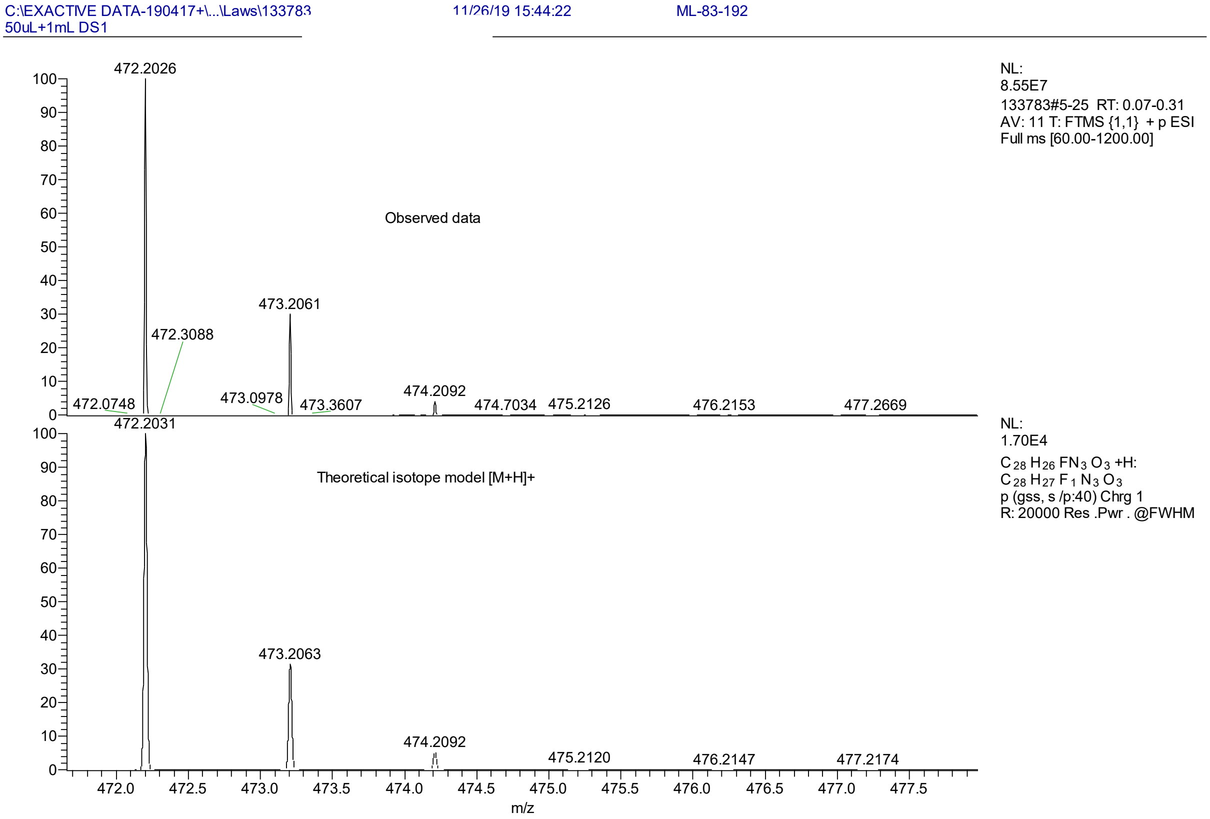

Compound 19

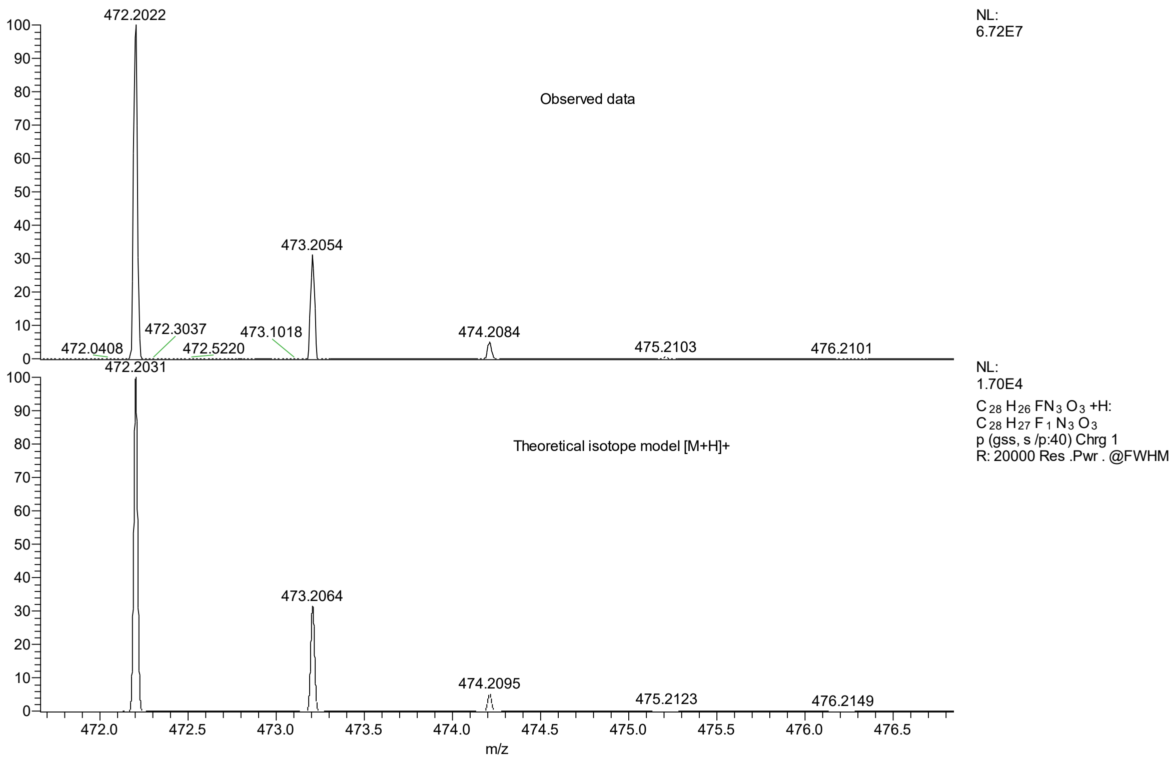
NL: 6.72E7
Observed data
472.2022
473.2054
474.2084
475.2103
476.2101
472.0408
472.3037
472.5220
473.1018
NL: 1.70E4
C 28 H 26 FN 3 O 3 +H:
C 28 H 27 F 1 N 3 O 3
p (gss, s /p:40) Chrg 1
R: 20000 Res .Pwr . @FWHM
Theoretical isotope model [M+H]+
472.2031
473.2064
474.2095
475.2123
476.2149
m/z

2.1.3 HRMS Spectra for Wider First-Generation ERB-fluoroquinolones

Compound 20 Salt

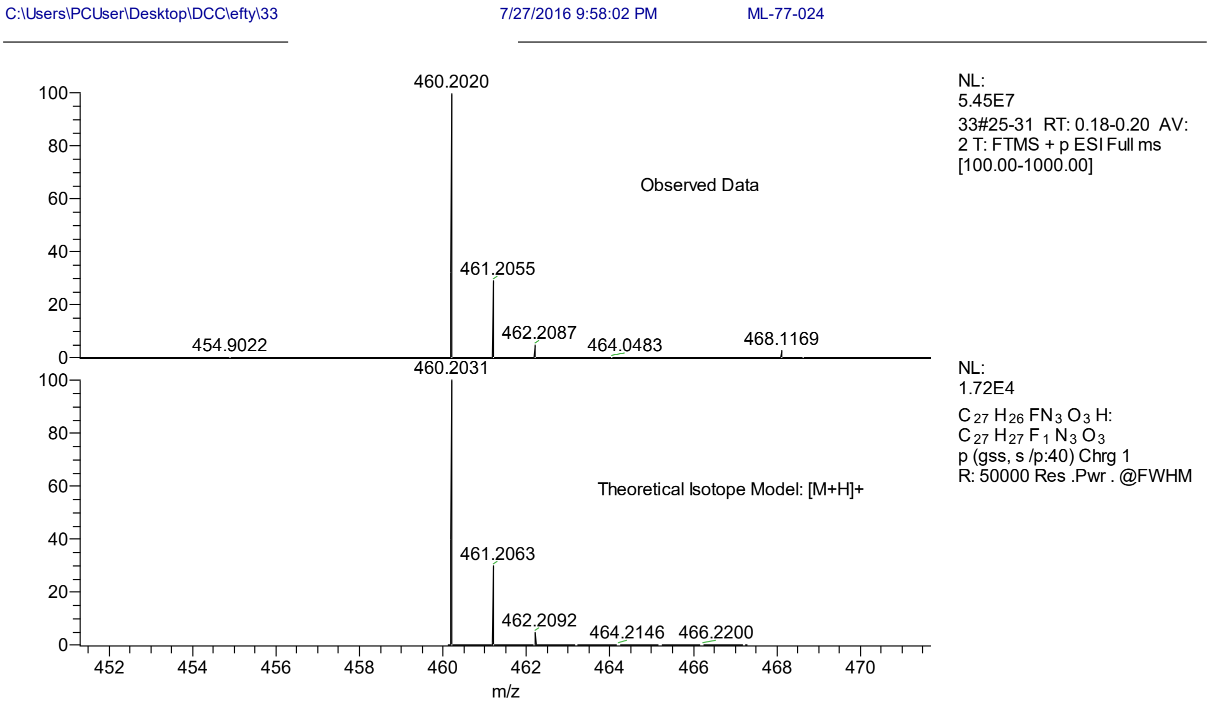

Compound 21 Salt

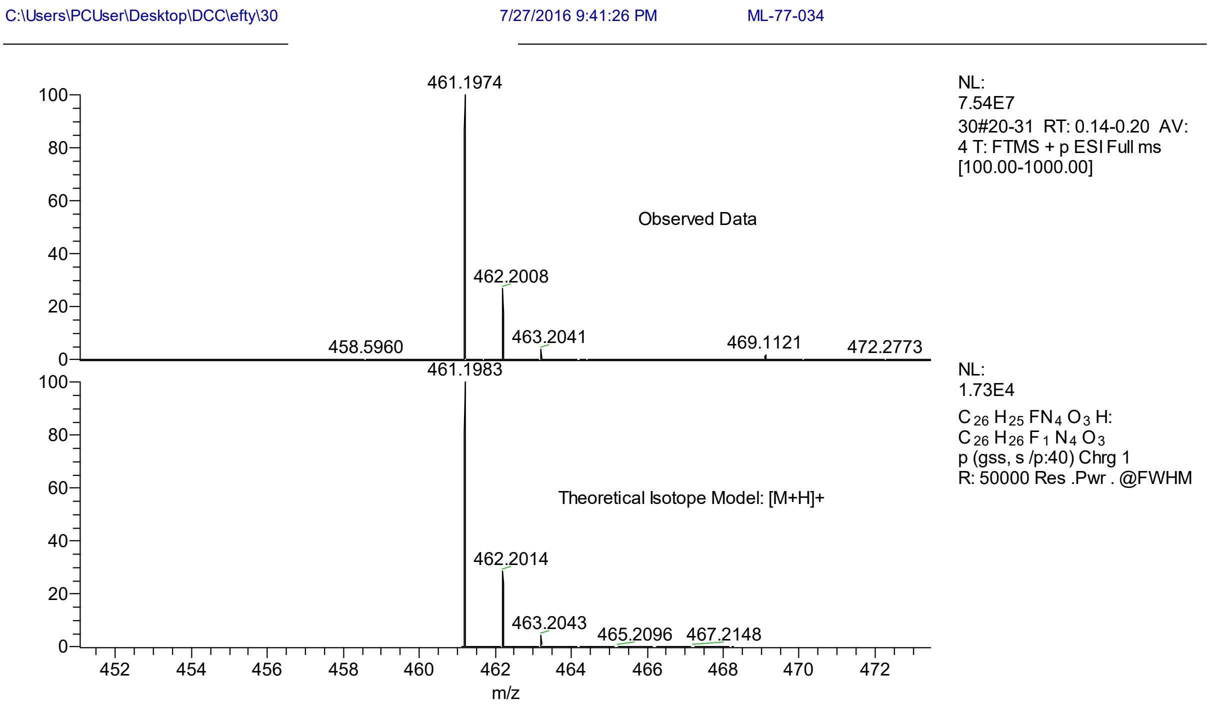

Compound 22

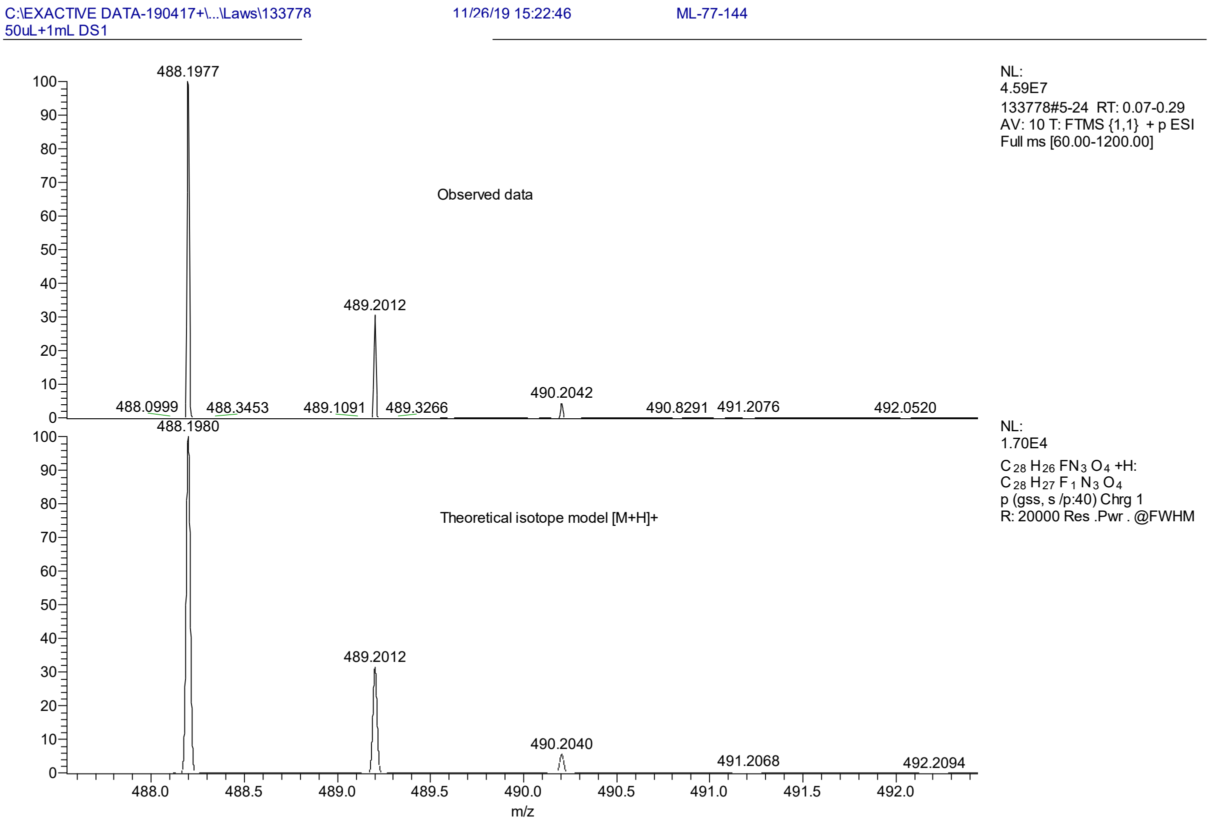

Compound 23 Salt

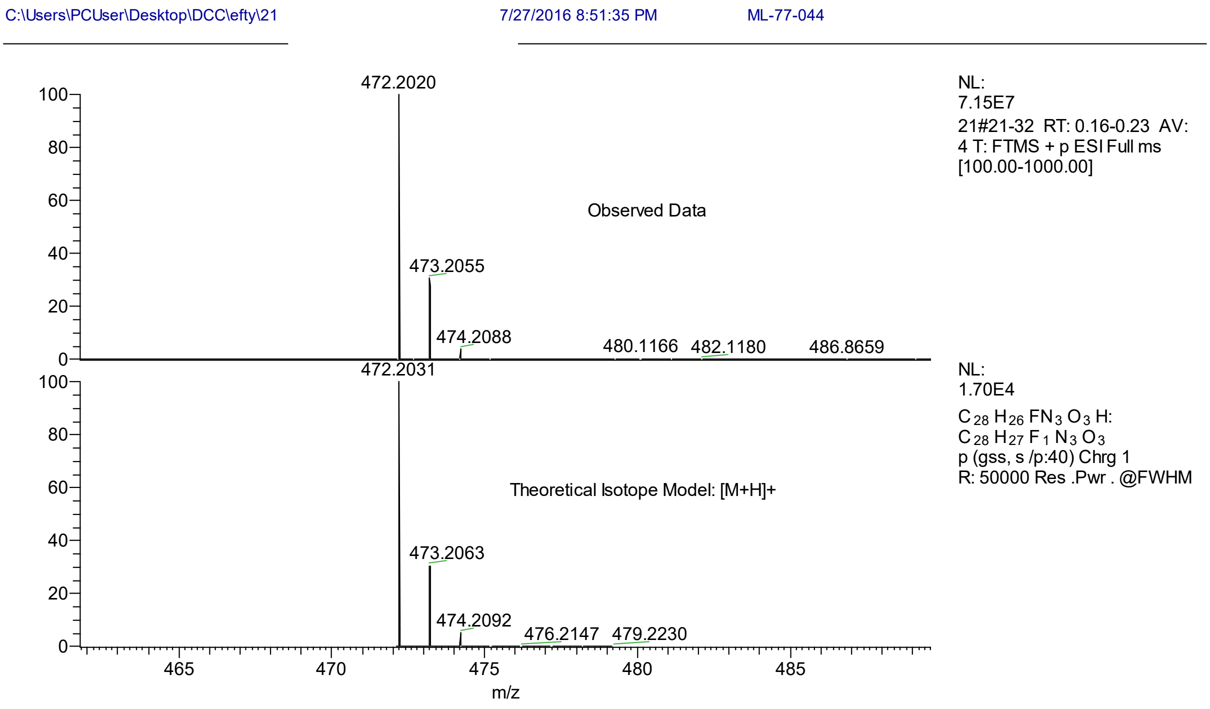

Compound 24 Salt

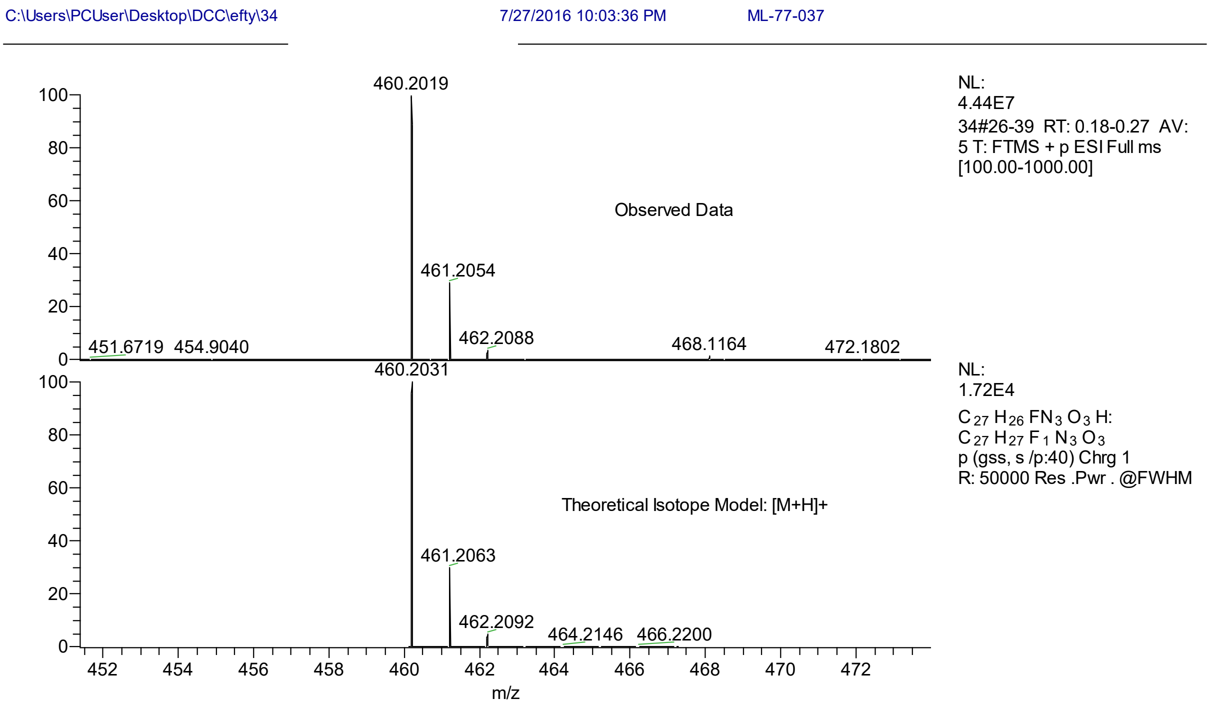

Compound 25 Salt

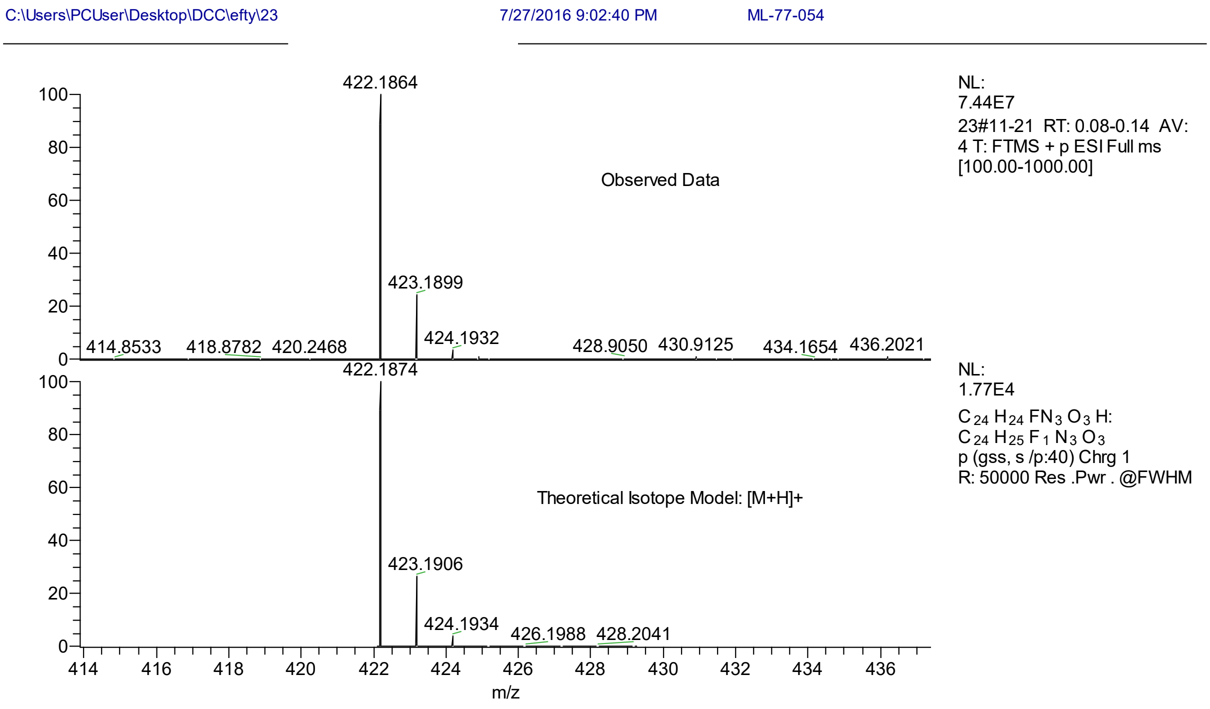

Compound 26 Salt

C:\Users\PCUser\Desktop\DCC\efty\36 7/27/2016 10:14:40 PM ML-77-055

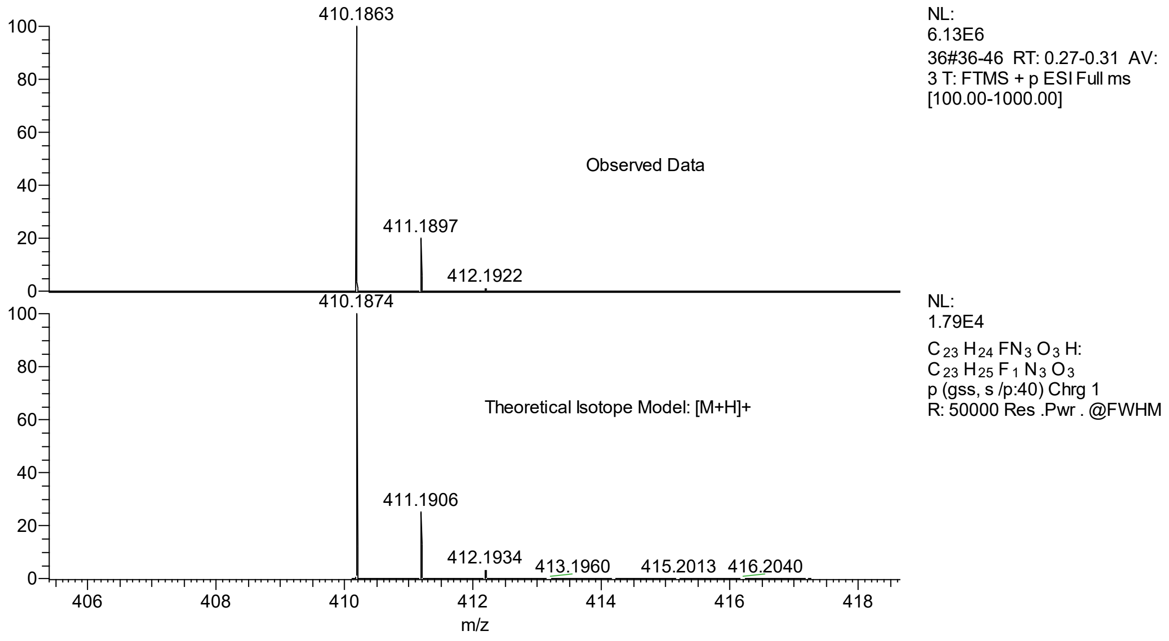

Compound 27 Salt

C:\Users\PCUser\Desktop\DCC\efty\26 7/27/2016 9:19:18 PM ML-77-063

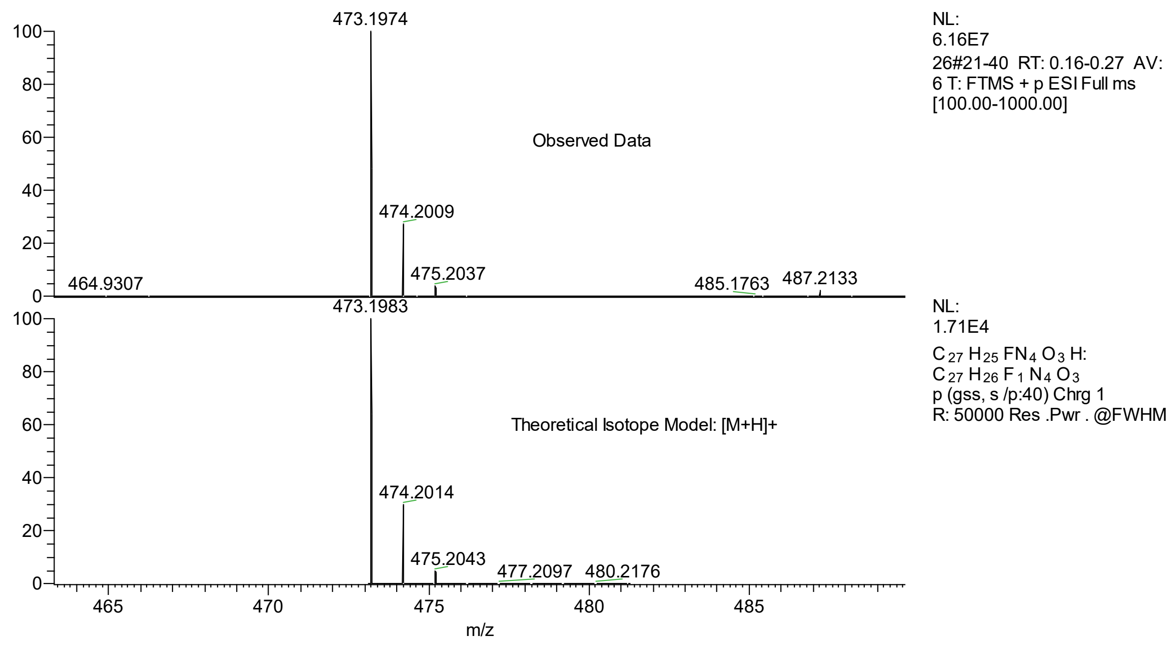

Compound 28 Salt

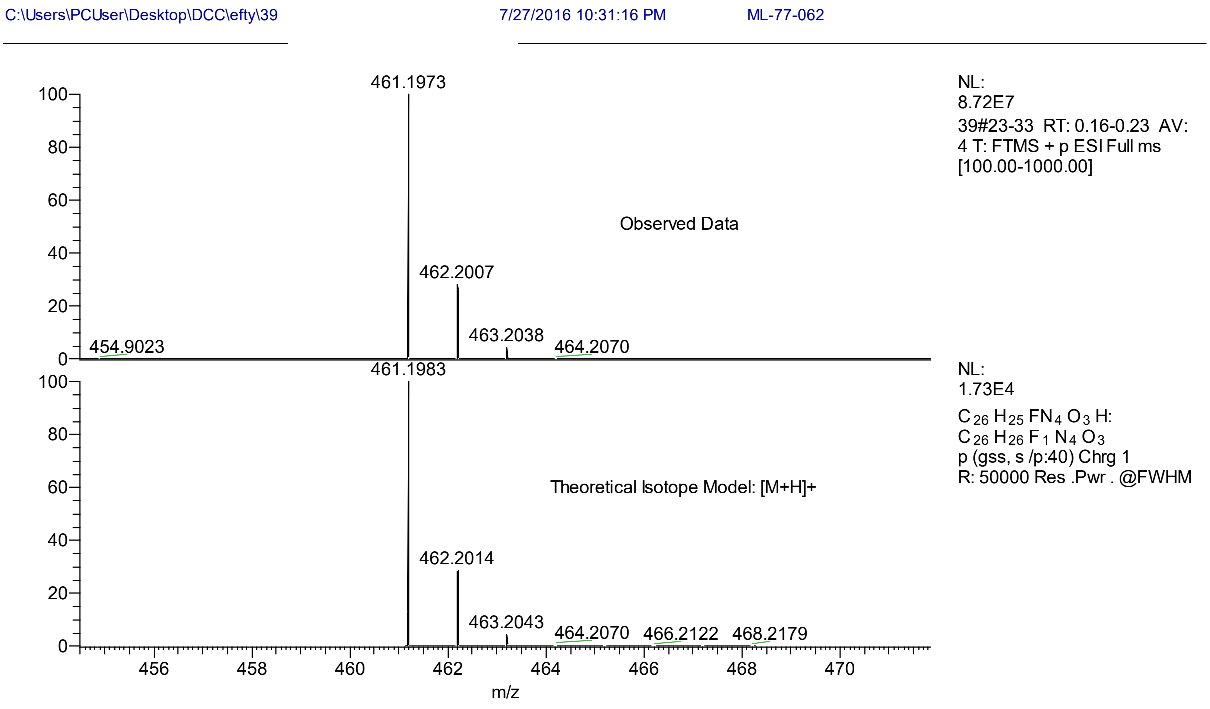

Compound 29 Salt

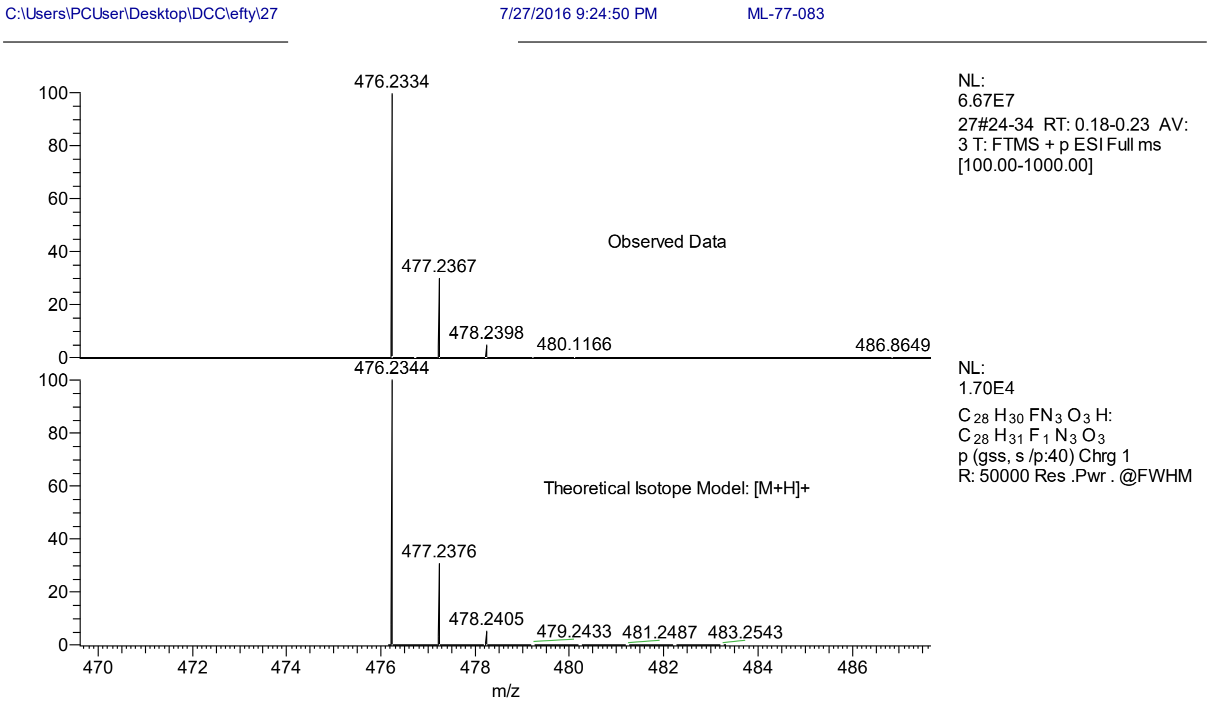

Compound 30 Salt

C:\Users\PCUser\Desktop\DCC\efty\40 7/27/2016 10:36:48 PM ML-77-064

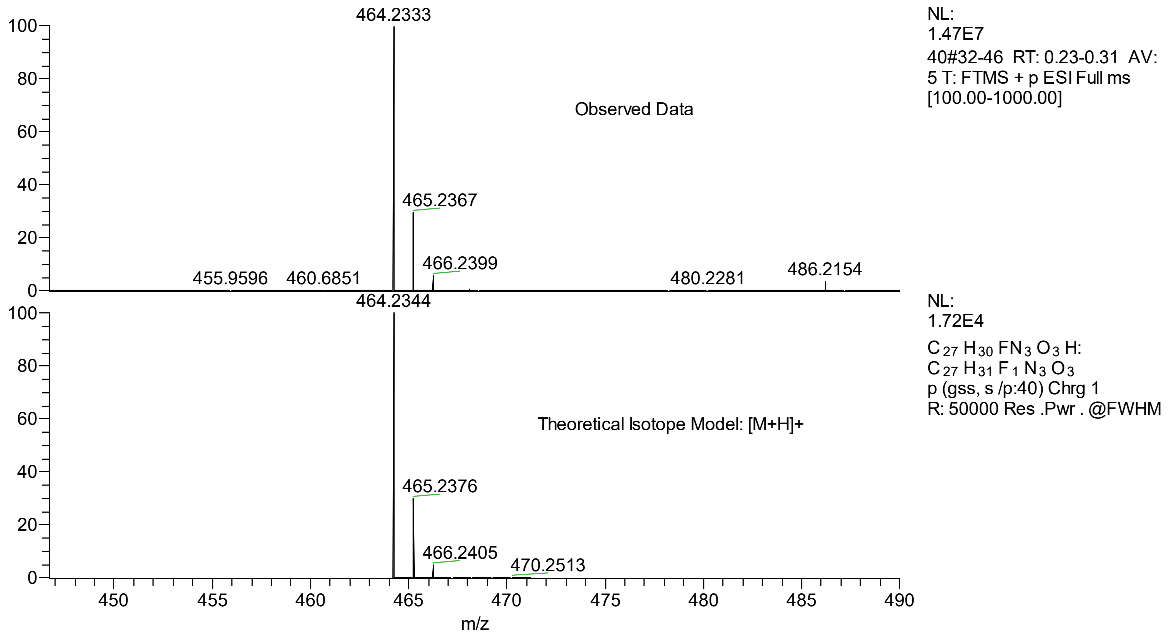

Compound 31 Salt

C:\Users\PCUser\Desktop\DCC\efty\29 7/27/2016 9:35:54 PM ML-77-119

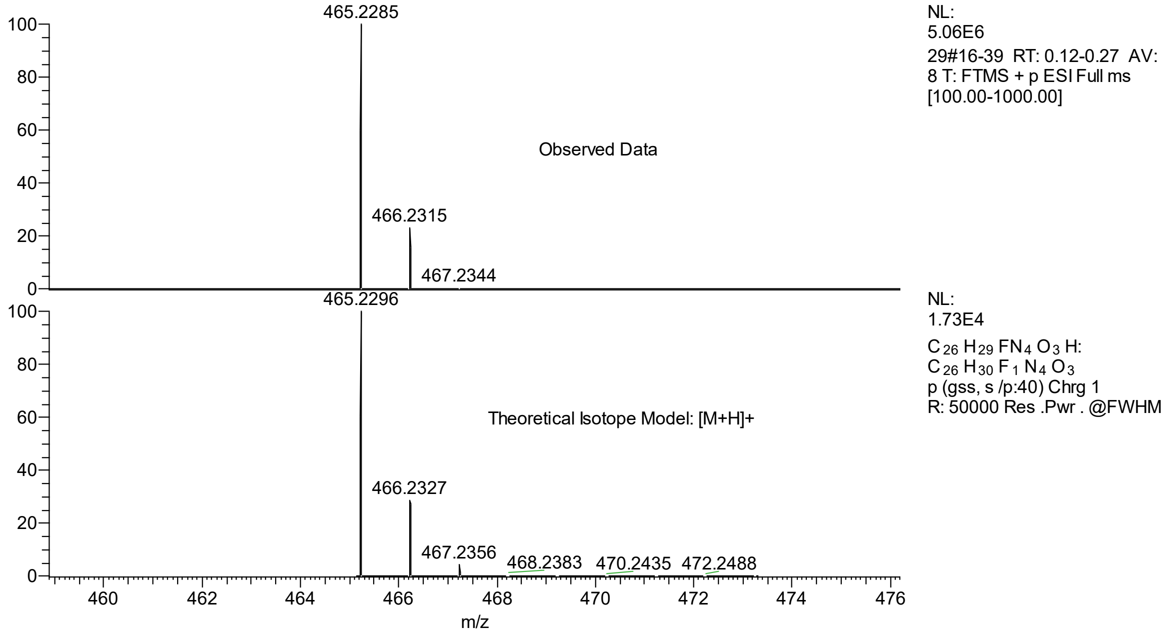

Compound 32 Salt

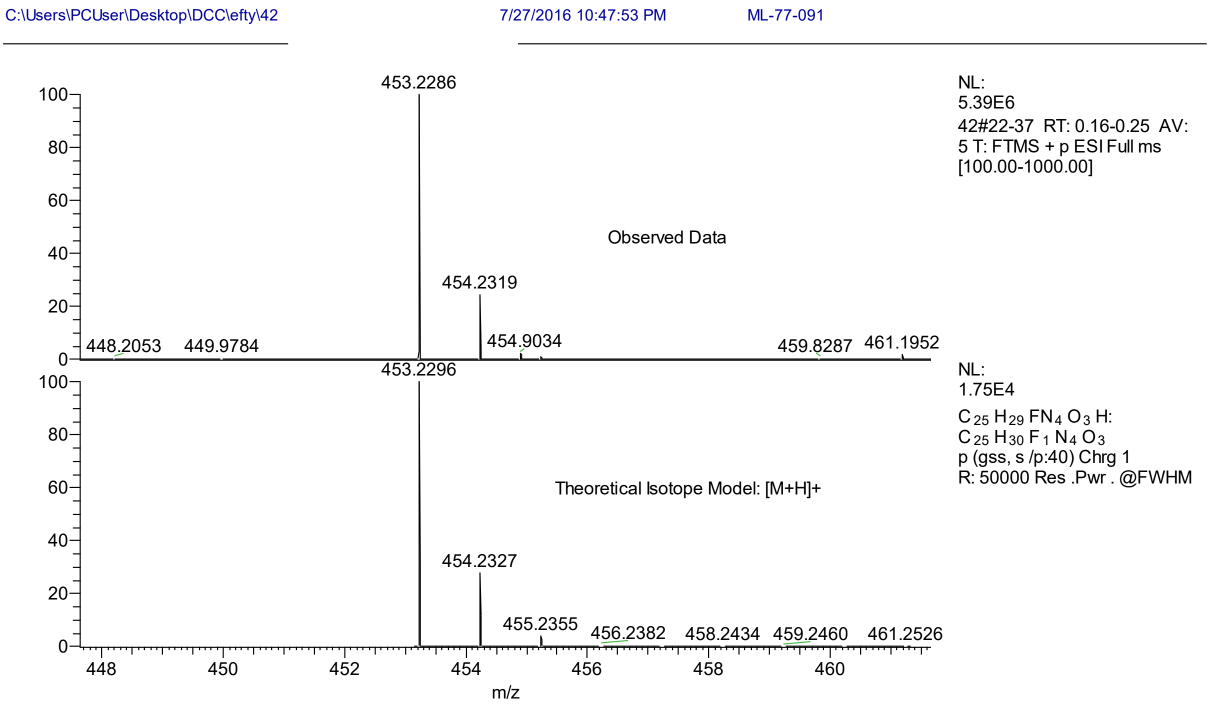

Compound 33 Salt

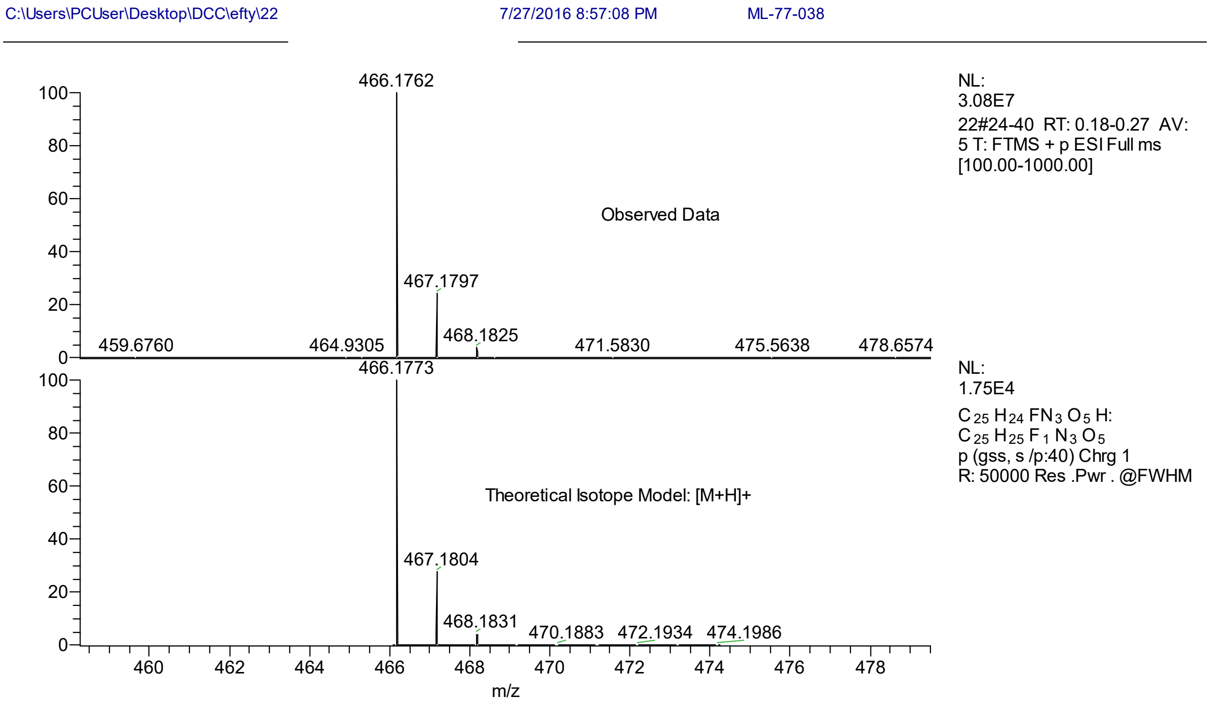

Compound 34 Salt

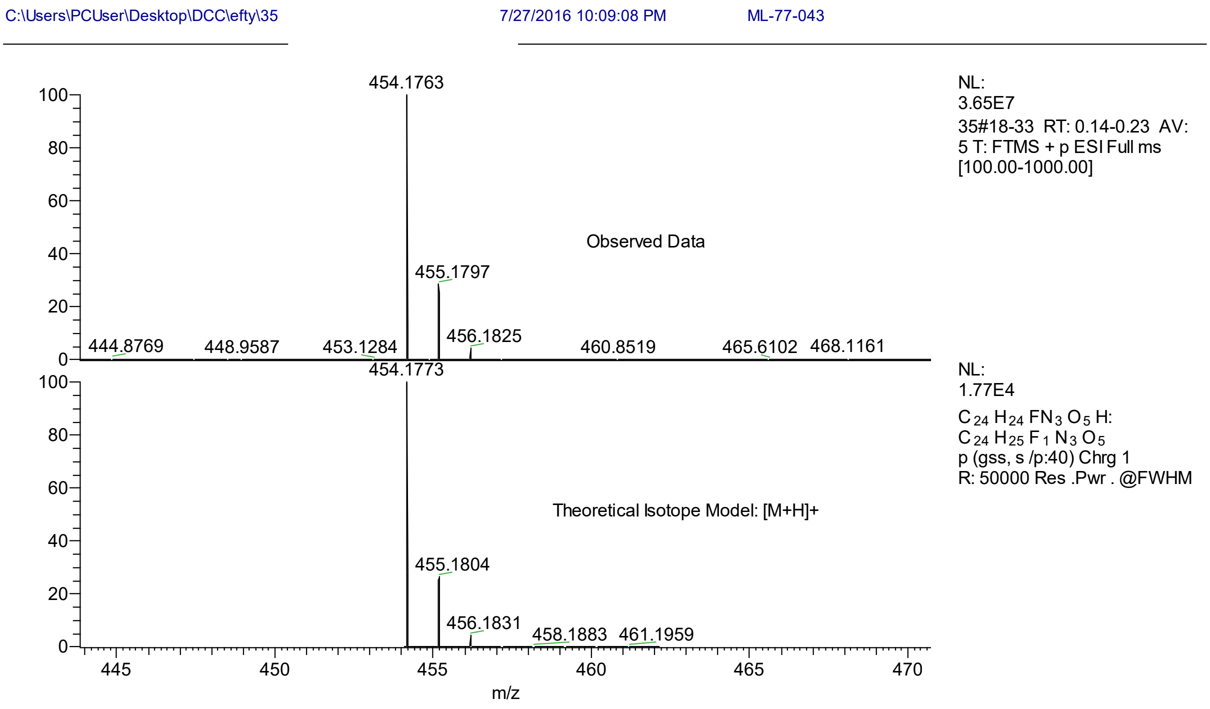

Compound 35 Salt

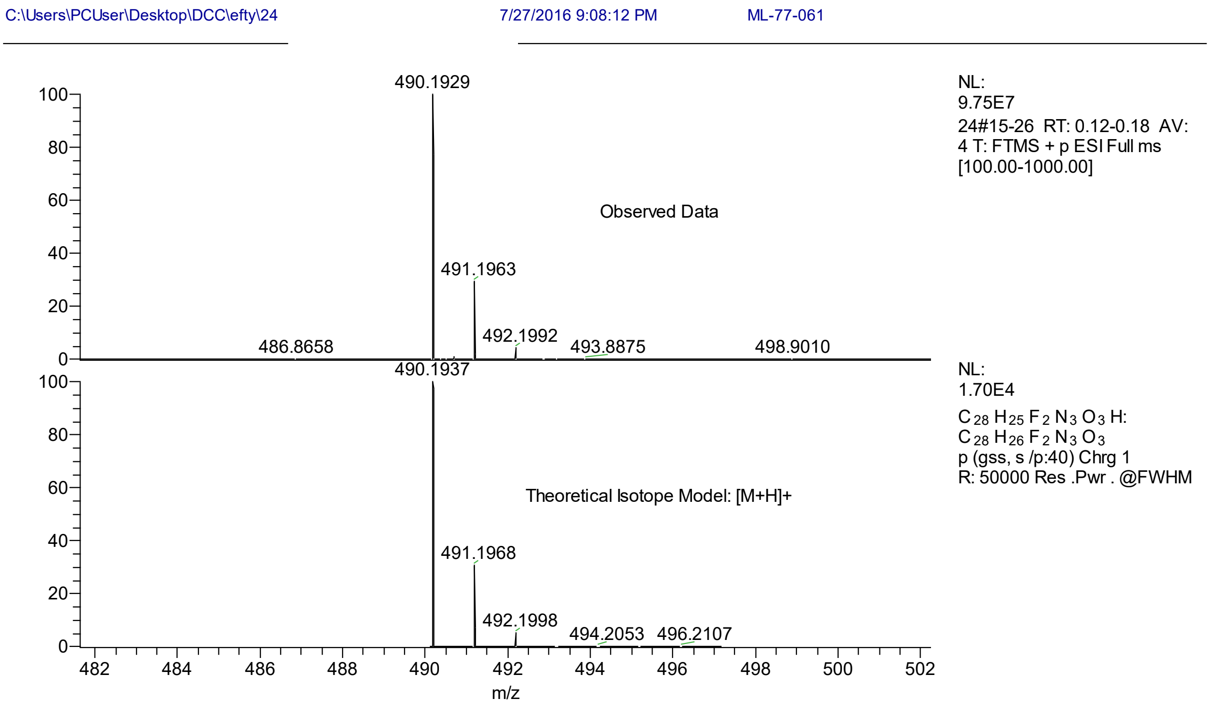

Compound 36 Salt

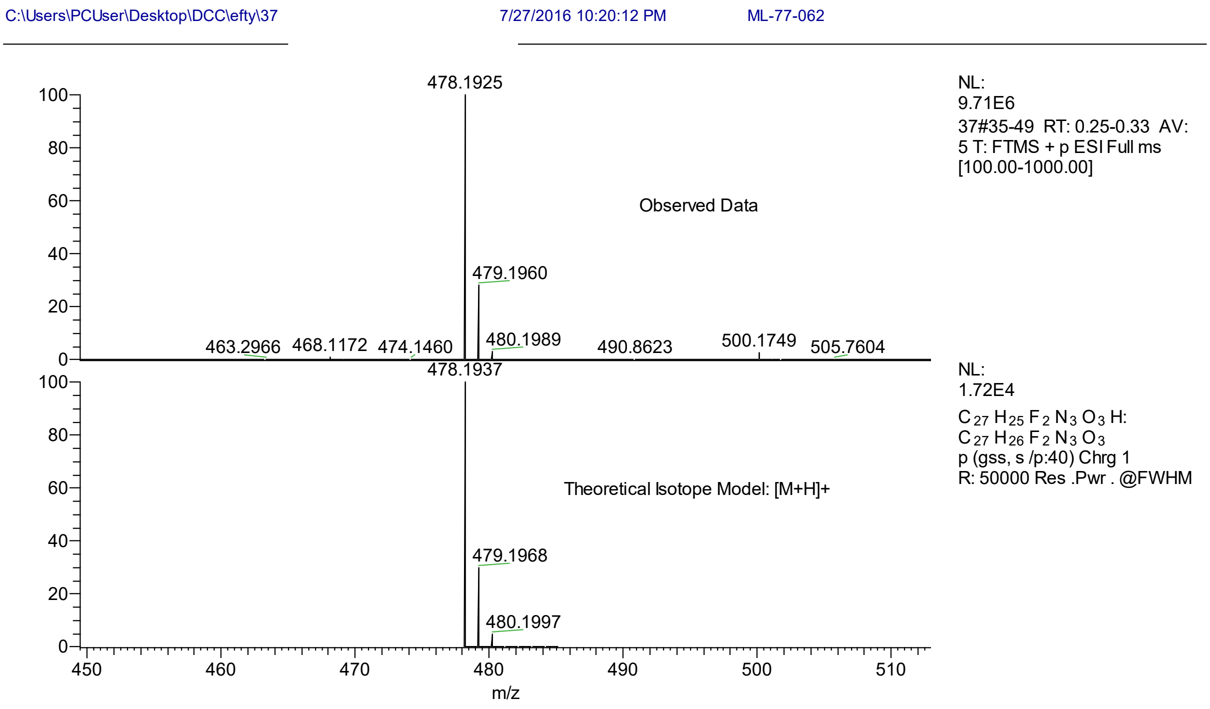

Compound 37 Salt

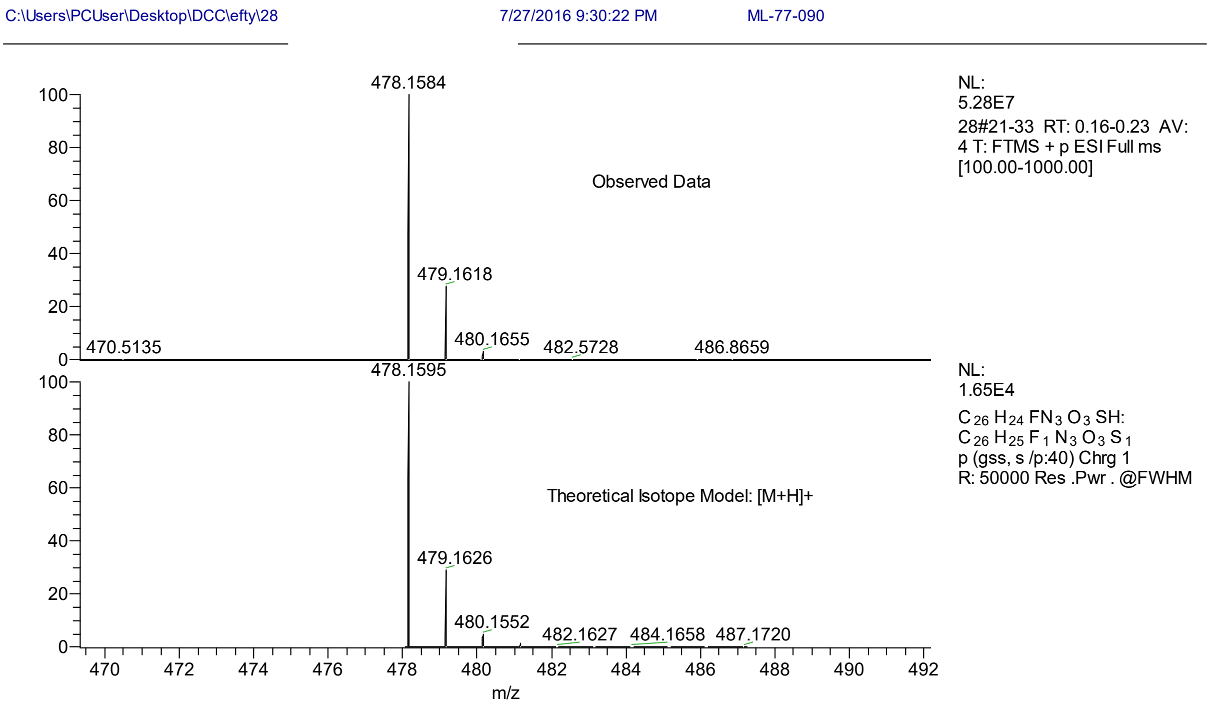

Compound 38 Salt

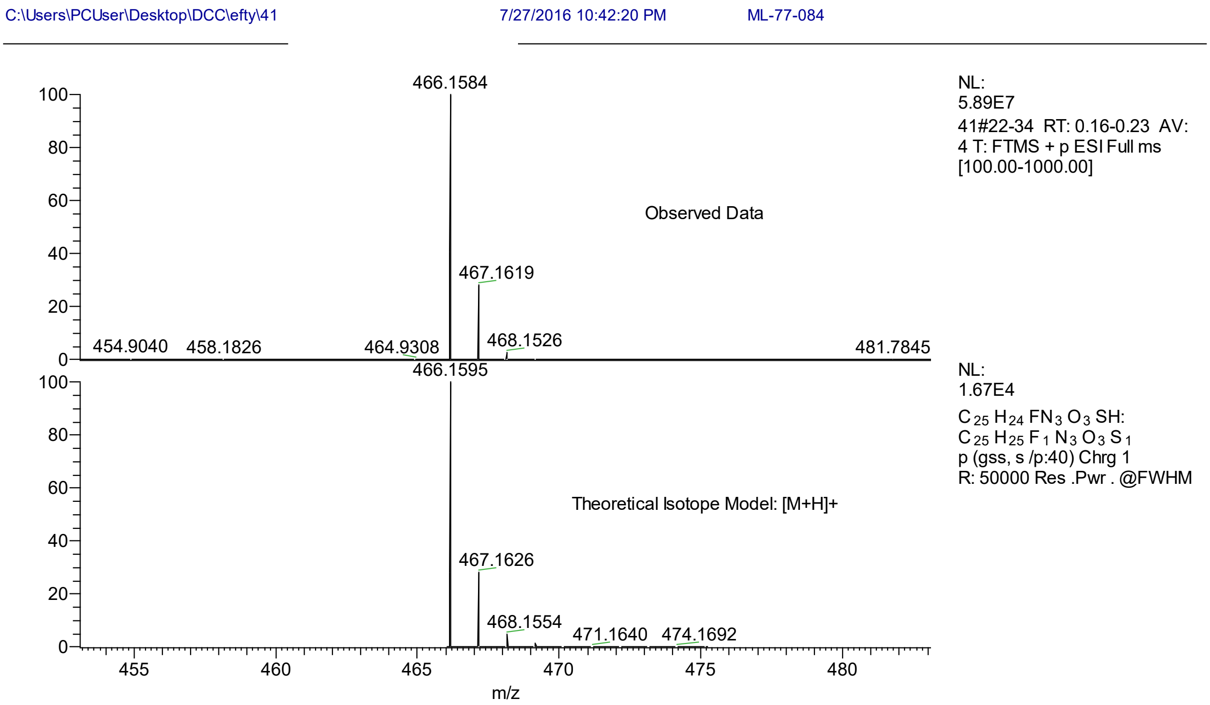

Compound 39 Salt

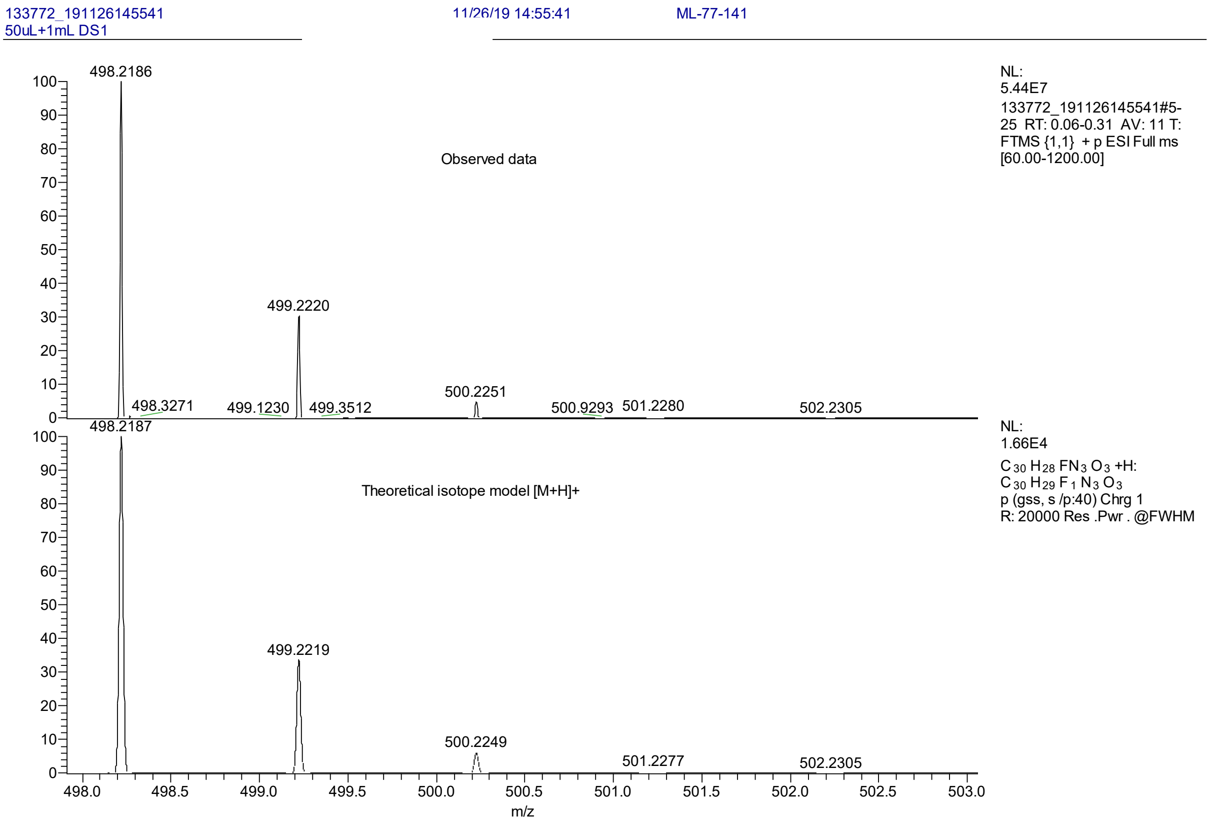

Compound 40 Salt

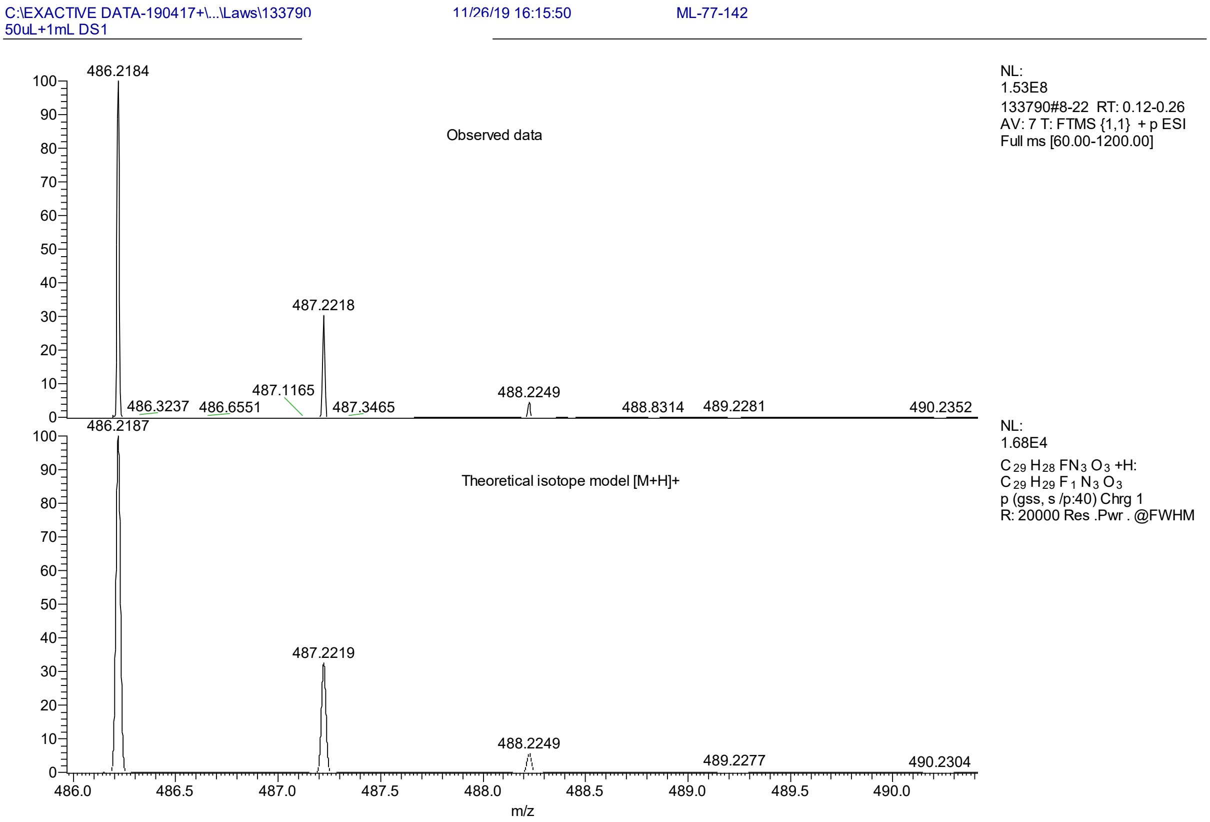

2.1.4 HRMS Spectra for Second-Generation ERB-fluoroquinolones

Compound 42

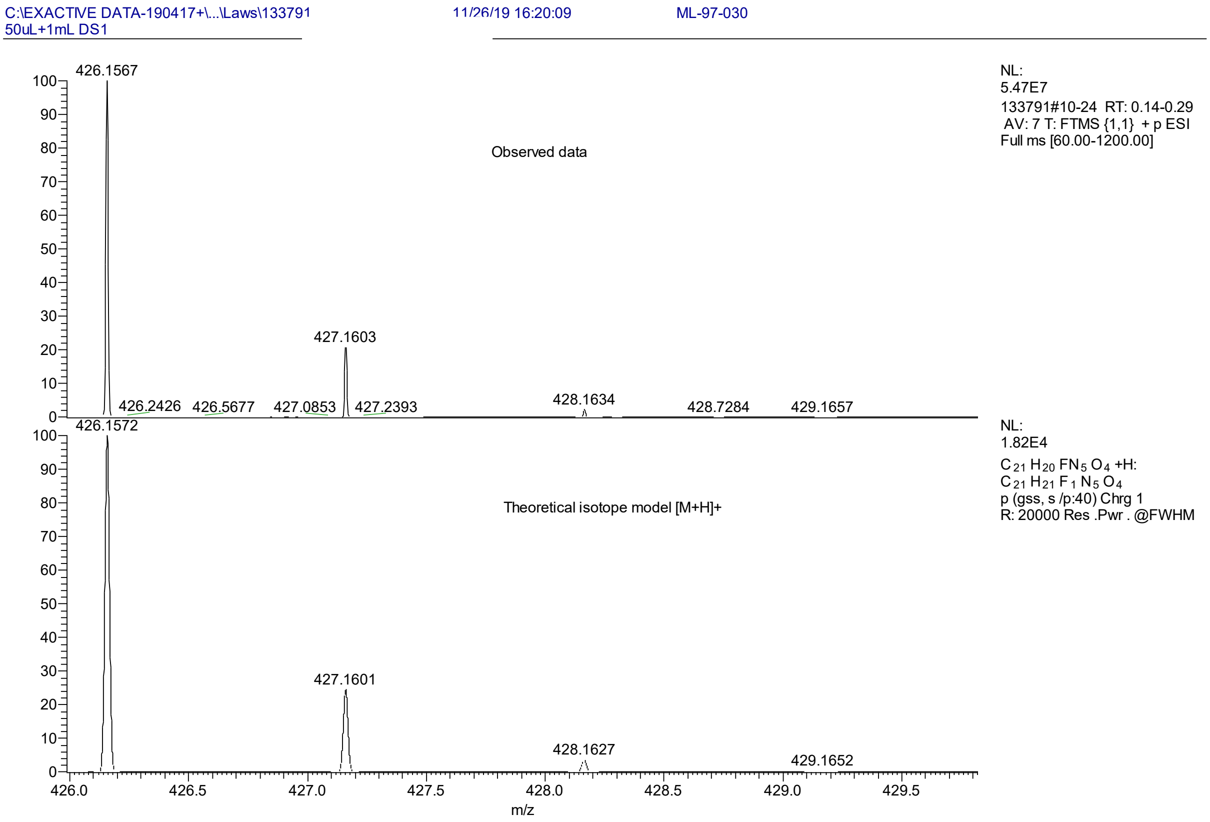

Compound 43

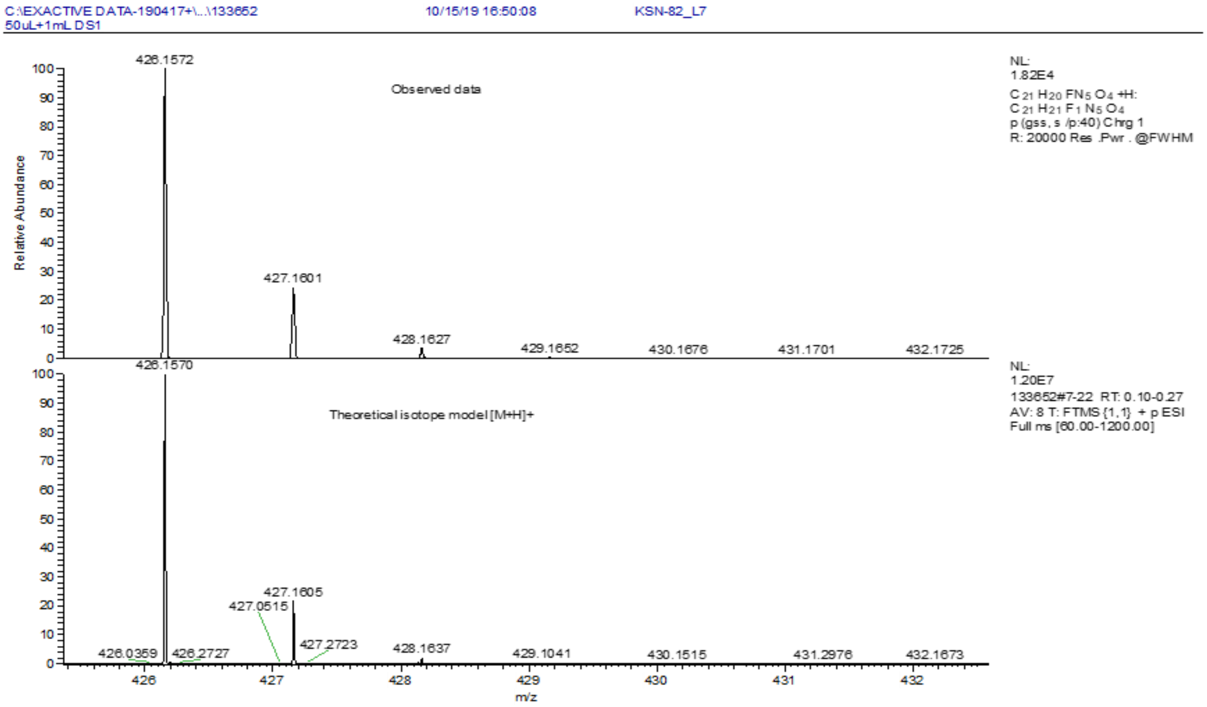
C:\EXACTIVE DATA-190417+\...\133652
50uL+1mL DS1
10/15/19 16:50:08
KSN-82_L7
Observed data
426.1572
427.1601
428.1627
429.1652
430.1676
431.1701
432.1725
NL:
1.82E4
C 21 H20 FN5 O4 +H:
C 21 H21 F1 N5 O4
p (gss, s /p:40) Chrg 1
R: 20000 Res .Pwr . @FWHM
Relative Abundance
Theoretical isotope model [M+H]+
426.1570
427.1605
427.0515
427.2723
426.0359
426.2727
428.1637
429.1041
430.1515
431.2976
432.1673
NL:
1.20E7
133652#7-22 RT: 0.10-0.27
AV: 8 T: FTMS {1,1} + p ESI
Full ms [60.00-1200.00]
m/z

Compound 44

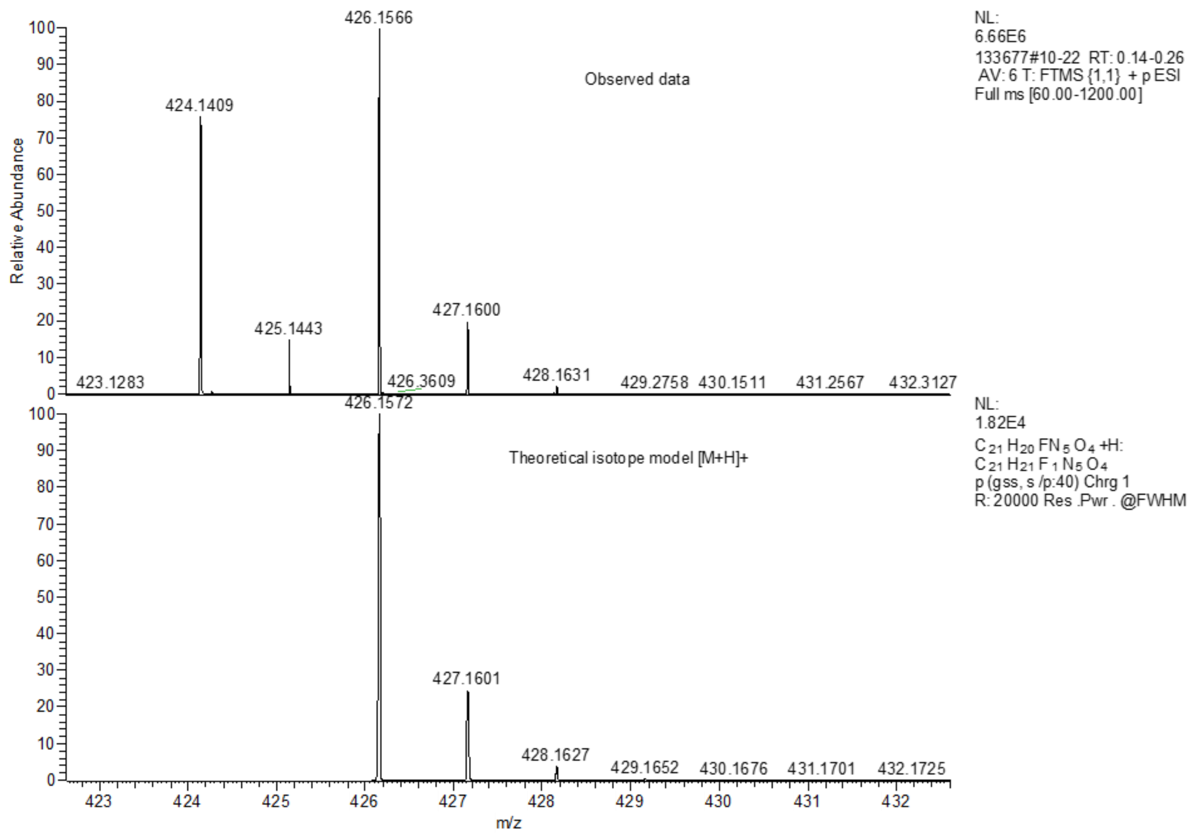

426.1566
424.1409
425.1443
427.1600
423.1283
426.3609
428.1631
429.2758
430.1511
431.2567
432.3127
Observed data
NL:
6.66E6
133677#10-22 RT: 0.14-0.26
AV: 6 T: FTMS {1,1} + p ESI
Full ms [60.00-1200.00]
Relative Abundance
426.1572
427.1601
428.1627
429.1652
430.1676
431.1701
432.1725
Theoretical isotope model [M+H]+
NL:
1.82E4
C 21 H20 FN 5 O 4 +H:
C 21 H21 F 1 N5 O 4
p (gss, s /p:40) Chrg 1
R: 20000 Res .Pwr . @FWHM
m/z

Compound 45

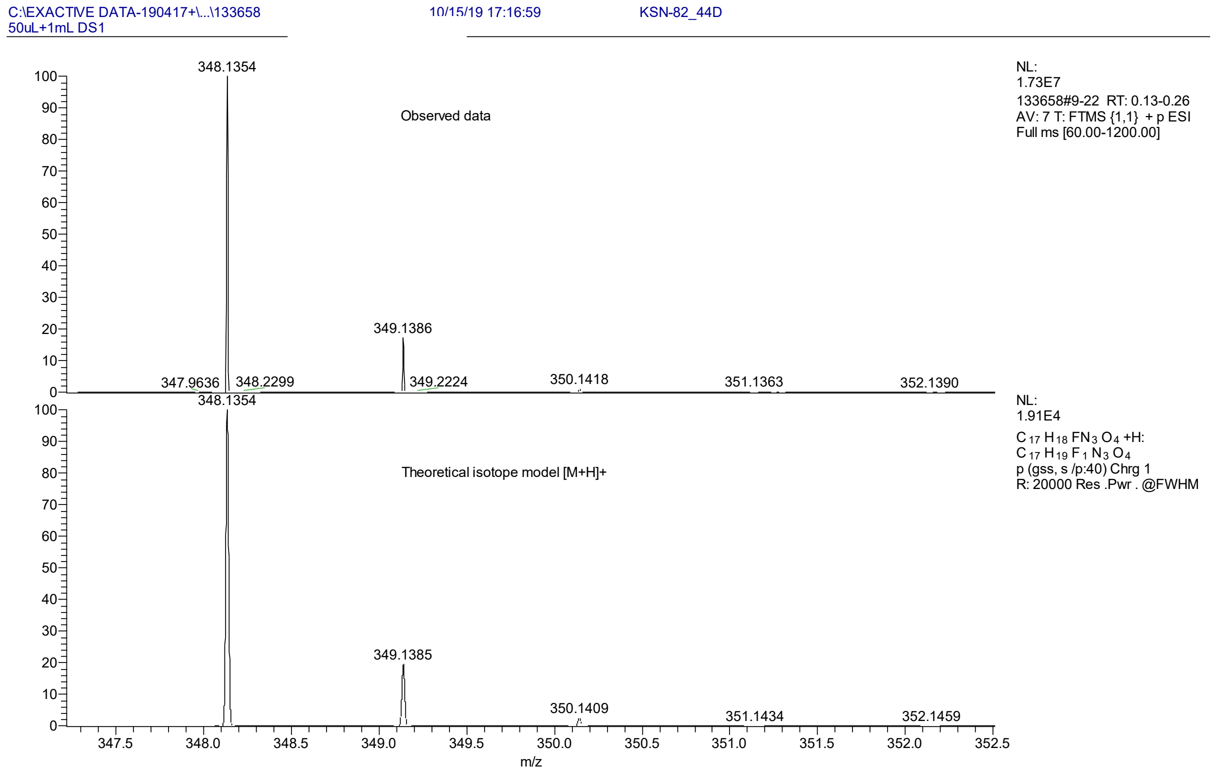

C:\EXACTIVE DATA-190417+\...\133658
50uL+1mL DS1
10/15/19 17:16:59
KSN-82_44D
348.1354
Observed data
349.1386
347.9636
348.2299
349.2224
350.1418
351.1363
352.1390
NL:
1.73E7
133658#9-22 RT: 0.13-0.26
AV: 7 T: FTMS {1,1} + p ESI
Full ms [60.00-1200.00]
348.1354
Theoretical isotope model [M+H]+
349.1385
350.1409
351.1434
352.1459
NL:
1.91E4
C 17 H 18 FN 3 O 4 +H:
C 17 H 19 F 1 N 3 O 4
p (gss, s /p:40) Chrg 1
R: 20000 Res .Pwr . @FWHM
m/z

Compound 46 (KSN-L22)

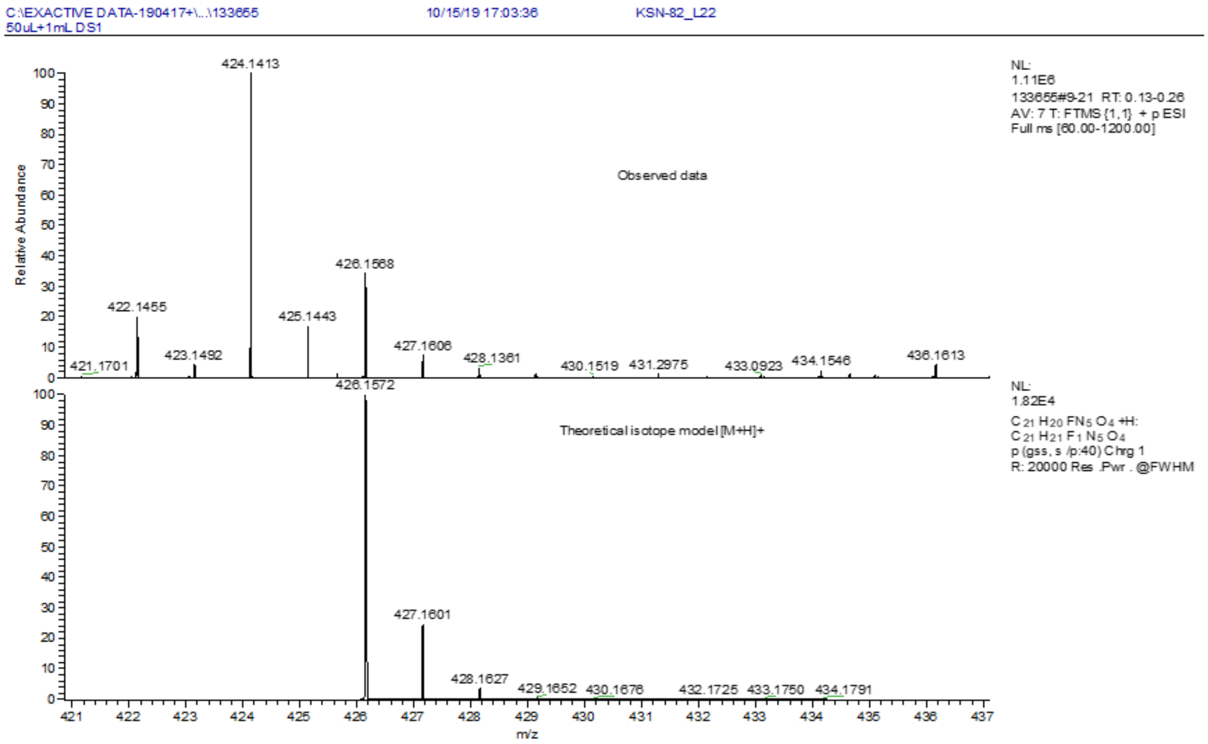

Compound 47

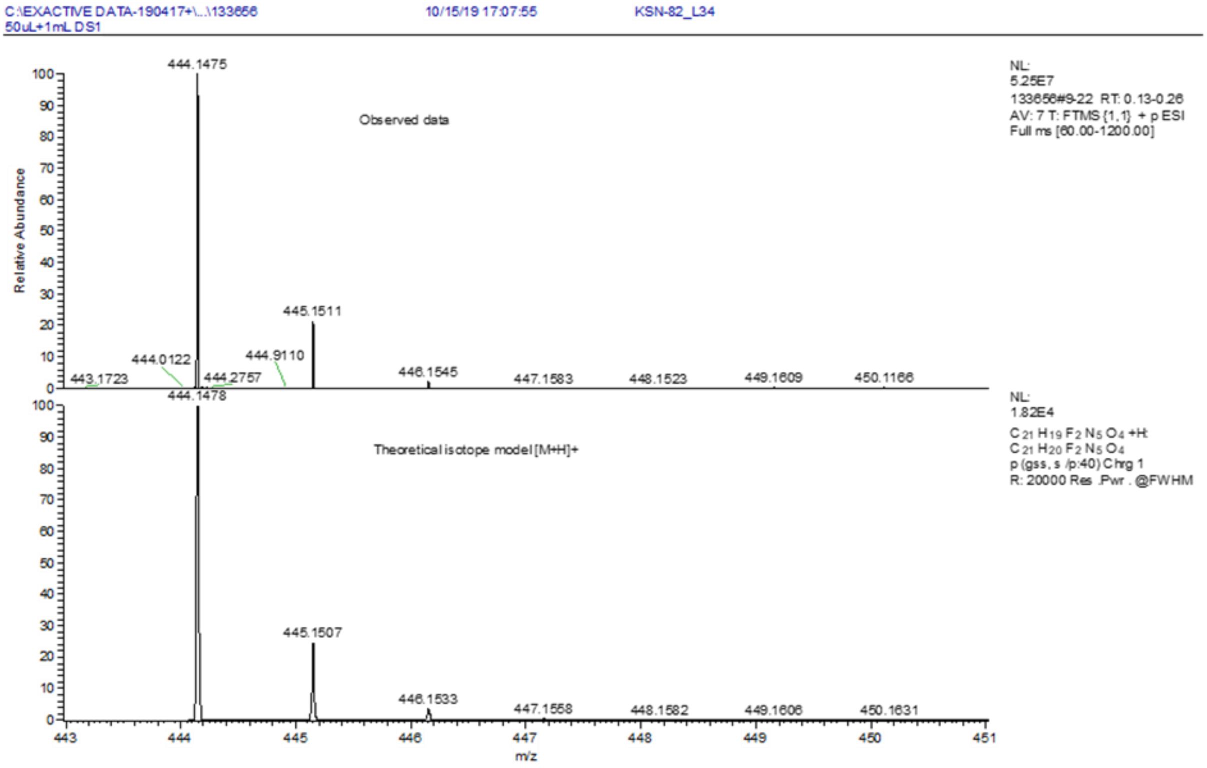

Compound 48

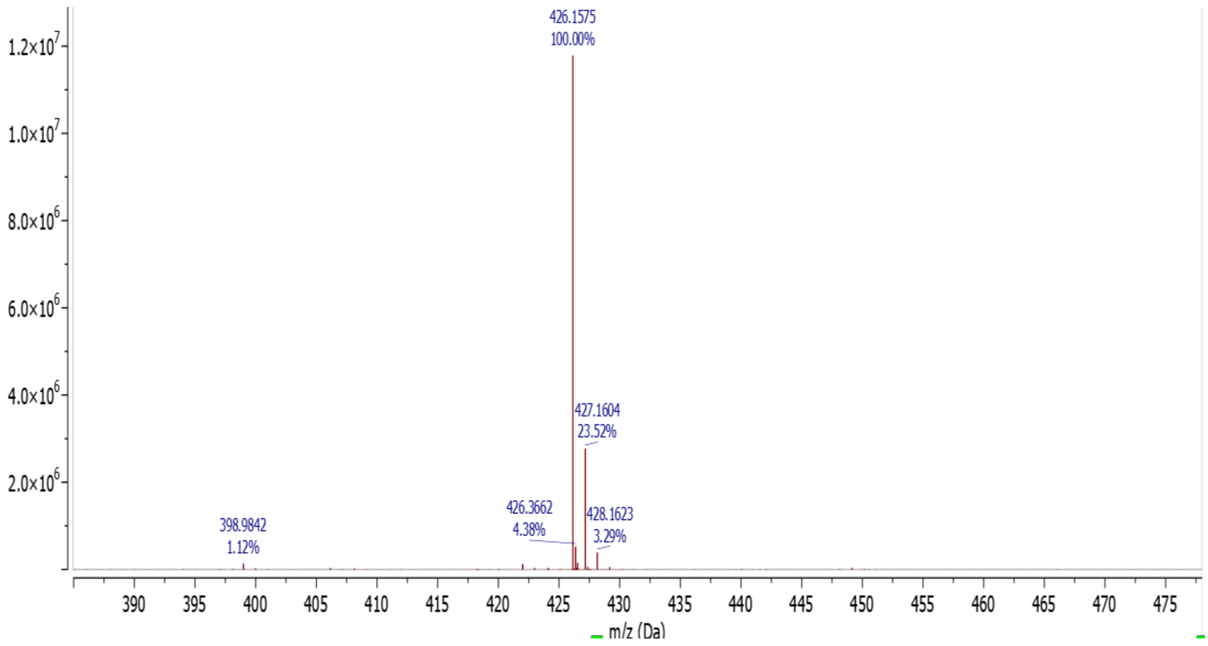

Compound 49

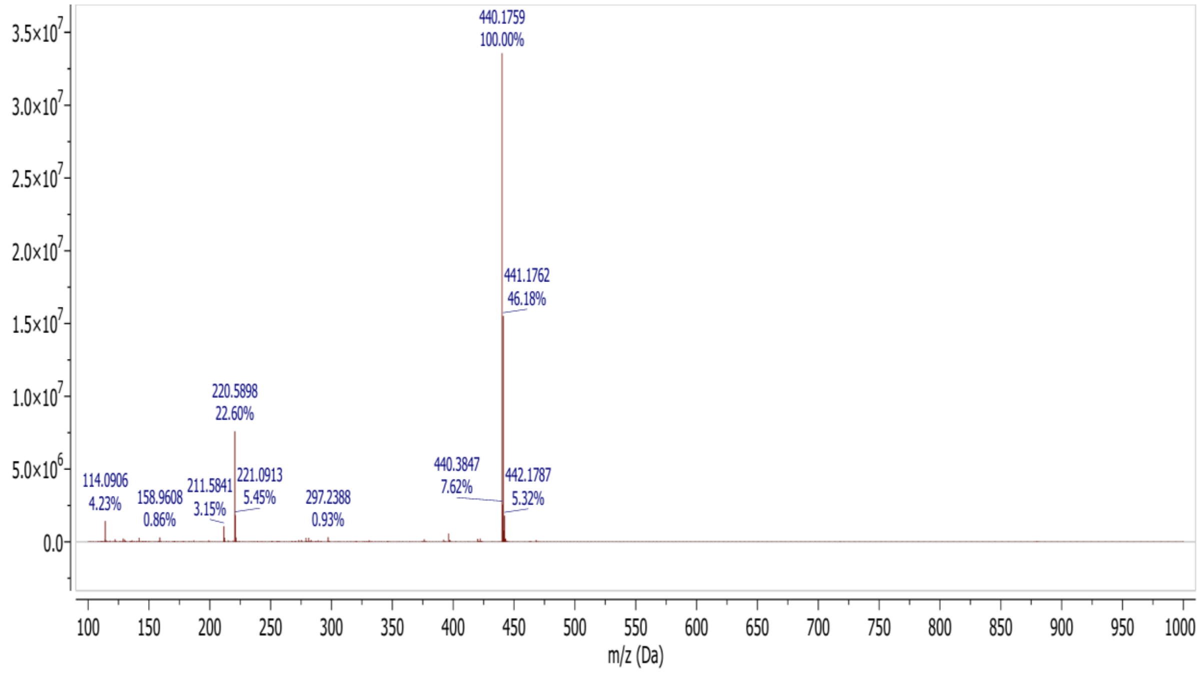
440.1759
100.00%
441.1762
46.18%
220.5898
22.60%
114.0906
4.23%
158.9608
0.86%
211.5841
3.15%
221.0913
5.45%
297.2388
0.93%
440.3847
7.62%
442.1787
5.32%
m/z (Da)

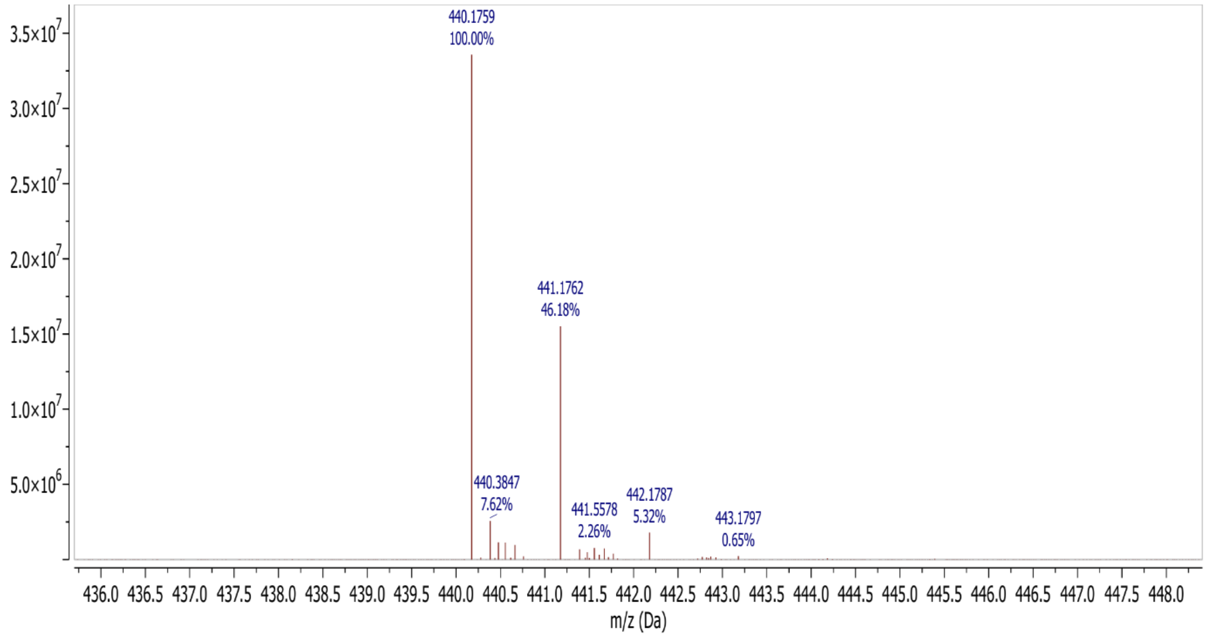
440.1759
100.00%
441.1762
46.18%
440.3847
7.62%
441.5578
2.26%
442.1787
5.32%
443.1797
0.65%
m/z (Da)

Compound 50 (BL-7)

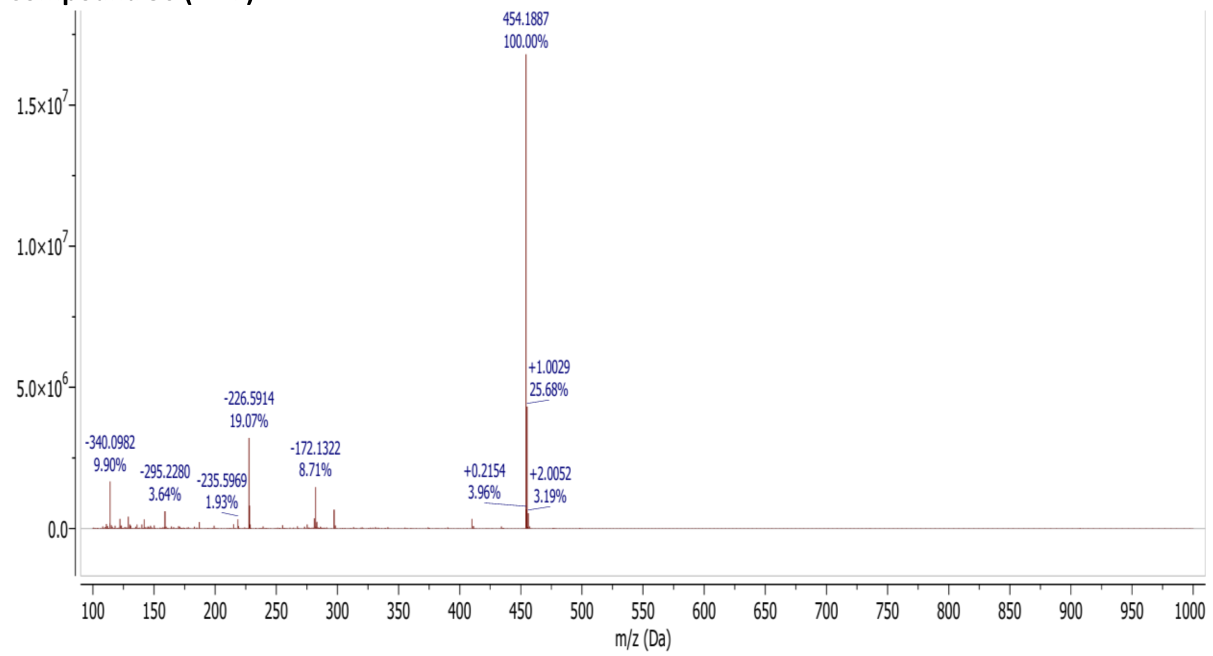

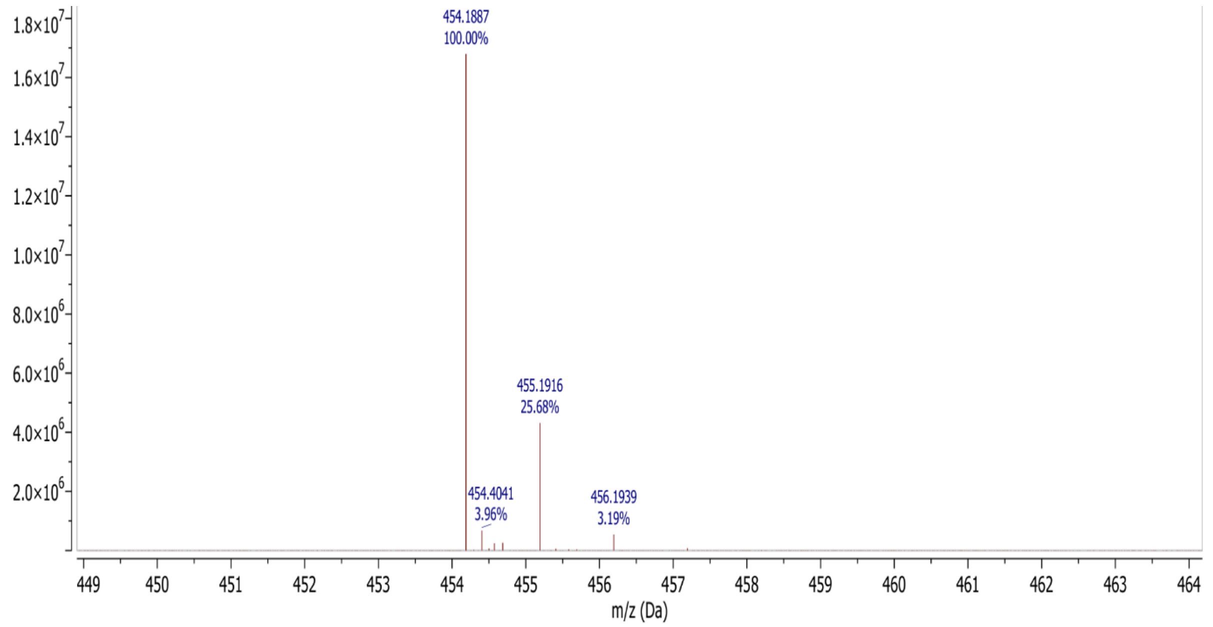

Compound 51 (ML-110-014)

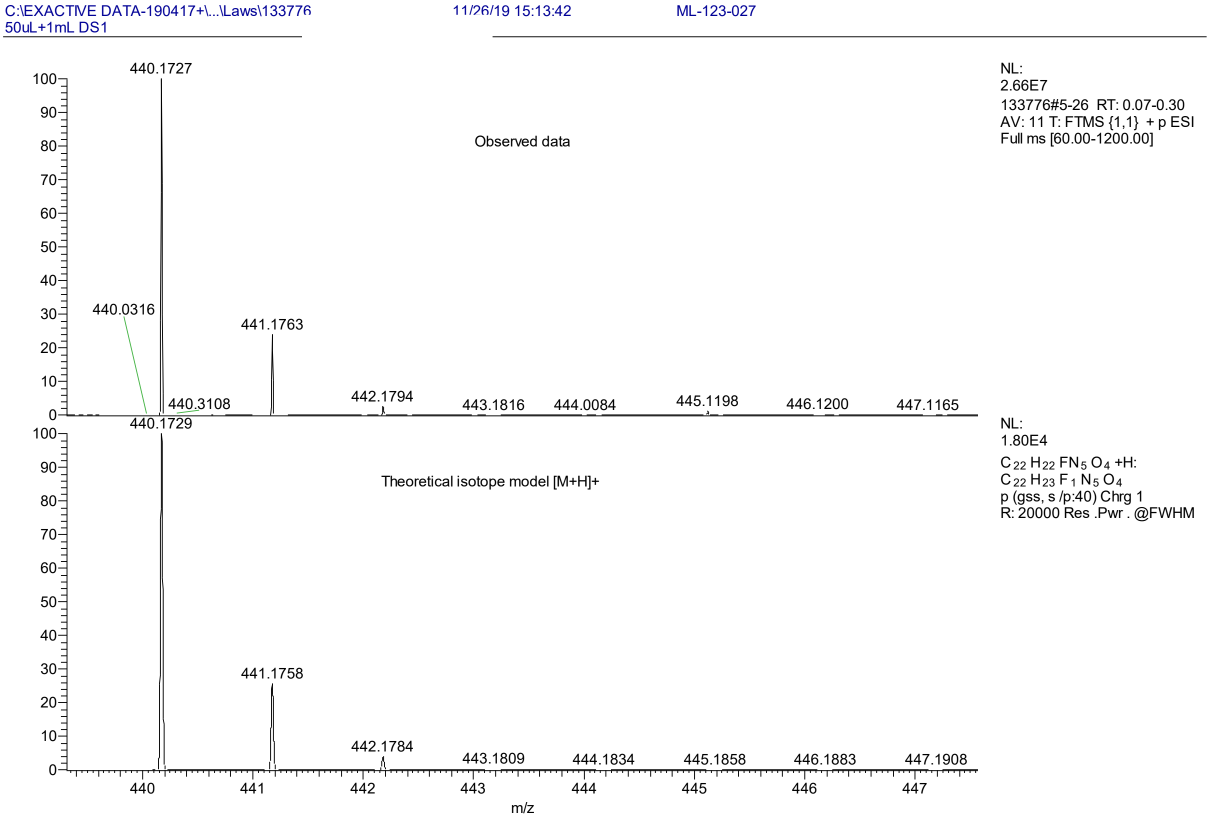

2.2 NMR Spectra

Note

Some compounds display complex and unusual splitting patterns and integrals in their associated NMR spectra due to the presence of fluorine. Fluorine-decoupled carbon-13 NMR spectra could not be generated, thus the highly fluorinated nature of the aromatic rings in both compounds causes splitting of the signals for carbons. Secondly, some of these compounds can form two distinct structural isomers due to the 3-aminoacrylate moiety. These isomers exist in equilibrium with an interconversion rate significantly less than the difference in frequency between the isomers ('slow on the NMR timescale'), meaning that both isomers are fully resolved in ^{1}H and ^{13}C NMR spectra. In the proton NMR spectrum of each compound, the non-integer integrals observed thus reflect the average percentage of the population of molecules adopting each of the isomers, since the integrals of each peak pair for a given proton environment add to the expected integer value.

2.2.1 ^{1}H, ^{13}C and ^{19}F NMR Spectra for Levofloxacin and Precursors

Compound 4– ^{1}H spectrum

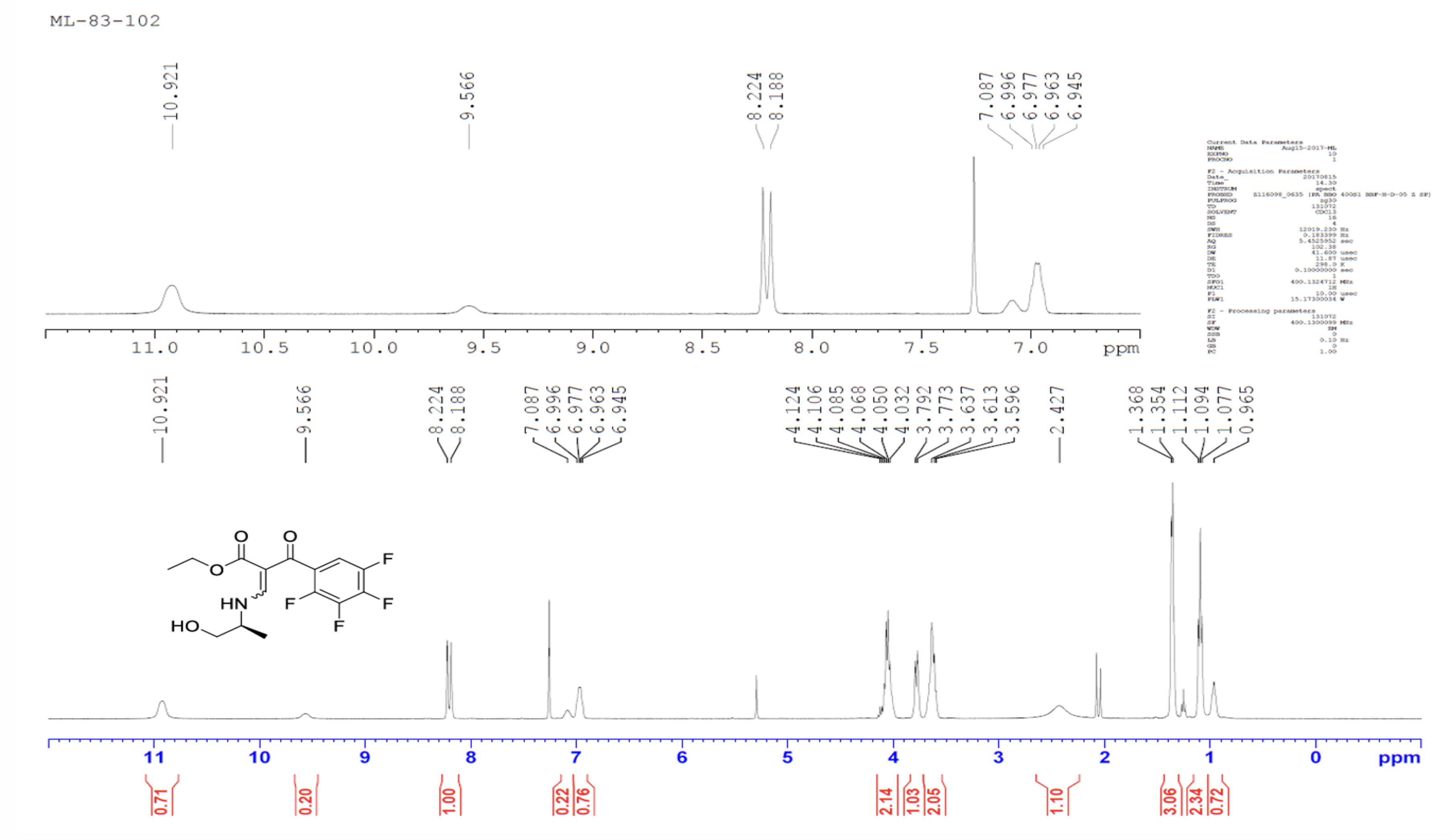

Compound 4– ^{13}C spectrum

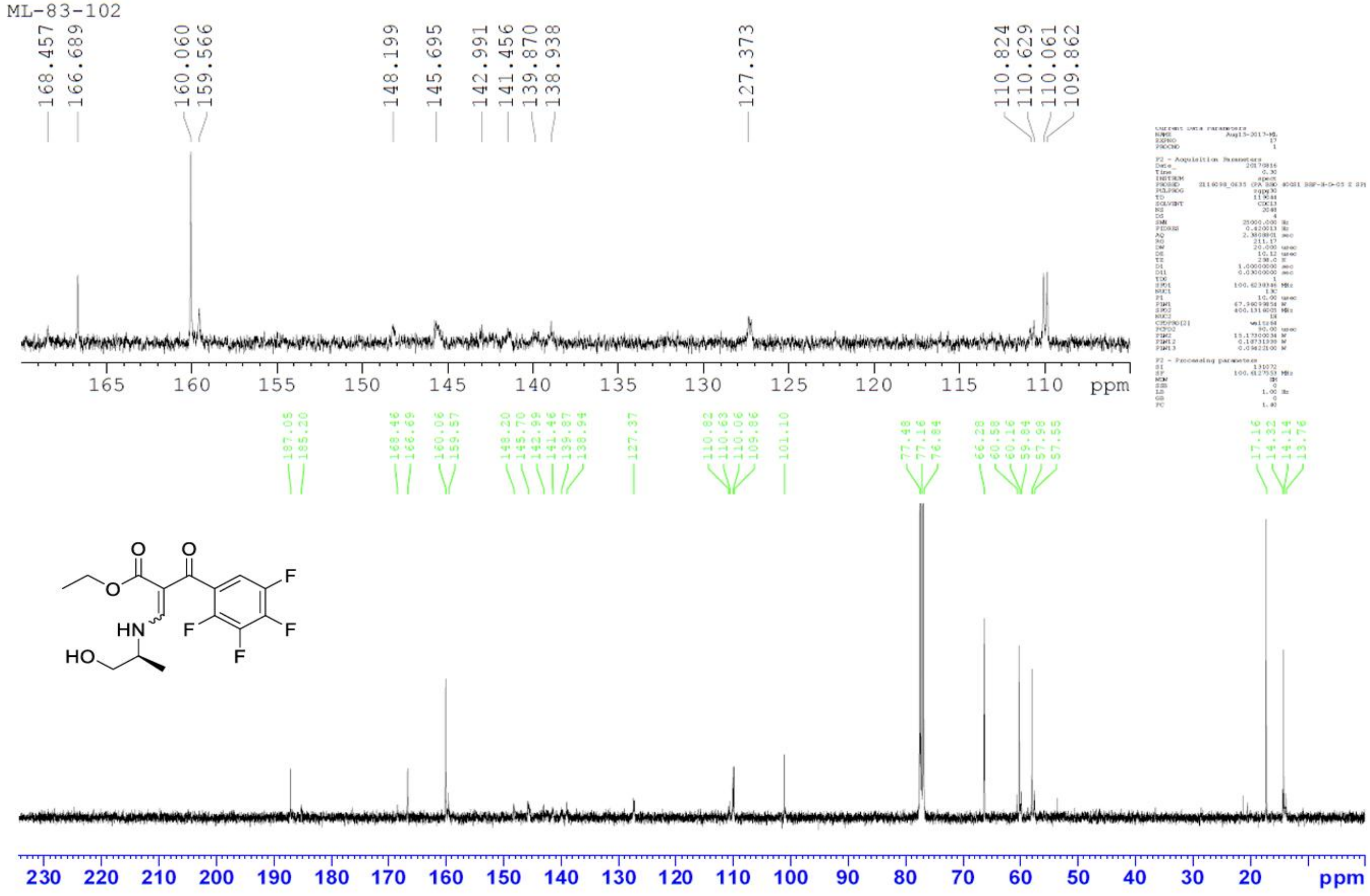

Compound 5– ^{1}H spectrum

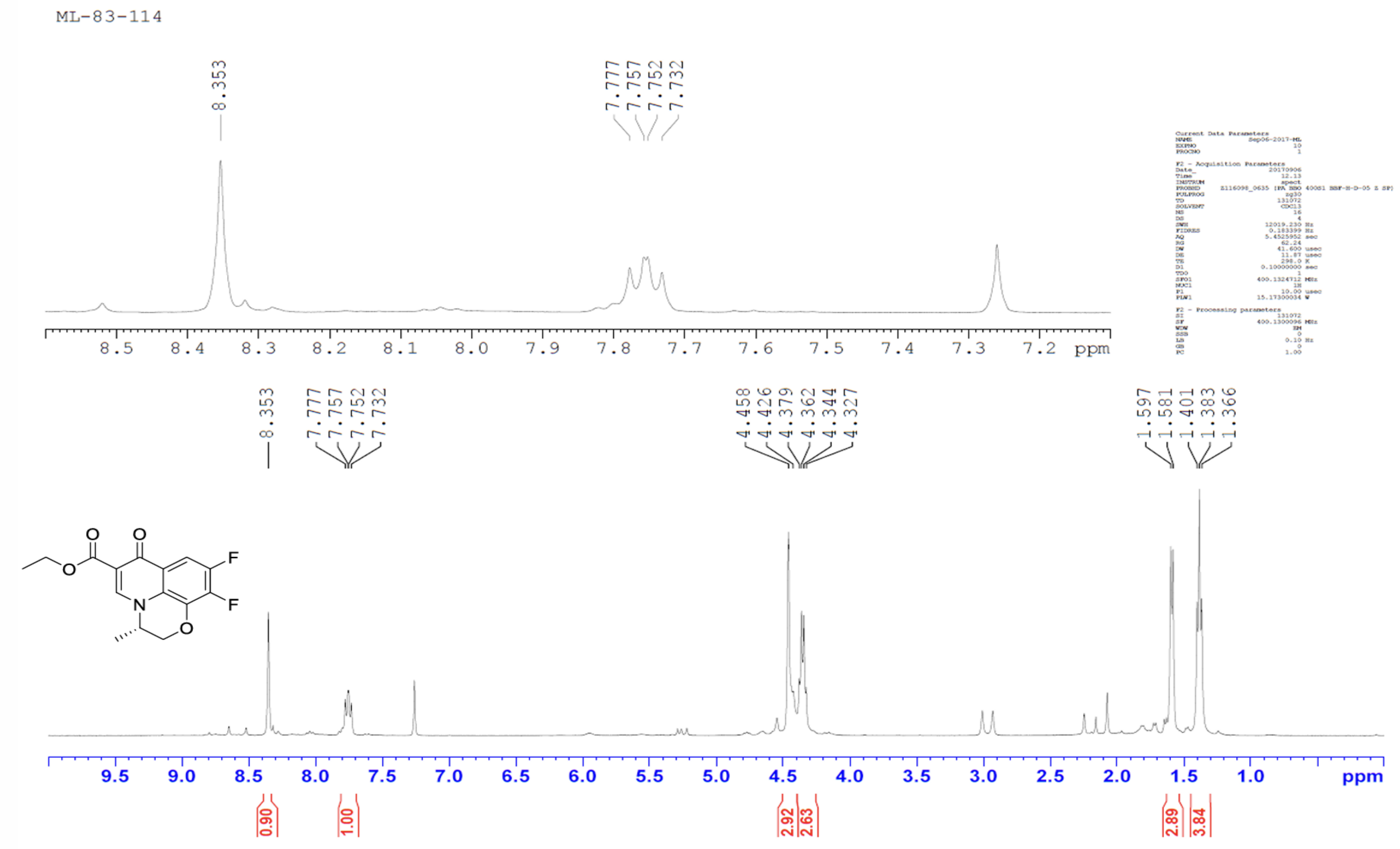

Compound 5– ^{13}C spectrum

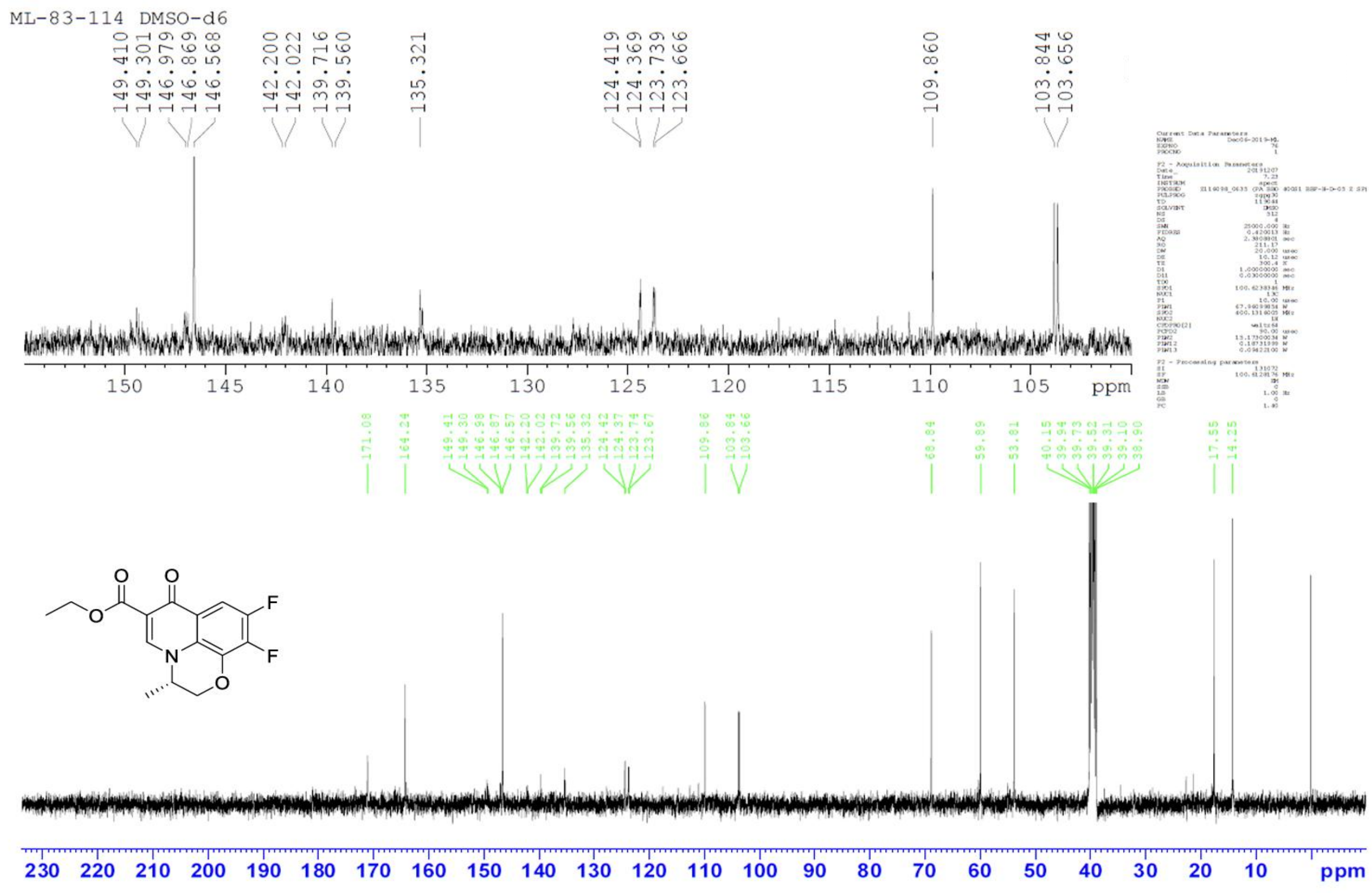

Compound 5– ^{19}F spectrum

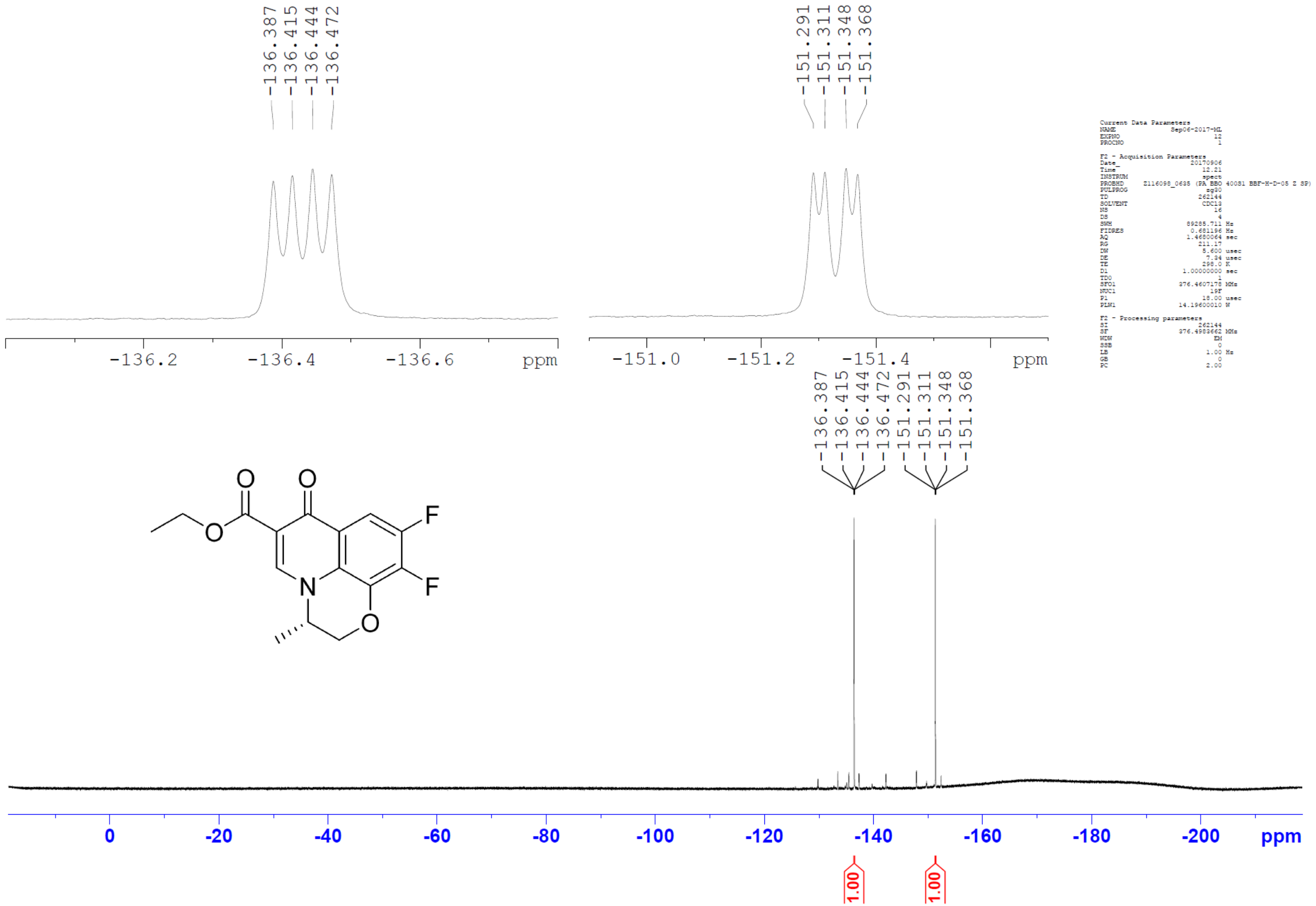

Compound 5– proton-decoupled ^{19}F spectrum

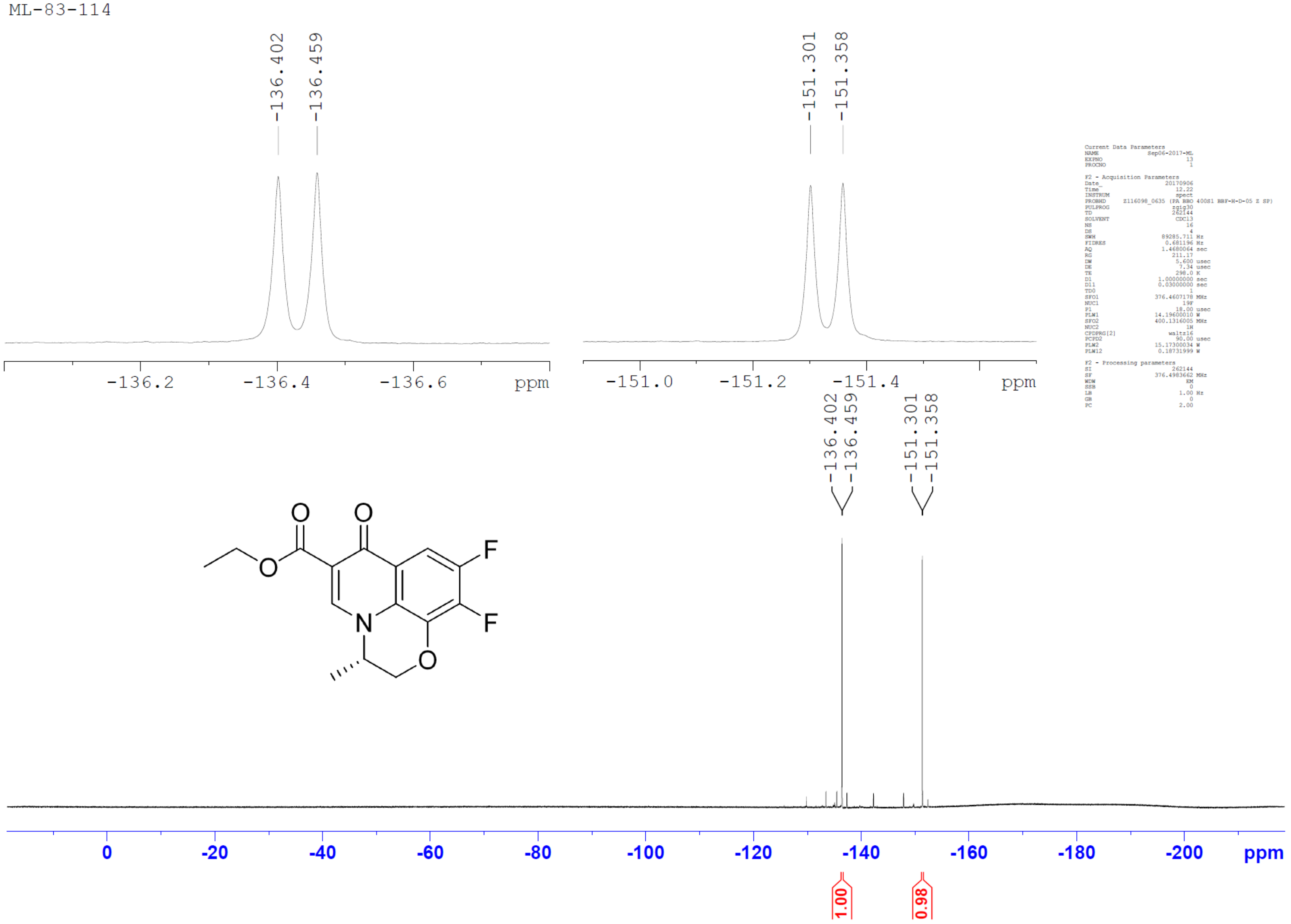

Compound 6 – ^{1}H spectrum

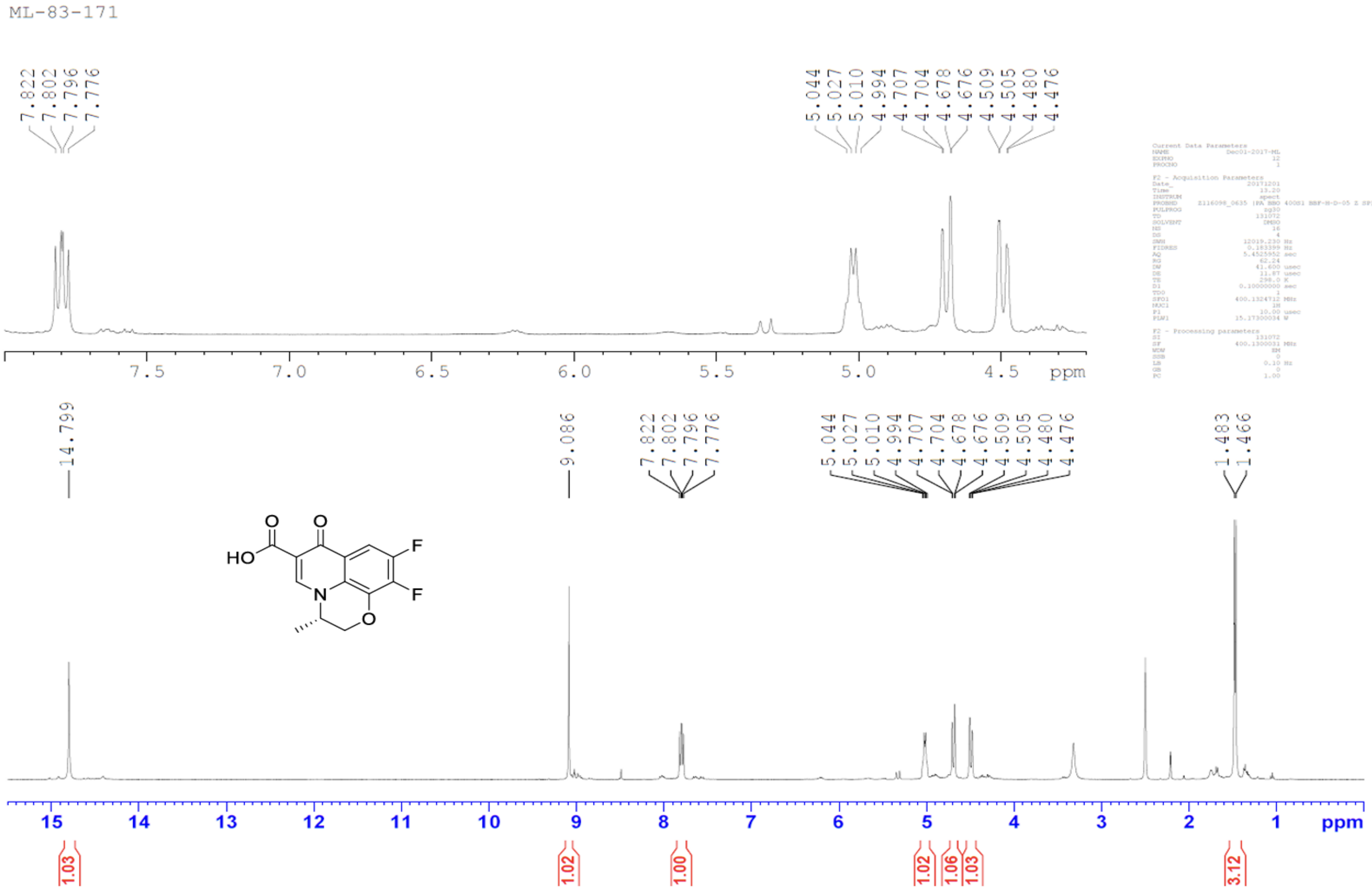

Compound 6– ^{13}C spectrum

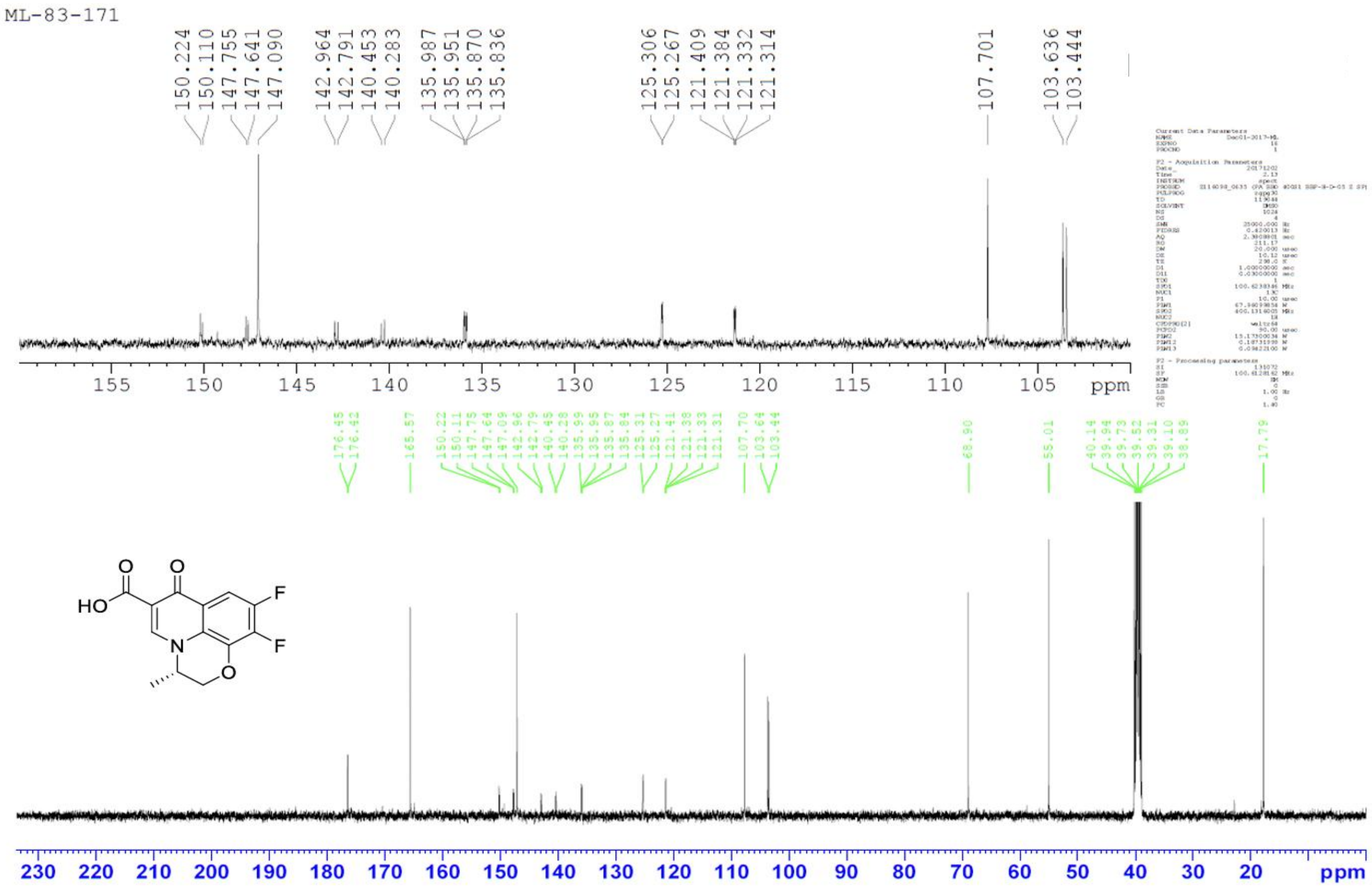

Compound 6– ^{19}F spectrum

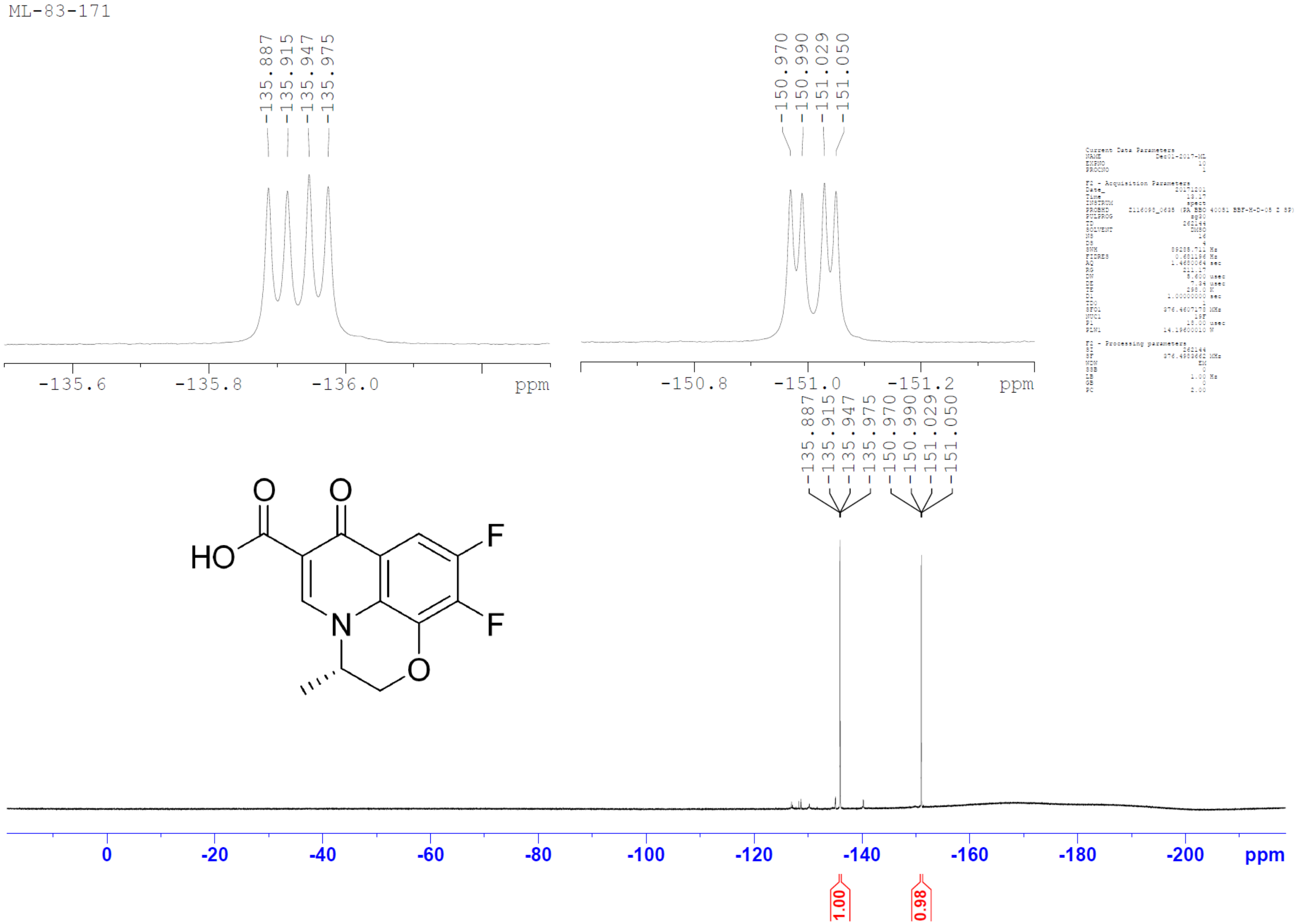

Compound 6– proton-decoupled ^{19}F spectrum

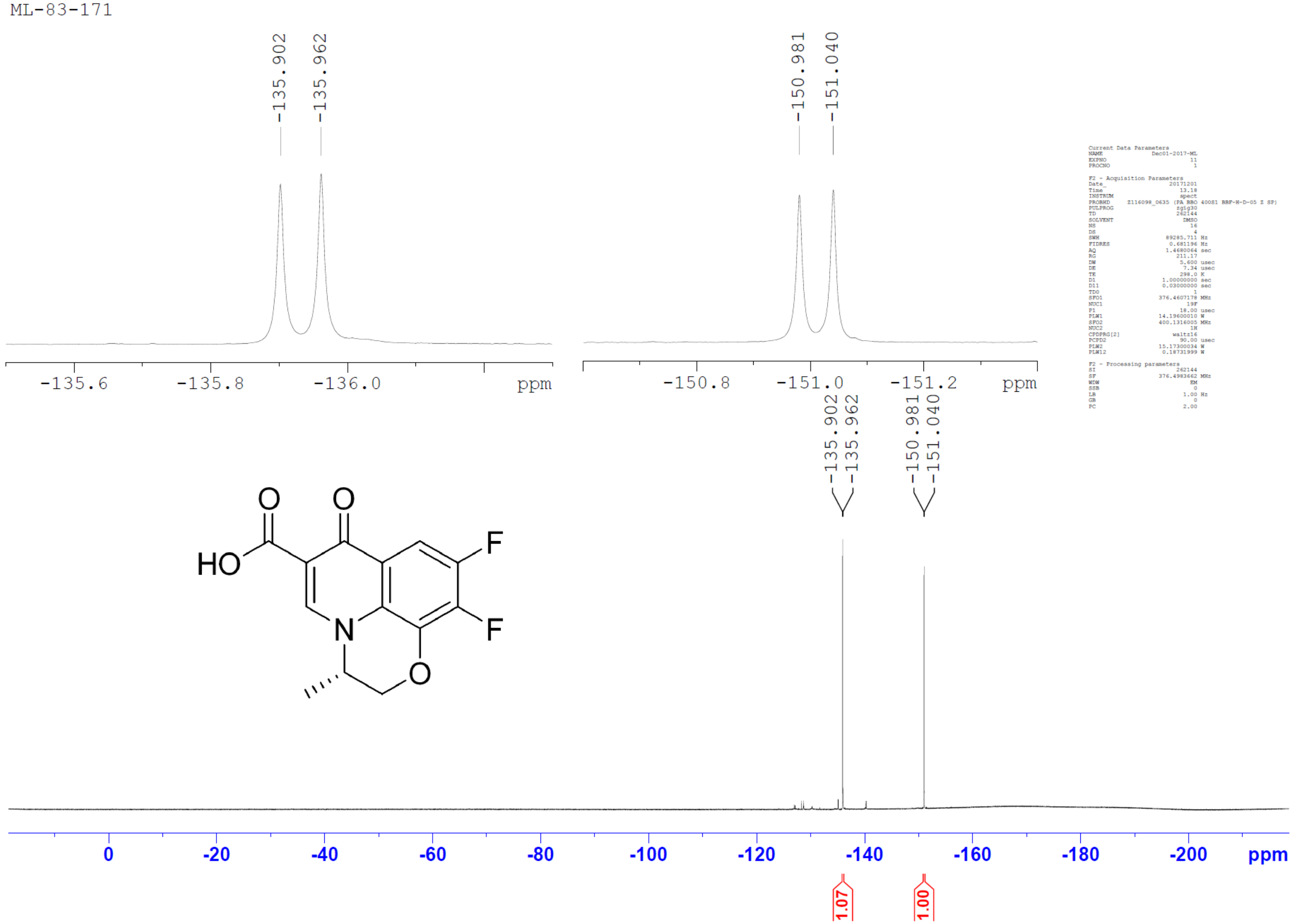

Compound 7– ^{1}H spectrum

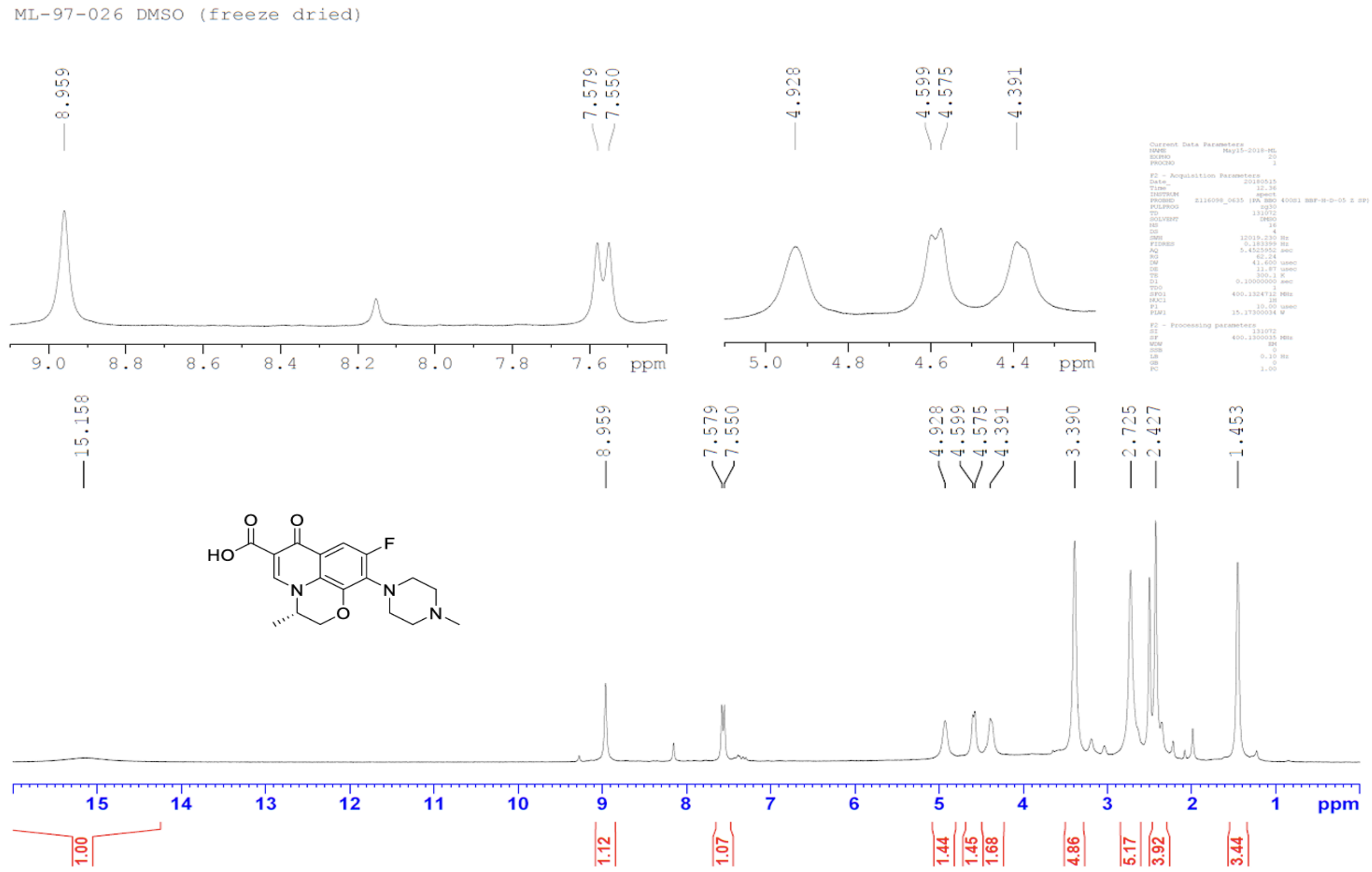

Compound 7– ^{13}C spectrum

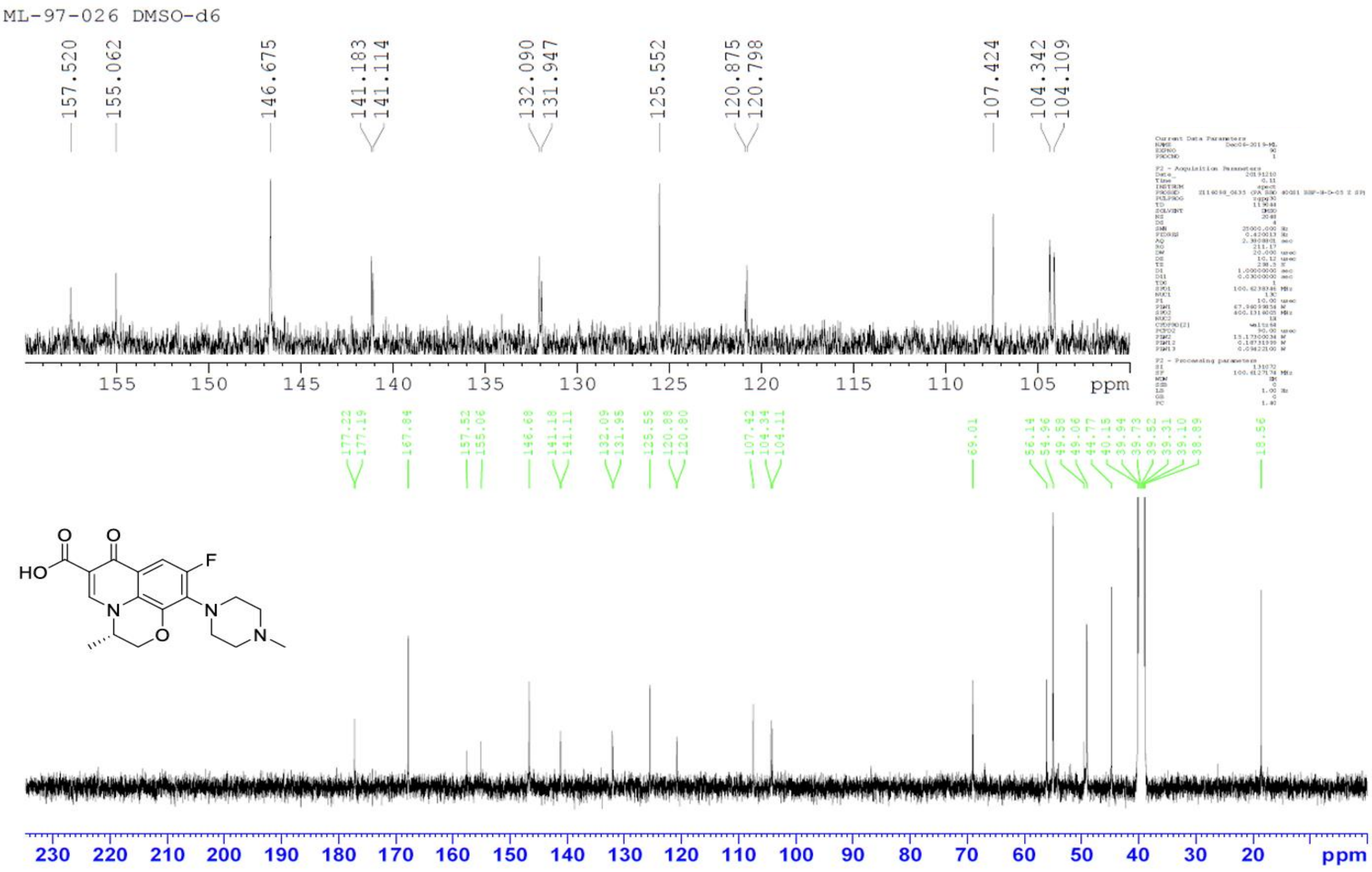

Compound 7– ^{19}F spectrum

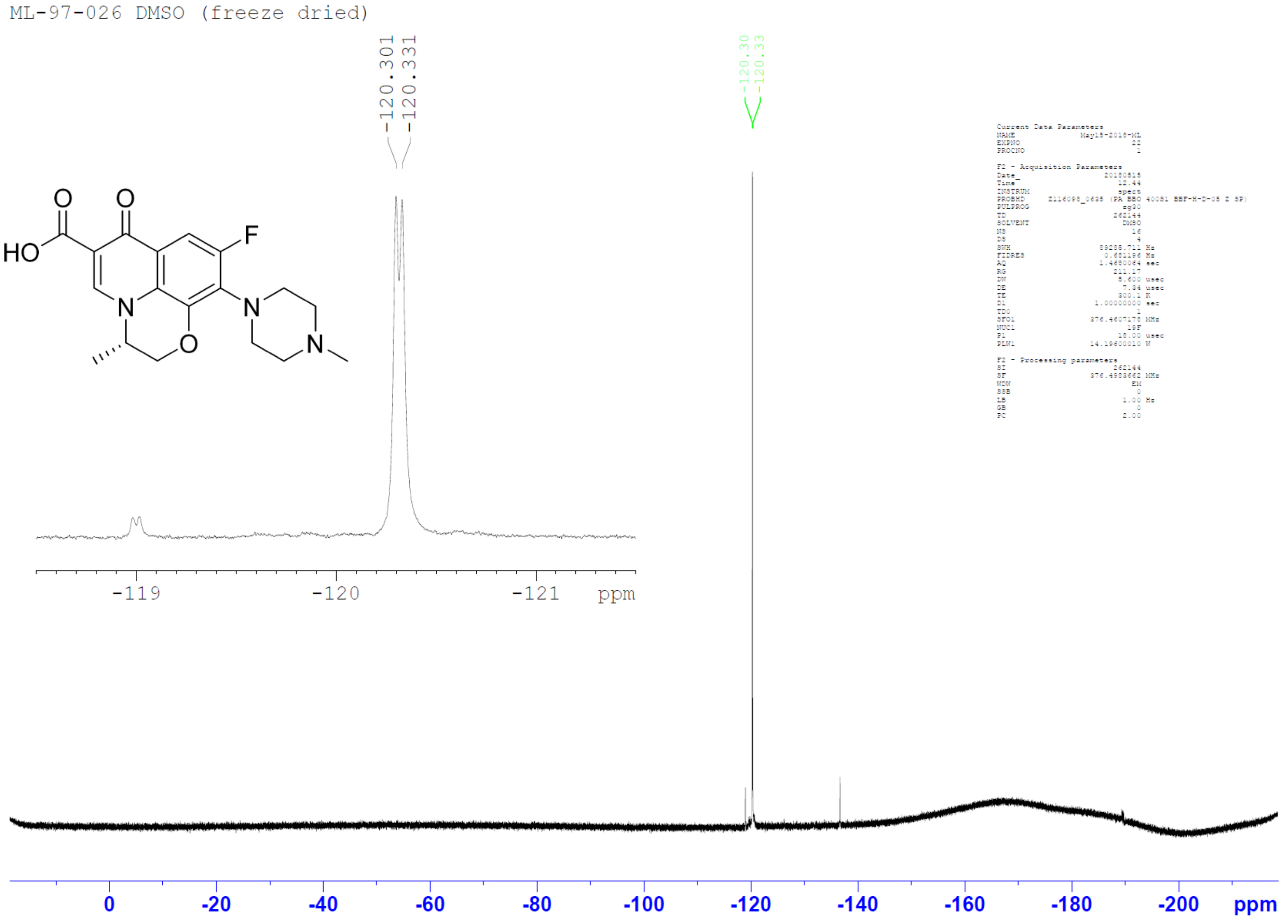

Compound 7– proton-decoupled ^{19}F spectrum

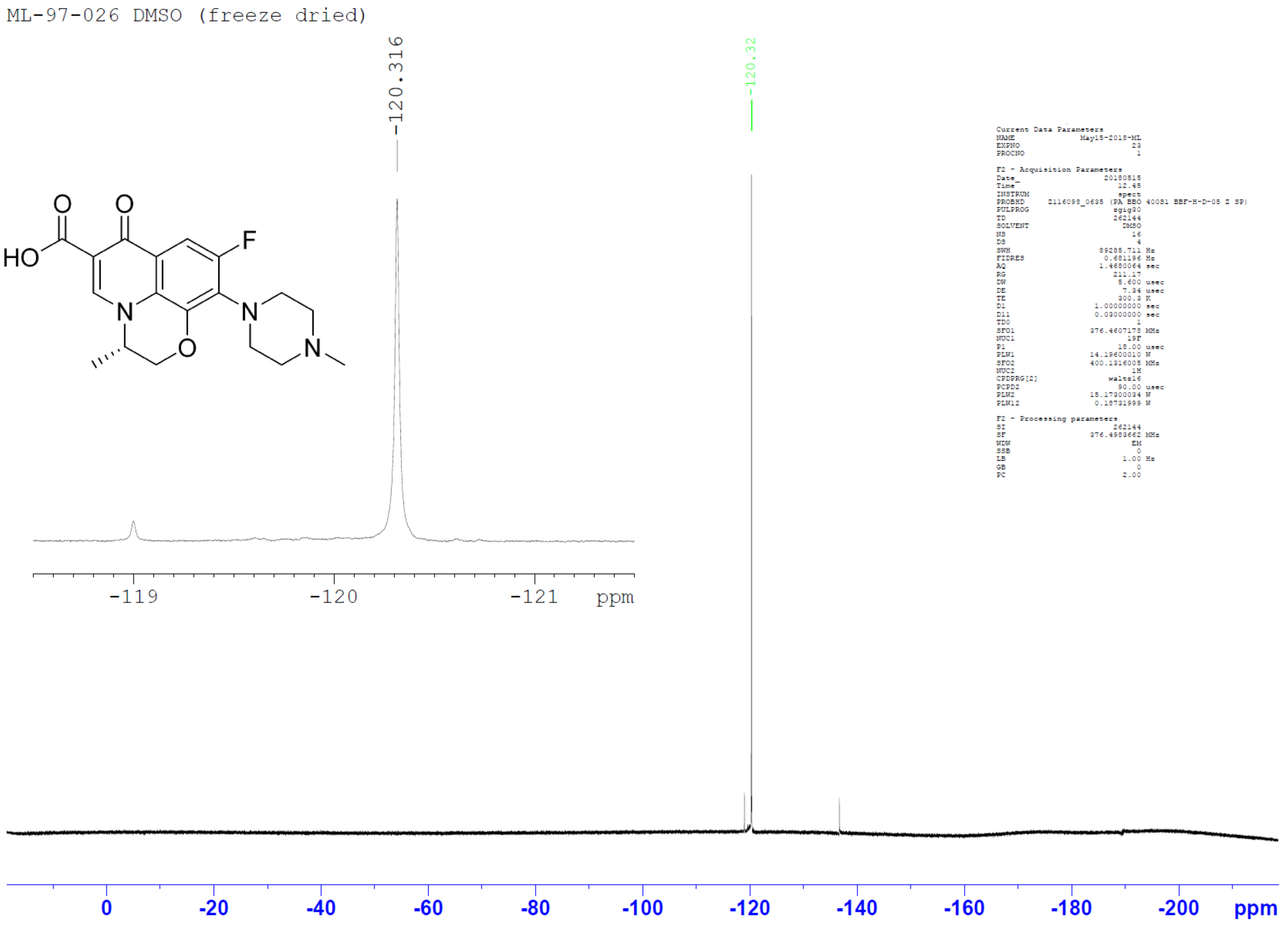

2.2.2 ^{1}H, ^{13}C and ^{19}F NMR Spectra for ML-77-005 Regioisomers and Precursors

Compound 8 (ML-77-005) (batch ML-77-022) – ^{1}H spectrum

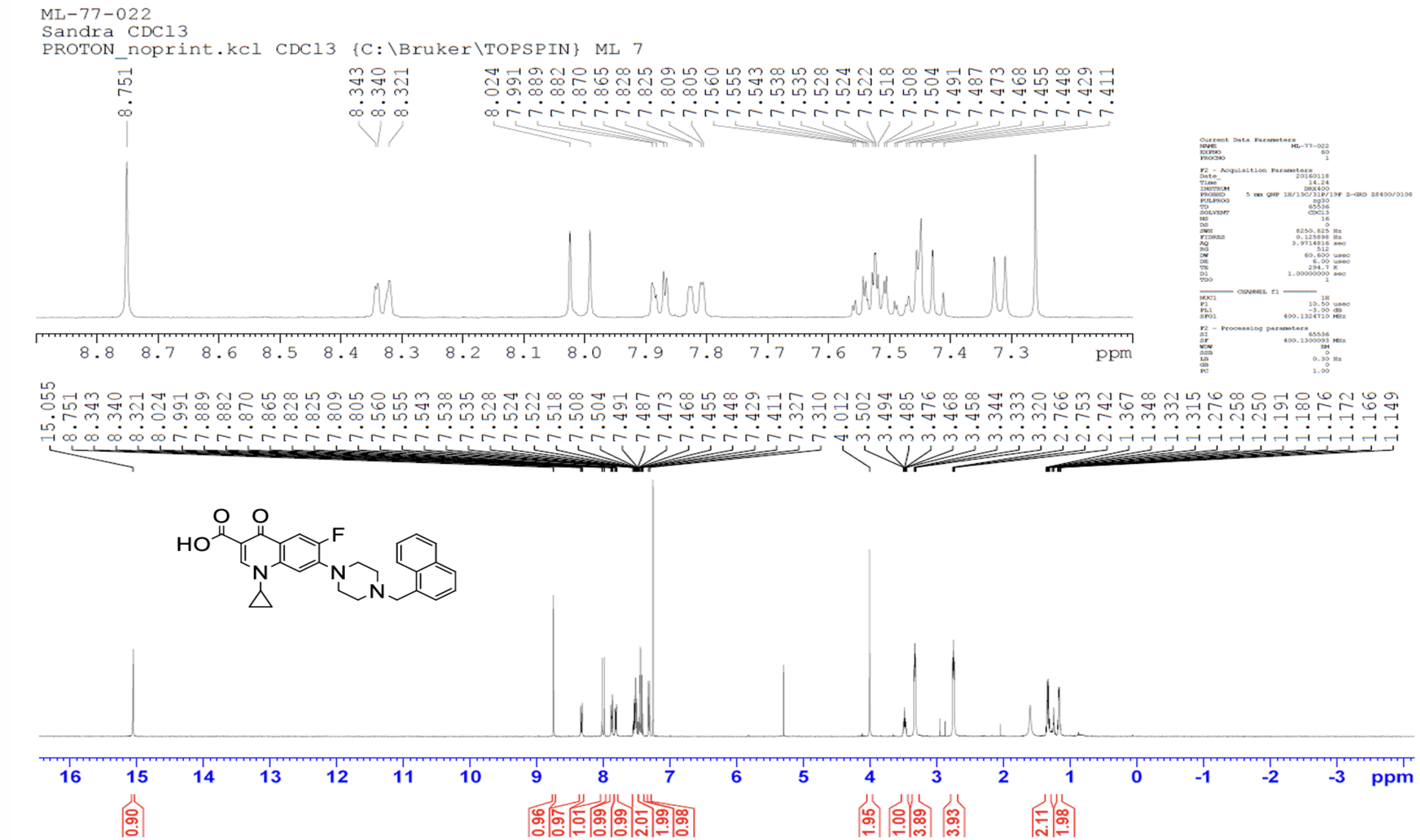

Compound 8 (ML-77-005) (batch ML-77-022 as HCl salt ML-77-023) – ^{13}C spectrum

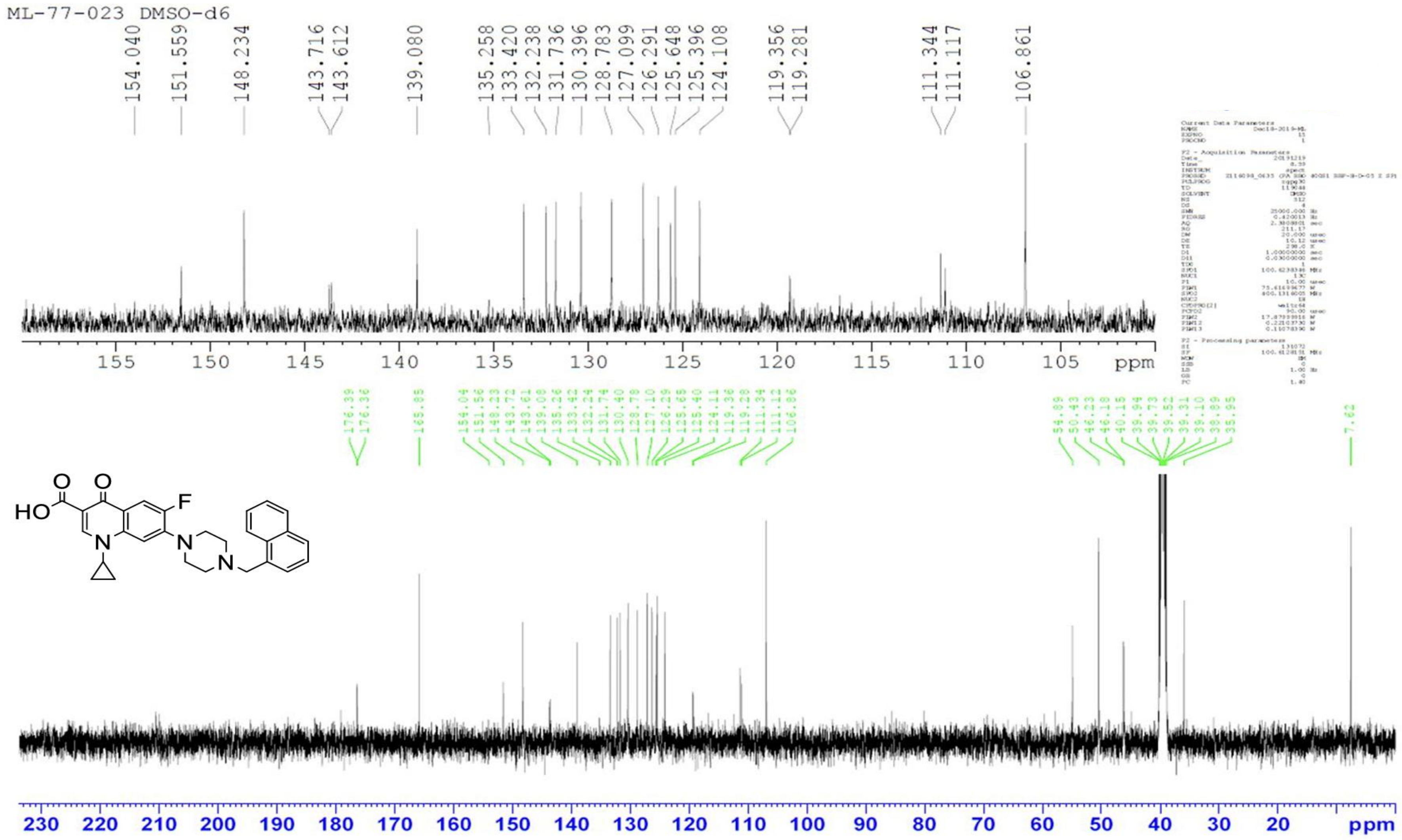

Compound 9 – ^{1}H spectrum

This report was created by ACD/NMR Processor Academic Edition. For more information go to www.acdlabs.com/nmrproc/

15/11/2025 18:15:07

**Acquisition Time (sec)**	5.4526	**Comment**	ML-83-185 CDCl3	**Date**	18 Dec 2019 19:54:40		
**Date Stamp**	18 Dec 2019 19:54:40						
**File Name**	D:\Full KCL backup 20-11-24\Mark Laws\Documents\PhD\Work\Manuscripts\005 positions paper\Supporting info\ML-83-185\130\PDATA\1\1r						
**Frequency (MHz)**	400.13	**Nucleus**	1H	**Number of Transients**	16	**Origin**	spect
**Original Points Count**	65536	**Owner**	nmrsu	**Points Count**	131072	**Pulse Sequence**	zg30
**Receiver Gain**	62.24	**SW(cyclical) (Hz)**	12019.23	**Solvent**	CHLOROFORM-d	**Spectrum Offset (Hz)**	2465.6240
**Spectrum Type**	STANDARD	**Sweep Width (Hz)**	12019.14	**Temperature (degree C)**	24.943		

^{1}H NMR (400 MHz, CHLOROFORM-d) δ 8.15 (d, J = 8.34 Hz, 1H), 7.86 (d, J = 7.79 Hz, 1H), 7.77 (d, J = 8.07 Hz, 1H), 7.53 - 7.59 (m, 1H), 7.46 - 7.53 (m, 1H), 7.31 - 7.43 (m, 2H), 6.22 (br. s., 1H), 5.19 (br. s., 1H), 4.54 (q, J = 7.06 Hz, 1H), 3.94 (d, J = 15.59 Hz, 1H), 3.78 (d, J = 18.16 Hz, 1H), 3.69 (s, 3H), 3.56 (br. s., 1H), 3.50 (br. s., 1H), 1.38 (br. s., 9H)

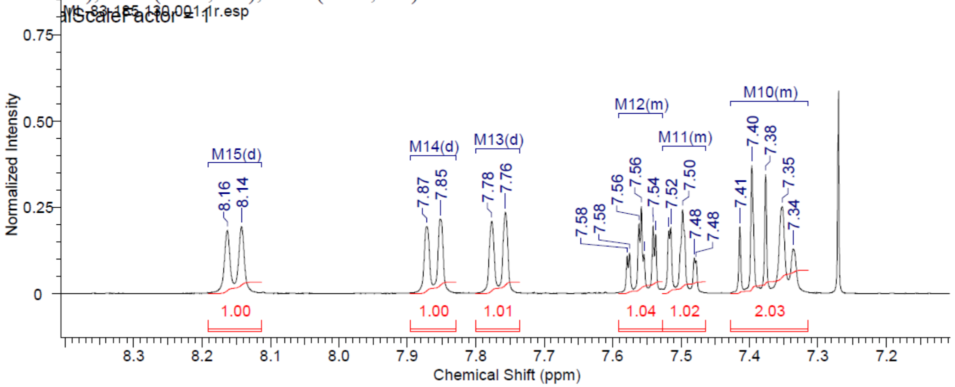

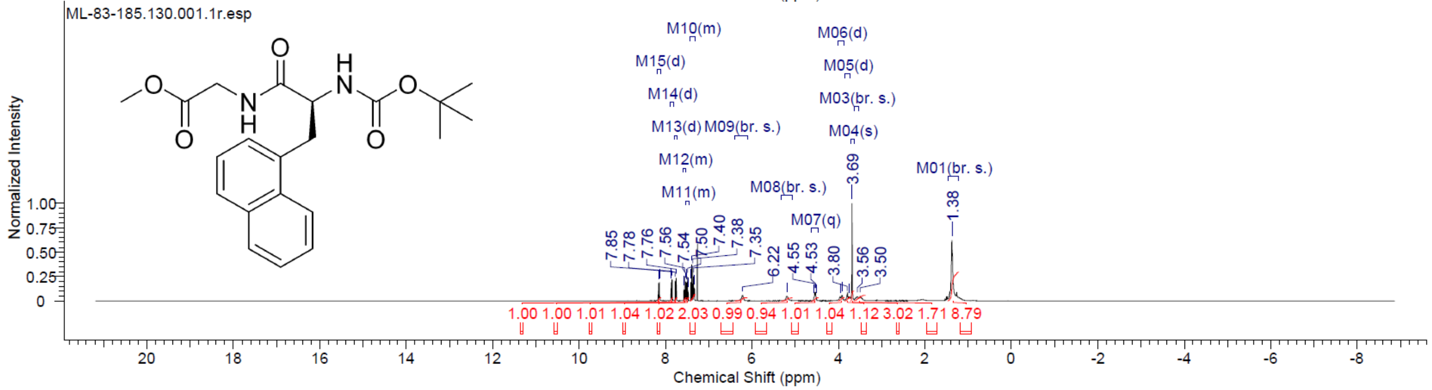

Compound 9– ^{13}C spectrum

This report was created by ACD/NMR Processor Academic Edition. For more information go to www.acdlabs.com/nmrproc/

15/11/2025 14:51:54

Acquisition Time (sec)	2.3809	*Comment*	ML-83-185 CDCl3	*Date*	18 Dec 2019 21:02:56		
Date Stamp	18 Dec 2019 21:02:56						
File Name	D:\Full KCL backup 20-11-24\Mark Laws\Documents\PhD\Work\Manuscripts\005 positions paper\Supporting info\ML-83-185\134\PDATA\1\1r						
Frequency (MHz)	100.61	*Nucleus*	13C	*Number of Transients*	512	*Origin*	spect
Original Points Count	59522	*Owner*	nmrsu	*Points Count*	131072	*Pulse Sequence*	zgpg30
Receiver Gain	211.17	*SW(cyclical) (Hz)*	25000.00	*Solvent*	CHLOROFORM-d	*Spectrum Offset (Hz)*	11062.6318
Spectrum Type	STANDARD	*Sweep Width (Hz)*	24999.81	*Temperature (degree C)*	25.004		

^{13}C NMR (101 MHz, CHLOROFORM-d) δ 171.5, 169.6, 155.3, 133.9, 132.8, 131.9, 128.8, 127.8, 127.7, 126.5, 125.8, 125.4, 123.5, 80.2, 55.4, 52.3, 41.1, 35.9, 28.2

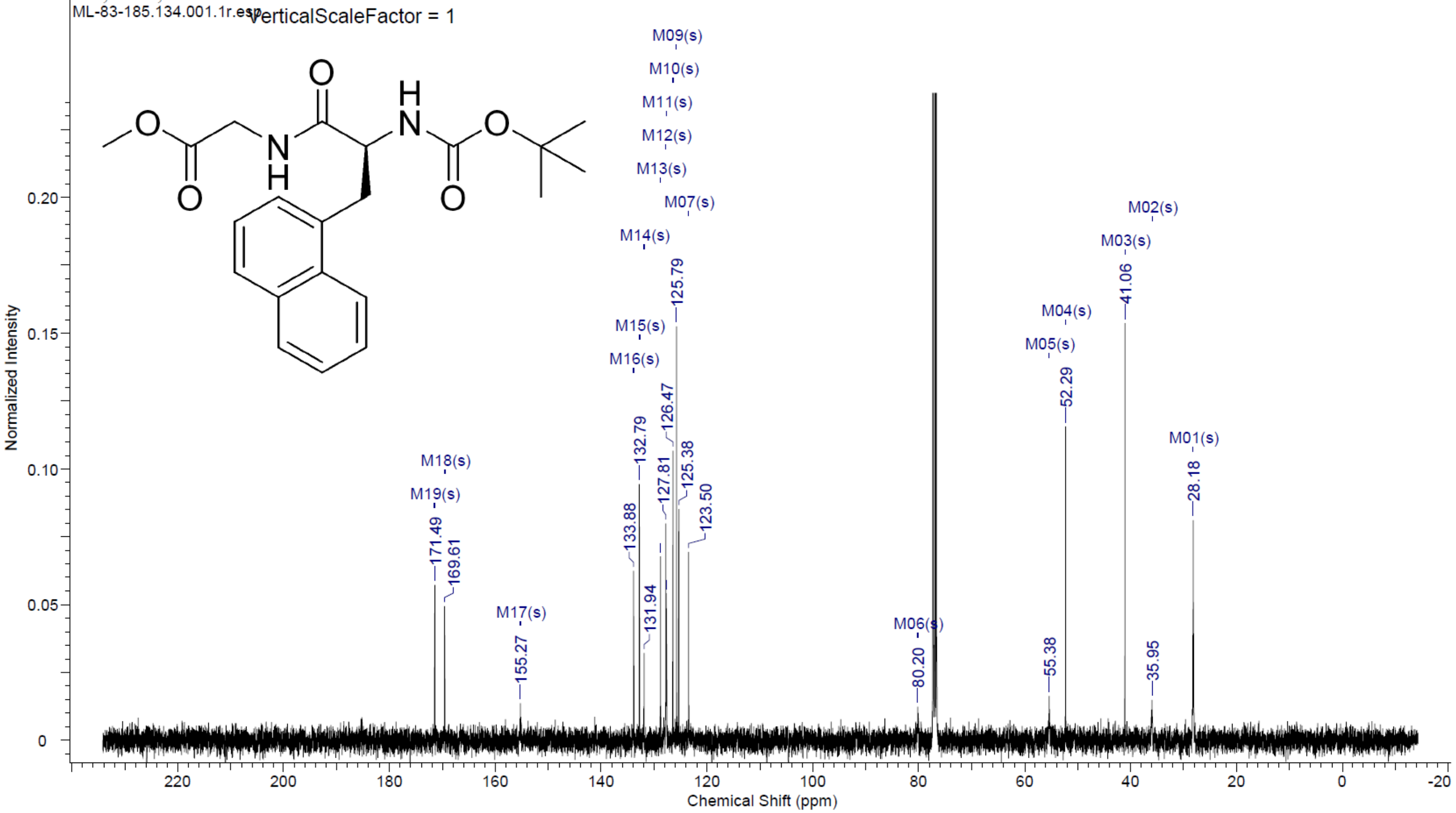

Compound 11– ^{1}H spectrum

This report was created by ACD/NMR Processor Academic Edition. For more information go to www.acdlabs.com/nmrproc/

15/11/2025 18:18:28

Acquisition Time (sec)	5.4526	***Comment***	ML-83-189	***Date***	02 Feb 2018 12:50:08		
Date Stamp	02 Feb 2018 12:50:08			***File Name***	\\137.73.205.115\data\ML\nmr\Feb02-2018-ML\11\PDATA\1\1r		
Frequency (MHz)	400.13	***Nucleus***	1H	***Number of Transients***	2	***Origin***	spect
Original Points Count	65536	***Owner***	nmrsu	***Points Count***	131072	***Pulse Sequence***	zg30
Receiver Gain	41.08	***SW(cyclical) (Hz)***	12019.23	***Solvent***	CHLOROFORM-d		
Spectrum Offset (Hz)	2465.1655	***Spectrum Type***	STANDARD	***Sweep Width (Hz)***	12019.14	***Temperature (degree C)***	25.001

^{1}H NMR (400 MHz, CHLOROFORM-d) δ 8.06 (d, J = 7.61 Hz, 1H), 7.86 (d, J = 6.88 Hz, 1H), 7.75 (d, J = 7.61 Hz, 1H), 7.50 (t, J = 6.14 Hz, 2H), 7.29 - 7.44 (m, 2H), 3.13 - 3.31 (m, 1H), 3.05 (d, J = 10.73 Hz, 2H), 2.84 - 2.99 (m, 3H), 2.80 (t, J = 11.55 Hz, 1H), 2.54 - 2.72 (m, 2H), 2.08 (br. s., 3H)

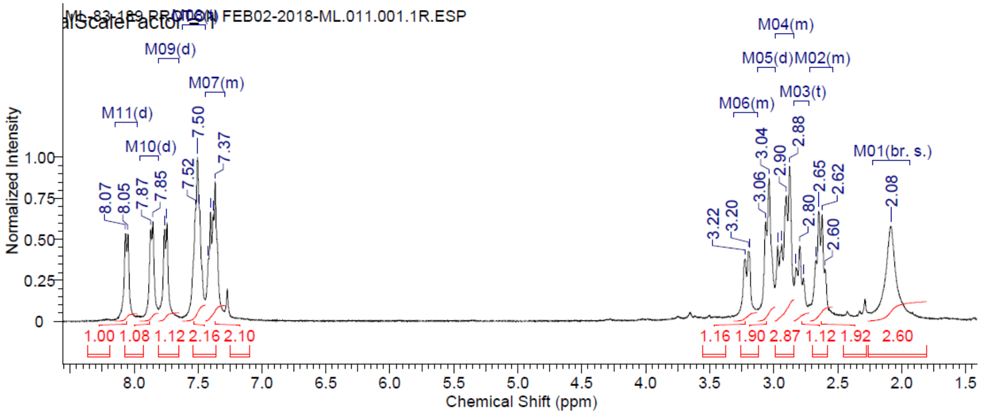

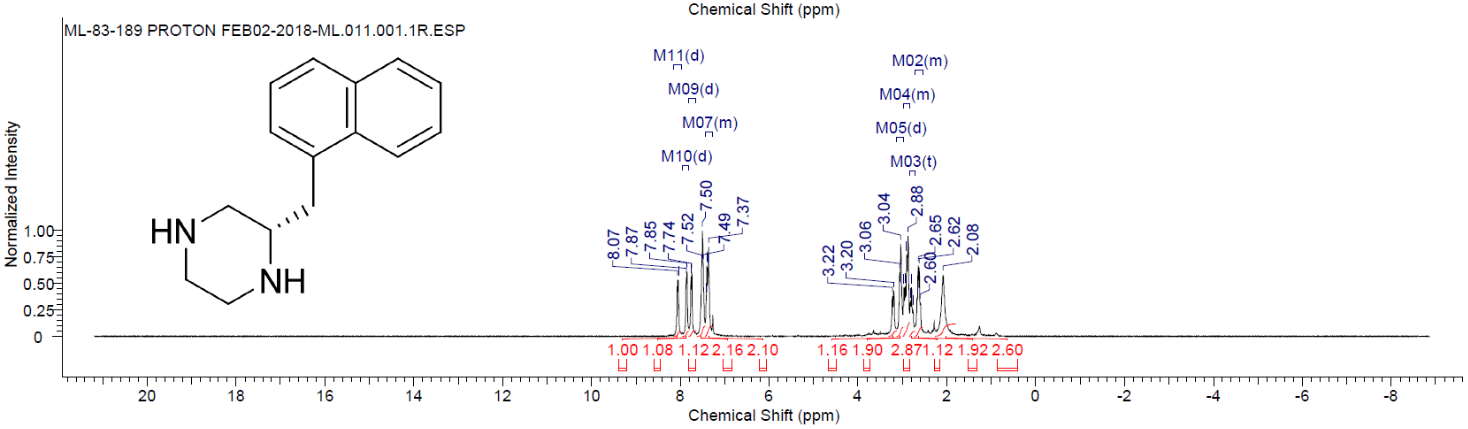

Compound 11– ^{13}C spectrum

This report was created by ACD/NMR Processor Academic Edition. For more information go to www.acdlabs.com/nmrproc/

15/11/2025 13:30:00

Acquisition Time (sec)	2.3809	*Comment*	ML-83-189	*Date*	02 Feb 2018 13:34:56		
Date Stamp	02 Feb 2018 13:34:56			*File Name*	\\137.73.205.115\data\ML\nmr\Feb02-2018-ML\13\PDATA\1\1r		
Frequency (MHz)	100.61	*Nucleus*	13C	*Number of Transients*	512	*Origin*	spect
Original Points Count	59522	*Owner*	nmrsu	*Points Count*	131072	*Pulse Sequence*	zgpg30
Receiver Gain	211.17	*SW(cyclical) (Hz)*	25000.00	*Solvent*	CHLOROFORM-d		
Spectrum Offset (Hz)	11056.5283	*Spectrum Type*	STANDARD	*Sweep Width (Hz)*	24999.81	*Temperature (degree C)*	25.001

^{13}C NMR (101 MHz, CHLOROFORM-d) δ 134.2, 133.9, 132.0, 128.7, 127.4, 127.2, 125.9, 125.6, 125.3, 123.8, 56.5, 52.5, 47.1, 46.1, 37.9

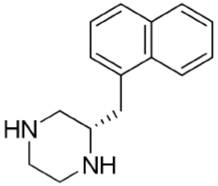

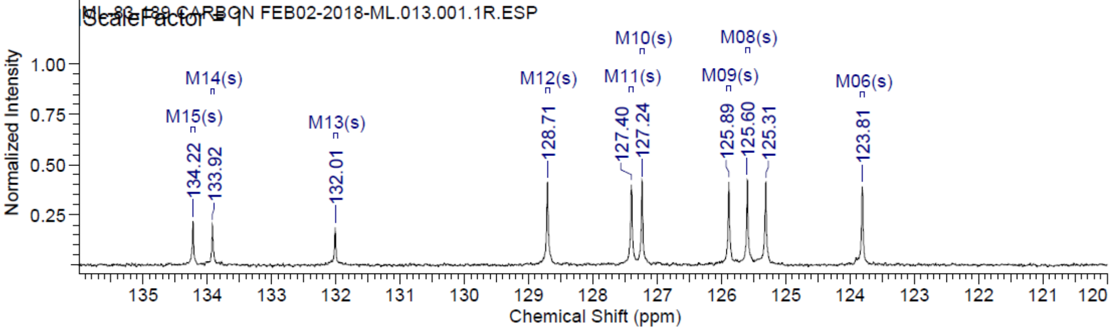

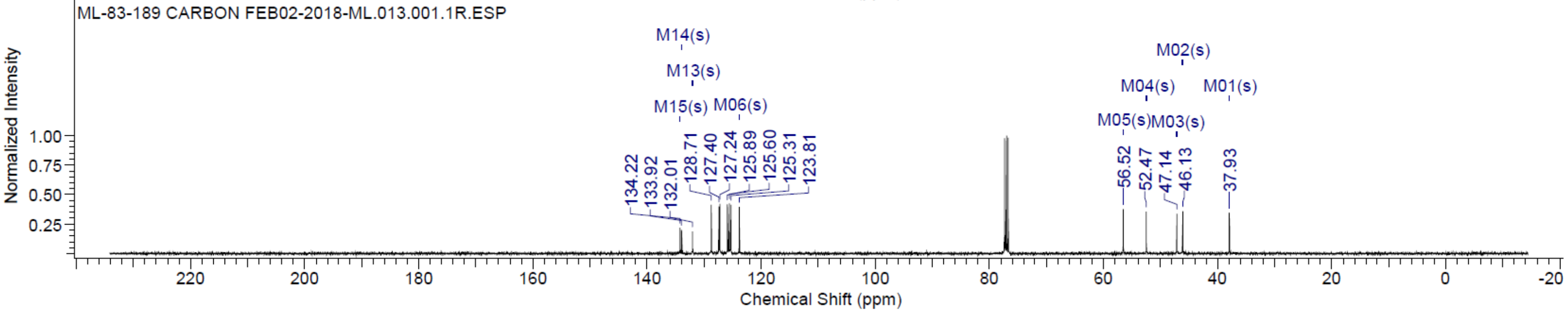

No.	(ppm)	(Hz)	Height	No.	(ppm)	(Hz)	Height	No.	(ppm)	(Hz)	Height	No.	(ppm)	(Hz)	Height
1	37.93	3816.5	0.3454	5	56.52	5686.9	0.3751	9	125.89	12665.7	0.4145	13	132.01	13281.9	0.1887
2	46.13	4641.6	0.3581	6	123.81	12456.6	0.3927	10	127.24	12801.5	0.4204	14	133.92	13474.0	0.2003
3	47.14	4743.3	0.3387	7	125.31	12607.9	0.4104	11	127.40	12818.3	0.4006	15	134.22	13504.7	0.2188
4	52.47	5279.6	0.3540	8	125.60	12636.7	0.4257	12	128.71	12949.5	0.4119				

Compound 12– ^{1}H spectrum

This report was created by ACD/NMR Processor Academic Edition. For more information go to www.acdlabs.com/nmrproc/

15/11/2025 17:30:31

Acquisition Time (sec)	5.4526	***Comment***	ML-83-192	***Date***	21 Feb 2018 18:08:00		
Date Stamp	21 Feb 2018 18:08:00			***File Name***	\\137.73.205.115\data\ML\nmr\Feb21-2018-ML\10\PDATA\1\1r		
Frequency (MHz)	400.13	***Nucleus***	1H	***Number of Transients***	16	***Origin***	spect
Original Points Count	65536	***Owner***	nmr	***Points Count***	131072	***Pulse Sequence***	zg30
Receiver Gain	62.24	***SW(cyclical) (Hz)***	12019.23	***Solvent***	CHLOROFORM-d		
Spectrum Offset (Hz)	2465.4412	***Spectrum Type***	STANDARD	***Sweep Width (Hz)***	12019.14	***Temperature (degree C)***	24.262

^{1}H NMR (400 MHz, CHLOROFORM-d) δ 8.70 (s, 1H), 8.10 (d, J = 8.16 Hz, 1H), 7.96 (d, J = 13.20 Hz, 1H), 7.86 - 7.92 (m, 1H), 7.77 - 7.83 (m, 1H), 7.48 - 7.60 (m, 2H), 7.39 - 7.48 (m, 2H), 7.18 (d, J = 7.15 Hz, 1H), 3.71 (d, J = 10.91 Hz, 1H), 3.64 (d, J = 11.55 Hz, 1H), 3.37 - 3.47 (m, 1H), 3.26 - 3.37 (m, 2H), 3.16 - 3.25 (m, 1H), 3.04 - 3.16 (m, 2H), 2.95 - 3.04 (m, 1H), 2.91 (t, J = 10.73 Hz, 1H), 1.13 - 1.23 (m, 2H), 1.01 - 1.10 (m, 2H)

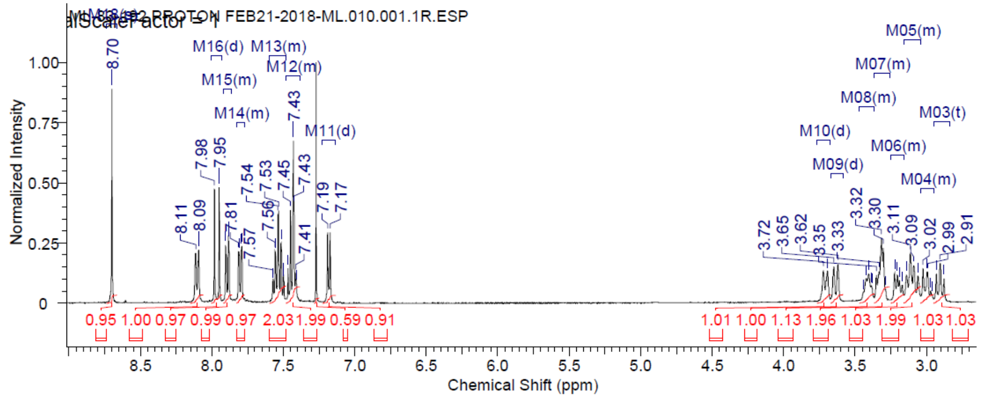

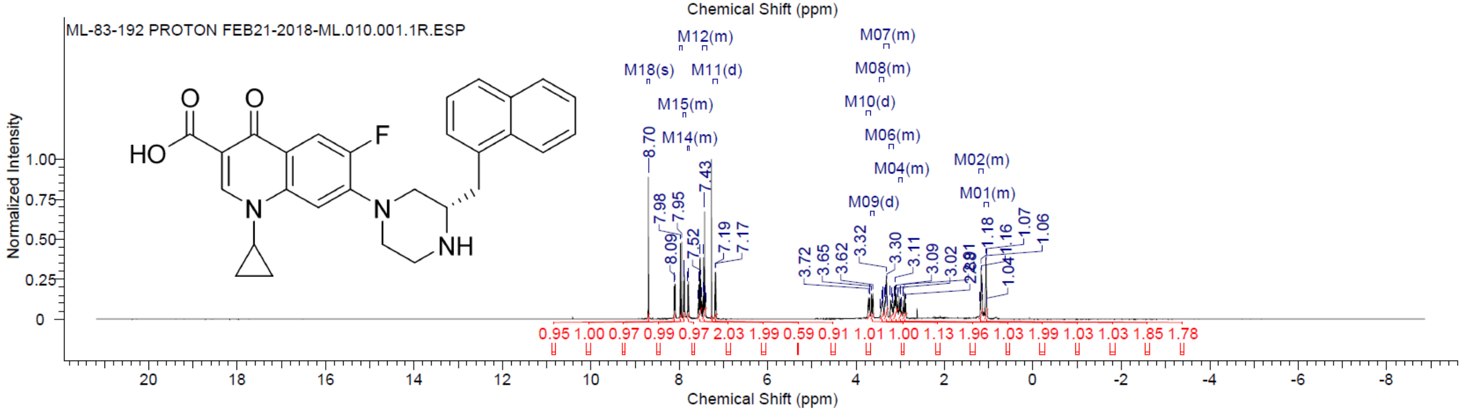

Compound 12– ^{13}C spectrum

This report was created by ACD/NMR Processor Academic Edition. For more information go to www.acdlabs.com/nmrproc/

15/11/2025 13:45:33

Acquisition Time (sec)	2.3809	***Comment***	ML-83-192	***Date***	21 Feb 2018 21:20:00		
Date Stamp	21 Feb 2018 21:20:00			***File Name***	\\137.73.205.115\data\ML\nmr\Feb21-2018-ML\15\PDATA\1\1r		
Frequency (MHz)	100.61	***Nucleus***	13C	***Number of Transients***	512	***Origin***	spect
Original Points Count	59522	***Owner***	nmr	***Points Count***	131072	***Pulse Sequence***	zgpg30
Receiver Gain	211.17	***SW(cyclical) (Hz)***	25000.00	***Solvent***	CHLOROFORM-d		
Spectrum Offset (Hz)	11061.6787	***Spectrum Type***	STANDARD	***Sweep Width (Hz)***	24999.81	***Temperature (degree C)***	25.082

^{13}C NMR (101 MHz, CHLOROFORM-d) δ 177.0, 177.0, 167.1, 154.7, 152.2, 147.2, 145.7, 145.6, 139.0, 134.0, 133.6, 132.0, 128.9, 127.7, 127.6, 126.2, 125.8, 125.4, 123.6. 119.5, 119.5, 112.5, 112.3, 108.0, 104.7, 104.7, 55.5, 55.5, 55.1, 50.5, 50.4, 45.6, 37.5, 35.1, 8.1, 8.0

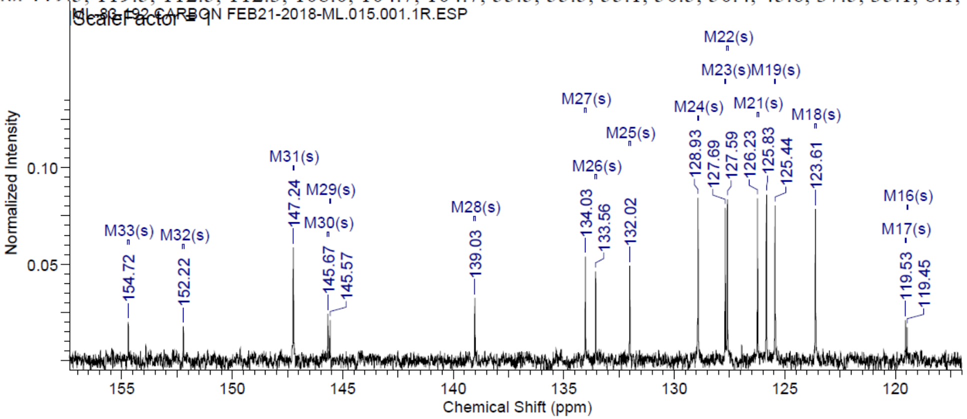

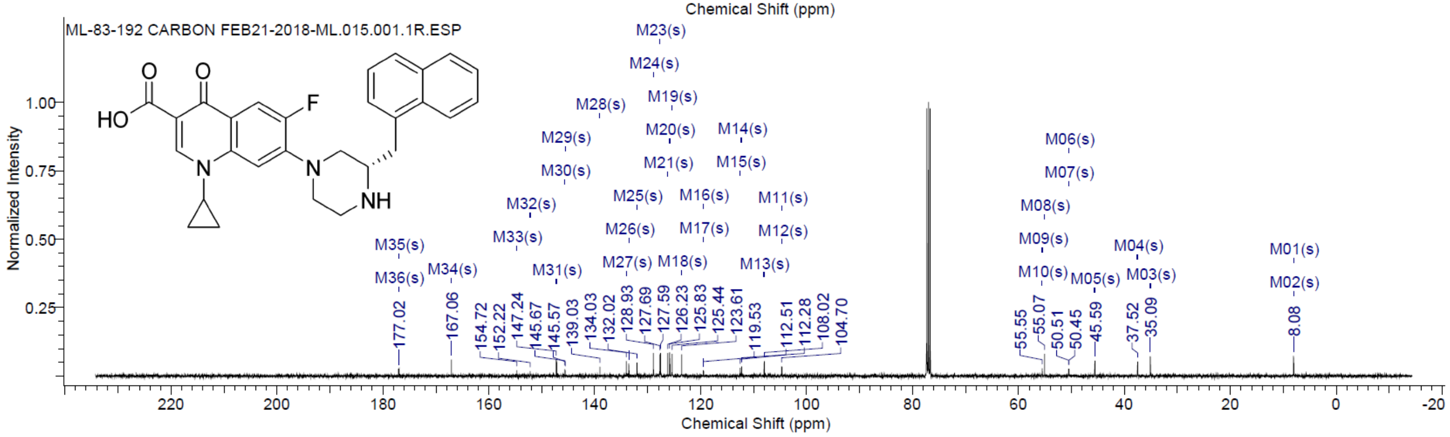

Compound 12– proton-decoupled ^{19}F spectrum

This report was created by ACD/NMR Processor Academic Edition. For more information go to www.acdlabs.com/nmrproc/

16/11/2025 10:50:05

Acquisition Time (sec)	1.4680	*Comment*	ML-83-192	*Date*	21 Feb 2018 18:16:32		
Date Stamp	21 Feb 2018 18:16:32			*File Name*	\\137.73.205.115\data\ML\nmr\Feb21-2018-ML\13\PDATA\1\1r		
Frequency (MHz)	376.50	*Nucleus*	19F	*Number of Transients*	16	*Origin*	spect
Original Points Count	131072	*Owner*	nmr	*Points Count*	262144	*Pulse Sequence*	zgig30
Receiver Gain	211.17	*SW(cyclical) (Hz)*	89285.71	*Solvent*	CHLOROFORM-d		
Spectrum Offset (Hz)	-37648.4180	*Spectrum Type*	STANDARD	*Sweep Width (Hz)*	89285.37	*Temperature (degree C)*	24.422

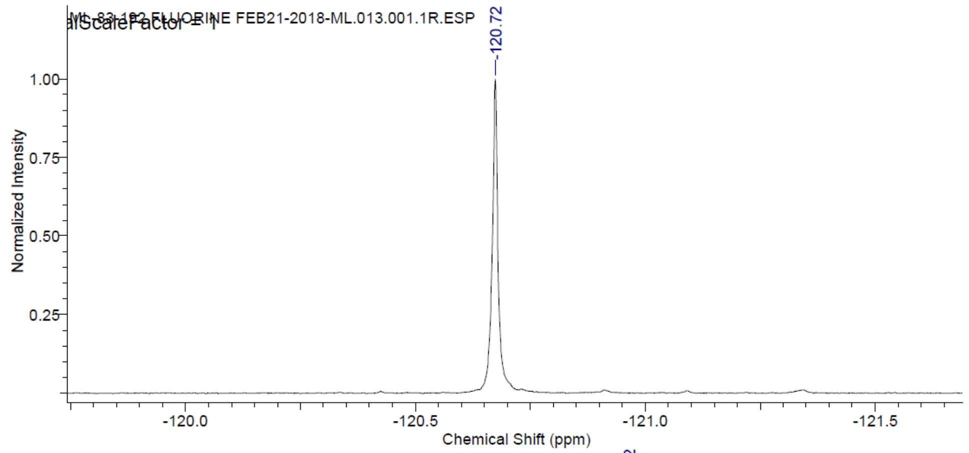

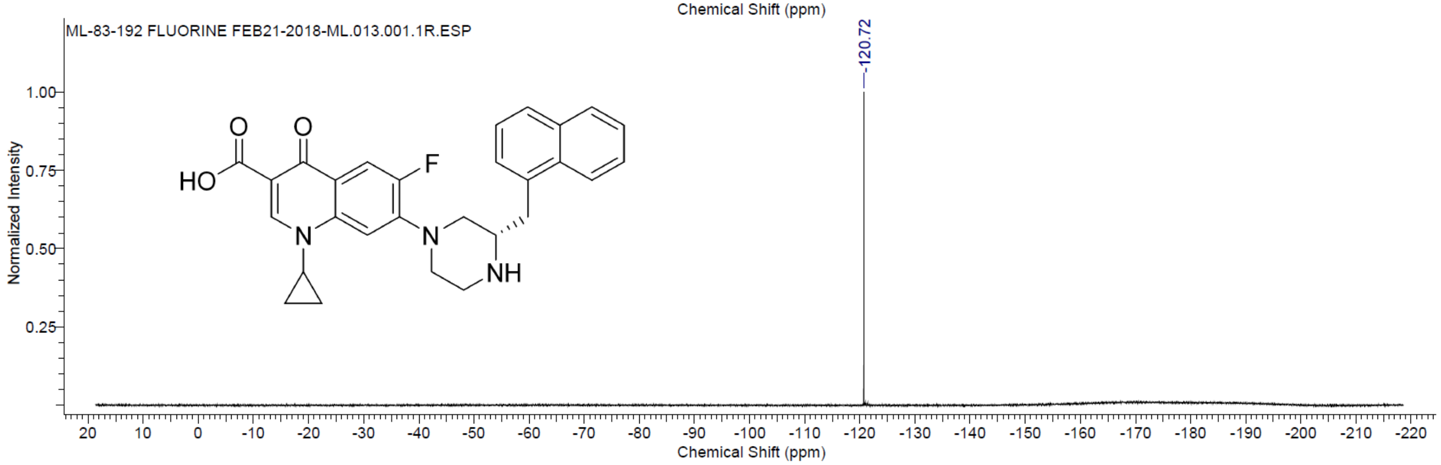

Compound 13– ^{1}H spectrum

This report was created by ACD/NMR Processor Academic Edition. For more information go to www.acdlabs.com/nmrproc/

15/11/2025 17:34:13

Acquisition Time (sec)	5.4526	*Comment*	ML-123-008	*Date*	10 Dec 2018 15:32:16		
Date Stamp	10 Dec 2018 15:32:16			*File Name*	\\137.73.205.115\data\ML\nmr\Dec10-2018-ML\12\PDATA\1\1r		
Frequency (MHz)	400.13	*Nucleus*	1H	*Number of Transients*	16	*Origin*	spect
Original Points Count	65536	*Owner*	nmrsu	*Points Count*	131072	*Pulse Sequence*	zg30
Receiver Gain	126.48	*SW(cyclical) (Hz)*	12019.23	*Solvent*	CHLOROFORM-d		
Spectrum Offset (Hz)	2465.6240	*Spectrum Type*	STANDARD	*Sweep Width (Hz)*	12019.14	*Temperature (degree C)*	22.186

^{1}H NMR (400 MHz, CHLOROFORM-d) δ 11.63 (br. s., 1H), 7.83 - 7.95 (m, 2H), 7.07 (dt, J = 6.24, 9.81 Hz, 2H)

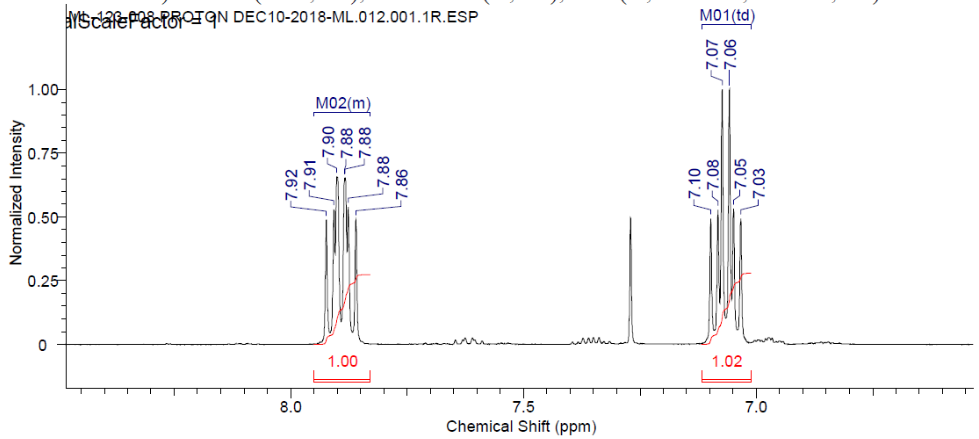

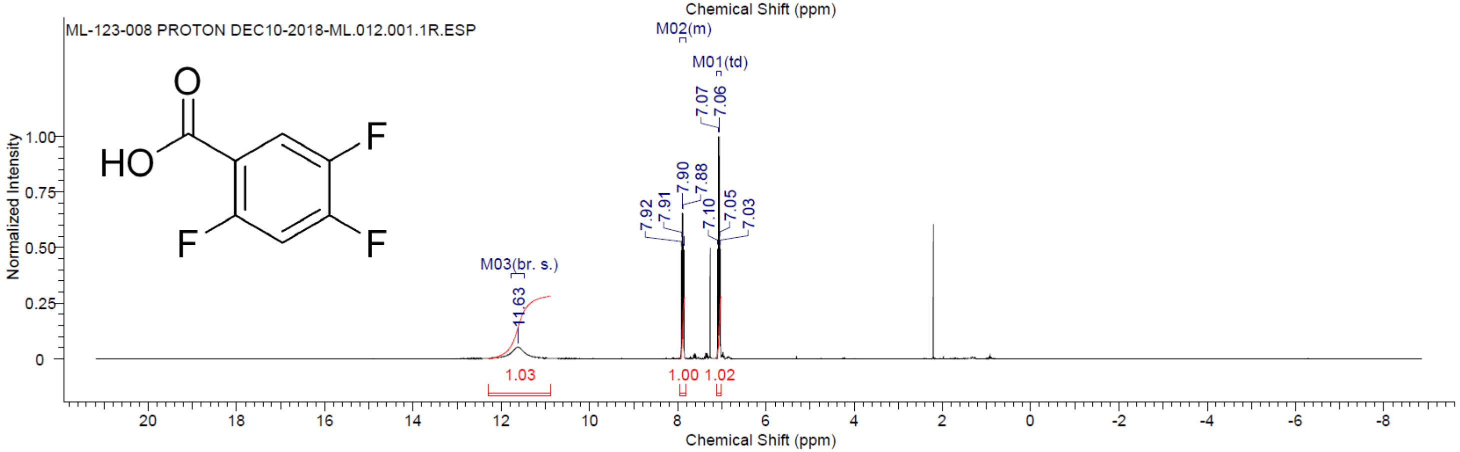

Compound 13– ^{13}C spectrum

This report was created by ACD/NMR Processor Academic Edition. For more information go to www.acdlabs.com/nmrproc/

15/11/2025 13:42:39

Acquisition Time (sec)	2.3809	***Comment***	ML-123-008	***Date***	10 Dec 2018 21:20:00		
Date Stamp	10 Dec 2018 21:20:00			***File Name***	\\137.73.205.115\data\ML\nmr\Dec10-2018-ML\17\PDATA\1\1r		
Frequency (MHz)	100.61	***Nucleus***	13C	***Number of Transients***	512	***Origin***	spect
Original Points Count	59522	***Owner***	nmrsu	***Points Count***	131072	***Pulse Sequence***	zgpg30
Receiver Gain	211.17	***SW(cyclical) (Hz)***	25000.00	***Solvent***	CHLOROFORM-d		
Spectrum Offset (Hz)	11066.0557	***Spectrum Type***	STANDARD	***Sweep Width (Hz)***	24999.81	***Temperature (degree C)***	23.498

^{13}C NMR (101 MHz, CHLOROFORM-d) δ 168.0, 168.0, 160.1, 160.1, 160.0, 160.0, 157.5, 157.5, 157.4, 157.4, 155.5, 155.4, 155.4, 155.2, 152.9, 152.8, 152.8, 152.6, 147.8, 147.8, 147.7, 147.7, 145.4, 145.3, 145.3, 145.2, 120.7, 120.7, 120.7, 120.5, 120.5, 120.4, 113.9, 113.9, 113.9, 113.8, 113.8, 113.8, 113.8, 113.7, 107.6, 107.4, 107.3, 107.1

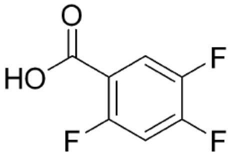

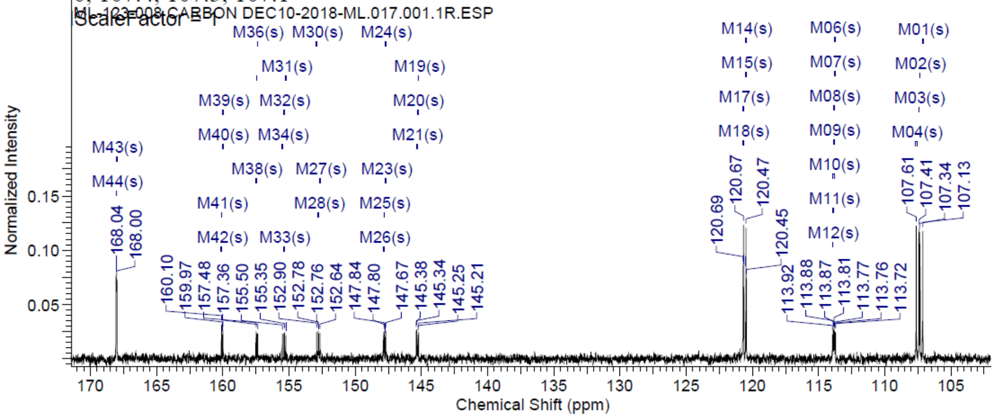

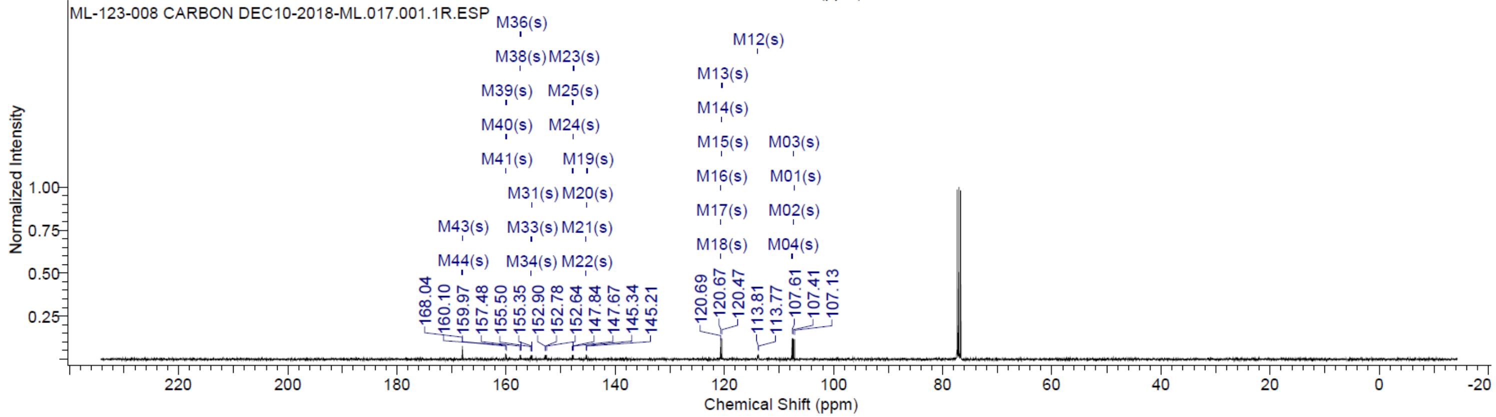

Compound 13– proton-decoupled ^{19}F spectrum

This report was created by ACD/NMR Processor Academic Edition. For more information go to www.acdlabs.com/nmrproc/

16/11/2025 10:53:19

Acquisition Time (sec)	1.4680	***Comment***	ML-123-008	***Date***	10 Dec 2018 15:30:08		
Date Stamp	10 Dec 2018 15:30:08			***File Name***	\\137.73.205.115\data\ML\nmr\Dec10-2018-ML\11\PDATA\1\1r		
Frequency (MHz)	376.50	***Nucleus***	19F	***Number of Transients***	16	***Origin***	spect
Original Points Count	131072	***Owner***	nmrsu	***Points Count***	262144	***Pulse Sequence***	zgig30
Receiver Gain	211.17	***SW(cyclical) (Hz)***	89285.71	***Solvent***	CHLOROFORM-d		
Spectrum Offset (Hz)	-37648.4180	***Spectrum Type***	STANDARD	***Sweep Width (Hz)***	89285.37	***Temperature (degree C)***	22.384

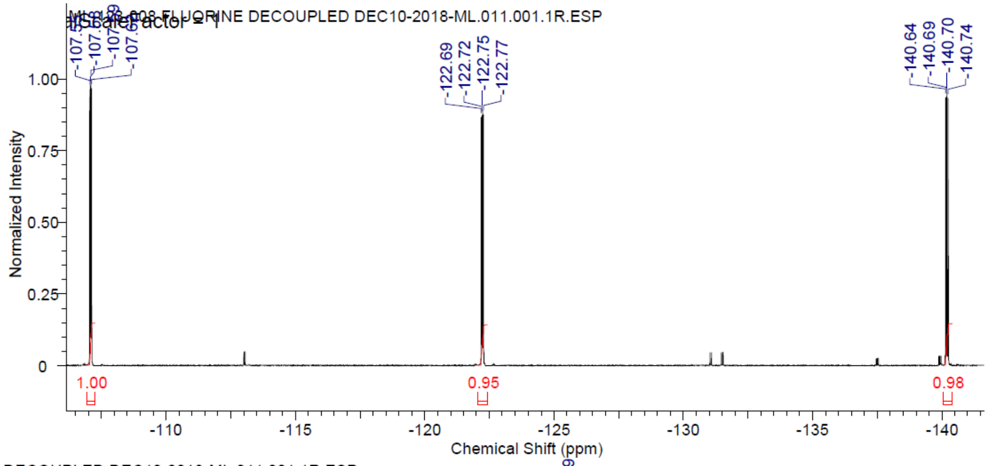

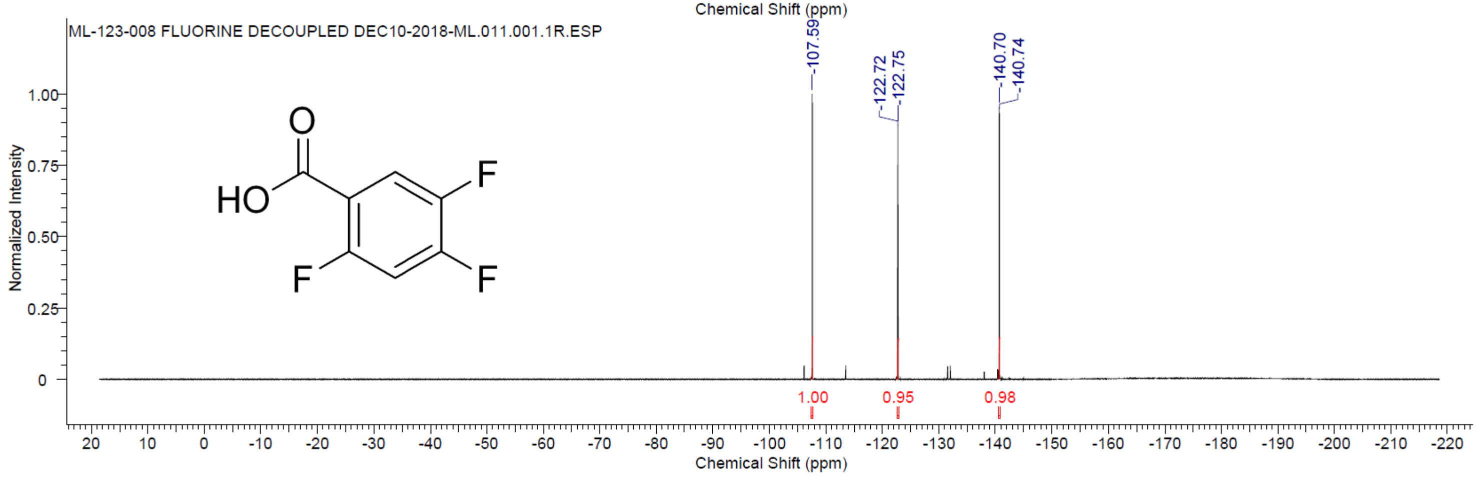

Compound 14– ^{1}H spectrum

^{1}H NMR (400 MHz, CHLOROFORM-d) δ 8.15 (d, J = 5.96 Hz, 1H), 7.78 - 7.99 (m, 3H), 7.40 - 7.69 (m, 4H), 6.99 (br. s., 1H)

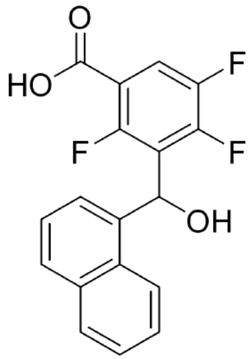

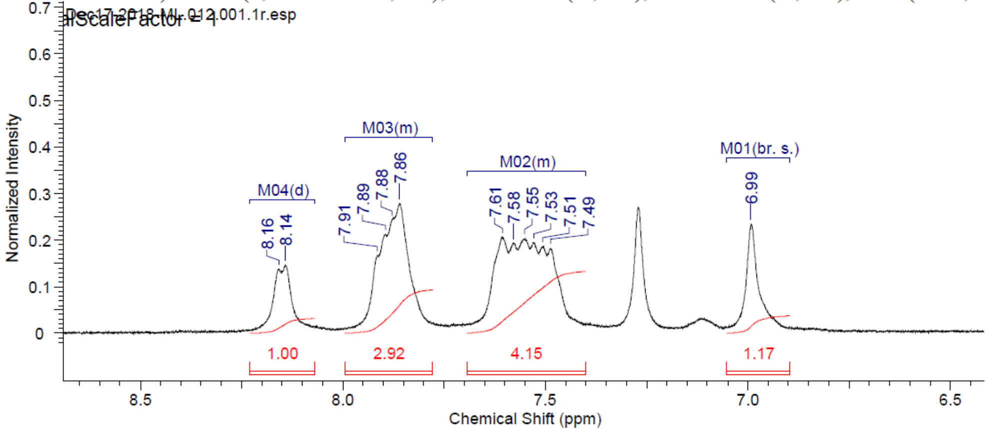

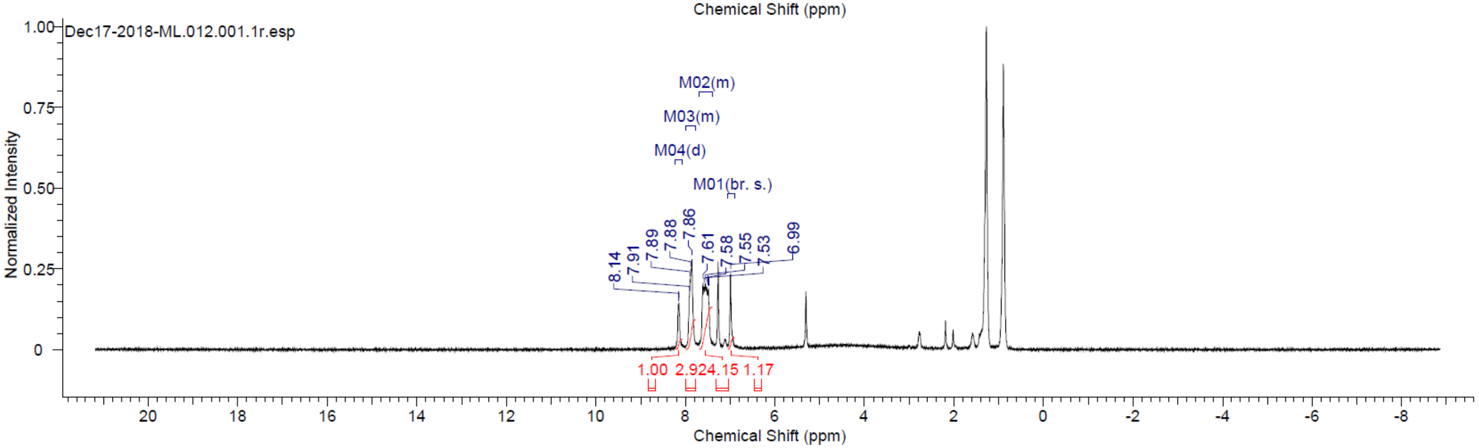

Compound 14– ^{13}C spectrum

This report was created by ACD/NMR Processor Academic Edition. For more information go to www.acdlabs.com/nmrproc/

15/11/2025 15:11:01

**Acquisition Time (sec)**	2.3809	**Comment**	ML-123-010 CDCl3	**Date**	18 Dec 2019 23:32:16		
**Date Stamp**	18 Dec 2019 23:32:16						
**File Name**	D:\Full KCL backup 20-11-24\Mark Laws\Documents\PhD\Work\Manuscripts\005 positions paper\Supporting info\ML-123-009\70\PDATA\1\1r						
**Frequency (MHz)**	100.61	**Nucleus**	13C	**Number of Transients**	512	**Origin**	spect
**Original Points Count**	59522	**Owner**	nmrsu	**Points Count**	131072	**Pulse Sequence**	zgpg30
**Receiver Gain**	211.17	**SW(cyclical) (Hz)**	25000.00	**Solvent**	CHLOROFORM-d	**Spectrum Offset (Hz)**	11055.9561
**Spectrum Type**	STANDARD	**Sweep Width (Hz)**	24999.81	**Temperature (degree C)**	25.001		

^{13}C NMR (101 MHz, CHLOROFORM-d) δ 164.2, 164.2, 156.4, 156.4, 156.3, 156.3, 156.1, 156.0, 156.0, 155.9, 153.8, 153.8, 153.7, 153.7, 153.5, 153.4, 153.4, 153.3, 143.0, 142.9, 142.8, 142.8, 140.4, 140.4, 140.3, 140.2, 137.9, 137.8, 137.8, 137.7, 137.7, 137.6, 133.9, 131.2, 131.0, 129.0, 128.8, 127.3, 126.4, 125.1, 125.0, 122.8, 111.5, 111.5, 111.4, 111.4, 111.3, 108.3, 108.0, 108.0, 107.8, 77.3, 76.7

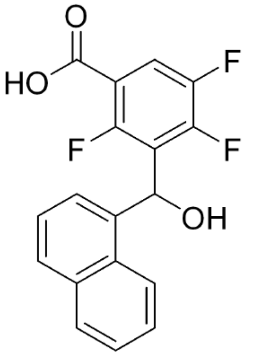

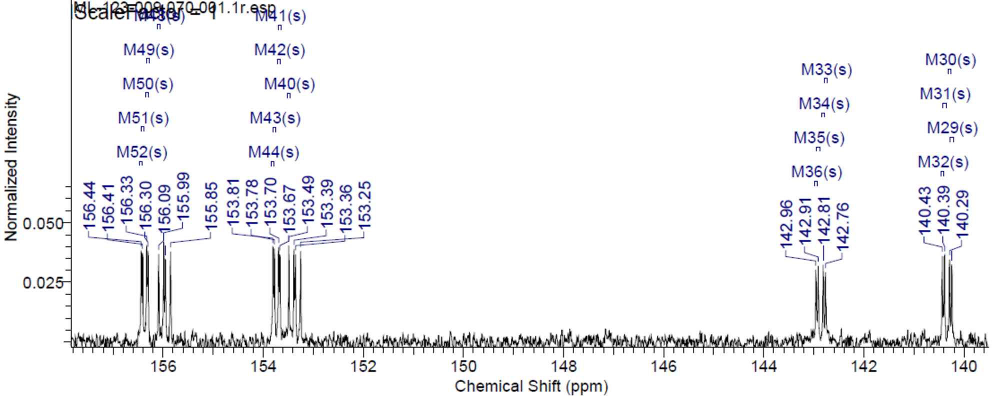

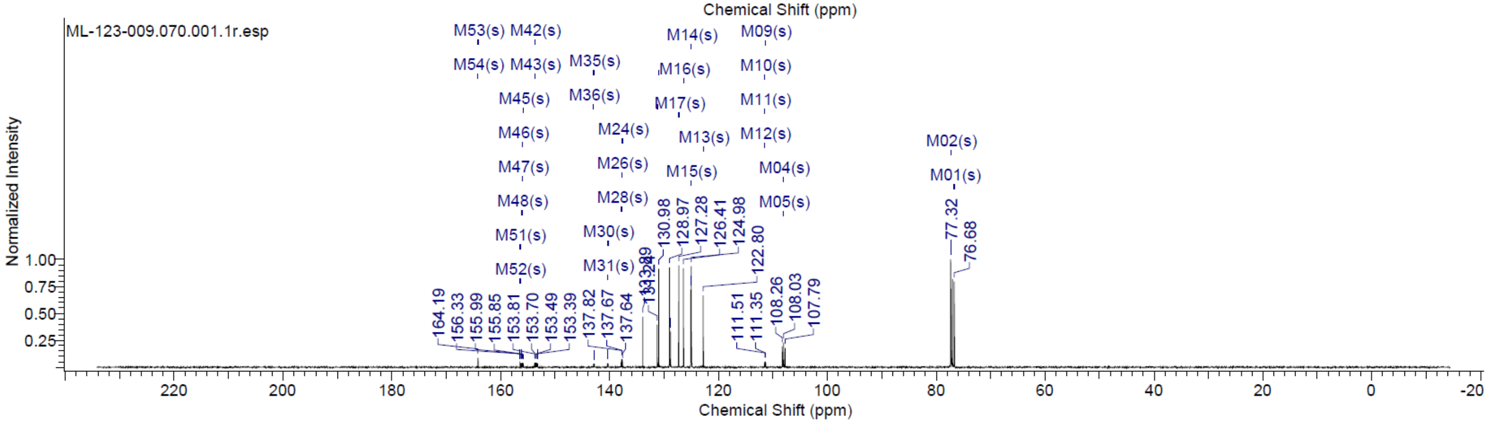

Compound 14– proton-decoupled ^{19}F spectrum

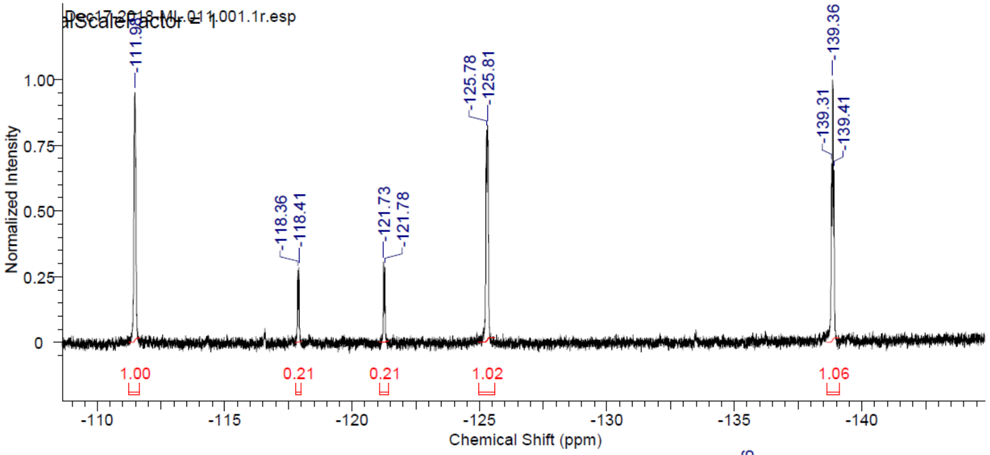

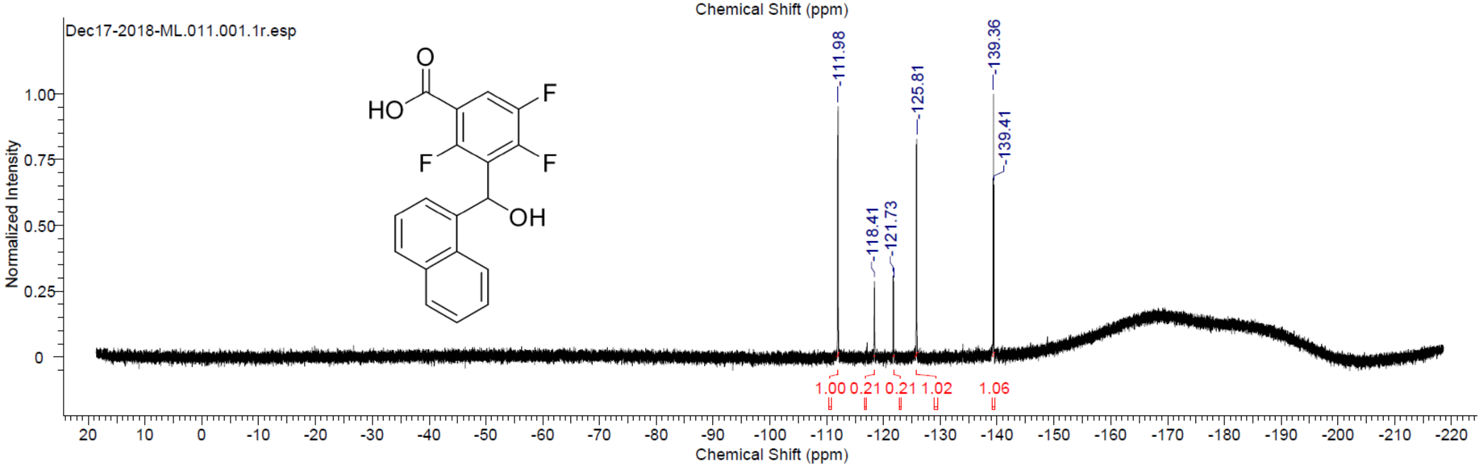

Compound 15– ^{1}H spectrum

This report was created by ACD/NMR Processor Academic Edition. For more information go to www.acdlabs.com/nmrproc/

15/11/2025 17:37:51

Acquisition Time (sec)	5.4526	***Comment***	ML-123-011 acetone-d6			***Date***	20 Dec 2018 18:18:40
Date Stamp	20 Dec 2018 18:18:40			***File Name***	\\137.73.205.115\data\ML\nmr\Dec20-2018-ML\12\PDATA\1\1r		
Frequency (MHz)	400.13	***Nucleus***	1H	***Number of Transients***	16	***Origin***	spect
Original Points Count	65536	***Owner***	nmrsu	***Points Count***	131072	***Pulse Sequence***	zg30
Receiver Gain	116.97	***SW(cyclical) (Hz)***	12019.23	***Solvent***	Acetone	***Spectrum Offset (Hz)***	2464.4834
Spectrum Type	STANDARD	***Sweep Width (Hz)***	12019.14	***Temperature (degree C)***	24.019		

^{1}H NMR (400 MHz, Acetone) δ 8.28 (d, *J* = 8.53 Hz, 1H), 7.95 (d, *J* = 8.16 Hz, 1H), 7.78 - 7.89 (m, 2H), 7.63 (ddd, *J* = 1.38, 6.99, 8.32 Hz, 1H), 7.56 (dt, *J* = 1.10, 7.47 Hz, 1H), 7.41 (t, *J* = 7.66 Hz, 1H), 7.14 (d, *J* = 7.15 Hz, 1H), 4.62 (s, 2H)

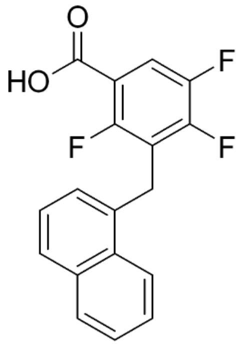

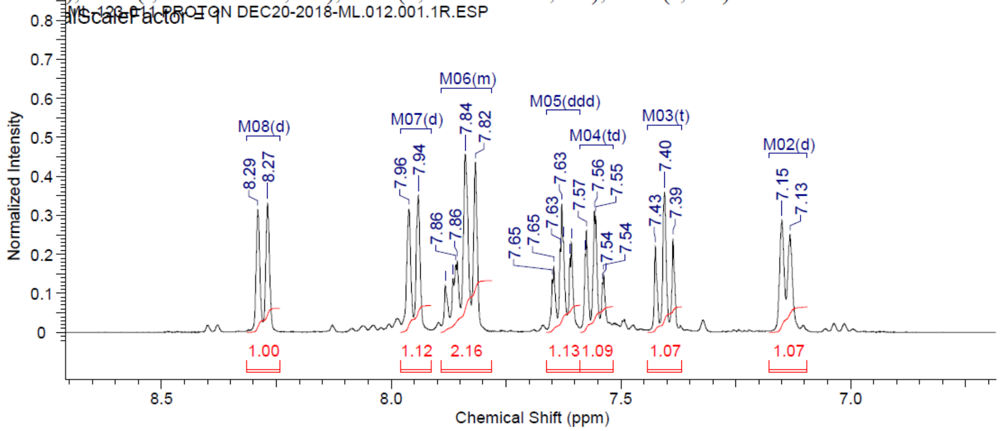

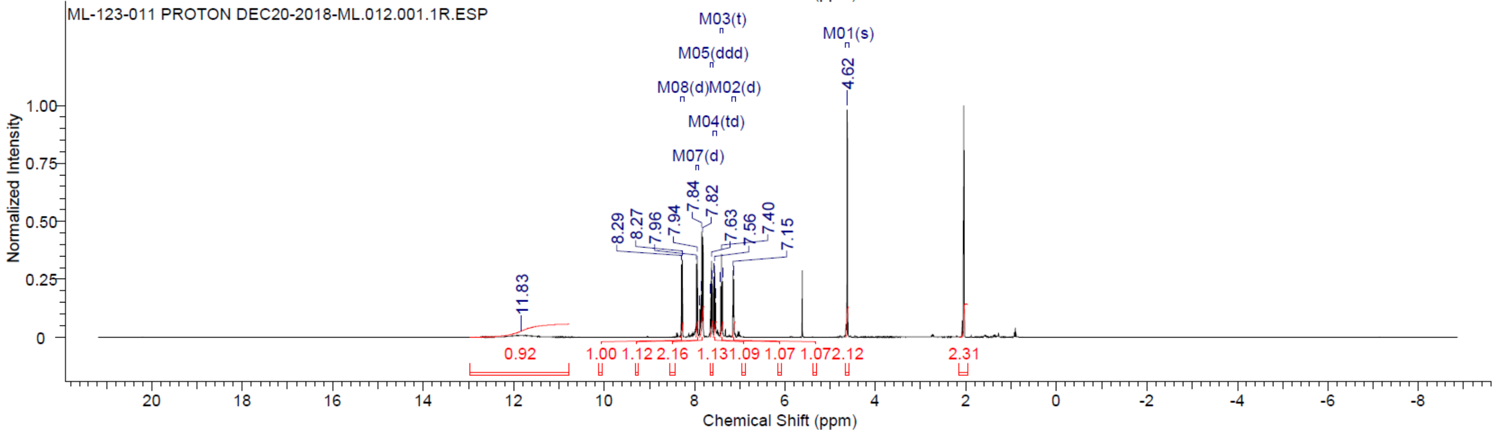

Compound 15– ^{13}C spectrum

This report was created by ACD/NMR Processor Academic Edition. For more information go to www.acdlabs.com/nmrproc/

15/11/2025 13:57:03

Acquisition Time (sec)	2.3809	***Comment***	ML-123-011 acetone-d6			***Date***	21 Dec 2018 01:29:36
Date Stamp	21 Dec 2018 01:29:36			***File Name***	\\137.73.205.115\data\ML\nmr\Dec20-2018-ML\17\PDATA\1\1r		
Frequency (MHz)	100.61	***Nucleus***	13C	***Number of Transients***	512	***Origin***	spect
Original Points Count	59522	***Owner***	nmrsu	***Points Count***	131072	***Pulse Sequence***	zgpg30
Receiver Gain	211.17	***SW(cyclical) (Hz)***	25000.00	***Solvent***	Acetone	***Spectrum Offset (Hz)***	11163.7725
Spectrum Type	STANDARD	***Sweep Width (Hz)***	24999.81	***Temperature (degree C)***	23.080		

^{13}C NMR (101 MHz, Acetone) δ 163.8, 159.0, 159.0, 158.9, 158.9, 156.4, 156.3, 154.2, 153.9, 151.6, 151.6, 151.5, 151.4, 148.7, 148.6, 146.3, 146.1, 134.9, 134.4, 132.6, 129.7, 128.4, 127.4, 126.8, 126.5, 126.2, 124.1, 120.6, 120.5, 120.4, 120.2, 118.8, 118.6, 116.6, 116.6, 116.5, 116.5, 116.4, 116.4, 116.4, 116.3, 26.2

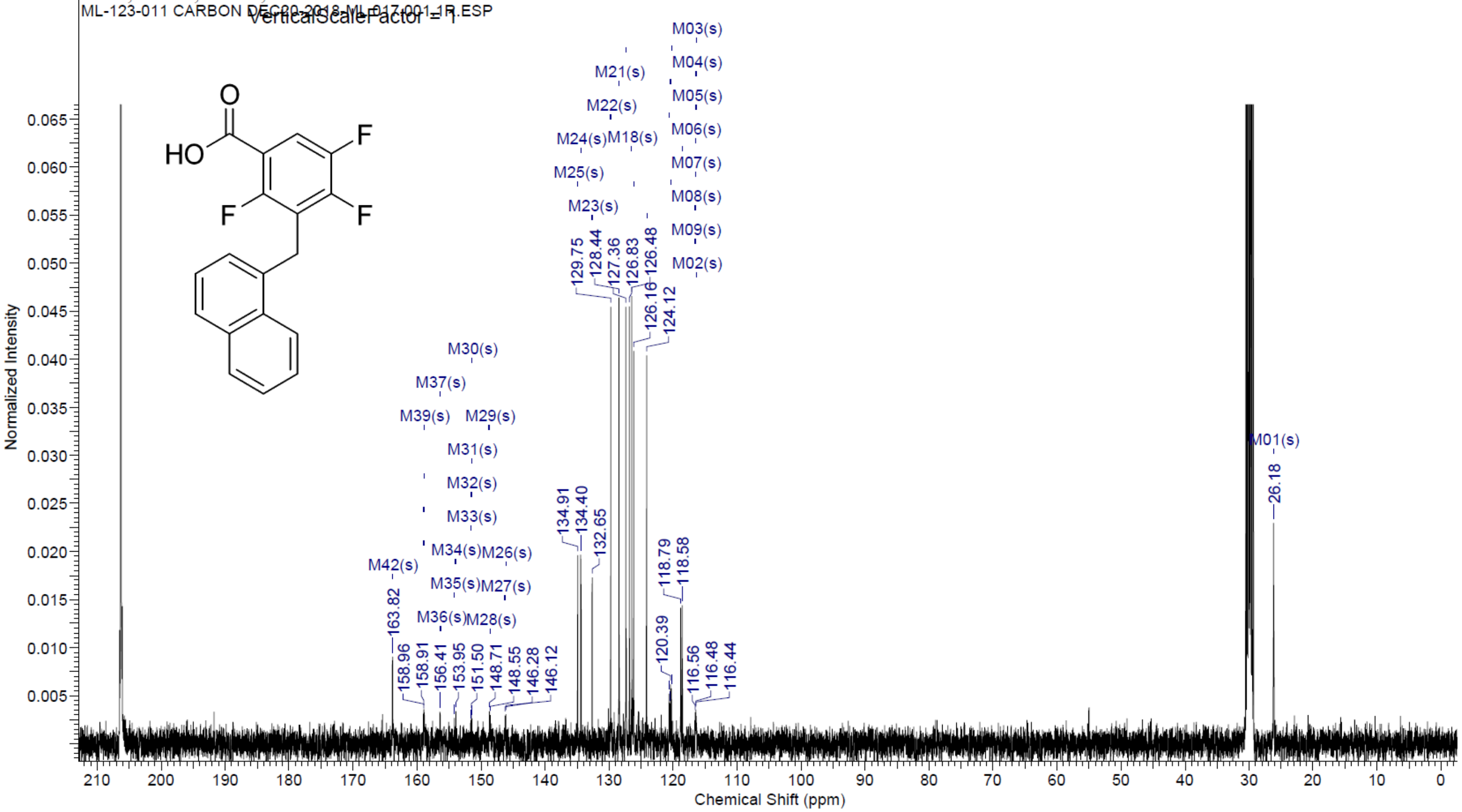

Compound 15– ^{19}F spectrum

This report was created by ACD/NMR Processor Academic Edition. For more information go to www.acdlabs.com/nmrproc/

16/11/2025 11:08:00

Acquisition Time (sec)	1.4680	***Comment***	ML-123-011 acetone-d6			***Date***	20 Dec 2018 18:16:32
Date Stamp	20 Dec 2018 18:16:32			***File Name***	\\137.73.205.115\data\ML\nmr\Dec20-2018-ML\11\PDATA\1\1r		
Frequency (MHz)	376.50	***Nucleus***	19F	***Number of Transients***	16	***Origin***	spect
Original Points Count	131072	***Owner***	nmrsu	***Points Count***	262144	***Pulse Sequence***	zgig30
Receiver Gain	211.17	***SW(cyclical) (Hz)***	89285.71	***Solvent***	Acetone	***Spectrum Offset (Hz)***	-37648.4180
Spectrum Type	STANDARD	***Sweep Width (Hz)***	89285.37	***Temperature (degree C)***	24.327		

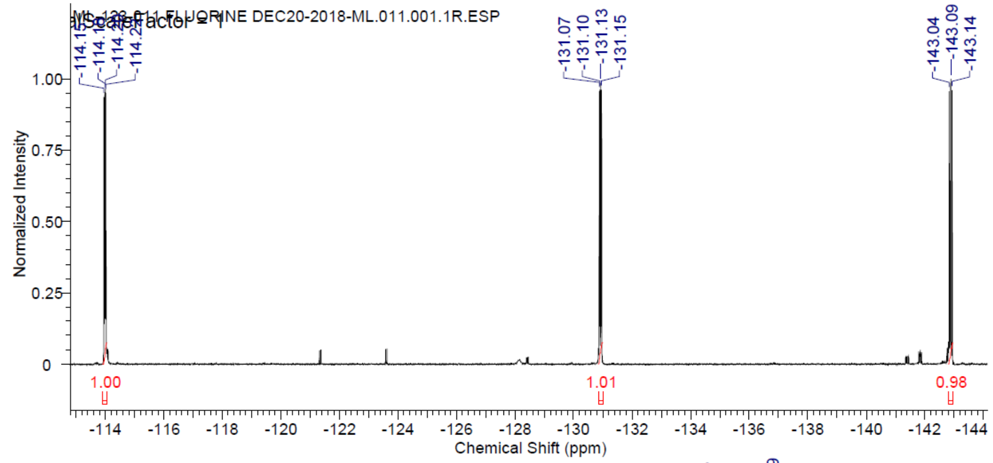

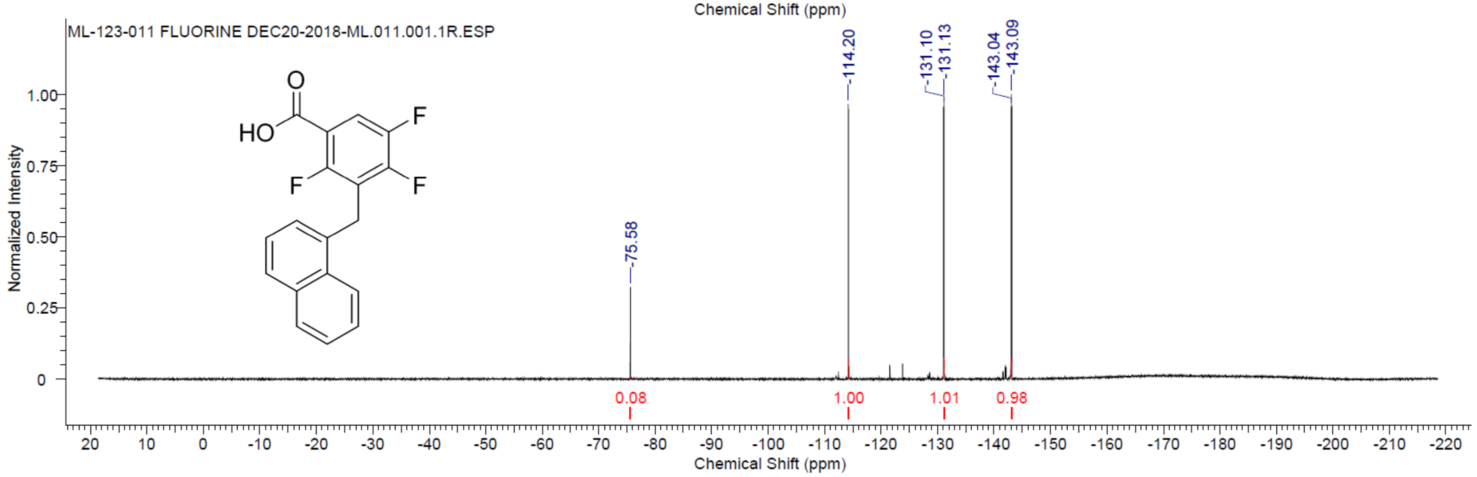

Compound 16– ^{1}H spectrum

This report was created by ACD/NMR Processor Academic Edition. For more information go to www.acdlabs.com/nmrproc/

15/11/2025 17:40:45

**Acquisition Time (sec)**	5.4526	**Comment**	ML-123-012 CDCl3			**Date**	28 May 2019 22:47:44
**Date Stamp**	28 May 2019 22:47:44			**File Name**	\\137.73.205.115\data\ML\nmr\May28-2019-ML\10\PDATA\1\1r		
**Frequency (MHz)**	400.13	**Nucleus**	1H	**Number of Transients**	16	**Origin**	spect
**Original Points Count**	65536	**Owner**	nmrsu	**Points Count**	131072	**Pulse Sequence**	zg30
**Receiver Gain**	20.03	**SW(cyclical) (Hz)**	12019.23	**Solvent**	CHLOROFORM-d		
**Spectrum Offset (Hz)**	2408.6333	**Spectrum Type**	STANDARD	**Sweep Width (Hz)**	12019.14	**Temperature (degree C)**	25.405

^{1}H NMR (400 MHz, CHLOROFORM-d) δ 10.72 (d, *J* = 13.48 Hz, 1H), 8.00 - 8.11 (m, 2H), 7.73 (d, *J* = 7.98 Hz, 1H), 7.61 (d, *J* = 8.16 Hz, 1H), 7.40 - 7.49 (m, 1H), 7.32 - 7.40 (m, 1H), 7.24 (t, *J* = 7.66 Hz, 1H), 7.06 - 7.14 (m, 1H), 6.98 - 7.06 (m, 1H), 4.37 (s, 2H), 3.78 (q, *J* = 7.15 Hz, 1H), 3.60 (q, *J* = 7.09 Hz, 1H), 2.69 - 2.83 (m, 1H), 0.80 (t, *J* = 7.15 Hz, 2H), 0.58 - 0.75 (m, 4H), 0.55 (t, *J* = 7.11 Hz, 1H)

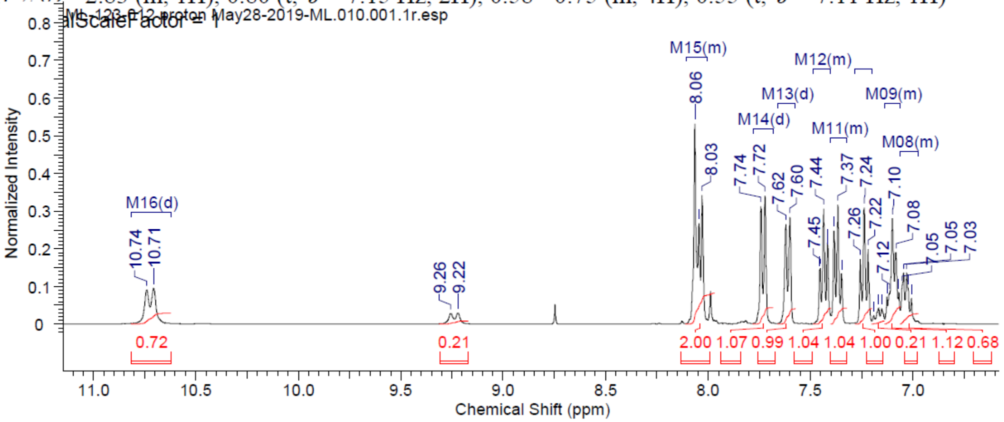

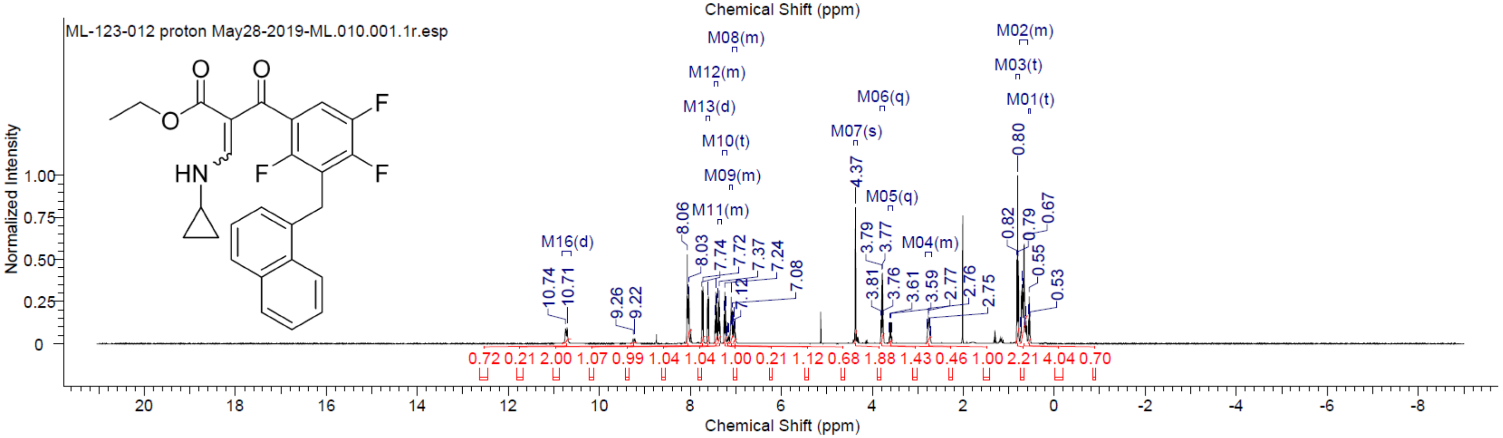

Compound 16– ^{13}C spectrum

This report was created by ACD/NMR Processor Academic Edition. For more information go to www.acdlabs.com/nmrproc/

15/11/2025 14:02:01

Acquisition Time (sec)	2.3809	***Comment***	ML-123-012 CDCl3			***Date***	29 May 2019 01:12:48
Date Stamp	29 May 2019 01:12:48			***File Name***	\\137.73.205.115\data\ML\nmr\May28-2019-ML\16\PDATA\1\1r		
Frequency (MHz)	100.61	***Nucleus***	13C	***Number of Transients***	1024	***Origin***	spect
Original Points Count	59522	***Owner***	nmrsu	***Points Count***	131072	***Pulse Sequence***	zgpg30
Receiver Gain	211.17	***SW(cyclical) (Hz)***	25000.00	***Solvent***	CHLOROFORM-d		
Spectrum Offset (Hz)	11047.5645	***Spectrum Type***	STANDARD	***Sweep Width (Hz)***	24999.81	***Temperature (degree C)***	25.549

^{13}C NMR (101 MHz, CHLOROFORM-d) δ 188.3, 186.2, 168.2, 166.5, 160.5, 160.1, 154.9, 154.8, 154.8, 154.8, 154.2, 154.2, 154.2, 154.1, 152.4, 152.4, 152.4, 152.3, 151.8, 151.8, 151.7, 151.7, 151.0, 150.9, 150.8, 150.8, 150.7, 150.6, 150.6, 150.5, 148.5, 148.4, 148.2, 148.1, 148.1, 148.0, 148.0, 147.9, 147.8, 145.6, 145.5, 145.4, 145.4, 134.3, 133.7, 133.6, 133.4, 133.4, 131.5, 128.7, 128.6, 127.4, 127.2, 127.1, 127.0, 127.0, 126.9, 126.9, 126.8, 126.8, 126.2, 126.1, 125.9, 125.8, 125.6, 125.5, 125.3, 125.2, 123.1, 117.5, 117.3, 117.3, 117.2, 117.2, 117.1, 117.1, 116.9, 115.2, 115.2, 115.0, 115.0, 114.4. 114.3, 114.2, 114.1, 101.7, 59.6, 59.3, 30.7, 30.3, 29.9, 25.6, 13.8, 13.3, 6.3

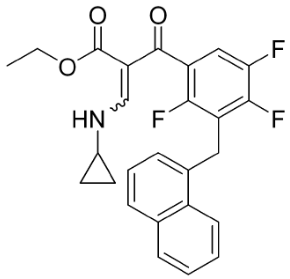

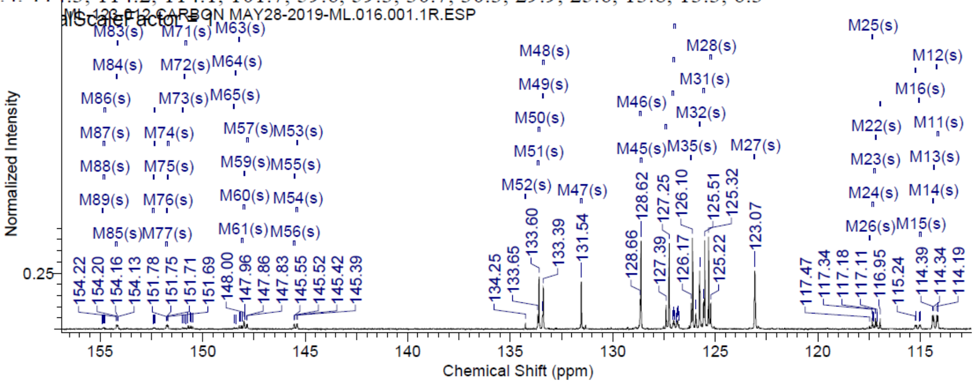

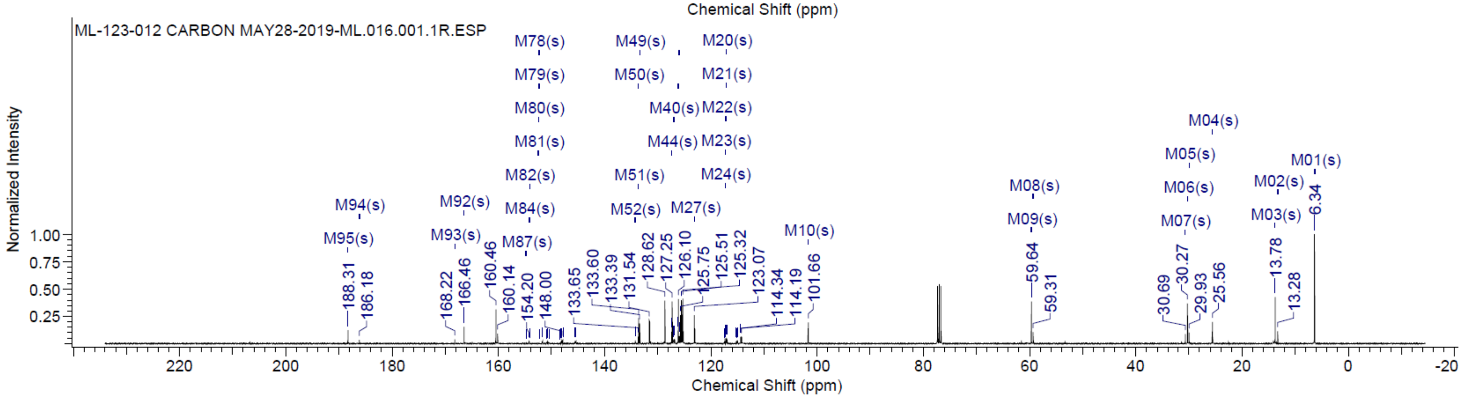

Compound 16– proton-decoupled ^{19}F spectrum

This report was created by ACD/NMR Processor Academic Edition. For more information go to www.acdlabs.com/nmrproc/

16/11/2025 11:12:26

Acquisition Time (sec)	1.4680	***Comment***	ML-123-012 CDCl3			***Date***	28 May 2019 22:56:16
Date Stamp	28 May 2019 22:56:16			***File Name***	\\137.73.205.115\data\ML\nmr\May28-2019-ML\12\PDATA\1\1r		
Frequency (MHz)	376.50	***Nucleus***	19F	***Number of Transients***	16	***Origin***	spect
Original Points Count	131072	***Owner***	nmrsu	***Points Count***	262144	***Pulse Sequence***	zg30
Receiver Gain	211.17	***SW(cyclical) (Hz)***	89285.71	***Solvent***	CHLOROFORM-d		
Spectrum Offset (Hz)	-37648.4063	***Spectrum Type***	STANDARD	***Sweep Width (Hz)***	89285.37	***Temperature (degree C)***	25.380

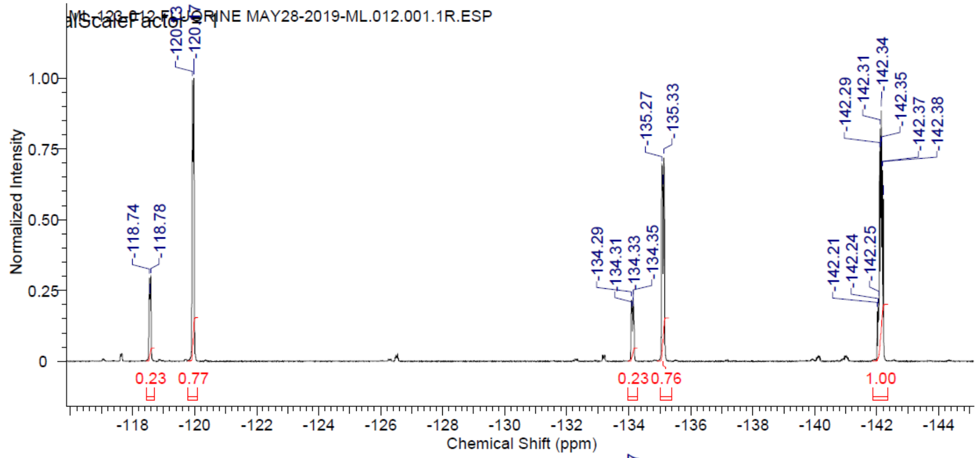

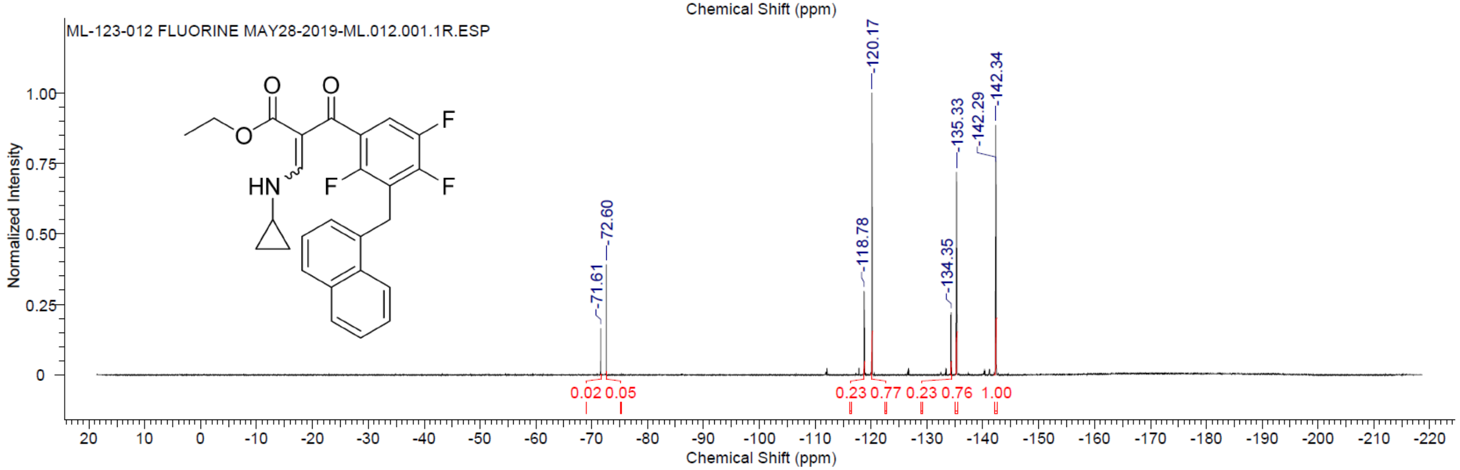

Compound 17– ^{1}H spectrum

This report was created by ACD/NMR Processor Academic Edition. For more information go to www.acdlabs.com/nmrproc/

15/11/2025 17:42:54

Acquisition Time (sec)	5.4526	***Comment***	ML-123-015 CDCl3			***Date***	29 May 2019 17:19:12
Date Stamp	29 May 2019 17:19:12			***File Name***	\\137.73.205.115\data\ML\nmr\May29-2019-ML\10\PDATA\1\1r		
Frequency (MHz)	400.13	***Nucleus***	1H	***Number of Transients***	16	***Origin***	spect
Original Points Count	65536	***Owner***	nmrsu	***Points Count***	131072	***Pulse Sequence***	zg30
Receiver Gain	116.97	***SW(cyclical) (Hz)***	12019.23	***Solvent***	CHLOROFORM-d		
Spectrum Offset (Hz)	2465.3491	***Spectrum Type***	STANDARD	***Sweep Width (Hz)***	12019.14	***Temperature (degree C)***	26.086

^{1}H NMR (400 MHz, CHLOROFORM-d) δ 8.50 (s, 1H), 8.37 (t, *J* = 9.54 Hz, 1H), 8.13 (d, *J* = 8.34 Hz, 1H), 7.93 (d, *J* = 7.70 Hz, 1H), 7.76 (d, *J* = 8.34 Hz, 1H), 7.53 - 7.68 (m, 2H), 7.27 (d, *J* = 15.41 Hz, 1H), 6.47 (d, *J* = 7.15 Hz, 1H), 4.39 (q, *J* = 7.09 Hz, 2H), 3.31 - 3.42 (m, 1H), 1.40 (t, *J* = 7.15 Hz, 3H), 0.94 - 1.08 (m, 4H)

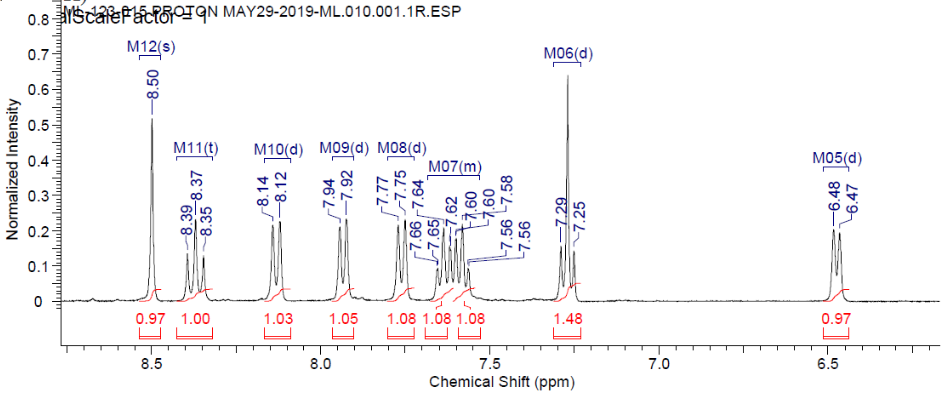

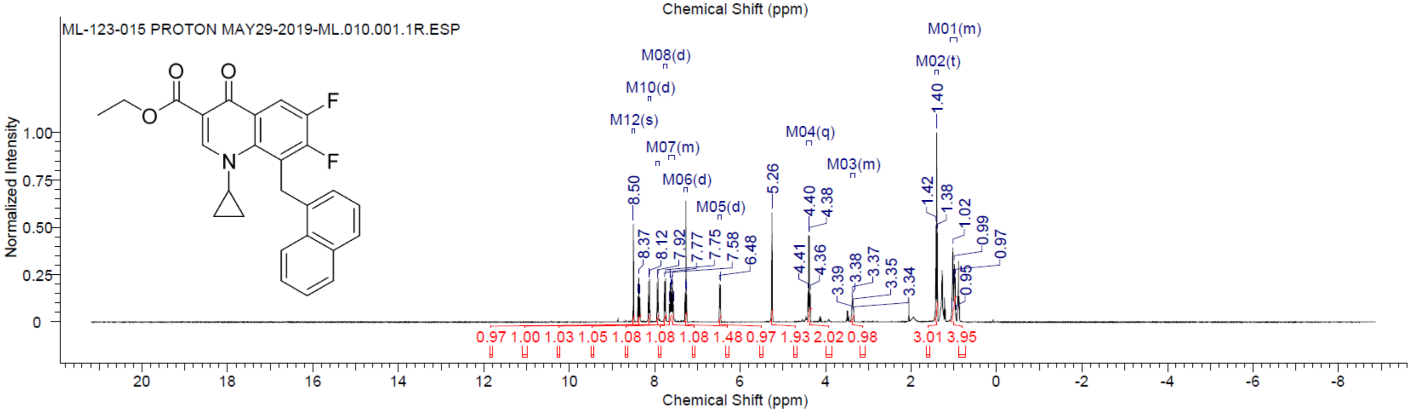

Compound 17– ^{13}C spectrum

This report was created by ACD/NMR Processor Academic Edition. For more information go to www.acdlabs.com/nmrproc/

15/11/2025 14:06:02

Acquisition Time (sec)	2.3809	***Comment***	ML-123-015 CDCl3			***Date***	29 May 2019 21:43:44
Date Stamp	29 May 2019 21:43:44			***File Name***	\\137.73.205.115\data\ML\nmr\May29-2019-ML\16\PDATA\1\1r		
Frequency (MHz)	100.61	***Nucleus***	13C	***Number of Transients***	1024	***Origin***	spect
Original Points Count	59522	***Owner***	nmrsu	***Points Count***	131072	***Pulse Sequence***	zgpg30
Receiver Gain	211.17	***SW(cyclical) (Hz)***	25000.00	***Solvent***	CHLOROFORM-d		
Spectrum Offset (Hz)	11062.4414	***Spectrum Type***	STANDARD	***Sweep Width (Hz)***	24999.81	***Temperature (degree C)***	25.605

^{13}C NMR (101 MHz, CHLOROFORM-d) δ 172.9, 165.1, 151.8, 150.0, 149.8, 147.5, 147.3, 138.0, 138.0, 138.0, 138.0, 133.9, 133.8, 131.0, 129.2, 127.6, 126.8, 126.1, 125.7, 123.7, 122.1, 118.7, 118.6, 114.0, 114.0, 113.9, 113.8, 110.7, 61.1, 38.3, 28.4, 28.3, 14.4, 11.2

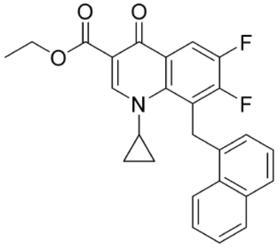

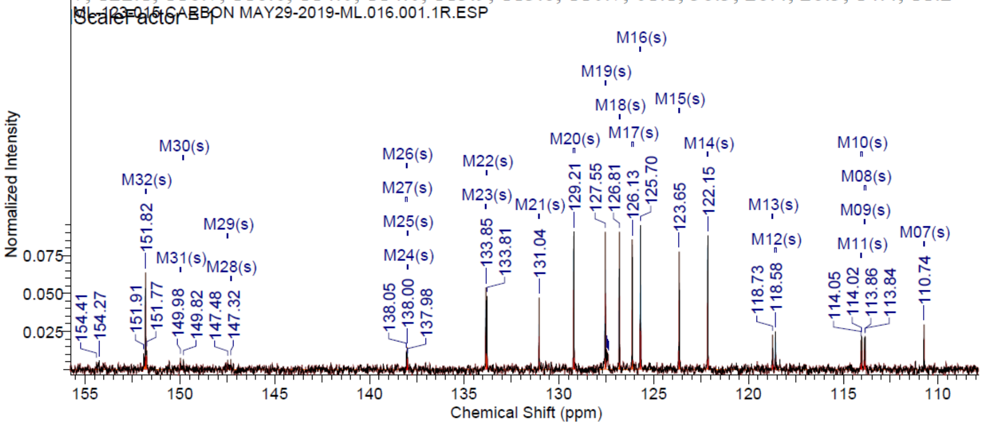

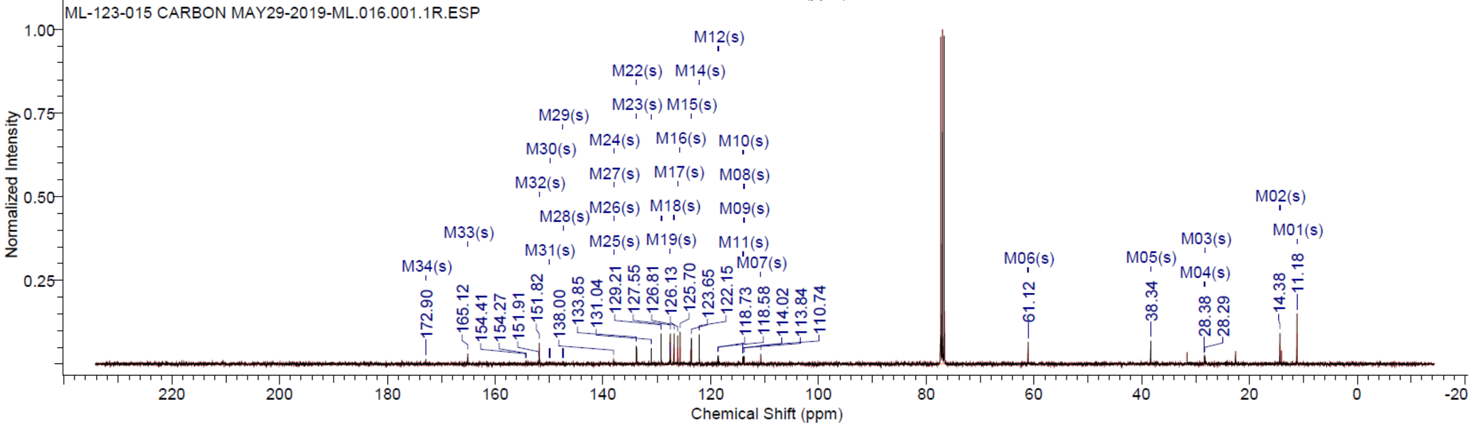

Compound 17– proton-decoupled ^{19}F spectrum

This report was created by ACD/NMR Processor Academic Edition. For more information go to www.acdlabs.com/nmrproc/

16/11/2025 11:17:09

Acquisition Time (sec)	1.4680	***Comment***	ML-123-015 CDCl3			***Date***	29 May 2019 17:27:44
Date Stamp	29 May 2019 17:27:44			***File Name***	\\137.73.205.115\data\ML\nmr\May29-2019-ML\13\PDATA\1\1r		
Frequency (MHz)	376.50	***Nucleus***	19F	***Number of Transients***	16	***Origin***	spect
Original Points Count	131072	***Owner***	nmrsu	***Points Count***	262144	***Pulse Sequence***	zgig30
Receiver Gain	211.17	***SW(cyclical) (Hz)***	89285.71	***Solvent***	CHLOROFORM-d		
Spectrum Offset (Hz)	-37648.4180	***Spectrum Type***	STANDARD	***Sweep Width (Hz)***	89285.37	***Temperature (degree C)***	26.351

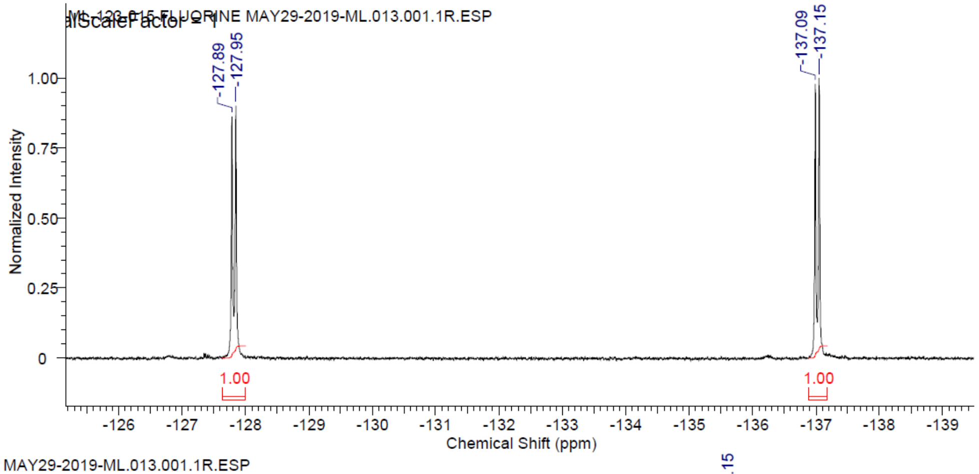

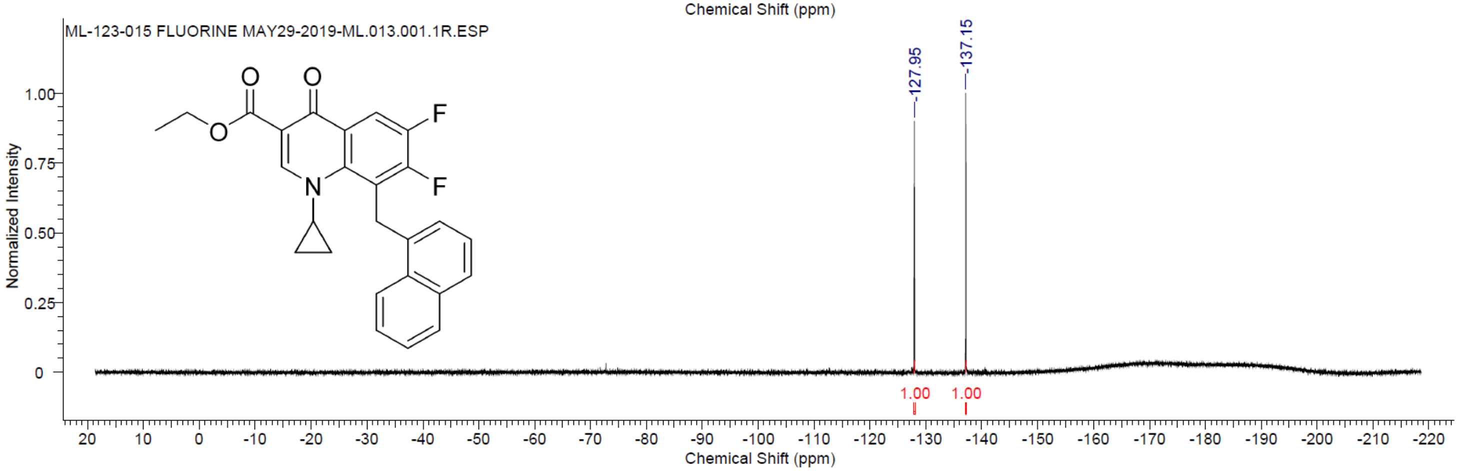

Compound 18– ^{1}H spectrum

This report was created by ACD/NMR Processor Academic Edition. For more information go to www.acdlabs.com/nmrproc/

15/11/2025 17:44:41

Acquisition Time (sec)	5.4526	*Comment*	ML-123-017 CDCl3			*Date*	31 May 2019 19:59:12
Date Stamp	31 May 2019 19:59:12			*File Name*	\\137.73.205.115\data\ML\nmr\May31-2019-ML\10\PDATA\1\1r		
Frequency (MHz)	400.13	*Nucleus*	1H	*Number of Transients*	16	*Origin*	spect
Original Points Count	65536	*Owner*	nmrsu	*Points Count*	131072	*Pulse Sequence*	zg30
Receiver Gain	116.97	*SW(cyclical) (Hz)*	12019.23	*Solvent*	CHLOROFORM-d		
Spectrum Offset (Hz)	2465.4409	*Spectrum Type*	STANDARD	*Sweep Width (Hz)*	12019.14	*Temperature (degree C)*	27.662

^{1}H NMR (400 MHz, CHLOROFORM-d) δ 14.36 (br. s., 1H), 8.76 (s, 1H), 8.40 (t, *J* = 9.17 Hz, 1H), 8.13 (d, *J* = 8.44 Hz, 1H), 7.95 (d, *J* = 7.79 Hz, 1H), 7.78 (d, *J* = 8.25 Hz, 1H), 7.63 - 7.71 (m, 1H), 7.55 - 7.63 (m, 1H), 7.23 - 7.31 (m, 2H), 6.43 (d, *J* = 7.15 Hz, 1H), 5.33 (s, 2H), 3.46 - 3.57 (m, 1H), 1.09 - 1.16 (m, 2H), 1.00 - 1.09 (m, 2H)

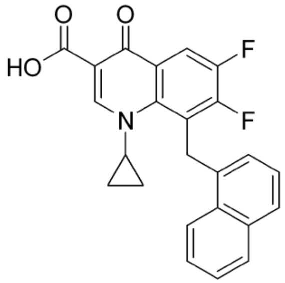

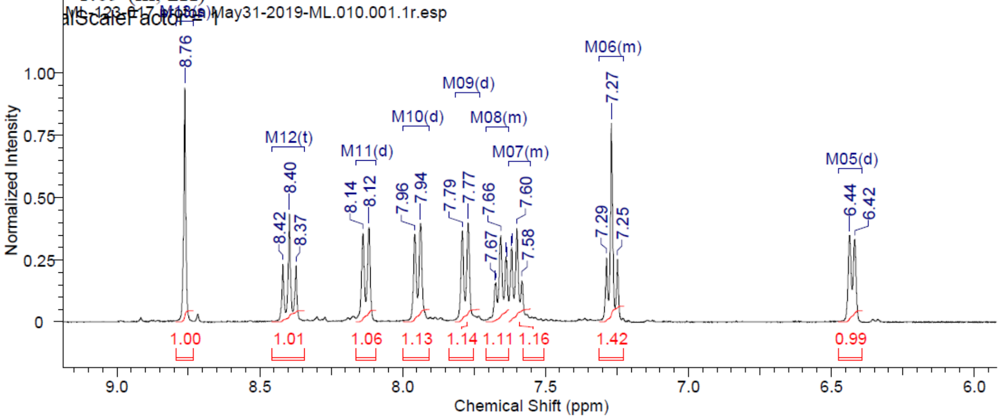

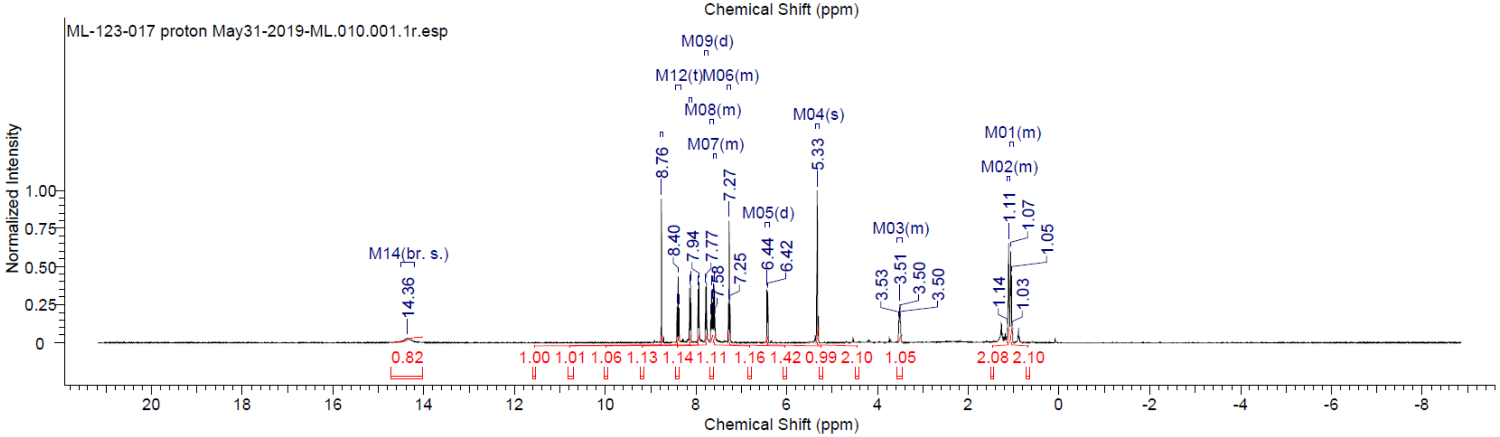

Compound 18– ^{13}C spectrum

This report was created by ACD/NMR Processor Academic Edition. For more information go to www.acdlabs.com/nmrproc/

15/11/2025 14:35:06

Acquisition Time (sec)	2.3809	***Comment***	ML-123-017 CDCl3			***Date***	31 May 2019 22:26:24
Date Stamp	31 May 2019 22:26:24			***File Name***	\\137.73.205.115\data\ML\nmr\May31-2019-ML\16\PDATA\1\1r		
Frequency (MHz)	100.61	***Nucleus***	13C	***Number of Transients***	1024	***Origin***	spect
Original Points Count	59522	***Owner***	nmrsu	***Points Count***	131072	***Pulse Sequence***	zgpg30
Receiver Gain	211.17	***SW(cyclical) (Hz)***	25000.00	***Solvent***	CHLOROFORM-d		
Spectrum Offset (Hz)	11063.0137	***Spectrum Type***	STANDARD	***Sweep Width (Hz)***	24999.81	***Temperature (degree C)***	27.507

^{13}C NMR (101 MHz, CHLOROFORM-d) δ 177.4, 177.4, 165.9, 155.4, 155.2, 152.9, 152.7, 151.4, 150.5, 150.4, 148.0, 147.8, 138.9, 138.9, 138.9, 138.9, 133.9, 133.4, 130.9, 129.3, 127.9, 127.0, 126.3, 125.6, 123.5, 122.0, 119.9, 119.8, 113.4, 113.3, 113.2, 113.1, 108.6, 39.4, 28.6, 28.6, 28.5, 28.5, 11.4

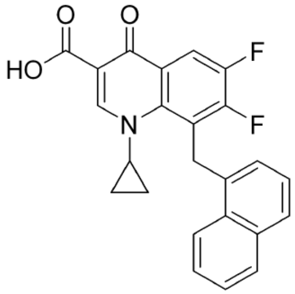

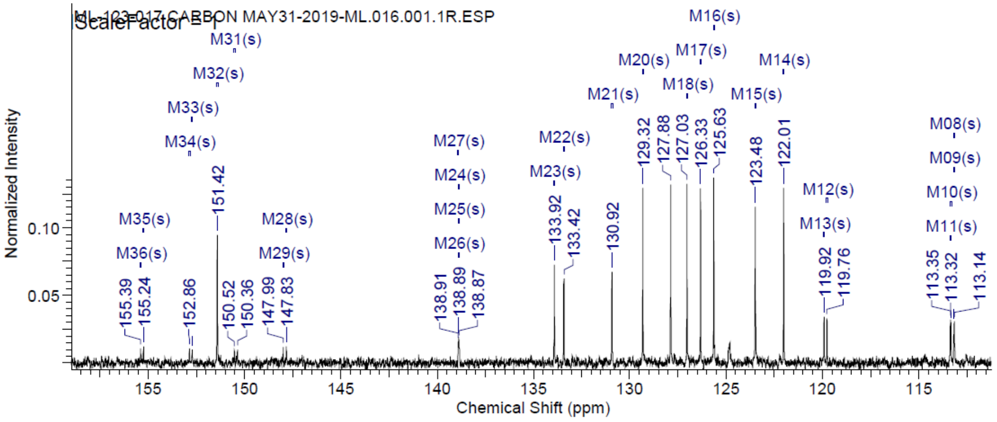

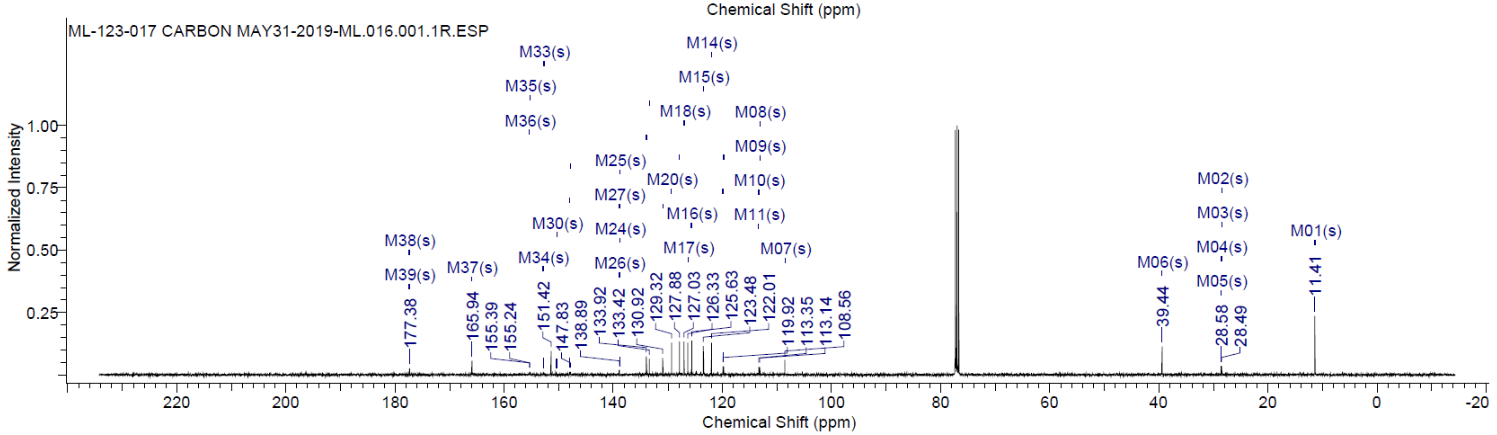

Compound 18– proton-decoupled ^{19}F spectrum

This report was created by ACD/NMR Processor Academic Edition. For more information go to www.acdlabs.com/nmrproc/

16/11/2025 11:19:33

**Acquisition Time (sec)**	1.4680	**Comment**	ML-123-017 CDCl3	**Date**	31 May 2019 20:09:52
**Date Stamp**	31 May 2019 20:09:52	**File Name**	\\137.73.205.115\data\ML\nmr\May31-2019-ML\13\PDATA\1\1r		
**Frequency (MHz)**	376.50	**Nucleus**	19F	**Number of Transients**	16
**Origin**	spect				
**Original Points Count**	131072	**Owner**	nmrsu	**Points Count**	262144
**Pulse Sequence**	zgig30				
**Receiver Gain**	211.17	**SW(cyclical) (Hz)**	89285.71	**Solvent**	CHLOROFORM-d
**Spectrum Offset (Hz)**	-37648.4180	**Spectrum Type**	STANDARD	**Sweep Width (Hz)**	89285.37
**Temperature (degree C)**	27.754				

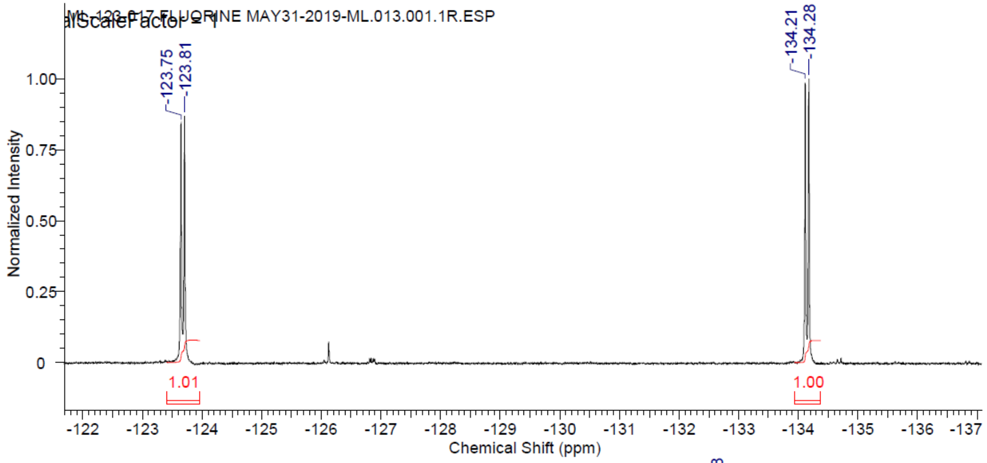

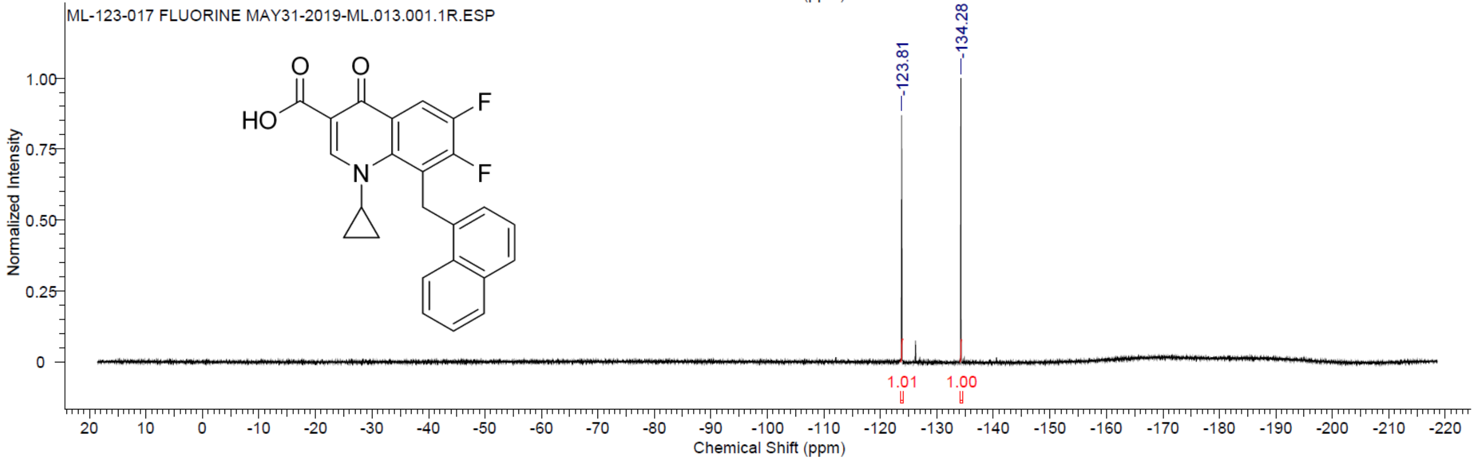

Compound 19– ^{1}H spectrum

This report was created by ACD/NMR Processor Academic Edition. For more information go to www.acdlabs.com/nmrproc/

15/11/2025 18:07:07

Acquisition Time (sec)	5.4526	***Comment***	ML-123-021 DMSO-d6 dried, more concentrated			***Date***	08 Jul 2019 18:59:28
Date Stamp	08 Jul 2019 18:59:28			***File Name***	\\137.73.205.115\data\ML\nmr\Jul08-2019-ML\20\PDATA\1\1r		
Frequency (MHz)	400.13	***Nucleus***	1H	***Number of Transients***	16	***Origin***	spect
Original Points Count	65536	***Owner***	nmrsu	***Points Count***	131072	***Pulse Sequence***	zg30
Receiver Gain	126.48	***SW(cyclical) (Hz)***	12019.23	***Solvent***	DMSO-d6	***Spectrum Offset (Hz)***	2471.1484
Spectrum Type	STANDARD	***Sweep Width (Hz)***	12019.14	***Temperature (degree C)***	28.838		

^{1}H NMR (400 MHz, DMSO-d_6) δ 8.73 (s, 1H), 8.37 (d, *J* = 8.16 Hz, 1H), 8.08 (d, *J* = 11.74 Hz, 1H), 7.97 (d, *J* = 8.53 Hz, 2H), 7.75 (d, *J* = 8.25 Hz, 1H), 7.64 (d, *J* = 8.16 Hz, 1H), 7.60 (d, *J* = 7.61 Hz, 1H), 7.22 (t, *J* = 7.70 Hz, 1H), 6.25 (d, *J* = 7.06 Hz, 1H), 5.44 (br. s., 2H), 3.74 (br. s., 2H), 2.89 (br. s., 4H), 1.16 (d, *J* = 5.87 Hz, 2H), 1.01 (br. s., 2H)

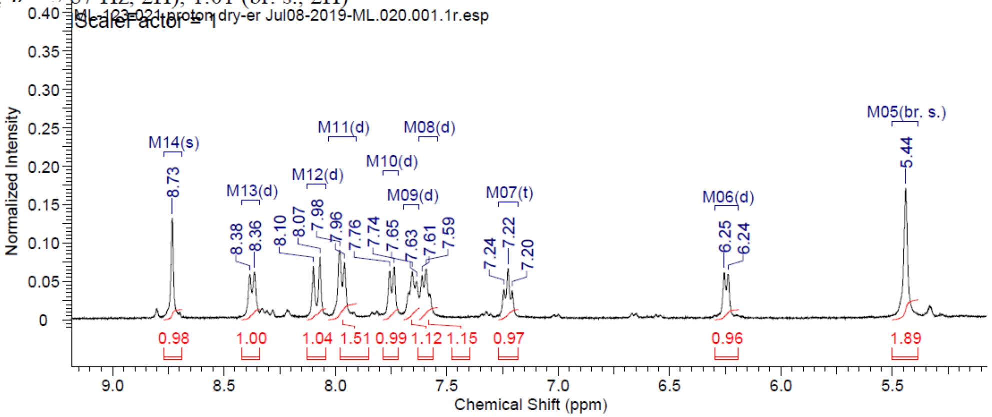

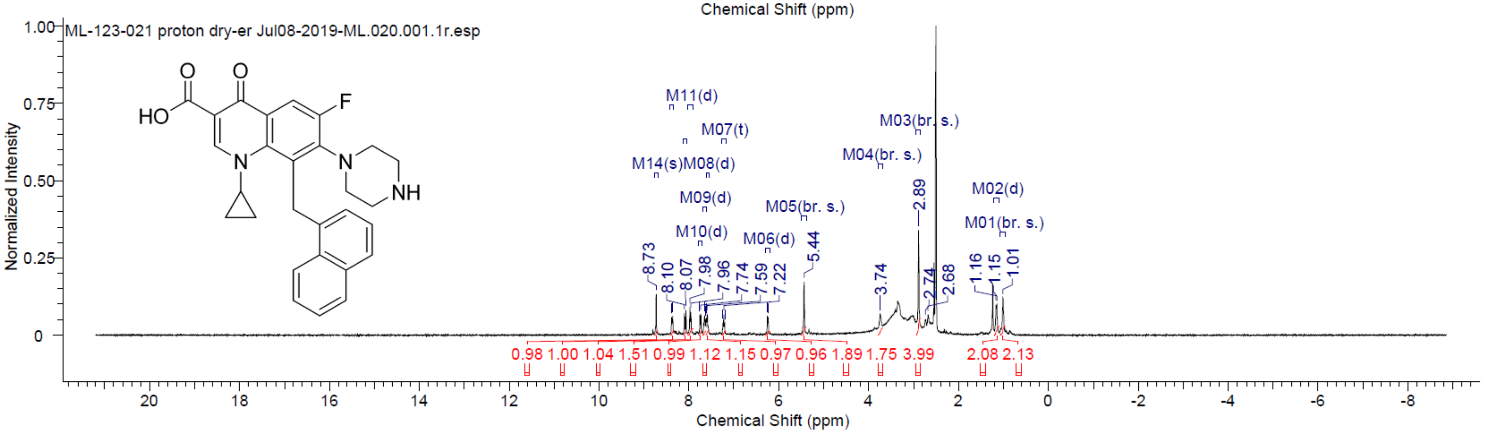

Compound 19 (+ drop of TFA) – ^{1}H spectrum

This report was created by ACD/NMR Processor Academic Edition. For more information go to www.acdlabs.com/nmrproc/

15/11/2025 18:09:07

Acquisition Time (sec)	5.4526	***Comment***	ML-123-021 DMSO-d6 + drop of TFA			***Date***	09 Jul 2019 13:09:36
Date Stamp	09 Jul 2019 13:09:36			***File Name***	\\137.73.205.115\data\ML\nmr\Jul09-2019-ML\10\PDATA\1\1r		
Frequency (MHz)	400.13	***Nucleus***	1H	***Number of Transients***	16	***Origin***	spect
Original Points Count	65536	***Owner***	nmrsu	***Points Count***	131072	***Pulse Sequence***	zg30
Receiver Gain	102.38	***SW(cyclical) (Hz)***	12019.23	***Solvent***	DMSO-d6	***Spectrum Offset (Hz)***	2467.6538
Spectrum Type	STANDARD	***Sweep Width (Hz)***	12019.14	***Temperature (degree C)***	27.256		

^{1}H NMR (400 MHz, DMSO-d_6) δ 9.21 (br. s., 1H), 8.76 (s, 1H), 8.65 (br. s., 2H), 8.39 (d, *J* = 8.44 Hz, 1H), 8.11 (d, *J* = 11.65 Hz, 1H), 7.97 (d, *J* = 8.16 Hz, 1H), 7.75 (d, *J* = 7.98 Hz, 1H), 7.65 (t, *J* = 7.34 Hz, 1H), 7.60 (t, *J* = 7.24 Hz, 1H), 7.23 (t, *J* = 7.61 Hz, 1H), 6.29 (d, *J* = 7.52 Hz, 1H), 5.42 (br. s., 2H), 3.80 (br. s., 2H), 3.58 (d, *J* = 4.22 Hz, 2H), 3.32 (s, 6H), 3.13 (br. s., 2H), 3.07 (br. s., 2H), 1.23 (br. s., 2H), 1.13 (d, *J* = 6.14 Hz, 2H), 1.01 (br. s., 3H)

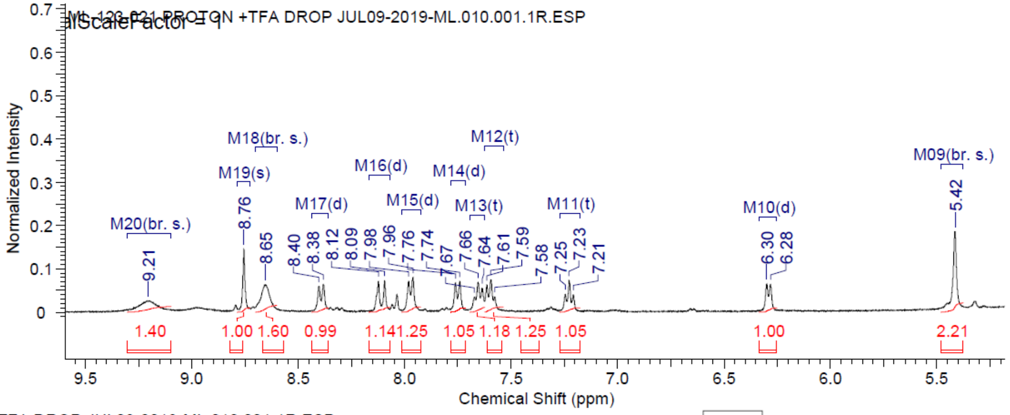

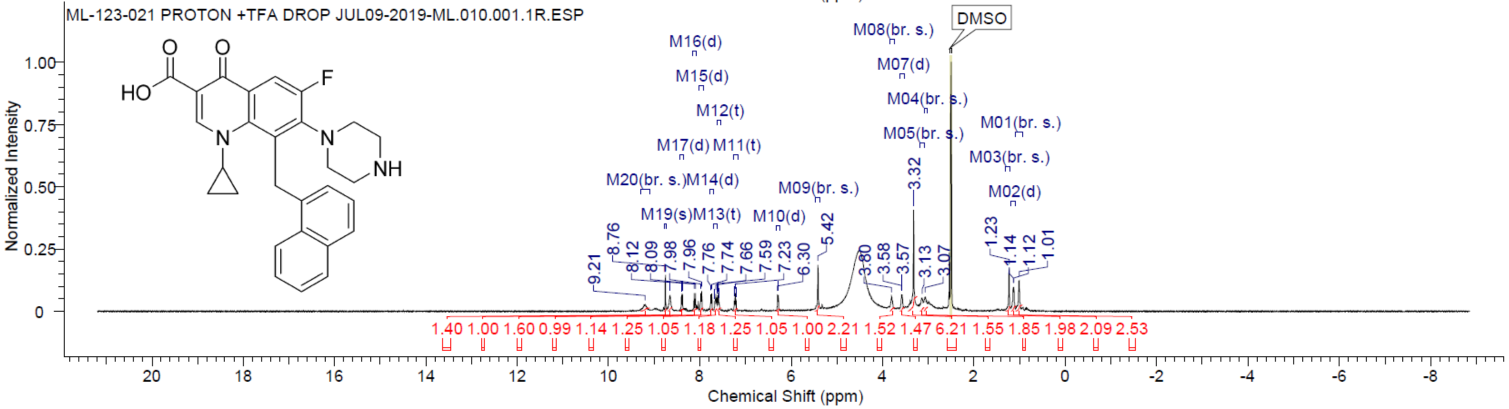

Compound 19– ^{13}C spectrum

This report was created by ACD/NMR Processor Academic Edition. For more information go to www.acdlabs.com/nmrproc/

15/11/2025 14:40:02

Acquisition Time (sec)	2.3809	***Comment***	ML-123-021 DMSO-d6 + drop of TFA			***Date***	10 Jul 2019 02:38:08
Date Stamp	10 Jul 2019 02:38:08			***File Name***	\\137.73.205.115\data\ML\nmr\Jul09-2019-ML\17\PDATA\1\1r		
Frequency (MHz)	100.61	***Nucleus***	13C	***Number of Transients***	4096	***Origin***	spect
Original Points Count	59522	***Owner***	nmrsu	***Points Count***	131072	***Pulse Sequence***	zgpg30
Receiver Gain	211.17	***SW(cyclical) (Hz)***	25000.00	***Solvent***	DMSO-d6	***Spectrum Offset (Hz)***	11018.9531
Spectrum Type	STANDARD	***Sweep Width (Hz)***	24999.81	***Temperature (degree C)***	26.910		

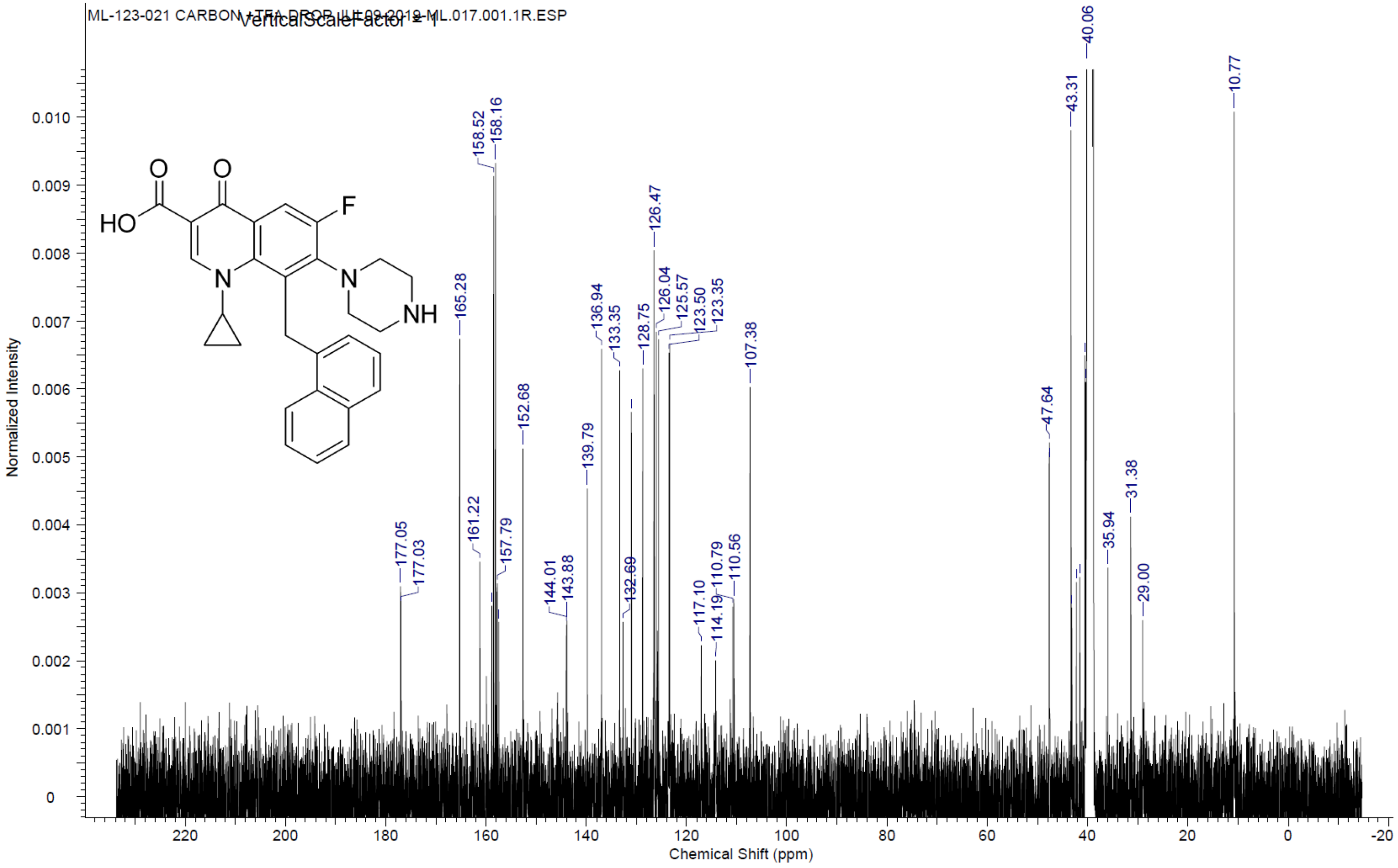

Compound 19 (+ drop of TFA) – ^{19}F spectrum

This report was created by ACD/NMR Processor Academic Edition. For more information go to www.acdlabs.com/nmrproc/

16/11/2025 11:22:47

Acquisition Time (sec)	1.4680	***Comment***	ML-123-021 DMSO-d6 + drop of TFA			***Date***	09 Jul 2019 13:11:44
Date Stamp	09 Jul 2019 13:11:44			***File Name***	\\137.73.205.115\data\ML\nmr\Jul09-2019-ML\11\PDATA\1\1r		
Frequency (MHz)	376.50	***Nucleus***	19F	***Number of Transients***	16	***Origin***	spect
Original Points Count	131072	***Owner***	nmrsu	***Points Count***	262144	***Pulse Sequence***	zg30
Receiver Gain	211.17	***SW(cyclical) (Hz)***	89285.71	***Solvent***	DMSO-d6	***Spectrum Offset (Hz)***	-37648.4063
Spectrum Type	STANDARD	***Sweep Width (Hz)***	89285.37	***Temperature (degree C)***	27.265		

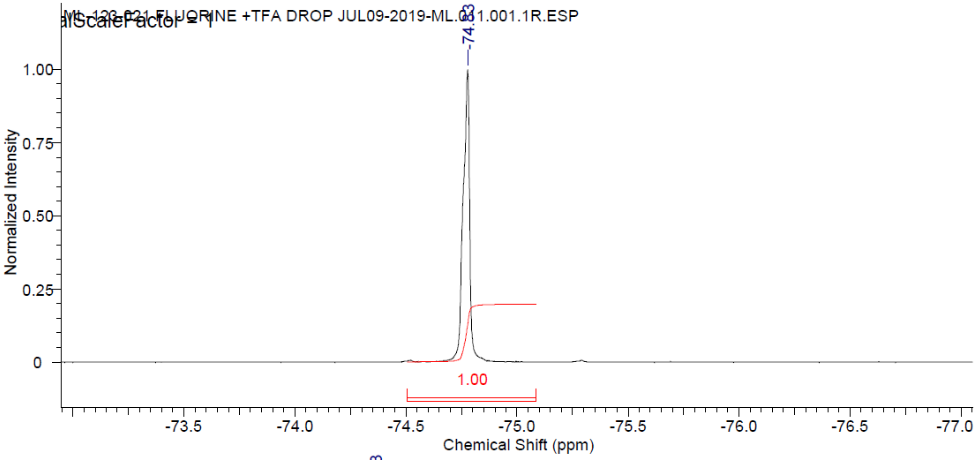

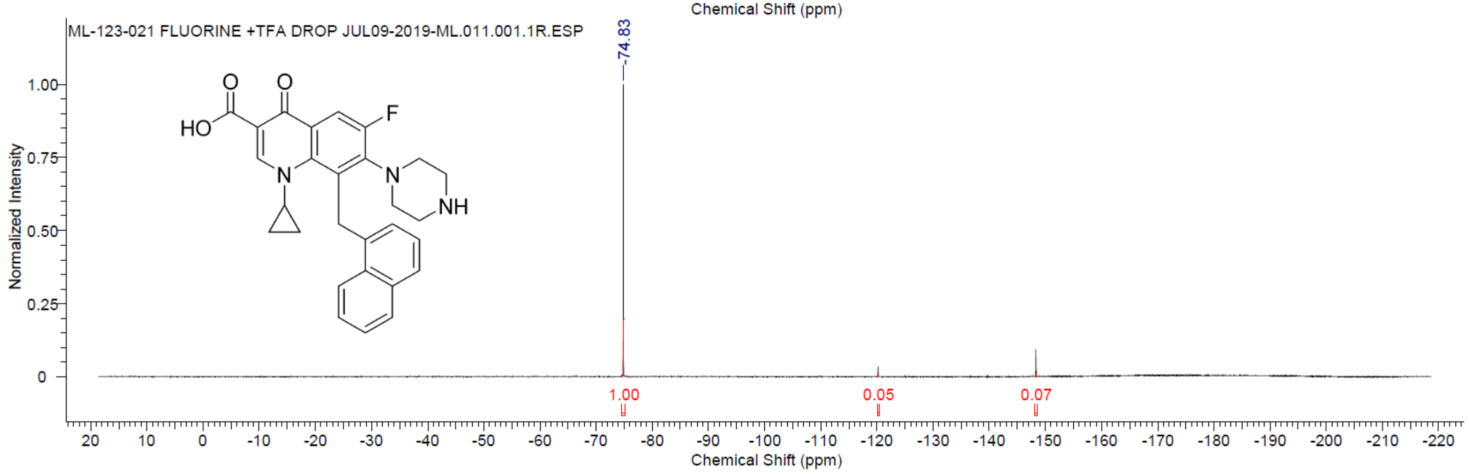

Compound 19 (+ drop of TFA) – proton-decoupled ^{19}F spectrum

This report was created by ACD/NMR Processor Academic Edition. For more information go to www.acdlabs.com/nmrproc/

16/11/2025 11:23:31

Acquisition Time (sec)	1.4680	*Comment*	ML-123-021 DMSO-d6 + drop of TFA			*Date*	09 Jul 2019 13:11:44
Date Stamp	09 Jul 2019 13:11:44			*File Name*	\\137.73.205.115\data\ML\nmr\Jul09-2019-ML\12\PDATA\1\1r		
Frequency (MHz)	376.50	*Nucleus*	19F	*Number of Transients*	16	*Origin*	spect
Original Points Count	131072	*Owner*	nmrsu	*Points Count*	262144	*Pulse Sequence*	zgig30
Receiver Gain	211.17	*SW(cyclical) (Hz)*	89285.71	*Solvent*	DMSO-d6	*Spectrum Offset (Hz)*	-37648.4180
Spectrum Type	STANDARD	*Sweep Width (Hz)*	89285.37	*Temperature (degree C)*	27.478		

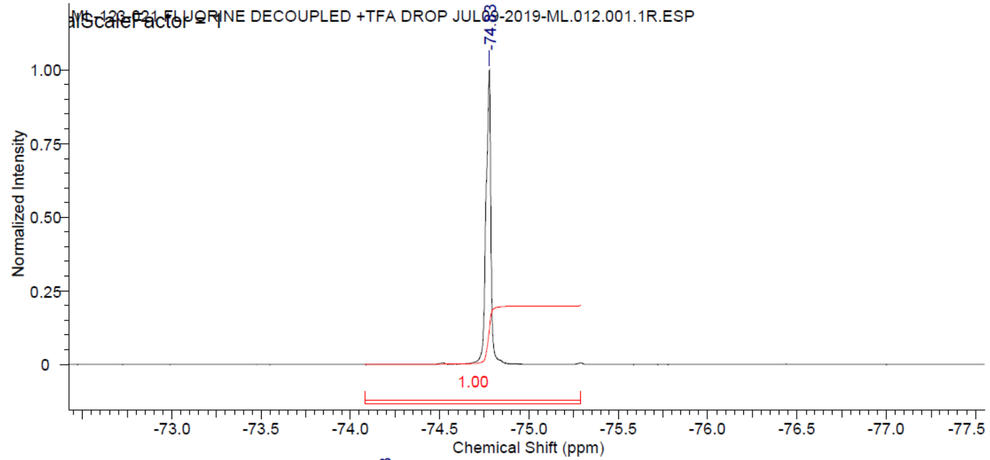

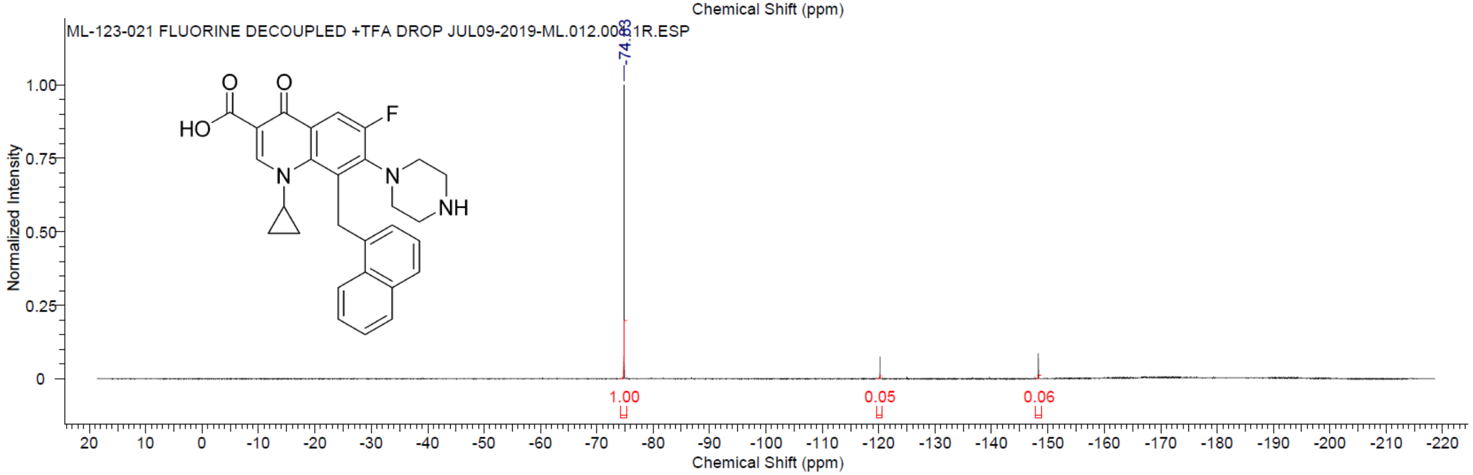

2.2.3 ^{1}H and ^{13}C spectrum for Wider First-Generation ERB-Fluoroquinolones

Compound 20-^{1}H spectrum

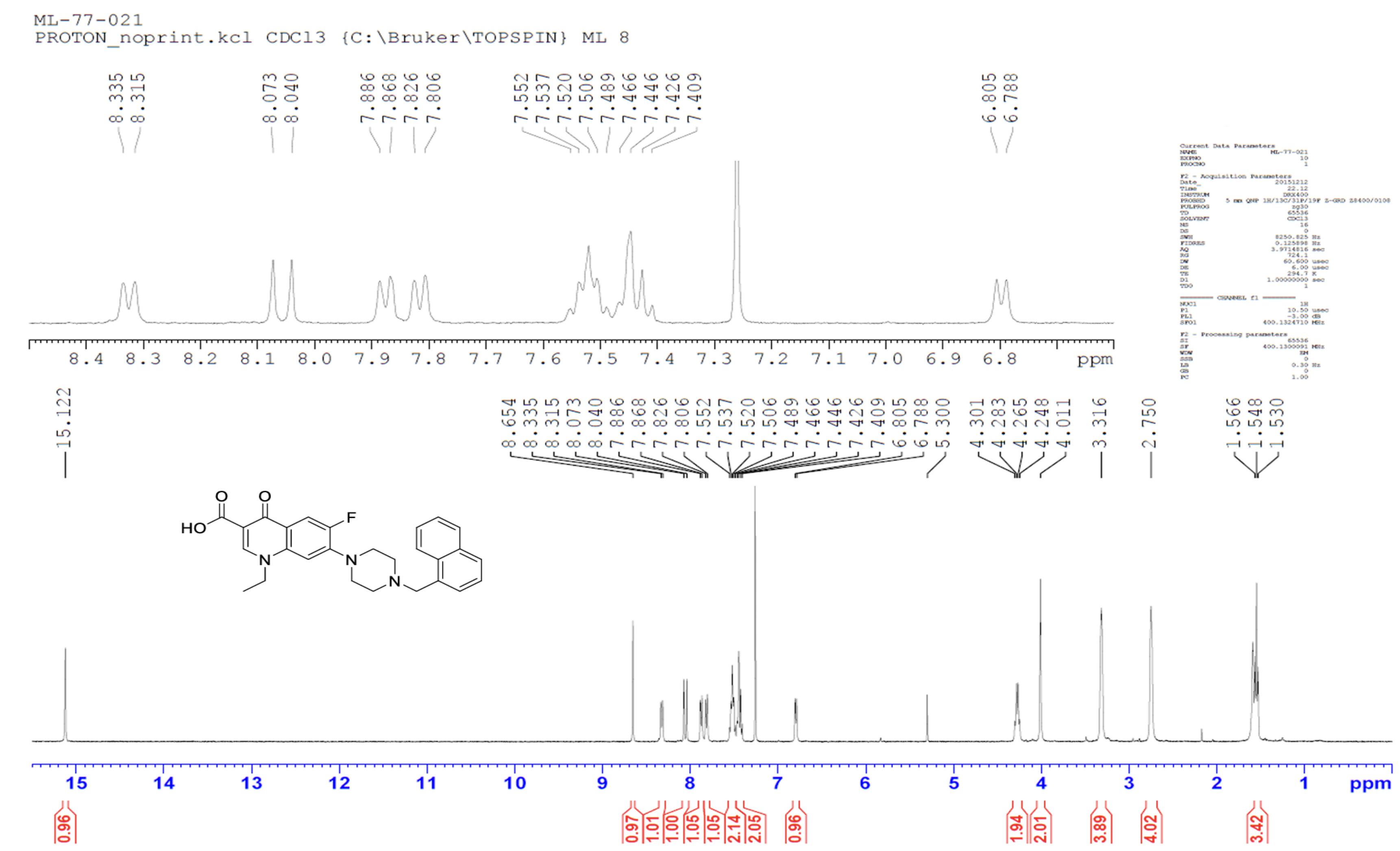

Compound 20– ^{13}C spectrum

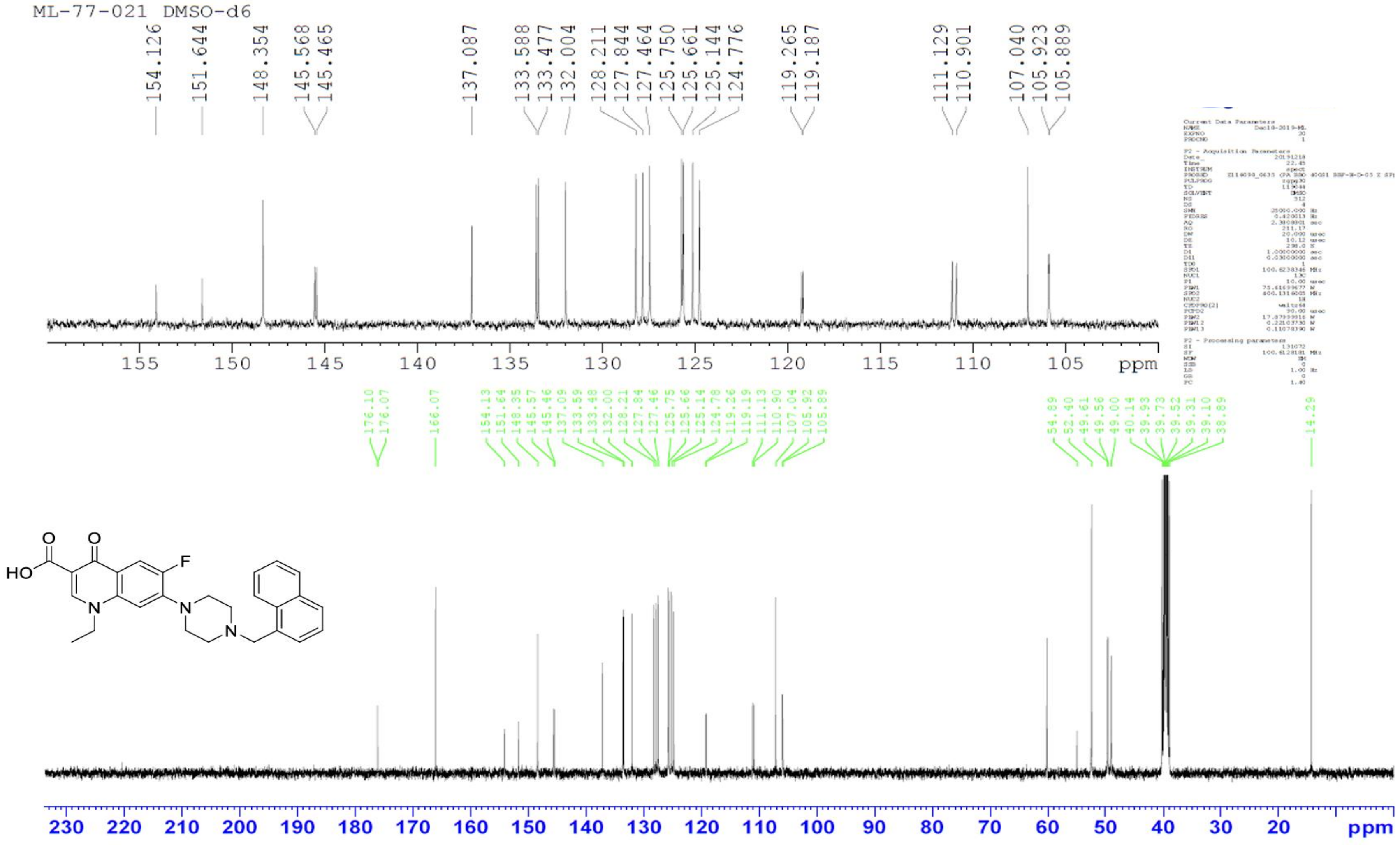

Compound 21– ^{1}H spectrum

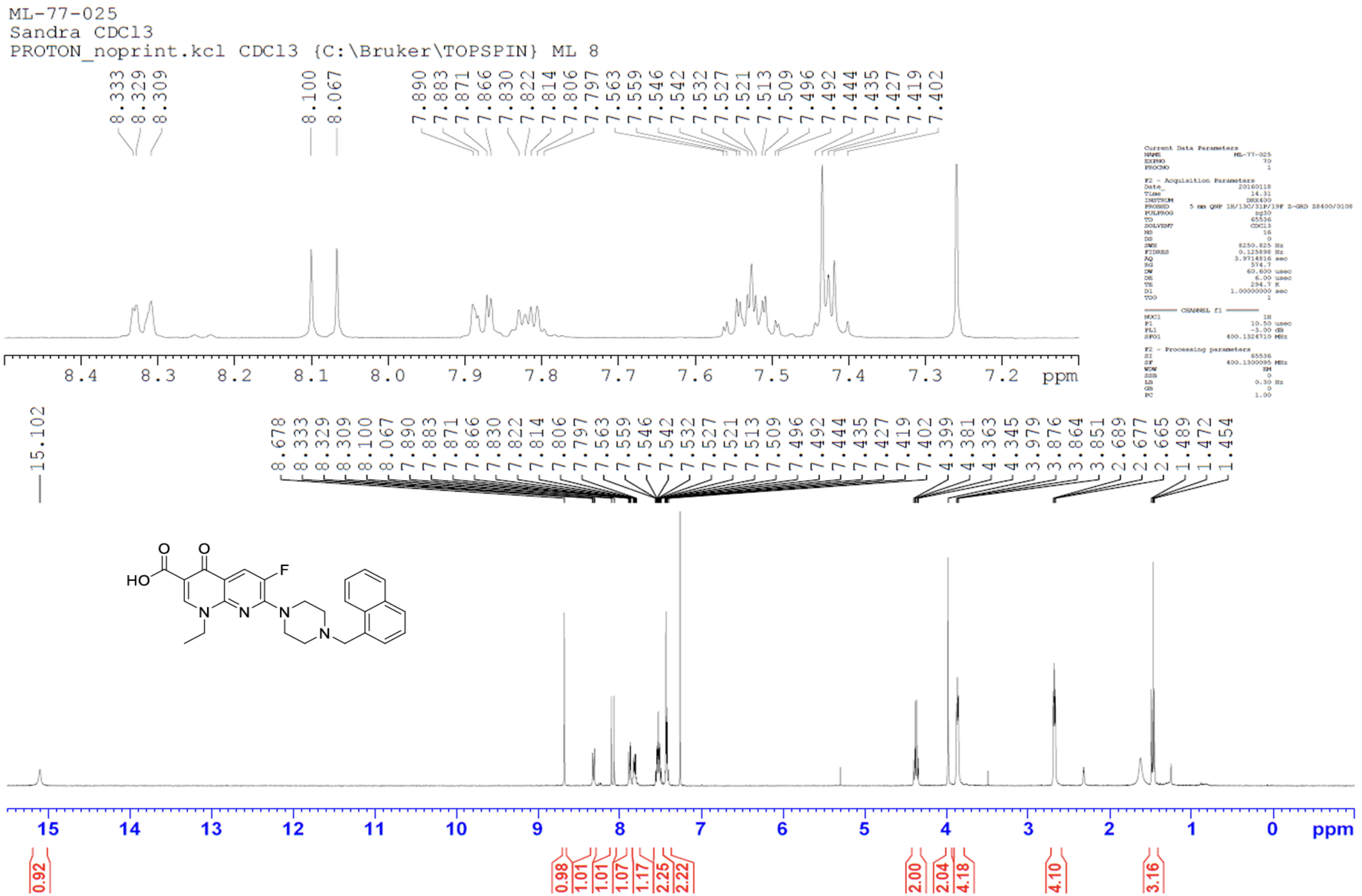

Compound 21 Salt – ^{13}C spectrum

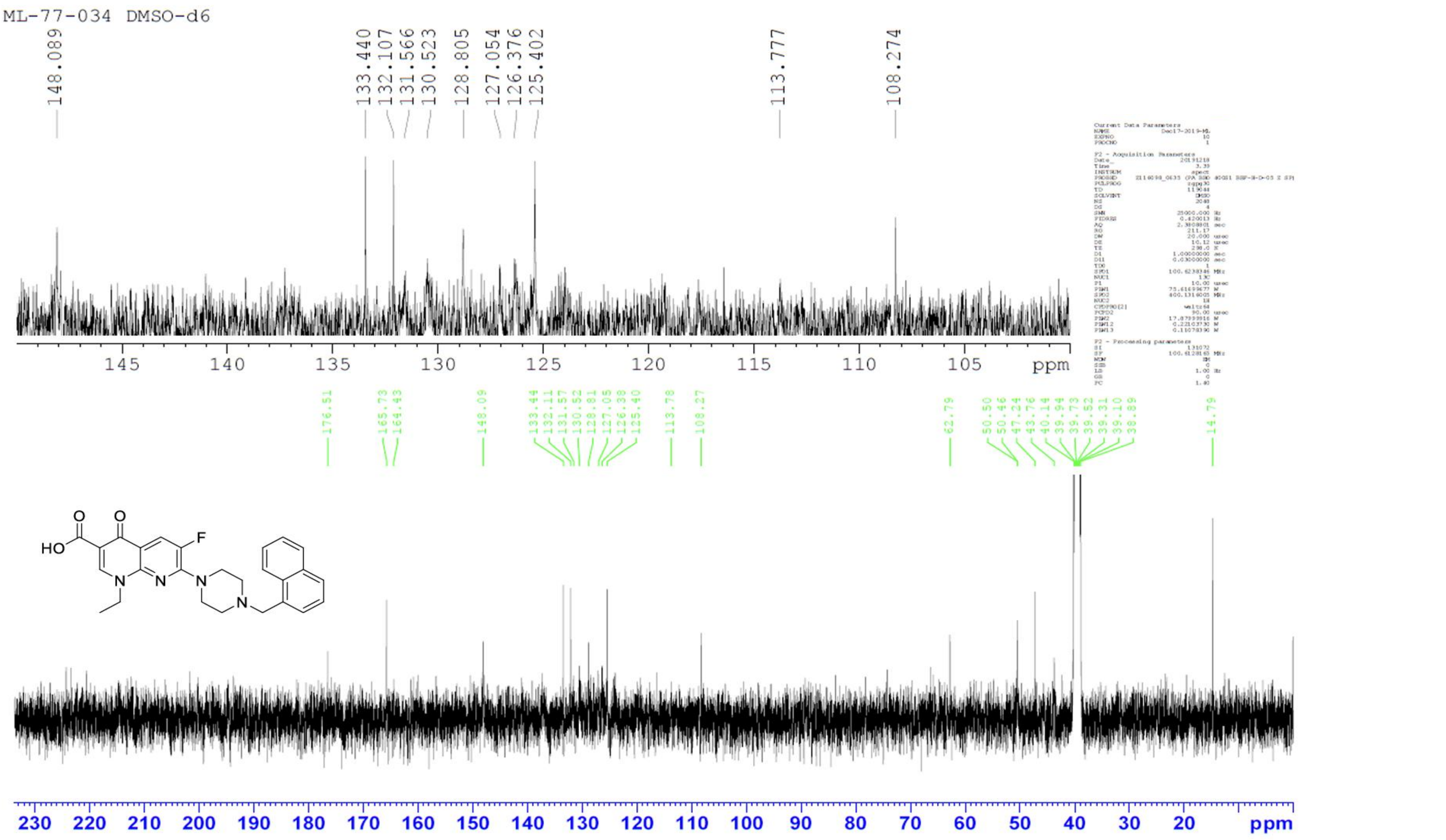

Compound 23– ^{1}H spectrum

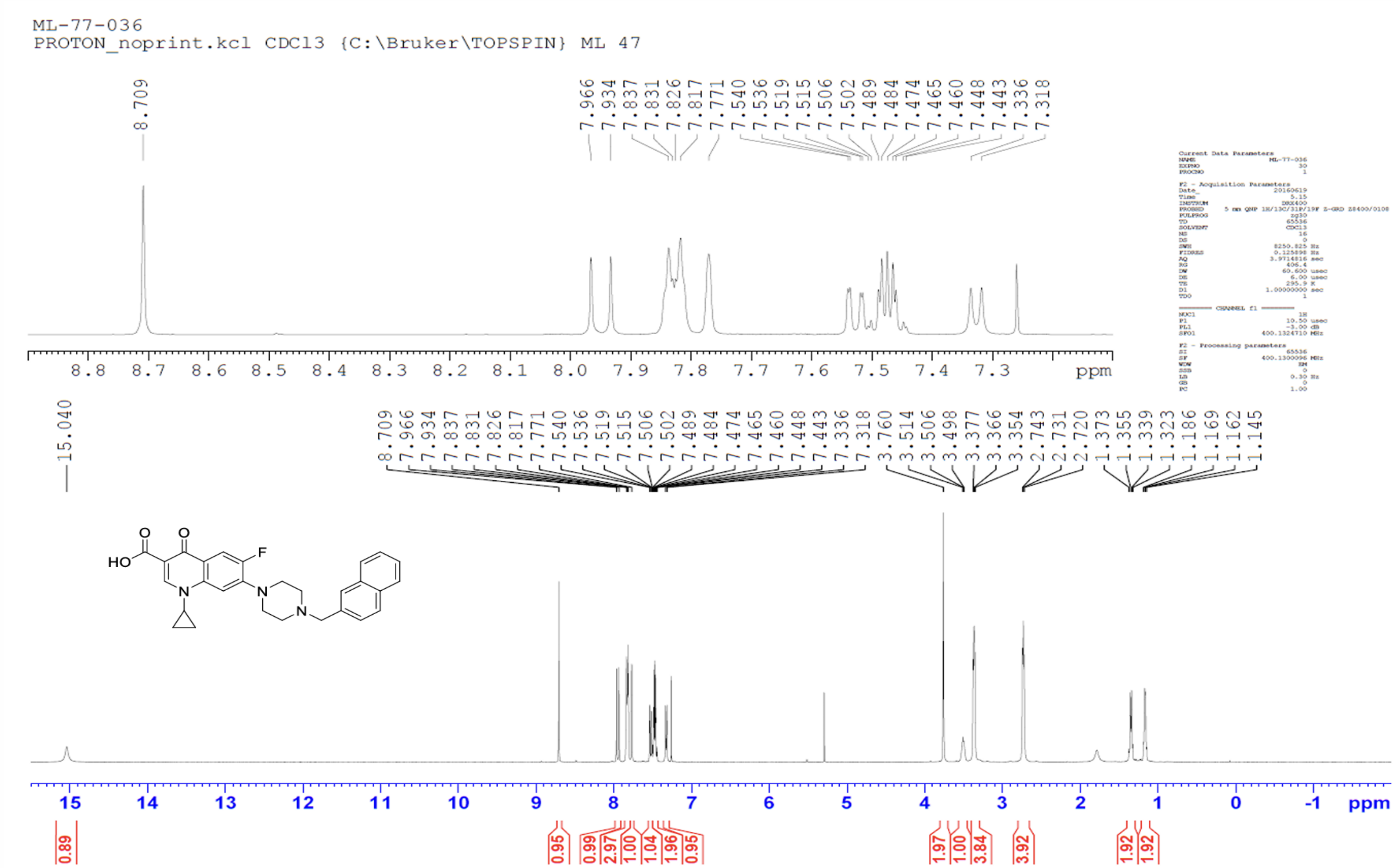

Compound 23 Salt– ^{13}C spectrum

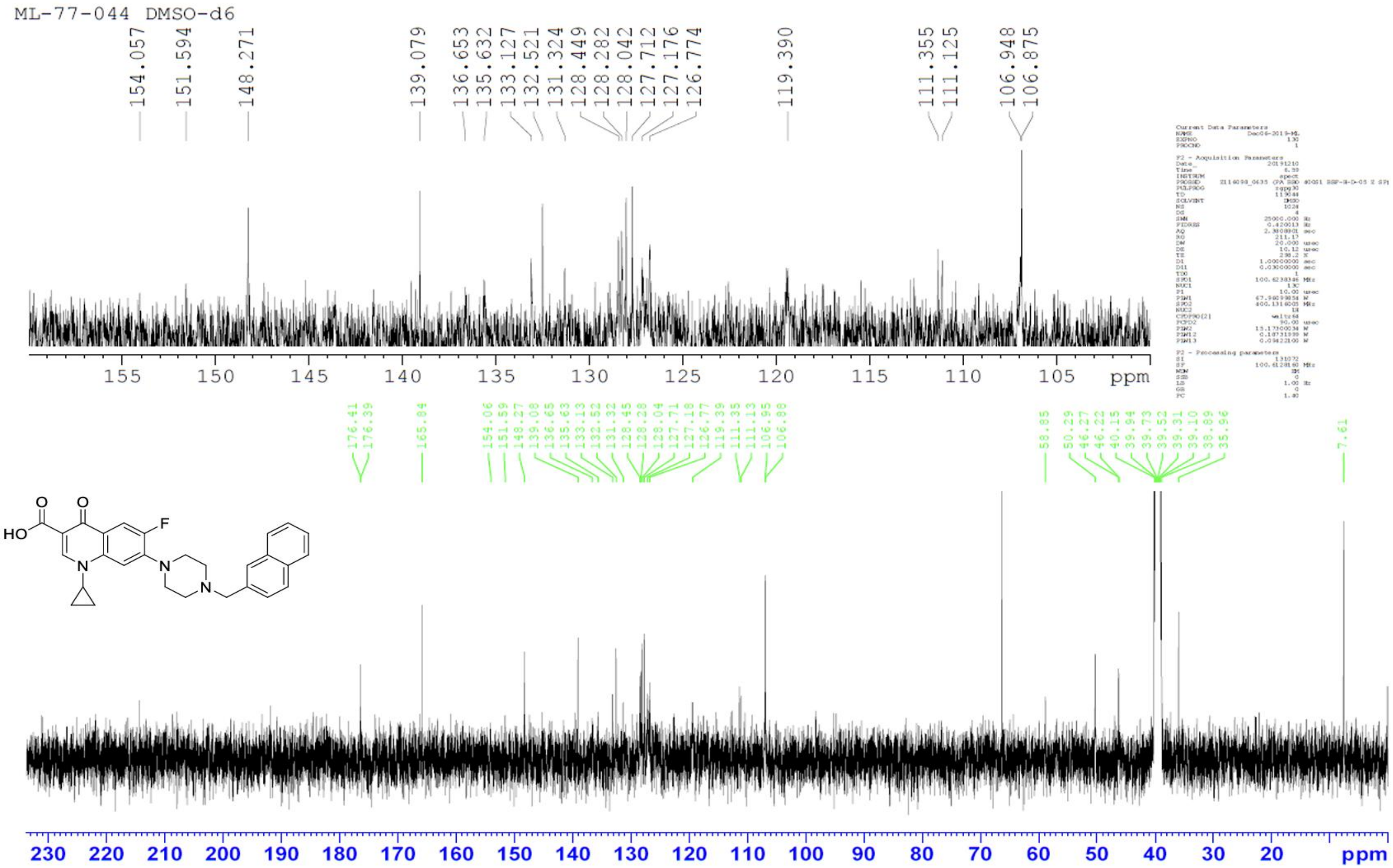

Compound 24– ^{1}H spectrum

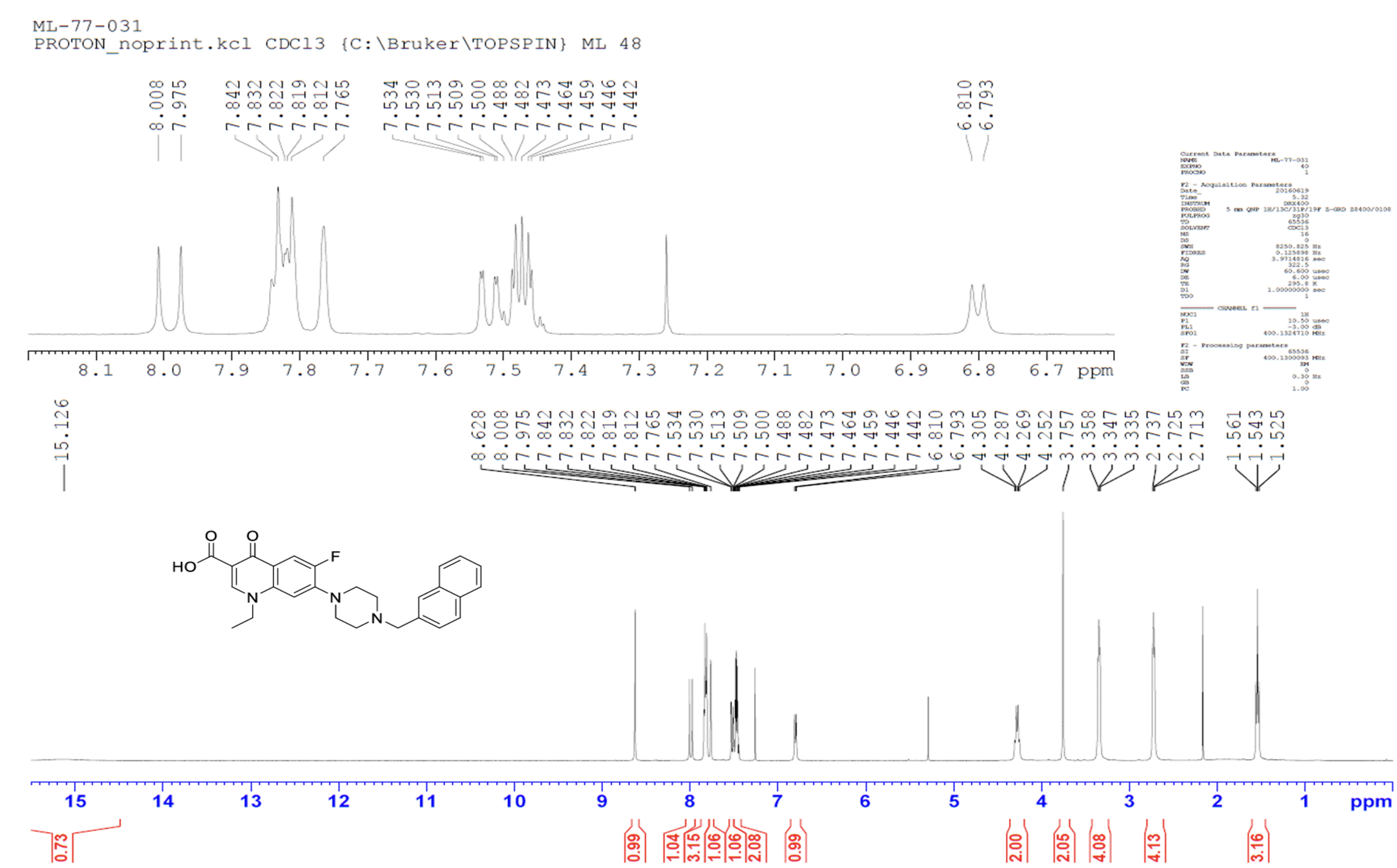

Compound 24 Salt– ^{13}C spectrum

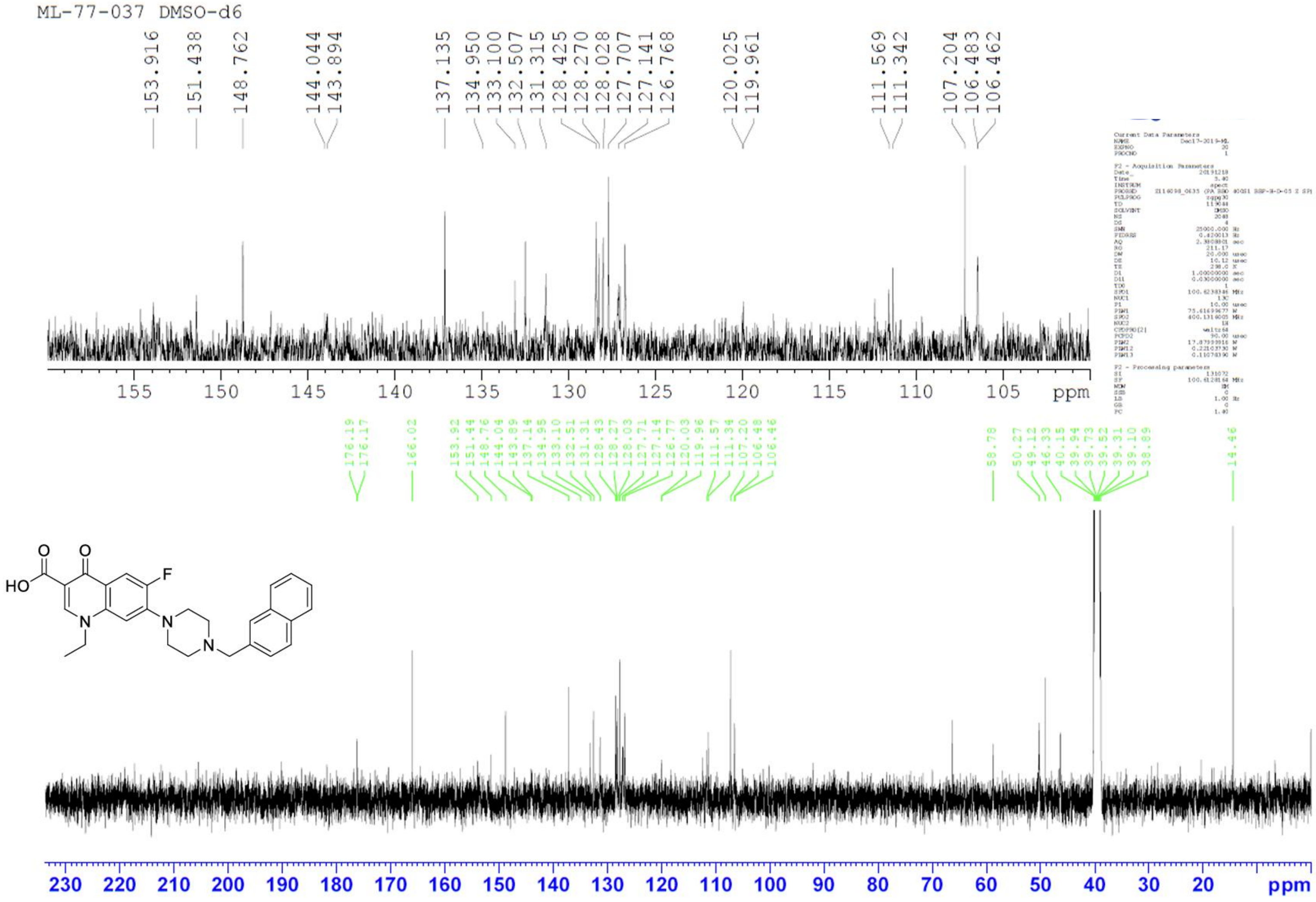

Compound 25– ^{1}H spectrum

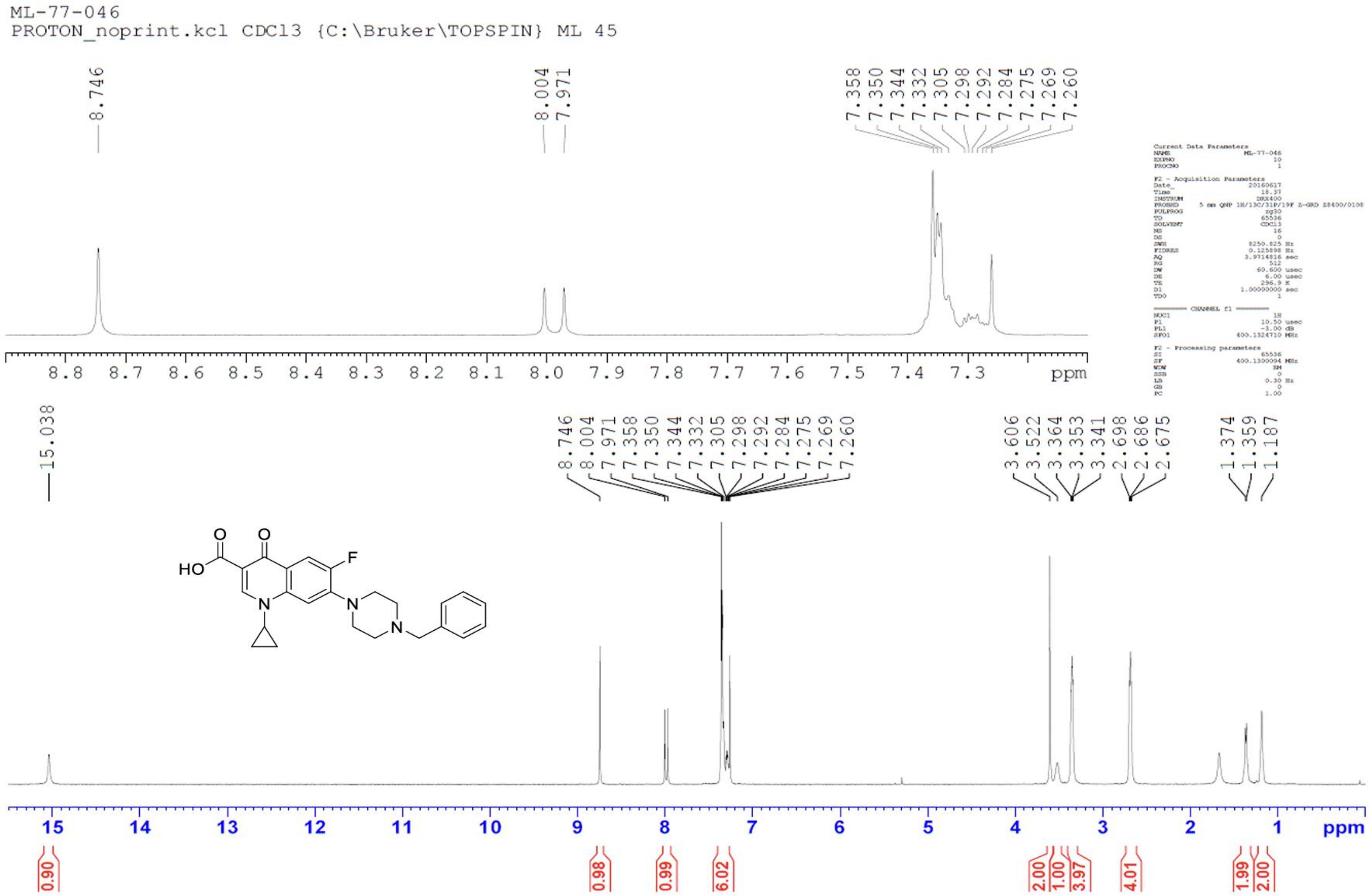

Compound 25 Salt – ^{13}C spectrum

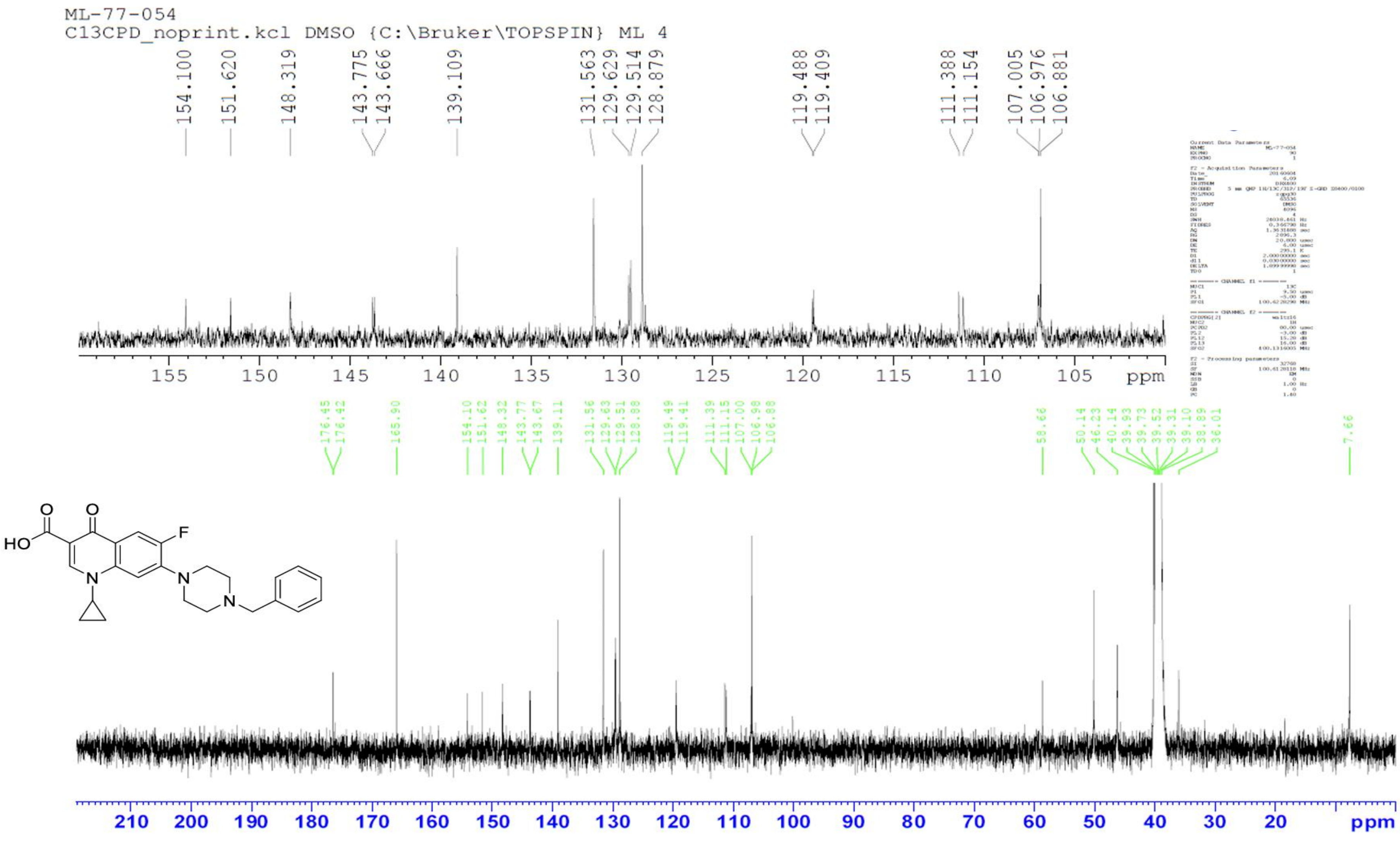

Compound 26– ^{1}H spectrum

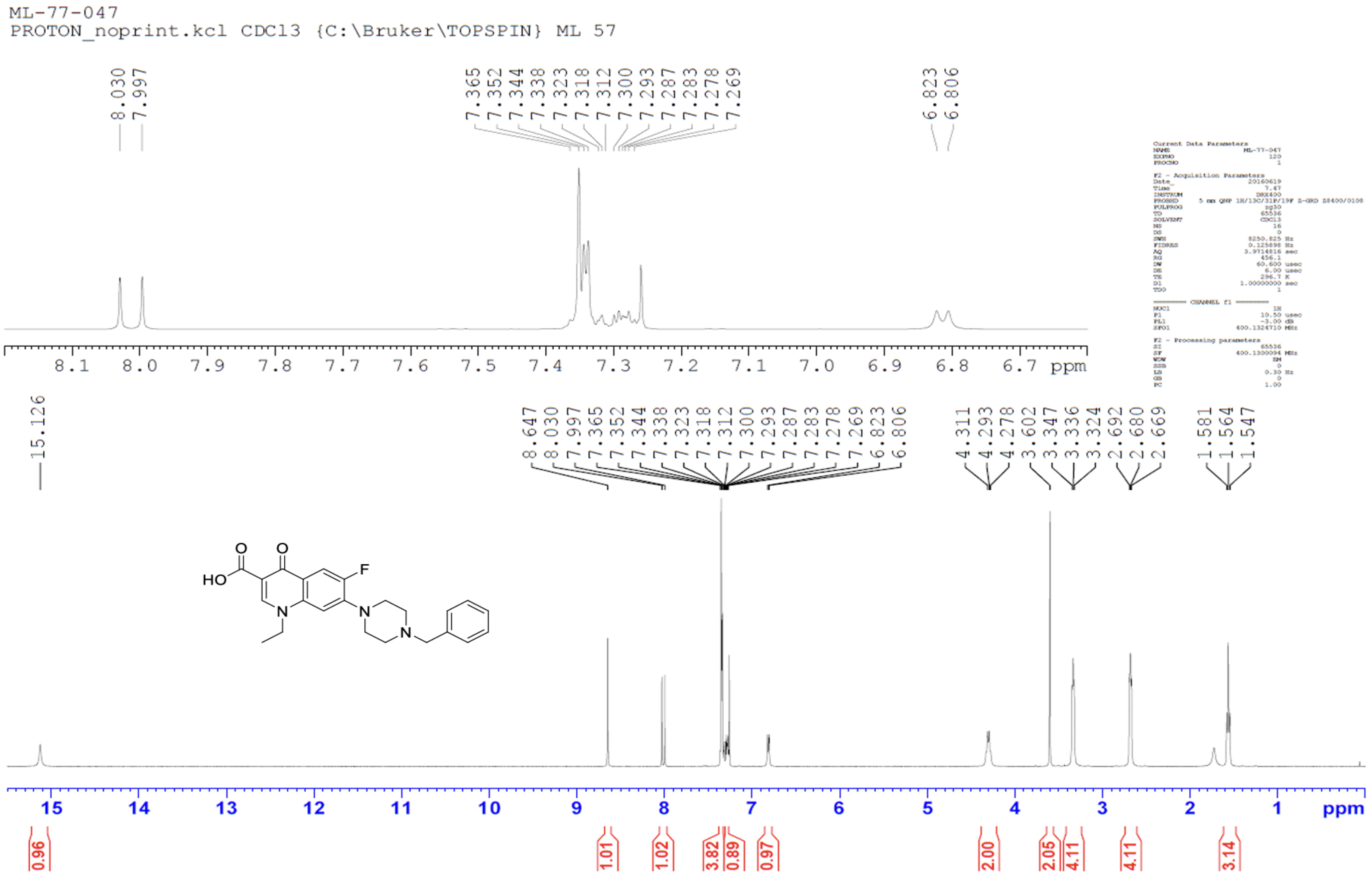

Compound 26 Salt– ^{13}C spectrum

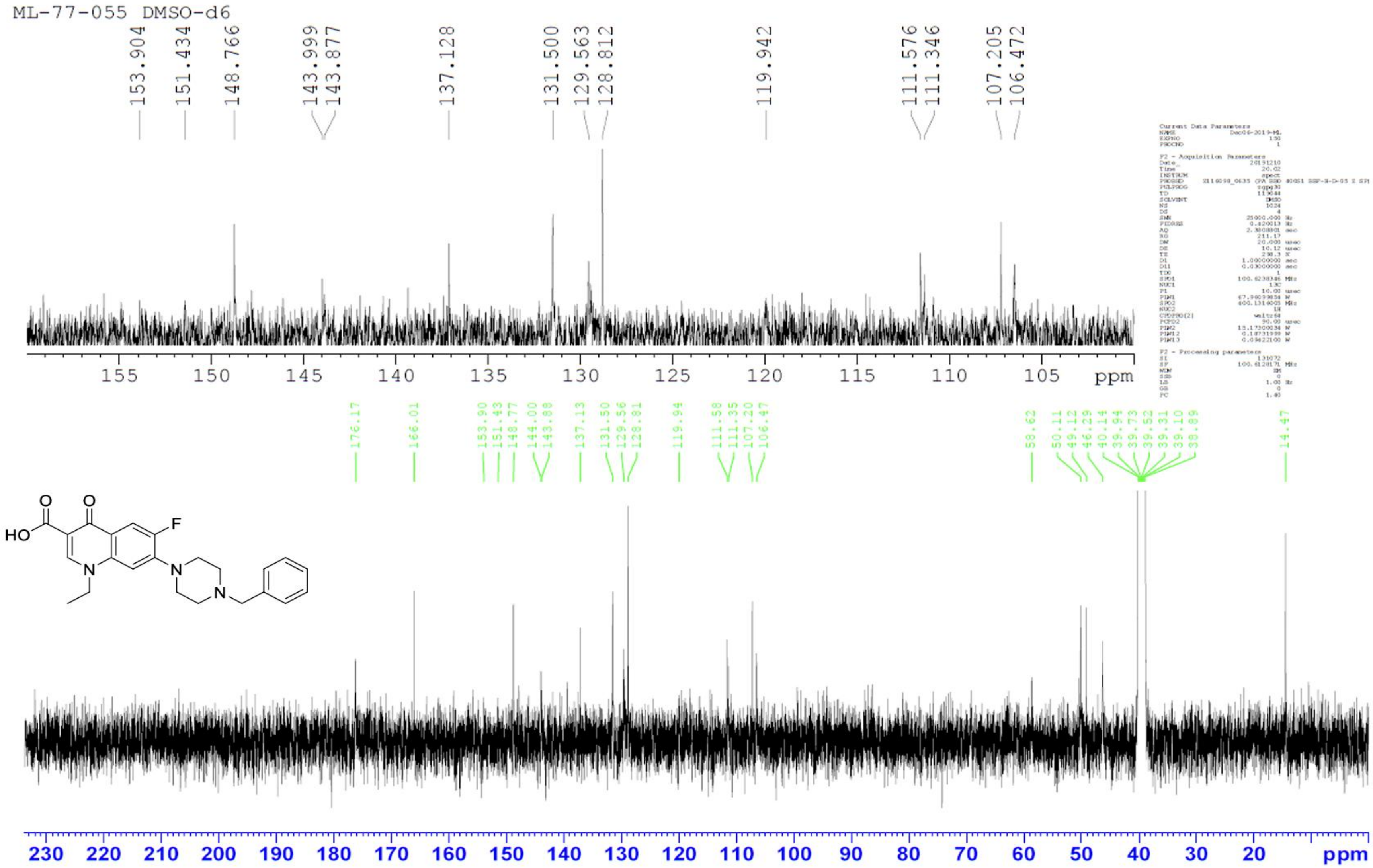

Compound 27 Salt – ^{1}H spectrum

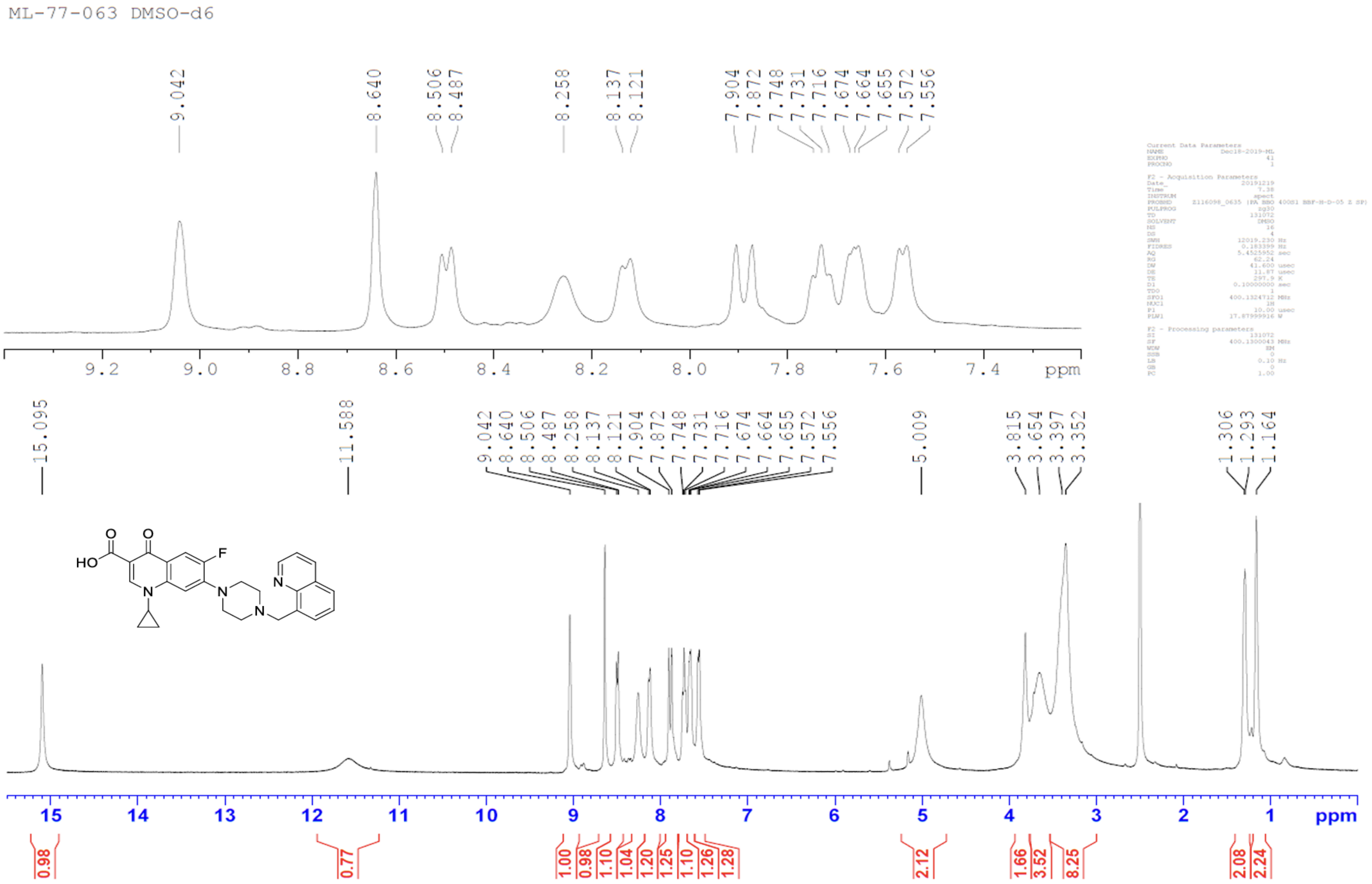

Compound 27 Salt– ^{13}C spectrum

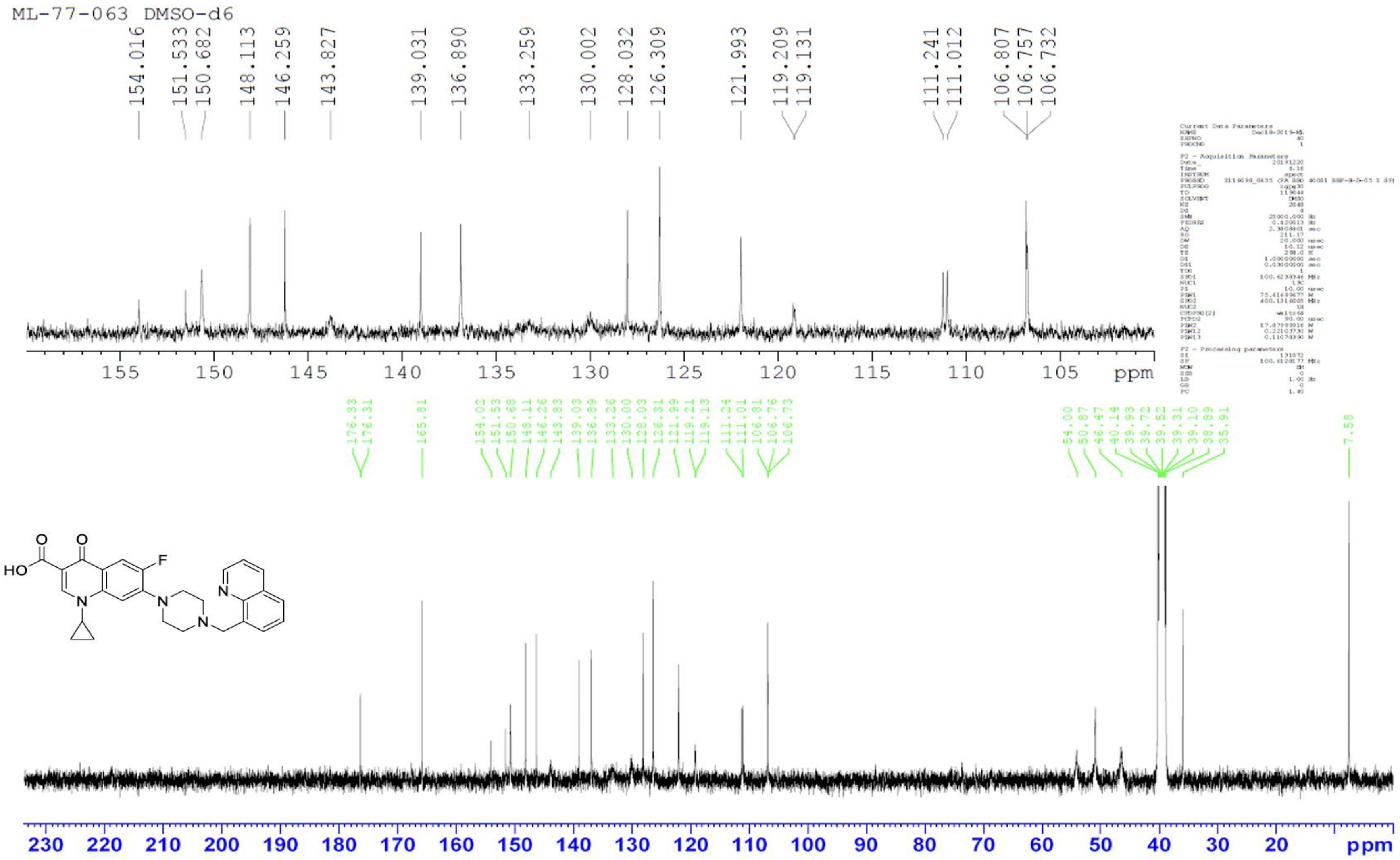

Compound 28– ^{1}H spectrum

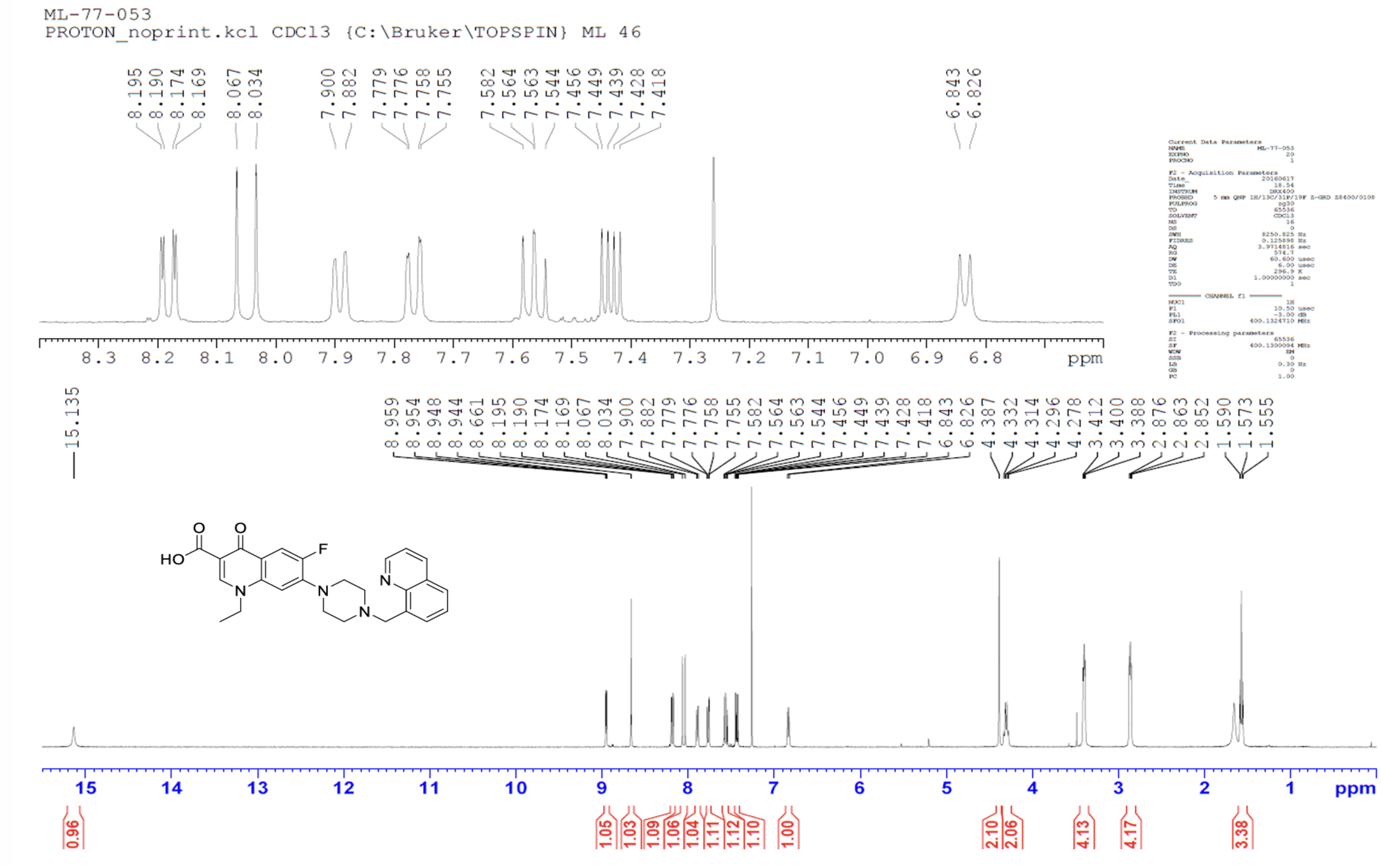

Compound 28 Salt – ^{13}C spectrum

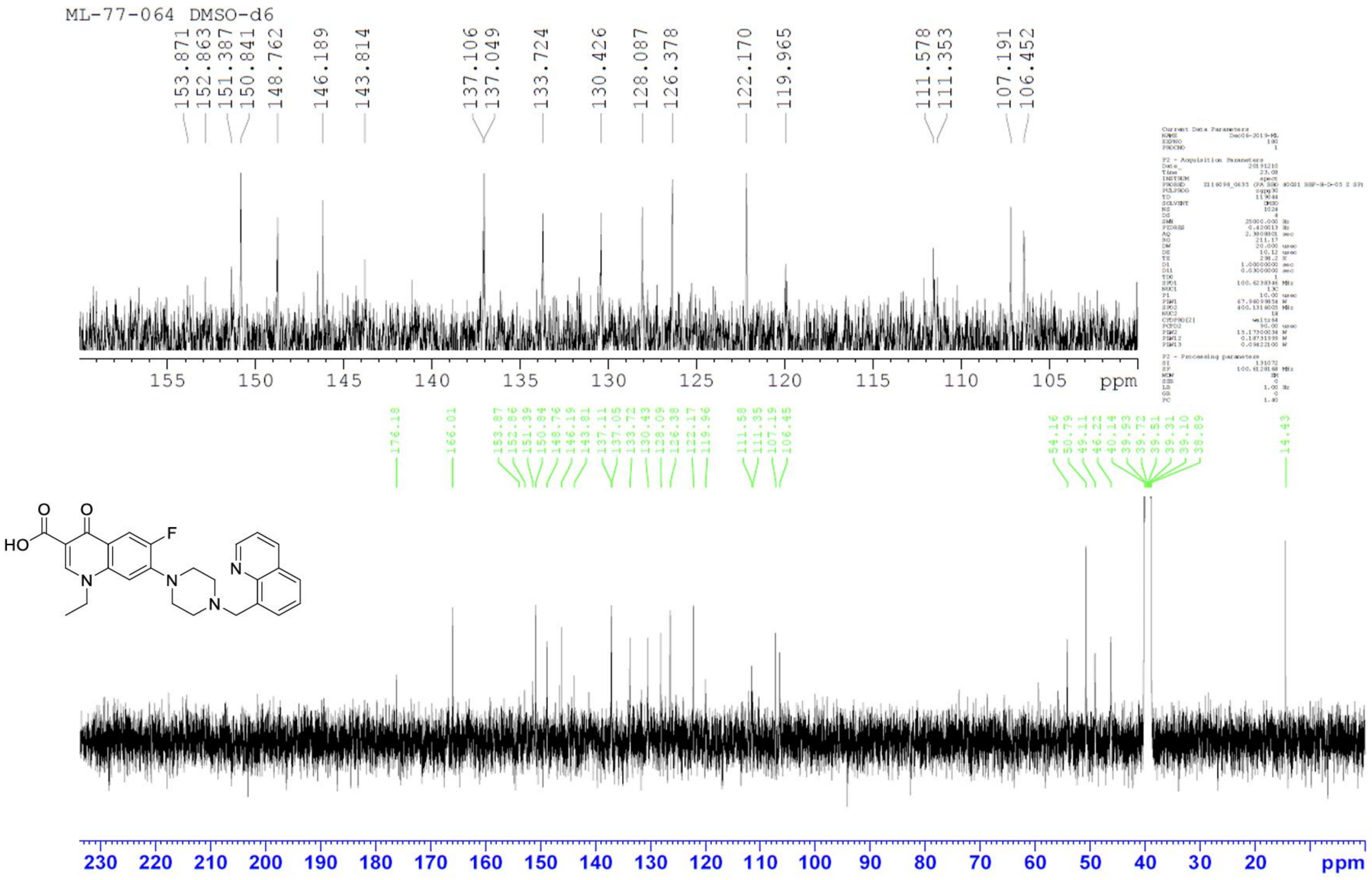

Compound 29– ^{1}H spectrum

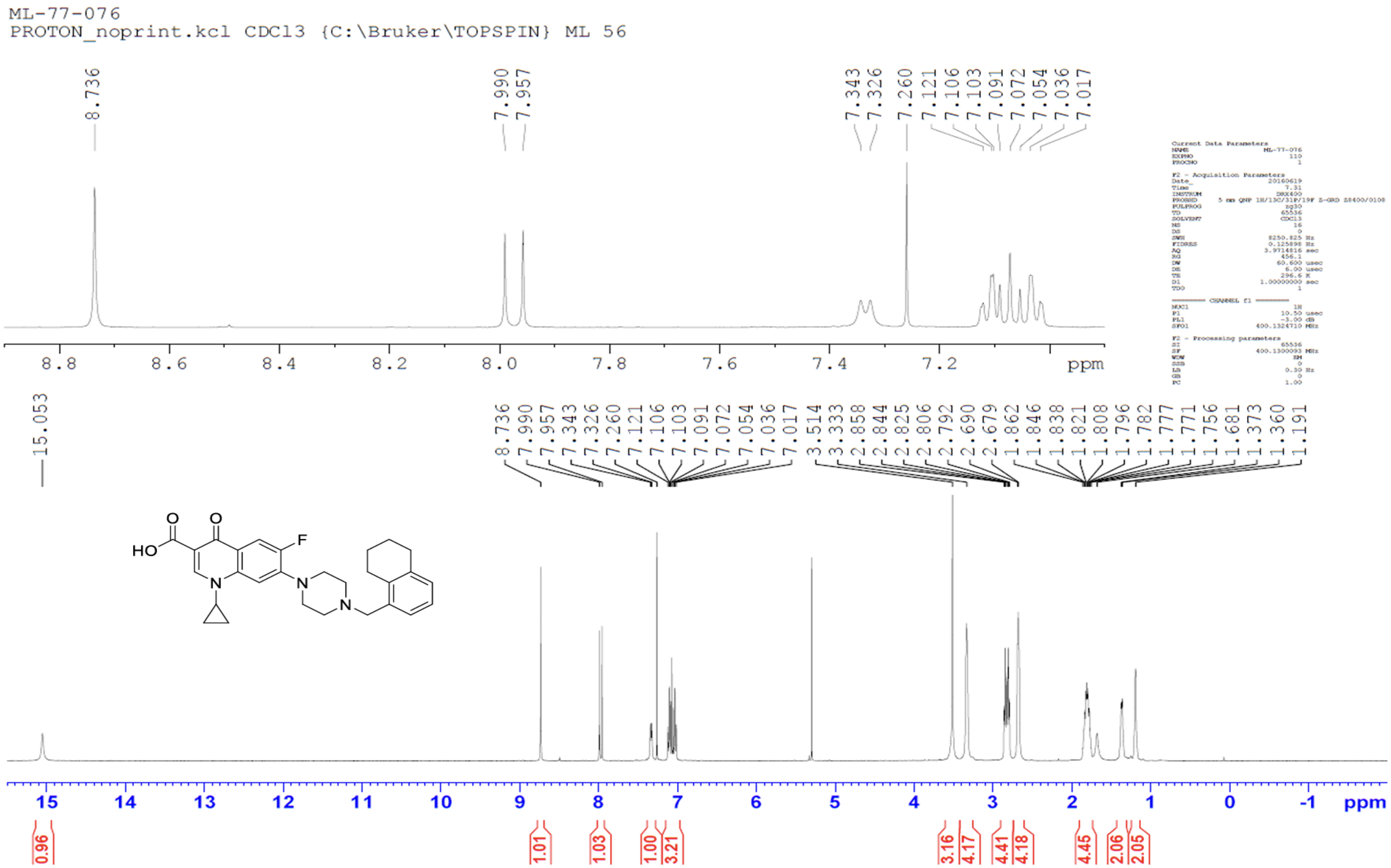

Compound 29– ^{13}C spectrum

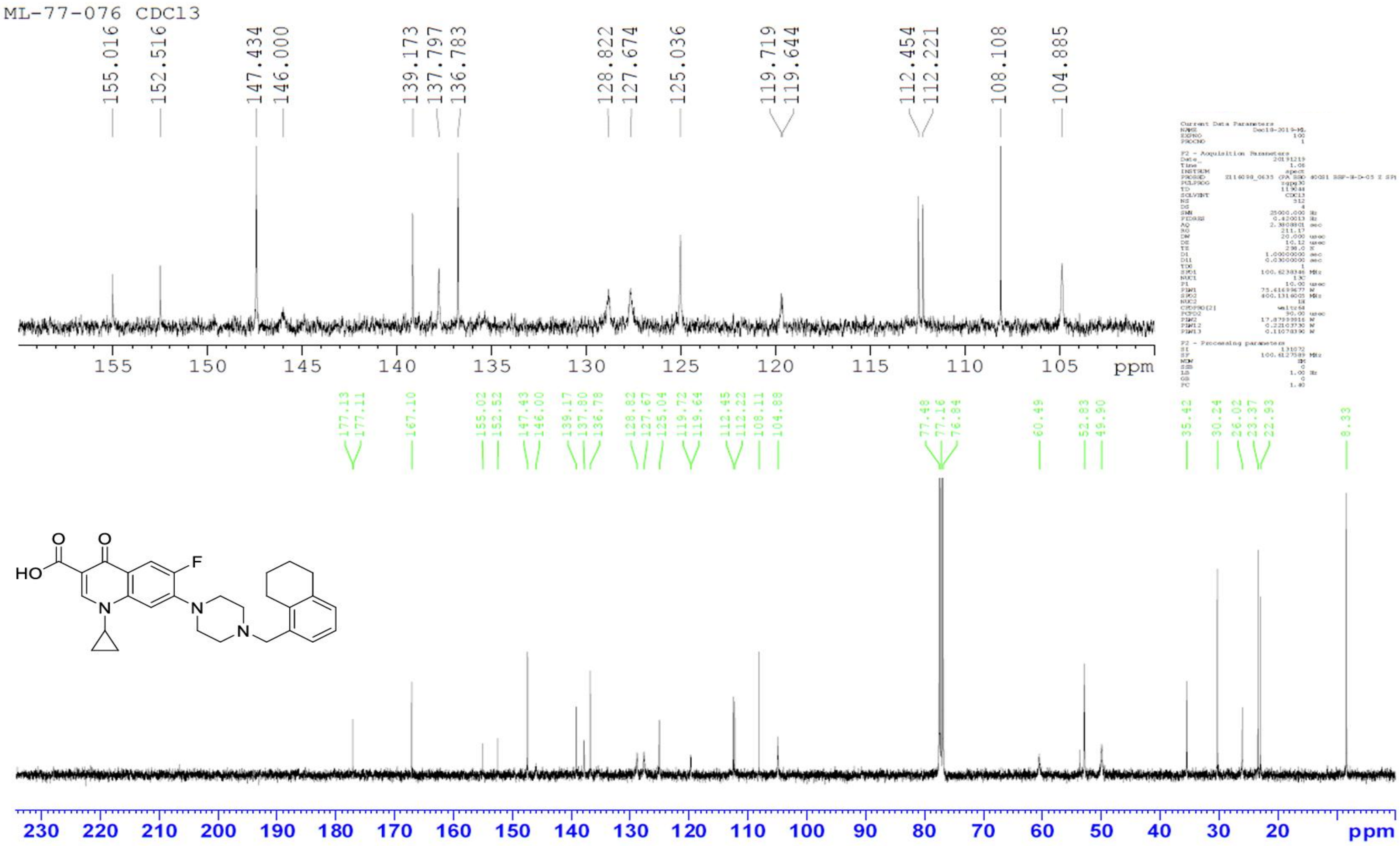

Compound 30– ^{1}H spectrum

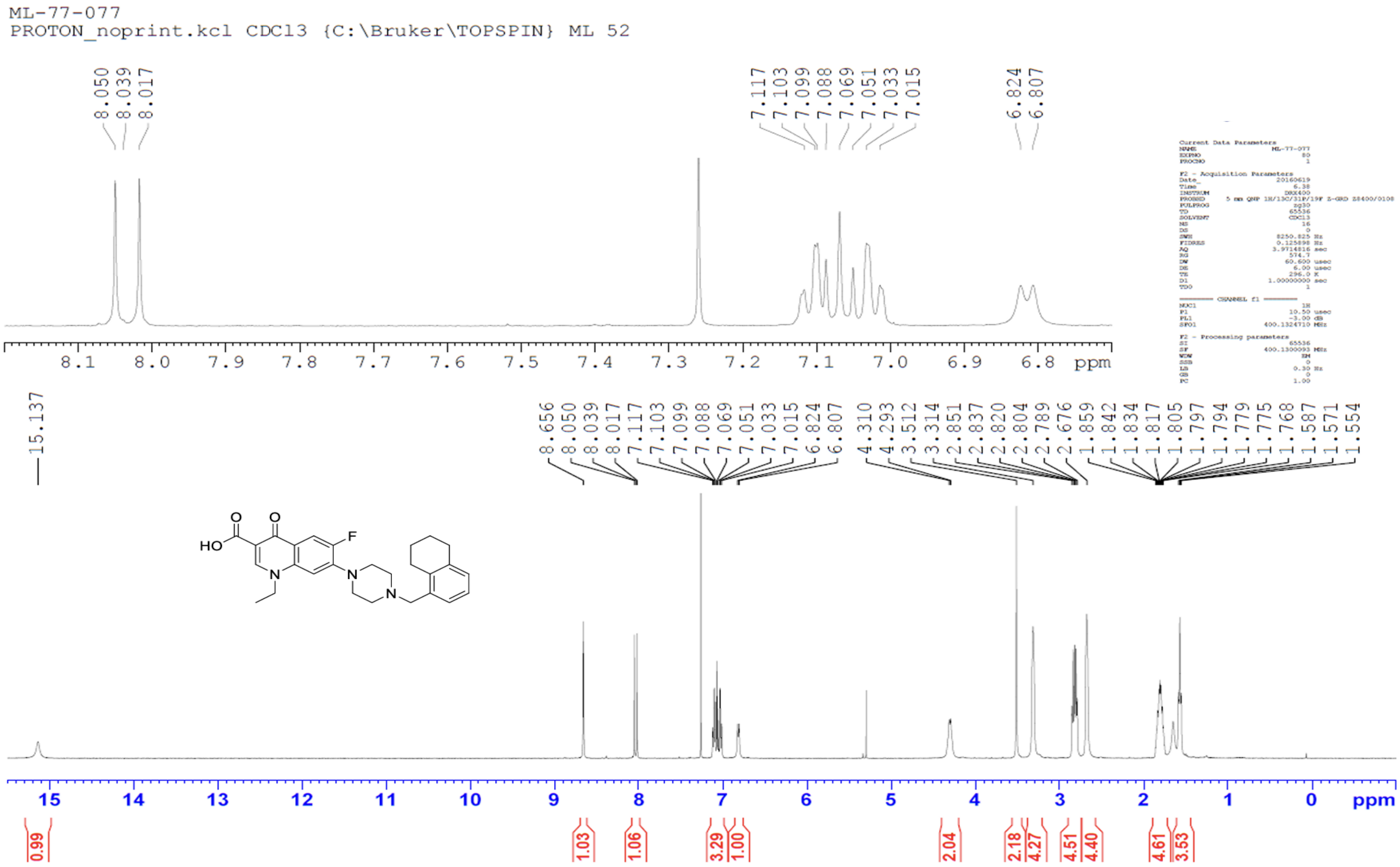

Compound 30– ^{13}C spectrum

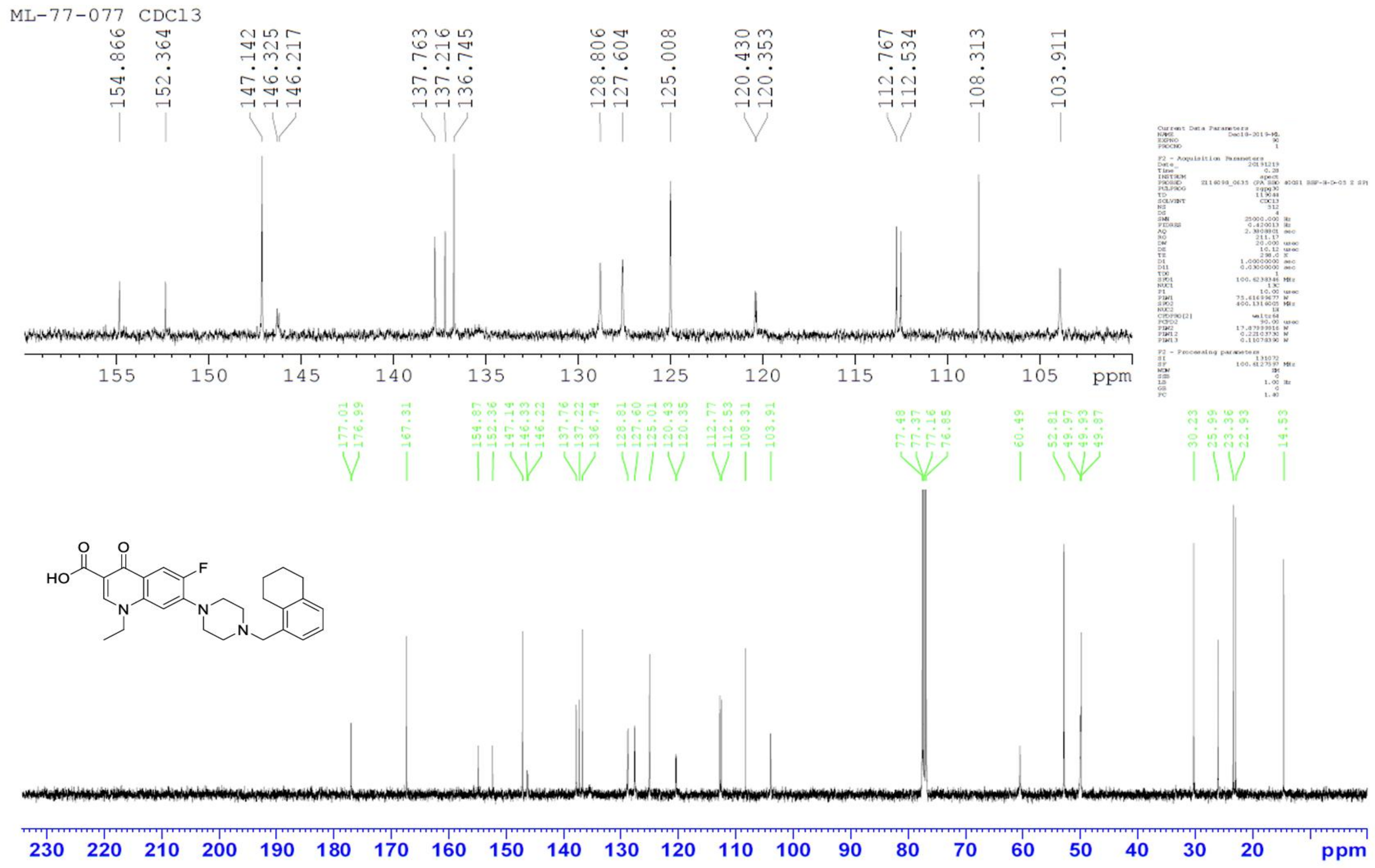

Compound 31– ^{1}H spectrum

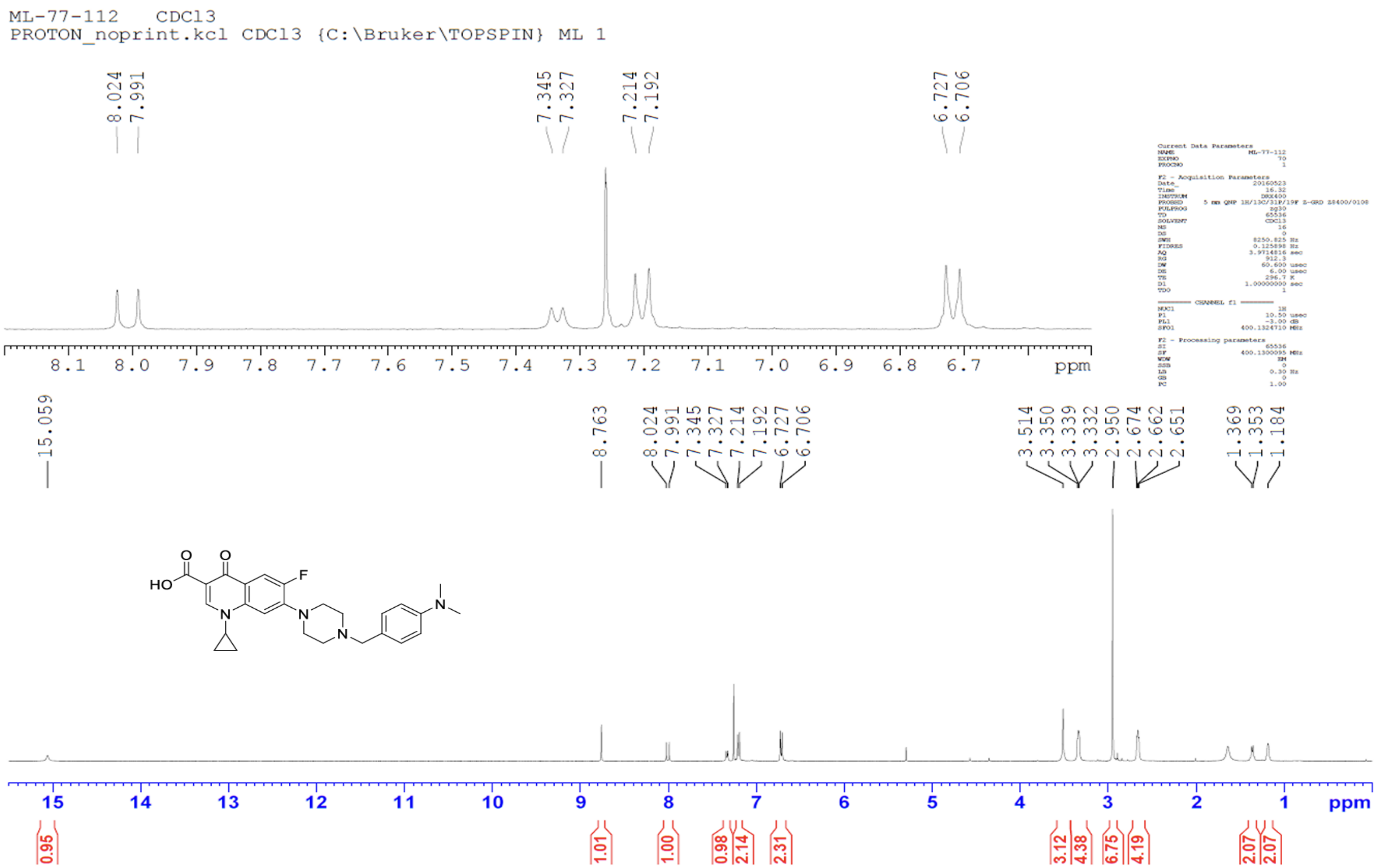

Compound 31– ^{13}C spectrum

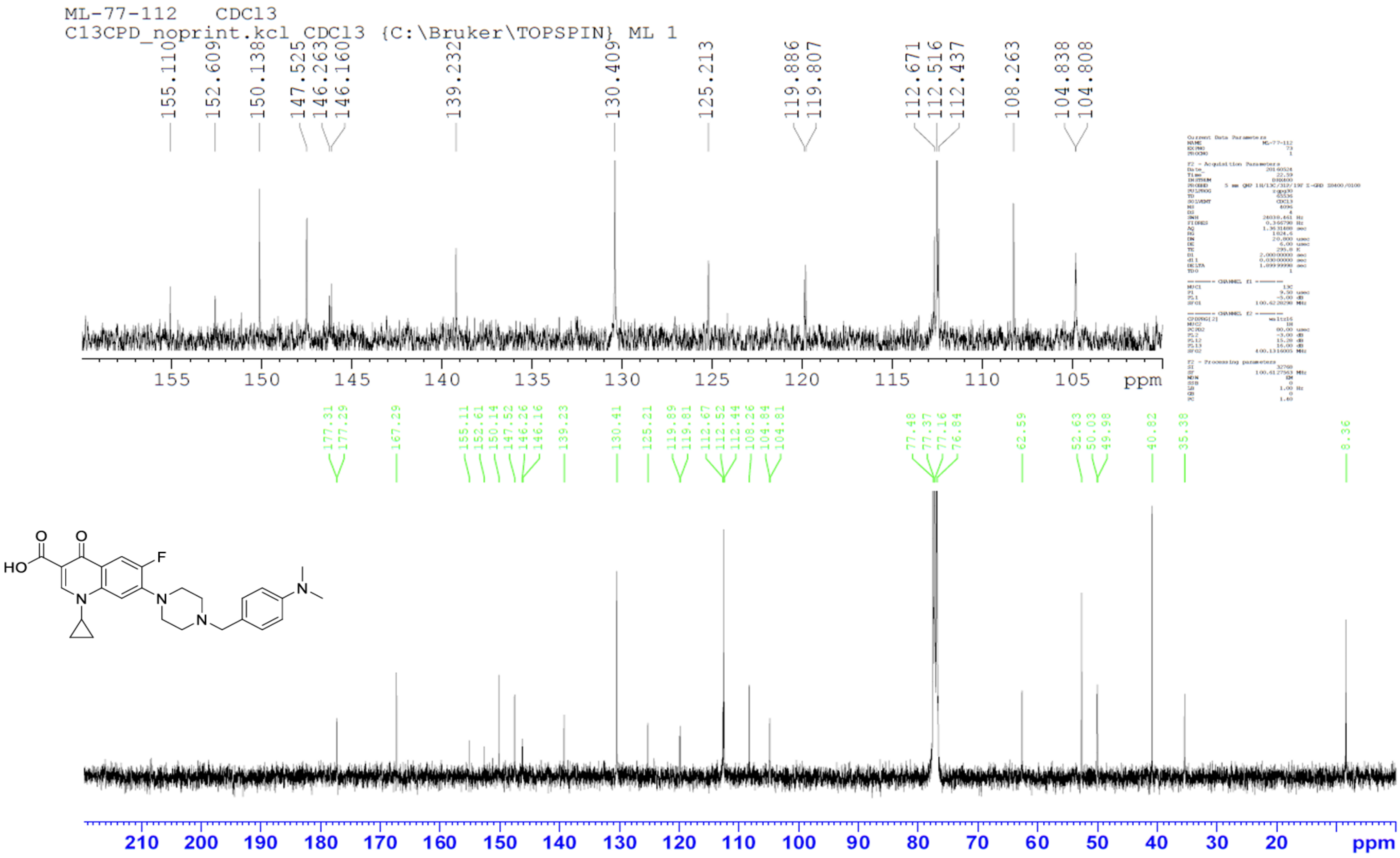

Compound 32– ^{1}H spectrum

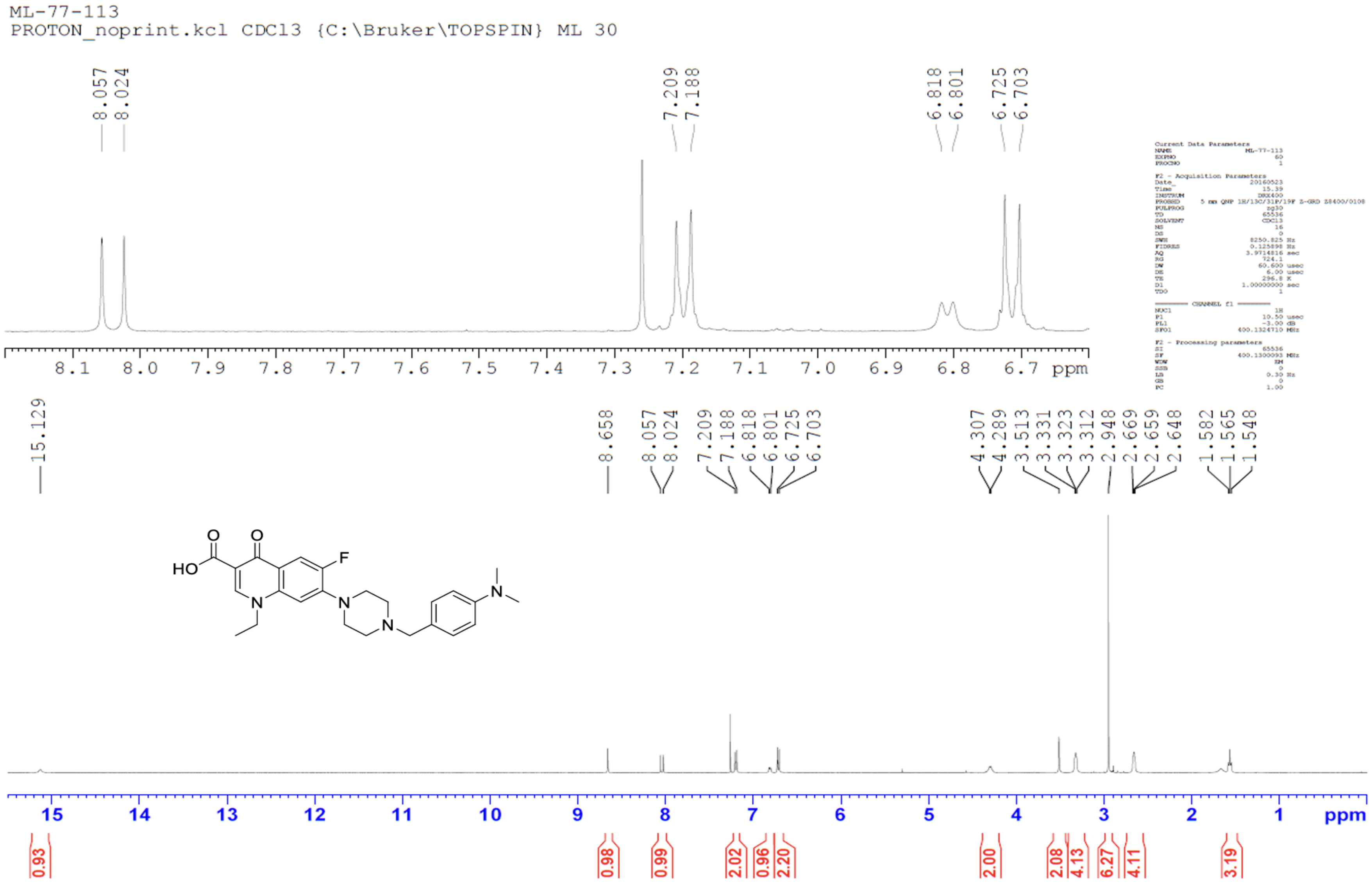

Compound 32– ^{13}C spectrum

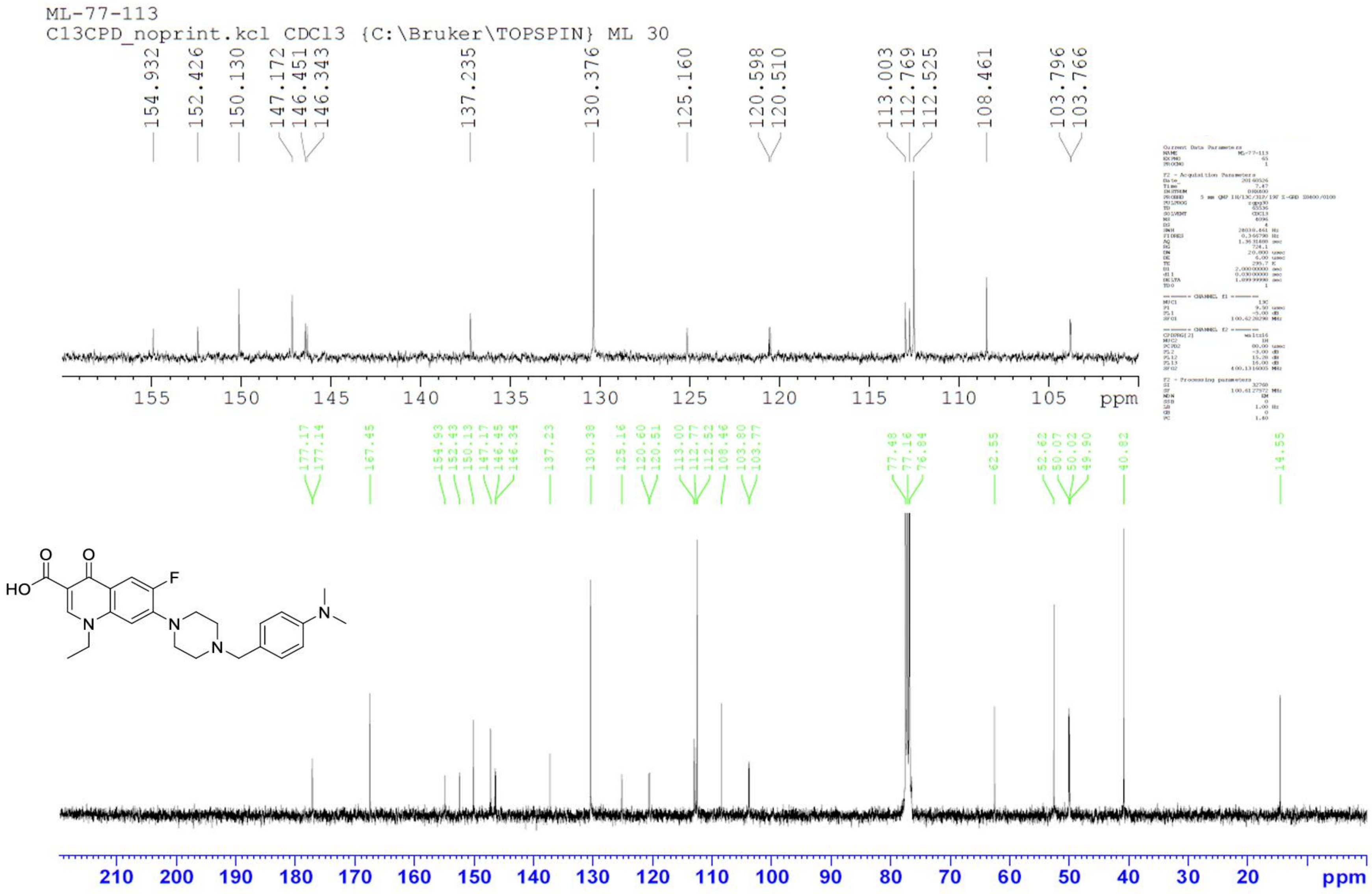

Compound 33– ^{1}H spectrum

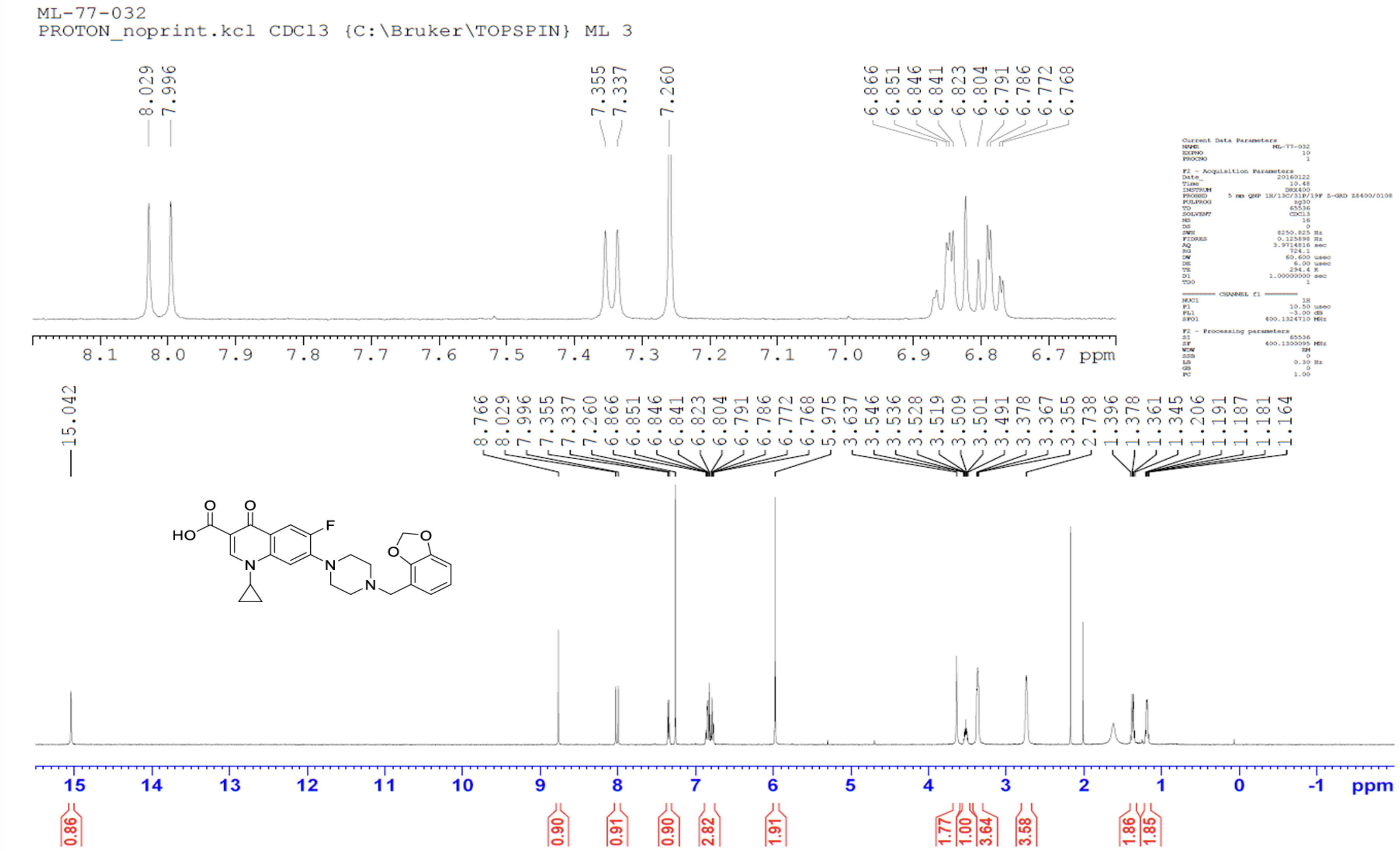

Compound 33– ^{13}C spectrum

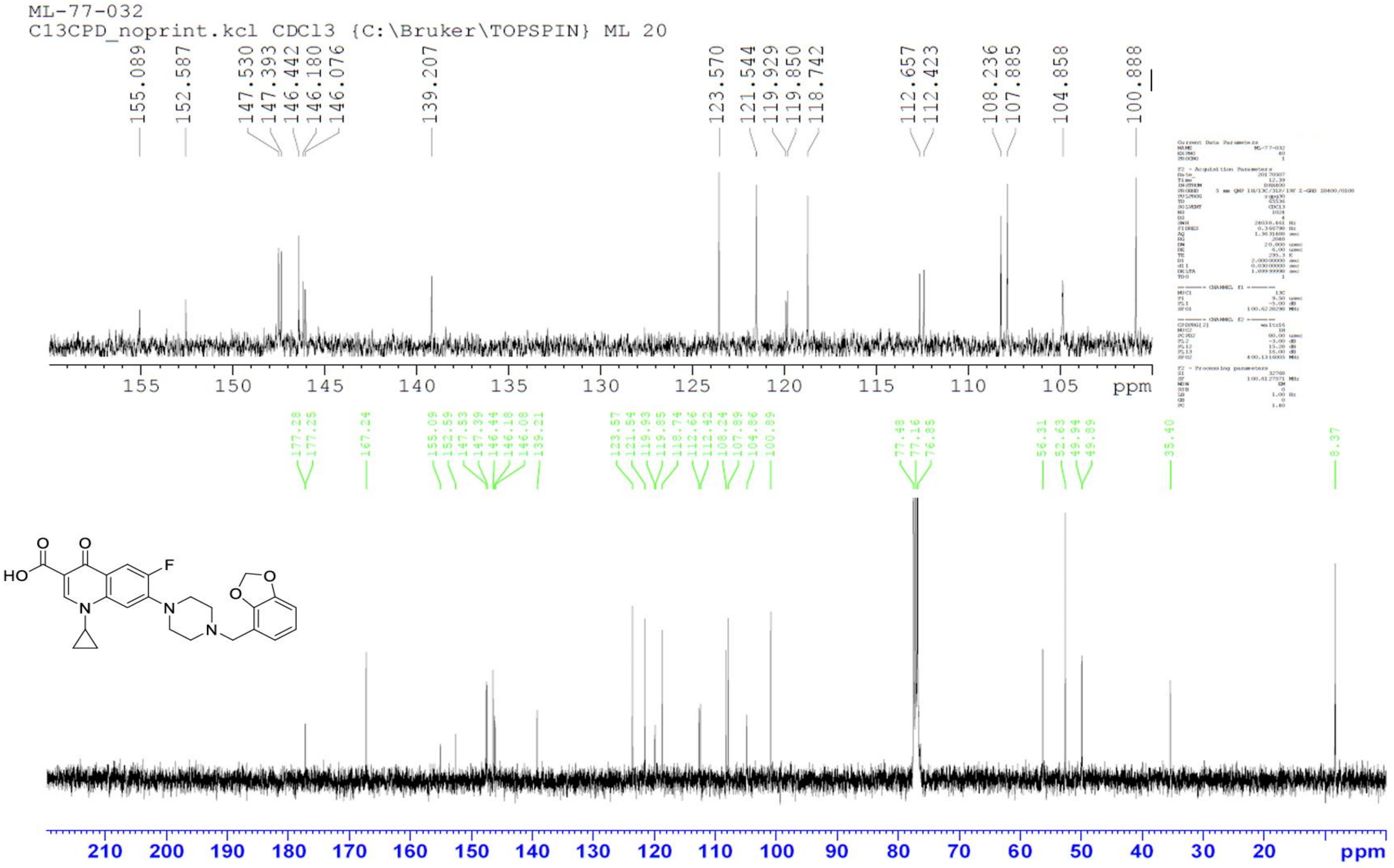

Compound 34– ^{1}H spectrum

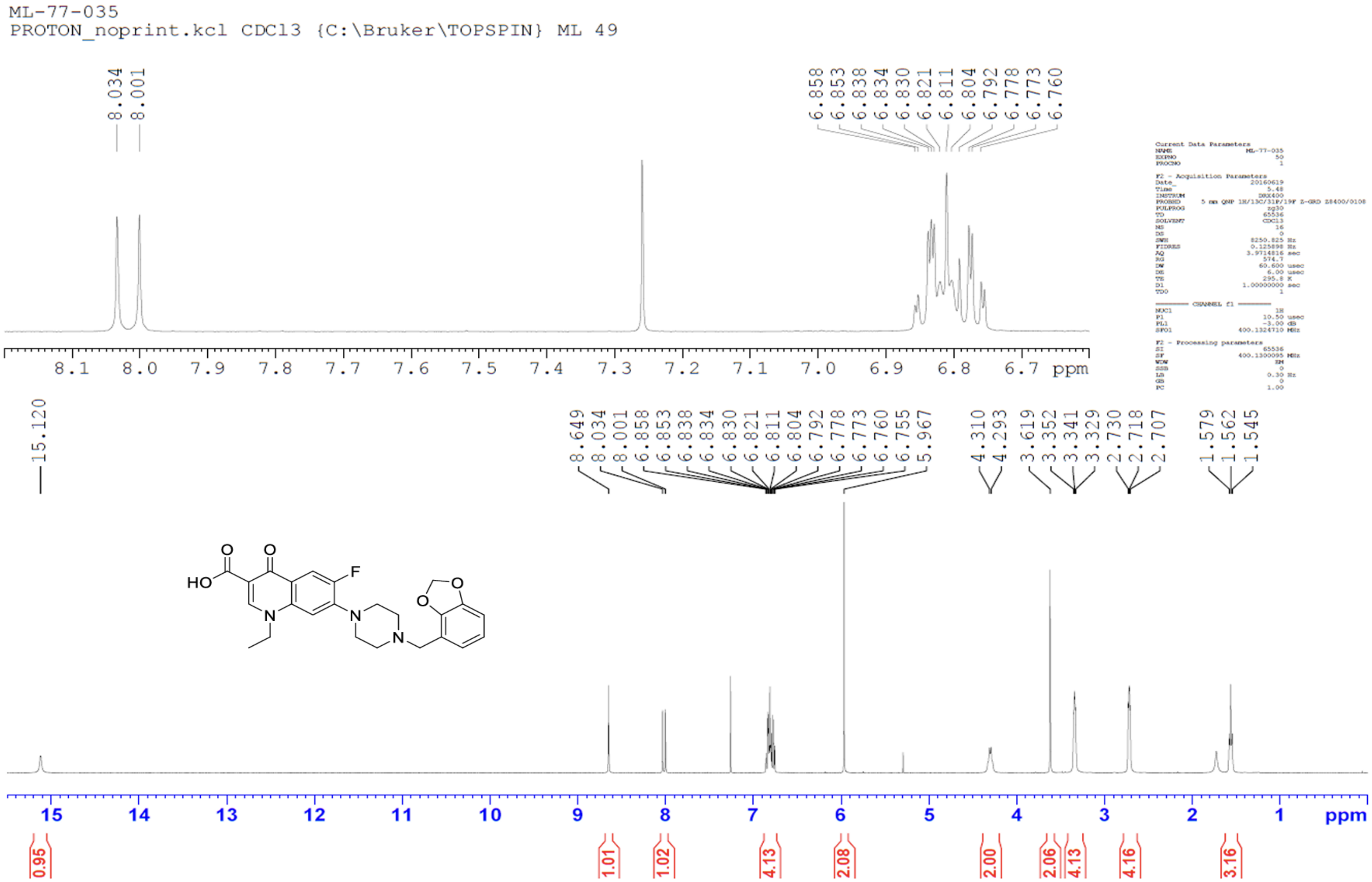

Compound 34 Salt– ^{13}C spectrum

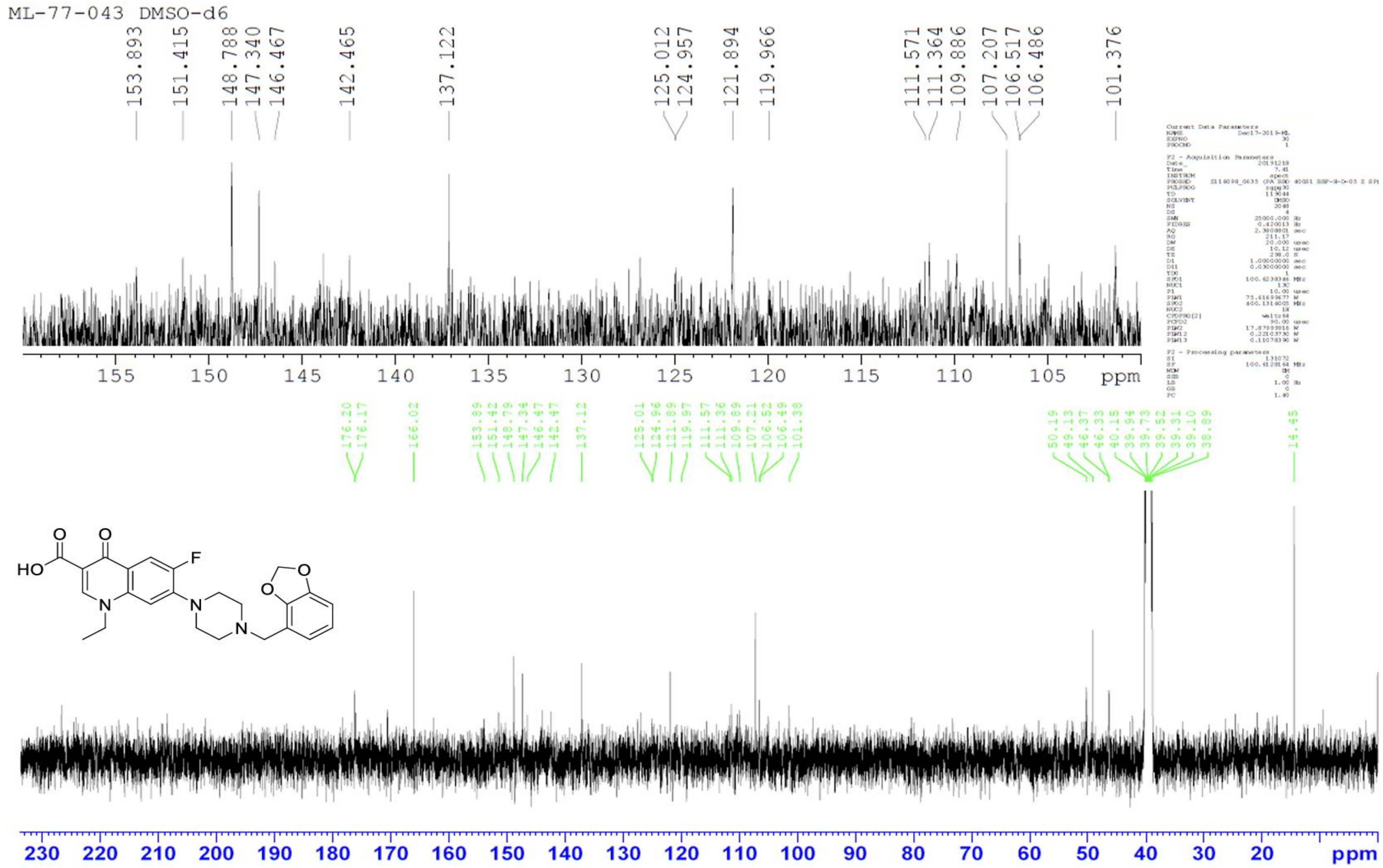

Compound 35– ^{1}H spectrum

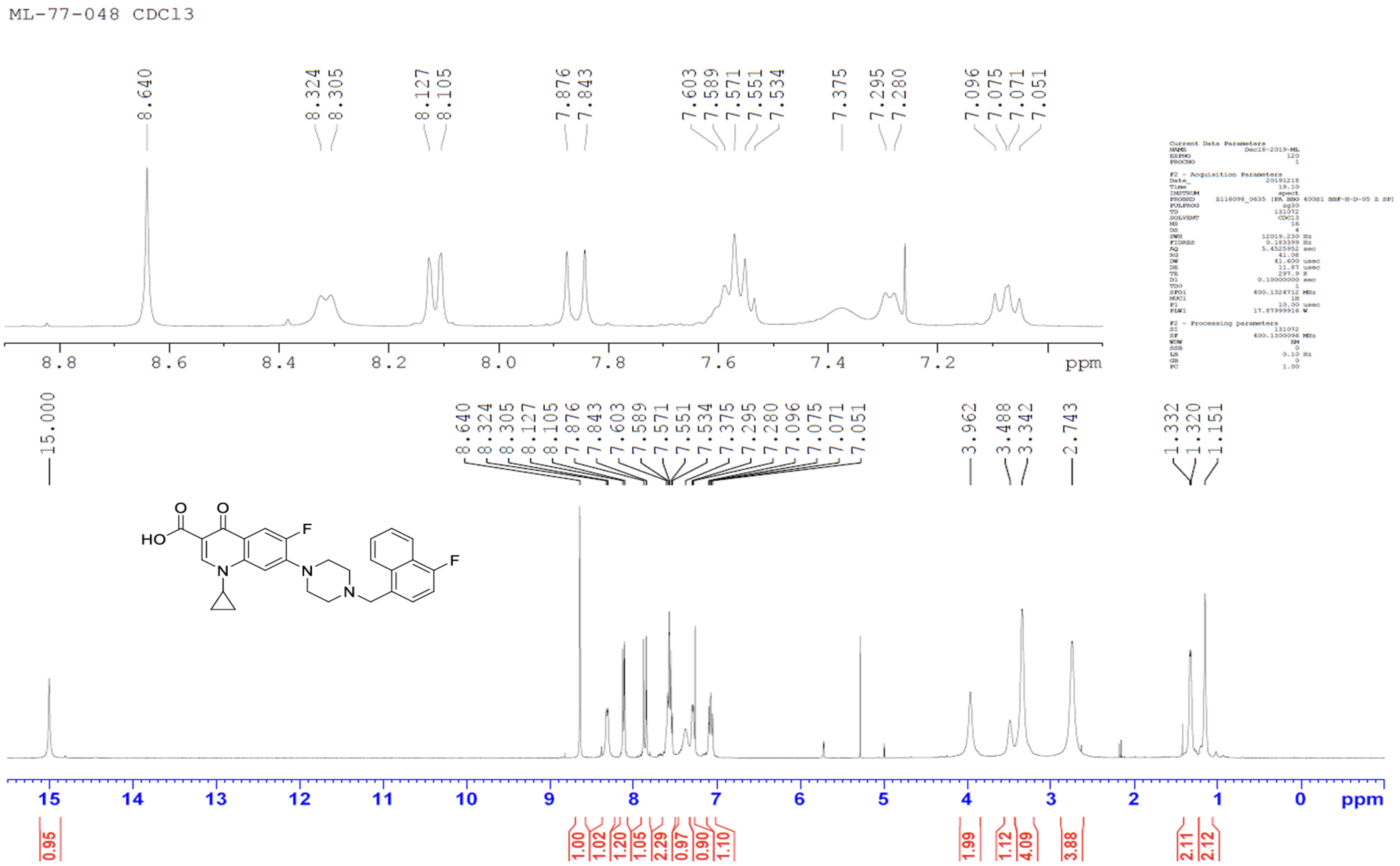

Compound 35– ^{13}C spectrum

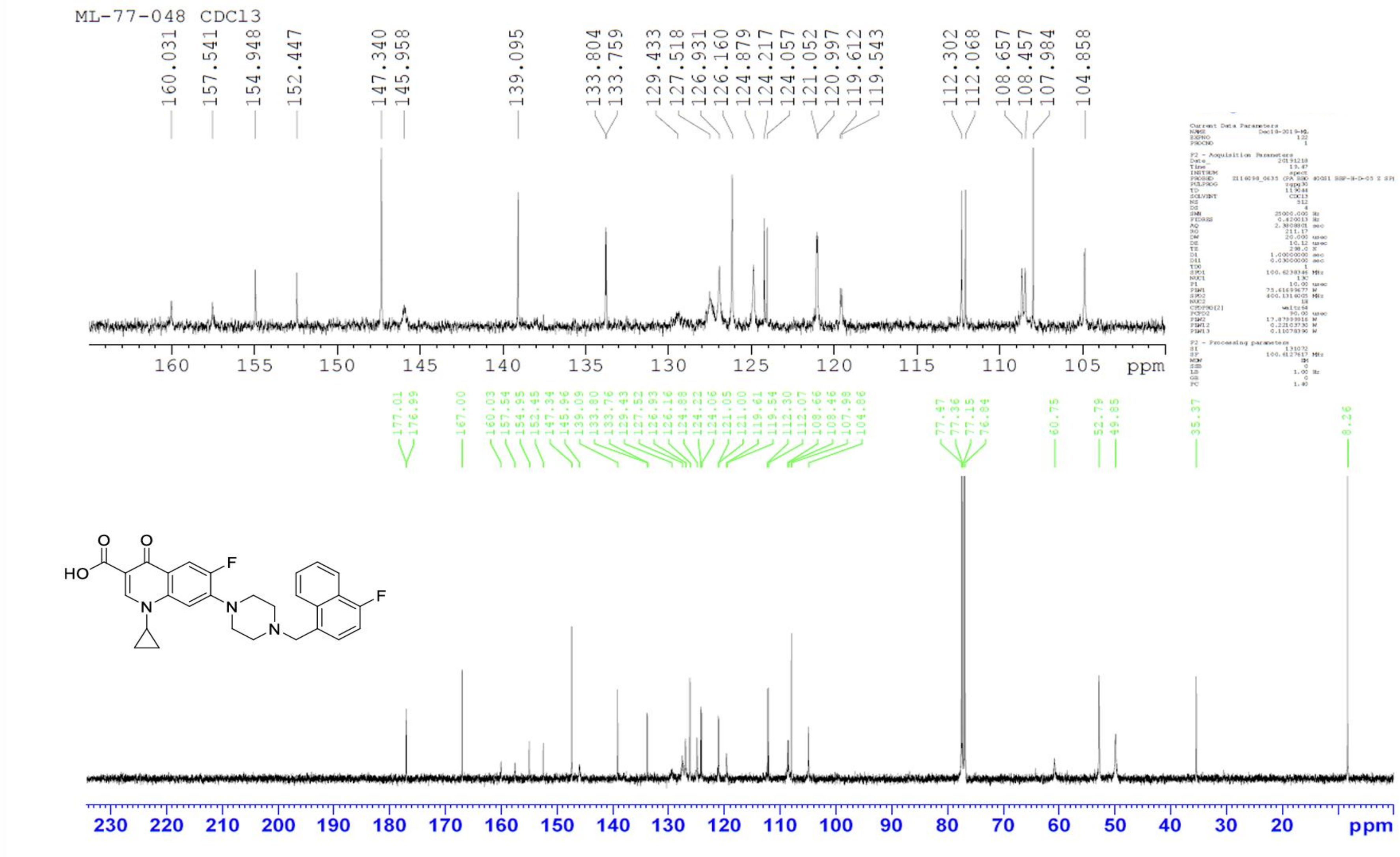

Compound 36– ^{1}H spectrum

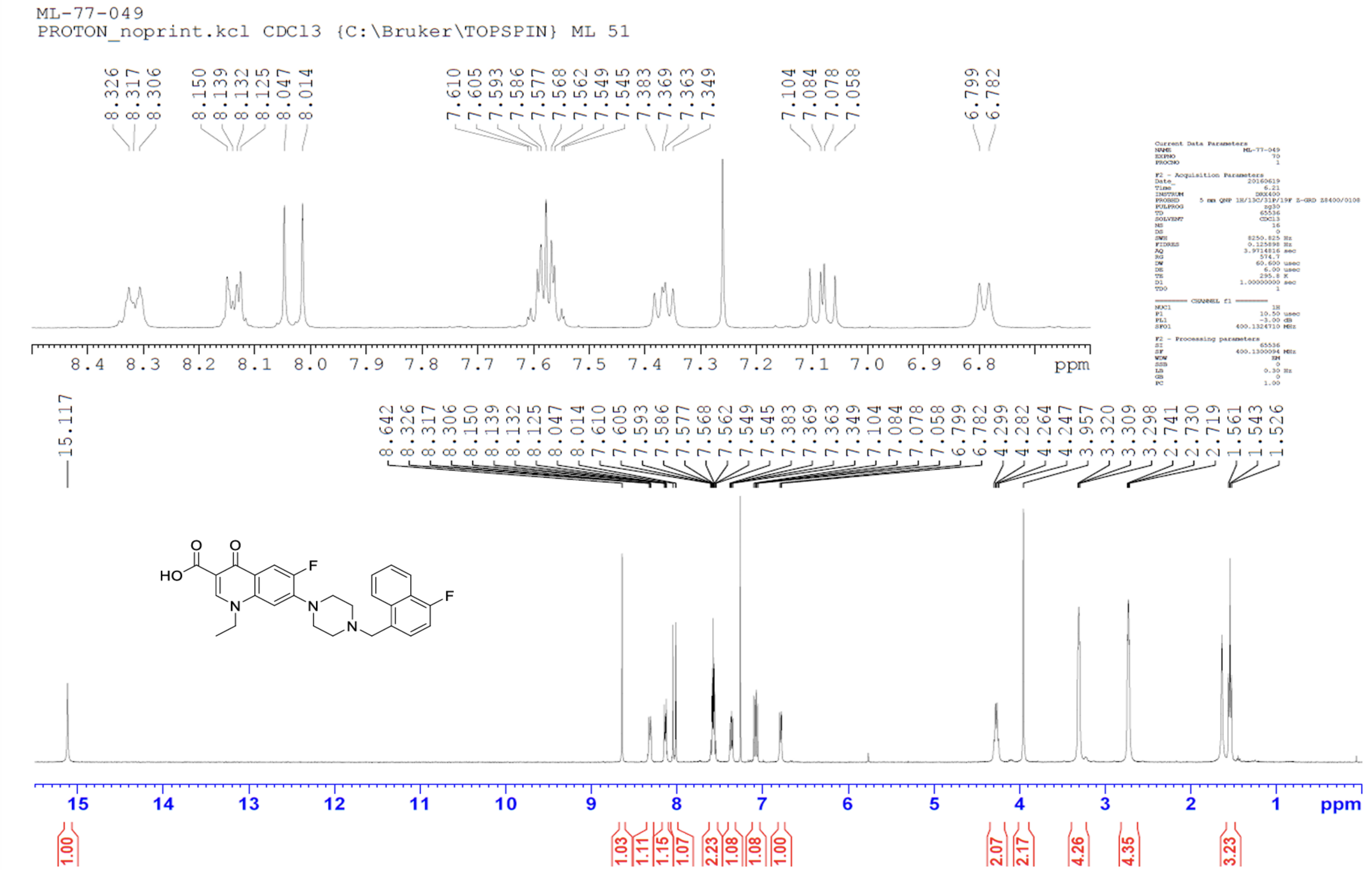

Compound 36 Salt – ^{13}C spectrum

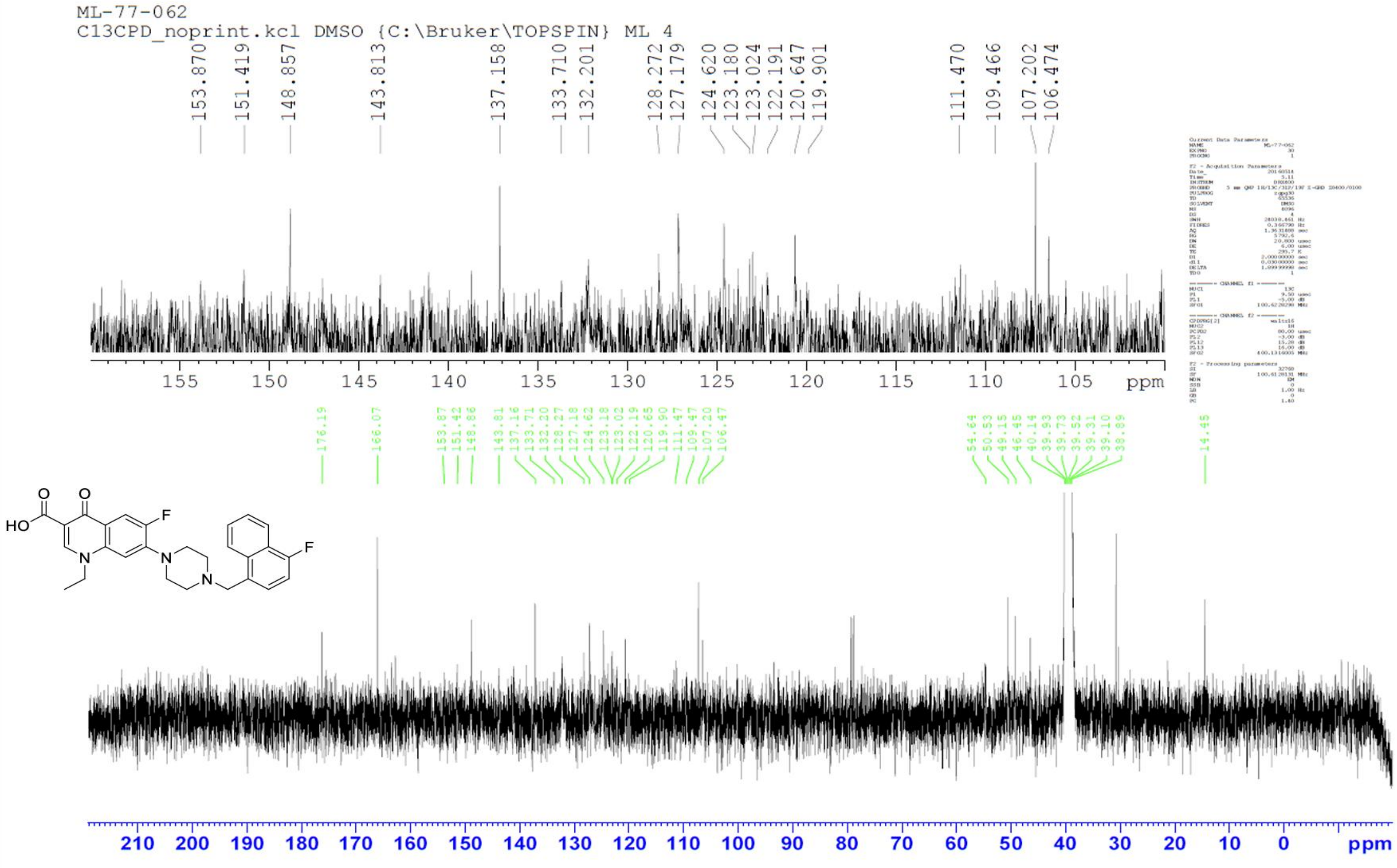

Compound 37 Salt – ^{1}H spectrum

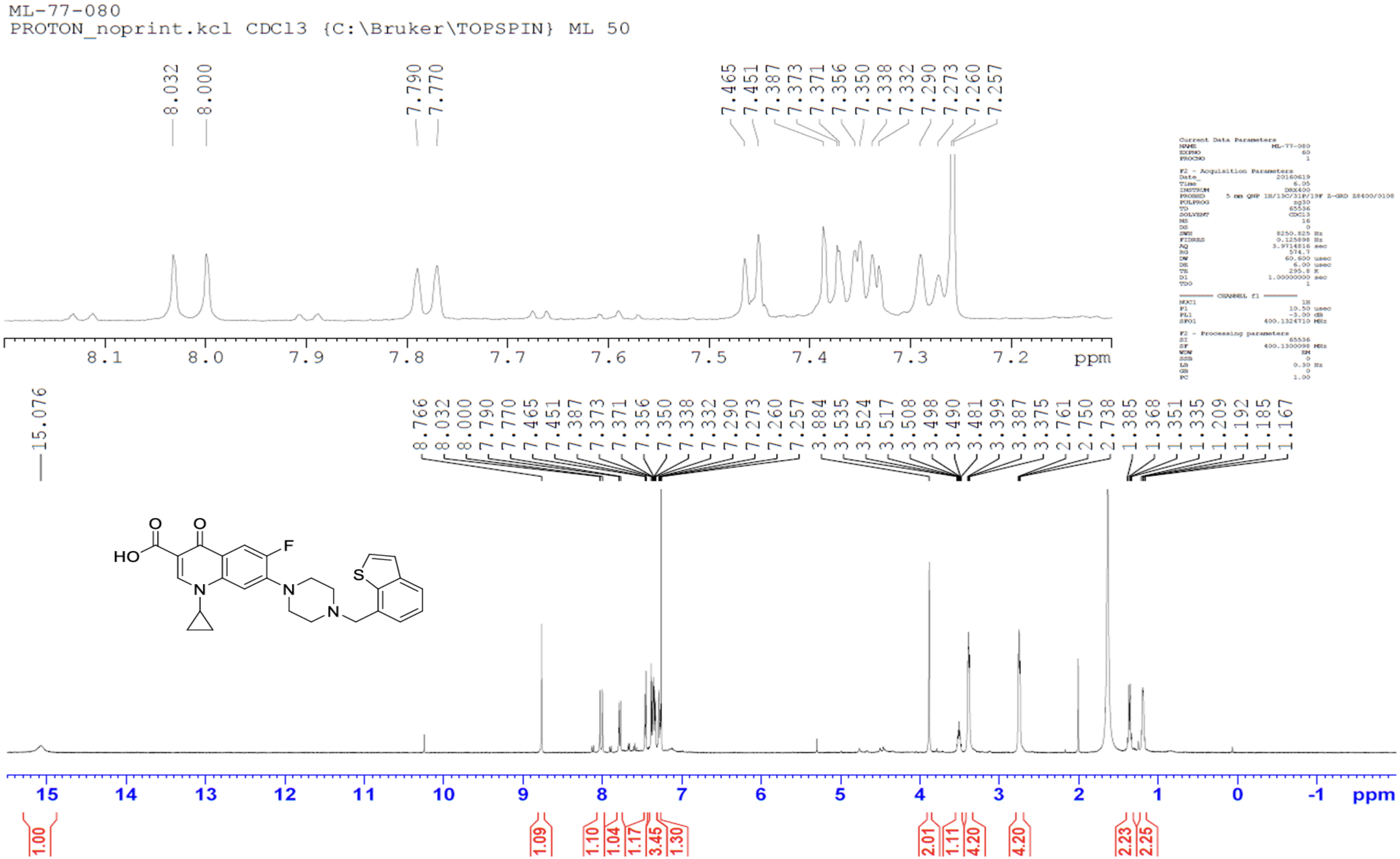

Compound 37 Salt– ^{13}C spectrum

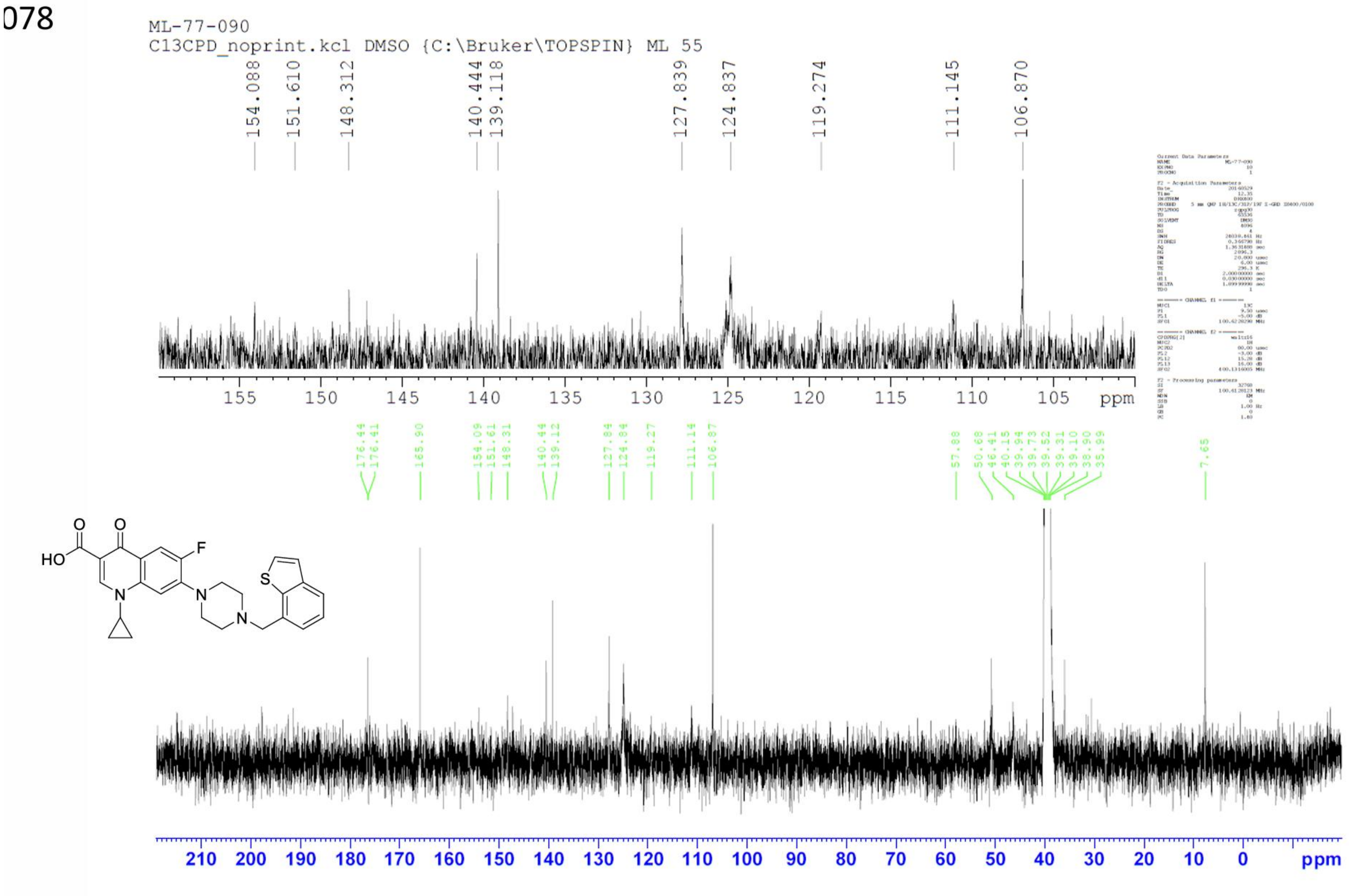

Compound 38– ^{1}H spectrum

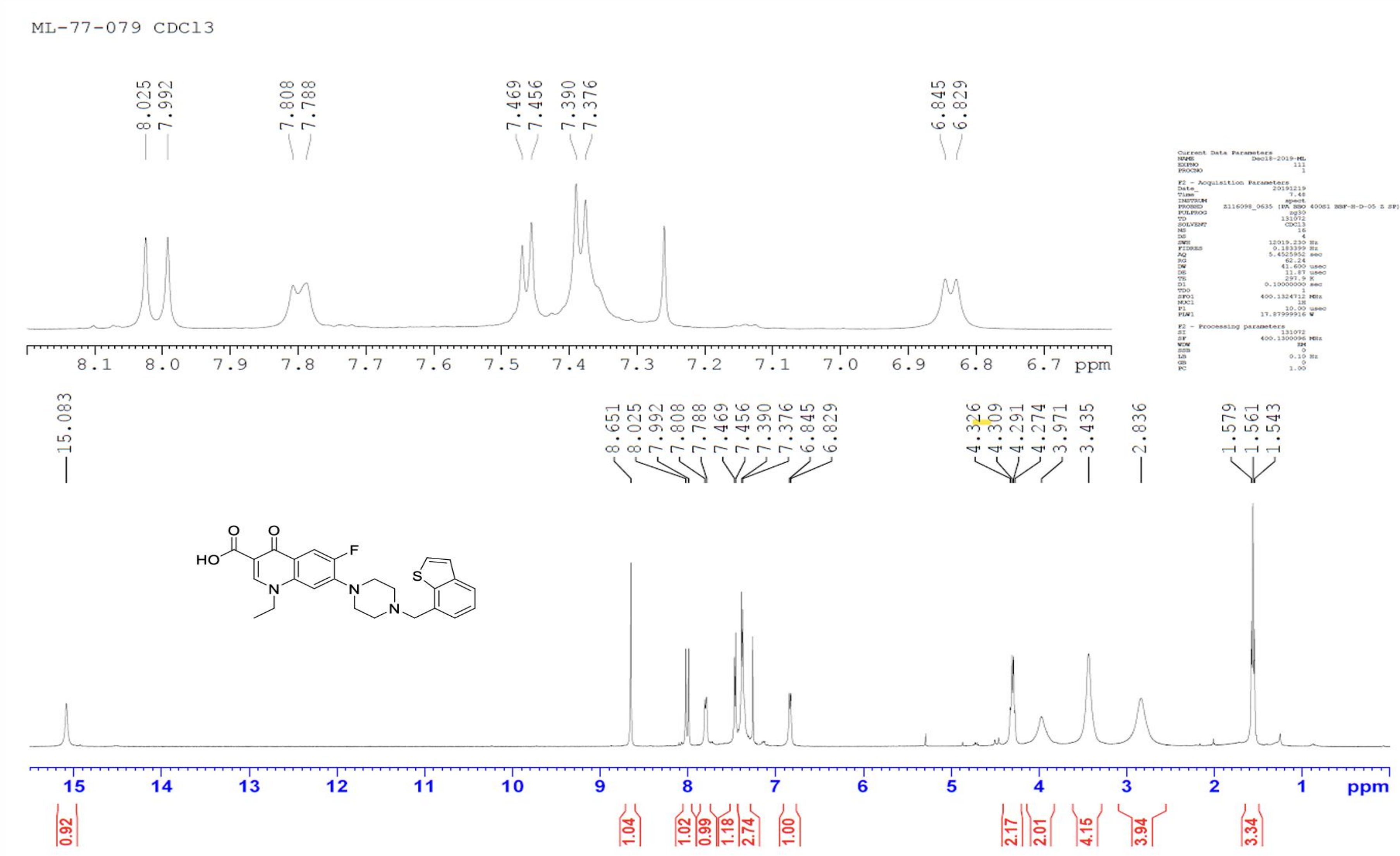

Compound 38– ^{13}C spectrum

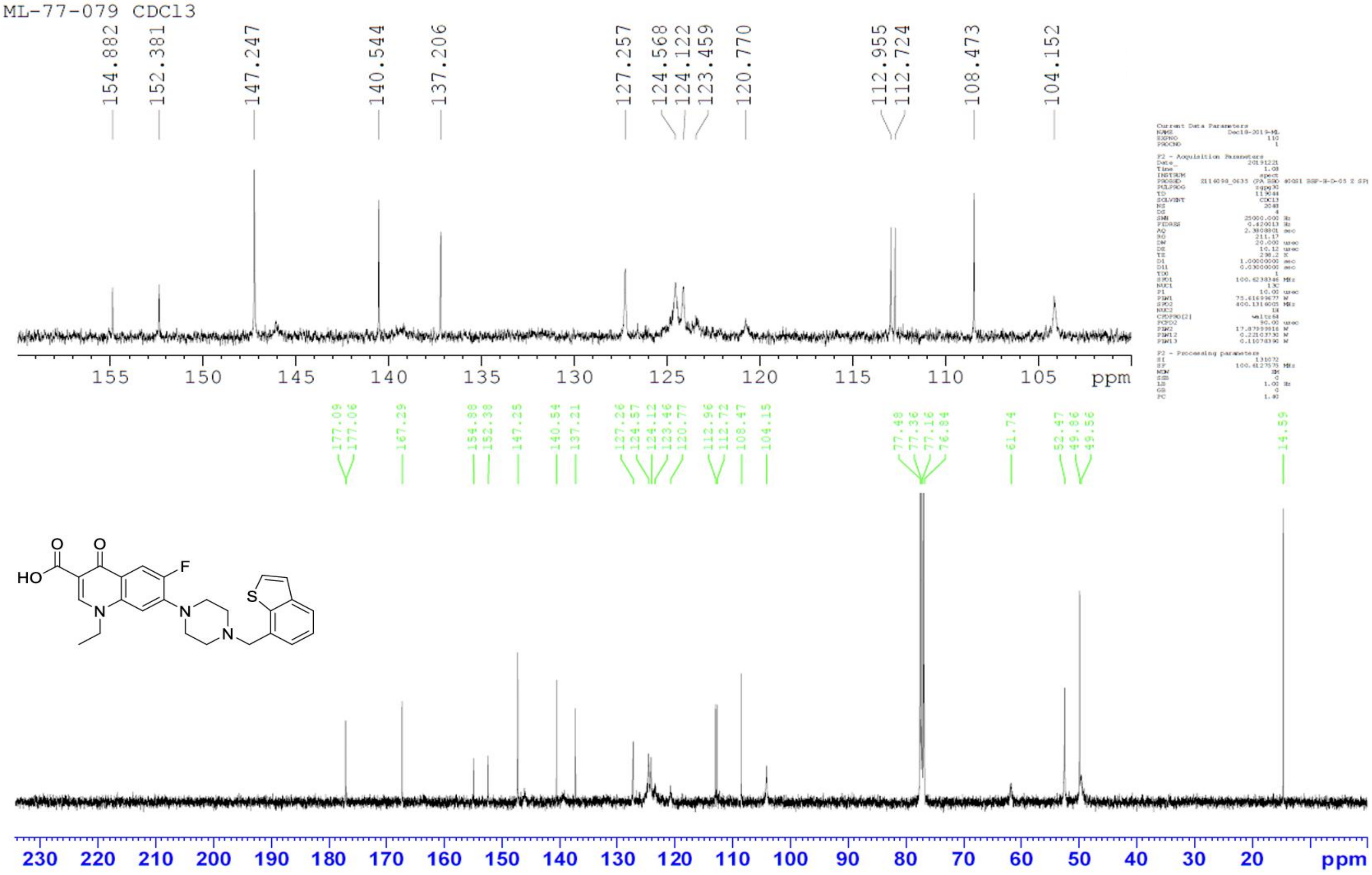

Compound 39– ^{1}H spectrum

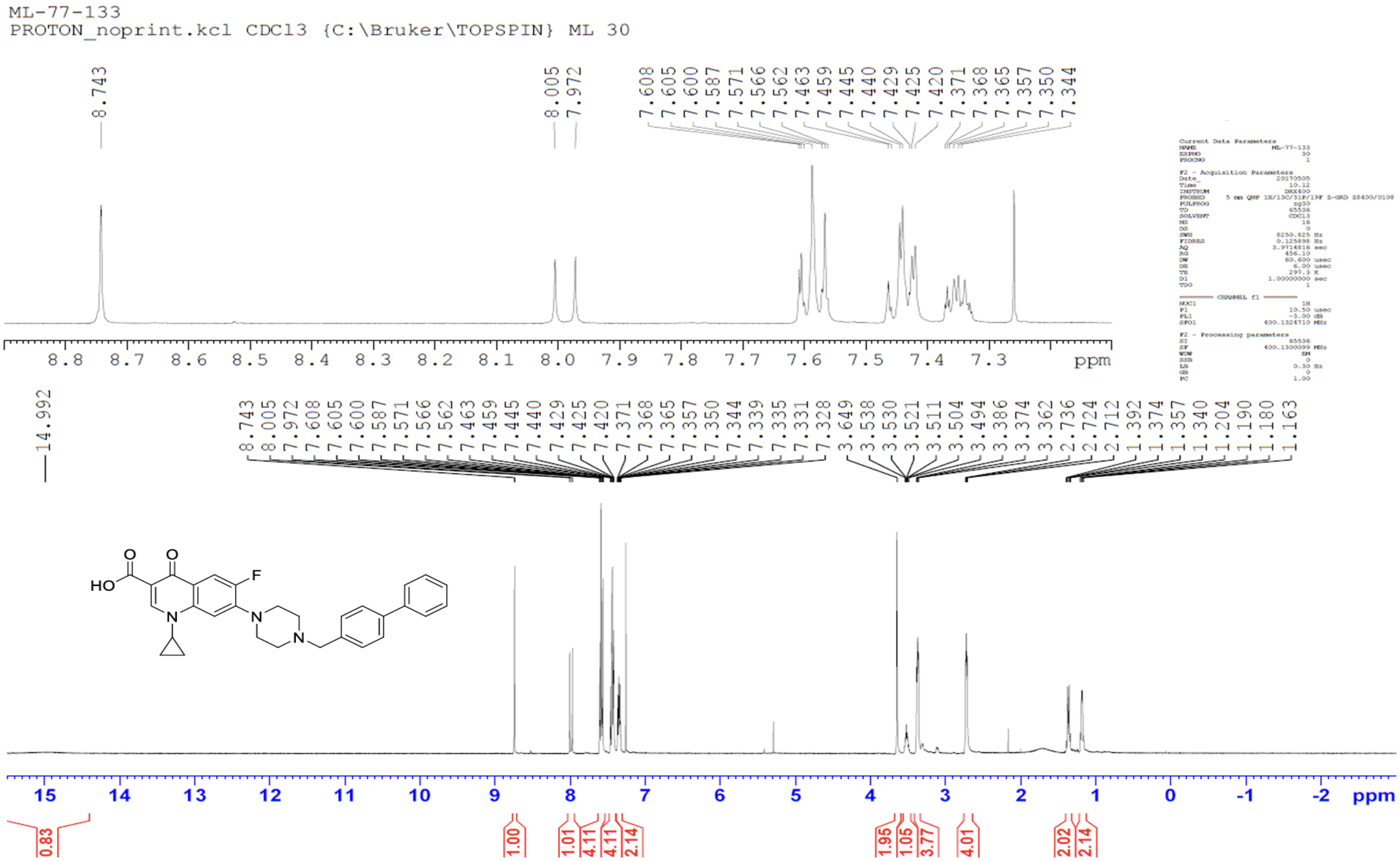

Compound 39– ^{13}C spectrum

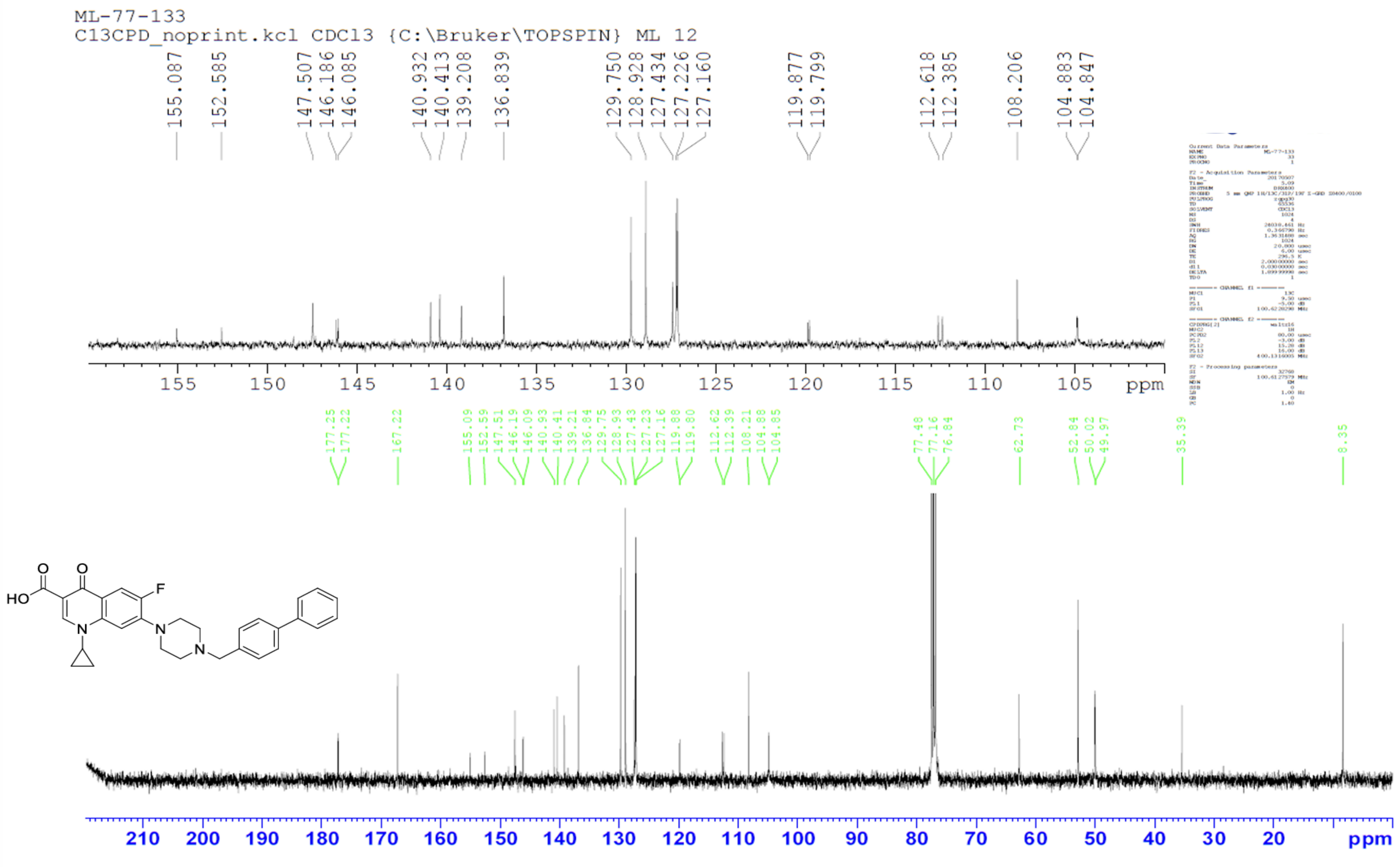

Compound 40– ^{1}H spectrum

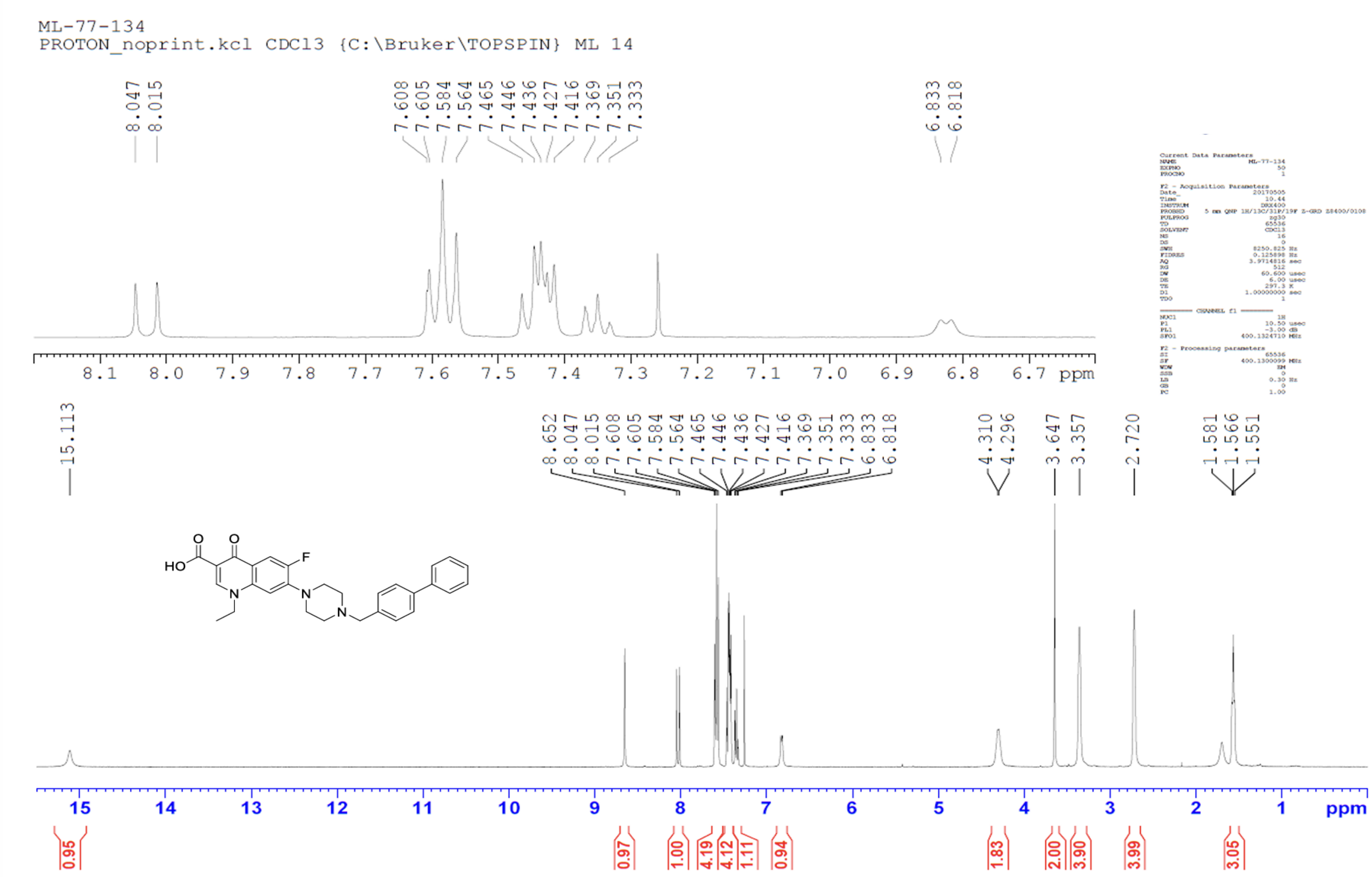

Compound 40– ^{13}C spectrum

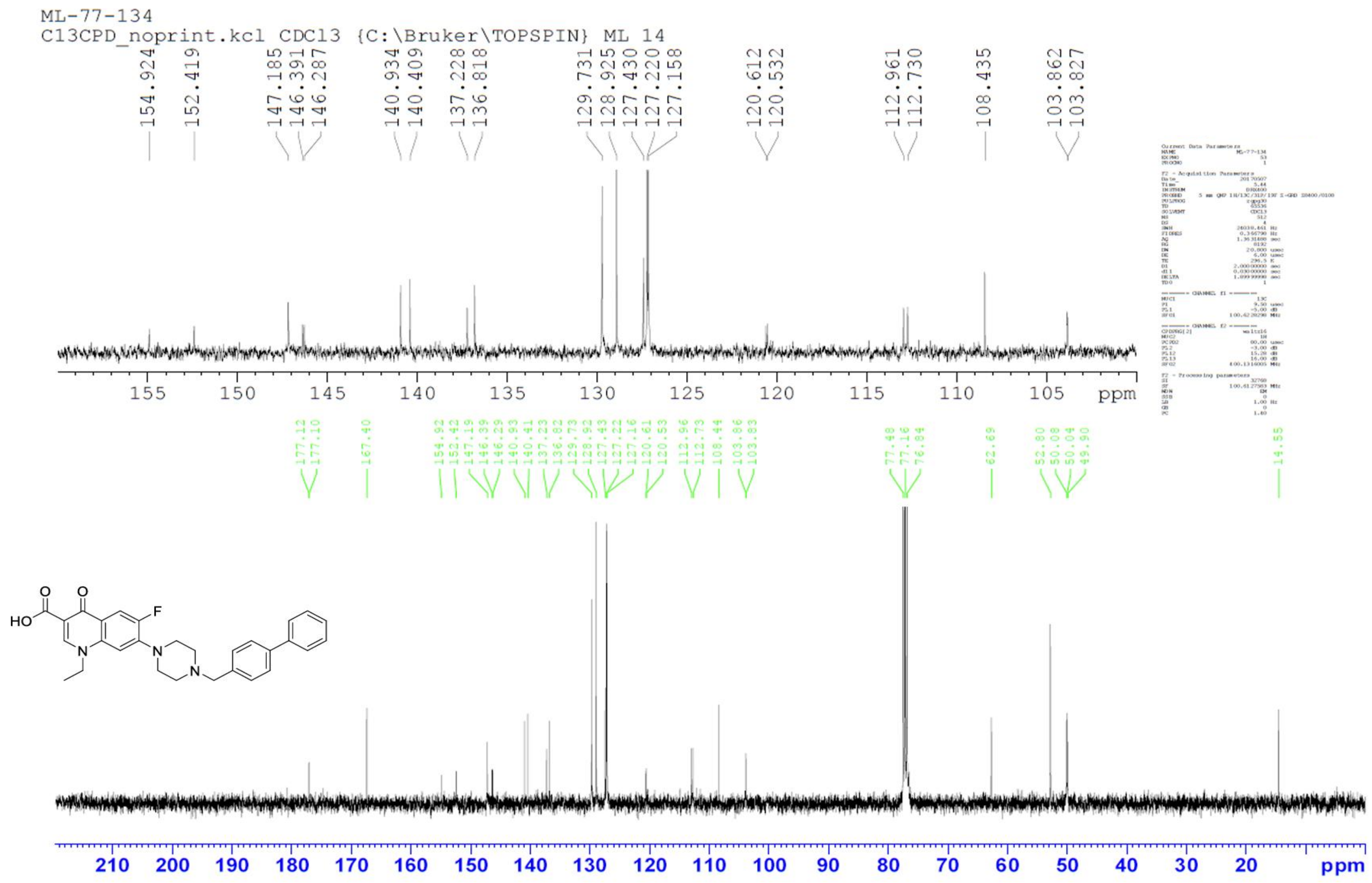

2.2.4 ^{1}H and ^{13}C-NMR Spectra for Second Generation ERB-fluoroquinolones

Compound 22– ^{1}H spectrum

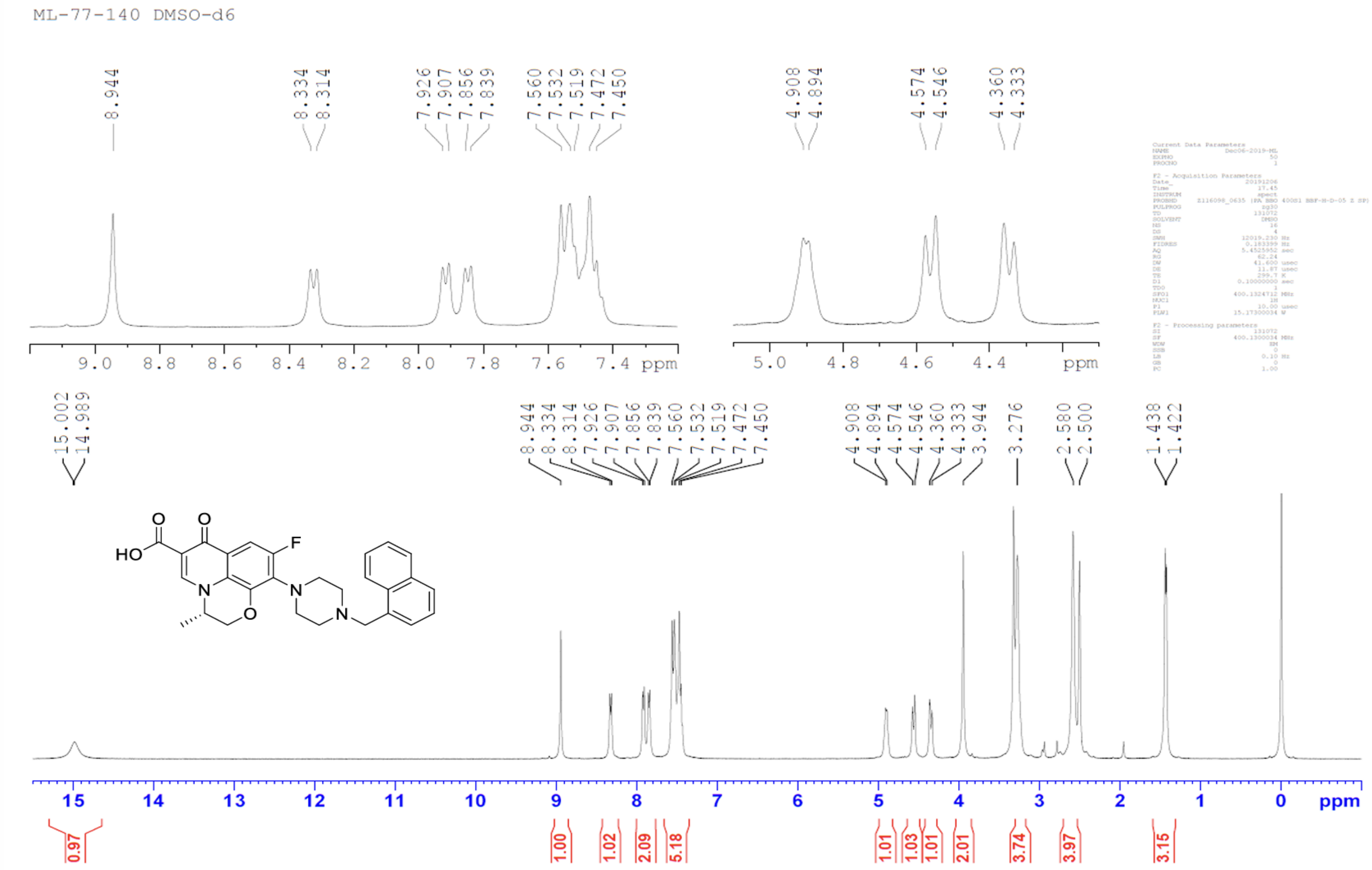

Compound 22– ^{13}C spectrum

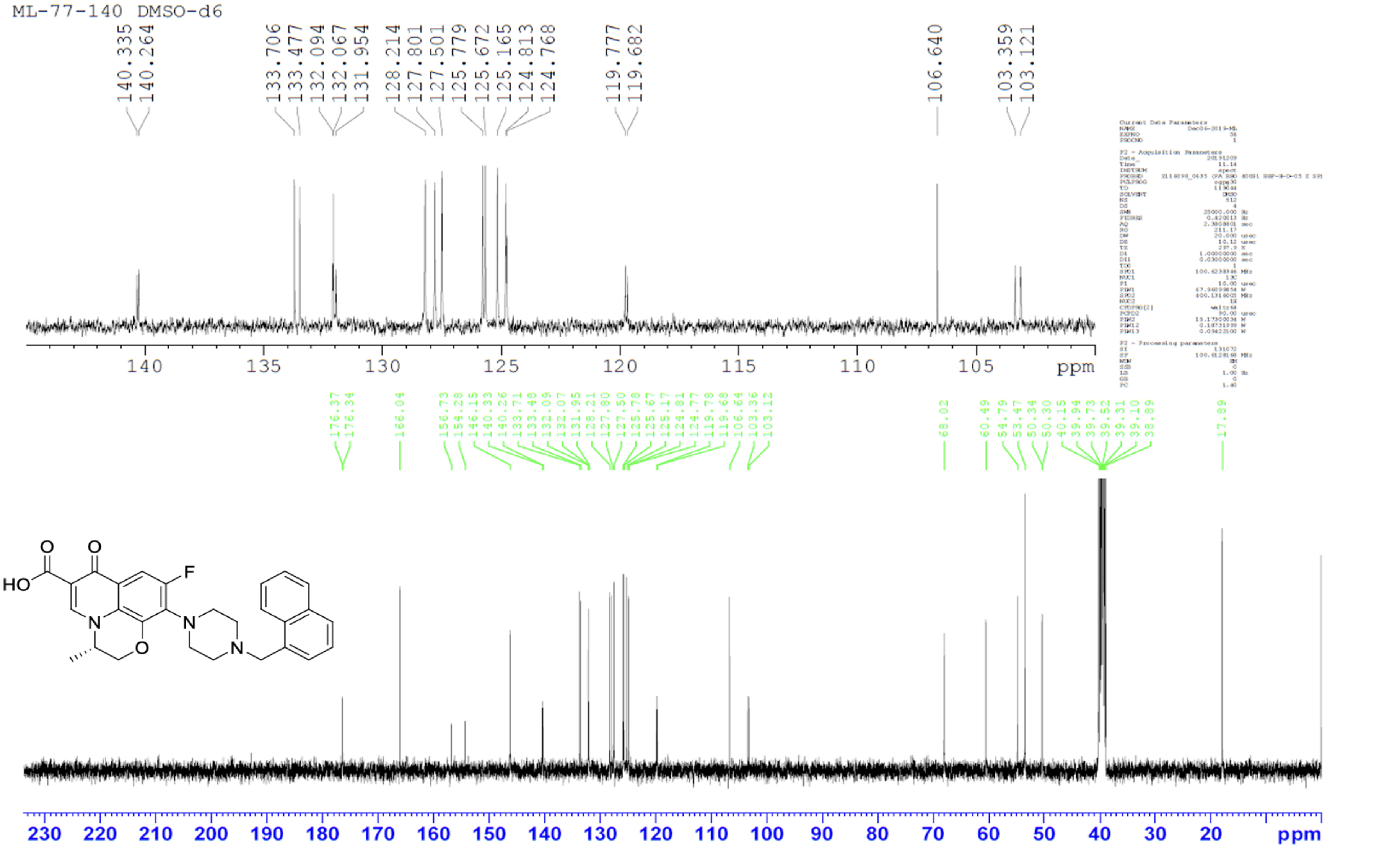

Compound 42– ^{1}H spectrum

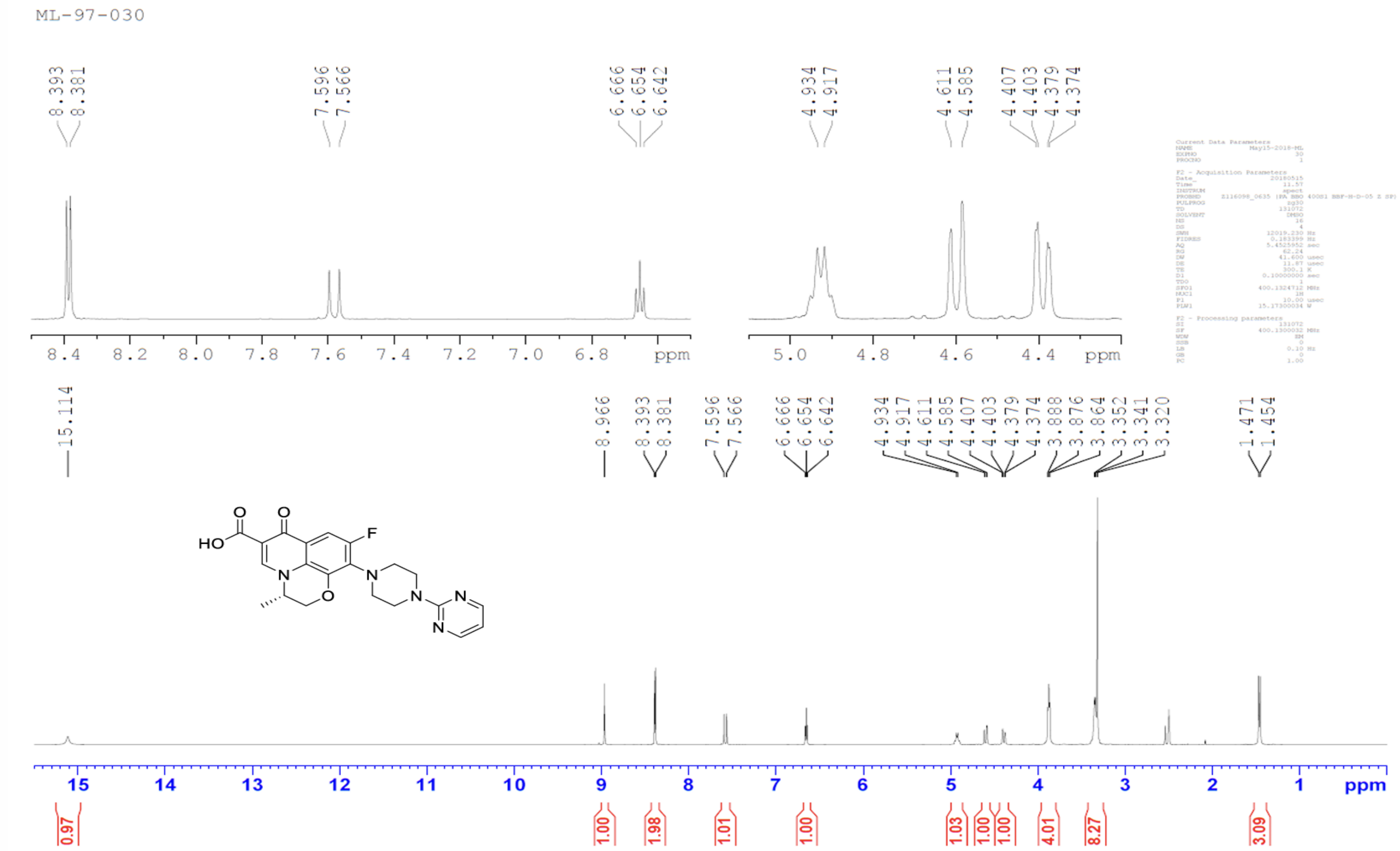

Compound 42– ^{13}C spectrum

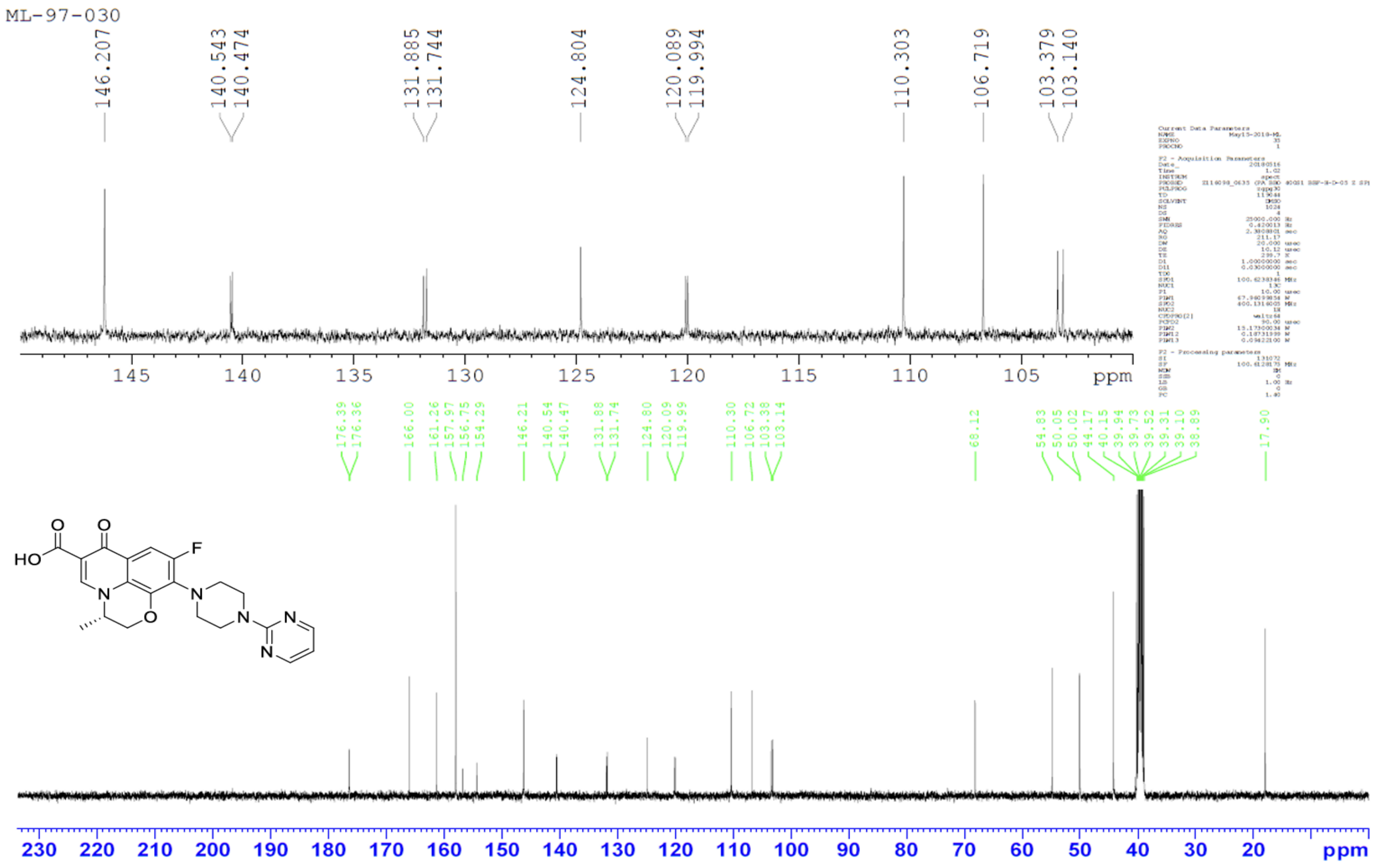

Compound 43– ^{1}H spectrum

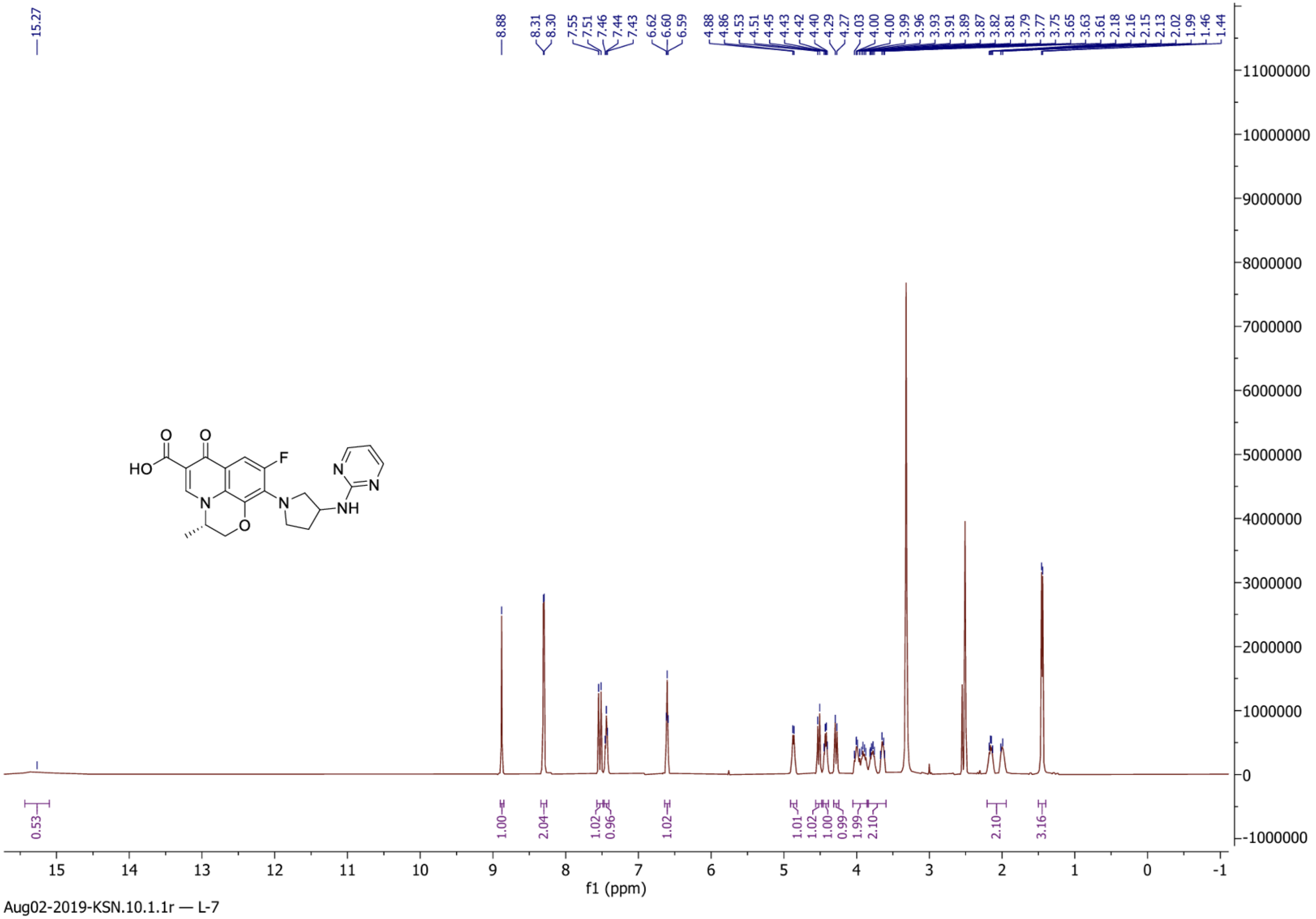

Compound 43– ^{13}C spectrum

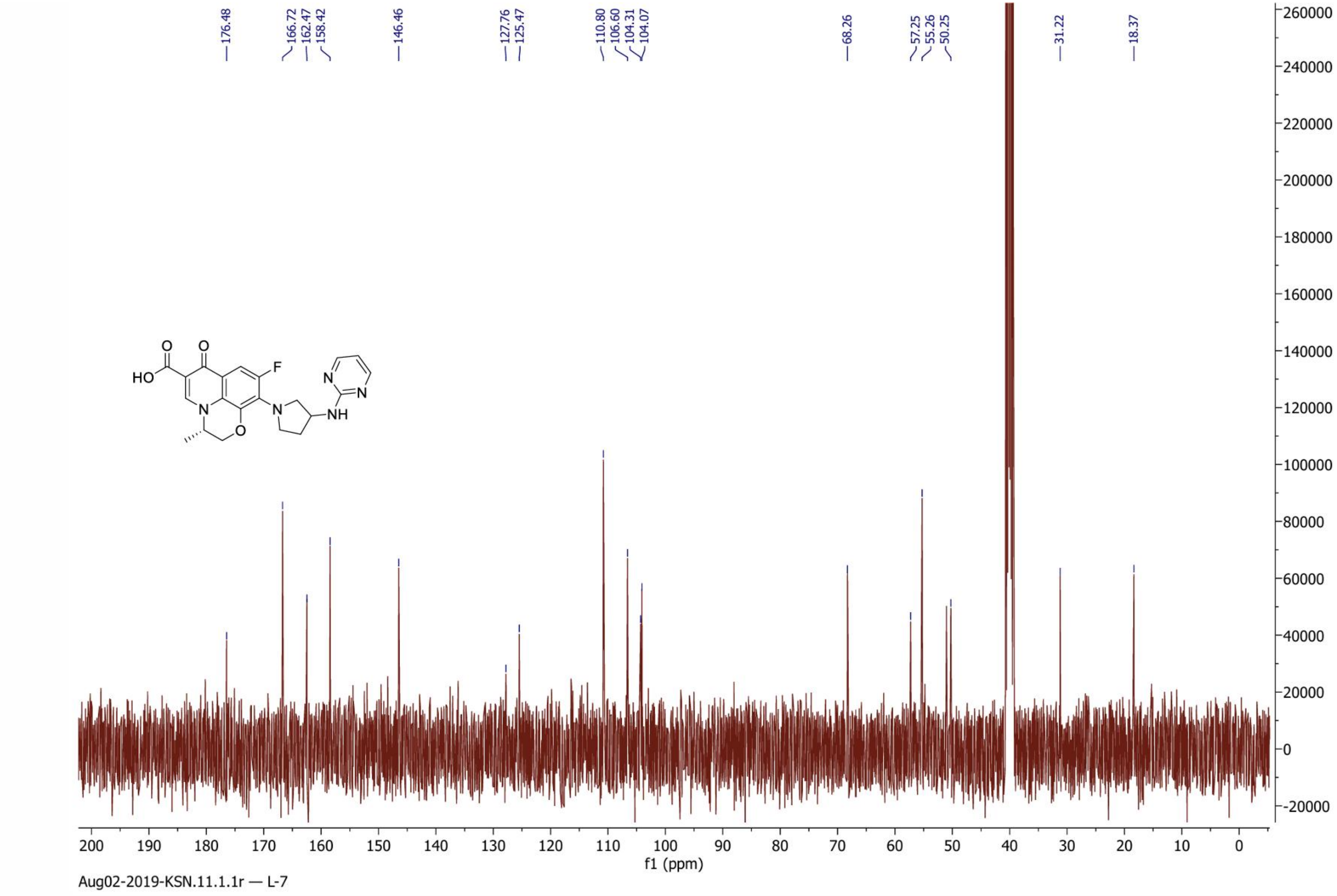

Compound 44– ^{1}H spectrum

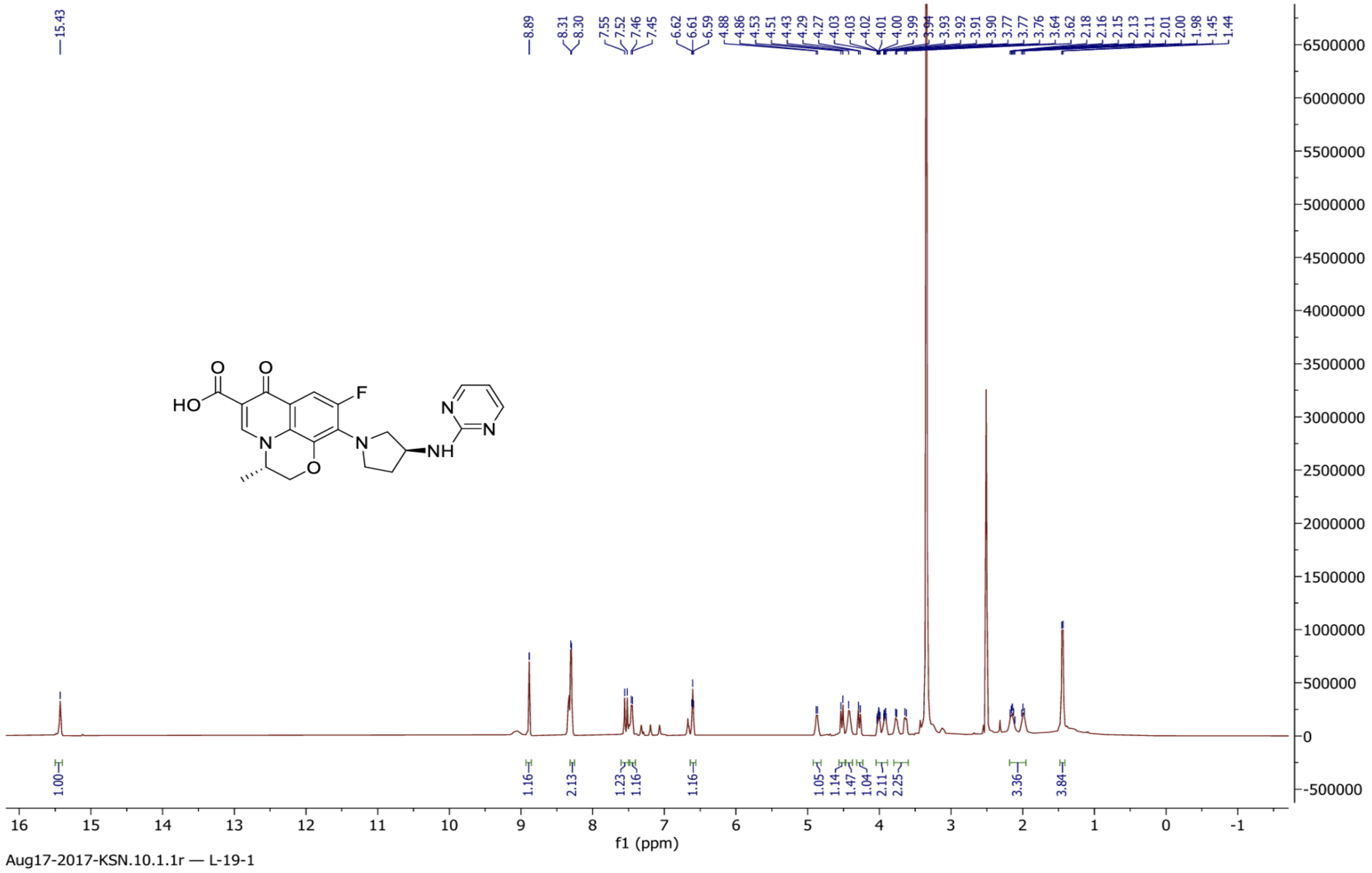

Compound 44– ^{13}C spectrum

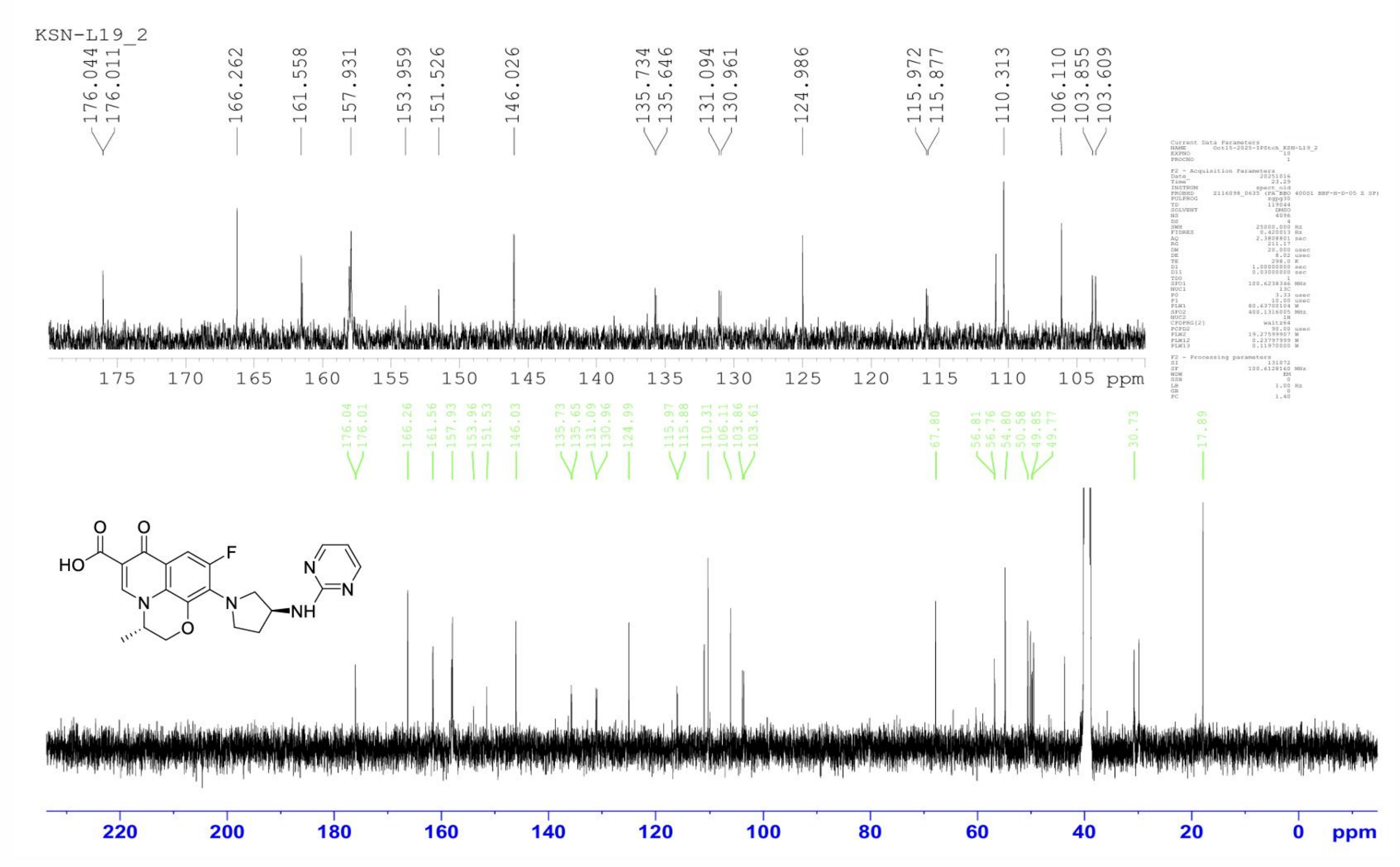

Compound 45– ^{1}H spectrum

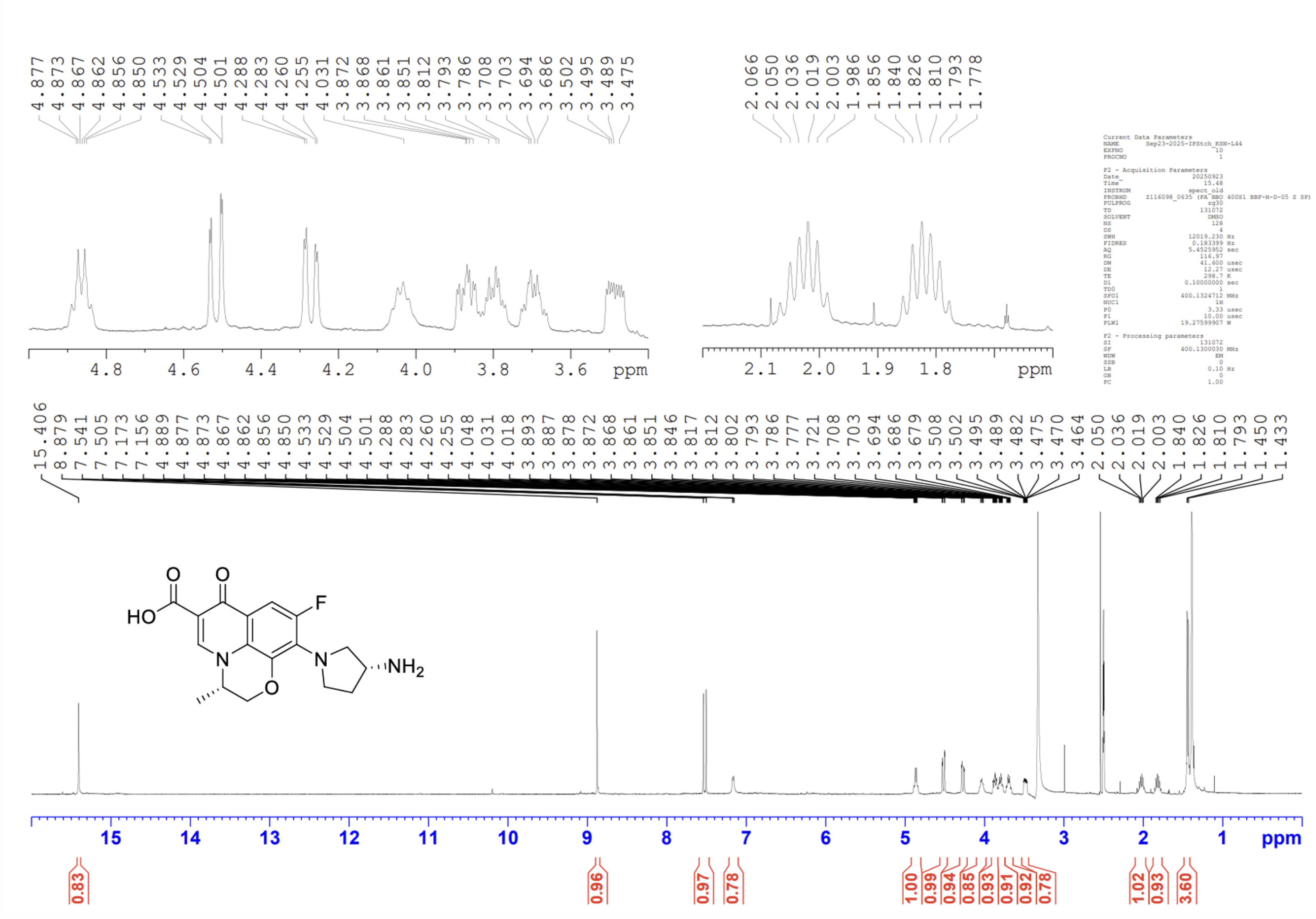

Compound 45– ^{13}C spectrum

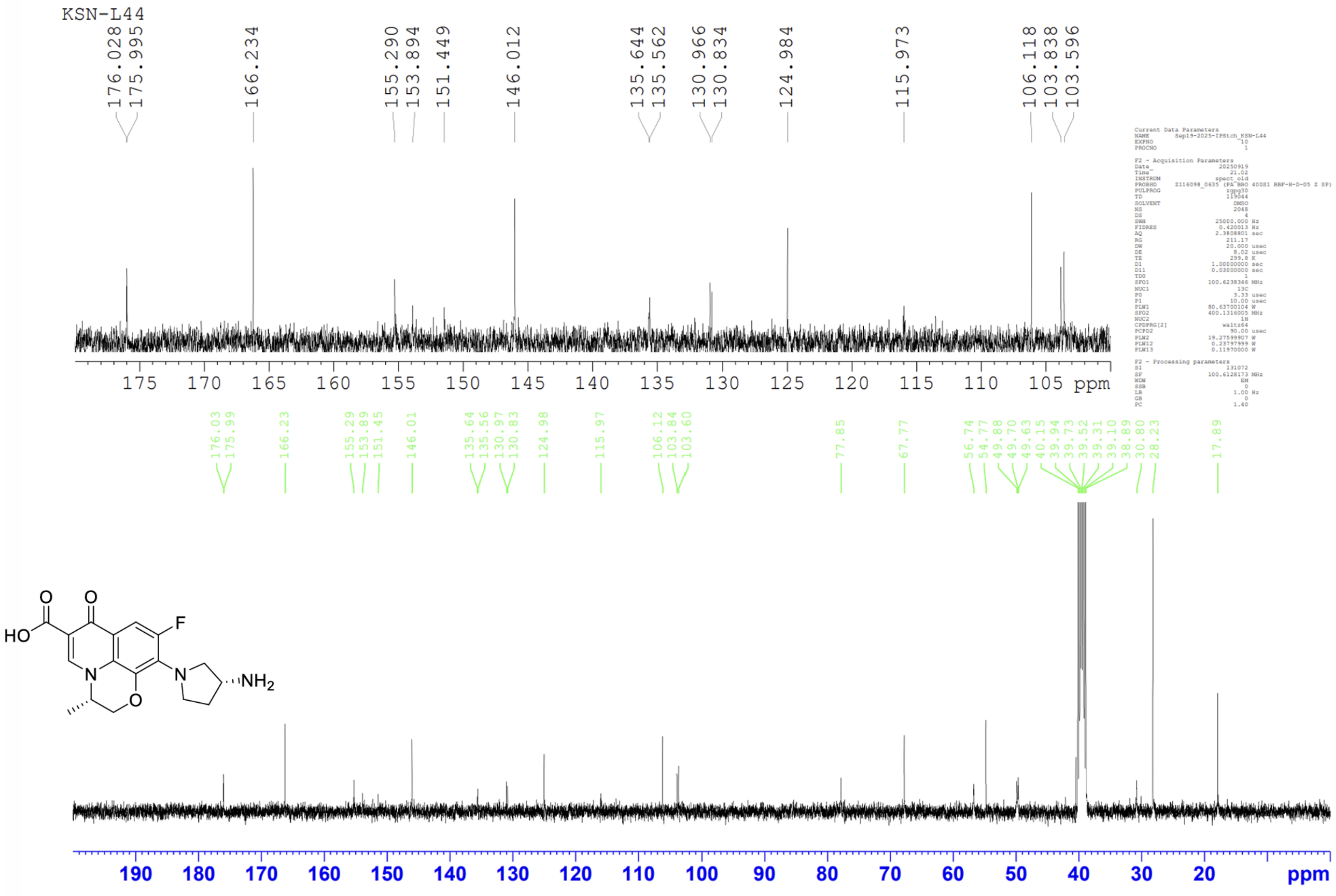

Compound 46 (KSN-L22)– ^{1}H spectrum

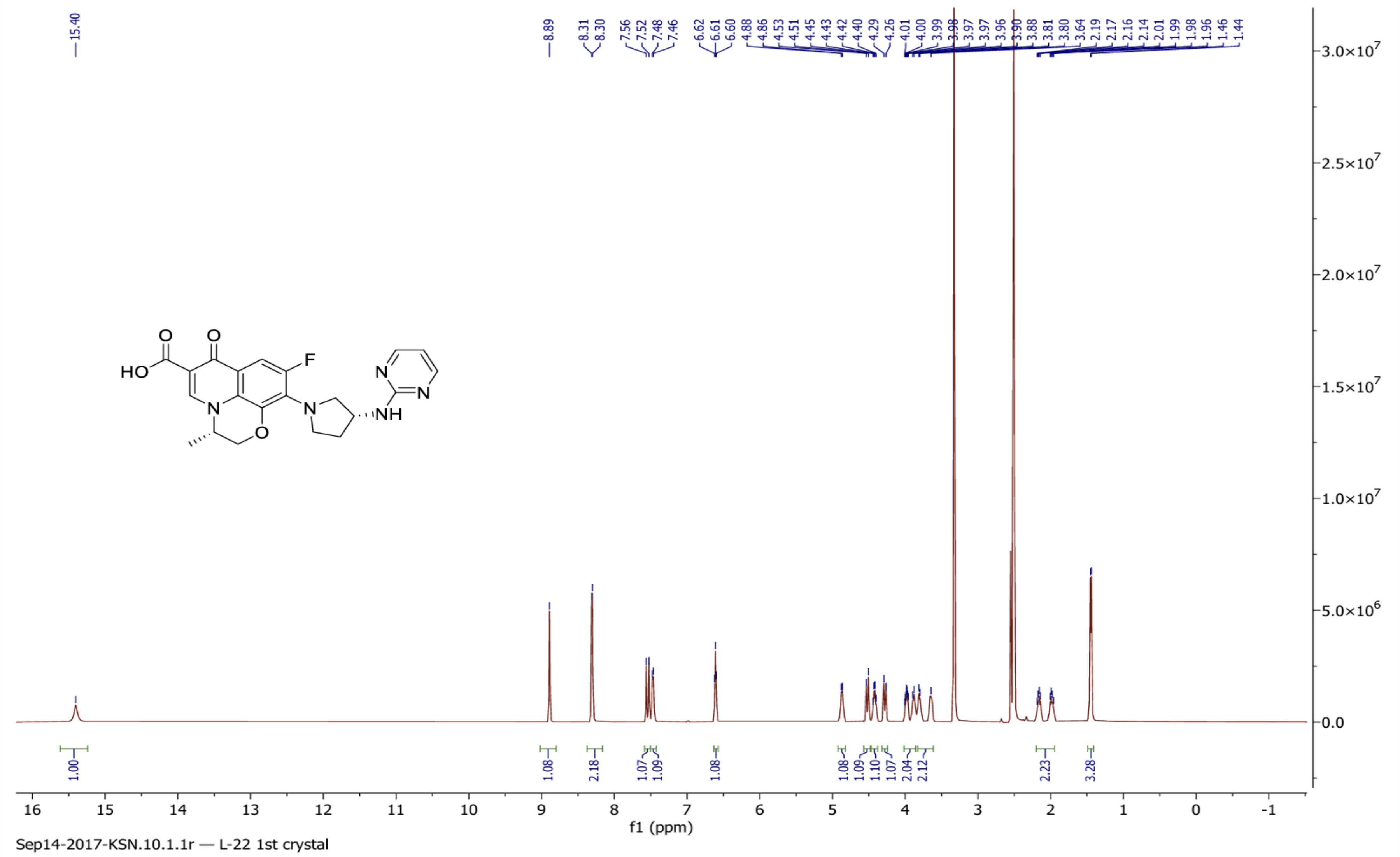

Compound 46 (KSN-L22)– ^{13}C spectrum

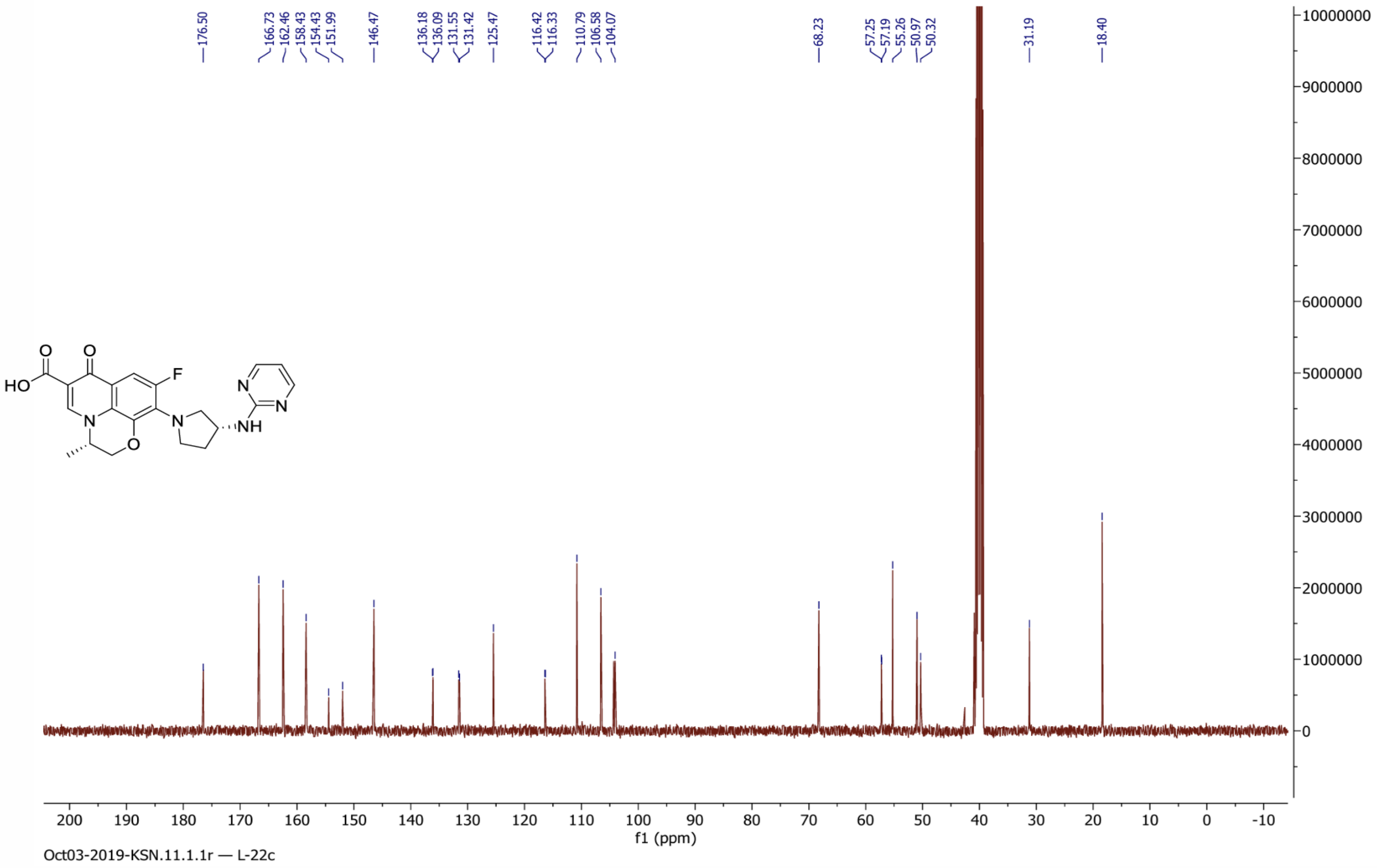

Compound 47– ^{1}H spectrum

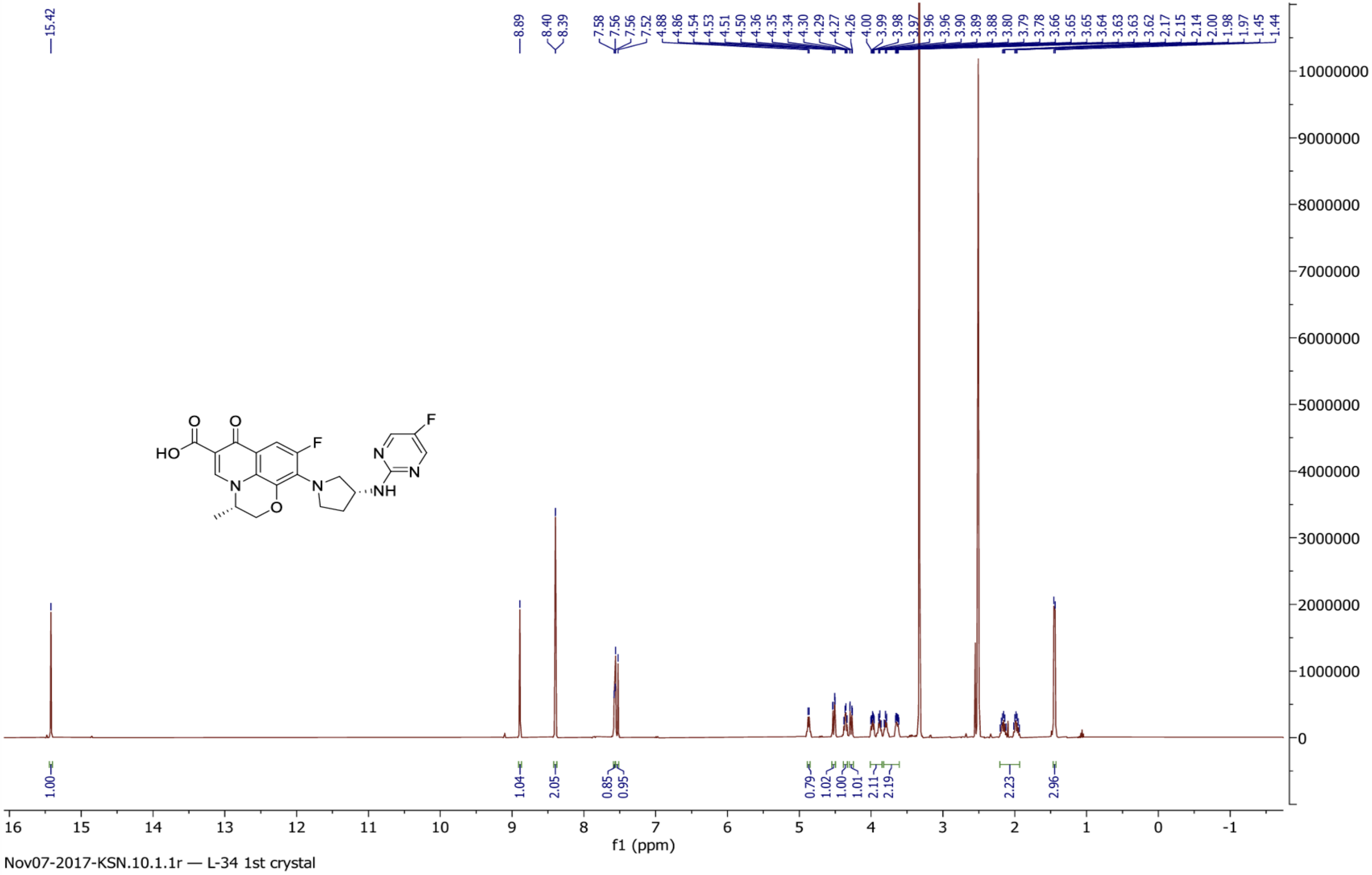

Compound 47– ^{13}C spectrum

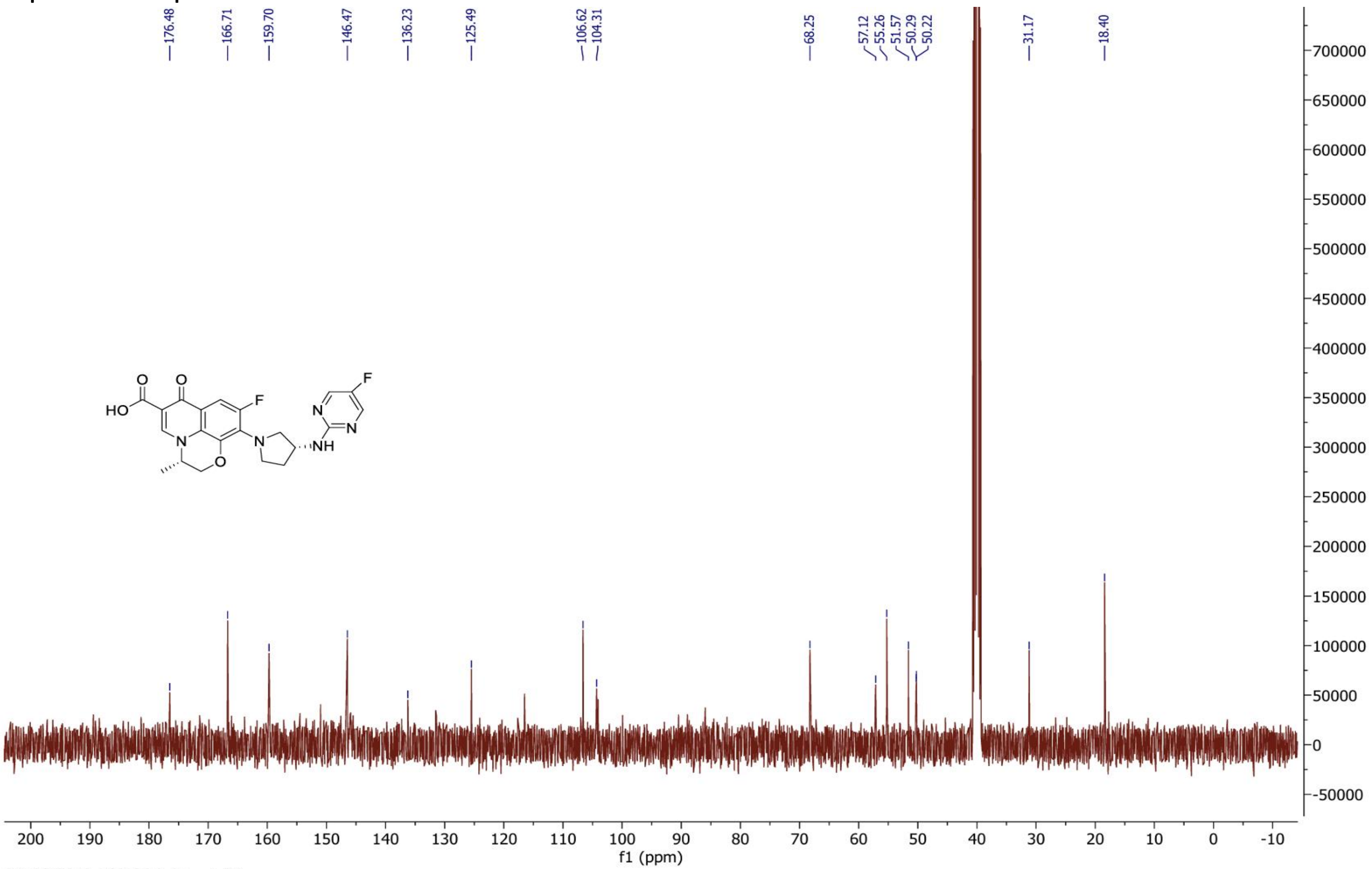

Aug02-2019-KSN.31.1.1r — L-34

Compound 48– ^{1}H spectrum

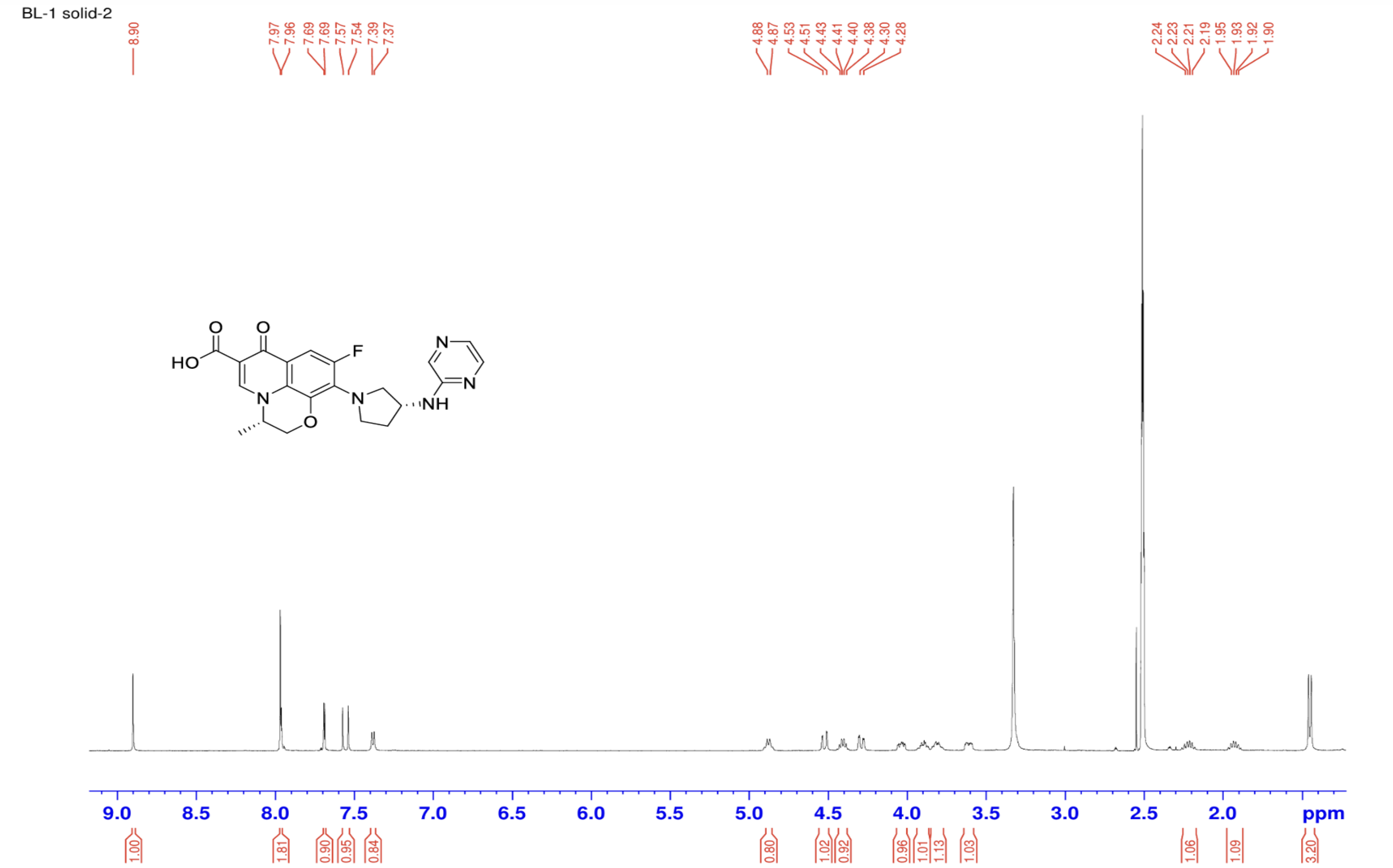

Compound 48– ^{13}C spectrum

BL_1

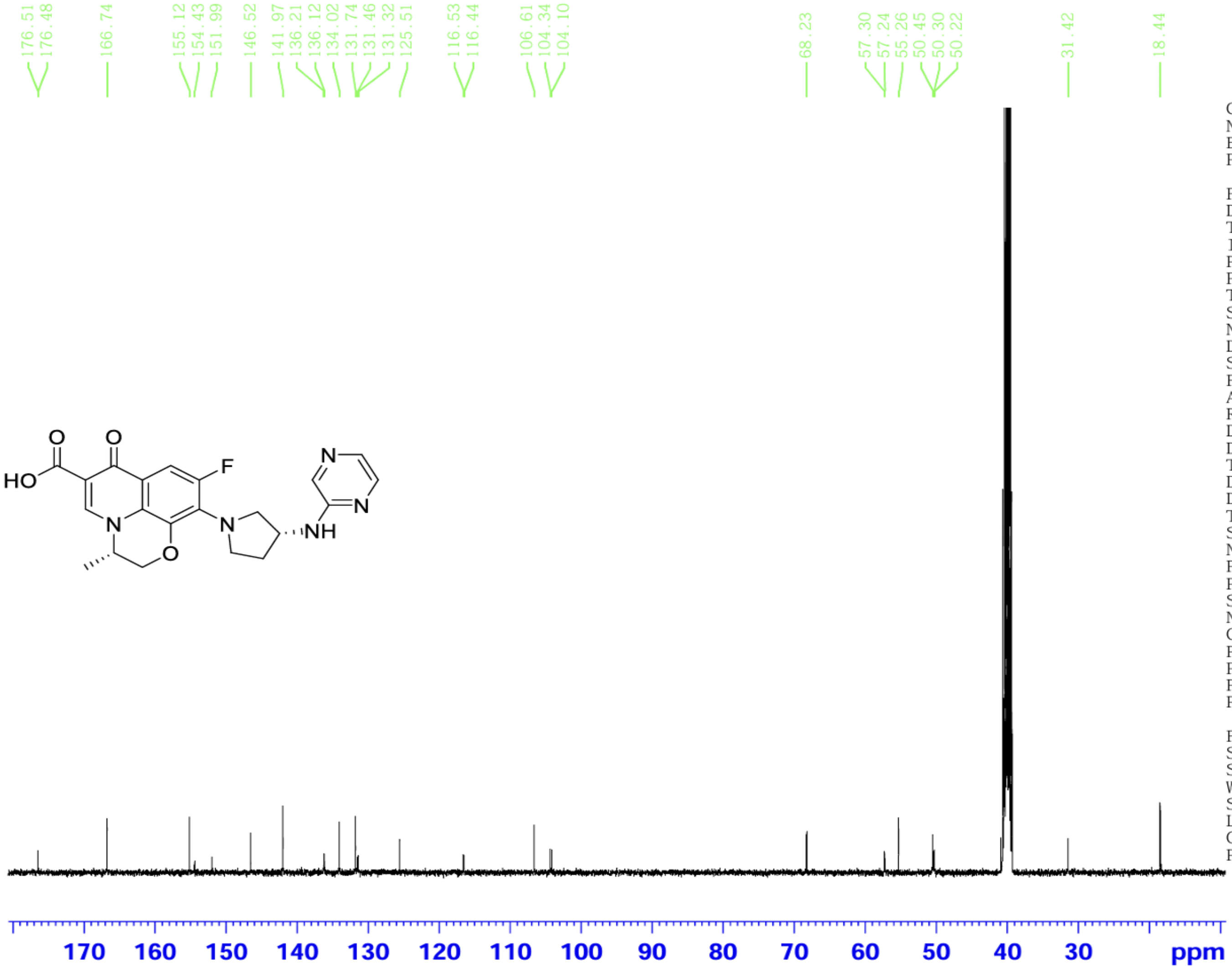

Current Data Parameters
NAME Mar05-2018-KSN
EXPNO 12
PROCNO 1

F2 - Acquisition Parameters
Date_ 20180305
Time 22.53 h
INSTRUM spect
PROBHD Z116098_0635 (
PULPROG zgpg30
TD 119044
SOLVENT DMSO
NS 4000
DS 4
SWH 25000.000 Hz
FIDRES 0.420013 Hz
AQ 2.3808801 sec
RG 211.17
DW 20.000 usec
DE 10.12 usec
TE 298.0 K
D1 1.00000000 sec
D11 0.03000000 sec
TD0 1
SFO1 100.6238346 MHz
NUC1 13C
P1 10.00 usec
PLW1 67.96099854 W
SFO2 400.1316005 MHz
NUC2 1H
CPDPRG[2 waltz64
PCPD2 90.00 usec
PLW2 15.17300034 W
PLW12 0.18731999 W
PLW13 0.09422100 W

F2 - Processing parameters
SI 131072
SF 100.6127685 MHz
WDW EM
SSB 0
LB 1.00 Hz
GB 0
PC 1.40

Compound 49 – ^{1}H spectrum

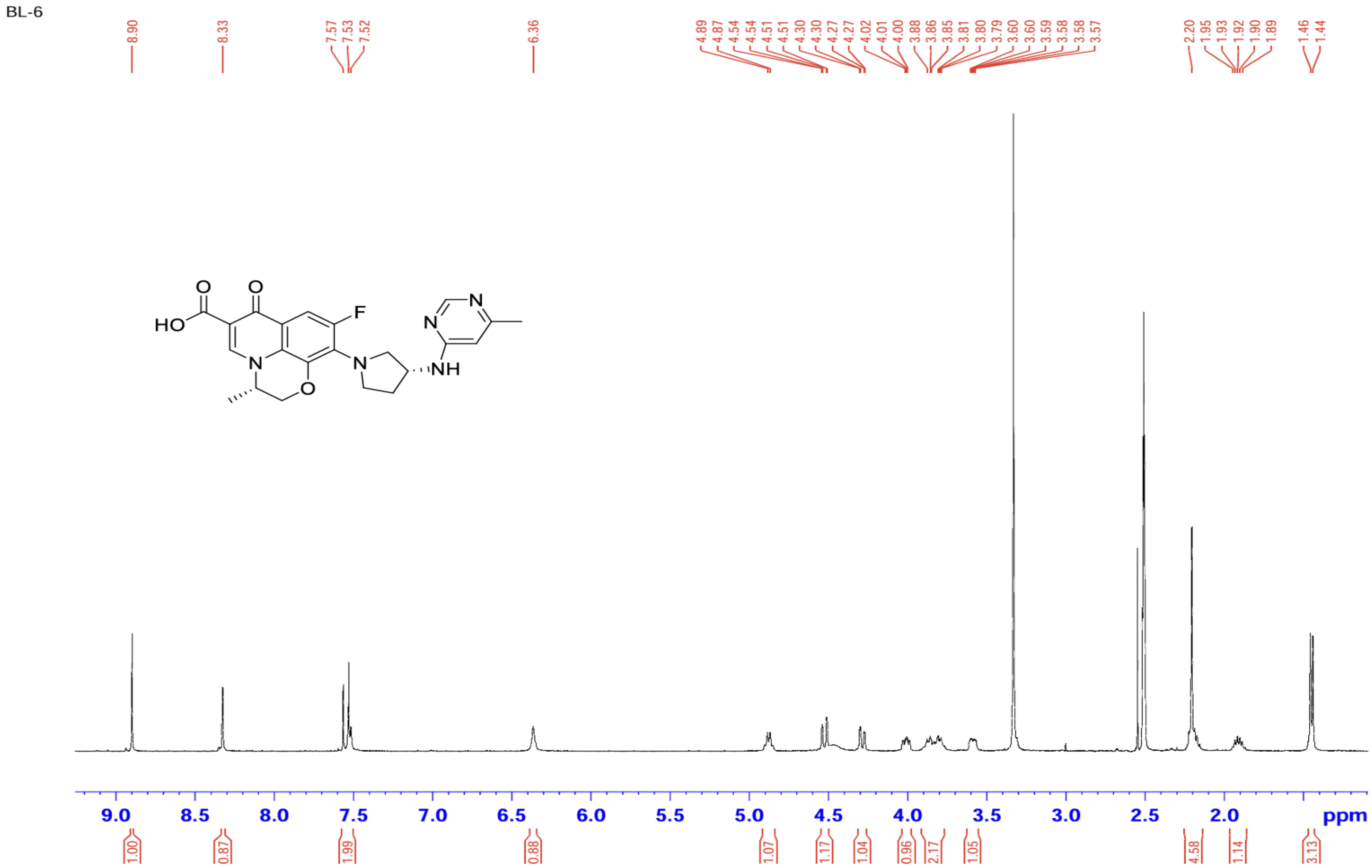

Compound 49– ^{13}C spectrum

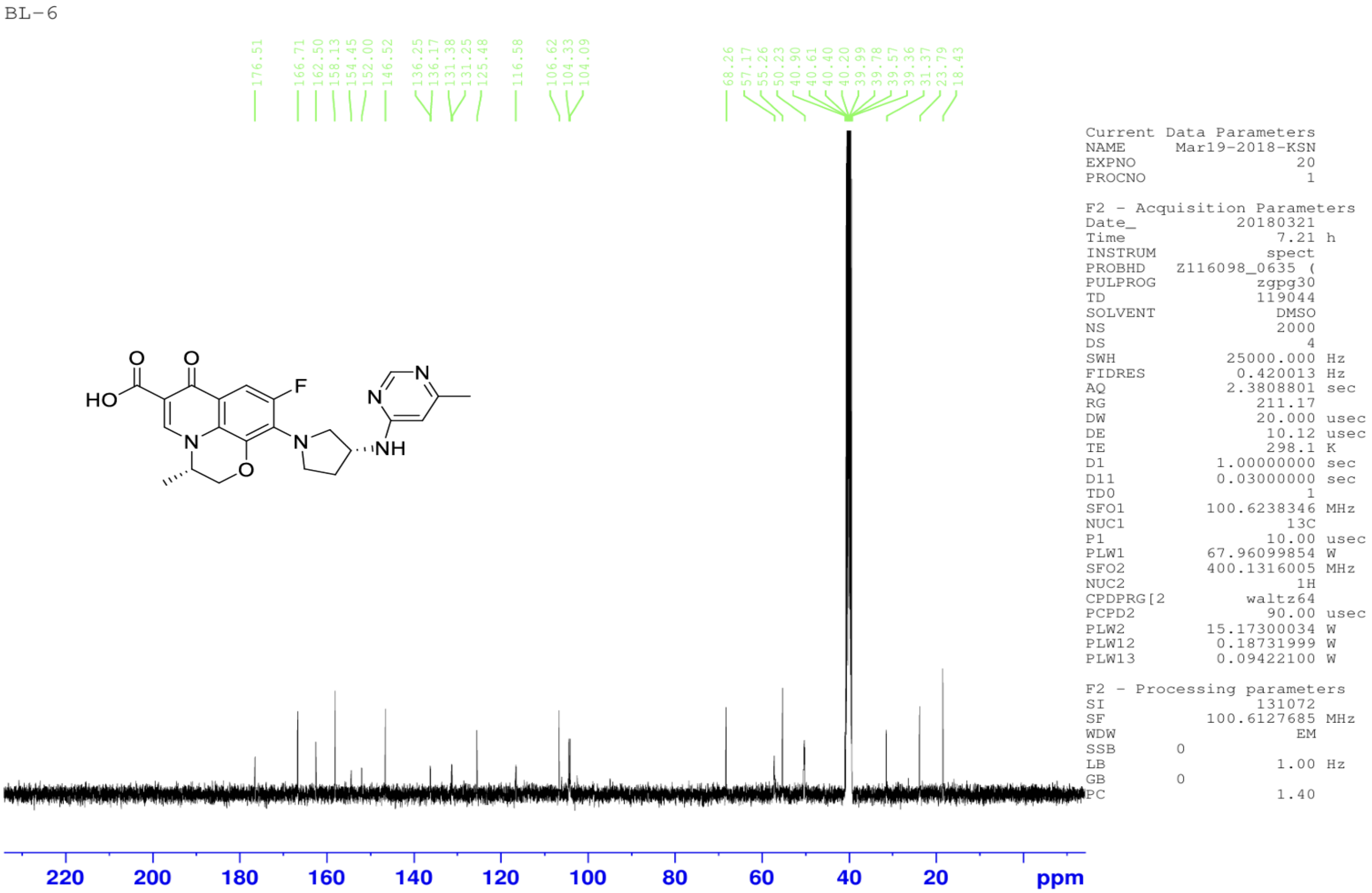

Compound 50 (BL-7)– ¹H spectrum

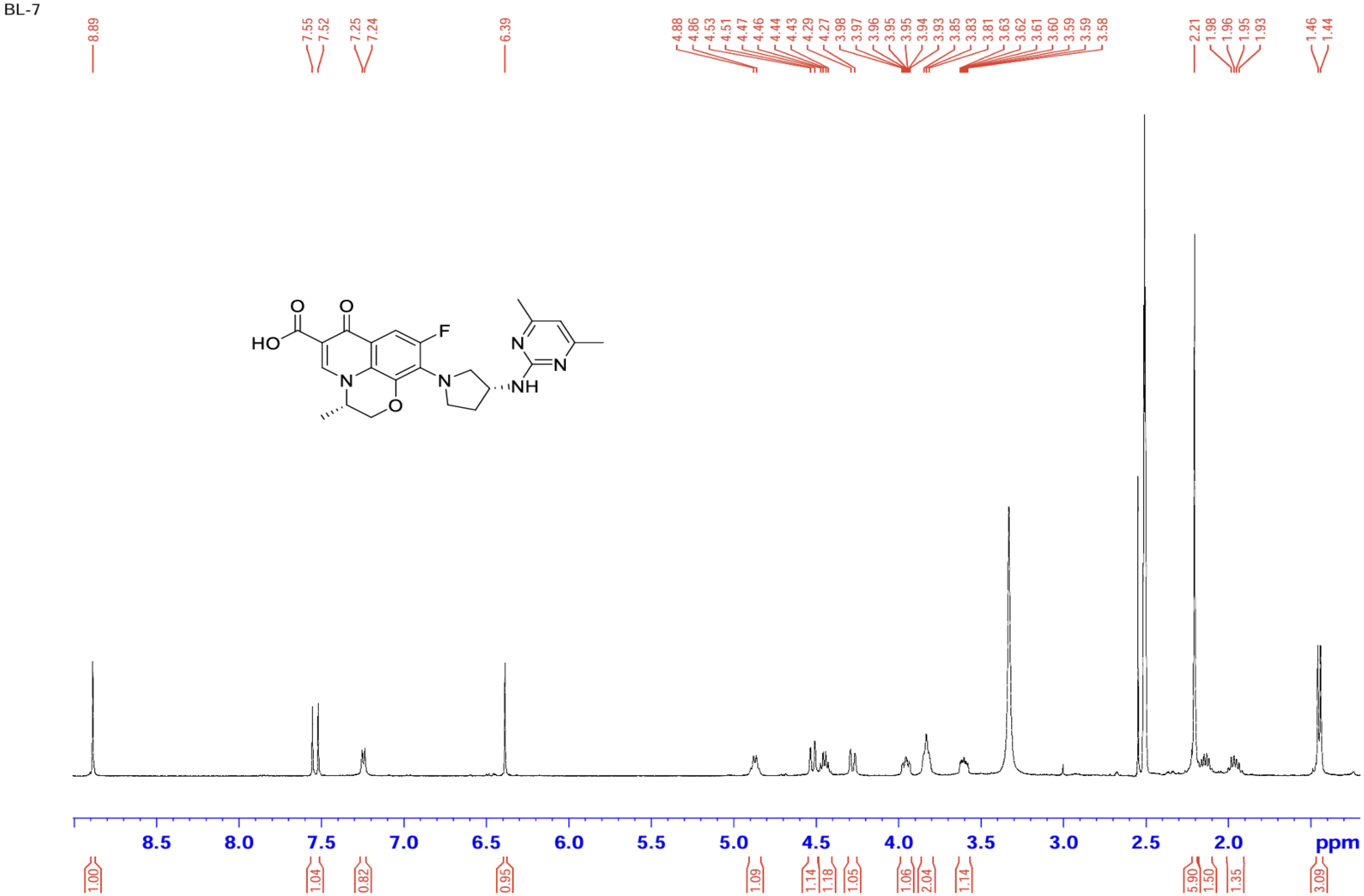

Compound 50 (BL-7)– ^{13}C spectrum

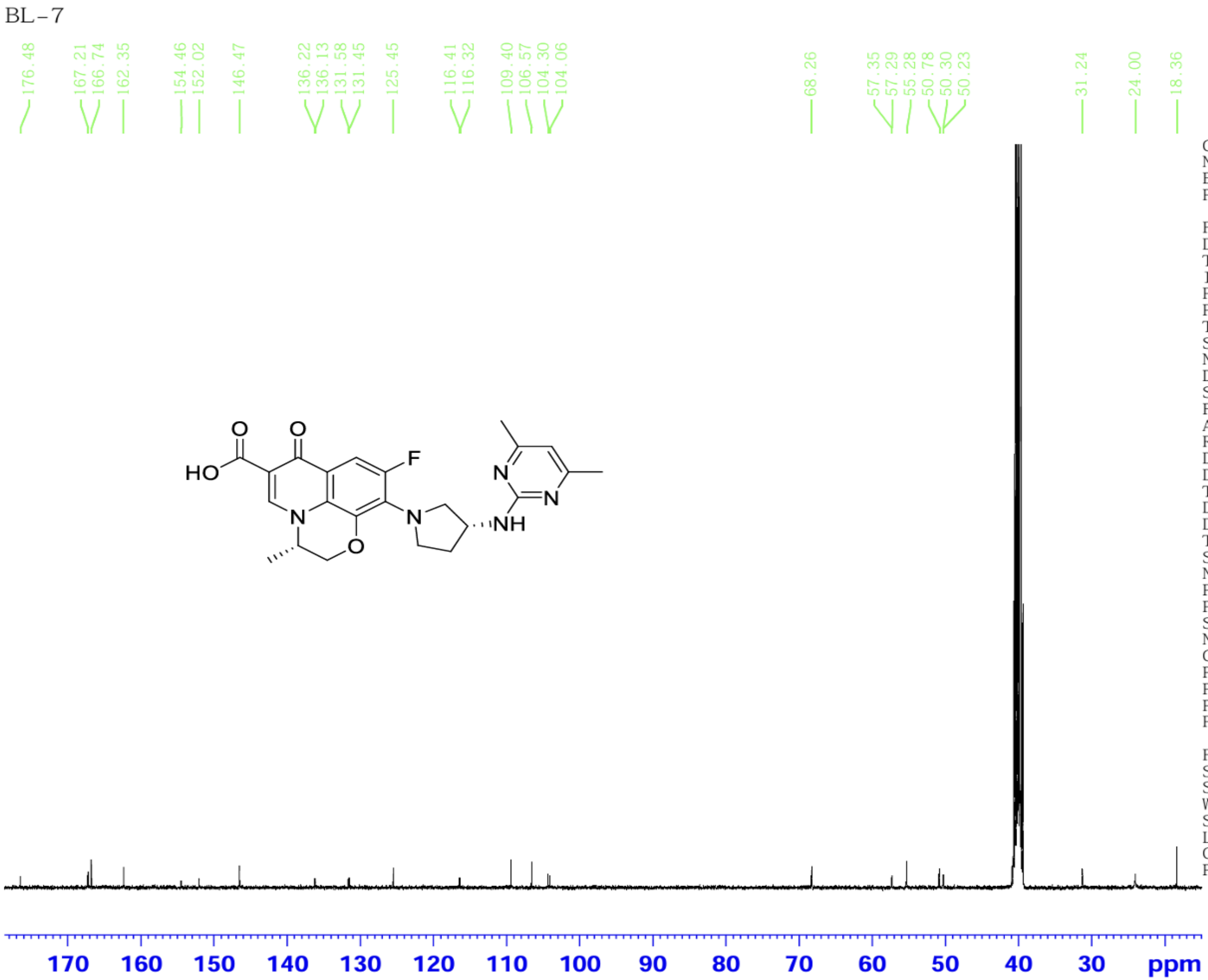

Current Data Parameters
NAME Mar15-2018-KSN
EXPNO 32
PROCNO 1

F2 - Acquisition Parameters
Date_ 20180316
Time 2.42 h
INSTRUM spect
PROBHD Z116098_0635 (
PULPROG zgpg30
TD 119044
SOLVENT DMSO
NS 3000
DS 4
SWH 25000.000 Hz
FIDRES 0.420013 Hz
AQ 2.3808801 sec
RG 211.17
DW 20.000 usec
DE 10.12 usec
TE 298.4 K
D1 1.00000000 sec
D11 0.03000000 sec
TDO 1
SFO1 100.6238346 MHz
NUC1 13C
P1 10.00 usec
PLW1 67.96099854 W
SFO2 400.1316005 MHz
NUC2 1H
CPDPRG[2 waltz64
PCPD2 90.00 usec
PLW2 15.17300034 W
PLW12 0.18731999 W
PLW13 0.09422100 W

F2 - Processing parameters
SI 131072
SF 100.6127685 MHz
WDW EM
SSB 0
LB 1.00 Hz
GB 0
PC 1.40

Compound 51– ^{1}H spectrum

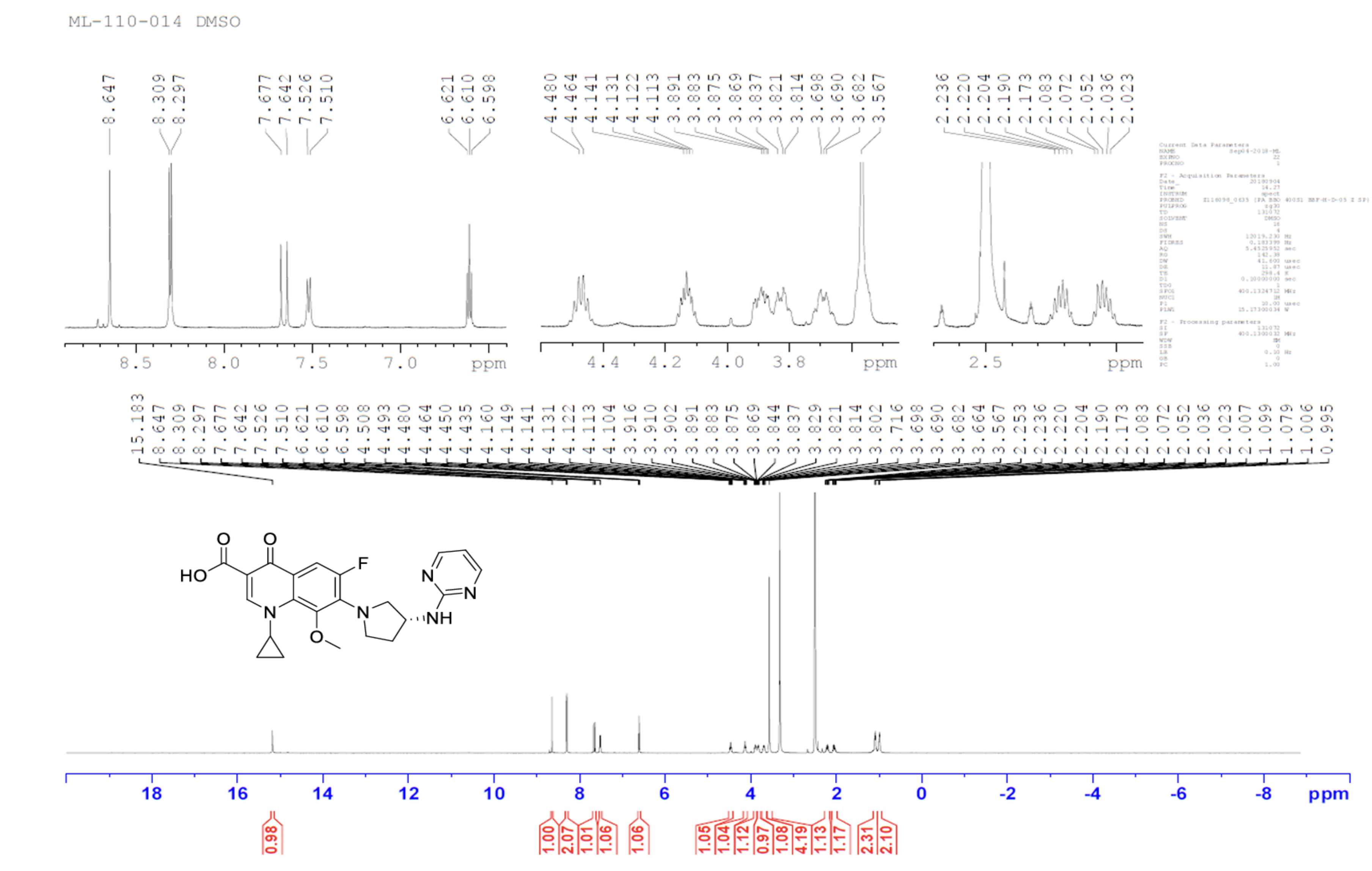

Compound 51– ^{13}C spectrum

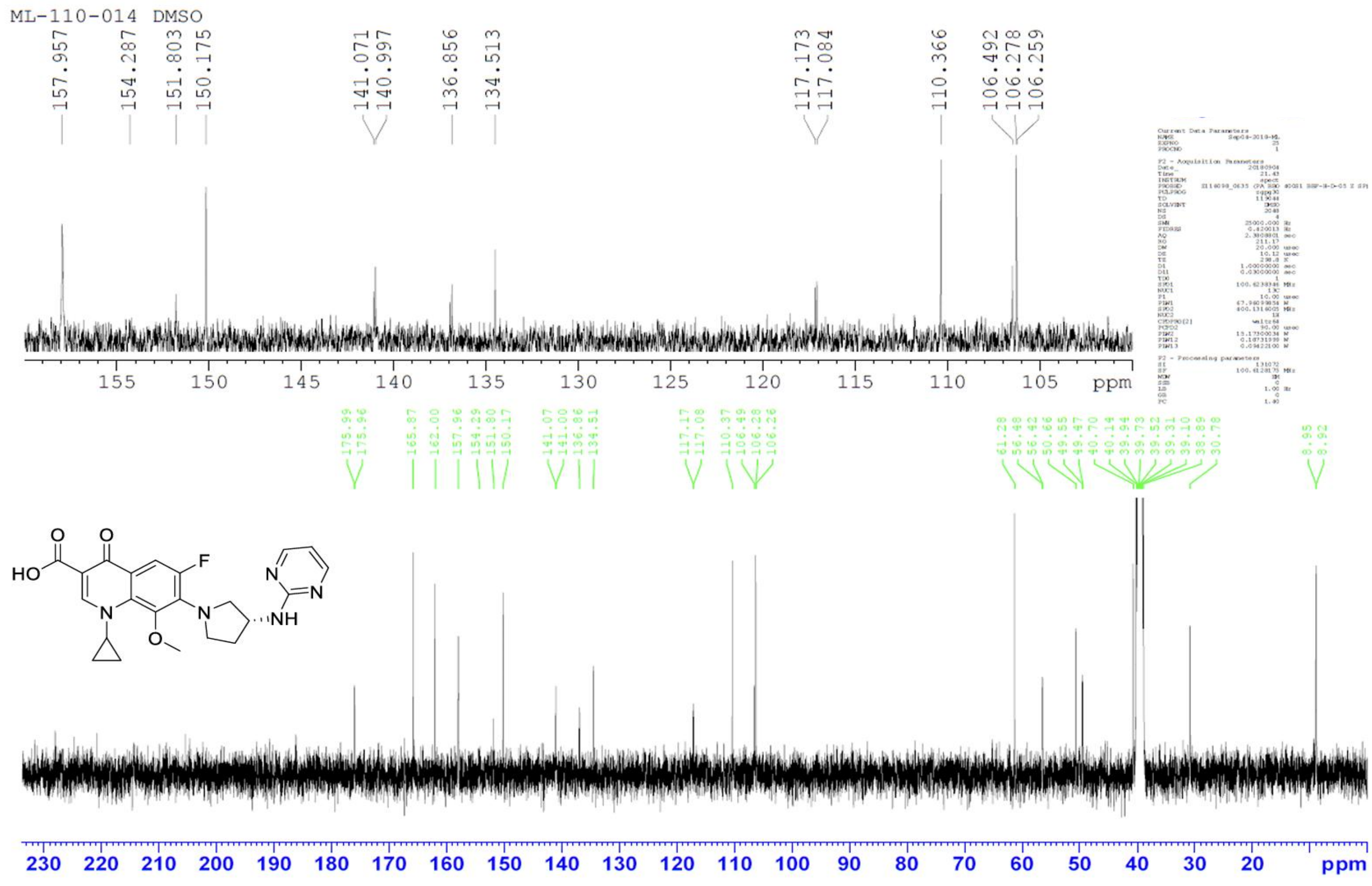

2.3 LC Traces of Lead Compounds

ML-77-005 (8)

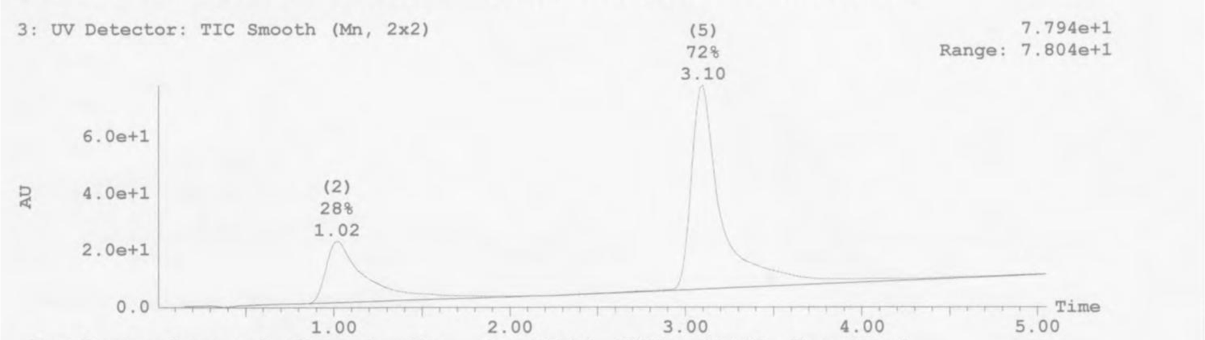

Peak Number	Compound	Time	AreaAbs	Area %Total	Width	Height	Mass Found
2		1.02	6e+006	27.79	1	2e+007	
5		3.10	2e+007	72.21	3	7e+007	

KSN-L22 (46)

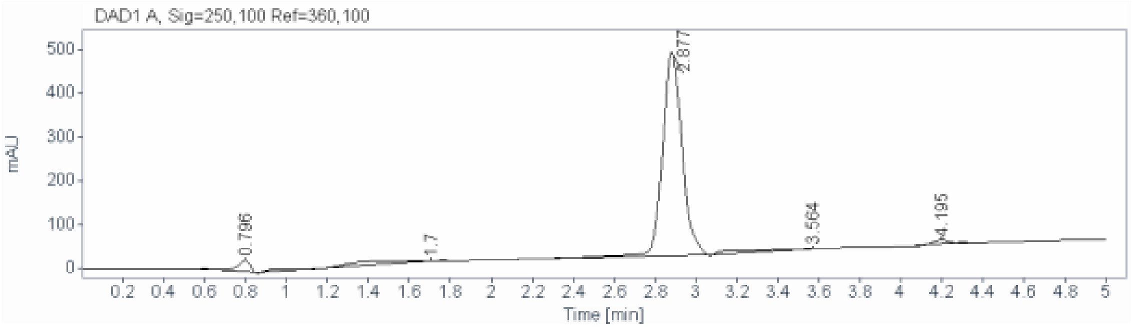

BL-7 (50)

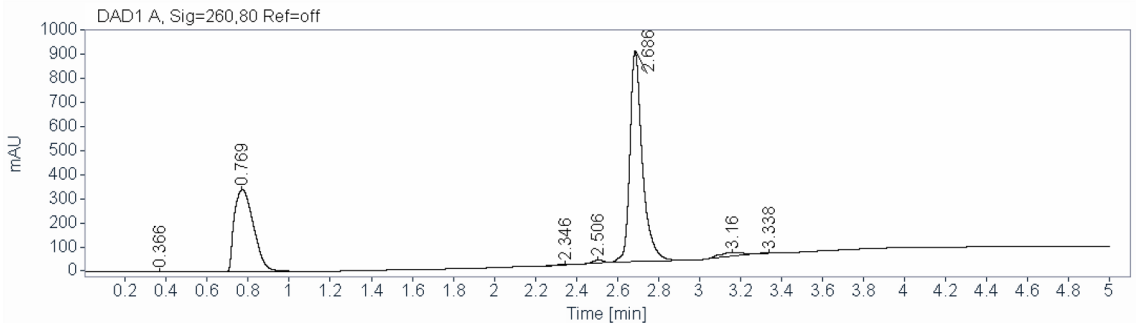

ML-110-014 (51)

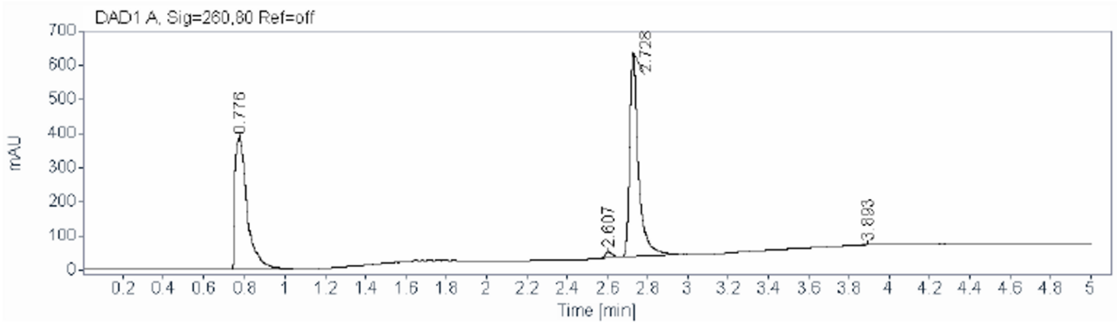

Section 3: Supporting Figures

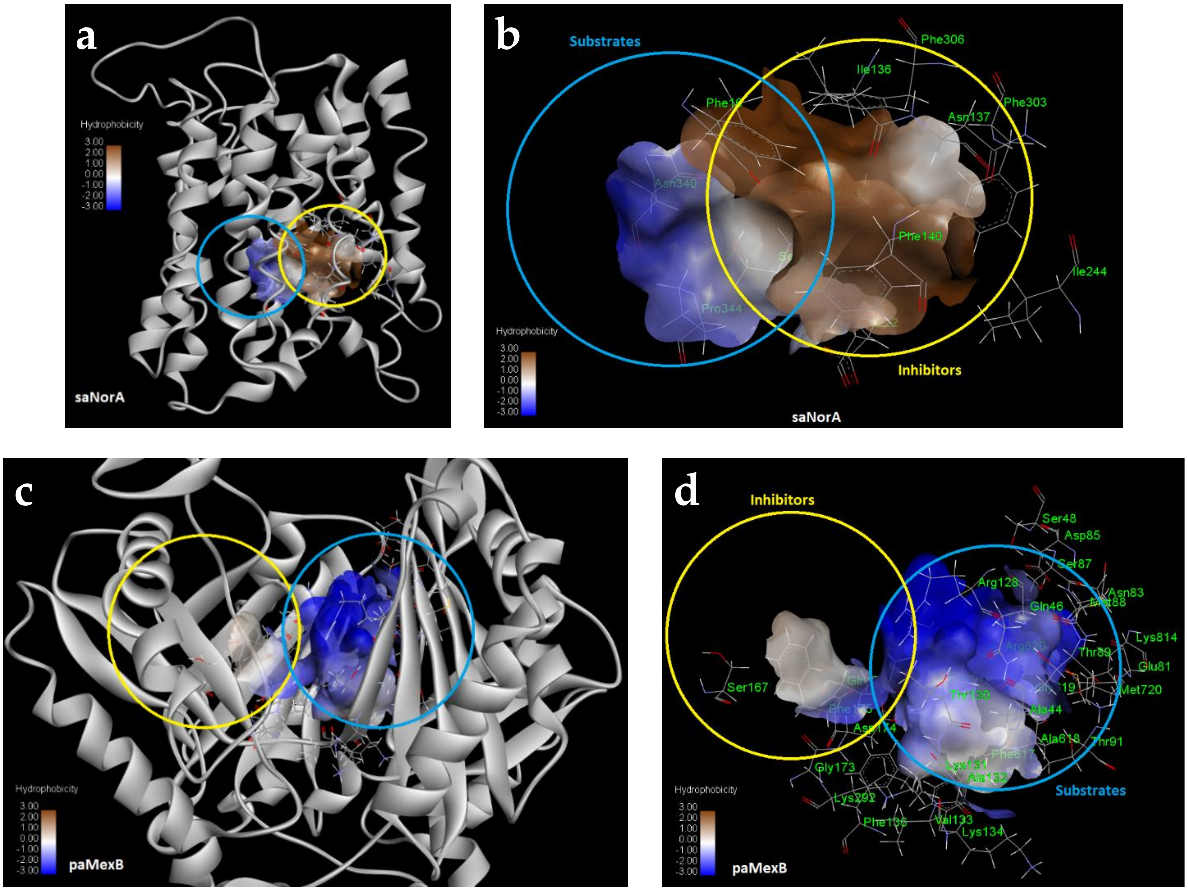

Figure S1 | a) Computational model of the NorA (PDB ID: 7LO8) efflux pump, an MFS-family transporter found in the Gram-positive bacterium *Staphylococcus aureus*. The regions of substrate (blue) and inhibitor (yellow) binding are circled. **b**) A closer view of the two NorA binding sites. **c**) Computational model of the MexB (PDB ID: 3W9J) efflux pump, an RND-family transporter found in the Gram-negative bacterium *Pseudomonas aeruginosa*. The regions of substrate (blue) and inhibitor (yellow) binding are circled. **d**) A closer view of the two MexB binding sites.

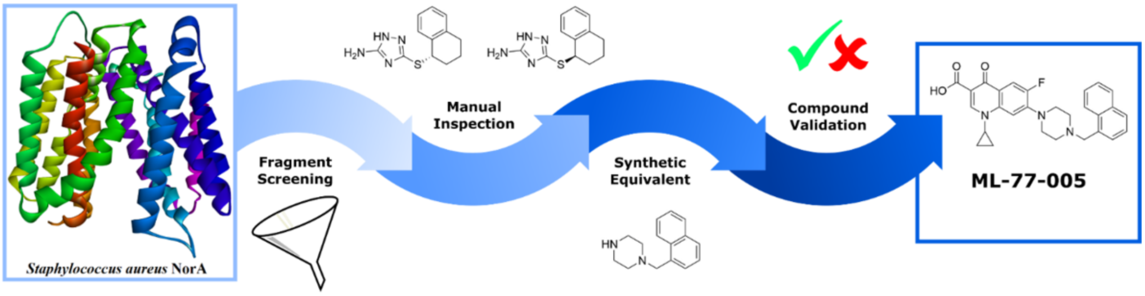

Figure S2 │ The process of fragment screening, selection, refinement and compound validation used to design **ML-77-005 (Compound 8)**. Fragments were screened against the inhibitor binding site of the *S. aureus* efflux pump NorA, then top hits were manually inspected, synthetic equivalents proposed and attached to the fluoroquinolone ciprofloxacin *in silico*. The resulting compounds (such as Compound 8) were then docked against NorA to evaluate their binding *versus* the fragments and ciprofloxacin individually.

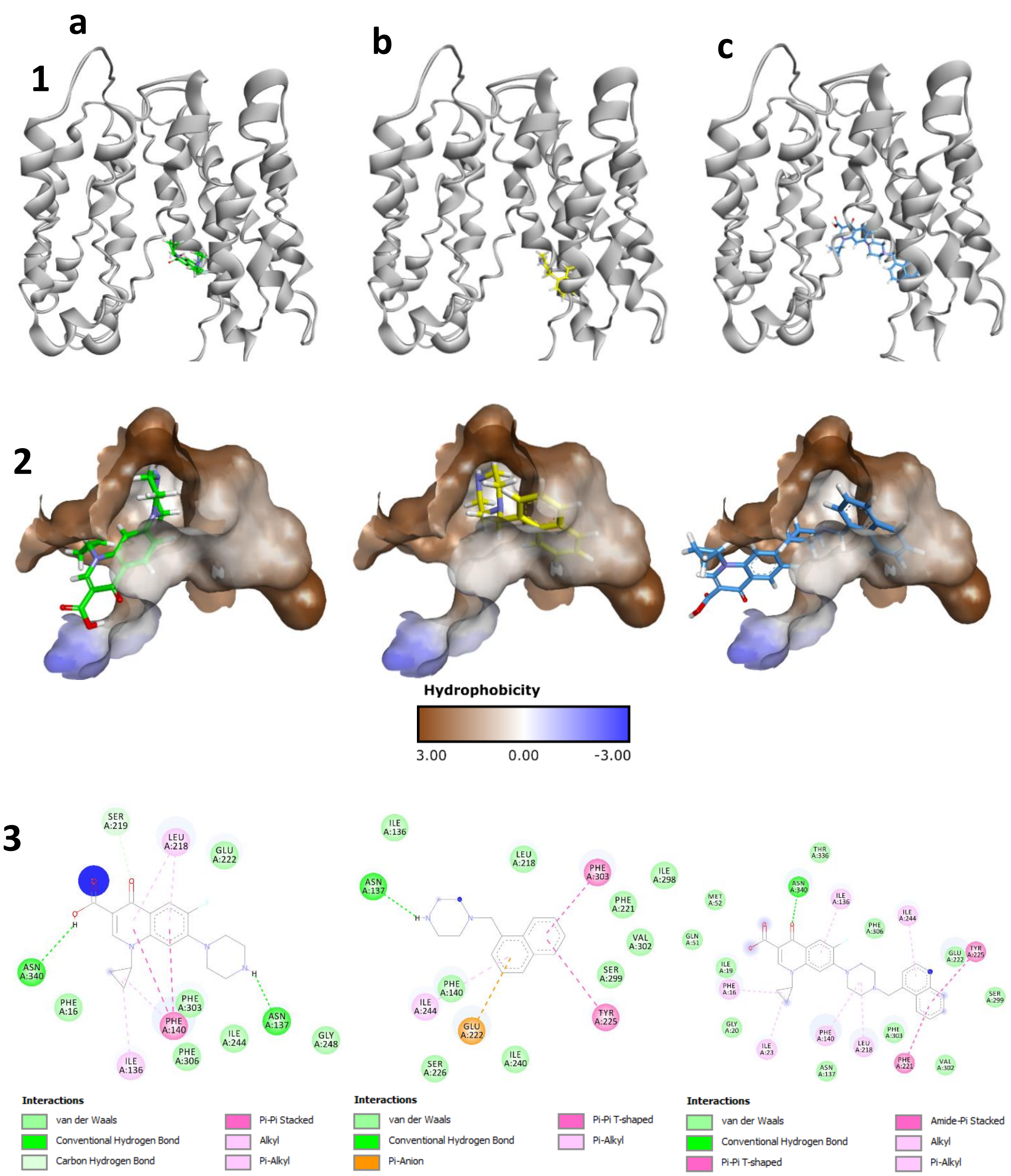

Figure S3 | a) **Compound 1**, **b**) **NMP** and **c**) **ML-77-005 (Compound 8)** bound to NorA (PDB ID: 7LO8) . **1**) Lateral, **2**) 3D and **3**) 2D views of compound binding sites are shown. Unlike compound 1 and NMP, compound 8 is predicted to contact both the hydrophilic substrate and hydrophobic inhibitor binding sites.

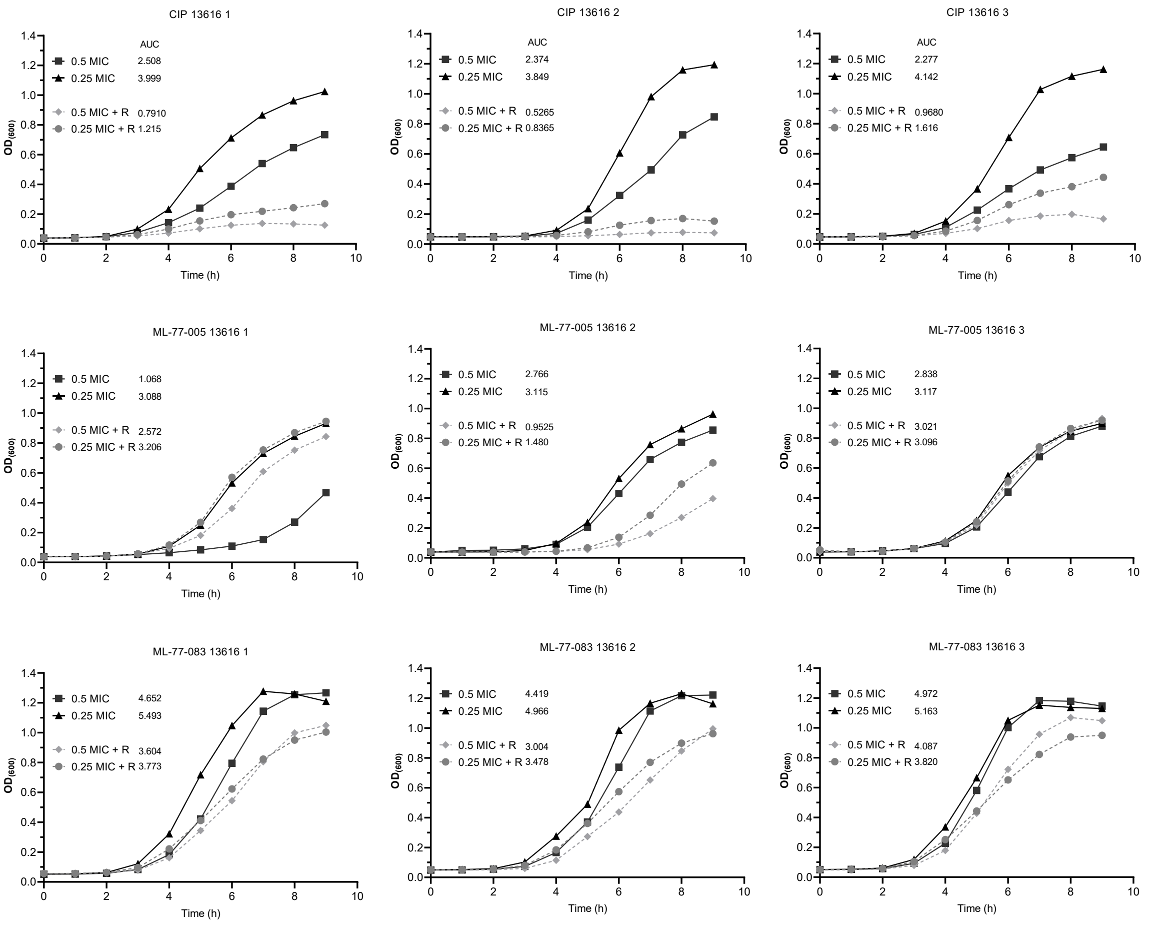

Figure S4 | Reserpine efflux inhibition assay NCTC 13616 growth curves for **Compound 1** and select first-generation ERB-fluoroquinolone compounds. Each biological replicate is shown separately for 0.5x MIC and 0.25x MIC, with and without reserpine. The AUC value for each curve is displayed next to the legends.

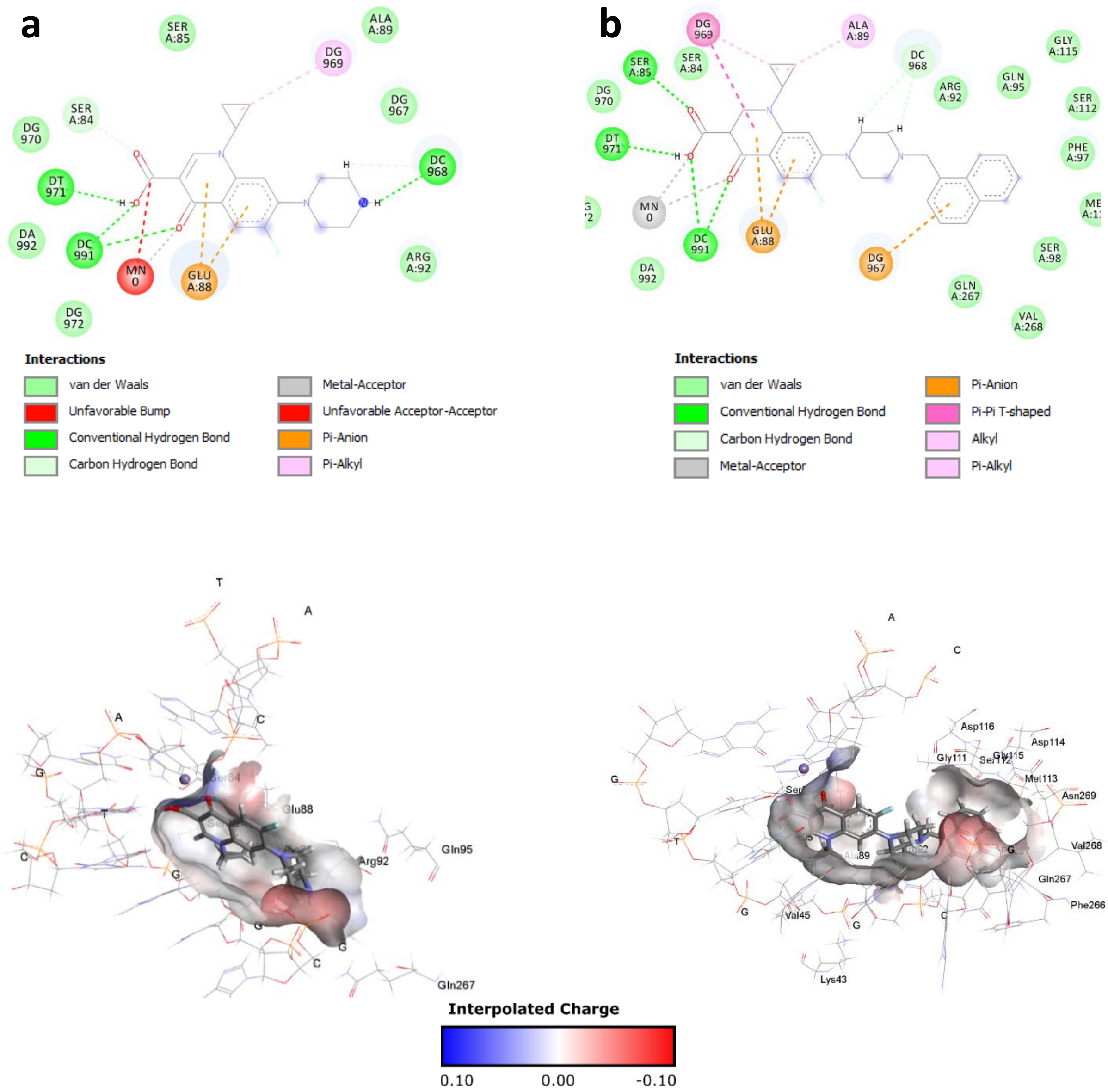

Figure S5 | 1) 2D and **2**) 3D docking images of **a**) **Compound 1** and **b**) **ML-77-005** (**Compound 8**) against the *S. aureus* DNA gyrase-DNA complex.

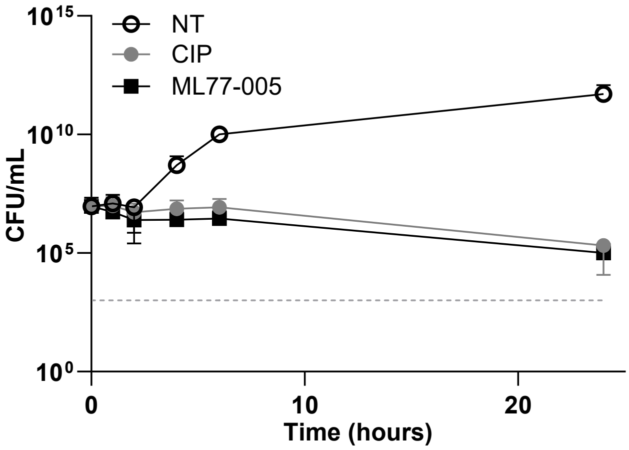

Figure S6 | Time-kill assay data for **Compound 1** and **ML-77-005** (**Compound 8)** in NCTC 13616. Time-kill assays involve incubating bacteria with a 4x MIC dose of antibiotic and monitoring population changes over at least 24 hours; a ≥ 3log10 decrease in bacterial population is indicative of bactericidal activity. Both compounds have comparable bacteriostatic profiles.

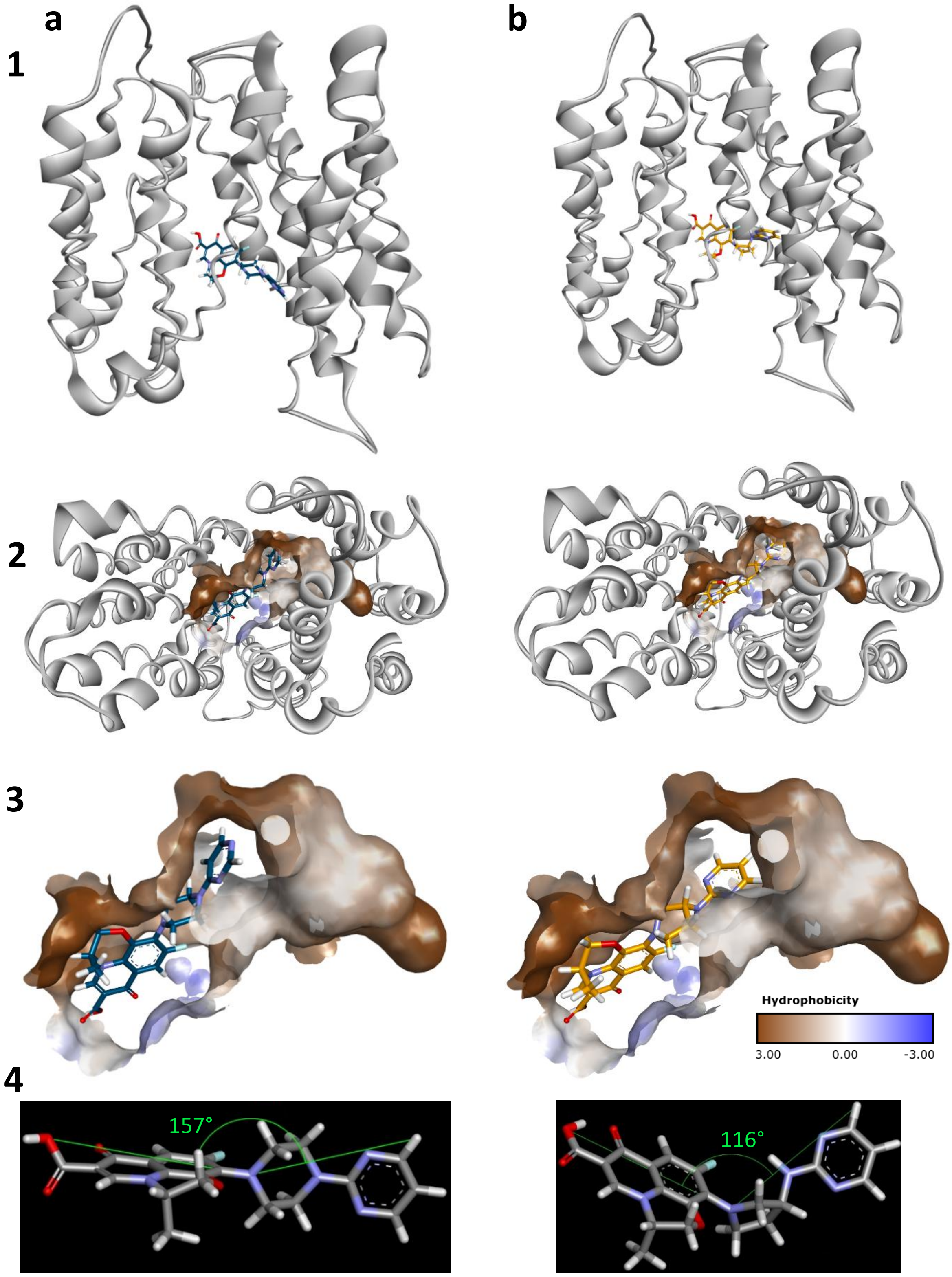

Figure S7 | a) **Compound 42** and **b**) **KSN-L22 (Compound 46)** bound to NorA (PDB ID: 7LO8) . **1**) Lateral and **2**) vertical 3D views of NorA are presented as well as **3**) 3D views of compound binding sites and **4**) angle of the terminal ERB building allows access to the hydrophobic pocket.

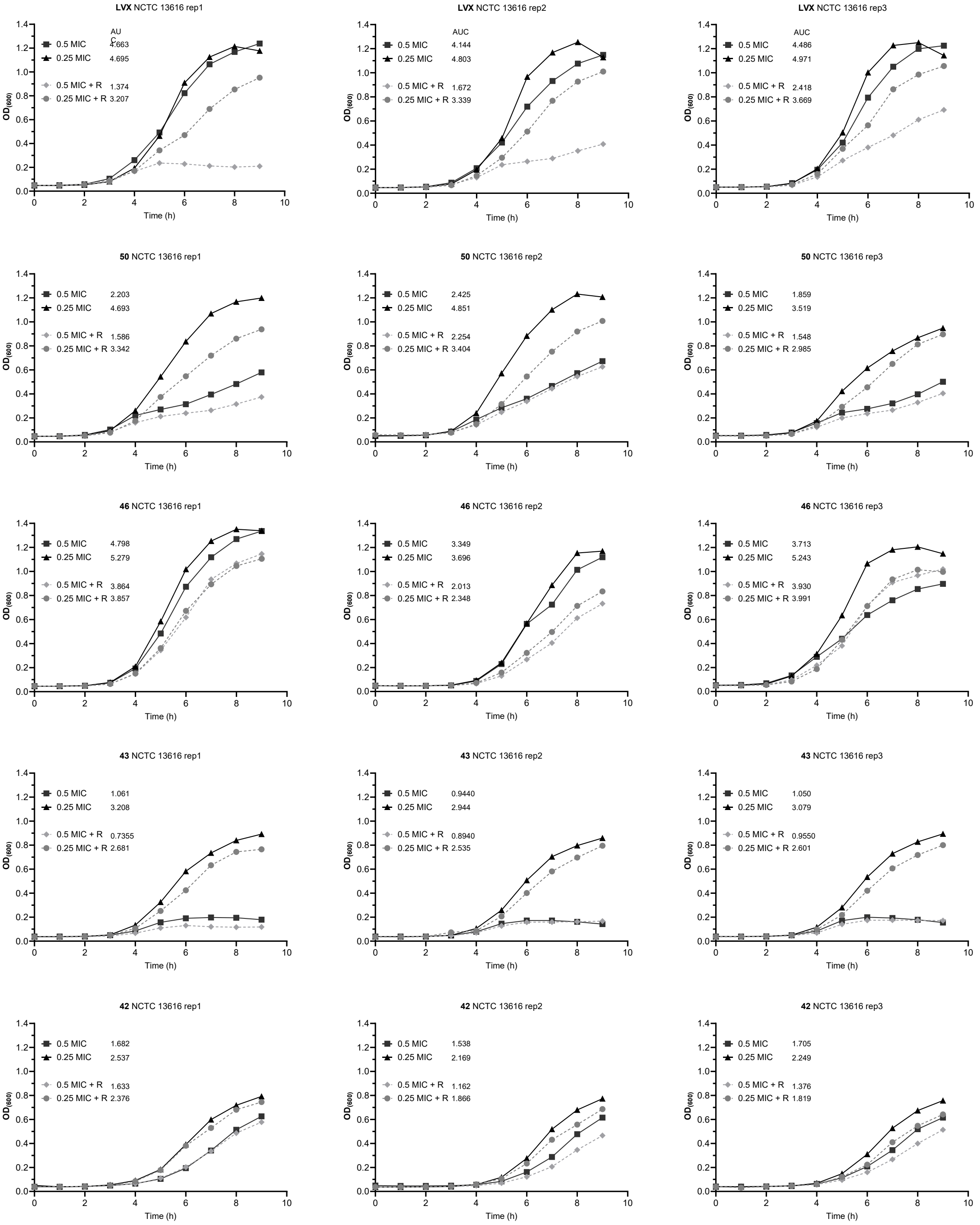

Figure S8a | Reserpine efflux inhibition assay NCTC 13616 growth curves for **Compound 7** and select second-generation ERB-fluoroquinolone compounds. Each biological replicate is shown separately for 0.5x MIC and 0.25x MIC, with and without reserpine. The AUC value for each curve is displayed next to the legends.

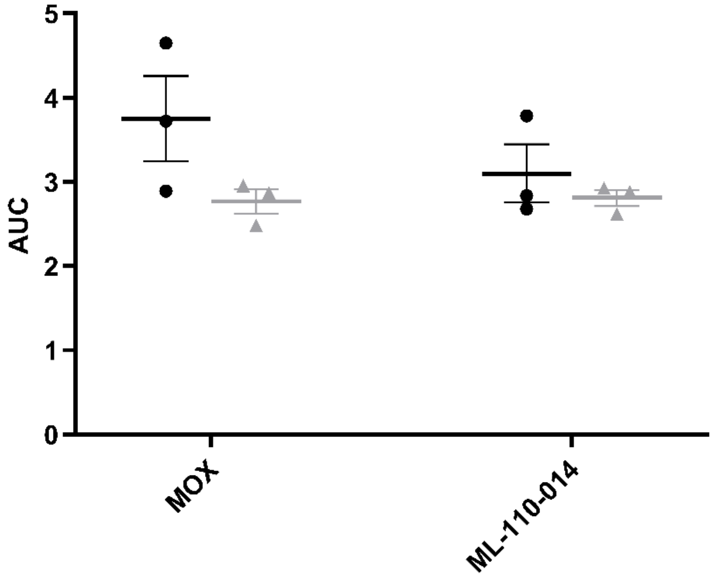

Figure S8b | Reserpine assay data for **moxifloxacin** (MOX) and **ML-110-014 (Compound 51)** in NorA overexpressing *S. aureus* strain NCTC 13616. Each point represents a biological replicate, with the bar displaying the mean AUC and SEM displayed by the error bars.

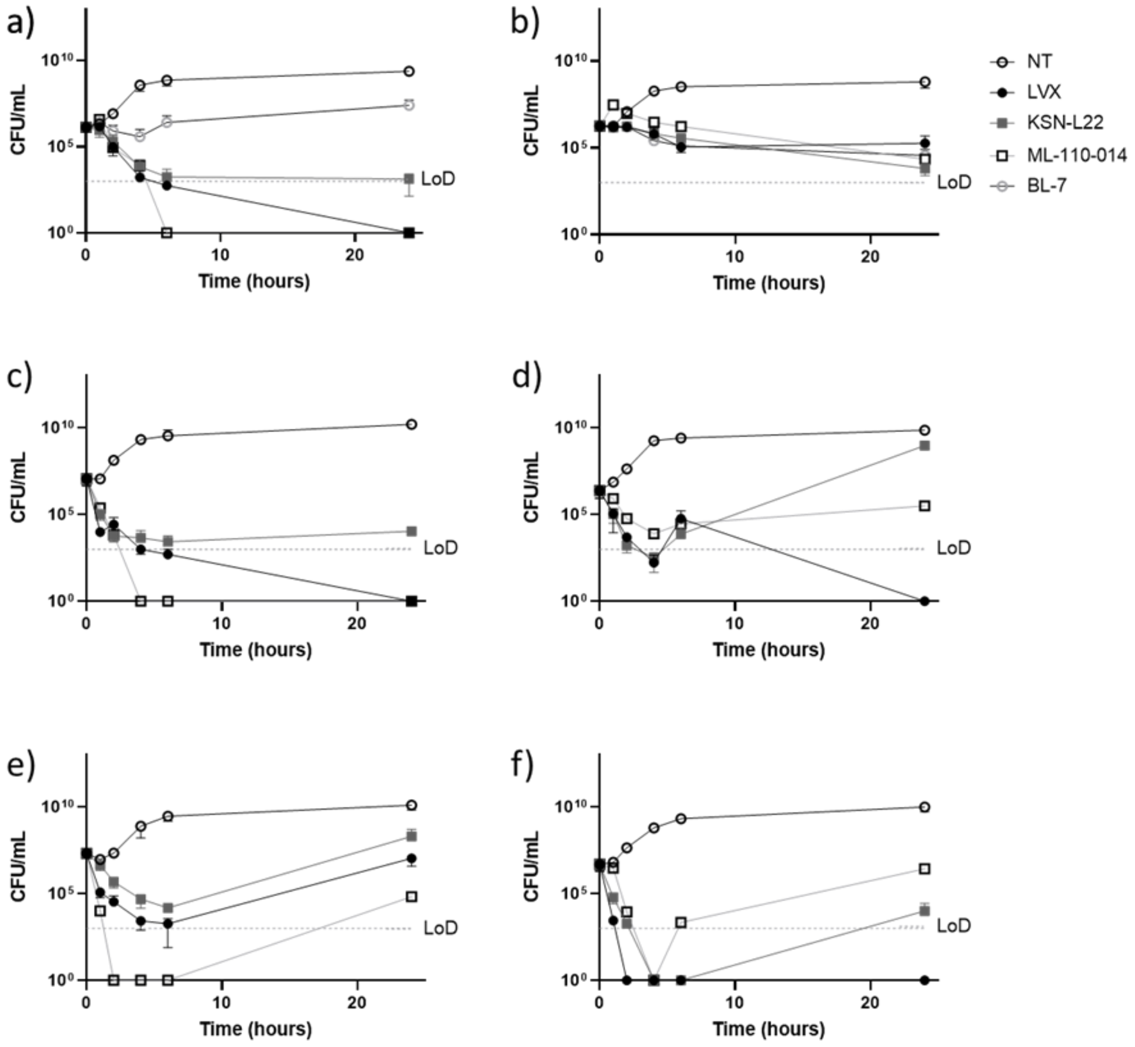

Figure S9 | Time-kill assay data for **Compound 7**, **KSN-L22 (Compound 46)**, **ML-110-014 (Compound 51)** and **BL-7 (Compound 50)** against **a**) *S. aureus* NCTC 13616, **b**) *E. faecium* NCTC 12204, **c**) *A. baumannii* AYE, **d**) *K. pneumoniae* NCTC 13368, **e**) *P. aeruginosa* PAO1 and **f**) *E. coli* NCTC 12923. Resistant populations were isolated in all 3 repeats for NCTC 13368, AYE and PAO1. After 10x passaging in the absence of selective pressure, all strains had MICs of >32 µg/ml against KSN-L22 (Compound 46). However, no SNPs were detected after whole genome sequencing.

a)

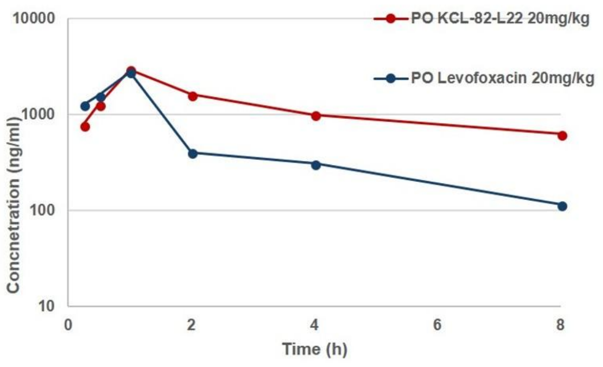

Mean TOTAL blood concentrations of KSN-82-L22 and Levofloxacin following PO administration to Male CD1 Mouse at 20 mg/kg

PK Parameter (PO)	KSN-L22	Levofloxacin
	Mean / Median (T_{max})	Mean / Median (T_{max})
Dose (mg/kg)	20.0	20.0
Dose (µmol/kg)	47.0	55.3
C_0 / C_{max} (ng/mL)	2965	2947
C_0 / C_{max} (nM)	4007	8156
T_{max} (h)	1.00	0.50
$t_{1/2}$ (h)	5.1	5.7
MRT (h)	-	-
Vdss (L/kg)	-	-
Blood CL (mL/min/kg)	-	-
CL_F (mL/min/kg)	26.4	65.4
Liver Blood Flow (%)	-	-
AUC_{inf} (ng.hr/mL)	13203	5388
AUC_{inf} (nM.hr)	31036	14909
AUC_{0-t} (ng.hr/mL)	8231	4264
AUC_{0-t} (nM.hr)	19347	11800
Fraction Absorbed		
C_{last} (ng/mL)	628	115
Bioavailability (%) Using AUC_{inf}	49.3%	40.7%
Bioavailability (%) Using AUC_{0-t}	32.0%	34.5%

b)

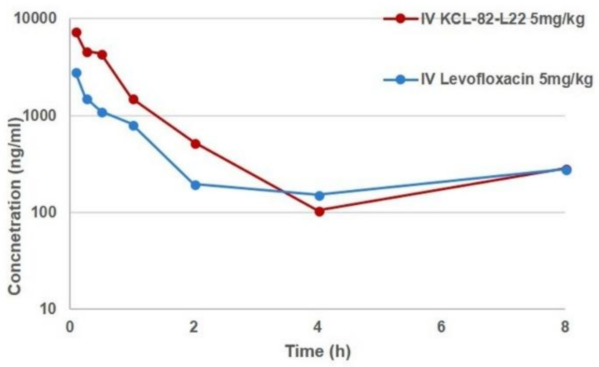

Mean TOTAL blood concentrations of KSN-82-L22 and Levofloxacin following administration to Male CD1 Mouse at 5 mg/kg

PK Parameter (iv)	KSN-L-22	Levofloxacin
	Composite Mean	Composite Mean
Dose (mg/kg)	5.0	5.0
Dose (µmol/kg)	11.8	13.8
C_0 / C_{max} (ng/mL)	9389	3890
C_0 / C_{max} (nM)	22071	10764
T_{max} (h)	-	-
$t_{1/2}$ (h)	0.6	0.5
MRT (h)	1.8	3.0
Vdss (L/kg)	1.4	4.5
Blood CL (mL/min/kg)	12.5	25.2
CL_F (mL/min/kg)	-	-
Liver Blood Flow (%)	10.4%	21.0%
AUC_{inf} (ng.hr/mL)	6691	3313
AUC_{inf} (nM.hr)	15728	9169
AUC_{0-t} (ng.hr/mL)	6425	3091
AUC_{0-t} (nM.hr)	15103	8553
Fraction Absorbed	-	-
C_{last} (ng/mL)	286	281.6
Bioavailability (%) Using AUC_{inf}	-	-
Bioavailability (%) Using AUC_{0-t}	-	-

Figure S10 | *In vivo* pharmacokinetics of **a**) oral **KSN-L22 (Compound 46)** *versus* **Compound 7** and **b**) iv **KSN-L22 (Compound 46)** *versus* **Compound 7**.

a)

Animal	$t_{1/2}$ (h)	C_0 (ng/mL)	AUClast (h*ng/mL)	AUCInf (h*ng/mL)	AUC Extr (%)	MRT (h)	Vss (L/kg)	CL (mL/min/kg)
**IV-Mouse**	2.023	31385	31442	33079	4.95	2.30	0.35	2.519

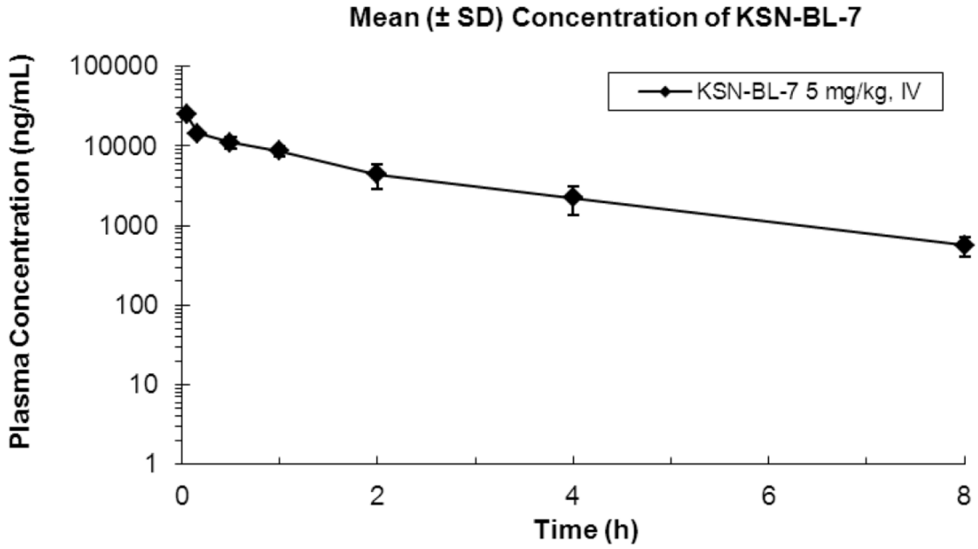

b)

Animal	T_{max} (h)	$t_{1/2}$ (h)	C_{max} (ng/mL)	AUClast (h*ng/mL)	AUCInf (h*ng/mL)	AUC/D (h*kg*ng/ml/mg)	AUC Extr (%)	MRT (h)	F (%)
**PO-Mouse**	0.500	1.790	14542	104349	104367	5218.4	0.02	4.54	82.97

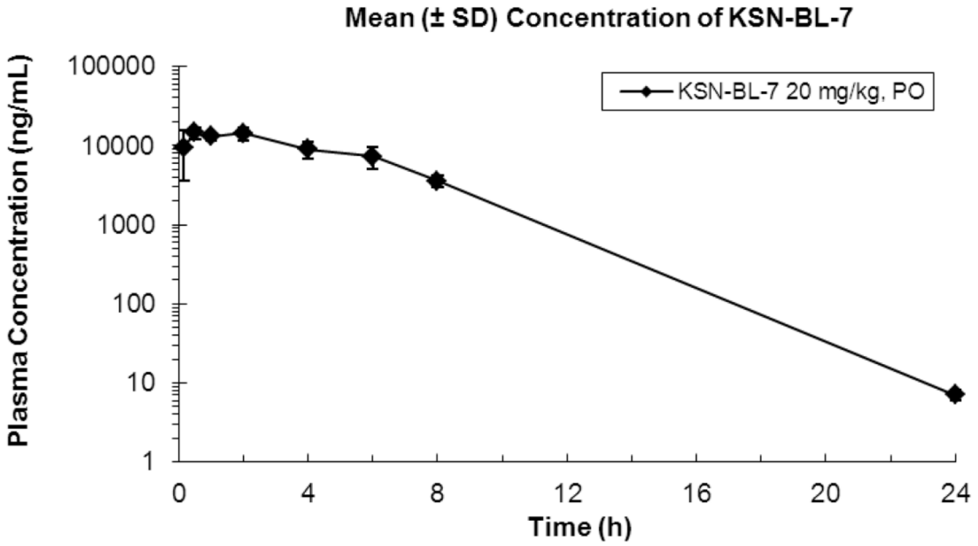

Figure S11 | *In vivo* pharmacokinetics of **a**) oral **BL-7 (Compound 50)** **b**) iv **BL-7 (Compound 50)** profile.

Section 4: Supporting Tables

Table S1: Structures and binding energies of four top-ranked ERB fragments identified by the *in silico* screening against the NorA efflux pump and their synthetic equivalent **NMP**.

Compound		NorA	
**Code**	**Structure**	**ChemScore**	**ΔG (kJ/mol)**
**Fragment 1**	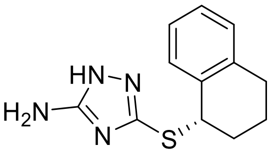	24.20	-104.5
**Fragment 2**	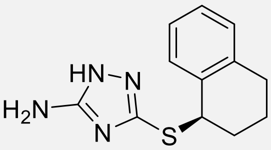	24.12	-101.8
**Fragment 3**	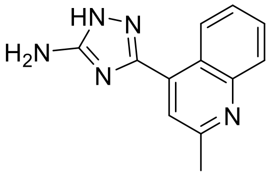	25.76	-110.3
**Fragment 4**	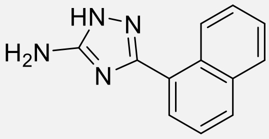	26.72	-114.2
**NMP (synthetic equivalent)**	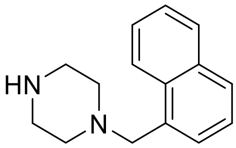	34.26	-144.9

Table S2: **ML-77-005 (Compound 8)** improves upon the combination of **Compound 1** and **NMP** in ciprofloxacin-resistant strains of *S. aureus*. **NMP** was used in combination with **Compound 1** at 50 µg/mL (0.22 mM) and 100 µg/mL (0.44 mM) in line with previous work in *E. coli* by Bohnert and co-workers[29]. At MIC concentration in strains NCTC 13616 and NCTC 13277 (64 µg/mL, 0.19 mM), molar ratios of **NMP** to **Compound 1** were 1.2 and 2.3, respectively.

		**MIC µg/mL**			
		**Compound 1**	**Compound 1 + 50 µg/mL NMP**	**Compound 1 + 100 µg/mL NMP**	**Compound 8**
***S. aureus* strains**	**ATCC 9144**	0.25	1	0.25	0.25
	**NCTC 13616**	128	64	64	2
	**NCTC 13277**	128	64	64	2

Table S3 | Gram-positive MIC data for **ML-77-005 (Compound 8)**. A decreased disparity in the **Compound 8** MICs of strains SA-1199 and SA-1199B *versus* **Compound 1** further supports the conclusion that **Compound 8** is resistant to efflux from *S. aureus*, though this assay alone is insufficient to prove this point.

**Species**	**Strain**	**Fluoroquinolone Resistance**	**Compound 1**	**Compound 8**
S. aureus	ATCC 9144	N/A	0.25	0.25
	NCTC 13616	GyrA S84L, GrlA S80F Upregulated NorA	128	2
	NCTC 13277	GyrA S84L, GrlA S80F Upregulated NorA	128	2
	SA-1199	N/A	0.25	0.125
	SA-1199B	Upregulated NorA	8	0.25
	SA215	GyrA S84L, GrlA S80F	64-128	2
	SA282	N/A	1	0.5
	SA238	GyrA S84L, GrlA S80F	32	2
	SA454	GyrA S84L, GrlA S80F	16	2
	SA105	GyrA S84L, GrlA S80F	32-64	2
	SA275	GyrA S84V, GrlA S80F	128	2
	SA388	GyrA S84L, GrlA S80F	64	2
	SA046	GyrA S84L, GrlA S80F	64	2
	SA236	GyrA S84L, GrlA S80F	128	2
	SA318	GyrA S84L, GrlA S80F	32-64	2
S. epidermidis	SE378	N/A	0.5-1	0.25-2
S. haemolyticus	SH324	Unknown	16	4
E. faecalis	NCTC 775	N/A	1	2
	NCTC 12201	N/A	0.5	0.5
	EF602	Unknown	64	2
E. faecium	NCTC 12204	N/A	1	2
	EF205	Unknown	128	2

Table S4 | Investigation of the structure-activity relationship of ML-77-005 (Compound 8). Hydrophobicity, but not aromaticity, was found to be a crucial property of the aromatic ring within the ARB unit.

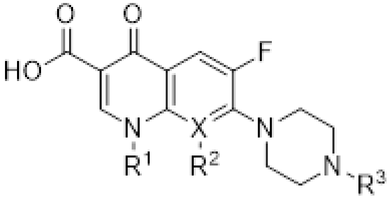

		1	2	3	7	8	20	21	22	23	24	25	26	27	28	29	30
**Structural Features**	**X**	C	C	N	C	C	C	N	C	C	C	C	C	C	C	C	C
	**R^1**	cPr	Et	Et	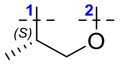	cPr	Et	Et	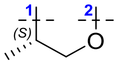	cPr	Et	cPr	Et	cPr	Et	cPr	Et
	**R^2**	H	H	-		H	H	-		H	H	H	H	H	H	H	H
	**R^3**	H			CH_3	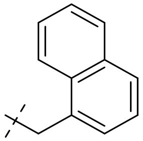				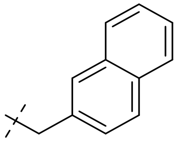		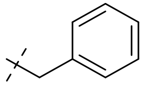		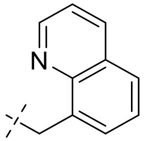		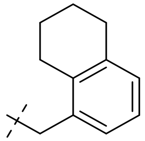	
***S. aureus* Strains**	**ATCC 9144**	0.25	2	2	0.125	0.25	1	0.5-1	0.125-1	0.125	0.25	0.125-0.25	0.25	0.5	1	0.25-1	1
	**SA-1199**	0.25	ND	ND	0.125	0.5	ND	ND	0.25	ND	ND	ND	ND	0.125	ND	0.5	0.25
	**SA-1199B**	8	ND	ND	1	0.5	ND	ND	0.5	ND	ND	ND	ND	0.25	ND	0.5	0.5
	**NCTC 13616**	128	>128	>128	16	2	2	2-8	4	2	4	16-32	16	64	128	1	2
	**NCTC 13277**	128	>128	128	16	2	2	2-4	2-4	2	4	32	16-32	128	128	1-2	2

Table S4 (continued) | Investigation of the structure-activity relationship of **Compound 8**. Hydrophobicity, but not aromaticity, was found to be a crucial property of the aromatic ring within the ARB unit.

		31	32	33	34	35	36	37	38	39	40
Structural Features	X	C	C	C	C	C	C	C	C	C	C
	R^1	cPr	Et	cPr	Et	cPr	Et	cPr	Et	cPr	Et
	R^2	H	H	H	H	H	H	H	H	H	H
	R^3	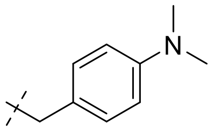		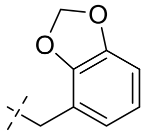		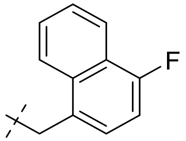		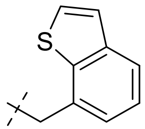		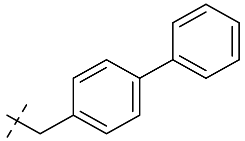	
S. aureus Strains	ATCC 9144	0.06-0.125	0.125-0.25	0.25	0.5	4	8	<0.125	0.25	1	2
	SA-1199	0.125	ND	0.125	ND	ND	ND	ND	ND	ND	ND
	SA-1199B	0.25	ND	0.25	ND	ND	ND	ND	ND	ND	ND
	NCTC 13616	8-16	16-32	64	>32	4	8	2	2	2	2
	NCTC 13277	16-32	>32	64	>32	8	8	2	2	2	4

Table S5 | Serial passaging of **ML-77-005 (Compound 8)** against *S. aureus* strains ATCC 9144 and NCTC 13616. No mutations were observed in NorA in either strain, in line with predictions.

<table>
<tr><th>Species</th><th>Strain</th><th>MIC</th><th>Mutations</th></tr>
<tr><td rowspan="4">S. aureus</td><td>ATCC 9144 WT</td><td>0.5</td><td>None</td></tr>
<tr><td>ATCC 9144 4x</td><td>2</td><td>GyrA D83G
GrlA S80F
2-oxo acid dehydrogenase subunit E2 D33E
Promoter of pepT_2 (encodes Tripeptidase T)
YpdA family putative bacillithiol disulfide reductase Q2STOP</td></tr>
<tr><td>NCTC 13616 WT</td><td>2</td><td>None</td></tr>
<tr><td>NCTC 13616 4x</td><td>4</td><td>None</td></tr>
</table>

Table S6 | Gram-negative MIC data for **ML-77-005 (Compound 8)** in the absence and presence of PMBN.

Species	Strain	Compound 8	Compound 1	Compound 8 +PMBN
K. pneumoniae	NCTC 13368	>128	0.5	1-2
	M6	32-64	0.125	0.5
A. baumannii	AYE	>128	>128	32
	ATCC 17978	4	0.25	0.125
P. aeruginosa	PAO1	>128	0.25	0.125
	NCTC 13437	128	64	32

Table S7: ADMETLab 3.0-predicted physicochemical properties of representative fluoroquinolone core scaffolds considered for ERB modification

Property	Ciprofloxacin Core	enoxacin/norfloxacin/ pefloxacin core	Lomefloxacin core	Levofloxacin core	Sparfloxacin core	Gatifloxacin/balofloxacin core	Tosufloxacin core
Molecular Weight (MW)	265.06	253.06	271.05	281.05	298.06	295.07	338.03
Volume	241.419	232.679	238.747	250.209	258.483	267.505	291.233
Density	1.098	1.088	1.135	1.123	1.153	1.103	1.161
nHA	4.0	4.0	4.0	5.0	5.0	5.0	5.0
nHD	1.0	1.0	1.0	1.0	3.0	1.0	1.0
nRot	2.0	2.0	2.0	1.0	2.0	3.0	2.0
nRing	3.0	2.0	2.0	3.0	3.0	3.0	3.0
MaxRing	10.0	10.0	10.0	12.0	10.0	10.0	10.0
nHet	6.0	6.0	7.0	7.0	8.0	7.0	9.0
fChar	0.0	0.0	0.0	0.0	0.0	0.0	0.0
nRig	16.0	13.0	13.0	17.0	16.0	16.0	19.0
Flexibility	0.125	0.154	0.154	0.059	0.125	0.188	0.105
Stereo Centers	0.0	0.0	0.0	1.0	0.0	0.0	0.0
TPSA	59.3	59.3	59.3	68.53	85.32	68.53	72.19
logS	-3.426	-3.955	-3.901	-3.087	-3.071	-3.113	-6.088
logP	1.831	1.275	1.613	1.358	1.551	1.771	2.015
logD7.4	1.814	1.408	1.482	1.371	1.593	1.751	1.616
pka (Acid)	2.969	2.926	2.697	2.639	4.485	2.945	1.749
pka (Base)	2.185	1.679	1.112	1.572	2.26	2.43	-0.155

Melting point	224.974	278.282	277.038	226.192	210.49	202.88	327.563
Boiling point	227.357	256.447	246.971	253.618	258.722	236.77	281.447

Table S8: Swiss-ADME-predicted physicochemical properties of representative fluoroquinolone core scaffolds considered for ERB modification

Molecule	MW	#Rotatable bonds	MR	TPSA	Consensus Log P	ESOL Log S	ESOL Solubility (mg/ml)	Silicos-IT Solubility (mol/l)	Brenk #alerts
**Ciprofloxacin Core**	265.21	2	63.85	59.3	2.25	-2.89	3.38e-01	4.83e-04	0
**enoxacin/ norfloxacin/ pefloxacin core**	253.2	2	61.15	59.3	2.11	-2.87	3.39e-01	3.08e-04	0
**Lomefloxacin core**	271.19	2	61.11	59.3	2.39	-2.9	3.41e-01	1.63e-04	1
**Levofloxacin core**	281.21	1	65.53	68.53	2.04	-3.19	1.84e-01	6.92e-04	0
**Sparfloxacin core**	298.22	2	68.21	85.32	2.21	-3.27	1.60e-01	5.96e-04	2
**Gatifloxacin/ balofloxacin core**	295.24	3	70.34	68.53	2.42	-3.53	8.67e-02	3.68e-04	0
**Tosufloxacin core**	338.21	2	74.13	72.19	3.16	-4.43	1.26e-02	4.10e-06	1

Table S9 | Compounds made as part of **KSN-L22 (Compound 46)** SAR. Compounds **BL-7 (Compound 50)** and **ML-110-014 (Compound 51)** were identified as promising leads. General structure for compounds synthesised shown top right. Unit µg/mL.

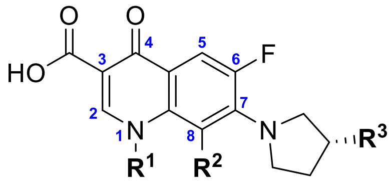

		45	46	51	48	47	52	50
Structural Features	R^1	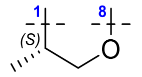	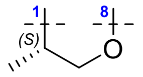	cPr	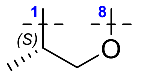	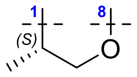	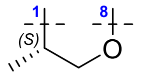	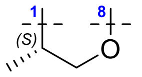
	R^2			OMe				
	R^3	NH_2	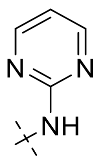	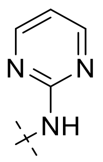	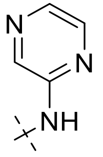	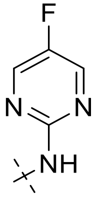	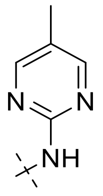	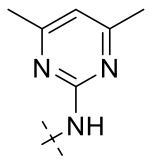
S. aureus **Strains**	**ATCC 9144**	0.25	≤0.03	≤0.03	≤0.125	≤0.125	0.03	≤0.004
	**SA-1199**	ND	0.008	0.004	0.03	ND	0.03	0.016
	**SA-1199B**	ND	0.016	0.004	0.125	ND	0.06	0.016
	**NCTC 13616**	32	0.5	0.125	1	0.5	1	0.125-0.25
	**NCTC 13277**	32	1	≤0.125-0.25	1	1	2	0.25-0.5

Table S10. Molecular dynamics simulation systems and parameters used for saNorA simulations.

**Simulation**	**Duration (300ns x 40)**	**Forcefield**	**Membrane Composition**	**MDengine[58]**	**Ligands Forcefield**
saNorA-Apo	12μs	CHARMM36m	DPPC	ACEMD	N/A
saNorA-Levofloxacin **(7)**	12μs	CHARMM36m	DPPC	ACEMD	CGENFF
saNorA-KSN-L22 **(46)**	12μs	CHARMM36m	DPPC	ACEMD	CGENFF

Table S11A | Extended spectrum MIC of **BL-7 (Compound 50)** and **KSN-L22 (Compound 46)** against *Streptococcus* clinical isolates.

Species / Strain	MIC (µg/mL) – Compound 50	MIC (µg/mL) – Compound 46
S. pyogenes 82434	≤0.03	0.06
S. pyogenes 82896	≤0.03	0.06
S. pyogenes 84285	≤0.03	0.06
S. pyogenes 81017	0.06	0.125
S. pyogenes 83581	≤0.03	0.06-0.125
S. pyogenes 13738	0.125	0.125
S. pyogenes 13737	0.125	0.125
S. pyogenes 13736	≤0.03	0.06
S. pyogenes 8198	≤0.03	≤0.03
S. pyogenes 12048	≤0.03	≤0.03-0.125
S. pyogenes 86519	0.06	0.125
S. pyogenes 80661	≤0.03	0.06-0.125
S. agalactiae 8181	0.06	0.125
S. agalactiae 8183	0.125	0.125
S. agalactiae 83190	0.06	0.125
S. agalactiae 84115	0.125	0.125-0.5
S. agalactiae 83740	0.06	0.125
S. agalactiae 10182	0.06	0.125
S. agalactiae 10069	≤0.03	≤0.03
S. agalactiae 6175	0.06	0.125
S. agalactiae 8017	0.25	0.5
S. agalactiae 11080	0.125	0.25
S. dysgalactiae 10238	≤0.03	0.06-0.25
S. dysgalactiae 84557	≤0.03	≤0.03-0.125
S. dysgalactiae 84143	0.06	0.06-0.125
S. dysgalactiae 13762	≤0.03	0.06

S. dysgalactiae 13759	≤0.03	0.06
S. dysgalactiae 84574	0.06	0.06-0.125
S. dysgalactiae 81553	0.125	0.125
S. dysgalactiae 81251	≤0.03	0.125

Table S11B | Extended spectrum MIC of **BL-7 (Compound 50)** against Gram-positive clinical isolates (MDR = Multi-drug resistant, PRSP = Penicillin-Resistant *Streptococcus pneumoniae*, ERY-R = erythromycin resistant, MRSA = methicillin resistant *Staphylococcus aureus*, VRSA = vancomycin resistant *Staphylococcus aureus*, MRSA DAP-NS = methicillin resistant *Staphylococcus aureus* not susceptible to Daptomycin, MRSA VISA = methicillin resistant *Staphylococcus aureus* vancomycin intermediate *Staphylococcus aureus*, MSSA VISA = methicillin sensitive *Staphylococcus aureus* vancomycin intermediate *Staphylococcus aureus*.

**Species / Strain**	**MIC (µg/mL)**
Enterococcus faecalis (ATCC 29212)	0.25
Enterococcus faecalis, VanB (ATCC 51575)	0.25
Enterococcus faecalis, VanB (ATCC 51299)	0.125
Enterococcus faecalis, VanA (CCUG 47775)	0.5
Enterococcus faecalis, VanB (ATCC 700802)	0.125
Enterococcus faecium (ATCC 19434)	1
Enterococcus faecium, VanA (ATCC 700221)	0.5
Enterococcus faecium, VanA (BAA-2320)	1
Enterococcus faecium, VanB (CCUG 56431)	0.25
Enterococcus faecium, VanB (CCUG 56858)	0.125
Enterococcus faecium, VanD (CCUG 58354)	0.25
Enterococcus faecium, VanA (TUH44-29; CCUG 59167)	1
Enterococcus gallinarum, VanC (ATCC 49608)	0.25
Streptococcus agalactiae (ATCC 12386)	0.25
Streptococcus oralis (ATCC 9811)	0.25
Streptococcus pneumoniae (ATCC 6301)	0.125
Streptococcus pneumoniae (ATCC 700675)	0.06

Streptococcus pneumoniae (ATCC 49619)	0.125
Streptococcus pneumoniae, MDR (ATCC 51916)	0.125
Streptococcus pneumoniae, PRSP (ATCC 700677)	0.06
Streptococcus pneumoniae, ST 23F (ATCC 700669)	0.06
Streptococcus pneumoniae, MDR (ATCC 700673)	0.06
Streptococcus pneumoniae, ERY-R (ATCC 700676)	0.125
Streptococcus pyogenes (ATCC 14289)	0.125
Streptococcus pyogenes (ATCC 19615)	0.125
Staphylococcus aureus (ATCC 6538P)	0.03
Staphylococcus aureus, MRSA (ATCC 33592)	0.03
Staphylococcus aureus (ATCC 33594)	0.03
Staphylococcus aureus (ATCC 27660)	0.03
Staphylococcus aureus USA400 MRSA (MW2)	0.03
Staphylococcus aureus MRSA VISA (Mu50)	2
Staphylococcus aureus USA300 MRSA (TCH1516)	0.03
Staphylococcus aureus (ATCC 13709)	0.03
Staphylococcus aureus USA300 MRSA (FPR3757)	0.5
Staphylococcus aureus (ATCC 49230)	0.03
Staphylococcus aureus, MRSA (R136)	0.5
Staphylococcus aureus (ATCC 10390)	0.03
Staphylococcus aureus (ATCC 29213)	0.03
Staphylococcus aureus (ATCC 29213) + 50% serum	1
Staphylococcus aureus MRSA DAP-NS (ECL 2963621)	0.25
Staphylococcus aureus VRSA (ECL 2963646)	0.5
Staphylococcus aureus MRSA DAP-NS (ECL 2963666)	1
Staphylococcus aureus MRSA DAP-NS (ECL 2963667)	1
Staphylococcus aureus MRSA DAP-NS (ECL 2963743)	0.5
Staphylococcus aureus MRSA (COL; NRS100)	0.03
Staphylococcus epidermidis (NRS101)	0.03
Staphylococcus aureus MRSA Linezolid-R (NRS119)	1

Staphylococcus aureus VISA (NRS12)	0.06
Staphylococcus aureus USA400 MRSA (NRS123)	0.03
Staphylococcus aureus LZD-NS MRSA (NRS127)	4
Staphylococcus aureus (NRS157)	0.03
Staphylococcus aureus VISA MRSA (NRS17)	1
Staphylococcus aureus USA600 MRSA GISA (NRS22)	4
Staphylococcus aureus TGC-NS MRSA (NRS269)	2
Staphylococcus aureus Linezolid-R MRSA (NRS271)	0.5
Staphylococcus aureus VISA (NRS3)	0.5
Staphylococcus aureus USA100 MRSA (NRS382)	0.5
Staphylococcus aureus USA200 MRSA (NRS383)	1
Staphylococcus aureus USA300 MRSA (NRS384)	0.03
Staphylococcus aureus USA500 MRSA (NRS385)	0.5
Staphylococcus aureus USA700 MRSA (NRS386)	0.25
Staphylococcus aureus USA800 MRSA (NRS387)	0.03
Staphylococcus aureus VISA DAP-NS MRSA (NRS402)	1
Staphylococcus aureus USA1000 MRSA (NRS483)	0.03
Staphylococcus aureus USA1100 MRSA (NRS484)	0.03
Staphylococcus aureus VISA (NRS56)	1
Staphylococcus epidermidis GIS (NRS60)	0.06
Staphylococcus epidermidis GIS (NRS7)	0.5
Staphylococcus aureus MRSA (NRS71)	1
Staphylococcus aureus (Sanger-476; NRS72)	0.03
Staphylococcus epidermidis GIS (NRS8)	1
Staphylococcus aureus VanA MRSA (VRS1)	1
Staphylococcus aureus VanA (VRS11b)	2
Staphylococcus aureus VanA MRSA (VRS2)	0.5
Staphylococcus aureus VanA MRSA (VRS3a)	0.5
Staphylococcus aureus MRSA VISA (CDC AR-BANK #0215)	16
Staphylococcus aureus MRSA VISA (CDC AR-BANK #0216)	0.5

Staphylococcus aureus MRSA VISA (CDC AR-BANK #0217)	1
Staphylococcus aureus MRSA VISA Mupirocin-R (CDC #0218)	1
Staphylococcus aureus MRSA VISA (CDC #0219)	4
Staphylococcus aureus MRSA VISA (CDC #0220)	1
Staphylococcus aureus MRSA VISA (CDC #0221)	0.5
Staphylococcus aureus MSSA VISA (CDC #0222)	0.03
Staphylococcus aureus MRSA VISA (CDC #0223)	1
Staphylococcus aureus MRSA VISA Mupirocin-R (CDC #0224)	1
Staphylococcus aureus MRSA VISA (CDC #0225)	1
Staphylococcus aureus MRSA VISA (CDC #0226)	0.03
Staphylococcus aureus MRSA VISA (CDC #0227)	1
Staphylococcus aureus MRSA VISA Mupirocin-R (CDC #0228)	2
Streptococcus intermedius (ATCC 29663)	0.03
Staphylococcus epidermidis (ATCC 12228)	0.03
Staphylococcus epidermidis MRSE (ATCC 51625)	0.03
Staphylococcus haemolyticus (ATCC 29970)	0.03
Staphylococcus saprophyticus (ATCC 15305)	0.06
Streptococcus mutans (ATCC 25175)	0.03
Streptococcus salivarius (ATCC 13419)	0.25
Streptococcus sanguinis (ATCC 10556)	0.25
Staphylococcus aureus MRSA (ATCC 33591)	0.03
Staphylococcus aureus (Smith; ATCC 19636)	0.03
Streptococcus pneumoniae, PRSP (TM532)	0.06

Table S12 | MICs (µg/mL) of **KSN-L22 (Compound 46)** in *A. baumannii* strains with transposon mutations in efflux pump components.

		**Gene**	**Compound 1**	**Compound 46**
**AB5075-UW**		-	4	4
**AB05226**	*adeA*165::T26	adeA	4	4
**AB05227**	*adeA*138::T26	adeA	2	2
**AB05228**	*adeB*150::T26	adeB	2	1
**AB05230**	*adeB*181::T26	adeB	2	1
**AB05219**	*adeR*103::T26	adeR	4	4
**AB05222**	*adeR*160::T101	adeR	2	4
**AB05215**	*adeS*132::T26	adeS	2	4
**AB05218**	*adeS*141::T26	adeS	4	4
**AB01335**	*adeG*183::T26	adeG	4	4

Table S13A | MICs of **BL-7 (Compound 50), KSN-L22 (Compound 46)** and **ML-110-014 (Compound 51)** against *E. coli* and *K. pneumoniae*.

		MIC, µg/mL				
**Species**	**Strain**	**Compound 46**	**Compound 50**	**Compound 51**	**Compound 1**	**Compound 7**
Escherichia coli	319238/UR	16	32	16	>128	8
	NCTC 10418	≤0.06	0.125	0.125	≤0.125	≤0.06
	NCTC 11954	0.125	0.25	0.125	≤0.125	≤0.06
	NCTC 12241	0.06	0.25	0.06	≤0.125	≤0.06
	NCTC 12923	0.25-0.5	1	≤0.125	≤0.125	≤0.016
	LEC006	0.125	0.5	0.25	≤0.125	≤0.06
	LEC007	0.125	0.25	0.125	≤0.125	≤0.06
	LEC077	4	8	4	0.25	0.5
	LEC081	2	8	2	0.25	0.5
Klebsiella pneumoniae	M6	1-2	2	1-2	0.125	0.125
	NCTC 13368	8	16	4	0.5	2
	TW3	1	-	0.5	≤0.125	0.125

Table S13B | *Pseudomonas aeruginosa* MICs of **KSN-L22 (Compound 46)** with PMBN.

		**MIC, µg/mL**			
**Species**	**Strain**	**Compound 46**	**Compound 46 + PMBN**	**Compound 7**	**Compound 7 + PMBN**
Pseudomonas aeruginosa	PAO1	4 - 8	-	2	0.125
	PAO1 ΔmexB	4-8	≤0.03	0.5	≤0.03
	NCTC 10662	8	≤0.03	2	≤0.125
	NCTC 12903	8	≤0.03	4	≤0.125
	NCTC 13359	4-32	0.06	4	0.25
	CAS4	8	0.06	2	≤0.125
	GH12	>32	0.5	64	4
	GH100	1-4	-	0.25	-
	CFL 004 CRCN	>32	8	128	16

Table S13C | MICs of **KSN-L22 (Compound 46)** against resistant *Neisseria gonorrhoeae* strains including fluoroquinolone resistant strains.

			**MIC, μg/mL**
**Species**	**Strain**	**Resistance**	**Compound 46**
Neisseria gonorrhoeae	ATCC 49226	PEN-I TET-I	≤0.03
	ATCC 700825	---	≤0.03
	CCUG 57595	---	≤0.03
	CCUG 57596	CIP-I PEN-I TET-R	0.125
	CCUG 57597	FEP-NS FOX-I CAZ-NS CIP-R OFX-R PEN-R TET-R	2
	CCUG 57598	FOX-R CAZ-NS CRO-NS CIP-R OFX-R PEN-R TET-R	2
	CCUG 57599	CIP-R OFX-R PEN-R TET-R	0.5
	CCUG 57600	CIP-R OFX-R PEN-R TET-R	1
	CCUG 57601	PEN-R TET-R	≤0.03
	CCUG 57602	PEN-I TET-I	0.06
	NCTC 13477		≤0.125
	NCTC 13817	PEN-R TET-I	0.5
	NCTC 13818	CIP-R OFX-R PEN-R TET-R	2
	NCTC 13819	FEP-NS CAZ-NS CIP-R OFX-R PEN-R TET-I	2
	NCTC 13820	FEP-NS CFM-NS CAZ-NS CRO-NS CIP-R OFX-R PEN-R TET-R	2
	NCTC 13821	FEP-NS CFM-NS CAZ-NS CRO-NS CIP-R PEN-I TET-I	2
	NCTC 13822	FEP-NS CFM-NS CAZ-NS CIP-R OFX-R PEN-R TET-I	2
	BAA-1833	PEN-I TET-I	≤0.03
	BAA-1838	---	≤0.03
	TCDC-NG08107	PEN-R CFM-R CPD-R CIP-R	2
	2007NG046	PEN-R CFM-R CPD-R CIP-R	4
	2008NG057	PEN-R CFM-R CPD-R CIP-R	2
	2008NG097	PEN-R CFM-R CPD-R CIP-R	2
	2009NG514	PEN-R CPD-R CIP-R	2
	2010NG122	PEN-R CFM-R CIP-R	2
	FDA-CDC AR-BANK#0165	CIP-R PEN-I TET-R	2

	FDA-CDC AR-BANK#0166	CIP-R PEN-I TET-R	2
	FDA-CDC AR-BANK#0167	CIP-S PEN-S TET-I	≤0.03
	FDA-CDC AR-BANK#0168	CIP-R PEN-I TET-R	1
	FDA-CDC AR-BANK#0169	CIP-R PEN-I TET-R	2
	FDA-CDC AR-BANK#0170	CIP-R PEN-I TET-R	2
	FDA-CDC AR-BANK#0171	CIP-R PEN-I TET-R	1
	FDA-CDC AR-BANK#0172	CIP-R PEN-R TET-R	1
	FDA-CDC AR-BANK#0173	CIP-R PEN-R TET-R	2
	FDA-CDC AR-BANK#0174	CIP-R PEN-R TET-R	2
	FDA-CDC AR-BANK#0175	CIP-S PEN-I TET-I	≤0.03
	FDA-CDC AR-BANK#0176	CIP-R PEN-R TET-R	2
	FDA-CDC AR-BANK#0177	CIP-S PEN-I TET-R	≤0.03
	FDA-CDC AR-BANK#0178	CIP-R PEN-R TET-R	2
	FDA-CDC AR-BANK#0179	CIP-S PEN-S TET-I	≤0.03
	FDA-CDC AR-BANK#0180	CIP-R PEN-I TET-R	2
	FDA-CDC AR-BANK#0181	CIP-S PEN-I TET-R	≤0.03
	FDA-CDC AR-BANK#0182	CIP-R PEN-I TET-R	2
	FDA-CDC AR-BANK#0183	CIP-R PEN-R TET-R	2
	FDA-CDC AR-BANK#0184	CIP-R PEN-R TET-R	2
	FDA-CDC AR-BANK#0185	CIP-R PEN-I TET-R	2
	FDA-CDC AR-BANK#0186	CIP-R PEN-R TET-R	1
	FDA-CDC AR-BANK#0187	CIP-R PEN-I TET-R	2
	FDA-CDC AR-BANK#0188	CIP-R PEN-I TET-R	1
	FDA-CDC AR-BANK#0189	CIP-R PEN-R TET-R	2
	FDA-CDC AR-BANK#0190	CIP-R PEN-R TET-R	2
	FDA-CDC AR-BANK#0191	CIP-R PEN-R TET-R	2
	FDA-CDC AR-BANK#0192	CIP-R PEN-R TET-R	2
	FDA-CDC AR-BANK#0193	CIP-S PEN-R TET-R	≤0.03
	FDA-CDC AR-BANK#0194	CRO-NS CIP-S PEN-I TET-R	≤0.03

	FDA-CDC AR-BANK#0195	CIP-R PEN-I TET-R	2
	FDA-CDC AR-BANK#0196	CIP-R PEN-R TET-R	2
	FDA-CDC AR-BANK#0197	CIP-R PEN-I TET-I	2
	FDA-CDC AR-BANK#0198	CIP-R PEN-R TET-R	2
	FDA-CDC AR-BANK#0199	CIP-S PEN-R TET-R	≤0.03
	FDA-CDC AR-BANK#0200	CIP-R PEN-R TET-R	2
	FDA-CDC AR-BANK#0201	CIP-R PEN-R TET-R	2
	FDA-CDC AR-BANK#0202	CIP-S PEN-S TET-I	≤0.03
	FDA-CDC AR-BANK#0203	CIP-R PEN-R TET-R	2
	FDA-CDC AR-BANK#0204	CIP-R PEN-I TET-R	2
	FDA-CDC AR-BANK#0205	CIP-R PEN-R TET-R	2
	FDA-CDC AR-BANK#0206	CIP-R PEN-R TET-R	2
	FDA-CDC AR-BANK#0207	CIP-R PEN-R TET-R	2
	FDA-CDC AR-BANK#0208	CIP-R PEN-I TET-R	2
	FDA-CDC AR-BANK#0209	CIP-R PEN-I TET-R	2
	FDA-CDC AR-BANK#0210	CIP-R PEN-I TET-I	2
	FDA-CDC AR-BANK#0211	CIP-R PEN-R TET-R	2
	FDA-CDC AR-BANK#0212	CIP-R PEN-I TET-R	2
	FDA-CDC AR-BANK#0213	CIP-R PEN-I TET-I	1
	FDA-CDC AR-BANK#0214	CIP-R PEN-R TET-R	2

Table S14 | Serial passaging data for **Compound 7, ML-110-014 (Compound 51), KSN-L22 (Compound 46)**, and **BL-7 (Compound 50)**. MICs of three *S. aureus* strains after adaptation to 4 x MIC of **Compound 51**, **Compound 46**, **Compound 7** or **Compound 50**. All strains demonstrated reduced susceptibility to the compound they were adapted to, but this did not necessarily result in cross-resistance to the other compounds.

		MIC (µg/mL)				Whole genome sequencing mutations
**Exposed to:**		**Compound 51**	**Compound 46**	**Compound 7**	**Compound 50**	
NCTC 13616	WT	0.125	0.5	8	0.125	-
	Compound 51	**0.5**	2	32	1	FocA Synonymous, 15 bp del aroC_2 chorismate synthase, 1 bp del PheT-1
	Compound 46	0.125	**0.5**	16	0.25	Nothing
	Compound 7	0.25	0.5	**>32**	0.5	ParC E84K
	Compound 50	0.25	0.5	16	**0.5**	Nothing
1199B	WT	0.008	0.015	1	0.03	-
	Compound 51	**0.015**	0.06	2	0.03	Nothing
	Compound 46	0.03	**0.125**	2	0.125	GyrA E88K, Prom HoxN Family transporter
	Compound 7	0.008	0.03	**4**	≤0.015	Nothing
	Compound 50	0.125	0.25	4	**0.25**	GyrA S84L
1199	WT	0.004	0.008	0.125	0.015	-
	Compound 51	**0.015**	0.03	0.25	≤0.015	GyrA S84L
	Compound 46	≤0.008	**0.03**	0.25	≤0.015	Nothing
	Compound 7	≤0.008	0.015	**1**	0.03	Nothing
	Compound 50	0.06	0.125	1	**0.125**	GyrA S84L, ParC I487L

Table S15 | Mutation frequencies for **Compound 7** (control), **KSN-L22 (Compound 46)**, and **BL-7 (Compound 50)**. For the NCTC 13616 2x MIC frequencies, p-values when compared to **Compound 7** (t-test) were 0.0006 and 0.0005 for **Compound 46** and **Compound 50**, respectively. These values indicate extreme statistical significance.

Species	Strain	Compound	Agar MIC	2 x MIC	4 x MIC	8 x MIC
S. aureus	ATCC 9144	Compound 7	0.12	7.08×10^{-6}	1.98×10^{-7}	$<1.5 \times 10^{-8}$
		Compound 46	0.008	7.01×10^{-6}	$<1.5 \times 10^{-8}$	$<1.5 \times 10^{-8}$
		Compound 50	0.03	$<1.5 \times 10^{-8}$	$<1.5 \times 10^{-8}$	$<1.5 \times 10^{-8}$
	NCTC 13616	Compound 7	4	1.87×10^{-5}	3.2×10^{-6}	ND
		Compound 46	0.5	1.98×10^{-6}	$<1.4 \times 10^{-7}$	ND
		Compound 50	0.5	1.58×10^{-6}	$<1.4 \times 10^{-7}$	ND
E. faecium	NCTC 12204	Compound 7	1	4.6×10^{-5}	$<9.0 \times 10^{-8}$	ND
		Compound 50	2	1.77×10^{-5}	$<9.0 \times 10^{-8}$	ND
K. pneumoniae	M6	Compound 7	0.03	1.99×10^{-6}	1.9×10^{-7}	ND
		Compound 46	0.5	3.17×10^{-6}	$\leq 4.4 \times 10^{-7}$	ND
A. baumannii	A118	Compound 7	0.06	3.35×10^{-6}	5.45×10^{-8}	$<2.0 \times 10^{-8}$
		Compound 46	0.03	3.86×10^{-6}	$<2.0 \times 10^{-8}$	$<2.0 \times 10^{-8}$
		Compound 50	0.25	1.62×10^{-6}	$<2.0 \times 10^{-8}$	$<2.0 \times 10^{-8}$
E. coli	NCTC 12241	Compound 7	0.03	3.17×10^{-7}	$<5.0 \times 10^{-8}$	$<5.0 \times 10^{-8}$
		Compound 46	0.03	1.38×10^{-6}	$<5.0 \times 10^{-8}$	$<5.0 \times 10^{-8}$
		Compound 50	0.25	3.38×10^{-6}	$<6.12 \times 10^{-8}$	$<5.0 \times 10^{-8}$
	NCTC 12923	Compound 7	0.0078	3.7×10^{-6}	$<6.2 \times 10^{-8}$	ND
		Compound 46	0.12	1.32×10^{-5}	5.8×10^{-7}	ND

Table S16: MICs of **KSN-L22 (46)** and **BL-7 (50)** in the presence of 4% serum albumin.

S. aureus strains	**Levofloxacin**		**KSN-L22 (46)**		**BL7 (50)**	
		+ 4% albumin		+ 4% albumin		+ 4% albumin
ATCC 9144	0.25	0.25	0.03	0.03-0.06	≤0.004	0.125
1199	0.25	0.25	0.03	0.03-0.06	0.016	0.06
1199b	2	2	0.03	0.06	0.016	0.125
NCTC 13616	16	16	1	1-2	0.5	2
NCTC 13277	16	16	1	1-2	1	4

Table S17 | SafetyScreen44 data results for **BL-7 (Compound 50)** performed as off-target toxicity evaluation at 1.0E-05 M concentration. Results showing an inhibition of higher than 50% represent significant effects of the test compound. Results showing an inhibition between 25% and 50% are indicative of weak to moderate effects while those showing an inhibition lower than 25% are not considered significant.

Assay #	Assay Name	Compound	Response/Readout	% Inhibition
4	A2A (h) (agonist radioligand)	Compound 50	Specific binding	82.8099
93	M2 (h) (antagonist radioligand)	Compound 50	Specific binding	62.7802
471	5-HT2A (h) (agonist radioligand)	Compound 50	Specific binding	41.495
363	acetylcholinesterase (h)	Compound 50	Enzymatic activity	34.2169
4077	PDE4D2 (h)	Compound 50	Enzymatic activity	31.8186
4186	COX2(h)	Compound 50	Enzymatic activity	25.9133
91	M1 (h) (antagonist radioligand)	Compound 50	Specific binding	25.4809
13	alpha 2A (h) (antagonist radioligand)	Compound 50	Specific binding	23.4814
52	dopamine transporter (h) (antagonist radioligand)	Compound 50	Specific binding	22.5664
28	BZD (central) (agonist radioligand)	Compound 50	Specific binding	22.3569
1971	kappa (KOP) (agonist radioligand)	Compound 50	Specific binding	20.0688
4173	COX1(h)	Compound 50	Enzymatic activity	15.0111
443	MAO-A (antagonist radioligand)	Compound 50	Specific binding	15.0018
18	beta 1 (h) (agonist radioligand)	Compound 50	Specific binding	12.491
4072	PDE3A (h)	Compound 50	Enzymatic activity	12.1599
132	5-HT1B (antagonist radioligand)	Compound 50	Specific binding	10.2234
1333	5-HT2B (h) (agonist radioligand)	Compound 50	Specific binding	9.77943
159	V1a (h) (agonist radioligand)	Compound 50	Specific binding	9.52513
469	GR (h) (agonist radioligand)	Compound 50	Specific binding	9.18835
411	5-HT3 (h) (antagonist radioligand)	Compound 50	Specific binding	7.01754
2338	alpha 1A (h) (antagonist radioligand)	Compound 50	Specific binding	6.96175
870	H1 (h) (antagonist radioligand)	Compound 50	Specific binding	5.56645
95	M3 (h) (antagonist radioligand)	Compound 50	Specific binding	3.56255
933	AR (h) (agonist radioligand)	Compound 50	Specific binding	3.16911
166	KV channel (antagonist radioligand)	Compound 50	Specific binding	3.11262
2906	Lck kinase (h)	Compound 50	Enzymatic activity	2.86546
439	5-HT transporter (h) (antagonist radioligand)	Compound 50	Specific binding	2.22772
355	norepinephrine transporter (h) (antagonist radioligand)	Compound 50	Specific binding	2.06287
114	delta (DOP) (h) (agonist radioligand)	Compound 50	Specific binding	1.76644
37	CB2 (h) (agonist radioligand)	Compound 50	Specific binding	0.987728
118	mu (MOP) (h) (agonist radioligand)	Compound 50	Specific binding	0.498849
3029	N neuronal alpha 4beta 2 (h) (agonist radioligand)	Compound 50	Specific binding	0.422093
54	ETA (h) (agonist radioligand)	Compound 50	Specific binding	0.265781
4094	Potassium Channel hERG (human)- [3H] Dofetilide	Compound 50	Specific binding	-1.13176
131	5-HT1A (h) (agonist radioligand)	Compound 50	Specific binding	-2.72291
1322	D2S (h) (agonist radioligand)	Compound 50	Specific binding	-3.92829
20	beta 2 (h) (antagonist radioligand)	Compound 50	Specific binding	-4.14768
66	NMDA (antagonist radioligand)	Compound 50	Specific binding	-5.13749
44	D1 (h) (antagonist radioligand)	Compound 50	Specific binding	-5.65806

Assay #	Assay Name	Compound	Response/Readout	% Inhibition
161	Ca2+ channel (L, dihydropyridine site) (antagonist radioligand)	Compound 50	Specific binding	-7.1517
36	CB1 (h) (agonist radioligand)	Compound 50	Specific binding	-8.2829
1208	H2 (h) (antagonist radioligand)	Compound 50	Specific binding	-9.62583
169	Na+ channel (site 2) (antagonist radioligand)	Compound 50	Specific binding	-10.2569
39	CCK1 (CCKA) (h) (agonist radioligand)	Compound 50	Specific binding	-39.6471

Table S18 | SafetyScreen44 data results for **KSN-L22 (Compound 46)** performed as off-target toxicity evaluation at 1.0E-05 M concentration. Results showing an inhibition of higher than 50% represent significant effects of the test compound. Results showing an inhibition between 25% and 50% are indicative of weak to moderate effects while those showing an inhibition lower than 25% are not considered significant.

Assay #	Assay Name	Study Number	Compound ID	% Inhibition
933	AR (h) (agonist radioligand)	FR095-0004874	Compound 46	4.24138
39	CCK1 (CCKA) (h) (agonist radioligand)	FR095-0004874	Compound 46	-2.03813
18	beta 1 (h) (agonist radioligand)	FR095-0004874	Compound 46	1.92061
469	GR (h) (agonist radioligand)	FR095-0004874	Compound 46	3.45577
118	mu (MOP) (h) (agonist radioligand)	FR095-0004874	Compound 46	6.53465
471	5-HT2A (h) (agonist radioligand)	FR095-0004874	Compound 46	-8.88055
132	5-HT1B (antagonist radioligand)	FR095-0004874	Compound 46	-16.7063
4173	COX1(h)	FR095-0004874	Compound 46	30.3185
439	5-HT transporter (h) (antagonist radioligand)	FR095-0004874	Compound 46	-16.9663
2338	alpha 1A (h) (antagonist radioligand)	FR095-0004874	Compound 46	11.5312
131	5-HT1A (h) (agonist radioligand)	FR095-0004874	Compound 46	-3.77016
93	M2 (h) (antagonist radioligand)	FR095-0004874	Compound 46	1.06575
4	A2A (h) (agonist radioligand)	FR095-0004874	Compound 46	53.628
54	ETA (h) (agonist radioligand)	FR095-0004874	Compound 46	-5.23641
1208	H2 (h) (antagonist radioligand)	FR095-0004874	Compound 46	-40.1709
166	KV channel (antagonist radioligand)	FR095-0004874	Compound 46	-4.64385
28	BZD (central) (agonist radioligand)	FR095-0004874	Compound 46	-44.8289
1322	D2S (h) (agonist radioligand)	FR095-0004874	Compound 46	-7.80669
20	beta 2 (h) (antagonist radioligand)	FR095-0004874	Compound 46	11.3086
52	dopamine transporter (h) (antagonist radioligand)	FR095-0004874	Compound 46	10.0257
13	alpha 2A (h) (antagonist radioligand)	FR095-0004874	Compound 46	4.4791
4186	COX2(h)	FR095-0004874	Compound 46	8.11129
1971	kappa (KOP) (agonist radioligand)	FR095-0004874	Compound 46	27.3513
95	M3 (h) (antagonist radioligand)	FR095-0004874	Compound 46	2.27343
114	delta (DOP) (h) (agonist radioligand)	FR095-0004874	Compound 46	2.15892
37	CB2 (h) (agonist radioligand)	FR095-0004874	Compound 46	0
161	Ca2+ channel (L, dihydropyridine site) (antagonist radioligand)	FR095-0004874	Compound 46	6.04874
159	V1a (h) (agonist radioligand)	FR095-0004874	Compound 46	-12.5662
1333	5-HT2B (h) (agonist radioligand)	FR095-0004874	Compound 46	5.37361
4077	PDE4D2 (h)	FR095-0004874	Compound 46	14.8003
44	D1 (h) (antagonist radioligand)	FR095-0004874	Compound 46	-15.4033
870	H1 (h) (antagonist radioligand)	FR095-0004874	Compound 46	5.56418
3029	N neuronal alpha 4beta 2 (h) (agonist radioligand)	FR095-0004874	Compound 46	-41.8108
66	NMDA (antagonist radioligand)	FR095-0004874	Compound 46	12.6124
443	MAO-A (antagonist radioligand)	FR095-0004874	Compound 46	-9.12972
4072	PDE3A (h)	FR095-0004874	Compound 46	7.50221

2906	Lck kinase (h)	FR095-0004874	Compound 46	-2.89115
4094	Potassium Channel hERG (human)- [3H] Dofetilide	FR095-0004874	Compound 46	-0.246078
169	Na+ channel (site 2) (antagonist radioligand)	FR095-0004874	Compound 46	23.6539
363	acetylcholinesterase (h)	FR095-0004874	Compound 46	32.9861
411	5-HT3 (h) (antagonist radioligand)	FR095-0004874	Compound 46	-4.70826
36	CB1 (h) (agonist radioligand)	FR095-0004874	Compound 46	1.85658
355	norepinephrine transporter (h) (antagonist radioligand)	FR095-0004874	Compound 46	-6.47059
91	M1 (h) (antagonist radioligand)	FR095-0004874	Compound 46	3.12943

Table S19 | Cytochrome P450 interaction profile of **KSN-L22 (Compound 46)** and **BL-7 (Compound 50)**.

Assay Number	Assay Name	Compound 46	Compound 50
2064	CYP1A inhibition (HLM, phenacetin substrate)	9.6	16.0408
2065	CYP2B6 inhibition (HLM, bupropion substrate)	-1.3	4.82131
1772	CYP2C19 inhibition (HLM, omeprazole substrate)	0.6	9.86547
4481	CYP2C8 inhibition (HLM, amodiaquine substrate)	3.1	13.3884
2066	CYP2C9 inhibition (HLM, diclofenac substrate)	12.8	16.7752
1838	CYP2D6 inhibition (HLM, dextromethorphan substrate)	0.0	3.80389
1770	CYP3A inhibition (HLM, midazolam substrate)	25.2	10.5178
1769	CYP3A inhibition (HLM, testosterone substrate)	6.5	6.31929

Table S20 | Extended toxicity data for **KSN-L22 (Compound 46)** tested in ICR 2 male mice. **a-c**) Effects of **Compound 46** during dose escalated maximum tolerated dose (MTD) study **d**) Mice body weights pre- and post-treatment with **Compound 46**.

a

WO# 1064175 (AB123754) — Strain: ICR (Male)

Compound	Route	Dose (mg/kg)	Time after 1st dosing – Response (death / test) 1 hr	6 hrs	24 hrs	48 hrs	72 hrs
Vehicle (4% DMSO/PBS)	PO	10 mL/kg	0/3	0/3	NA		
Compound 46	PO	100	0/3	0/3	NA		
Compound 46	PO	200	0/3	0/3	NA		
Compound 46	PO	400	0/3	0/3	NA		

b

WO# 1064175 (AB123754) — Strain: ICR (Male)

Compound	Route	Dose (mg/kg)	Time after 2nd dosing – Response (death / test) 1 hr	6 hrs	24 hrs	48 hrs	72 hrs
Vehicle (4% DMSO/PBS)	PO	10 mL/kg	0/3	0/3	NA		
Compound 46	PO	100	0/3	0/3	NA		
Compound 46	PO	200	0/3	0/3	NA		
Compound 46	PO	400	0/3	0/3	NA		

c

WO# 1064175 (AB123754) — Strain: ICR (Male)

Compound	Route	Dose (mg/kg)	Time after 3rd dosing – Response (death / test) 1 hr	2 hrs	24 hrs	48 hrs	72 hrs
Vehicle (4% DMSO/PBS)	PO	10 mL/kg	0/3	0/3	0/3	0/3	0/3
Compound 46	PO	100	0/3	0/3	0/3	0/3	0/3
Compound 46	PO	200	0/3	0/3	0/3	0/3	0/3
Compound 46	PO	400	0/3	0/3	0/3	0/3	0/3

d

WO# 1064175 (AB123754)

Compound	Route	Dose (mg/kg)	N	B.W. Pre-dose	B.W. 72 hrs after final dose
Vehicle (4% DMSO/PBS)	PO	10 mL/kg	1	23	25
			2	23	26
			3	24	25
Compound 46	PO	100	1	24	25
			2	24	27
			3	25	27
Compound 46	PO	200	1	24	26
			2	24	27

			3	23	26
Compound 46	PO	400	1	21	26
			2	20	22
			3	22	24

Table S21 | Extended toxicity data for **BL-7** (Compound 50) tested in male ICR mice. **a-c**) Effects of **Compound 50** during dose escalated maximum tolerated dose (MTD) study. **d**) Mice body weights pre- and post-treatment with **Compound 50**.

WO# 1064183 (AB129420) Strain: ICR (Male)

a

Compound	Route	Dose (mg/kg)	Time after 1st dosing				
			Response (death / test)				
			1 hr	6 hrs	24 hrs	48 hrs	72 hrs
Vehicle (4% DMSO/PBS)	PO	10 mL/kg	0/3	0/3	NA		
Compound 50	PO	100	0/3	0/3	NA		
Compound 50	PO	200	0/3	0/3	NA		
Compound 50	PO	400	0/3	0/3	NA		

WO# 1064183 (AB129420) Strain: ICR (Male)

b

Compound	Route	Dose (mg/kg)	Time after 2nd dosing				
			Response (death / test)				
			1 hr	6 hrs	24 hrs	48 hrs	72 hrs
Vehicle (4% DMSO/PBS)	PO	10 mL/kg	0/3	0/3	NA		
Compound 50	PO	100	0/3	0/3	NA		
Compound 50	PO	200	0/3	0/3	NA		
Compound 50	PO	400	0/3	0/3	NA		

WO# 1064183 (AB129420) Strain: ICR (Male)

c

Compound	Route	Dose (mg/kg)	Time after 3rd dosing				
			Response (death / test)				
			1 hr	2 hrs	24 hrs	48 hrs	72 hrs
Vehicle (4% DMSO/PBS)	PO	10 mL/kg	0/3	0/3	0/3	0/3	0/3
Compound 50	PO	100	0/3	0/3	0/3	0/3	0/3
Compound 50	PO	200	0/3	0/3	0/3	0/3	0/3
Compound 50	PO	400	0/3	0/3	0/3	0/3	0/3

WO# 1064183 (AB129420) Strain: ICR (Male)

d

Compound	Route	Dose (mg/kg)	N	B.W.	
				Pre-dose	72 hrs after final dose
Vehicle (4% DMSO/PBS)	PO	10 mL/kg	1	24	27
			2	25	28
			3	25	29
Compound 50	PO	100	1	24	24
			2	23	24
			3	24	25
Compound 50	PO	200	1	23	25
			2	24	28
			3	23	25
Compound 50	PO	400	1	20	25
			2	20	23
			3	21	23
